# Exercise-induced changes in systemic inflammatory biomarkers in overweight and obese populations: a bibliometric analysis and umbrella review of meta-analyses

**DOI:** 10.3389/fimmu.2026.1838118

**Published:** 2026-05-20

**Authors:** Lei Chen, Aichun Li, Xiangyang Zhou, Wenhao Chen, Yanyan Zhou, Rui Wang, Yujia Kou, Junlai Zhou

**Affiliations:** 1 School of Physical Education, Hainan Normal University, Haikou, China; 2 Faculty of Science, The University of Western Australia, Perth, WA, Australia; 3 School of Education, Hainan Normal University, Haikou, China; 4 School of Physical Education and Health, East China Normal University, Shanghai, China

**Keywords:** obesity, exercise, inflammation, umbrella review, bibliometric analysis

## Abstract

**Background:**

Systemic chronic low-grade inflammation contributes to obesity-related comorbidities. Exercise is widely recommended as a non-pharmacological strategy, but evidence regarding its biomarker-specific anti-inflammatory effects remains fragmented. This study integrated bibliometric analysis with an umbrella review to map global trends and evaluate the certainty of evidence regarding exercise-induced immunomodulation in overweight and obese populations.

**Methods:**

Bibliometric data from 2005 to 2025 were retrieved from the Web of Science Core Collection to map publication trends, knowledge structure, and emerging research topics. Concurrently, a comprehensive search across five databases through December 1, 2025 identified pairwise meta-analyses of randomized controlled trials (RCTs). Methodological quality and primary-study overlap were evaluated using A Measurement Tool to Assess Systematic Reviews 2 (AMSTAR-2), Risk of Bias in Systematic Reviews (ROBIS), and the corrected covered area (CCA). Pooled standardized mean differences (SMDs) were re-estimated using random-effects models, and evidence certainty was appraised using the Grading of Recommendations Assessment, Development and Evaluation (GRADE) framework.

**Results:**

The bibliometric analysis of 12,101 records revealed a shift in research emphasis toward mechanism-oriented topics, including cellular immune crosstalk and inflammatory pathway regulation. The umbrella review included 25 meta-analyses encompassing 342 RCTs (>30,000 participants). Class II evidence supported exercise-related reductions in C-reactive protein (CRP) (SMD = -0.29, 95% confidence interval [CI]: -0.35 to -0.23), interleukin-1β (IL-1β) (SMD = -0.43, 95% CI: -0.70 to -0.17), and interleukin-8 (IL-8) (SMD = -0.32, 95% CI: -0.50 to -0.14). The IL-1β estimate demonstrated improved consistency following sensitivity analysis. Tumor necrosis factor-α (TNF-α) showed Class III evidence, whereas interleukin-6 (IL-6), leptin, adiponectin, and interleukin-18 (IL-18) were supported by Class IV evidence, mainly because of substantial heterogeneity, publication bias, limited information size, or high review overlap. Interleukin-10 (IL-10) showed no significant systemic response (SMD = 0.18, 95% CI: -0.10 to 0.45; Class V).

**Conclusions:**

Exercise is associated with favorable changes in several systemic inflammatory biomarkers in overweight and obese populations, with the most consistent evidence for CRP, IL-1β, and IL-8. Nevertheless, evidence maturity remains uneven across biomarkers, underscoring the need for better-standardized and adequately powered randomized trials to support precision exercise prescription.

**Systematic review registration:**

https://www.crd.york.ac.uk/prospero/, identifier CRD420251246180.

## Graphical Abstract

**Figure:**
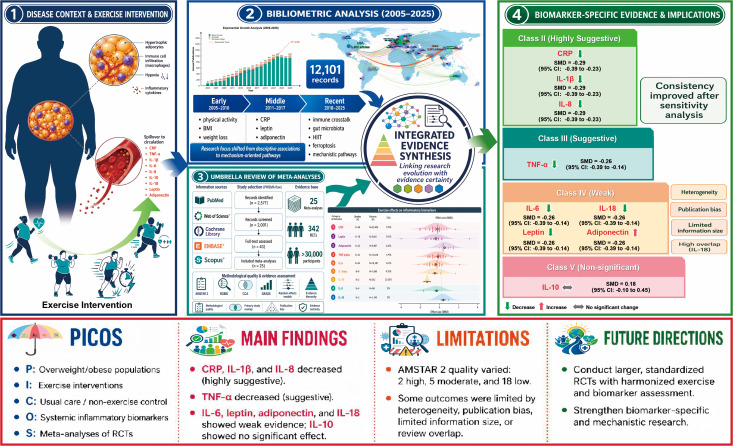
A bibliometric analysis and umbrella review evaluating the certainty of evidence for exercise-induced immunomodulation in overweight and obese populations.

## 1 Introduction

Obesity is a complex, multifactorial chronic disease in which chronic low-grade inflammation (CLGI), often referred to as metaflammation, plays a central pathophysiological role (1, 2). During the pathological expansion of adipose tissue, adipocyte hypertrophy, local hypoxia, endoplasmic reticulum stress, and impaired lipid buffering promote immune-cell infiltration and a shift toward pro-inflammatory macrophage phenotypes (3). This immunometabolic remodeling is accompanied by increased secretion of inflammatory cytokines and acute-phase mediators, including tumor necrosis factor-α (TNF-α), interleukin-6 (IL-6), interleukin-1β (IL-1β), and C-reactive protein (CRP), together with adipokine dysregulation characterized by increased leptin and reduced adiponectin signaling (4). These alterations interfere with insulin receptor signaling, impair mitochondrial function, and contribute to the development of insulin resistance, type 2 diabetes (T2D), cardiovascular diseases, and other obesity-related complications (5, 6). Therefore, attenuating obesity-associated CLGI has become an important therapeutic target in metabolic disease prevention and management.

Exercise is widely recommended as a scalable non-pharmacological strategy for the management of overweight and obesity (7). Its anti-inflammatory potential may involve both adiposity-dependent and adiposity-independent mechanisms. On the one hand, regular exercise increases energy expenditure, reduces visceral adiposity, and alleviates adipose tissue stress, thereby limiting the upstream drivers of inflammatory activation (8). On the other hand, exercise induces coordinated adaptations across skeletal muscle, adipose tissue, liver, and the vascular system. Contracting skeletal muscle releases myokines that participate in muscle–adipose–immune crosstalk, while exercise training may improve mitochondrial oxidative capacity, endothelial function, antioxidant defense, and insulin sensitivity (9–11). These adaptations may downregulate pro-inflammatory signaling and support a more favorable cytokine and adipokine profile. Importantly, the inflammatory interpretation of some biomarkers is context-dependent. IL-6, for example, may act as a chronically elevated pro-inflammatory mediator in obesity but also as an exercise-induced myokine involved in metabolic regulation and anti-inflammatory cascades (12, 13). This biological complexity indicates that the immunomodulatory effects of exercise cannot be inferred from single biomarkers or isolated trials alone.

Despite strong biological plausibility, clinical evidence regarding the anti-inflammatory efficacy of exercise in overweight and obese populations remains fragmented. Randomized controlled trials (RCTs) and meta-analyses have reported inconsistent findings across inflammatory biomarkers: some syntheses indicate significant reductions in CRP, TNF-α, IL-6, or improvements in adipokine profiles after exercise interventions, whereas others report non-significant or highly heterogeneous results (14). In addition, it remains debated whether changes in inflammatory markers are directly attributable to exercise-induced immunometabolic adaptations or are primarily mediated by concurrent weight loss and changes in adiposity (15). Existing meta-analyses also differ in eligibility criteria, population characteristics, exercise modality classification, intervention duration, biomarker selection, statistical models, and handling of heterogeneity. Moreover, few previous syntheses have simultaneously examined methodological quality, risk of bias, publication bias, and overlap of primary RCTs across reviews (16–18). These limitations make it difficult to determine which inflammatory biomarkers are supported by credible evidence and which findings remain uncertain.

Bibliometric analysis offers a useful approach for mapping the intellectual structure, research hotspots, and emerging frontiers of a rapidly expanding field (19, 20). A recent bibliometric study by Wan et al. (2024) (21) examined the molecular mechanisms of exercise in metabolic syndrome and provided valuable insights into research trends and collaborative networks in this broader cardiometabolic condition. However, that study focused on metabolic syndrome as a composite disease context and did not specifically evaluate systemic inflammatory biomarkers in overweight and obese populations, nor did it assess the certainty of evidence from meta-analyses of RCTs. Therefore, an important gap remains between macro-level knowledge mapping and evidence-based evaluation of exercise-induced inflammatory biomarker responses.

To address this gap, the present study adopts a complementary two-stage evidence synthesis that integrates bibliometric analysis with an umbrella review. The bibliometric component maps publication trends, collaboration networks, intellectual structure, and emerging topics in research on exercise and inflammatory biomarkers among overweight and obese populations. In parallel, the umbrella review re-evaluates published meta-analyses of RCTs, assesses methodological quality and primary-study overlap, and grades the certainty of evidence for each inflammatory biomarker using established appraisal frameworks (22, 23). Thus, the bibliometric analysis addresses how the field has evolved and where it is moving, whereas the umbrella review addresses how credible and clinically actionable the accumulated intervention evidence is. By linking research trends with graded evidence certainty, this study aims to provide an integrated evidence base for future mechanistic research and more precise exercise recommendations for overweight and obese populations.

## 2 Methods

### 2.1 Bibliometric methods

To map the global research landscape on the effects of exercise on inflammation in overweight and obese populations, bibliometric data were retrieved from the Web of Science Core Collection (WoSCC), a widely used database for bibliometric studies because of its standardized indexing and comprehensive citation records (24). The search was performed by a single investigator (L.C.) on January 3, 2026, and covered publications from January 1, 2005, to December 31, 2025. The search strategy was developed specifically for WoSCC and used topic-based retrieval with Boolean operators and free-text terms related to “exercise”, “overweight/obesity”, and “inflammation” (**Supplementary Table 1**). Only original articles published in English were included, and retracted publications were excluded. Full records and cited references were exported in plain text format for subsequent analysis. A complementary multi-tool framework was then used to capture different dimensions of the bibliometric landscape: Bibliometrix was used for descriptive bibliometric indicators and thematic evolution, VOSviewer for collaboration and keyword co-occurrence networks, CiteSpace for co-citation analysis and citation burst detection, and Scimago Graphica for geographic visualization of international collaboration (25–28).

### 2.2 Umbrella review methods

This study adhered to the Preferred Reporting Items for Systematic Reviews and meta-analyses (PRISMA) guidelines (29) and was prospectively registered with the International Prospective Register of Systematic Reviews (PROSPERO) under registration number CRD420251246180.

#### 2.2.1 Protocol and search strategy

A comprehensive systematic search was independently conducted by two investigators (L.C. and W.C.) in PubMed, Web of Science, Cochrane Library, EMBASE, and Scopus from database inception to December 1, 2025; the final search was completed on December 3, 2025. The search strategy was developed according to the PICOS framework and adapted to the indexing structure and search syntax of each database, using controlled vocabulary where applicable (e.g., MeSH in PubMed and Emtree in EMBASE) together with free-text terms and Boolean operators related to “overweight/obesity,” “exercise,” “inflammation,” and “meta-analysis.” Database-specific search strings are provided in **Supplementary Tables S2-S6**.

#### 2.2.2 Eligibility criteria

Eligibility criteria were defined according to the PICOS framework (1): Population: eligible populations included adults with overweight or obesity, defined as BMI ≥25 kg/m² according to WHO adult BMI criteria (30), as well as children and adolescents with overweight or obesity, defined as age- and sex-specific BMI-for-age at or above the 85th percentile according to ISPAD Clinical Practice Consensus Guidelines (31). Populations with obesity-related comorbidities were also eligible (2). Intervention: any modality of exercise intervention (3). Comparator: non-exercise controls or other comparison groups defined in the eligible reviews (4). Outcomes: quantitative measures of inflammatory biomarkers, including acute phase proteins (CRP), adipokines (leptin and adiponectin), pro-inflammatory cytokines (TNF-α, IL-6, IL-1β, and IL-18), chemokines (IL-8), and anti-inflammatory cytokines (IL-10) (5). Study design: systematic reviews with pairwise meta-analyses of randomized controlled trials (RCTs). Narrative reviews, observational studies, studies with insufficient data for effect-size re-analysis, and network meta-analyses were excluded from the quantitative synthesis, as the latter incorporate both direct and indirect evidence and are not directly comparable with the pairwise effect estimates re-estimated in this umbrella review. Only English-language publications were included.

#### 2.2.3 Study selection and data extraction

Following the retrieval process, all records were imported into EndNote X9 (Clarivate Analytics) for duplicate removal and management. The selection process consisted of two phases: an initial title/abstract screening followed by a full-text review, both performed independently by two investigators (X.Z. and Y.Z.). Any discrepancies regarding study eligibility were resolved through consensus or arbitration by a senior investigator (A.L.). Data extraction was finalized on December 30, 2025, utilizing a standardized, pre-piloted form. Extracted data included key study characteristics (e.g., first author, year, sample size), intervention details (type, duration, intensity), and quantitative synthesis results, specifically the pooled effect sizes (ES), 95% confidence intervals (CI), and heterogeneity statistics (*I*²) for immune and inflammatory biomarkers.

#### 2.2.4 Methodological quality assessment

Methodological rigor and risk of bias were strictly appraised using a dual-instrument approach. The AMSTAR-2 tool (32) was employed to scrutinize critical domains including protocol adherence (PICOS), search comprehensiveness, and the validity of data synthesis. Complementarily, the ROBIS tool (33) was utilized to specifically identify bias introduced during the review process, focusing on the transparency of study selection and extraction. Based on this comprehensive evaluation, included meta-analyses were stratified into three quality tiers: High Quality (satisfying high AMSTAR-2 standards with low ROBIS risk), Moderate Quality, and Low Quality (34). The appraisal process was conducted independently by two investigators (W.C. and R.W.), with discrepancies adjudicated by a senior expert (J.Z.).

#### 2.2.5 Assessment of primary study overlap

To assess overlap in primary studies across the included meta-analyses and minimize potential double-counting, the Corrected Covered Area (CCA) index was calculated using the ccaR package in R (version 4.5.2) (35). Overlap was categorized as slight (0–5%), moderate (6–10%), high (11–15%), or very high (>15%) according to established recommendations for overviews of reviews (36). For outcomes with CCA > 5%, overlap was further interpreted in conjunction with review quality, recency, and concordance of findings to determine whether repeated inclusion of the same primary trials could materially affect the summary conclusions.

#### 2.2.6 Grading of evidence certainty

Evidence certainty was evaluated using a modified GRADE approach adapted for umbrella reviews (37). Because the unit of inclusion was the meta-analysis rather than the individual trial, the five GRADE domains were operationalized at the review/outcome level: risk of bias was judged using AMSTAR-2 and ROBIS; inconsistency from between-review heterogeneity and direction of effects; indirectness from the correspondence of populations, interventions, comparators, and outcomes to the review question; imprecision according to the optimal information size principle, with a total sample size <500 considered insufficient for continuous outcomes (23); and publication bias from funnel plots, Egger’s regression test, and trim-and-fill results when available. Following the initial GRADE rating (High, Moderate, Low, or Very Low), certainty was further translated into a five-tier hierarchy for umbrella-review presentation: Class I (Convincing), Class II (Highly Suggestive), Class III (Suggestive), Class IV (Weak), and Class V (Non-significant) (38).

#### 2.2.7 Data analysis

All statistical analyses were performed using R software version 4.5.2 utilizing the meta and metafor packages for data synthesis and ggplot2 for visualization (39, 40). Pooled effect sizes were expressed as standardized mean differences (SMDs) with 95% confidence intervals (CIs) and synthesized using restricted maximum likelihood (REML) random-effects models (41). The absolute magnitude of SMDs was interpreted as trivial (<0.20), small (0.20–0.49), moderate (0.50–0.79), and large (≥0.80) (42). Statistical heterogeneity was assessed by Cochran’s Q test with a significance threshold of P < 0.10 and quantified using the I² statistic where values greater than 50% indicated substantial heterogeneity while τ² estimated between-study variance (43). Pre-specified subgroup analyses stratified by age group, health status, intervention modality, duration, and sample size were conducted to investigate potential sources of heterogeneity. The robustness of summary estimates was evaluated through standard leave-one-out sensitivity analysis to identify individual studies with significant influence, followed by *post-hoc* sensitivity analyses to investigate sources of substantial heterogeneity (*I*^2^ > 50%) (44). Publication bias was examined using funnel plots and Egger’s regression test where a P value less than 0.05 indicated significant asymmetry prompting the implementation of the trim-and-fill method to adjust effect size estimates (45).

## 3 Results

### 3.1 Literature search and selection

**Figure 1:**
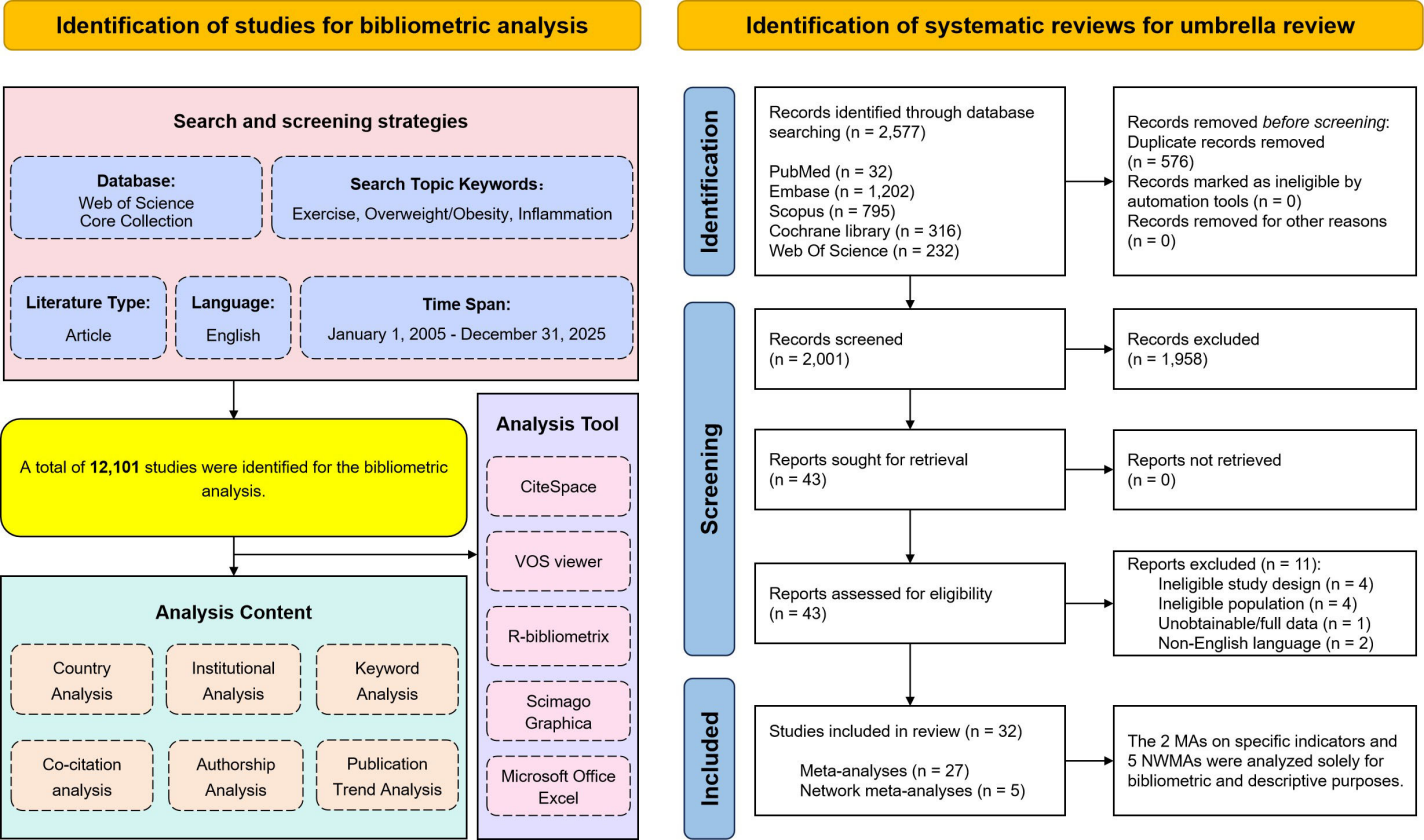
Flowchart of the literature screening process for the bibliometric and umbrella review.

A dual-trajectory search strategy was implemented. For the umbrella review, systematic searches across five databases yielded 43 full-text articles assessed for eligibility, of which 32 met inclusion criteria—comprising 27 pairwise meta-analyses (17, 18, 46–70) and 5 network meta-analyses (71–75). The 5 network meta-analyses and 2 meta-analyses focusing on single outcomes (69, 70) were included for descriptive appraisal but excluded from quantitative synthesis due to methodological heterogeneity. Concurrently, a dedicated search in WoSCC retrieved 12,101 records for bibliometric mapping. The screening procedure is detailed in the PRISMA flow diagram (**Figure 1**); excluded studies are summarized in **Supplementary Table 7**.

### 3.2 Bibliometric findings

#### 3.2.1 Publication trends and global contributions

**Figure 2:**
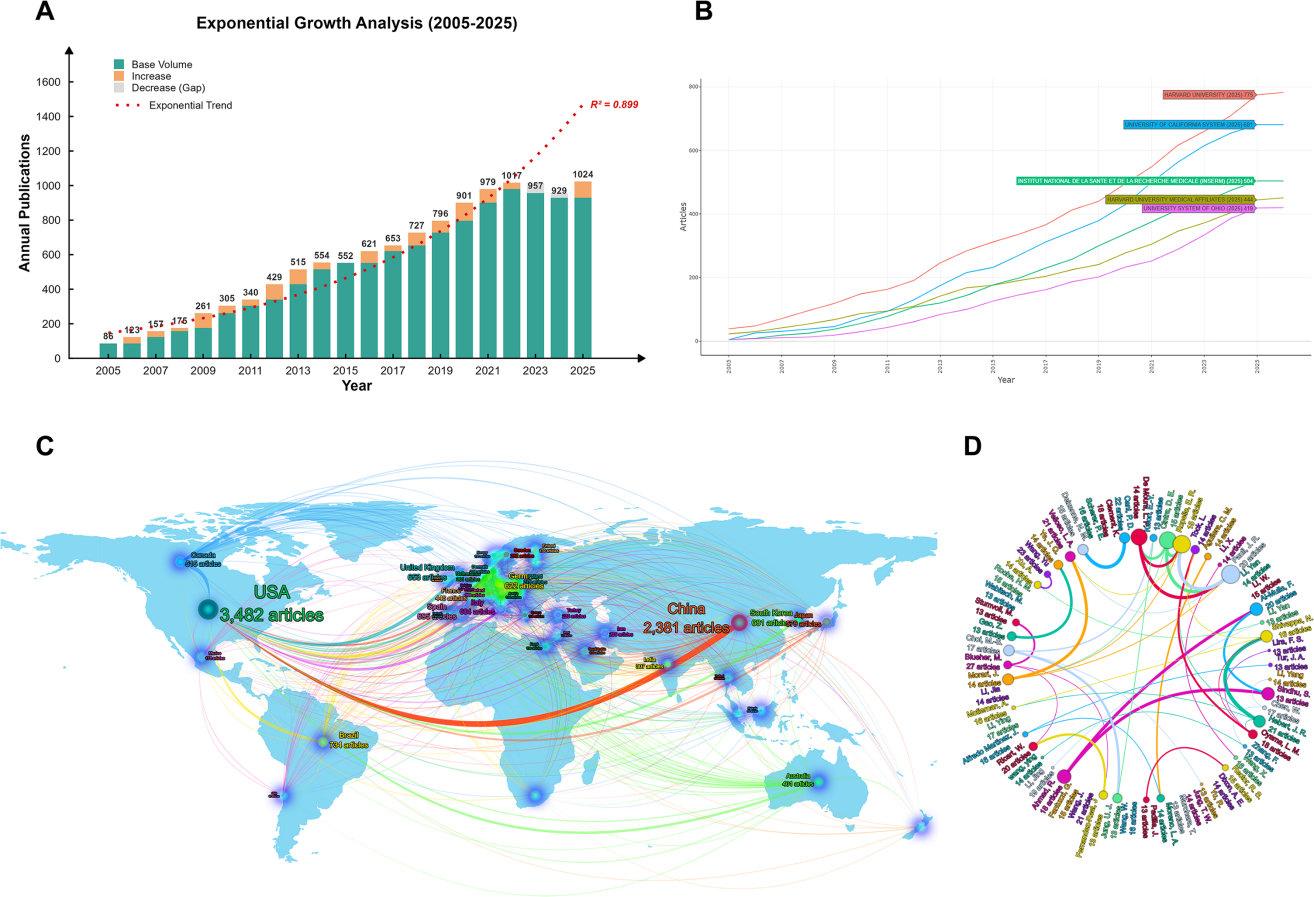
Bibliometric analysis of publication outputs and collaboration patterns: **(A)** annual trend of publication volume, **(B)** country-level contributions and collaboration network, **(C)** leading productive institutions/organizations, and **(D)** chord diagram visualizing institutional collaborations.

The annual number of publications on exercise and inflammatory markers in overweight/obese populations increased steadily between 2005 and 2025 (*R²* = 0.899; **Figure 2A**). The bibliometric dataset comprised 72,084 authors from 10,948 institutions across 125 countries. At the country level, the United States contributed the largest number of publications (n = 3,482) and occupied a central position in the international collaboration network (**Figure 2B**). At the institutional level, Harvard University had the highest publication output (n = 775; **Figure 2C**). Highly cited work from this institution frequently addressed exercise-related immunometabolic mechanisms, including myokine-mediated regulation of inflammation (76, 77). Matthias Blüher was the most productive author in this field (**Figure 2D**). His representative studies have focused on adipose tissue inflammation, oxidative stress, and adipokine-related responses in obesity (78–80).

#### 3.2.2 Keyword co-occurrence and research frontiers

**Figure 3:**
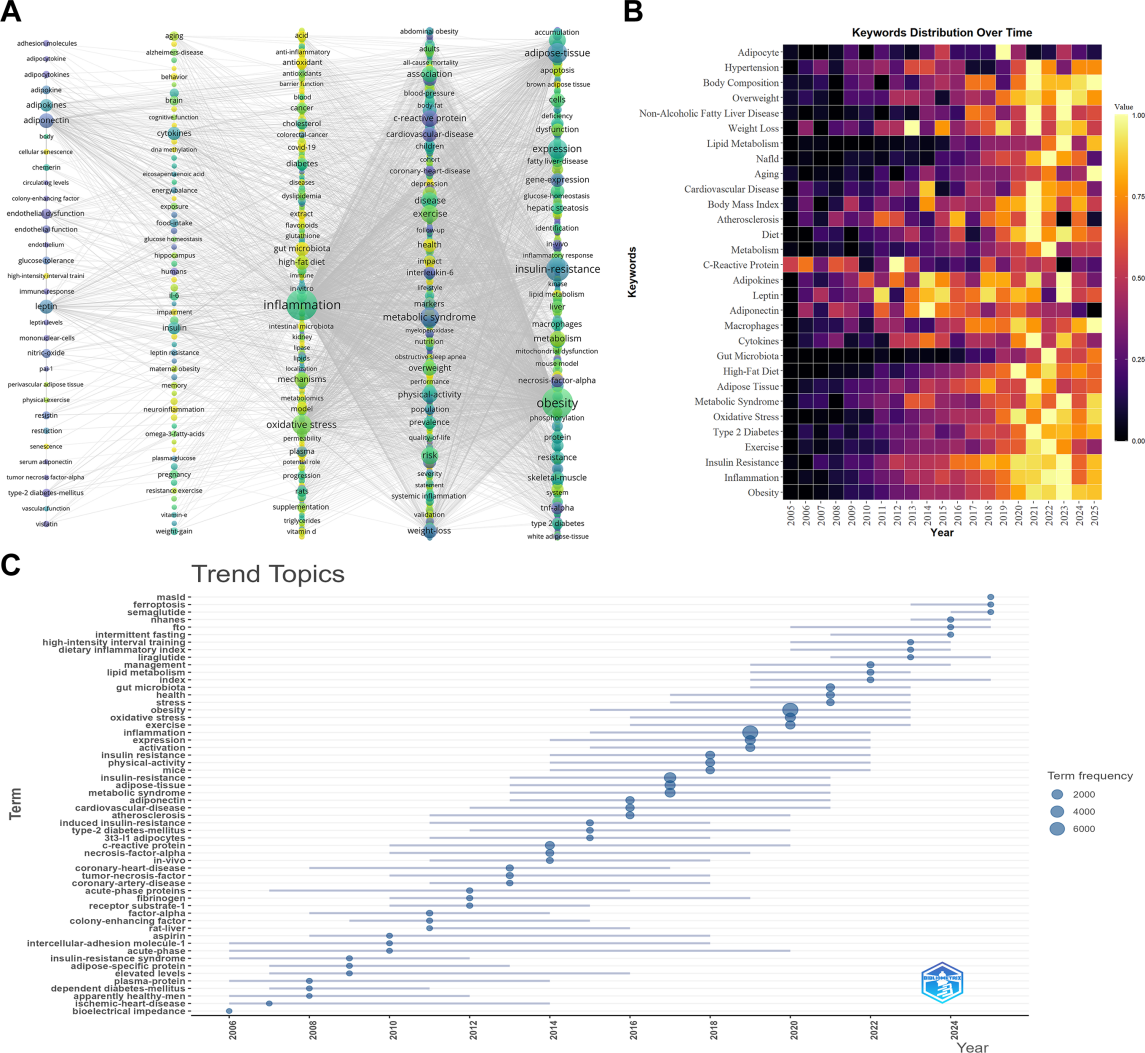
Keyword analysis: **(A)** co-occurrence network map, **(B)** heatmap of keyword prominence, and **(C)** evolution of trending topics.

Keyword co-occurrence, heatmap, and trend analyses indicated a shift in research emphasis over time (**Figure 3**). During 2005–2015, highly frequent terms were mainly related to physical activity, BMI, and weight loss. Between 2015 and 2020, keywords increasingly reflected inflammation–metabolism interactions, with greater attention to biomarkers such as CRP, adiponectin, and leptin. In 2021–2025, frequently emerging terms included gut microbiota, MASLD, ferroptosis, and HIIT, suggesting increasing interest in molecular mechanisms and more specific intervention strategies.

**Figure 4:**
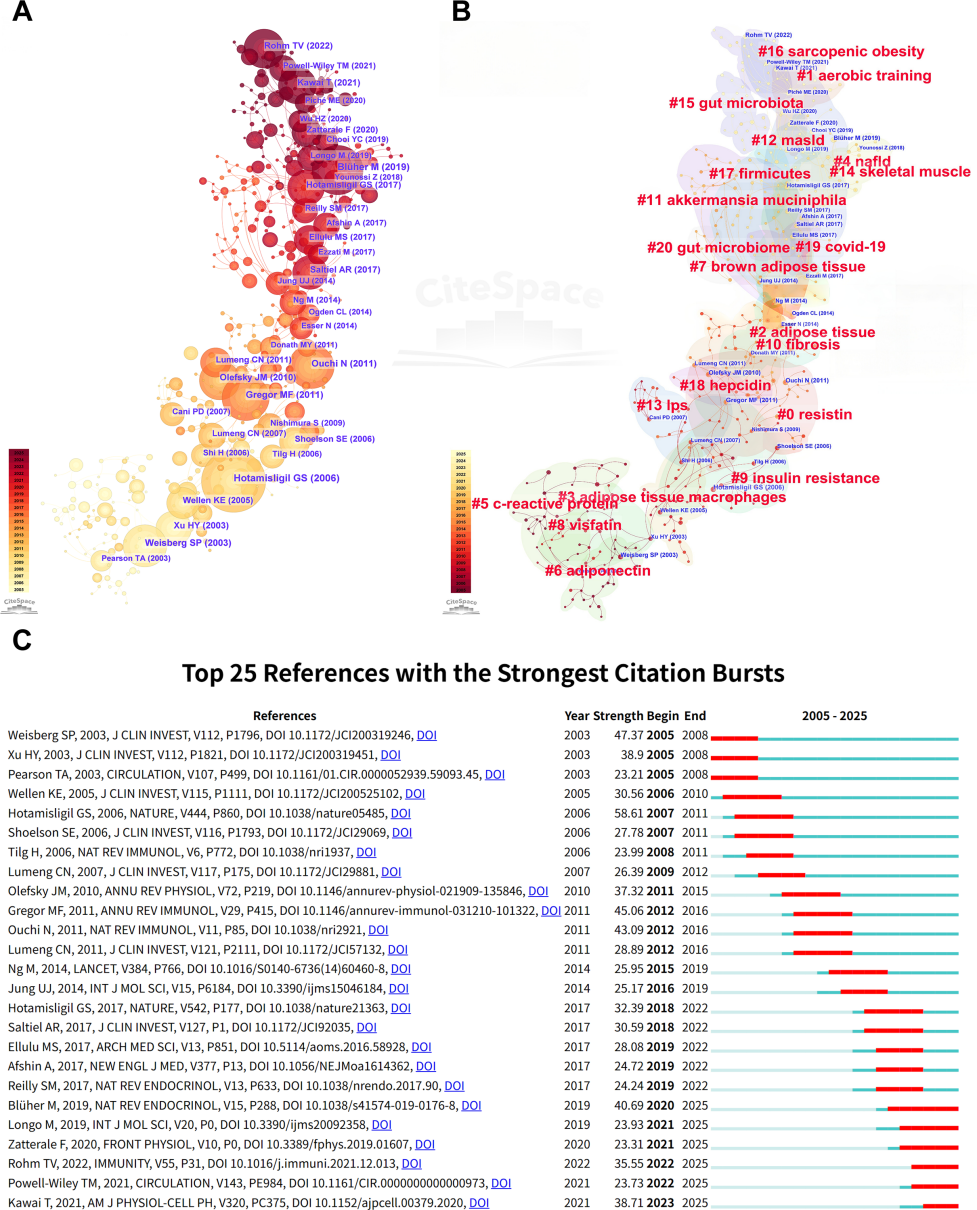
Co-citation network analysis: **(A)** network visualization, **(B)** thematic clusters, and **(C)** top 25 references with the strongest citation bursts.

The co-citation timeline further illustrated the development of the field’s intellectual structure (**Figure 4**). Early co-citation clusters were centered on obesity, inflammation, and insulin resistance, including landmark studies by Weisberg et al. (2003) (81), Xu et al. (2003) (82), and Hotamisligil (2006) (83). Subsequent clusters showed greater emphasis on adipokines and cardiometabolic regulation, represented by Olefsky and Glass (2010) (84). More recent clusters were associated with cellular remodeling and immune crosstalk, including Kawai et al. (2021) (85). Together, these patterns suggest a gradual transition from broad descriptive associations toward more mechanism-oriented research.

**Figure 5:**
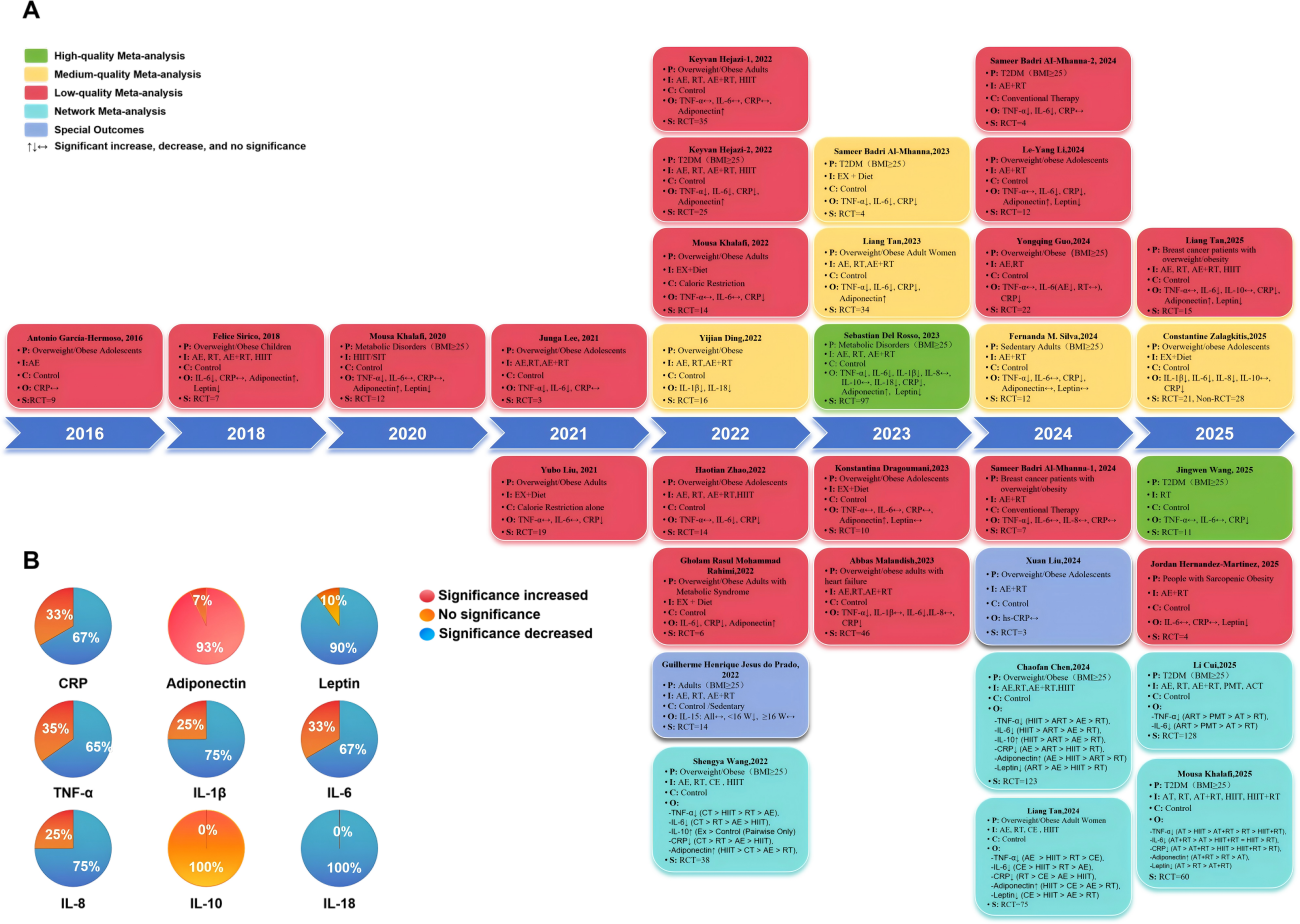
Umbrella review evidence statistics and timeline. **(A)** Quality assessment and PICOS characteristics of included studies, **(B)** Proportion of statistically significant outcomes across all meta-analyses.

Despite the expansion of the literature and the increasing focus on mechanisms, substantial discordance remained across the retrieved meta-analyses (**Figure 5**). For CRP, TNF-α, and IL-6, only 65.2% to 70.4% of meta-analyses reported statistically significant pooled effects, whereas adipokines such as adiponectin (92.9%) and leptin (90.0%) showed greater directional consistency. No consistent evidence of a beneficial IL-10 response was identified. In addition, prior network meta-analyses yielded inconsistent rankings of exercise modalities. These findings indicate that, despite growing research activity, the strength and consistency of evidence across inflammatory biomarkers remain variable, supporting the need for a formal umbrella review.

### 3.3 Umbrella review results

#### 3.3.1 Characteristics and quality of included meta-analyses

Twenty-five meta-analyses encompassing 342 original RCTs were included; the reported participant totals across reviews exceeded 30,000. Study populations spanned general adults, children/adolescents (n = 7) (17, 46, 54, 59, 65, 67, 68), and patients with comorbidities, including T2D (47, 52, 56, 61), breast cancer (49, 51), metabolic syndrome (57, 66). Methodological quality varied across the included meta-analyses, with two reviews rated as high quality, five as moderate quality, and eighteen as low quality. The main limitations were absent protocol registration and incomplete reporting of excluded studies. These assessments are summarized in **Figure 5A** and detailed in **Supplementary File 4**. Comprehensive study characteristics are provided in **Table 1**.

**Table 1:** Characteristics of systematic reviews and meta-analyses included in the umbrella review.

First author year	Literature search time frame	No. of studies	Duration time	Number of participants (n) INT/CON	Interventions	Comparisons	Subject of intervention	Mean age range (years)	Outcome	Risk of bias assessment
Zalagkitis 2025 (46)	Inception to February 2025	49	2 weeks - 30 months	1990/841	EX + Diet	Usual care	Overweight/obese Adolescents	4.6 - 17.0	IL-1β↓, IL-6↓, IL-8↓, IL-10↔, CRP↓	Cochrane RoB tool; RTI-IB; GRADE
Wang 2025 (47)	Inception to May 20, 2025	11	8 - 52 weeks	502/441	RT	Usual care	Middle-aged and elderly patients with T2DM (BMI ≥ 25)	51.3 - 70.5	TNF-α↔, IL-6↔, CRP↓	Cochrane RoB 2.0; GRADE
Hernandez-Martinez 2025 (48)	Inception to July 2025	4	8 - 24 weeks	157/153	AE + RT	Daily activities	Sarcopenic obese adults (BMI ≥ 25)	44.1 - 81.4	IL-6↔, CRP↔, Leptin↓	RoB 2.0; TESTEX; GRADE
Tan 2025 (49)	Inception to November 2024	15	12 weeks - 12 months	562/554	AE; RT; AE + RT; HIIT	Usual care	Breast cancer patients with overweight/obesity	48.4 - 59.8	TNF-α↔, IL-6↓, IL-10↔, CRP↓, Adiponectin↑, Leptin↓	Cochrane RoB tool
Silva 2024 (50)	Inception to December 2022	12	8–24 weeks	317/212	AE + RT	Usual care	Sedentary Adults(BMI ≥ 25)	18.4 - 58.7	TNF-α↓, IL-6↔, CRP↓, Adiponectin↔, Leptin↔	Cochrane RoB 2.0
Li 2024 (17)	Inception to October 1, 2023	12	4 weeks - 6 months	243/198	AE + RT	Usual care	Overweight/obese Adolescents	10.1 - 16.9	TNF-α↔, IL-6↓, CRP↓, Adiponectin↑, Leptin↓	Cochrane RoB tool
Al-Mhanna-1 2024 (51)	Inception to January 8, 2024	7	8–52 weeks	220/205	AE + RT	Usual care	Breast cancer patients with overweight/obesity	48.5 - 57.2	TNF-α↓, IL-6↔, IL-8↔, CRP↔	Cochrane RoB tool; GRADE
AL-Mhanna-2 2024 (52)	Inception to May 1, 2023	4	8–60 weeks	112/83	AE + RT	Usual care	T2DM (BMI ≥ 25)	29.3 - 58.5	TNF-α↓, IL-6↓, CRP↔	Cochrane RoB tool; GRADE
Guo, 2024 (18)	Inception to December 10, 2023	22	6–52 weeks	753/357	AE; RT	Usual care	Overweight/Obese	10.8 - 60.9	TNF-α↔, IL-6(AE↓, RT↔), CRP↓	Cochrane RoB tool
Malandish 2023 (53)	Inception to August 31, 2022	46	3 weeks - 24 months	1719/1489	AE; RT; AE + RT	Usual care	Overweight/obese HF patients	48.0 - 77.5	TNF-α↓, IL-1β↔, IL-6↓,IL-8↔, CRP↓	PEDro scale
Dragoumani 2023 (54)	Inception to November 2022	10	3–12 months	555	EX + Diet	Usual care	Overweight/Obese Adolescents	8.7 - 15.8	TNF-α↔, IL-6↔, CRP↔, Adiponectin↑, Leptin↔	NR
Tan 2023 (55)	Inception to May 2023	34	8 weeks - 12 months	1252/991	AE; RT; AE + RT	Usual care	Overweight/Obese Adult Women	46.7 - 88.9	TNF-α↓, IL-6↓, CRP↓, Adiponectin↑	Cochrane RoB tool
Al-Mhanna 2023 (56)	Inception to October 20, 2023	4	8 weeks - 24 months	106/71	EX + Diet	Usual care	T2DM(BMI ≥ 25)	41.5 - 56.2	TNF-α↓, IL-6↓, CRP↓, Adiponectin↑	Cochrane RoB tool; GRADE
Del Rosso 2023 (57)	January 2000 to July 2022	97	≥12 weeks	8263	AE; RT; AE + RT	Usual care	Metabolic Disorders (BMI ≥ 25)	19.2 - 76.8	TNF-α↓, IL-6↓, IL-1β↓, IL-8↔, IL-10↔, IL-18↓, CRP↓, Adiponectin↑, Leptin↓	Downs and Black checklist
Rahimi 2022 (58)	Inception to December 20, 2020	6	6–12 months	638/608	EX + Diet	Usual care	Overweight/Obese Adults with Metabolic Syndrome	49.0 - 67.0	IL-6↓, CRP↓, Adiponectin↑	TESTEX scale
Zhao 2022 (59)	Inception to January 2022	14	6–24 weeks	496/485	AE; RT; AE + RT; HIIT	Daily activities	Overweight/Obese Adolescents	12.1 - 17.0	TNF-α↔, IL-6↓, CRP↓	Cochrane RoB tool
Hejazi-1 2022 (60)	Inception to December 15, 2021	35	6–24 weeks	1741/1011	AE; RT; AE + RT; HIIT	Usual care	Overweight/Obese Adults	18.0 - 70.3	TNF-α↔, IL-6↔, CRP↔, Adiponectin↑	TESTEX scale
Hejazi-2 2022 (61)	Inception to January 2022	25	8–52 weeks	679/578	AE; RT; AE + RT; HIIT	Usual care	T2DM (BMI ≥ 25)	41.2 - 66.0	TNF-α↓, IL-6↓, CRP↓, Adiponectin↑	TESTEX scale
Khalafi 2022 (62)	Inception to April 2020	14	6 weeks - 18 months	926	EX + Diet	Calorie restriction	Overweight/Obese Adults	13.1 - 70.0	TNF-α↔, IL-6↔, CRP↓	Modified PRISMA checklist (8 items)
Ding 2022 (63)	Inception to June 12, 2021	16	4 weeks - 6 months	126/226	AE; RT; AE + RT	Usual care	Overweight/Obese	13.9 - 71.6	IL-1β↓, IL-18↓	Cochrane RoB tool; GRADE
Liu 2021 (64)	Inception to April 2021	19	8–72 weeks	688/621	EX + Diet	Calorie restriction	Overweight/Obese Adults	28.8 - 70.0	TNF-α↔, IL-6↔, CRP↓	Cochrane RoB tool; MINORS
Lee 2021 (65)	January 1990 to August 2020	3	≤12 weeks	265/254	AE; RT; AE + RT	Usual care	Overweight/Obese Adolescents	10.2 - 13.3	TNF-α↓, IL-6↓, CRP↔	NR
Khalafi 2020 (66)	Inception to January 2020	12	≤12 weeks	421	HIIT/SIT	Usual care	Metabolic Disorders (BMI ≥ 25)	14.2 - 55.7	TNF-α↓, IL-6↔, CRP↔, Adiponectin↑, Leptin↓	Modified PRISMA checklist (8 items)
Sirico 2018 (67)	Inception to March 2017	7	6–24 weeks	117/120	AE; RT; AE + RT; HIIT	Daily activities	Overweight/Obese Children	11.0 - 17.0	IL-6↓, CRP↔, Adiponectin↑, Leptin↓	Cochrane RoB tool
García-Hermoso 2016 (68)	Inception to September 2, 2015	9	6–24 weeks	219/208	AE	Daily activities	Overweight/Obese Adolescents	8.8 - 17.0	CRP↔	Delphi list

AE, aerobic exercise; CON, control group; CRP, C-reactive protein; EX, exercise; HF, heart failure; HIIT, high-intensity interval training; INT, intervention group; NR, not reported; RT, resistance training; SIT, sprint interval training; T2DM, type 2 diabetes mellitus; TESTEX, Tool for the assEssment of Study qualiTy and reporting in EXercise. Symbols: ↓, significant decrease (*p* < 0.05); ↑, significant increase (*p* < 0.05); ↔, no significant change (*p* ≥ 0.05).

#### 3.3.2 Effects of exercise on C-reactive protein

**Figure 6:**
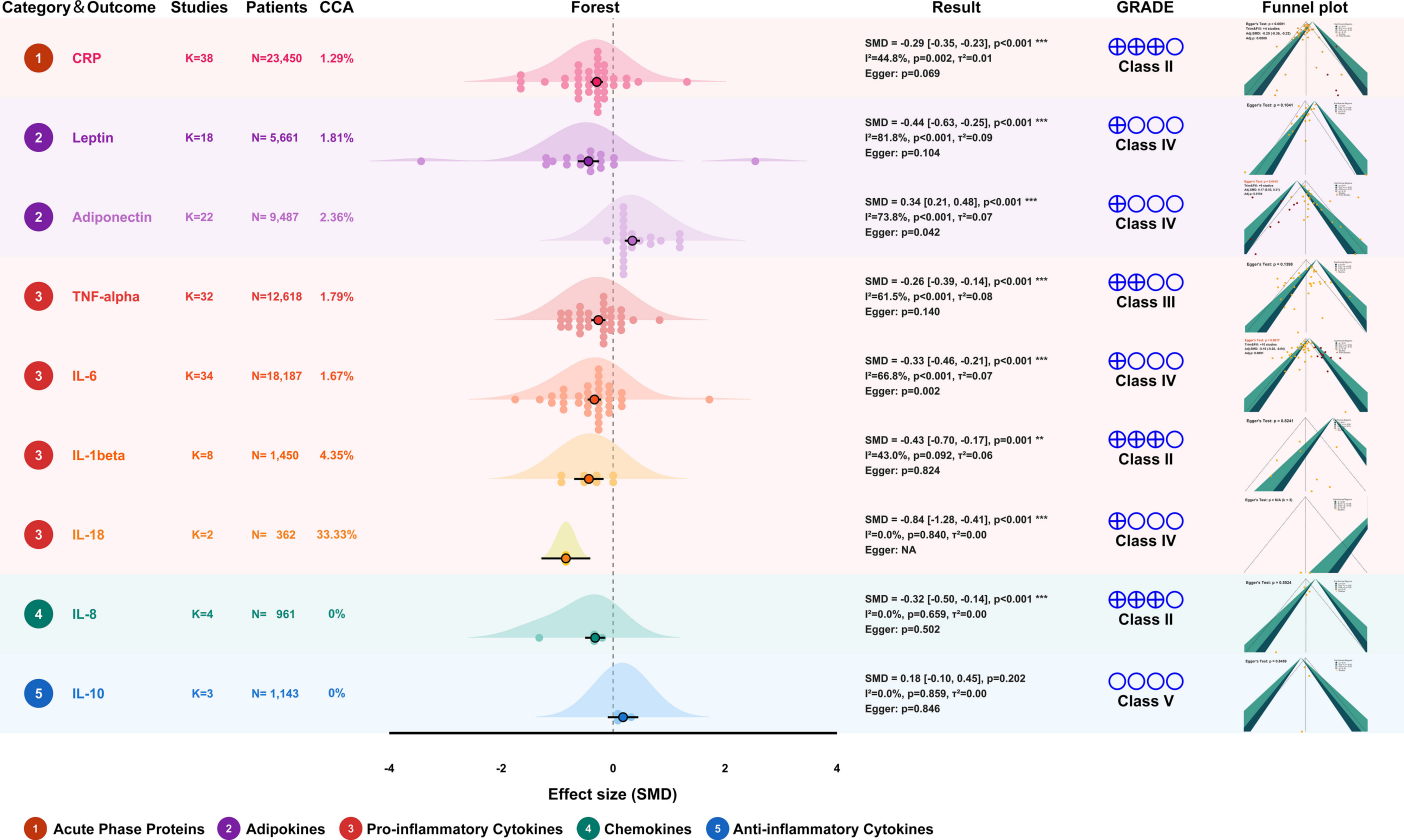
Forest plot of quantitative synthesis results. The forest plot displays the classification of outcomes, the number of included studies (K), total number of participants (N), pooled effect sizes (SMD) with 95% confidence intervals (*CI*), heterogeneity statistics (*I*² and *τ*²), publication bias (Egger’s test), GRADE certainty of evidence, and the degree of overlap measured by corrected covered area (CCA).

CRP, the quintessential acute-phase reactant indicative of low-grade systemic inflammation, was the most extensively studied biomarker. Data were synthesized from 23 meta-analyses, which provided 38 distinct exercise-control comparisons encompassing a total of 23,450 overweight/obese individuals. The pooled analysis showed a reduction in CRP concentrations following exercise interventions (SMD = -0.29, 95% CI: -0.35 to -0.23, *P* < 0.001; **Figure 6**). While the evidence exhibited moderate heterogeneity (*I*² = 44.8%, *P* = 0.002), the consistency of the effect direction across the diverse comparisons supported the robustness of the findings. Based on the GRADE assessment and hierarchical criteria, the evidence for exercise-induced CRP reduction was designated as Class II (Highly Suggestive).

#### 3.3.3 Impact of exercise on adipokines

Leptin acts as a pivotal regulator of energy balance and inflammatory responses. The synthesis of 10 meta-analyses, comprising 18 distinct comparisons (n = 5,661), yielded a pooled estimate favoring lower circulating leptin levels following exercise interventions (SMD = -0.44, 95% CI: -0.63 to -0.25, *P* < 0.001; **Figure 6**). However, heterogeneity was considerable (*I*² = 81.8%, *P* < 0.001), and the certainty of evidence was therefore rated as Class IV (Weak).

Adiponectin, an insulin-sensitizing and anti-inflammatory hormone primarily secreted by adipose tissue, was evaluated using data from 11 meta-analyses, which provided 22 distinct comparisons encompassing 9,487 participants. The pooled estimate favored higher adiponectin levels after exercise (SMD = 0.34, 95% CI: 0.21 to 0.48, *P* < 0.001; **Figure 6**). Nevertheless, substantial heterogeneity was present (*I*² = 73.8%, *P* < 0.001). Sensitivity analysis identified the meta-analysis by Rahimi et al. (2022) (58) as an important contributor to between-review variability; after excluding this review, heterogeneity decreased (*I*² = 34.4%) and the pooled estimate was attenuated (SMD = 0.24, 95% CI: 0.16 to 0.31; **Supplementary Figure 2**). Given the high heterogeneity in the primary analysis and evidence of publication bias (Egger’s *P* = 0.042), this outcome was classified as Class IV (Weak).

#### 3.3.4 Effects of exercise on pro-inflammatory cytokines

TNF-α, a pivotal mediator of insulin resistance and chronic low-grade inflammation, was examined using data synthesized from 19 systematic reviews, yielding 32 unique meta-analytic comparisons encompassing 9,398 participants. The pooled estimate favored lower TNF-α levels following exercise (SMD = -0.26, 95% CI: -0.39 to -0.14, *P* < 0.001; **Figure 6**). However, moderate-to-high heterogeneity was observed (*I*² = 61.5%, *P* < 0.001), and the certainty of evidence was therefore classified as Class III (Suggestive).

IL-6 functions as both a myokine and a pro-inflammatory mediator. Pooled data from 21 systematic reviews, providing 34 unique meta-analytic comparisons (n = 18,187), favored lower IL-6 levels after exercise (SMD = -0.33, 95% CI: -0.45 to -0.21, *P* < 0.001; **Figure 6**). This result should be interpreted cautiously because the primary analysis showed substantial heterogeneity (*I*² = 67.3%, *P* < 0.001) and evidence of publication bias (Egger’s *P* = 0.002). Accordingly, the certainty of evidence was rated as Class IV (Weak).

IL-1β plays a critical role in initiating the inflammatory cascade associated with obesity. The synthesis of 4 systematic reviews, comprising 8 unique meta-analytic comparisons (n = 1,450), showed that the primary pooled analysis favored lower circulating IL-1β concentrations after exercise (SMD = -0.43, 95% CI: -0.70 to -0.17, *P* = 0.001; **Figure 6**), with moderate heterogeneity (*I²* = 43.0%, *P* = 0.092). Sensitivity analysis suggested that the review by Malandish et al. (2023) (53) was a major contributor to heterogeneity; after excluding this review, heterogeneity decreased to zero and the pooled estimate was strengthened (SMD = -0.55, 95% CI: -0.75 to -0.35; **Supplementary Figure 5**). Because the zero-heterogeneity estimate was derived from sensitivity analysis rather than the primary analysis, and because the cumulative evidence base remained limited, this outcome was classified as Class II (Highly Suggestive).

#### 3.3.5 Effects of exercise on chemokines and regulatory cytokines

IL-8, a potent neutrophil chemoattractant, was assessed using data synthesized from 4 systematic reviews, which yielded 4 unique meta-analytic comparisons encompassing 961 participants. The pooled analysis favored lower IL-8 levels following exercise (SMD = -0.32, 95% CI: -0.50 to -0.14, *P* < 0.001; **Figure 6**), with no observed heterogeneity (*I*² = 0.0%, *P* = 0.659). However, given the limited number of contributing comparisons and the relatively small cumulative sample size, the certainty of evidence was considered Class II (Highly Suggestive).

IL-10 functions as a pivotal anti-inflammatory and immunoregulatory cytokine, preserving tissue integrity by curtailing excessive immune responses. Evidence synthesized from 3 systematic reviews (comprising 13 primary RCTs; n = 1,143) indicated that the effect of exercise on IL-10 levels did not reach statistical significance (SMD = 0.18, 95% CI: -0.10 to 0.45, *P* = 0.202; **Figure 6**). Although the analysis showed no observed heterogeneity (*I*² = 0.0%, *P* = 0.859), the wide confidence interval spanning the null line indicated imprecision. Given the lack of statistical significance, the current evidence was classified as Class V (Non-significant).

IL-18, a pleiotropic cytokine implicated in metabolic inflammation, was evaluated based on pooled results from 2 systematic reviews (n = 362). The pooled estimate favored lower IL-18 levels after exercise (SMD = -0.84, 95% CI: -1.28 to -0.41, *P* < 0.001; **Figure 6**), with no observed heterogeneity (*I*² = 0.0%, *P* = 0.840). However, this outcome should be interpreted with particular caution because the cumulative sample size did not meet the Optimal Information Size criterion and the corresponding reviews showed very high primary-study overlap (CCA = 33.33%), indicating a substantial risk of double-counting the same RCTs. Accordingly, the evidence was classified as Class IV (Weak).

#### 3.3.6 Subgroup analyses by age and health status

Prespecified subgroup analyses by age group and health status were conducted to explore potential sources of heterogeneity (**Table 2**). Across age strata, the pooled estimates for CRP consistently favored exercise, with larger reductions observed in mixed-age populations (SMD = -0.72) and adult women (SMD = -0.56), while the estimates remained smaller in children/adolescents (SMD = -0.25) and adults (SMD = -0.26). For adipokine-related outcomes, children and adolescents showed comparatively larger pooled estimates for leptin (SMD = -0.89) and adiponectin (SMD = 0.65), whereas the corresponding estimates in adults were more modest (leptin: SMD = -0.42; adiponectin: SMD = 0.33).

**Table 2:** Subgroup analysis of exercise effects on inflammatory markers in overweight/obese populations.

Outcome	CRP		Leptin		Adiponectin		TNF-α		IL-6	
Subgroups	K	SMD (95% C*I*) [Heterogeneity: *I*^2^; *p*-value]	K	SMD (95% C*I*) [Heterogeneity: *I*^2^; *p*-value]	K	SMD (95% C*I*) [Heterogeneity: *I*^2^; *p*-value]	K	SMD (95% C*I*) [Heterogeneity: *I*^2^; *p*-value]	K	SMD (95% C*I*) [Heterogeneity: *I*^2^; *p*-value]
Overall Effect	38	-0.29 (-0.35, -0.23) [*I*²=44.8%; *p* = 0.002]	18	-0.44 (-0.63, -0.25) [*I*²=81.8%; *p* < 0.001]	22	0.34 (0.21, 0.48) [*I*²=73.8%; *p* < 0.001]	32	-0.26 (-0.39, -0.14) [*I*²=61.5%; *p* < 0.001]	34	-0.33 (-0.45, -0.21) [*I*²=67.3%; *p* < 0.001]
Age group										
Children/Adolescents	12	-0.25 (-0.42, -0.09) [*I*²=52.9%; *p* = 0.016]	4	-0.89 (-1.52, -0.25) [*I*²=54.9%; *p* = 0.084]	3	0.65 (0.29, 1.00) [*I*²=2.5%; *p* = 0.358]	7	-0.04 (-0.46, 0.39) [*I*²=68.6%; *p* = 0.004]	8	-0.62 (-0.92, -0.33) [*I*²=56.8%; *p* = 0.023]
Adults	18	-0.26 (-0.30, -0.21) [*I*²=21.1%; *p* = 0.203]	11	-0.42 (-0.63, -0.22) [*I*²=85.9%; *p* < 0.001]	15	0.33 (0.18, 0.49) [*I*²=78.0%; *p* < 0.001]	17	-0.33 (-0.48, -0.17) [*I*²=64.1%; *p* < 0.001]	17	-0.21 (-0.34, -0.08) [*I*²=69.5%; *p* < 0.001]
Adult Women	6	-0.56 (-0.75, -0.37) [*I*²=17.7%; *p* = 0.299]	3	0.16 (-1.55, 1.87) [*I*²=75.3%; *p* = 0.017]	4	0.22 (-0.15, 0.59) [*I*²=61.2%; *p* = 0.052]	6	-0.35 (-0.63, -0.06) [*I*²=53.4%; *p* = 0.057]	7	-0.37 (-0.60, -0.13) [*I*²=19.5%; *p* = 0.281]
Mixed	2	-0.72 (-1.09, -0.35) [*I*²=0%; *p* = 0.477]	0	NA	0	NA	2	0.05 (-0.29, 0.39) [*I*²=2.90%; *p* = 0.310]	2	-0.28 (-0.53, -0.03) [*I*²=0%; *p* = 0.375]
Health status										
OW/OB	25	-0.29 (-0.37, -0.21) [*I*²=50.7%; *p* = 0.002]	10	-0.51 (-0.78, -0.24) [*I*²=88.5%; *p* < 0.001]	13	0.28 (0.17, 0.38) [*I*²=35.3%; *p* = 0.100]	21	-0.23 (-0.39, -0.06) [*I*²=67.4%; *p* < 0.001]	21	-0.32 (-0.47, -0.17) [*I*²=71.1%; *p* < 0.001]
T2D + OW/OB	5	-0.33 (-0.61, -0.06) [*I*²=61.9%; *p* = 0.033]	3	-0.30 (-0.60, -0.01) [*I*²=0%; *p* = 0.969]	4	0.24 (0.12, 0.37) [*I*²=0%; *p* = 0.966]	5	-0.45 (-0.76, -0.14) [*I*²=40.3%; *p* = 0.153]	5	-0.66 (-1.30, -0.01) [*I*²=75.0%; *p* = 0.003]
Cancer + OW/OB	4	-0.38 (-0.81, 0.05) [*I*²=0%; *p* = 0.580]	3	-0.44 (-1.03, 0.14) [*I*²=69.6%; *p* = 0.037]	3	0.06 (-0.20, 0.33) [*I*²=59.8%; *p* = 0.083]	4	-0.21 (-0.57, 0.14) [*I*²=33.3%; *p* = 0.212]	4	-0.12 (-0.31, 0.07) [*I*²=0%; *p* = 0.453]
Metabolic syndrome + OW/OB	2	-0.33 (-0.45, -0.21) [*I*²=0%; *p* = 0.774]	1	-1.26 (-2.40, -0.13) [*I*²=0%; *p* = 1.000]	2	1.16 (0.91, 1.41) [*I*²=0%; *p* = 0.387]	1	-0.57 (-0.95, -0.19) [*I*²=0%; *p* = 1.000]	2	-0.19 (-0.45, 0.07) [*I*²=0%; *p* = 0.440]
Heart failure + OW/OB	1	-0.38 (-0.56, -0.20) [*I*²=0%; *p* = 1.000]	0	NA	0	NA	1	-0.06 (-0.31, 0.19) [*I*²=0%; *p* = 1.000]	1	-0.20 (-0.33, -0.08) [*I*²=0%; *p* = 1.000]
Sarcopenia + OW/OB	1	1.32 (-0.65, 3.29) [*I*²=0%; *p* = 1.000]	1	2.54 (0.07, 5.01) [*I*²=0%; *p* = 1.000]	0	NA	0	NA	1	-0.87 (-1.62, -0.12) [*I*²=0%; *p* = 1.000]
Intervention										
AE	10	-0.24 (-0.30, -0.17) [*I*²=0%; *p* = 0.535]	4	-0.50 (-0.97, -0.03) [*I*²=95.3%; *p* < 0.001]	5	0.18 (0.11, 0.26) [*I*²=12.0%; *p* = 0.337]	7	-0.24 (-0.49, 0.01) [*I*²=66.8%; *p* = 0.006]	8	-0.31 (-0.48, -0.15) [*I*²=60.0%; *p* = 0.014]
RT	8	-0.45 (-0.71, -0.20) [*I*²=69.7%; *p* = 0.002]	4	-0.21 (-0.42, 0.00) [*I*²=31.1%; *p* = 0.226]	5	0.17 (0.01, 0.34) [*I*²=31.7%; *p* = 0.210]	8	-0.04 (-0.21, 0.14) [*I*²=39.9%; *p* = 0.113]	8	-0.24 (-0.63, 0.16) [*I*²=68.7%; *p* = 0.002]
AE + RT	12	-0.31 (-0.45, -0.18) [*I*²=26.9%; *p* = 0.180]	7	-0.62 (-0.96, -0.27) [*I*²=61.1%; *p* = 0.017]	7	0.37 (0.13, 0.60) [*I*²=54.2%; *p* = 0.041]	13	-0.49 (-0.72, -0.25) [*I*²=59.9%; *p* = 0.003]	12	-0.55 (-0.79, -0.31) [*I*²=62.3%; *p* = 0.002]
EX + Diet	6	-0.26 (-0.35, -0.17) [*I*²=60.3%; *p* = 0.028]	1	-0.46 (-1.14, 0.22) [*I*²=0%; *p* = 1.000]	2	0.86 (0.16, 1.57) [*I*²=85.6%; *p* = 0.008]	3	-0.12 (-0.25, 0.02) [*I*²=0%; *p* = 0.593]	5	-0.23 (-0.54, 0.09) [*I*²=81.3%; *p* < 0.001]
HIIT	2	-0.05 (-0.62, 0.52) [*I*²=66.1%; *p* = 0.086]	2	-0.65 (-1.62, 0.32) [*I*²=56.7%; *p* = 0.129]	3	0.30 (0.03, 0.58) [*I*²=17.5%; *p* = 0.297]	1	-0.57 (-0.95, -0.19) [*I*²=0%; *p* = 1.000]	1	-0.07 (-0.48, 0.33) [*I*²=0%; *p* = 1.000]
Sample size										
Large Sample (n≥500)	13	-0.27 (-0.32, -0.22) [*I*²=49.0%; *p* = 0.024]	3	-0.43 (-0.78, -0.07) [*I*²=66.7%; *p* = 0.050]	4	0.17 (0.10, 0.25) [*I*²=0%; *p* = 0.522]	9	-0.23 (-0.40, -0.06) [*I*²=57.0%; *p* = 0.017]	12	-0.25 (-0.39, -0.10) [*I*²=69.8%; *p* < 0.001]
Middle Sample (200≤n<500)	10	-0.34 (-0.54, -0.15) [*I*²=50.8%; *p* = 0.032]	9	-0.45 (-0.74, -0.16) [*I*²=65.7%; *p* = 0.003]	8	0.60 (0.27, 0.93) [*I*²=83.4%; *p* < 0.001]	5	-0.42 (-0.74, -0.09) [*I*²=63.6%; *p* = 0.027]	10	-0.34 (-0.54, -0.14) [*I*²=50.1%; *p* = 0.035]
Small Sample (n<200)	13	-0.29 (-0.44, -0.15) [*I*²=39.2%; *p* = 0.072]	6	-0.44 (-0.82, -0.06) [*I*²=92.0%; *p* < 0.001]	10	0.24 (0.08, 0.40) [*I*²=41.8%; *p* = 0.079]	15	-0.27 (-0.49, -0.05) [*I*²=66.2%; *p* < 0.001]	8	-0.28 (-0.56, 0.01) [*I*²=57.8%; *p* = 0.020]
Micro Sample (n<50)	2	-0.23 (-1.91, 1.45) [*I*²=50.9%; *p* = 0.154]	0	NA	0	NA	3	-0.00 (-0.80, 0.80) [*I*²=76.4%; *p* = 0.014]	4	-0.85 (-1.56, -0.13) [*I*²=74.9%; *p* = 0.008]
Duration category										
≤12w	6	-0.64 (-0.97, -0.31) [*I*²=49.0%; *p* = 0.081]	3	-1.32 (-1.93, -0.71) [*I*²=38.6%; *p* = 0.196]	2	1.00 (0.42, 1.58) [*I*²=0%; *p* = 0.525]	6	-0.32 (-0.64, 0.00) [*I*²=60.3%; *p* = 0.027]	9	-0.61 (-0.90, -0.31) [*I*²=65.9%; *p* = 0.003]
>12w	13	-0.29 (-0.38, -0.19) [*I*²=53.7%; *p* = 0.011]	6	-0.50 (-0.67, -0.32) [*I*²=41.0%; *p* = 0.132]	8	0.38 (0.11, 0.64) [*I*²=84.7%; *p* < 0.001]	6	-0.44 (-0.71, -0.17) [*I*²=67.6%; *p* = 0.009]	8	-0.24 (-0.37, -0.10) [*I*²=50.2%; *p* = 0.050]
6 - 24w	19	-0.25 (-0.33, -0.18) [*I*²=26.3%; *p* = 0.141]	9	-0.29 (-0.54, -0.05) [*I*²=87.0%; *p* < 0.001]	12	0.24 (0.13, 0.36) [*I*²=44.9%; *p* = 0.046]	20	-0.19 (-0.35, -0.03) [*I*²=59.9%; *p* < 0.001]	17	-0.25 (-0.42, -0.08) [*I*²=66.1%; *p* < 0.001]
Study quality										
High	4	-0.23 (-0.32, -0.15) [*I*²=0%; *p* = 0.777]	4	-0.52 (-0.74, -0.30) [*I*²=18.2%; *p* = 0.295]	4	0.20 (0.05, 0.35) [*I*²=0%; *p* = 0.910]	4	-0.42 (-0.67, -0.17) [*I*²=39.1%; *p* = 0.194]	4	-0.31 (-0.50, -0.12) [*I*²=0%; *p* = 0.656]
Moderate	8	-0.51 (-0.68, -0.34) [*I*²=39.5%; *p* = 0.115]	3	-0.37 (-1.05, 0.31) [*I*²=50.3%; *p* = 0.134]	5	0.19 (-0.11, 0.48) [*I*²=48.4%; *p* = 0.101]	8	-0.47 (-0.76, -0.18) [*I*²=68.2%; *p* = 0.003]	9	-0.41 (-0.63, -0.19) [*I*²=33%; *p* = 0.154]
Low	26	-0.26 (-0.32, -0.19) [*I*²=38.5%; *p* = 0.023]	12	-0.43 (-0.69, -0.18) [*I*²=86.2%; *p* < 0.001]	14	0.43 (0.24, 0.61) [*I*²=81.2%; *p* < 0.001]	20	-0.17 (-0.30, -0.03) [*I*²=53.9%; *p* = 0.002]	21	-0.34 (-0.52, -0.16) [*I*²=73%; *p* < 0.001]

AE, aerobic exercise; CRP, C-reactive protein; HIIT, high-intensity interval training; K, number of meta-analyses; OW/OB, overweight/obesity; RT, resistance training; SMD, standardized mean difference; T2D, type 2 diabetes; TNF-α, tumor necrosis factor-alpha.; NA, not applicable. Data: SMD (95% CI) with *I*² and p-value for heterogeneity. Subgroups: Defined by participant age, health status, intervention type, total sample size (Large: ≥500; Middle: 200-499; Small: <200), and intervention duration. Study quality: Rated as High, Moderate, or Low based on a comprehensive assessment using AMSTAR-2 and ROBIS tools.

By health status, participants with overweight/obesity without major comorbidities generally showed pooled estimates favoring exercise across several inflammatory markers, whereas the magnitude and consistency of effects varied in comorbid populations. In subgroups with T2D, pooled estimates favored lower CRP, TNF-α, and IL-6, together with higher adiponectin, although heterogeneity remained substantial for some outcomes, particularly IL-6 (SMD = -0.66, *I*² = 75.0%). In metabolic syndrome, the largest adipokine-related estimates were observed for leptin (SMD = -1.26) and adiponectin (SMD = 1.16), whereas findings in cancer-related populations were less consistent. These subgroup patterns may help explain part of the between-review heterogeneity, but they should be interpreted as review-level exploratory findings rather than definitive evidence of subgroup-specific responsiveness.

#### 3.3.7 Subgroup analyses by intervention characteristics

Subgroup analyses by intervention modality and duration indicated that pooled effects varied across exercise characteristics, although no single protocol can be considered uniformly superior across all biomarkers. Among modality-based subgroups, AE+RT was associated with comparatively larger pooled estimates for adiponectin (SMD = 0.37), leptin (SMD = -0.62), TNF-α (SMD = -0.49), and IL-6 (SMD = -0.55). For CRP, the largest pooled reduction was observed in RT-alone subgroups (SMD = -0.45), compared with AE alone (SMD = -0.24) and AE+RT (SMD = -0.31).

Duration-stratified analyses also suggested meaningful variation across intervention length. Interventions of ≤12 weeks were associated with larger pooled reductions in CRP (SMD = -0.64) and IL-6 (SMD = -0.61), whereas adiponectin showed a more favorable pooled estimate in interventions lasting >12 weeks (SMD = 0.38). Overall, these results suggest that different biomarkers may respond differently to exercise modality and duration, although formal between-subgroup comparisons should be interpreted cautiously because these analyses were not based on individual participant data.

#### 3.3.8 Subgroup analyses by methodological quality and sample size

Subgroup analyses stratified by methodological quality and sample size were undertaken to examine whether review-level characteristics were associated with heterogeneity and effect-size variation. Across several outcomes, high-quality studies tended to show lower heterogeneity (*I*²: 0%–43.4%) than low-quality studies, in which inconsistency was often substantial (frequently *I*² > 80%). At the same time, effect-size differences across quality strata were not unidirectional: for adiponectin, larger pooled estimates were observed in low-quality studies (SMD = 0.43) than in high-quality studies (SMD = 0.20), whereas for leptin and CRP, pooled reductions were similar or somewhat greater in high-quality strata.

Sample-size stratification further suggested possible small-study effects for some biomarkers. For IL-6, very small-sample studies (n < 50) showed a larger pooled estimate (SMD = -0.85) than large studies (n ≥ 500; SMD = -0.25). A similar pattern was observed for adiponectin, with medium-sized studies showing a larger pooled estimate (SMD = 0.60) than large studies (SMD = 0.17). Taken together, these findings indicate that study quality and sample size may contribute to between-review variability and to the magnitude of pooled estimates, and should therefore be considered when interpreting subgroup patterns.

#### 3.3.9 Primary-study overlap and publication bias

The CCA analysis indicated that most outcomes showed only slight overlap (CCA < 5%; **Figure 6**). An important exception was IL-18, for which overlap was very high (CCA = 33.33%). Although the contributing reviews reported directionally consistent findings, the substantial overlap indicates a risk of double-counting the same primary RCTs and warrants cautious interpretation of the pooled estimate (**Supplementary Table 17**). Regarding publication bias, Egger’s test and funnel plot inspections revealed no evidence of small-study effects for most markers (**Figure 6**). Potential bias was detected for adiponectin (*P* = 0.042) and IL-6 (*P* = 0.002). Subsequent trim-and-fill analyses attenuated the pooled estimates but retained statistical significance for adiponectin (adjusted SMD = 0.17, 95% CI 0.03 to 0.31, *P* = 0.018) and IL-6 (adjusted SMD = -0.16, 95% CI -0.28 to -0.04, *P* = 0.009), suggesting directional consistency despite reduced certainty due to heterogeneity and publication bias (**Supplementary Figures S10–S18**).

## 4 Discussion

This study combined a large-scale bibliometric analysis with an umbrella review to characterize research trends and re-evaluate the anti-inflammatory effects of exercise in overweight/obese populations. Across 25 meta-analyses including 342 RCTs, the certainty of evidence differed across biomarkers. CRP, IL-1β, and IL-8 showed pooled estimates favoring reduction with Class II (Highly Suggestive) evidence, whereas TNF-α was classified as Class III (Suggestive). IL-6, leptin, adiponectin, and IL-18 also showed pooled effects favoring improvement, but the certainty remained weak because of substantial heterogeneity, publication bias, limited information size, or high overlap. No significant systemic effect was observed for IL-10 (Class V).

The bibliometric findings indicated a shift in research emphasis over time. Early influential studies and co-citation clusters were centered on obesity, inflammation, and insulin resistance, including adipose tissue macrophage infiltration and endoplasmic reticulum stress mechanisms (86–88). Subsequent clusters increasingly focused on adipokines and cardiometabolic regulation (89), whereas more recent keyword and co-citation patterns highlighted cellular remodeling, immune crosstalk, gut microbiota, and other mechanism-oriented themes (90, 91). However, the umbrella review shows that this expansion in mechanistic interest has not been matched by uniformly strong evidence across inflammatory biomarkers. More consistent evidence was observed for CRP and, to a lesser extent, IL-1β and IL-8, whereas evidence for adipokines and several cytokines remained constrained by heterogeneity, publication bias, or overlap. Taken together, these findings suggest that the field has expanded rapidly in thematic breadth, but evidence maturity remains uneven across outcomes.

Among the included biomarkers, CRP provided the broadest and most stable evidence base. As a hepatic acute-phase reactant and a widely used indicator of systemic low-grade inflammation, CRP was synthesized from the largest number of comparisons and showed a pooled reduction with only moderate heterogeneity. This pattern is consistent with earlier meta-analyses of exercise and CRP (92) and remains supported after the incorporation of more recent evidence (93, 94). In the subgroup analyses, larger pooled CRP reductions were observed in RT-alone and shorter-duration subgroups. Nevertheless, because these subgroup comparisons were conducted at the review level rather than the individual-participant level, they should be interpreted as exploratory rather than as evidence of a definitively superior protocol.

Pooled estimates also favored lower IL-1β and IL-8 after exercise, and both outcomes were classified as Class II. For IL-1β, the primary analysis showed moderate heterogeneity, and the zero-heterogeneity estimate emerged only after the exclusion of the review by Malandish et al. (53) in the sensitivity analysis. Accordingly, the current findings support a likely beneficial effect but not definitive high-certainty evidence. Mechanistically, this is biologically plausible because IL-1β is closely linked to inflammasome activation and obesity-related sterile inflammation (95). Exercise-induced regulation of METRNL and β-hydroxybutyrate has also been proposed as a pathway through which inflammasome signaling may be attenuated (96, 97). For IL-8, the observed pooled reduction is also biologically compatible with exercise-related improvements in endothelial inflammatory signaling and leukocyte recruitment (98). However, the limited number of contributing reviews and participants indicates that both IL-1β and IL-8 still require confirmation in larger, methodologically consistent RCTs.

For TNF-α and IL-6, the pooled estimates in our umbrella review generally favored lower concentrations after exercise, but the certainty of evidence remained constrained by substantial between-review heterogeneity and, for IL-6, additional evidence of publication bias. Rather than indicating absence of effect, this pattern more likely reflects the context-dependent biology of these cytokines. In a network meta-analysis of randomized controlled trials in individuals with overweight and obesity, Chen et al. (2024) (99) reported that different exercise modalities showed different rankings across inflammatory biomarkers, with modality-specific effects for TNF-α and IL-6, suggesting that intervention composition is a plausible source of between-study variability. A recent endocrine review by James et al. (2025) (100) further highlighted that exercise-related changes in adipose tissue inflammation, myokine signaling, and systemic metabolic status may all influence circulating TNF-α and IL-6. Our findings extend this perspective by showing that these responses are unlikely to be uniform across populations and protocols. This is particularly relevant for IL-6, which functions both as a pro-inflammatory adipokine in obesity and as a contraction-induced myokine during exercise; consequently, basal reductions in circulating IL-6 may coexist with transient exercise-induced elevations depending on metabolic context and sampling conditions.

A similar interpretive framework applies to leptin and adiponectin. In our umbrella review, exercise was associated with lower leptin and higher adiponectin, which is directionally consistent with earlier syntheses in overweight/obese and metabolically at-risk populations, including the systematic review by Yu et al. (2017) (101) and the meta-analysis by Becic et al. (2018) (102), both of which reported improvement in adipokine profiles after exercise. More recently, Wang et al. (2025) (103) used pairwise, network, and dose–response meta-analyses to show that adipokine responses are modality- and dose-dependent, with HIIT ranking highest for adiponectin and combined training showing the greatest reduction in leptin. These observations are compatible with our subgroup findings, but they also caution against interpreting any single modality as uniformly superior across populations. Mechanistically, adiponectin may respond to exercise through improvements in insulin sensitivity, mitochondrial function, and attenuation of adipose tissue stress, whereas leptin is more tightly coupled to fat mass and whole-body energy balance (104, 105); accordingly, part of the apparent leptin response may still reflect concomitant changes in adiposity rather than a purely exercise-specific effect.

IL-18 requires additional caution. Although the pooled estimate favored lower IL-18 levels after exercise, the cumulative sample size was limited, and the overlap between the contributing reviews was very high (CCA = 33.33%). This degree of overlap raises the possibility of double-counting the same primary RCTs and therefore limits confidence in the summary estimate. Accordingly, the current evidence for IL-18 should be interpreted as weak. In contrast, no significant systemic effect was observed for IL-10. Although tissue-level or transient anti-inflammatory effects remain biologically plausible, the current umbrella review did not directly test such mechanisms. Therefore, interpretations involving localized or paracrine IL-10 regulation should be considered hypothesis-generating rather than confirmatory (106–108).

## 5 Strengths and limitations

A major strength of this study is the integration of bibliometric analysis with an umbrella review, allowing research trends, knowledge structure, and evidence certainty to be interpreted within a single framework. In addition, the study explicitly evaluated methodological quality, primary-study overlap, publication bias, and evidence certainty, which strengthened the transparency of the synthesis.

Several limitations should also be acknowledged. First, although CRP, IL-1β, and IL-8 showed the most consistent pooled signals, the evidence for IL-1β and IL-8 remained limited by the small number of contributing comparisons and cumulative sample size, and was therefore rated as highly suggestive rather than convincing. Second, the evidence for IL-18 was additionally constrained by very high overlap between reviews, which increased the risk of double-counting primary trials. Third, many included reviews were rated as low quality by AMSTAR-2 and ROBIS, largely because of reporting deficiencies; although this does not necessarily invalidate the underlying RCT evidence, it reduces confidence in review-level synthesis. Fourth, subgroup findings were exploratory and based on review-level rather than individual-level data. Finally, the restriction to English-language publications may have introduced language bias.

## 6 Conclusions

This integrated bibliometric analysis and umbrella review indicates that research on exercise-induced immunomodulation is becoming increasingly mechanism-oriented, while evidence maturity remains uneven across inflammatory biomarkers. Quantitative synthesis showed that exercise was associated with reductions in CRP (SMD = -0.29, 95% CI: -0.35 to -0.23), IL-1β (SMD = -0.43, 95% CI: -0.70 to -0.17), and IL-8 (SMD = -0.32, 95% CI: -0.50 to -0.14), all supported by Class II (Highly Suggestive) evidence, with the IL-1β estimate showing improved consistency after sensitivity analysis. Evidence for TNF-α was Class III (Suggestive), whereas evidence for IL-6, leptin, adiponectin, and IL-18 remained Class IV (Weak) because of substantial heterogeneity, publication bias, limited information size, or high primary-study overlap. No significant systemic effect was identified for IL-10 (Class V). Overall, these findings provide quantitative support for the beneficial effects of exercise on selected systemic inflammatory biomarkers, particularly CRP, IL-1β, and IL-8. Future adequately powered and standardized randomized trials are needed to resolve current evidence limitations and advance precision exercise prescription.

## Data Availability

The original contributions presented in the study are included in the article/**Supplementary Material**. Further inquiries can be directed to the corresponding authors.
