# Supplementary material for: Exercise-induced changes in systemic inflammatory biomarkers in overweight and obese populations: a bibliometric analysis and umbrella review of meta-analyses
Source: Front Immunol. 2026 May 20;17:1838118. doi: 10.3389/fimmu.2026.1838118 (PMC13230183; doi:10.3389/fimmu.2026.1838118)
Supplement: Supplementary file 2 [file SupplementaryFile2.pdf]

# *Supplementary File 2*

## Contents


**1 Search Strategy for the Bibliometric Analysis ........ 1**
1.1 Supplementary Table S1. Search Strategy for the Bibliometric Analysis (Web of Science Database) ........ 1
**2 Search Strategy for the Umbrella Review ........ 2**
2.1 Supplementary Table S2. Search Strategy for the PubMed Database ........ 2
2.2 Supplementary Table S3. Search Strategy for the WOS Database ........ 3
2.3 Supplementary Table S4. Search Strategy for the Cochrane Database ........ 4
2.4 Supplementary Table S5. Search Strategy for the EMbase Database ........ 5
2.5 Supplementary Table S6. Search Strategy for the Scopus Database ........ 5
**3 List of Studies Excluded After Full-Text Review ........ 6**
Supplementary Table S7. List of studies excluded after full-text review with reasons. ........ 6
**4 Supplementary Results: Sensitivity Analyses ........ 8**
Supplementary Figure S1. Results of Sensitivity Analysis for CRP ........ 8
Supplementary Figure S2. Results of Sensitivity Analysis for Adiponectin ........ 8
Supplementary Figure S3. Results of Sensitivity Analysis for Leptin ........ 9
Supplementary Figure S4. Results of Sensitivity Analysis for TNF-α ........ 9
Supplementary Figure S5. Results of Sensitivity Analysis for IL-1β ........ 9
Supplementary Figure S6. Results of Sensitivity Analysis for IL-6 ........ 10
Supplementary Figure S7. Results of Sensitivity Analysis for IL-8 ........ 10
Supplementary Figure S8. Results of Sensitivity Analysis for IL-10 ........ 10
Supplementary Figure S9. Results of Sensitivity Analysis for IL-18 ........ 10
**5 Supplementary Results: Publication Bias Analyses ........ 11**
Supplementary Figure S10. Results of Publication Bias Analysis for CRP ........ 11
Supplementary Figure S11. Results of Publication Bias Analysis for Leptin ........ 11
Supplementary Figure S12. Results of Publication Bias Analysis for Adiponectin ........ 12
Supplementary Figure S13. Results of Publication Bias Analysis for IL-6 ........ 12
Supplementary Figure S14. Results of Publication Bias Analysis for TNF-α ........ 13
Supplementary Figure S15. Results of Publication Bias Analysis for IL-1β ........ 13
Supplementary Figure S16. Results of Publication Bias Analysis for IL-8 ........ 14
Supplementary Figure S17. Results of Publication Bias Analysis for IL-10 ........ 14
Suplementary Figure S18. Results of Publication Bias Analysis for IL-18 ........ 14
**6 Supplementary Results: Subgroup Analyses ........ 15**
6.1 For CRP ........ 15
6.2 For Adiponectin ........ 21
6.3 For Leptin ........ 27
6.4 For TNF-α ........ 33
6.5 For IL-6 ........ 39
**7 Supplementary Results: Corrected Covered Area (CCA) Overlap Analysis by Outcome ........ 45**
7.1 For CRP ........ 45
7.2 For Adiponectin ........ 54
7.3 For Leptin ........ 58
7.4 For TNF-α ........ 61
7.5 For IL-1β ........ 65
7.6 For IL-6 ........ 66
7.7 For IL-8 ........ 72
7.8 For IL-10 ........ 73
7.9 For IL-18 ........ 74

# 1 Search Strategy for the Bibliometric Analysis

## 1.1 Supplementary Table S1. Search Strategy for the Bibliometric Analysis (Web of Science Database)

| Step | Search strategy | Number of articles |
|---|---|---|
| #1 | TS=(Overweight) | 28,436 |
| #2 | TS=(Obesity) | 116,183 |
| #3 | TS=(Exercise OR Exercises OR "Physical Exercise" OR "Physical Exercises" OR "Exercise, Physical" OR "Exercises, Physical" OR<br>Aerobic OR "Aerobic Exercise" OR "Aerobic Exercises" OR "Exercise, Aerobic" OR "Exercises, Aerobic" OR<br>Isometric OR "Isometric Exercise" OR "Isometric Exercises" OR "Exercise, Isometric" OR "Exercises, Isometric" OR<br>Acute OR "Acute Exercise" OR "Acute Exercises" OR "Exercise, Acute" OR "Exercises, Acute" OR<br>Training OR "Exercise Training" OR "Exercise Trainings" OR "Training, Exercise" OR "Trainings, Exercise" OR<br>Activity OR "Physical Activity" OR "Physical Activities" OR "Activity, Physical" OR "Activities, Physical") | 7,628,911 |
| #4 | TS=(Inflammation OR Inflammations OR "Innate Inflammatory Response" OR "Inflammatory Response, Innate" OR "Innate Inflammatory Responses") | 855,553 |
| #5 | #1 OR #2 | 505,885 |
| #6 | #3 AND #4 AND #5 | 16,257 |
| #7 | **Document Types: Article** | 12,276 |
| #8 | **NOT Document Types: Retracted Publication** | 12,248 |
| #9 | **Languages: English** | 12,125 |
| #10 | **Eliminate duplicates or extract the 2026 published literature.** | **10,101** |

## 2 Search Strategy for the Umbrella Review

### 2.1 Supplementary Table S2. Search Strategy for the PubMed Database

| Step | Search strategy | Number of articles |
|---|---|---|
| #1 | Search: "Overweight"[Mesh] Sort by: Most Recent | 301,848 |
| #2 | Search: "Obesity"[Mesh] Sort by: Most Recent | 289,717 |
| #3 | Search: ("Exercise"[MeSH Terms] OR ("Exercises"[Title/Abstract] OR "Exercise, Physical"[Title/Abstract] OR "Exercises, Physical"[Title/Abstract] OR "Physical Exercise"[Title/Abstract] OR "Physical Exercises"[Title/Abstract] OR "Exercise, Aerobic"[Title/Abstract] OR "Aerobic Exercise"[Title/Abstract] OR "Aerobic Exercises"[Title/Abstract] OR "Exercises, Aerobic"[Title/Abstract] OR "Exercise, Isometric"[Title/Abstract] OR "Exercises, Isometric"[Title/Abstract] OR "Isometric Exercises"[Title/Abstract] OR "Isometric Exercise"[Title/Abstract] OR "Acute Exercise"[Title/Abstract] OR "Acute Exercises"[Title/Abstract] OR "Exercise, Acute"[Title/Abstract] OR "Exercises, Acute"[Title/Abstract] OR "Exercise Training"[Title/Abstract] OR "Exercise Trainings"[Title/Abstract] OR "Training, Exercise"[Title/Abstract] OR "Trainings, Exercise"[Title/Abstract] OR "Physical Activity"[Title/Abstract] OR "Activities, Physical"[Title/Abstract] OR "Activity, Physical"[Title/Abstract] OR "Physical Activities"[Title/Abstract])) Sort by: Most Recent | 457,956 |
| #4 | Search: ("Inflammation"[MeSH Terms] OR "Inflammatory Response"[MeSH Terms] OR ("Inflammations"[Title/Abstract] OR "Innate Inflammatory Response"[Title/Abstract] OR "Inflammatory Response, Innate"[Title/Abstract] OR "Innate Inflammatory Responses"[Title/Abstract] OR "inflammatory reaction"[Title/Abstract] OR "inflammatory process"[Title/Abstract])) Sort by: Most Recent | 501,971 |
| #5 | Search: "Interleukins"[Mesh] Sort by: Most Recent | 290,783 |
| #6 | Search: ("Tumor Necrosis Factor-alpha"[MeSH Terms] OR ("Tumor Necrosis Factor alpha"[Title/Abstract] OR "TNF-alpha"[Title/Abstract] OR "Tumor Necrosis Factor"[Title/Abstract] OR "TNF Superfamily, Member 2"[Title/Abstract] OR "Tumor Necrosis Factor Ligand Superfamily Member 2"[Title/Abstract] OR "TNFalpha"[Title/Abstract] OR "Cachectin-Tumor Necrosis Factor"[Title/Abstract] OR "Cachectin Tumor Necrosis Factor"[Title/Abstract] OR "Cachectin"[Title/Abstract])) Sort by: Most Recent | 324,151 |
| #7 | Search: ("C-Reactive Protein"[MeSH Terms] OR ("C Reactive Protein"[Title/Abstract] OR "hs-CRP"[Title/Abstract] OR "hsCRP"[Title/Abstract] OR "High Sensitivity C-Reactive Protein"[Title/Abstract] OR "High Sensitivity C Reactive Protein"[Title/Abstract])) Sort by: Most Recent | 122,312 |
| #8 | Search: "Meta-Analysis" [Publication Type] Sort by: Most Recent | 229,206 |
| #9 | Search: ("Systematic Reviews as Topic"[MeSH Terms] OR ("Reviews Systematic as Topic"[Title/Abstract] OR "Systematic Review as Topic"[Title/Abstract] OR "Umbrella Reviews as Topic"[Title/Abstract])) Sort by: Most Recent | 16,847 |
| #10 | #1 OR #2 | 301,865 |
| #11 | #4 OR #5 OR #6 OR #7 | 1,048,909 |
| #12 | #8 OR #9 | 245,558 |
| #13 | #3 AND #10 AND #11 AND #12 | 32 |

### 2.2 Supplementary Table S3. Search Strategy for the WOS Database

| Step | Search strategy | Number of articles |
|---|---|---|
| #1 | TS=(Overweight) | 28,436 |
| #2 | TS=(Obesity) | 116,183 |
| #3 | TS=(Exercise OR Exercises OR "Physical Exercise" OR "Physical Exercises" OR "Exercise, Physical" OR "Exercises, Physical" OR Aerobic OR "Aerobic Exercise" OR "Aerobic Exercises" OR "Exercise, Aerobic" OR "Exercises, Aerobic" OR Isometric OR "Isometric Exercise" OR "Isometric Exercises" OR "Exercise, Isometric" OR "Exercises, Isometric" OR Acute OR "Acute Exercise" OR "Acute Exercises" OR "Exercise, Acute" OR "Exercises, Acute" OR Training OR "Exercise Training" OR "Exercise Trainings" OR "Training, Exercise" OR "Trainings, Exercise" OR Activity OR "Physical Activity" OR "Physical Activities" OR "Activity, Physical" OR "Activities, Physical") | 1,855,358 |
| #4 | TS=(Inflammation OR Inflammations OR "Innate Inflammatory Response" OR "Inflammatory Response, Innate" OR "Innate Inflammatory Responses") | 252,819 |
| #5 | TS=(Interleukins) | 2.309 |
| #6 | TS=(C-Reactive Protein OR "C Reactive Protein" OR hs-CRP OR hsCRP OR "High Sensitivity C-Reactive Protein" OR "High Sensitivity C Reactive Protein") | 28.340 |
| #7 | TS=("Tumor Necrosis Factor-alpha" OR "Tumor Necrosis Factor alpha" OR "TNF-alpha" OR "Tumor Necrosis Factor" OR "TNF Superfamily, Member 2" OR "Tumor Necrosis Factor Ligand Superfamily Member 2" OR "TNFalpha" OR "Cachectin-Tumor Necrosis Factor" OR "Cachectin Tumor Necrosis Factor" OR "Cachectin") | 65.315 |
| #8 | TS=(Systematic Reviews) | 218.129 |
| #9 | TS=(Meta-Analysis) | 148.504 |
| #10 | #1 OR #2 | 123.214 |
| #11 | #4 OR #5 OR #6 OR #7 | 301.351 |
| #12 | #8 OR #9 | 262.547 |
| #13 | #3 AND #10 AND #11 AND #12 | 232 |

## 2.3 Supplementary Table S4. Search Strategy for the Cochrane Database

| Step | Search strategy | Number of articles |
|---|---|---|
| #1 | TS=(Overweight) | 254,11 |
| #2 | TS=(Obesity) | 59,182 |
| #3 | TS=(Exercise OR Exercises OR "Physical Exercise" OR "Physical Exercises" OR "Exercise, Physical" OR "Exercises, Physical" OR Aerobic OR "Aerobic Exercise" OR "Aerobic Exercises" OR "Exercise, Aerobic" OR "Exercises, Aerobic" OR Isometric OR "Isometric Exercise" OR "Isometric Exercises" OR "Exercise, Isometric" OR "Exercises, Isometric" OR Acute OR "Acute Exercise" OR "Acute Exercises" OR "Exercise, Acute" OR "Exercises, Acute" OR Training OR "Exercise Training" OR "Exercise Trainings" OR "Training, Exercise" OR "Trainings, Exercise" OR Activity OR "Physical Activity" OR "Physical Activities" OR "Activity, Physical" OR "Activities, Physical") | 585,714 |
| #4 | TS=(Inflammation OR Inflammations OR "Innate Inflammatory Response" OR "Inflammatory Response, Innate" OR "Innate Inflammatory Responses") | 64,563 |
| #5 | TS=(Interleukins) | 1,630 |
| #6 | TS=(C-Reactive Protein OR "C Reactive Protein" OR hs-CRP OR hsCRP OR "High Sensitivity C-Reactive Protein" OR "High Sensitivity C Reactive Protein") | 28.859 |
| #7 | TS=("Tumor Necrosis Factor-alpha" OR "Tumor Necrosis Factor alpha" OR "TNF-alpha" OR "Tumor Necrosis Factor" OR "TNF Superfamily, Member 2" OR "Tumor Necrosis Factor Ligand Superfamily Member 2" OR "TNFalpha" OR "Cachectin-Tumor Necrosis Factor" OR "Cachectin Tumor Necrosis Factor" OR "Cachectin") | 17,110 |
| #8 | TS=(Systematic Reviews) | 18,180 |
| #9 | TS=(Meta-Analysis) | 30,913 |
| #10 | #1 OR #2 | 65,800 |
| #11 | #4 OR #5 OR #6 OR #7 | 92,582 |
| #12 | #8 OR #9 | 37,729 |
| #13 | #3 AND #10 AND #11 AND #12 | 407 |
| | Cochrane Reviews | 316 |

## 2.4 Supplementary Table S5. Search Strategy for the EMbase Database

| Step | Search strategy | Number of articles |
|---|---|---|
| #1 | obesity | 163,674 |
| #2 | overweight | 894,648 |
| #3 | exercise OR exercises OR 'physical exercise' OR 'physical exercises' OR 'exercise, physical' OR 'exercises, physical' OR aerobic OR 'aerobic exercise' OR 'aerobic exercises' OR 'exercise, aerobic' OR 'exercises, aerobic' OR isometric OR 'isometric exercise' OR 'isometric exercises' OR 'exercise, isometric' OR 'exercises, isometric' OR acute OR 'acute exercise' OR 'acute exercises' OR 'exercise, acute' OR 'exercises, acute' OR training OR 'exercise training' OR 'exercise trainings' OR 'training, exercise' OR 'trainings, exercise' OR activity OR 'physical activity' OR 'physical activities' OR 'activity, physical' OR 'activities, physical' | 9,841,596 |
| #4 | inflammation OR inflammations OR 'innate inflammatory response' OR 'inflammatory response, innate' OR 'innate inflammatory responses' | 1,551,126 |
| #5 | interleukins | 15,606 |
| #6 | 'c reactive' AND protein OR 'c reactive protein' OR 'hs crp' OR hscrp OR 'high sensitivity c-reactive protein' OR 'high sensitivity c reactive protein' | 349,952 |
| #7 | 'tumor necrosis factor-alpha'/exp OR 'tumor necrosis factor-alpha' OR 'tumor necrosis factor alpha'/exp OR 'tumor necrosis factor alpha' OR 'tnf-alpha'/exp OR 'tnf-alpha' OR 'tumor necrosis factor'/exp OR 'tumor necrosis factor' OR 'tnf superfamily, member 2' OR 'tumor necrosis factor ligand superfamily member 2' OR 'tnfalpha' OR 'cachectin-tumor necrosis factor' OR 'cachectin tumor necrosis factor' OR 'cachectin'/exp OR 'cachectin' | 639,277 |
| #8 | systematic AND reviews | 196,660 |
| #9 | 'meta analysis' | 531,425 |
| #10 | #1 OR #2 | 908,599 |
| #11 | #4 OR #5 OR #6 OR #7 | 2,174,941 |
| #12 | #8 OR #9 | 643,842 |
| #13 | #3 AND #10 AND #11 AND #12 | 1,202 |

## 2.5 Supplementary Table S6. Search Strategy for the Scopus Database

| Step | Search strategy | Number of articles |
|---|---|---|
| #1 | "Overweight" OR "Obesity" | 2,108,626 |
| #2 | "Exercise" OR "Exercises" OR "Exercise, Physical" OR "Exercises, Physical" OR "Physical Exercise" OR "Physical Exercises" OR "Exercise, Aerobic" OR "Aerobic Exercise" OR "Aerobic Exercises" OR "Exercises, Aerobic" OR "Exercise, Isometric" OR "Exercises, Isometric" OR "Isometric Exercises" OR "Isometric Exercise" OR "Acute Exercise" OR "Acute Exercises" OR "Exercise, Acute" OR "Exercises, Acute" OR "Exercise Training" OR "Exercise Trainings" OR "Training, Exercise" OR "Trainings, Exercise" OR "Physical Activity" OR "Activities, Physical" OR "Activity, Physical" OR "Physical Activities" | 2,923,419 |
| #3 | "Inflammation" OR "Inflammatory Response" OR "Inflammations" OR "Innate Inflammatory Response" OR "Inflammatory Response, Innate" OR "Innate Inflammatory Responses" OR "inflammatory reaction" OR "inflammatory process" OR "Tumor Necrosis Factor alpha" OR "TNF-alpha" OR "Tumor Necrosis Factor" OR "TNF Superfamily, Member 2" OR "Tumor Necrosis Factor Ligand Superfamily Member 2" OR "TNFalpha" OR "Cachectin" OR "C Reactive Protein" OR "hs-CRP" OR "hsCRP" OR "High Sensitivity C-Reactive Protein" OR "High Sensitivity C Reactive Protein" OR "Interleukins" | 4,910,505 |
| #4 | "meta-analysis" OR "systematic review" | 9,088,438 |
| #5 | #1 AND #2 AND #3 AND #4 | 795 |

## 3 List of Studies Excluded After Full-Text Review

### 3.1 Supplementary Table S7. List of studies excluded after full-text review with reasons.

| No | First author, yea | Title | Journal | Study population | Intervention | Control intervention | Exclusion reason |
|---|---|---|---|---|---|---|---|
| 1 | Katzmarzyk, 2012 [1] | Physical activity for obese individuals: a systematic review of effects on chronic disease risk factors | Obesity Reviews | Obese individuals | Aerobic exercise | Routine treatment | ***Not a meta-analysis*** |
| 2 | Marques Elias, 2015 [2] | Effect of physical exercise on inflammatory markers of adolescents with overweight: A systematic review | Revista de Educação Física/UEM | Overweight adolescents | Exercise training | Usual care | ***Not a meta-analysis*** |
| 3 | Chen, 2020 [3] | Effects of Exercise on Inflammatory Cytokines in Patients with Type 2 Diabetes: A Meta-analysis of Randomized Controlled Trials | Oxidative Medicine and Cellular Longevity | T2DM patients | Exercise training | Routine treatment | ***Ineligible study population*** |
| 4 | Saeidi, 2021 [4] | The effects of physical activity on adipokines in individuals with overweight/obesity across the lifespan: A narrative review | Obesity Reviews | Overweight/ Obese | Exercise training | Usual care | ***Not a meta-analysis*** |
| 5 | Gonzalo-Encabo, 2021 [5] | The role of exercise training on low-grade systemic inflammation in adults with overweight and obesity: A systematic review | International Journal of Environmental Research and Public Health | Overweight/ Obese adults | Exercise training | Routine medication | ***Not a meta-analysis*** |
| 6 | Jansson, 2022 [6] | Effects of Resistance and Endurance Training Alone or Combined on Hormonal Adaptations and Cytokines in Healthy Children and Adolescents: A Systematic Review and Meta- analysis | Sports Medicine - Open | Healthy children/adolescents | Anaerobic exercise | Usual care | ***Ineligible study population*** |
| 7 | Zhao, 2022 [7] | Effects of exercise combined with diet intervention on body composition and serum biochemical markers in adolescents with obesity: a systematic review and meta-analysis | Journal of Pediatric Endocrinology and Metabolism | Obese adolescents | Exercise + Diet control | Usual care | ***Insufficient data*** |
| 8 | Liu, 2022 [8] | Meta-analysis of effectiveness of different exercises on inflammatory response in obese adolescents | Chinese Journal of School Health | Obese adolescents | Exercise training | Routine treatment | ***Non-English publication*** |
| 9 | Papagianni, 2023 [9] | The anti-inflammatory effects of aerobic exercise training in patients with type 2 diabetes: A systematic review and meta-analysis | Cytokine | Adults with T2DM | Aerobic exercise | Usual care | ***Ineligible study population*** |
| 10 | Chen, 2024 [10] | Efficacy of different exercise types on inflammatory cytokines in individuals with overweight or obesity: a network meta-analysis | Chinese Journal of Evidence-Based Medicine | Overweight/ Obese | Exercise training | Diet control | ***Non-English publication*** |
| 11 | Kadoglou, 2025 [11] | The Anti-Inflammatory Effects of Resistance Training in Patients with Type 2 Diabetes: A Systematic Review and Meta-Analysis | Biomolecules | Adults with T2DM | Resistance training | Routine treatment | ***Ineligible study population*** |

## References


1. Zalagkitis C, Philippou A, Karatzanos E, Metsios GS, Dinas PC. Combined effects of physical activity and diet on chronic inflammation of overweight/obese children and adolescents: A systematic review and meta-analysis. Journal of Sports Sciences. 2025;43(22):2841-57. https://doi.org/10.1080/02640414.2025.2561349
2. Marques Elias RGM, de Farias JP, de Faria WF, Stabelini Neto AS, Silva CC, Rinaldi W. Effect of physical exercise on inflammatory markers of adolescents with overweight: A systematic review. Rev Educ Fis. 2015;26(4):633-45. https://doi.org/10.4025/reveducfis.v26i4.26416

3. Chen X, Sun X, Wang C, He H. Effects of Exercise on Inflammatory Cytokines in Patients with Type 2 Diabetes: A Meta-analysis of Randomized Controlled Trials. Oxidative Medicine and Cellular Longevity. 2020;2020. https://doi.org/10.1155/2020/6660557
4. Saeidi A, Haghighi MM, Kolahdouzi S, Daraei A, Abderrahmane AB, Essop MF, et al. The effects of physical activity on adipokines in individuals with overweight/obesity across the lifespan: A narrative review. Obesity Reviews. 2021;22(1). https://doi.org/10.1111/obr.13090
5. Gonzalo-Encabo P, Maldonado G, Valades D, Ferragut C, Pérez-López A. The role of exercise training on low-grade systemic inflammation in adults with overweight and obesity: A systematic review. International Journal of Environmental Research and Public Health. 2021;18(24). https://doi.org/10.3390/ijerph182413258
6. Jansson D, Lindberg AS, Lundberg E, Domellöf M, Theos A. Effects of Resistance and Endurance Training Alone or Combined on Hormonal Adaptations and Cytokines in Healthy Children and Adolescents: A Systematic Review and Meta-analysis. SPORTS MEDICINE-OPEN. 2022 DEC;8(1). https://doi.org/10.1186/s40798-022-00471-6
7. Zhao LY, Dong XS, Gao Y, Jia ZH, Han SY, Zhang JT, et al. Effects of exercise combined with diet intervention on body composition and serum biochemical markers in adolescents with obesity: a systematic review and meta-analysis. JOURNAL OF PEDIATRIC ENDOCRINOLOGY & METABOLISM. 2022 NOV 25;35(11):1319-36. https://doi.org/10.1515/jpem-2022-0193
8. Liu J, Lu JD, Liang T, Chen S, Su H. Meta-analysis of effectiveness of different exercises on inflammatory response in obese adolescents. Chin J Sch Health. 2022;43(1):45-52. https://doi.org/10.16835/j.cnki.1000-9817.2022.01.011
9. Papagianni G, Panayiotou C, Vardas M, Balaskas N, Antonopoulos C, Tachmatzidis D, et al. The anti-inflammatory effects of aerobic exercise training in patients with type 2 diabetes: A systematic review and meta-analysis. CYTOKINE. 2023 APR;164. https://doi.org/10.1016/j.cyto.2023.156157
10. Chen X, Li J, Chen C, Cao C. Efficacy of different exercise type on inflammatory cytokines in individuals with overweight or obesity: a network meta-analysis. Chinese Journal of Evidence-Based Medicine. 2024;24(5):565-71. https://doi.org/10.7507/1672-2531.202306137
11. Kadoglou NPE, Georgiou C, Balaskas N, Panayiotou C, Vardas M, Mitsis A, et al. The Anti-Inflammatory Effects of Resistance Training in Patients with Type 2 Diabetes: A Systematic Review and Meta-Analysis. Biomolecules. 2025;15(10). https://doi.org/10.3390/biom15101417

## 4 Supplementary Results: Sensitivity Analyses

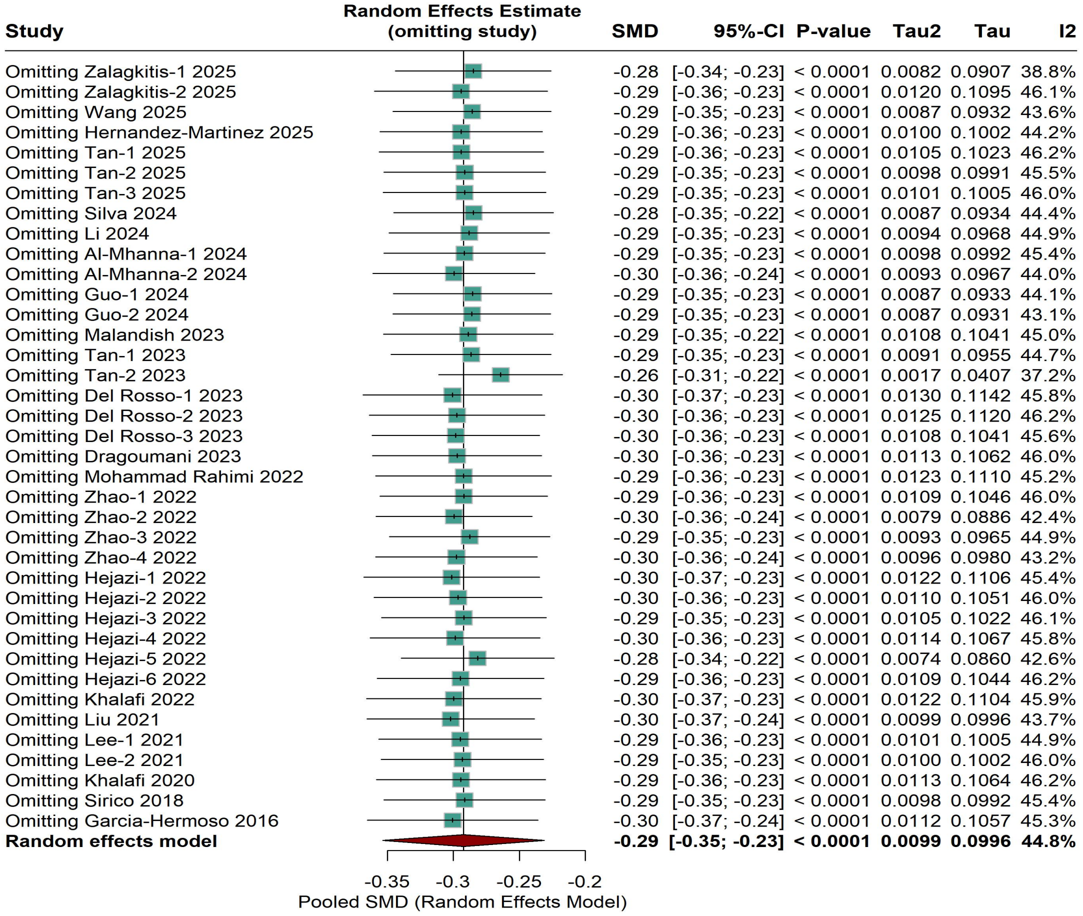


**Supplementary Figure S1. Results of Sensitivity Analysis for CRP**

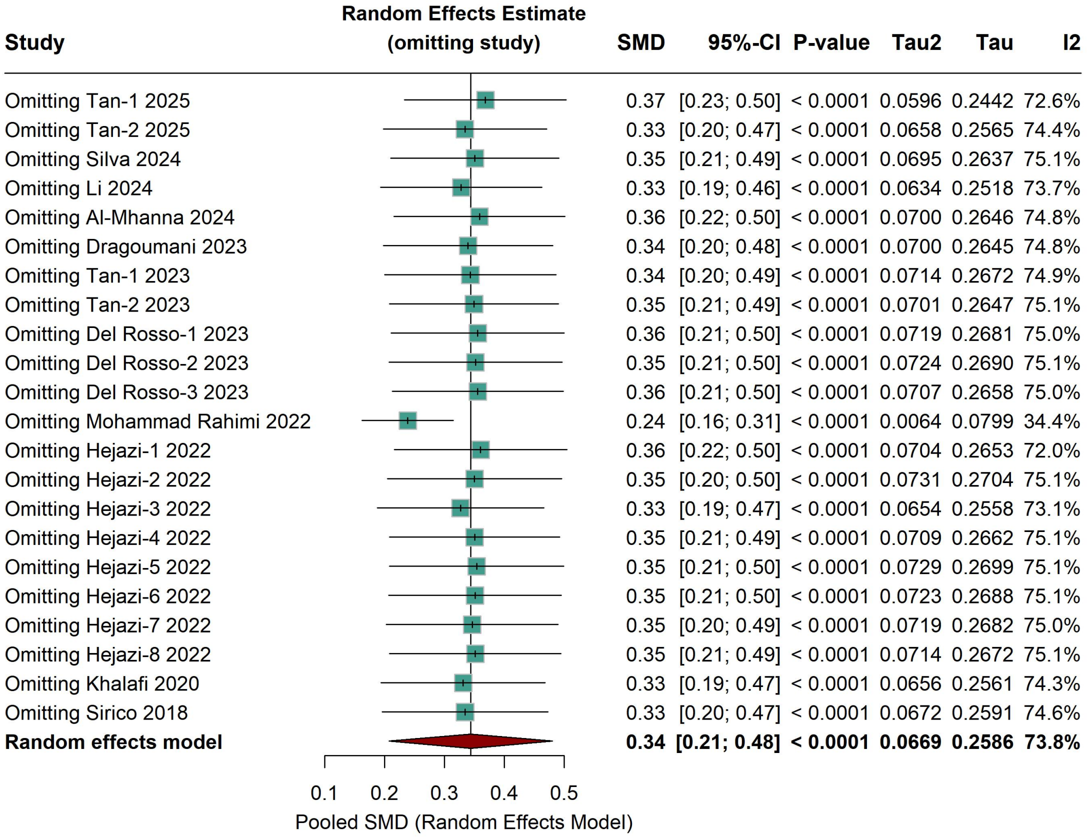


**Supplementary Figure S2. Results of Sensitivity Analysis for Adiponectin**

| Study | SMD | 95%-CI | P-value | Tau2 | Tau | I2 |
|---|---|---|---|---|---|---|
| Omitting Hernandez-Martinez 2025 | -0.46 | [-0.65; -0.27] | < 0.0001 | 0.0877 | 0.2961 | 81.9% |
| Omitting Tan-1 2025 | -0.48 | [-0.68; -0.28] | < 0.0001 | 0.0839 | 0.2897 | 82.2% |
| Omitting Tan-2 2025 | -0.42 | [-0.61; -0.23] | < 0.0001 | 0.0859 | 0.2931 | 82.4% |
| Omitting Silva 2024 | -0.44 | [-0.64; -0.25] | < 0.0001 | 0.0909 | 0.3015 | 82.9% |
| Omitting Li 2024 | -0.42 | [-0.61; -0.23] | < 0.0001 | 0.0829 | 0.2879 | 81.6% |
| Omitting Al-Mhanna 2024 | -0.43 | [-0.63; -0.23] | < 0.0001 | 0.0933 | 0.3055 | 82.6% |
| Omitting Del Rosso-1 2023 | -0.45 | [-0.66; -0.24] | < 0.0001 | 0.1022 | 0.3197 | 82.6% |
| Omitting Del Rosso-2 2023 | -0.44 | [-0.64; -0.23] | < 0.0001 | 0.0973 | 0.3120 | 82.6% |
| Omitting Del Rosso-3 2023 | -0.41 | [-0.60; -0.22] | < 0.0001 | 0.0827 | 0.2875 | 81.9% |
| Omitting Dragoumani 2023 | -0.44 | [-0.64; -0.24] | < 0.0001 | 0.0952 | 0.3085 | 82.8% |
| Omitting Hejazi-1 2022 | -0.49 | [-0.67; -0.31] | < 0.0001 | 0.0609 | 0.2468 | 67.1% |
| Omitting Hejazi-2 2022 | -0.47 | [-0.68; -0.27] | < 0.0001 | 0.0938 | 0.3063 | 82.7% |
| Omitting Hejazi-3 2022 | -0.39 | [-0.59; -0.20] | < 0.0001 | 0.0751 | 0.2741 | 71.8% |
| Omitting Hejazi-4 2022 | -0.45 | [-0.66; -0.25] | < 0.0001 | 0.0981 | 0.3132 | 82.9% |
| Omitting Hejazi-5 2022 | -0.46 | [-0.66; -0.25] | < 0.0001 | 0.0983 | 0.3135 | 82.9% |
| Omitting Hejazi-6 2022 | -0.45 | [-0.66; -0.25] | < 0.0001 | 0.0955 | 0.3091 | 82.9% |
| Omitting Khalafi 2020 | -0.42 | [-0.61; -0.23] | < 0.0001 | 0.0844 | 0.2906 | 82.3% |
| Omitting Sirico 2018 | -0.41 | [-0.60; -0.22] | < 0.0001 | 0.0797 | 0.2823 | 81.9% |
| **Random effects model** | **-0.44** | **[-0.63; -0.25]** | **< 0.0001** | **0.0884** | **0.2973** | **81.8%** |

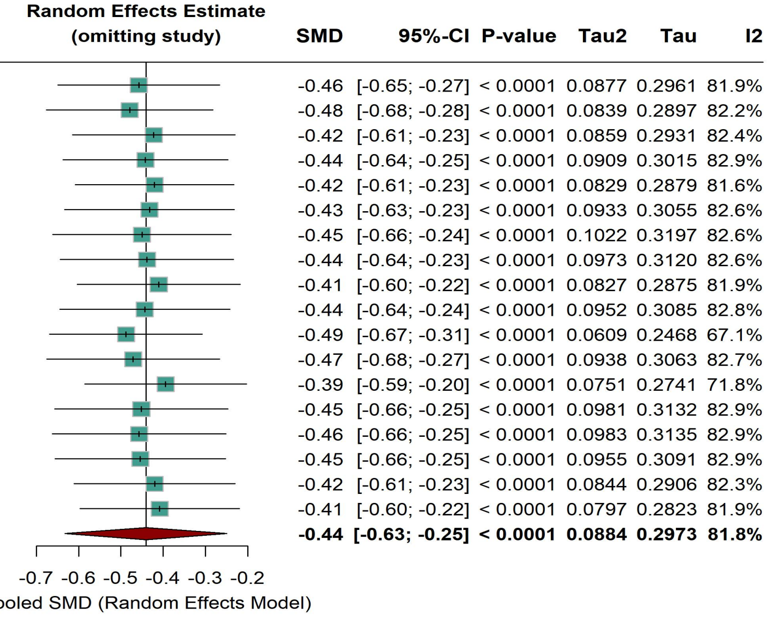


**Supplementary Figure S3. Results of Sensitivity Analysis for Leptin**

| Study | SMD | 95%-CI | P-value | Tau2 | Tau | I2 |
|---|---|---|---|---|---|---|
| Omitting Tan-1 2025 | -0.27 | [-0.40; -0.14] | < 0.0001 | 0.0802 | 0.2831 | 62.7% |
| Omitting Tan-2 2025 | -0.28 | [-0.41; -0.15] | < 0.0001 | 0.0778 | 0.2789 | 61.5% |
| Omitting Tan-3 2025 | -0.26 | [-0.39; -0.14] | < 0.0001 | 0.0763 | 0.2762 | 62.7% |
| Omitting Wang 2025 | -0.27 | [-0.40; -0.14] | < 0.0001 | 0.0808 | 0.2842 | 62.7% |
| Omitting Silva 2024 | -0.23 | [-0.35; -0.12] | < 0.0001 | 0.0509 | 0.2256 | 54.0% |
| Omitting Li 2024 | -0.26 | [-0.38; -0.13] | < 0.0001 | 0.0753 | 0.2743 | 62.1% |
| Omitting Al-Mhanna-1 2024 | -0.25 | [-0.38; -0.13] | < 0.0001 | 0.0733 | 0.2707 | 61.4% |
| Omitting Al-Mhanna-2 2024 | -0.24 | [-0.37; -0.12] | 0.0001 | 0.0656 | 0.2562 | 59.2% |
| Omitting Guo-1 2024 | -0.27 | [-0.40; -0.14] | < 0.0001 | 0.0799 | 0.2826 | 62.7% |
| Omitting Guo-2 2024 | -0.28 | [-0.41; -0.15] | < 0.0001 | 0.0721 | 0.2685 | 61.2% |
| Omitting Malandish 2023 | -0.27 | [-0.41; -0.14] | < 0.0001 | 0.0797 | 0.2823 | 62.1% |
| Omitting Tan-1 2023 | -0.25 | [-0.37; -0.12] | 0.0001 | 0.0692 | 0.2630 | 59.9% |
| Omitting Tan-2 2023 | -0.26 | [-0.39; -0.13] | 0.0001 | 0.0799 | 0.2826 | 62.1% |
| Omitting Tan-3 2023 | -0.26 | [-0.39; -0.13] | < 0.0001 | 0.0763 | 0.2762 | 62.1% |
| Omitting Al-Mhanna 2023 | -0.26 | [-0.39; -0.13] | < 0.0001 | 0.0767 | 0.2769 | 62.5% |
| Omitting Del Rosso-1 2023 | -0.25 | [-0.38; -0.12] | 0.0001 | 0.0730 | 0.2701 | 60.3% |
| Omitting Del Rosso-2 2023 | -0.27 | [-0.40; -0.14] | < 0.0001 | 0.0793 | 0.2816 | 62.6% |
| Omitting Del Rosso-3 2023 | -0.26 | [-0.39; -0.12] | 0.0001 | 0.0798 | 0.2825 | 61.4% |
| Omitting Zhao-1 2022 | -0.28 | [-0.41; -0.16] | < 0.0001 | 0.0662 | 0.2573 | 59.9% |
| Omitting Zhao-2 2022 | -0.28 | [-0.40; -0.16] | < 0.0001 | 0.0645 | 0.2540 | 59.0% |
| Omitting Zhao-3 2022 | -0.28 | [-0.40; -0.15] | < 0.0001 | 0.0761 | 0.2758 | 62.1% |
| Omitting Khalafi 2022 | -0.27 | [-0.41; -0.14] | < 0.0001 | 0.0797 | 0.2822 | 61.9% |
| Omitting Hejazi-1 2022 | -0.27 | [-0.41; -0.14] | < 0.0001 | 0.0814 | 0.2854 | 61.7% |
| Omitting Hejazi-2 2022 | -0.28 | [-0.41; -0.15] | < 0.0001 | 0.0763 | 0.2762 | 62.0% |
| Omitting Hejazi-3 2022 | -0.27 | [-0.40; -0.14] | < 0.0001 | 0.0795 | 0.2820 | 62.7% |
| Omitting Hejazi-4 2022 | -0.27 | [-0.40; -0.13] | < 0.0001 | 0.0831 | 0.2883 | 62.7% |
| Omitting Hejazi-5 2022 | -0.27 | [-0.40; -0.14] | < 0.0001 | 0.0788 | 0.2807 | 62.8% |
| Omitting Hejazi-6 2022 | -0.26 | [-0.39; -0.13] | 0.0001 | 0.0770 | 0.2775 | 62.1% |
| Omitting Liu 2021 | -0.27 | [-0.41; -0.14] | < 0.0001 | 0.0821 | 0.2866 | 62.5% |
| Omitting Lee 2021 | -0.25 | [-0.37; -0.12] | 0.0001 | 0.0698 | 0.2642 | 60.3% |
| Omitting Khalafi 2020 | -0.25 | [-0.38; -0.12] | 0.0001 | 0.0755 | 0.2748 | 61.0% |
| Omitting Sirico 2018 | -0.27 | [-0.40; -0.14] | < 0.0001 | 0.0765 | 0.2766 | 62.6% |
| **Random effects model** | **-0.26** | **[-0.39; -0.14]** | **< 0.0001** | **0.0753** | **0.2745** | **61.5%** |

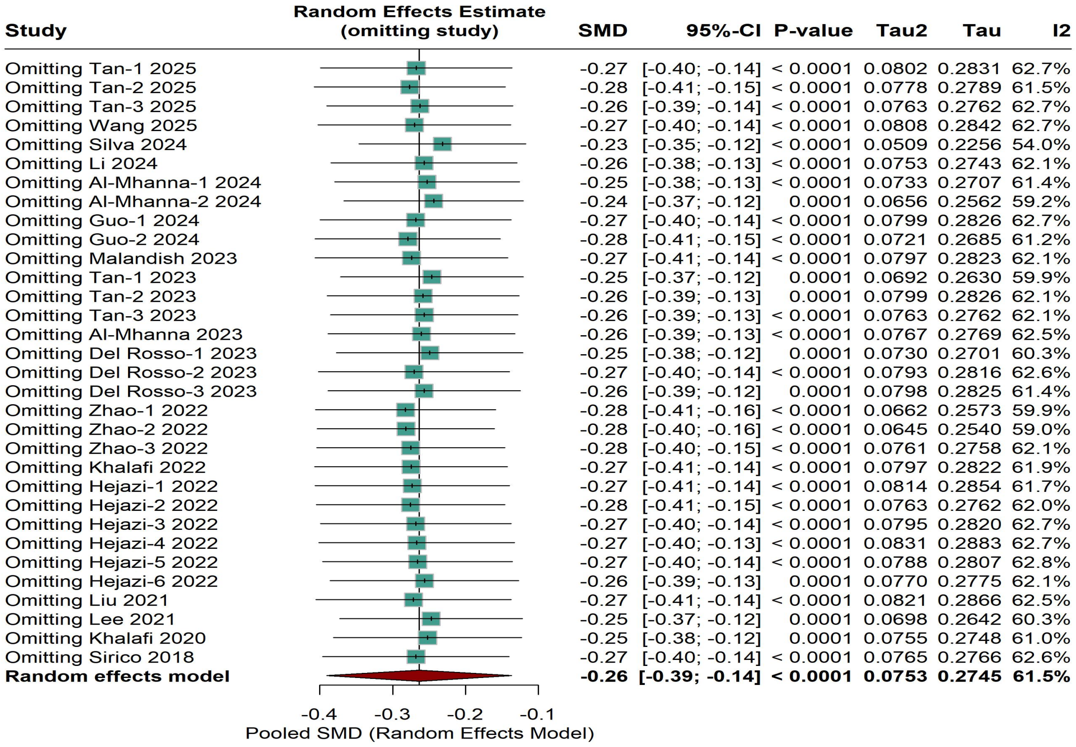


**Supplementary Figure S4. Results of Sensitivity Analysis for TNF-α**

| Study | SMD | 95%-CI | P-value | Tau2 | Tau | I2 |
|---|---|---|---|---|---|---|
| Omitting Zalagkitis 2025 | -0.42 | [-0.73; -0.11] | 0.0087 | 0.0833 | 0.2886 | 50.3% |
| Omitting Malandish 2023 | -0.55 | [-0.75; -0.35] | < 0.0001 | 0 | 0 | 0% |
| Omitting Del Rosso-1 2023 | -0.46 | [-0.74; -0.18] | 0.0012 | 0.0658 | 0.2566 | 48.3% |
| Omitting Del Rosso-2 2023 | -0.44 | [-0.72; -0.16] | 0.0023 | 0.0696 | 0.2639 | 51.0% |
| Omitting Del Rosso-3 2023 | -0.38 | [-0.65; -0.11] | 0.0054 | 0.0537 | 0.2318 | 40.4% |
| Omitting Ding-1 2022 | -0.41 | [-0.74; -0.09] | 0.0122 | 0.0862 | 0.2936 | 48.4% |
| Omitting Ding-2 2022 | -0.45 | [-0.74; -0.16] | 0.0022 | 0.0714 | 0.2673 | 50.5% |
| Omitting Ding-3 2022 | -0.36 | [-0.63; -0.10] | 0.0071 | 0.0458 | 0.2140 | 34.9% |
| **Random effects model** | **-0.43** | **[-0.70; -0.17]** | **0.0013** | **0.0597** | **0.2443** | **43.0%** |

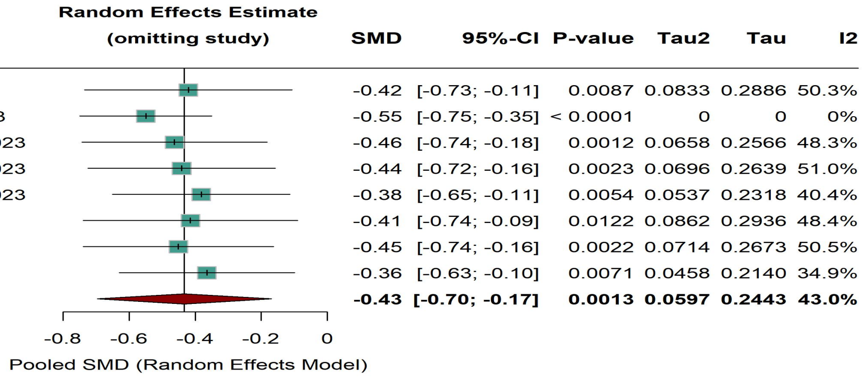


**Supplementary Figure S5. Results of Sensitivity Analysis for IL-1β**

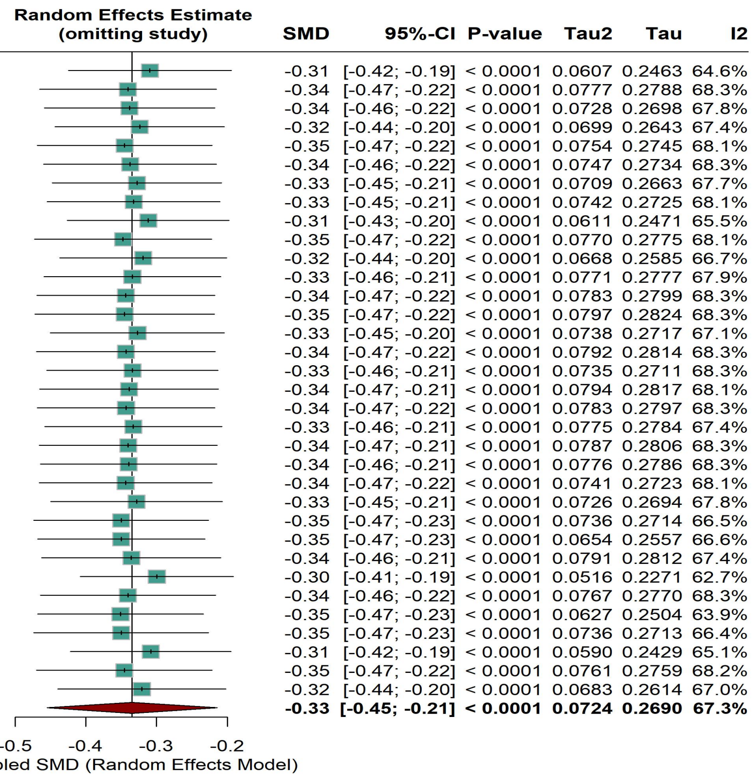


| Study | SMD | 95%-CI | P-value | Tau2 | Tau | I2 |
|---|---|---|---|---|---|---|
| Omitting Zalagkitis-1 2025 | -0.31 | [-0.42; -0.19] | < 0.0001 | 0.0607 | 0.2463 | 64.6% |
| Omitting Zalagkitis-2 2025 | -0.34 | [-0.47; -0.22] | < 0.0001 | 0.0777 | 0.2788 | 68.3% |
| Omitting Wang 2025 | -0.34 | [-0.46; -0.22] | < 0.0001 | 0.0728 | 0.2698 | 67.8% |
| Omitting Hernandez-Martinez 2025 | -0.32 | [-0.44; -0.20] | < 0.0001 | 0.0699 | 0.2643 | 67.4% |
| Omitting Tan-1 2025 | -0.35 | [-0.47; -0.22] | < 0.0001 | 0.0754 | 0.2745 | 68.1% |
| Omitting Tan-2 2025 | -0.34 | [-0.46; -0.22] | < 0.0001 | 0.0747 | 0.2734 | 68.3% |
| Omitting Tan-3 2025 | -0.33 | [-0.45; -0.21] | < 0.0001 | 0.0709 | 0.2663 | 67.7% |
| Omitting Silva 2024 | -0.33 | [-0.45; -0.21] | < 0.0001 | 0.0742 | 0.2725 | 68.1% |
| Omitting Li 2024 | -0.31 | [-0.43; -0.20] | < 0.0001 | 0.0611 | 0.2471 | 65.5% |
| Omitting Al-Mhanna-1 2024 | -0.35 | [-0.47; -0.22] | < 0.0001 | 0.0770 | 0.2775 | 68.1% |
| Omitting Al-Mhanna-2 2024 | -0.32 | [-0.44; -0.20] | < 0.0001 | 0.0668 | 0.2585 | 66.7% |
| Omitting Guo-1 2024 | -0.33 | [-0.46; -0.21] | < 0.0001 | 0.0771 | 0.2777 | 67.9% |
| Omitting Guo-2 2024 | -0.34 | [-0.47; -0.22] | < 0.0001 | 0.0783 | 0.2799 | 68.3% |
| Omitting Malandish 2023 | -0.35 | [-0.47; -0.22] | < 0.0001 | 0.0797 | 0.2824 | 68.3% |
| Omitting Tan-1 2023 | -0.33 | [-0.45; -0.20] | < 0.0001 | 0.0738 | 0.2717 | 67.1% |
| Omitting Tan-2 2023 | -0.34 | [-0.47; -0.22] | < 0.0001 | 0.0792 | 0.2814 | 68.3% |
| Omitting Tan-3 2023 | -0.33 | [-0.46; -0.21] | < 0.0001 | 0.0735 | 0.2711 | 68.3% |
| Omitting Del Rosso-1 2023 | -0.34 | [-0.47; -0.21] | < 0.0001 | 0.0794 | 0.2817 | 68.1% |
| Omitting Del Rosso-2 2023 | -0.34 | [-0.47; -0.22] | < 0.0001 | 0.0783 | 0.2797 | 68.3% |
| Omitting Del Rosso-3 2023 | -0.33 | [-0.46; -0.21] | < 0.0001 | 0.0775 | 0.2784 | 67.4% |
| Omitting Mohammad Rahimi 2022 | -0.34 | [-0.47; -0.21] | < 0.0001 | 0.0787 | 0.2806 | 68.3% |
| Omitting Zhao-1 2022 | -0.34 | [-0.46; -0.21] | < 0.0001 | 0.0776 | 0.2786 | 68.3% |
| Omitting Zhao-2 2022 | -0.34 | [-0.47; -0.22] | < 0.0001 | 0.0741 | 0.2723 | 68.1% |
| Omitting Zhao-3 2022 | -0.33 | [-0.45; -0.21] | < 0.0001 | 0.0726 | 0.2694 | 67.8% |
| Omitting Hejazi-1 2022 | -0.35 | [-0.47; -0.23] | < 0.0001 | 0.0736 | 0.2714 | 66.5% |
| Omitting Hejazi-2 2023 | -0.35 | [-0.47; -0.23] | < 0.0001 | 0.0654 | 0.2557 | 66.6% |
| Omitting Hejazi-3 2022 | -0.34 | [-0.46; -0.21] | < 0.0001 | 0.0791 | 0.2812 | 67.4% |
| Omitting Hejazi-4 2023 | -0.30 | [-0.41; -0.19] | < 0.0001 | 0.0516 | 0.2271 | 62.7% |
| Omitting Hejazi-5 2024 | -0.34 | [-0.46; -0.22] | < 0.0001 | 0.0767 | 0.2770 | 68.3% |
| Omitting Khalafi 2022 | -0.35 | [-0.47; -0.23] | < 0.0001 | 0.0627 | 0.2504 | 63.9% |
| Omitting Liu 2021 | -0.35 | [-0.47; -0.23] | < 0.0001 | 0.0736 | 0.2713 | 66.4% |
| Omitting Lee 2021 | -0.31 | [-0.42; -0.19] | < 0.0001 | 0.0590 | 0.2429 | 65.1% |
| Omitting Khalafi 2020 | -0.35 | [-0.47; -0.22] | < 0.0001 | 0.0761 | 0.2759 | 68.2% |
| Omitting Sirico 2018 | -0.32 | [-0.44; -0.20] | < 0.0001 | 0.0683 | 0.2614 | 67.0% |
| **Random effects model** | **-0.33** | **[-0.45; -0.21]** | **< 0.0001** | **0.0724** | **0.2690** | **67.3%** |

**Supplementary Figure S6. Results of Sensitivity Analysis for IL-6**

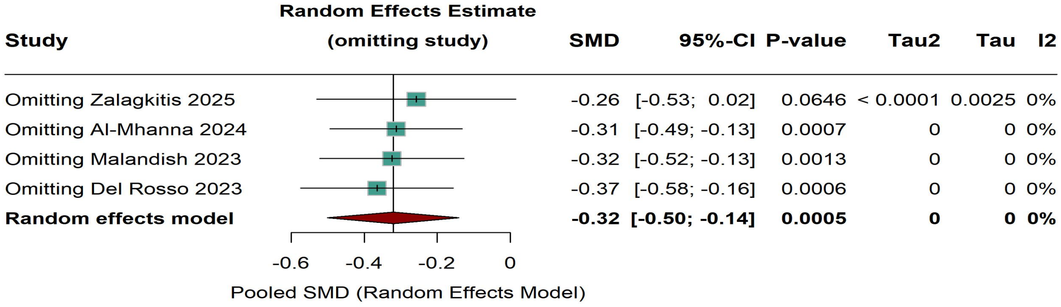


| Study | SMD | 95%-CI | P-value | Tau2 | Tau | I2 |
|---|---|---|---|---|---|---|
| Omitting Zalagkitis 2025 | -0.26 | [-0.53; 0.02] | 0.0646 | < 0.0001 | 0.0025 | 0% |
| Omitting Al-Mhanna 2024 | -0.31 | [-0.49; -0.13] | 0.0007 | 0 | 0 | 0% |
| Omitting Malandish 2023 | -0.32 | [-0.52; -0.13] | 0.0013 | 0 | 0 | 0% |
| Omitting Del Rosso 2023 | -0.37 | [-0.58; -0.16] | 0.0006 | 0 | 0 | 0% |
| **Random effects model** | **-0.32** | **[-0.50; -0.14]** | **0.0005** | **0** | **0** | **0%** |

**Supplementary Figure S7. Results of Sensitivity Analysis for IL-8**

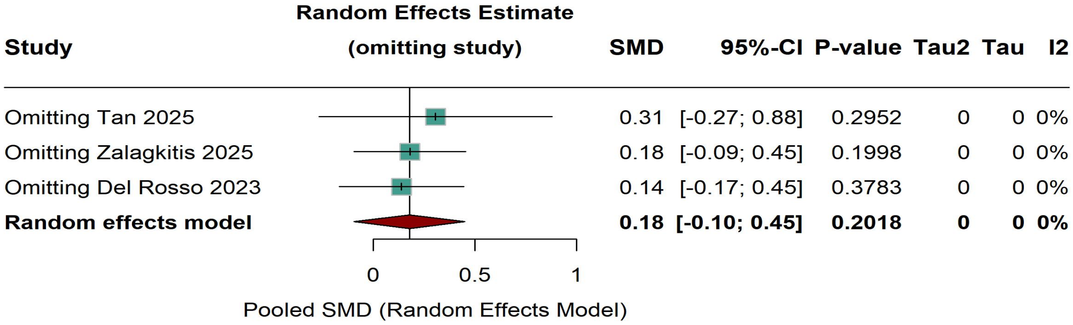


| Study | SMD | 95%-CI | P-value | Tau2 | Tau | I2 |
|---|---|---|---|---|---|---|
| Omitting Tan 2025 | 0.31 | [-0.27; 0.88] | 0.2952 | 0 | 0 | 0% |
| Omitting Zalagkitis 2025 | 0.18 | [-0.09; 0.45] | 0.1998 | 0 | 0 | 0% |
| Omitting Del Rosso 2023 | 0.14 | [-0.17; 0.45] | 0.3783 | 0 | 0 | 0% |
| **Random effects model** | **0.18** | **[-0.10; 0.45]** | **0.2018** | **0** | **0** | **0%** |

**Supplementary Figure S8. Results of Sensitivity Analysis for IL-10**

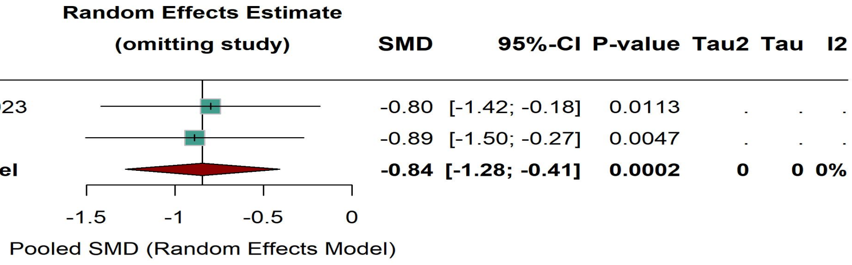


| Study | SMD | 95%-CI | P-value | Tau2 | Tau | I2 |
|---|---|---|---|---|---|---|
| Omitting Del Rosso 2023 | -0.80 | [-1.42; -0.18] | 0.0113 | . | . | . |
| Omitting Ding 2022 | -0.89 | [-1.50; -0.27] | 0.0047 | . | . | . |
| **Random effects model** | **-0.84** | **[-1.28; -0.41]** | **0.0002** | **0** | **0** | **0%** |

**Supplementary Figure S9. Results of Sensitivity Analysis for IL-18**

# 5 Supplementary Results: Publication Bias Analyses

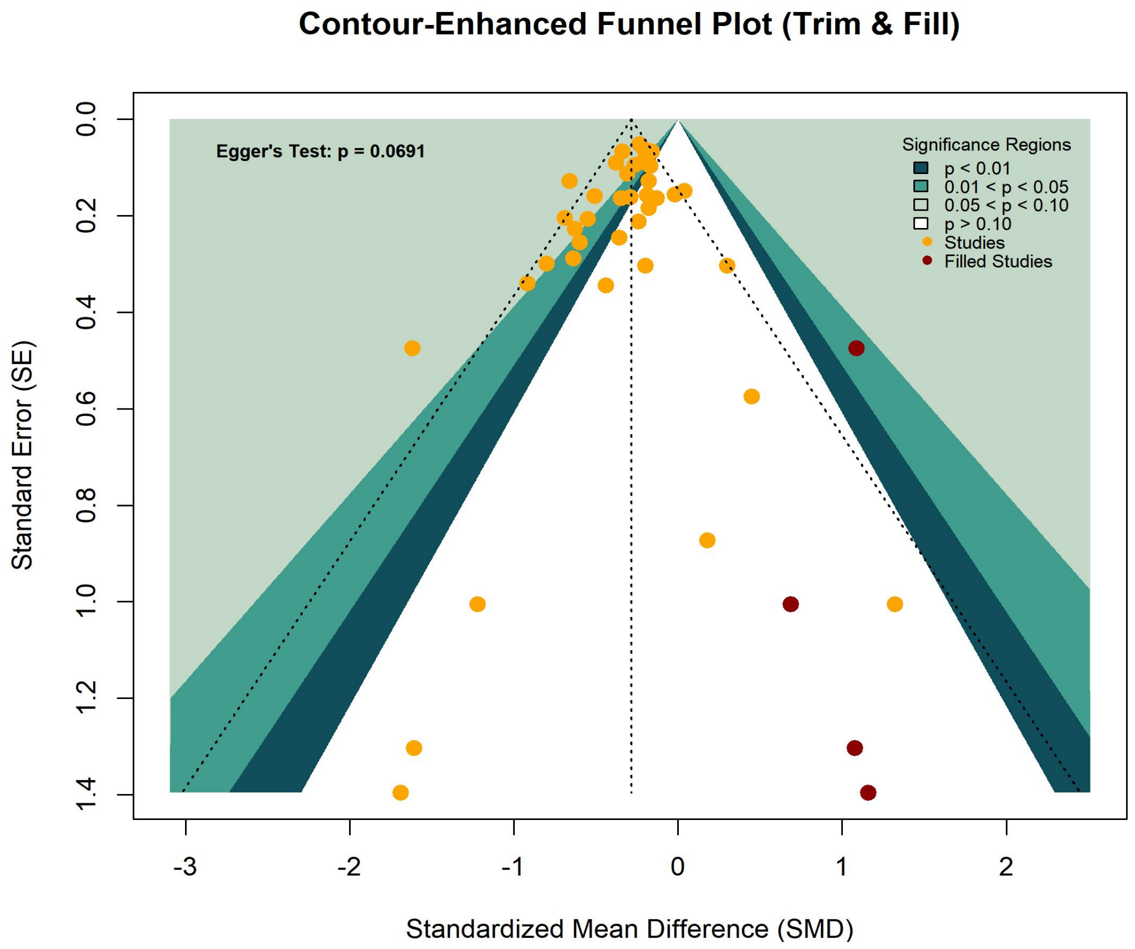


**Supplementary Figure S10. Results of Publication Bias Analysis for CRP**

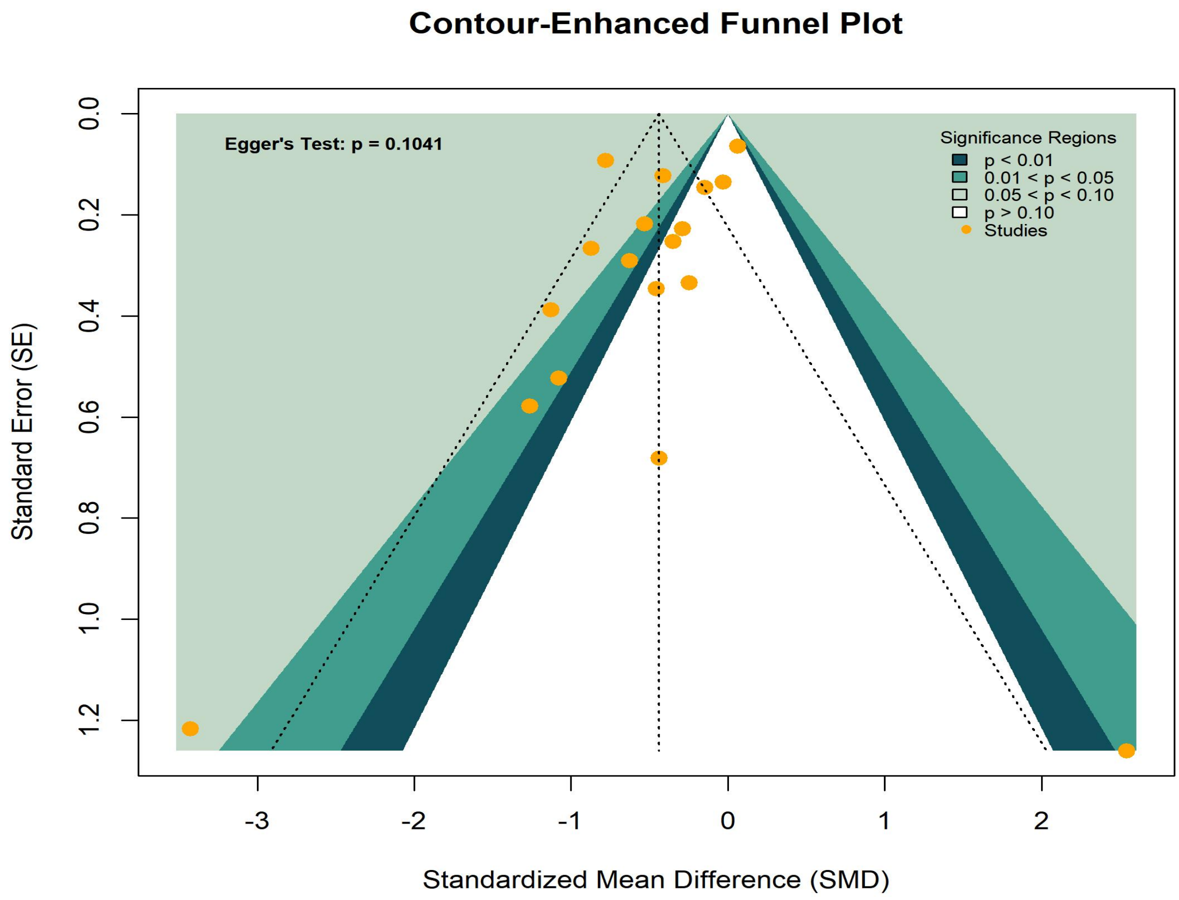


**Supplementary Figure S11. Results of Publication Bias Analysis for Leptin**

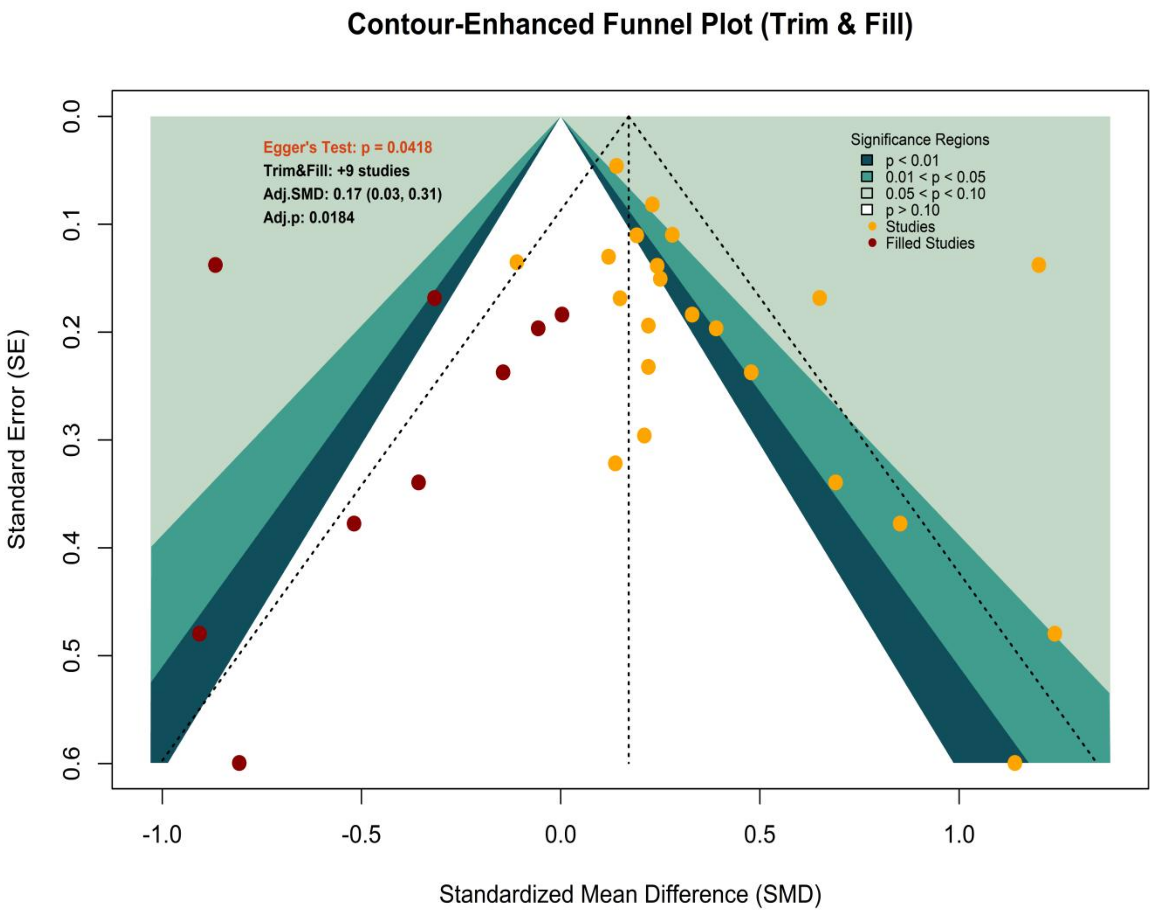


**Supplementary Figure S12. Results of Publication Bias Analysis for Adiponectin**

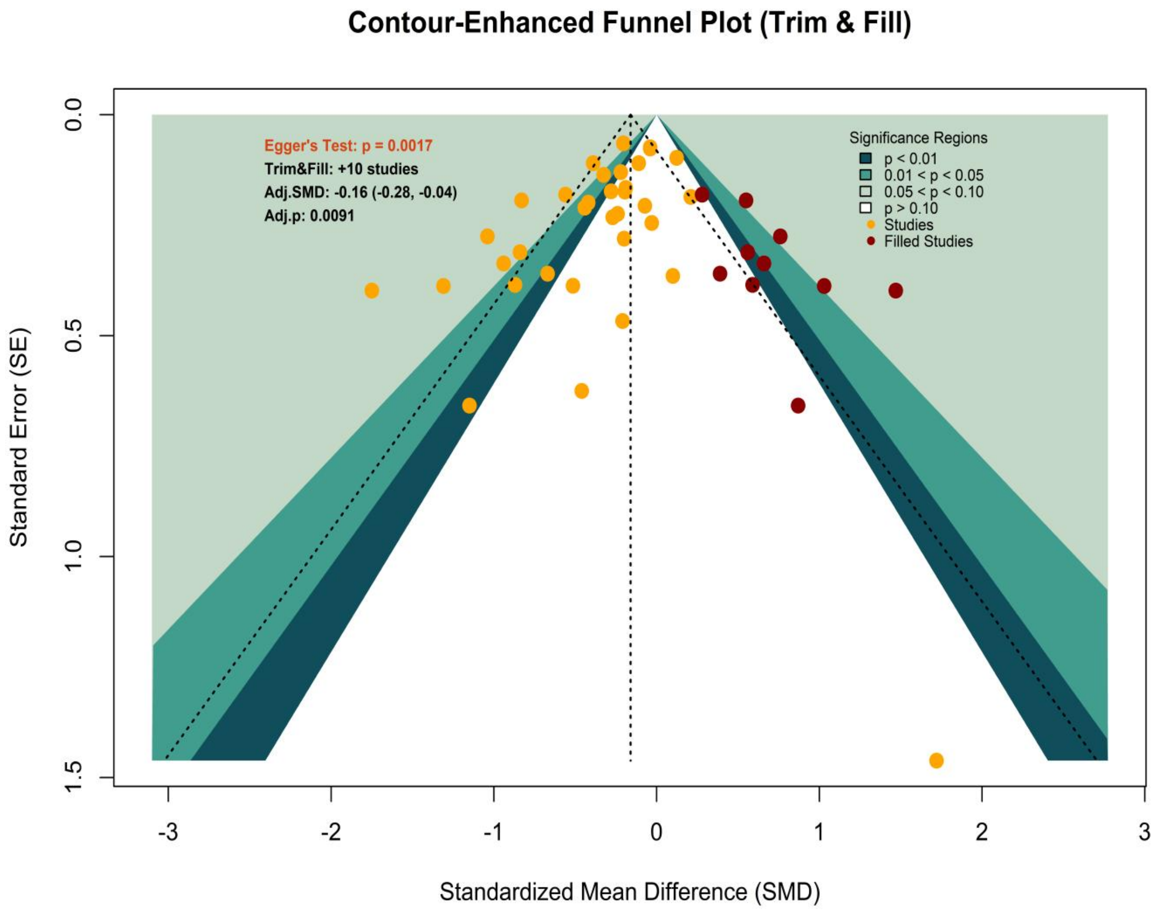


**Supplementary Figure S13. Results of Publication Bias Analysis for IL-6**

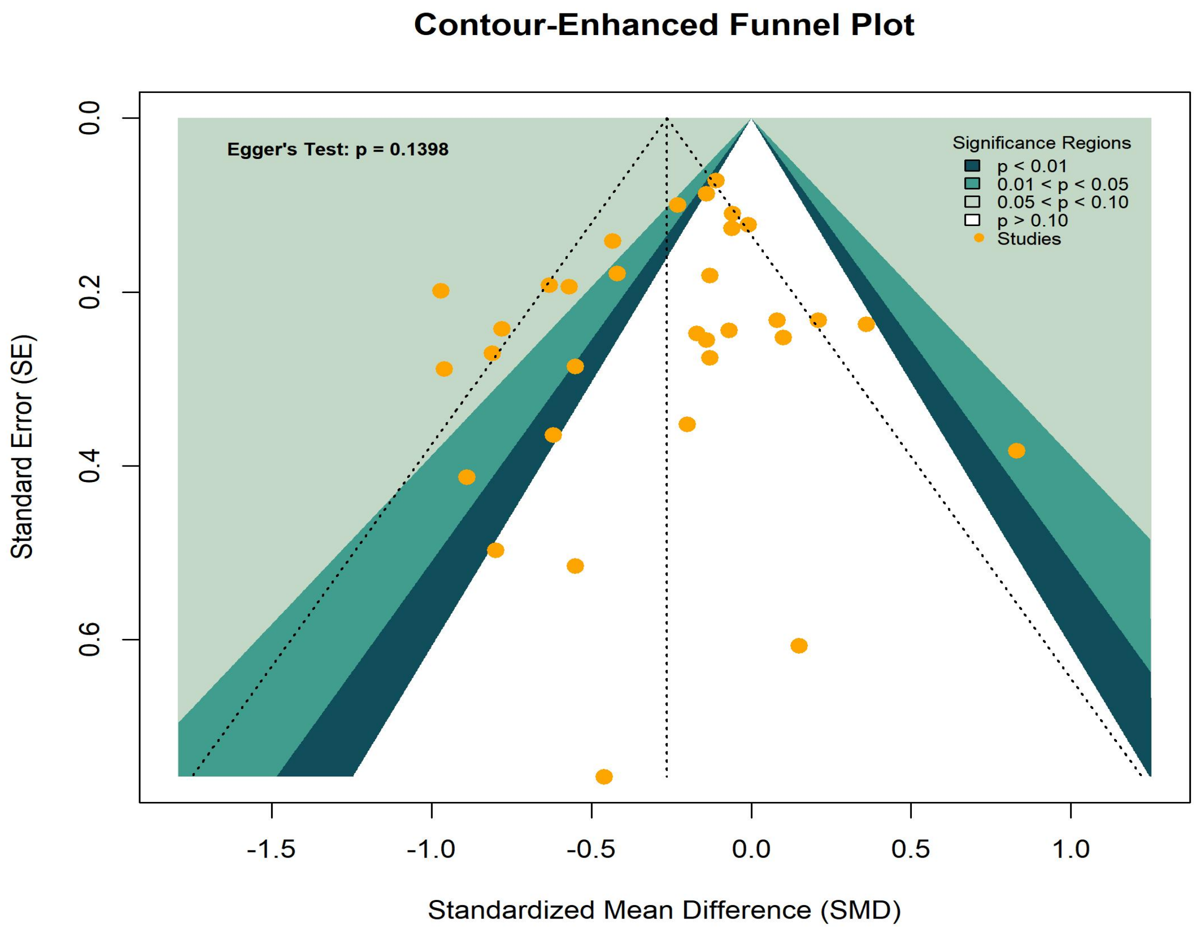


**Supplementary Figure S14. Results of Publication Bias Analysis for TNF-α**

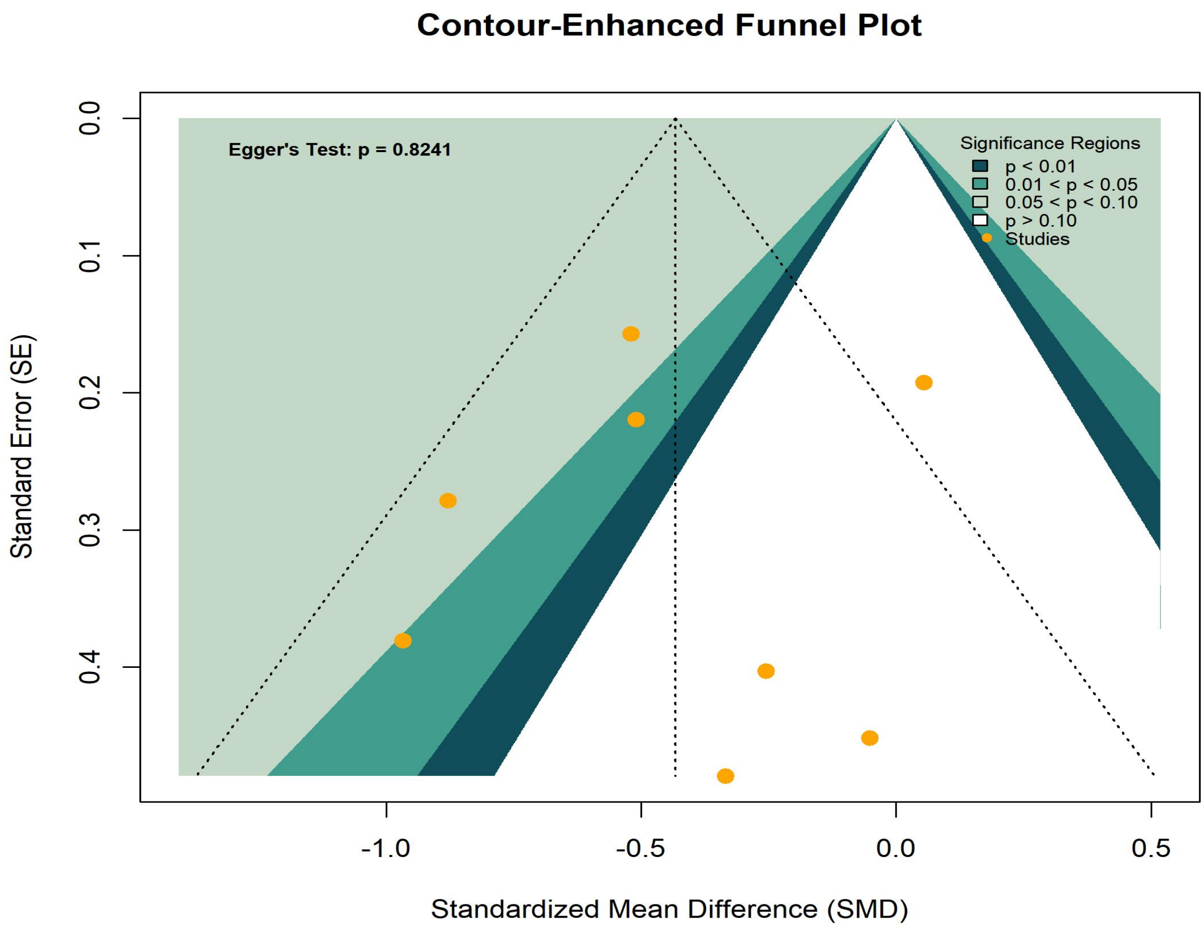


**Supplementary Figure S15. Results of Publication Bias Analysis for IL-1β**

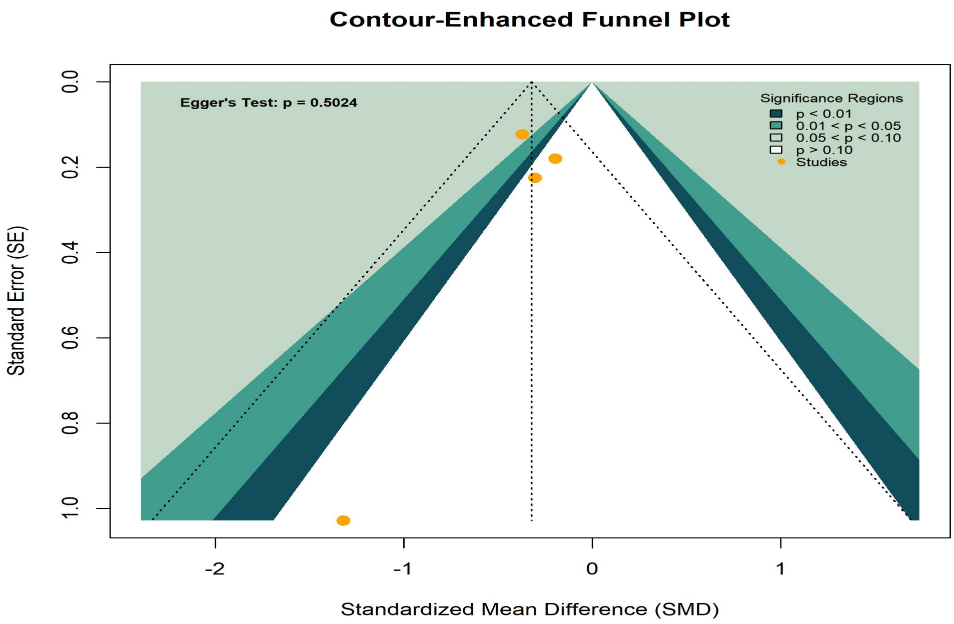


**Supplementary Figure S16. Results of Publication Bias Analysis for IL-8**

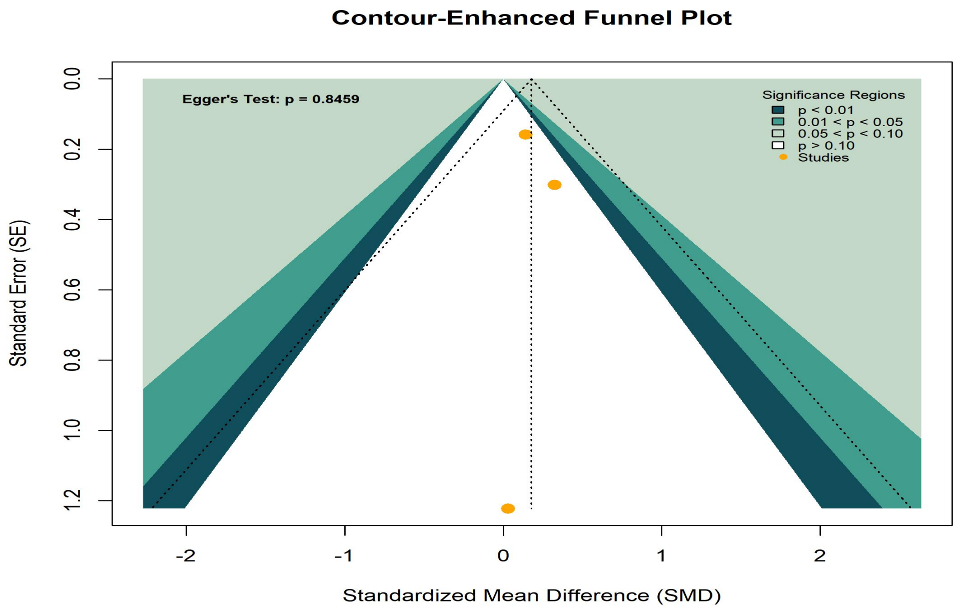


**Supplementary Figure S17. Results of Publication Bias Analysis for IL-10**

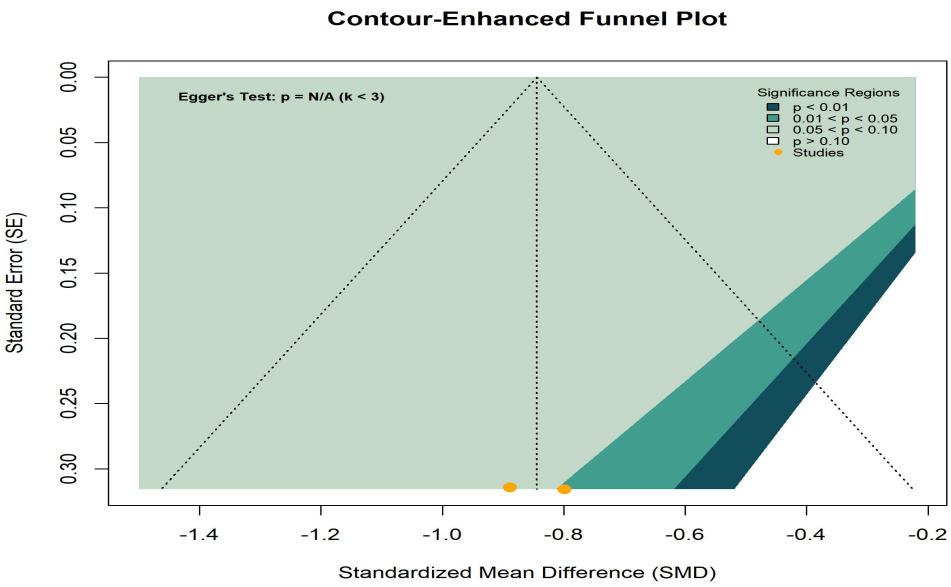


**Suplementary Figure S18. Results of Publication Bias Analysis for IL-18**

## 6 Supplementary Results: Subgroup Analyses

### 6.1 For CRP

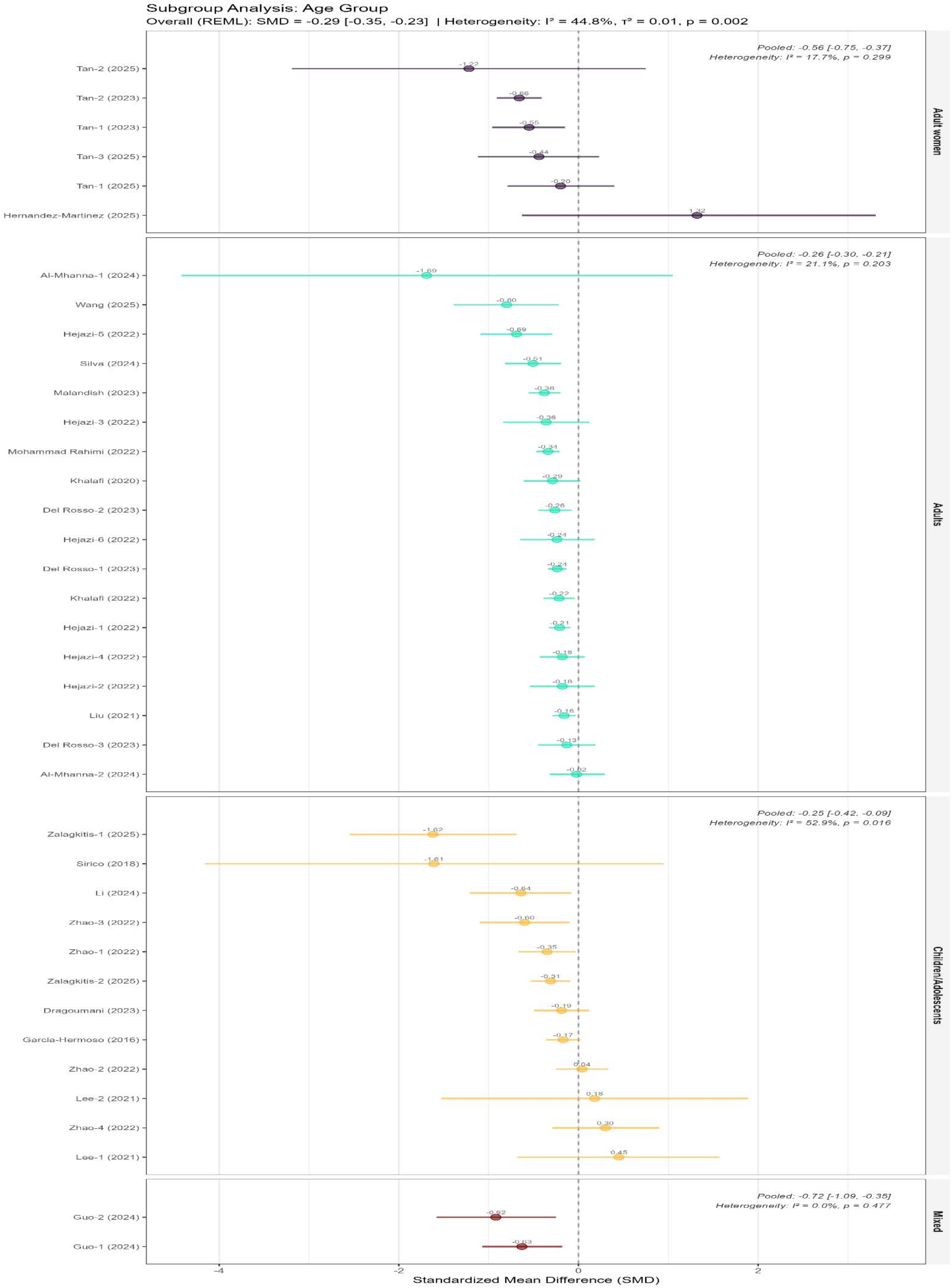


**Supplementary Figure S19. Results of Subgroup Analysis for CRP by Age Group**

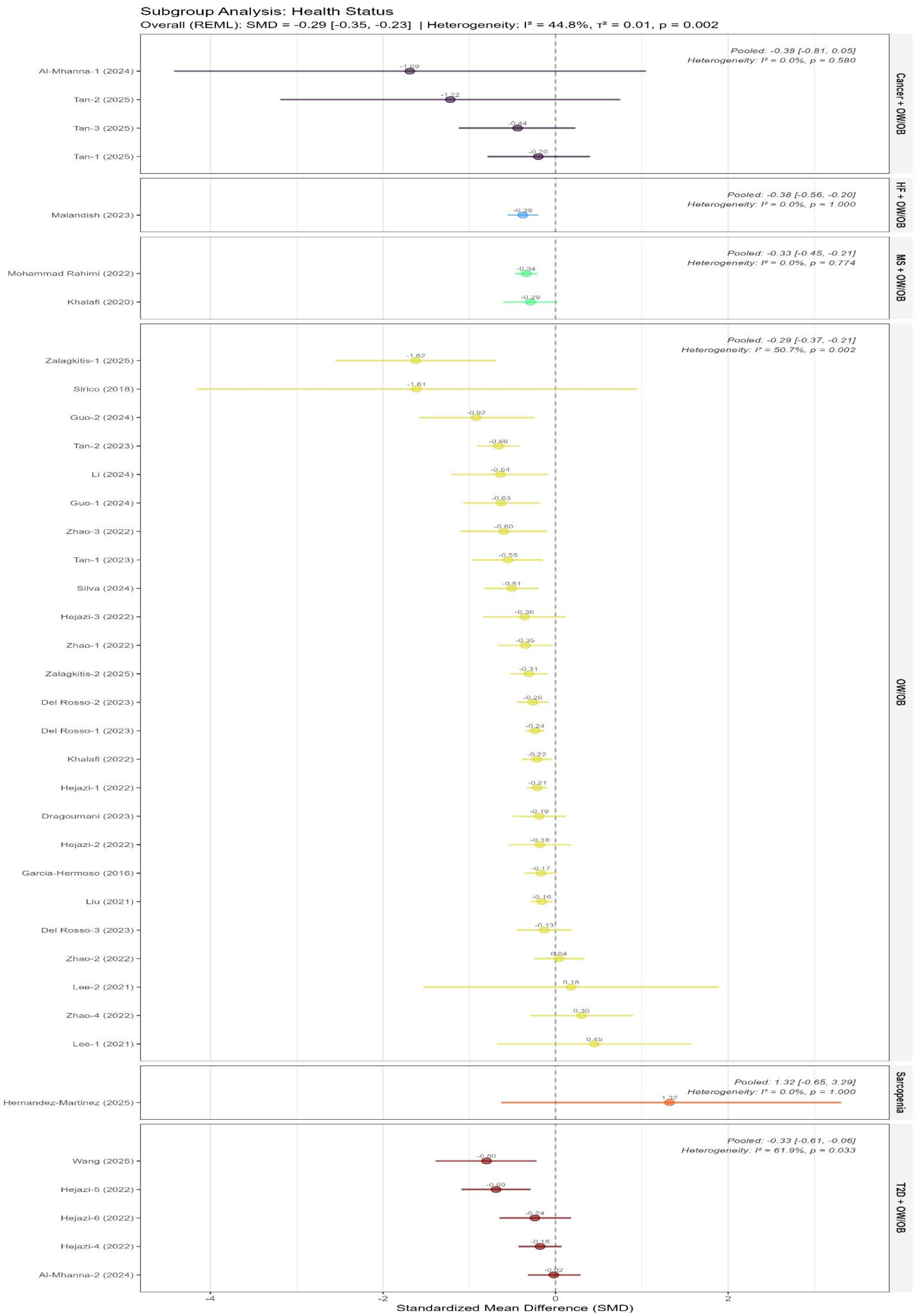


**Supplementary Figure S20. Results of Subgroup Analysis for CRP by Health Status**

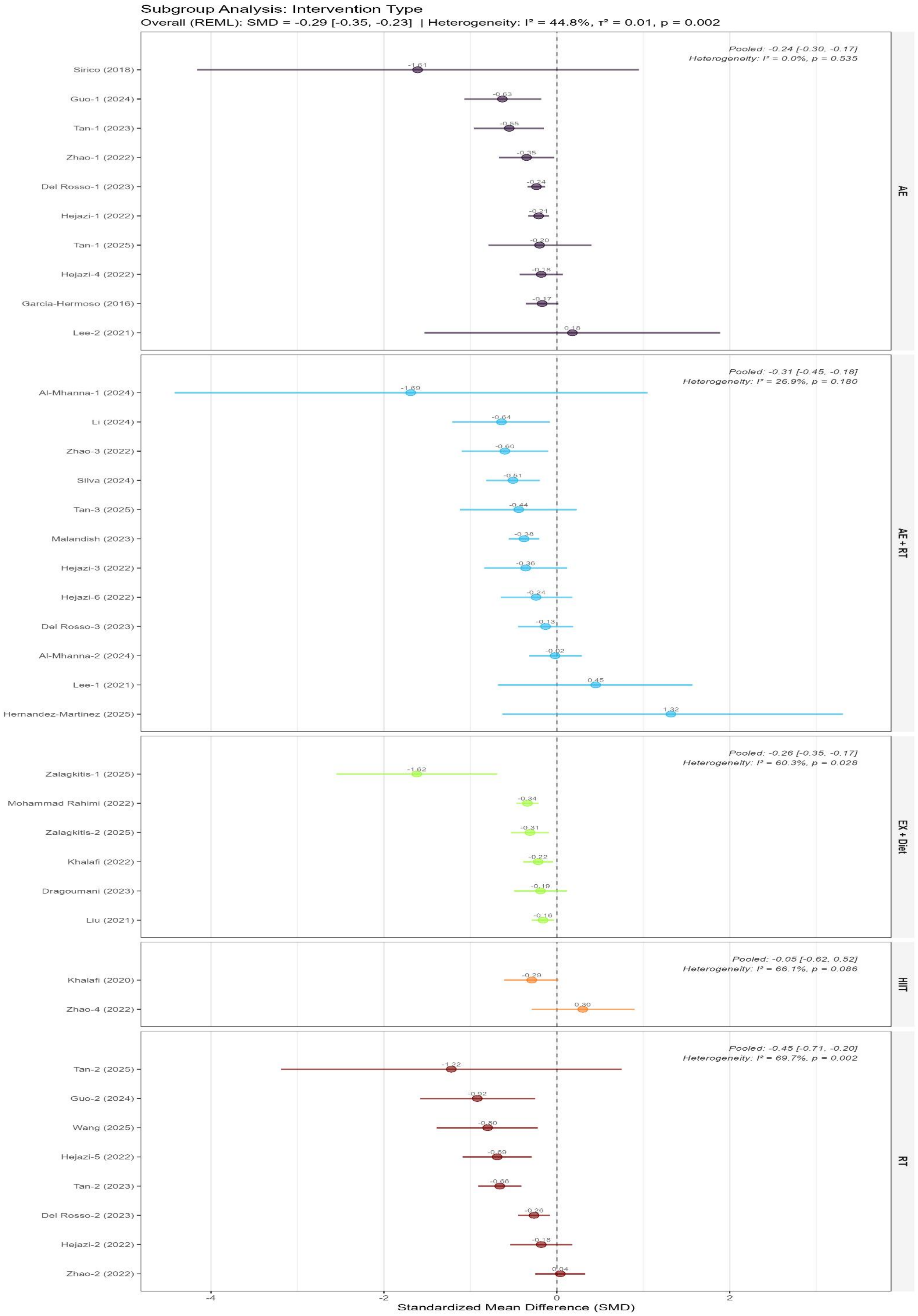


**Supplementary Figure S21. Results of Subgroup Analysis for CRP by Intervention Type**

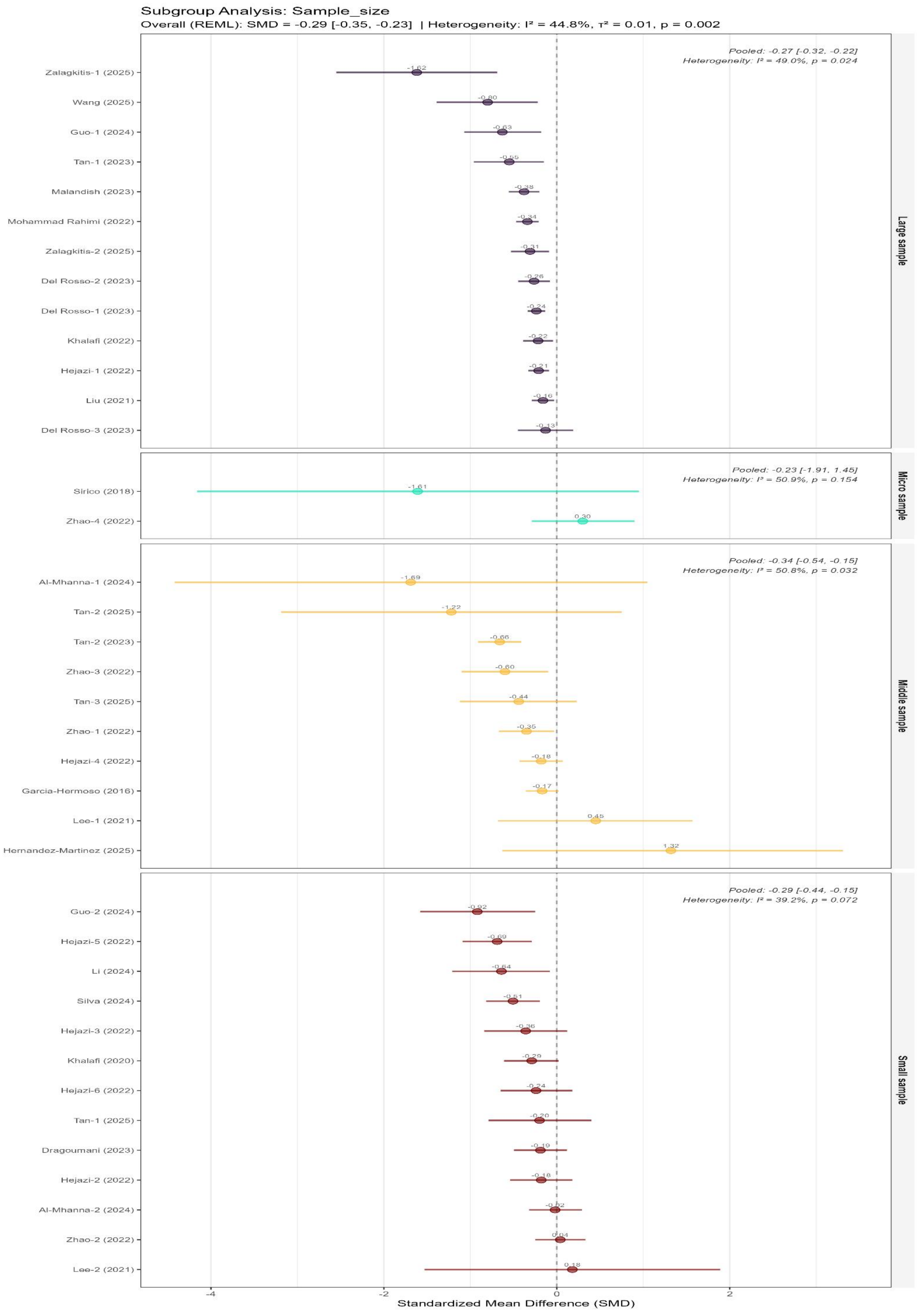


**Supplementary Figure S22. Results of Subgroup Analysis for CRP by Sample Size**

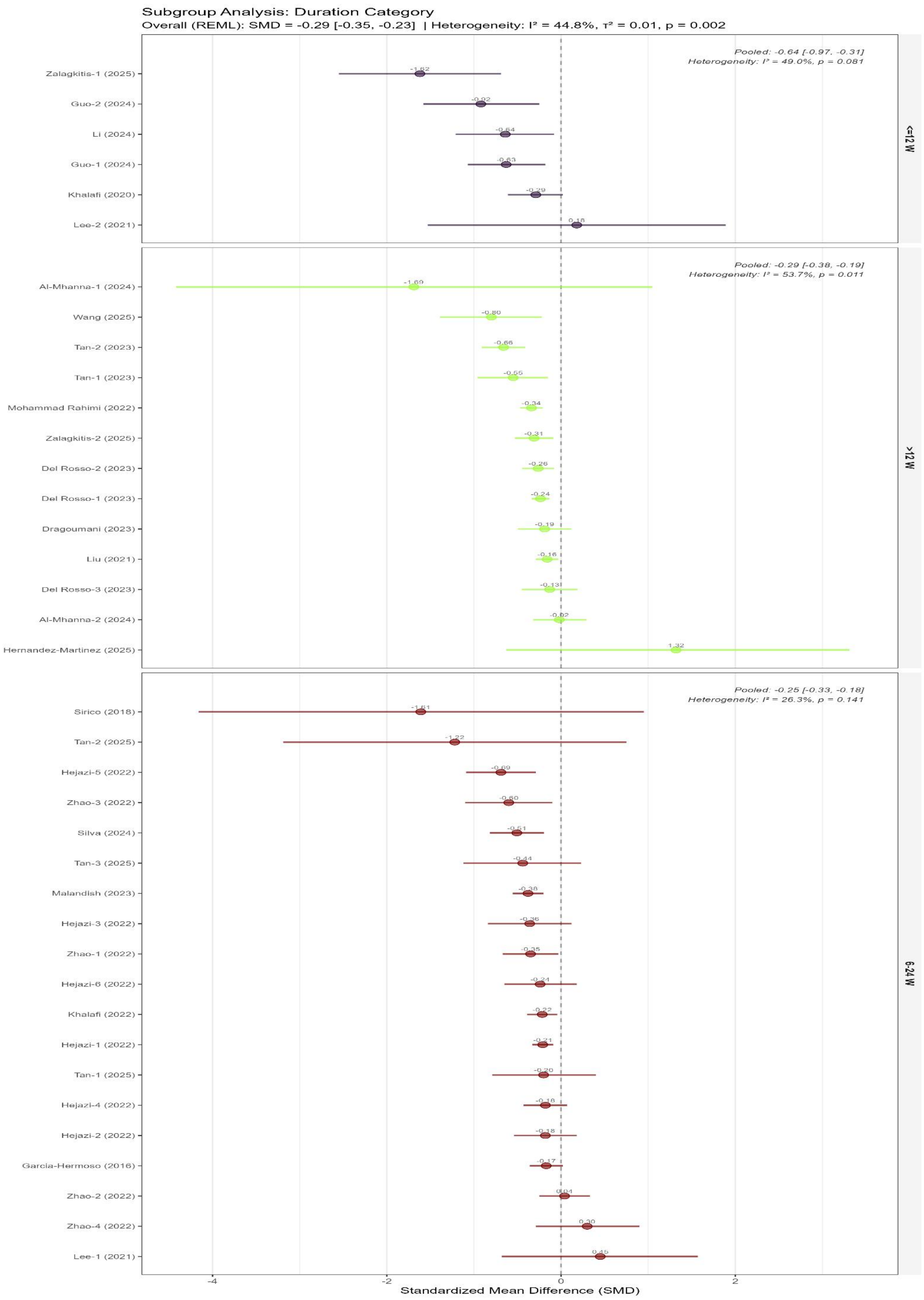


**Supplementary Figure S23. Results of Subgroup Analysis for CRP by Intervention Duration**

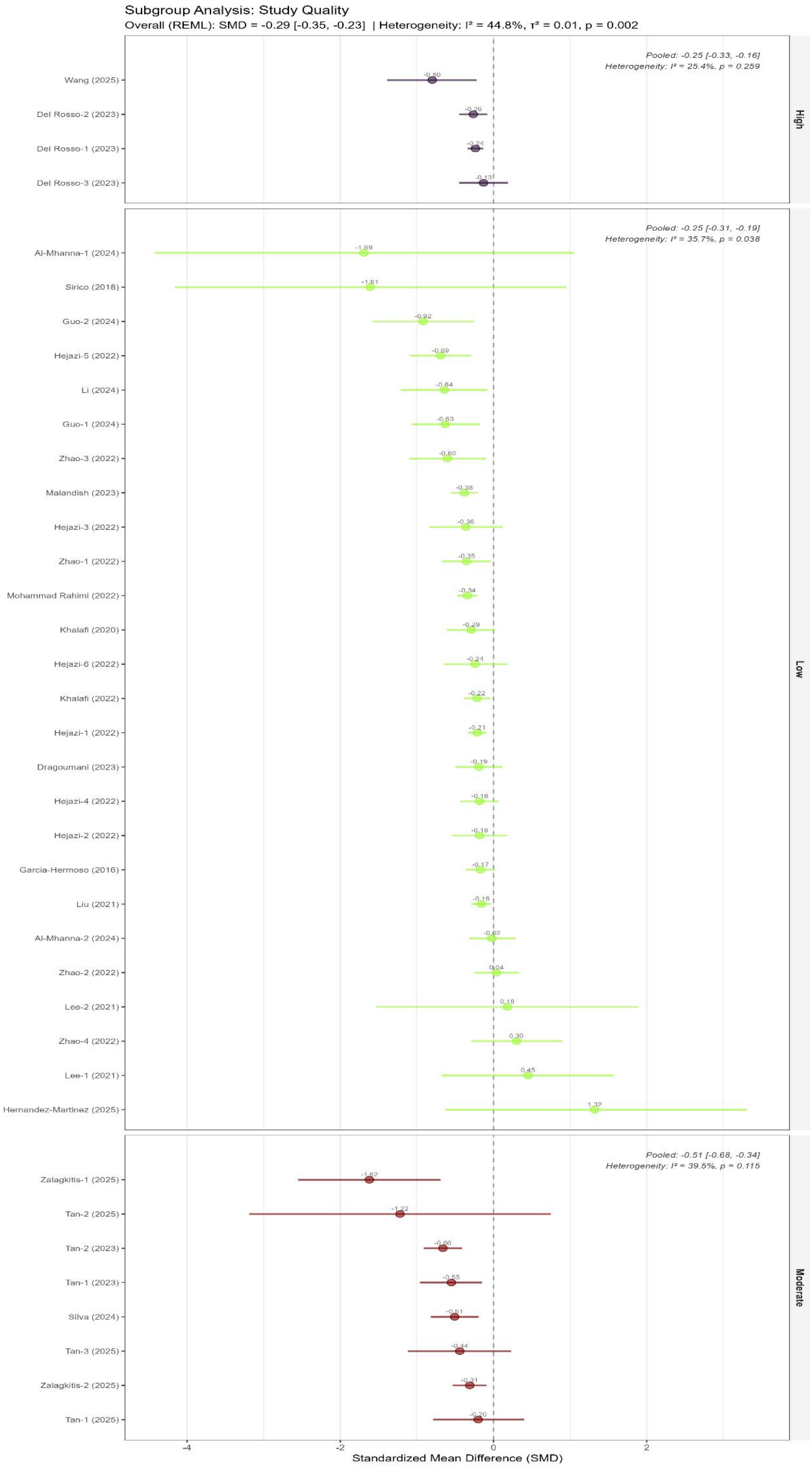


**Supplementary Figure S24. Results of Subgroup Analysis for CRP by Study Quality**

## 6.2 For Adiponectin

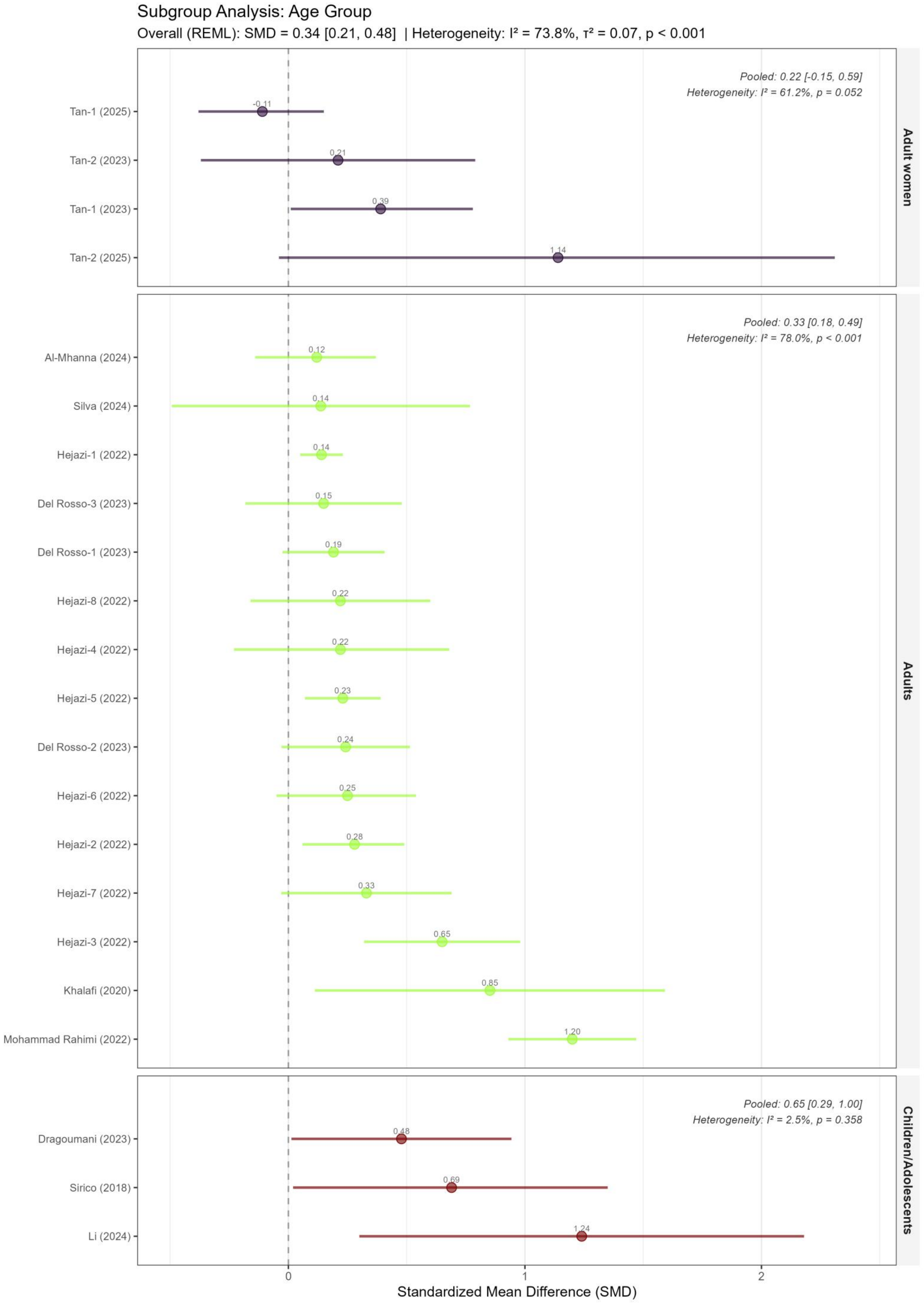


**Supplementary Figure S25. Results of Subgroup Analysis for Adiponectin by Age Group**

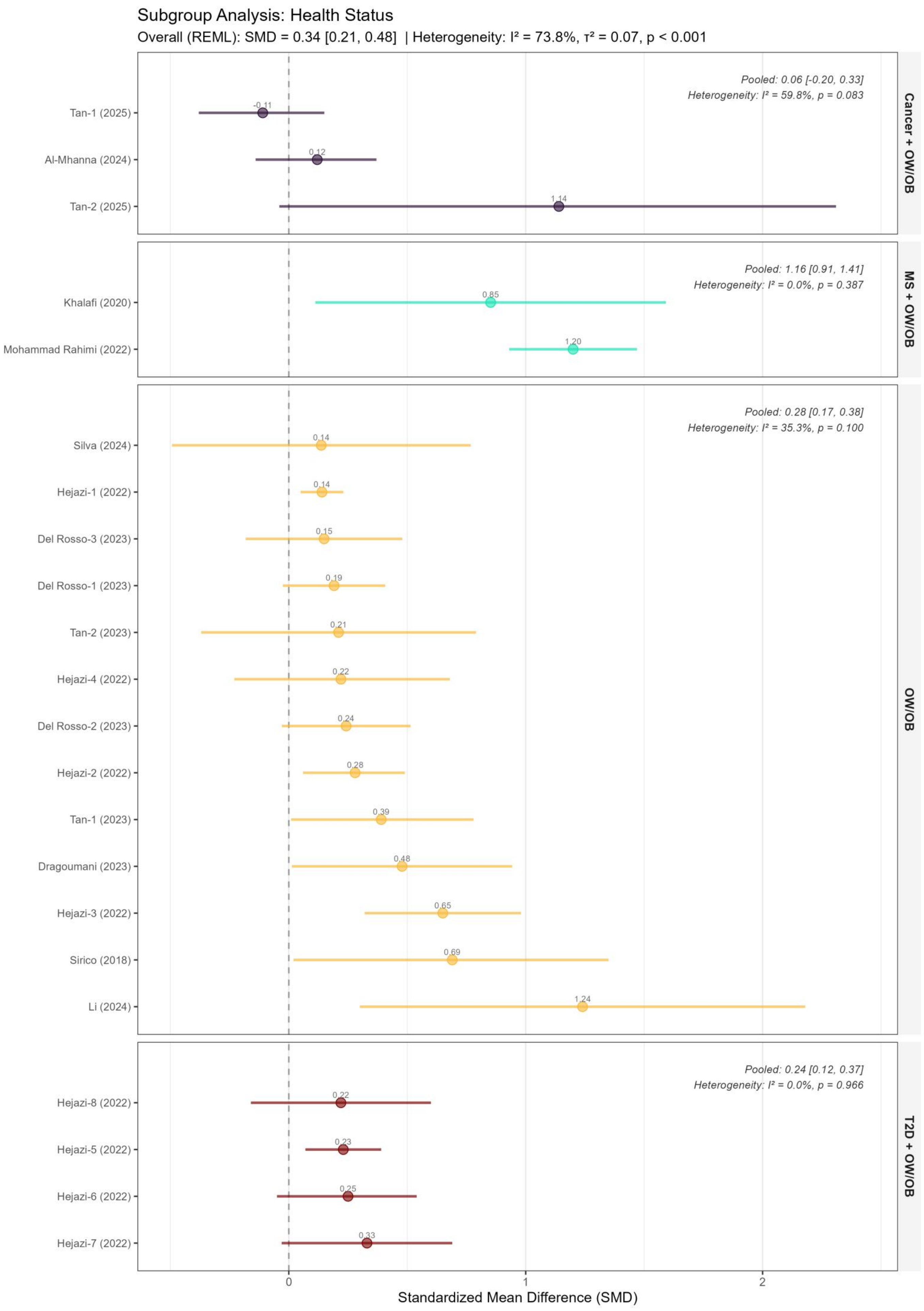


**Supplementary Figure S26. Results of Subgroup Analysis for Adiponectin by Health Status**

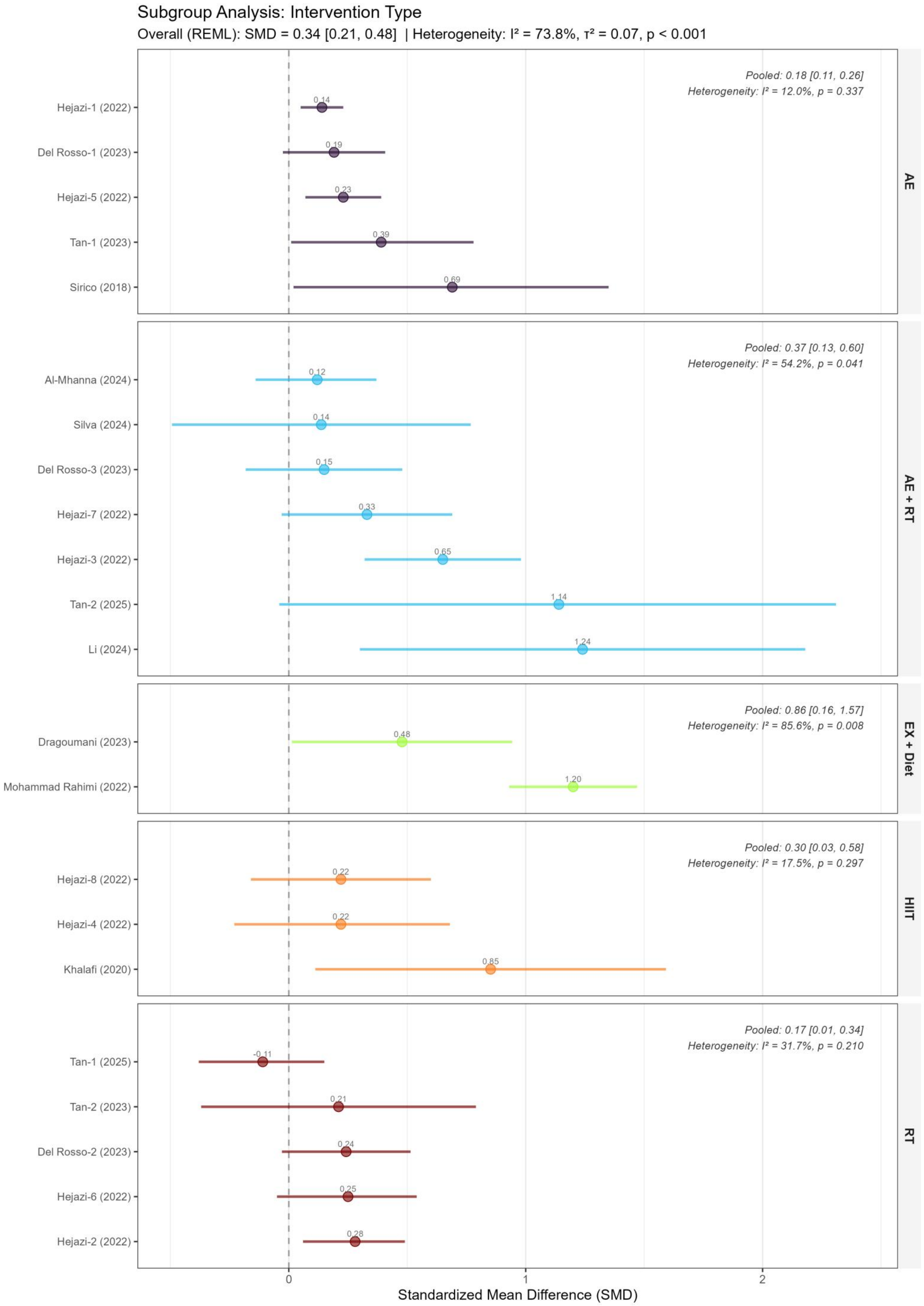


**Supplementary Figure S27. Results of Subgroup Analysis for Adiponectin by Intervention Type**

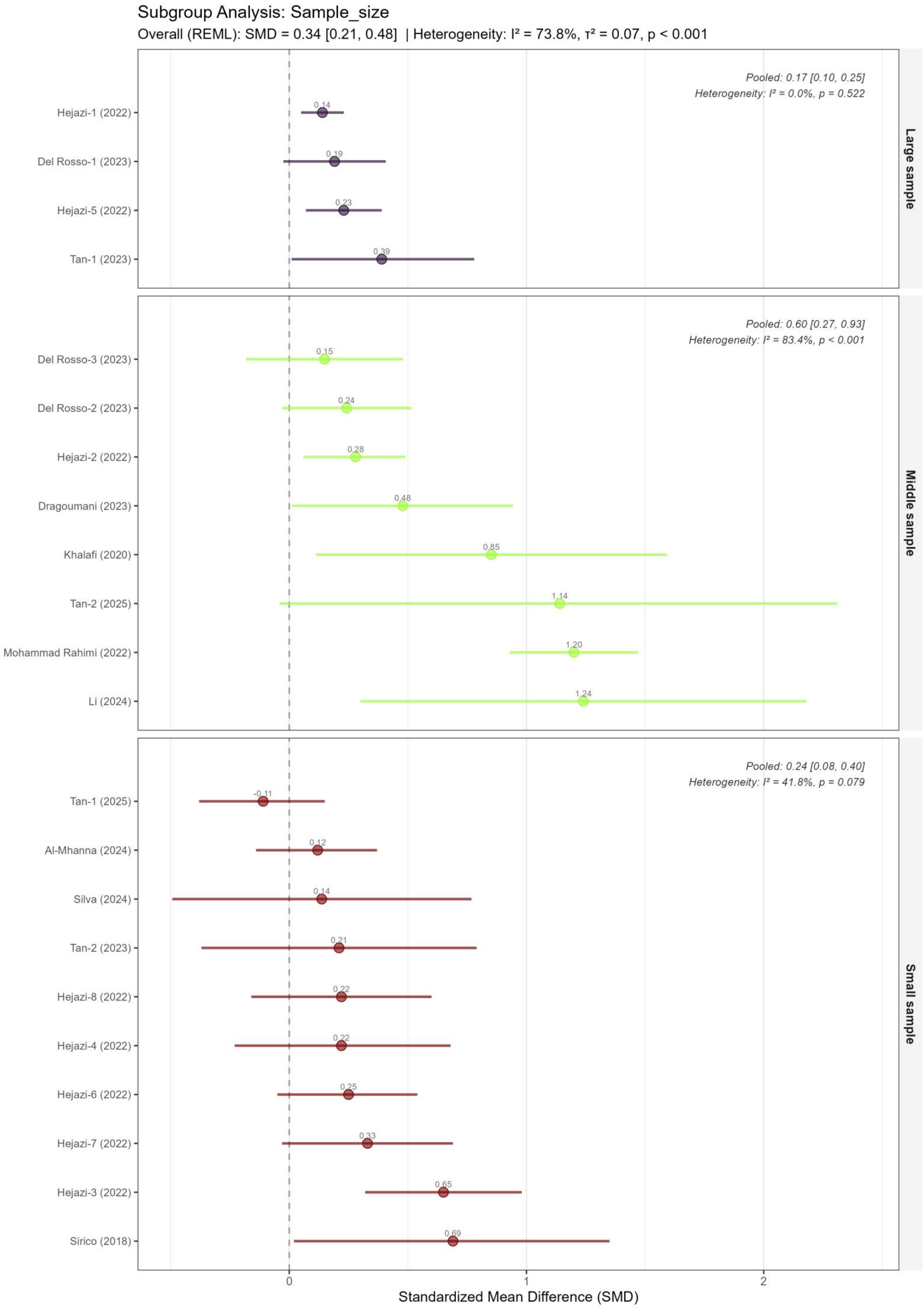


**Supplementary Figure S28. Results of Subgroup Analysis for Adiponectin by Sample Size**

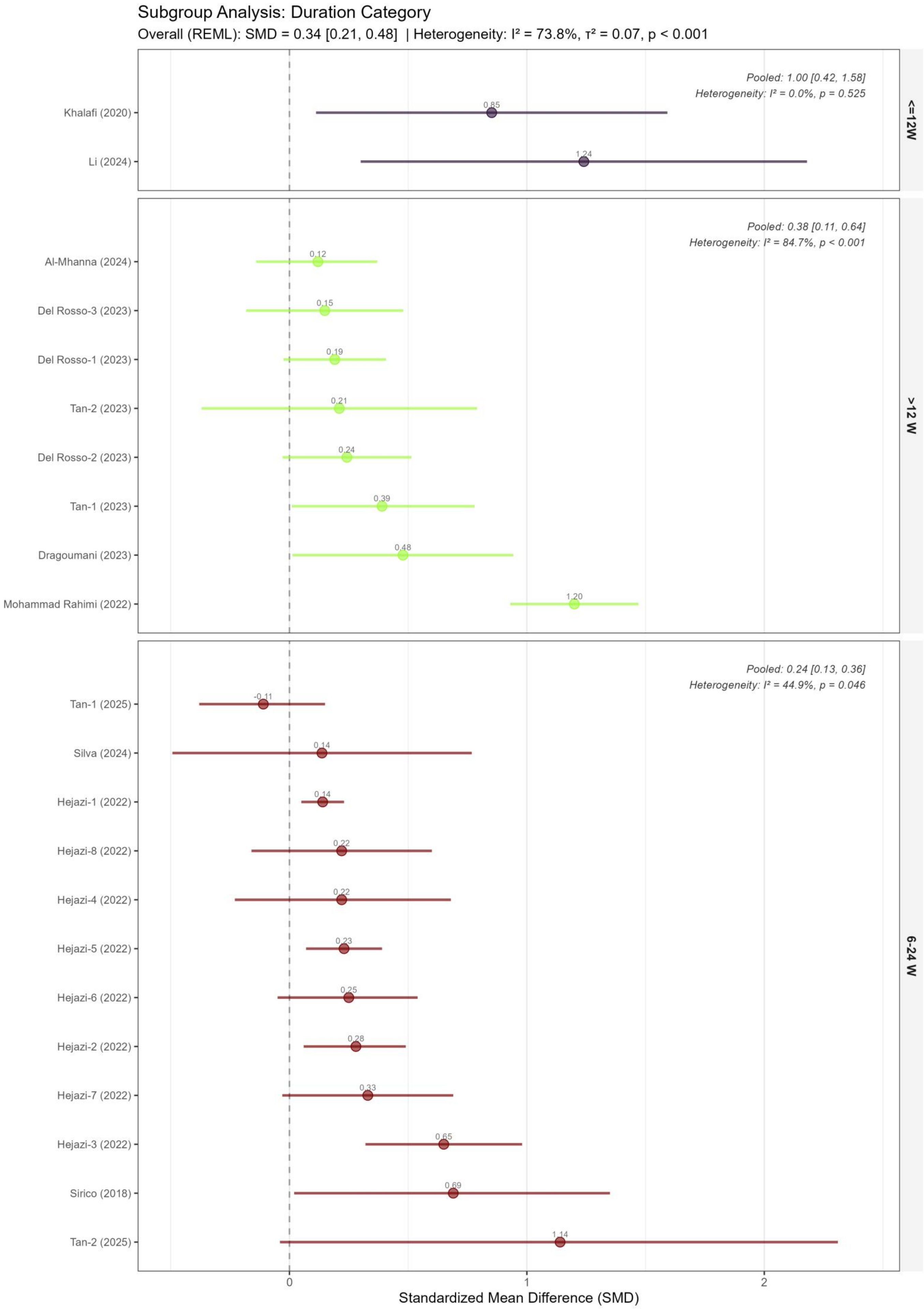


**Supplementary Figure S29. Results of Subgroup Analysis for Adiponectin by Intervention Duration**

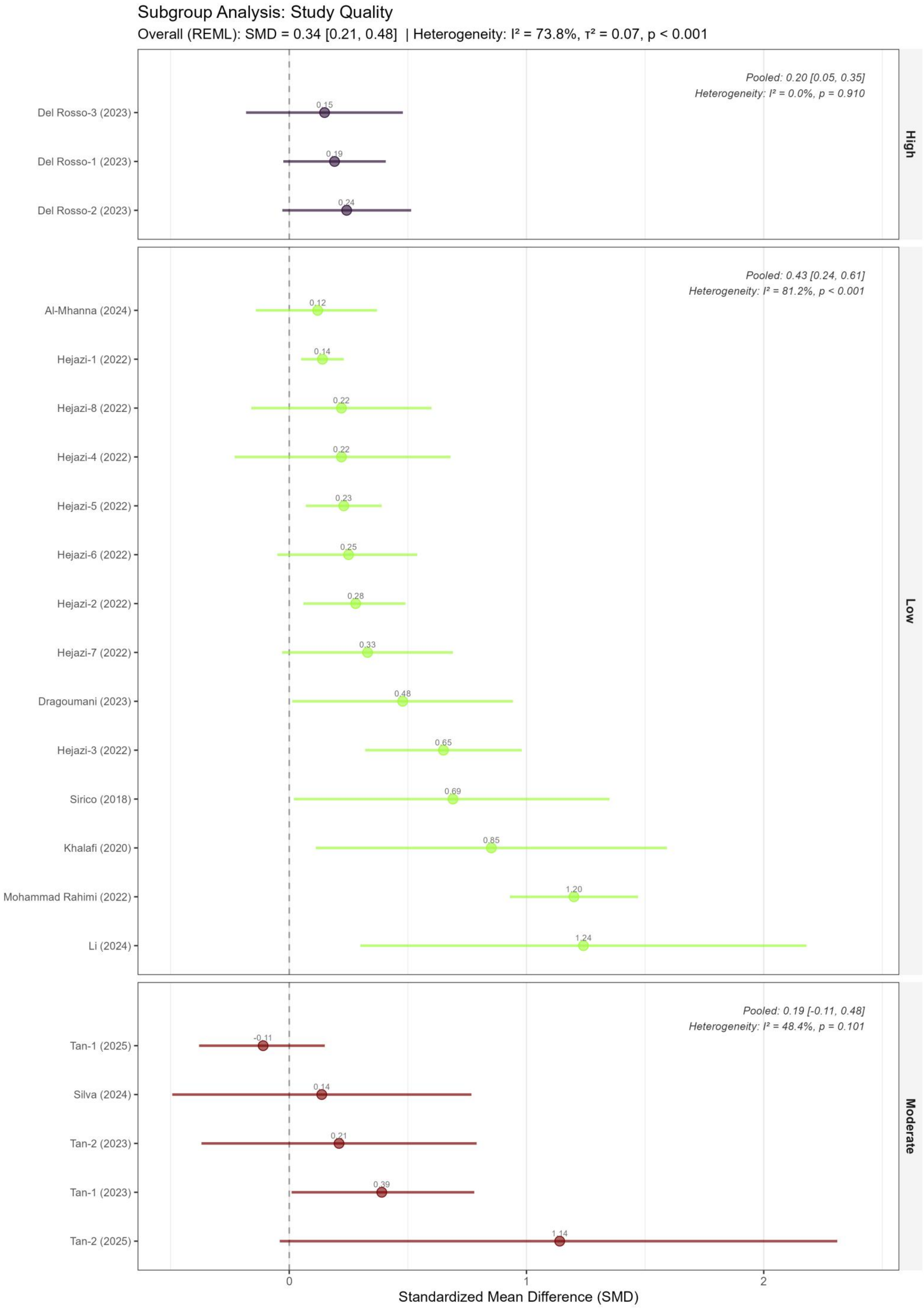


**Supplementary Figure S30. Results of Subgroup Analysis for Adiponectin by Study Quality**

### 6.3 For Leptin

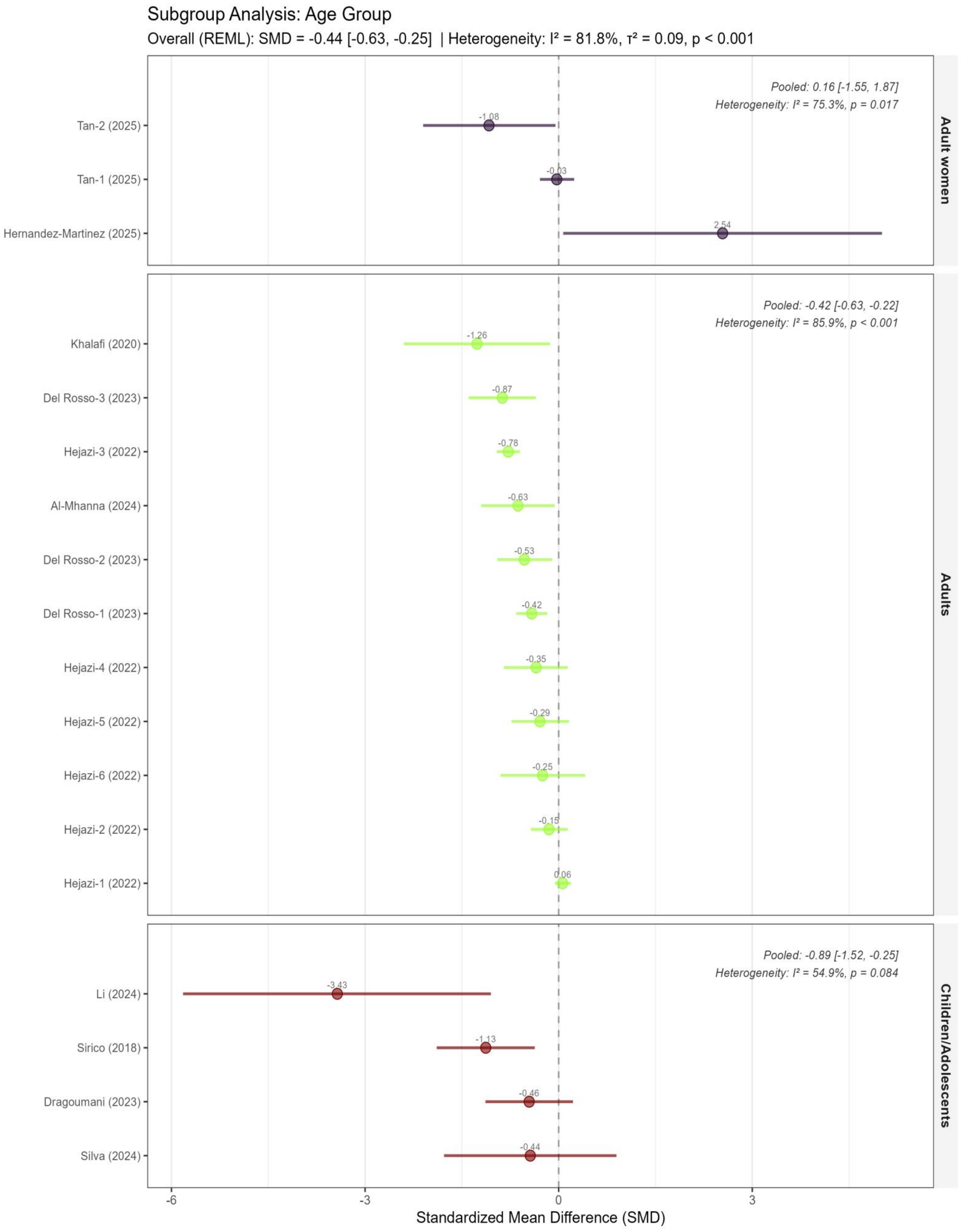


**Supplementary Figure S31. Results of Subgroup Analysis for Leptin by Age Group**

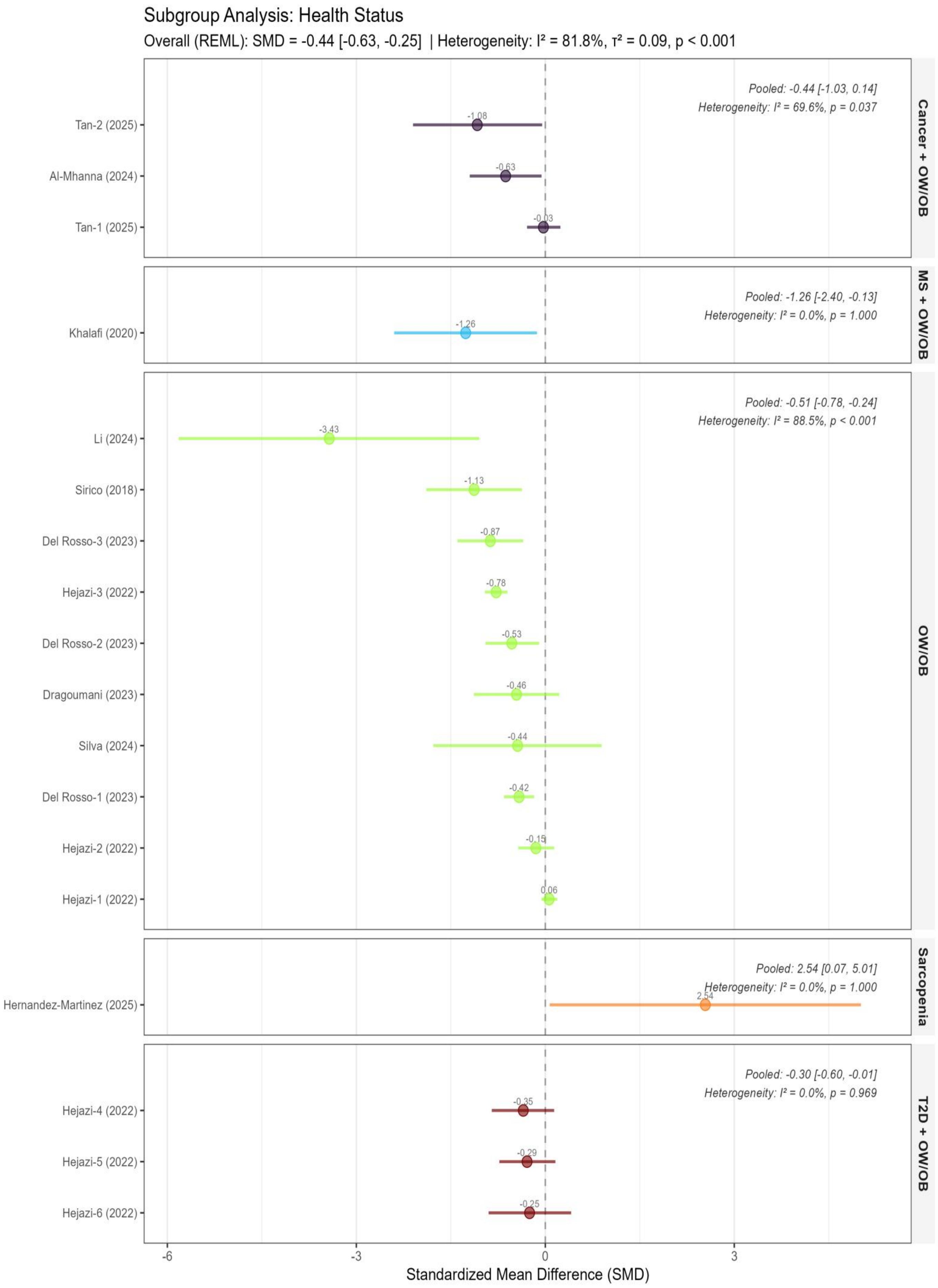


**Supplementary Figure S32. Results of Subgroup Analysis for Leptin by Health Status**

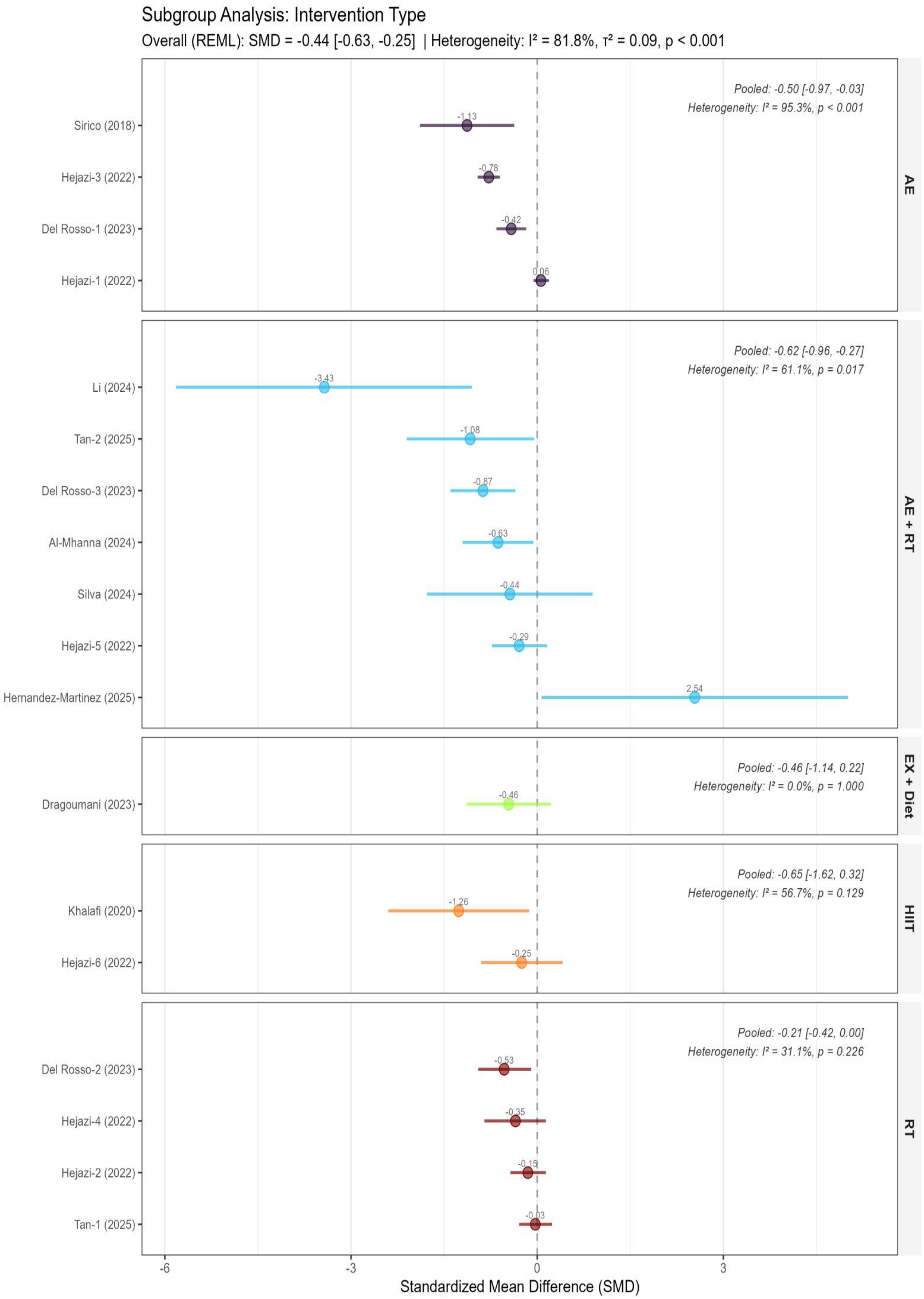


**Supplementary Figure S33. Results of Subgroup Analysis for Leptin by Intervention Type**

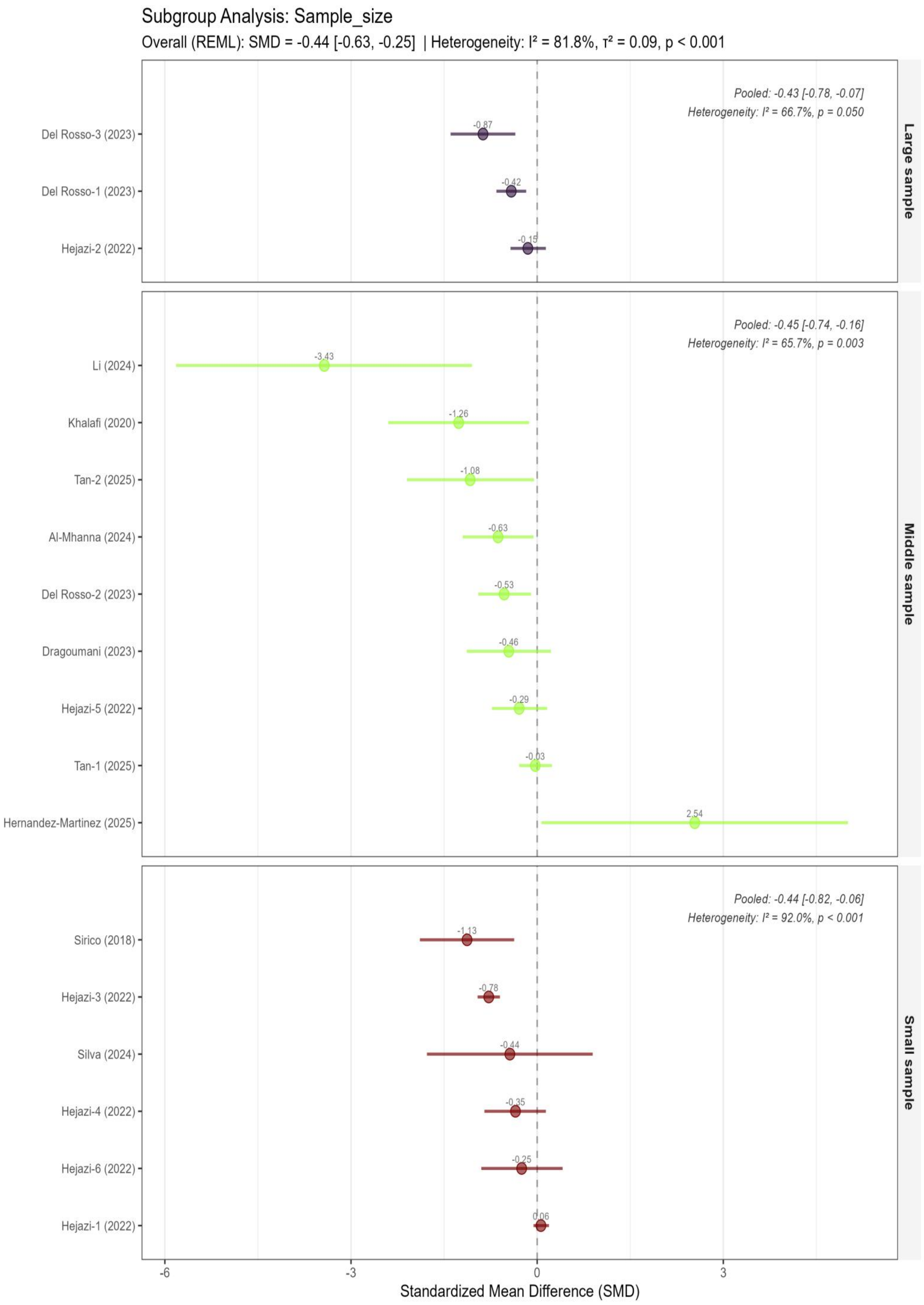


**Supplementary Figure S34. Results of Subgroup Analysis for Leptin by Sample Size**

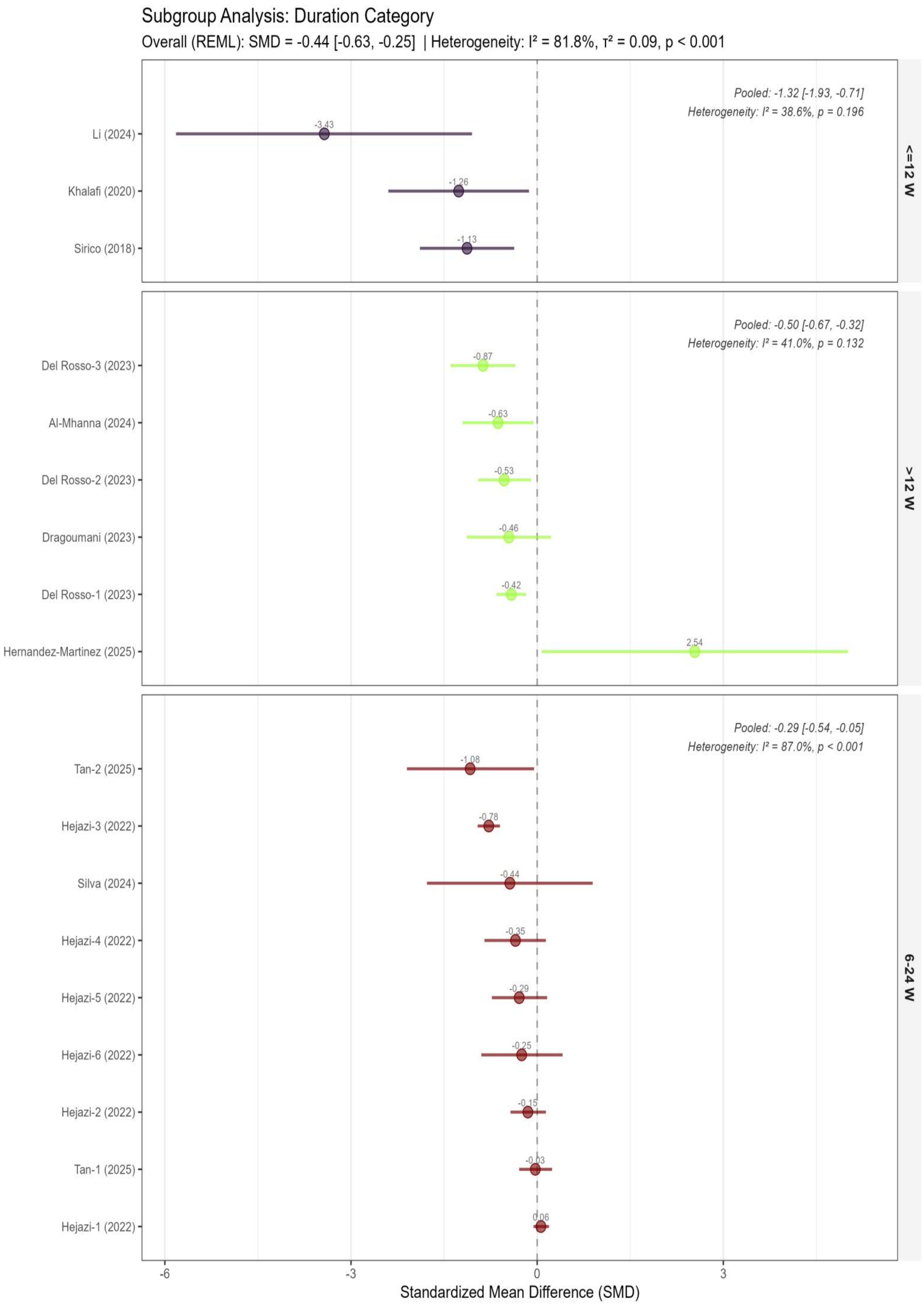


**Supplementary Figure S35. Results of Subgroup Analysis for Leptin by Intervention Duration**

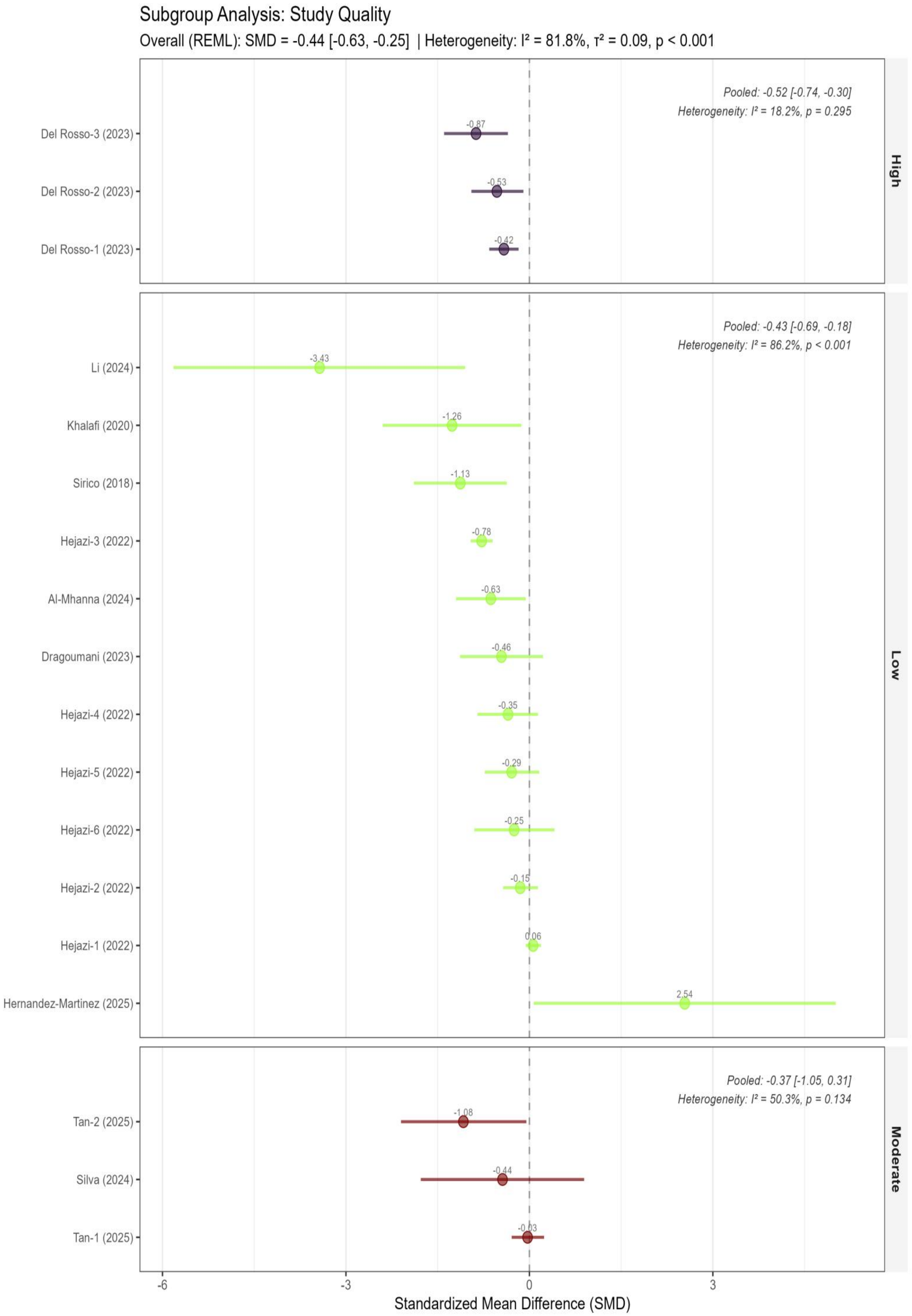


**Supplementary Figure S36. Results of Subgroup Analysis for Leptin by Study Quality**

## 6.4 For TNF-α

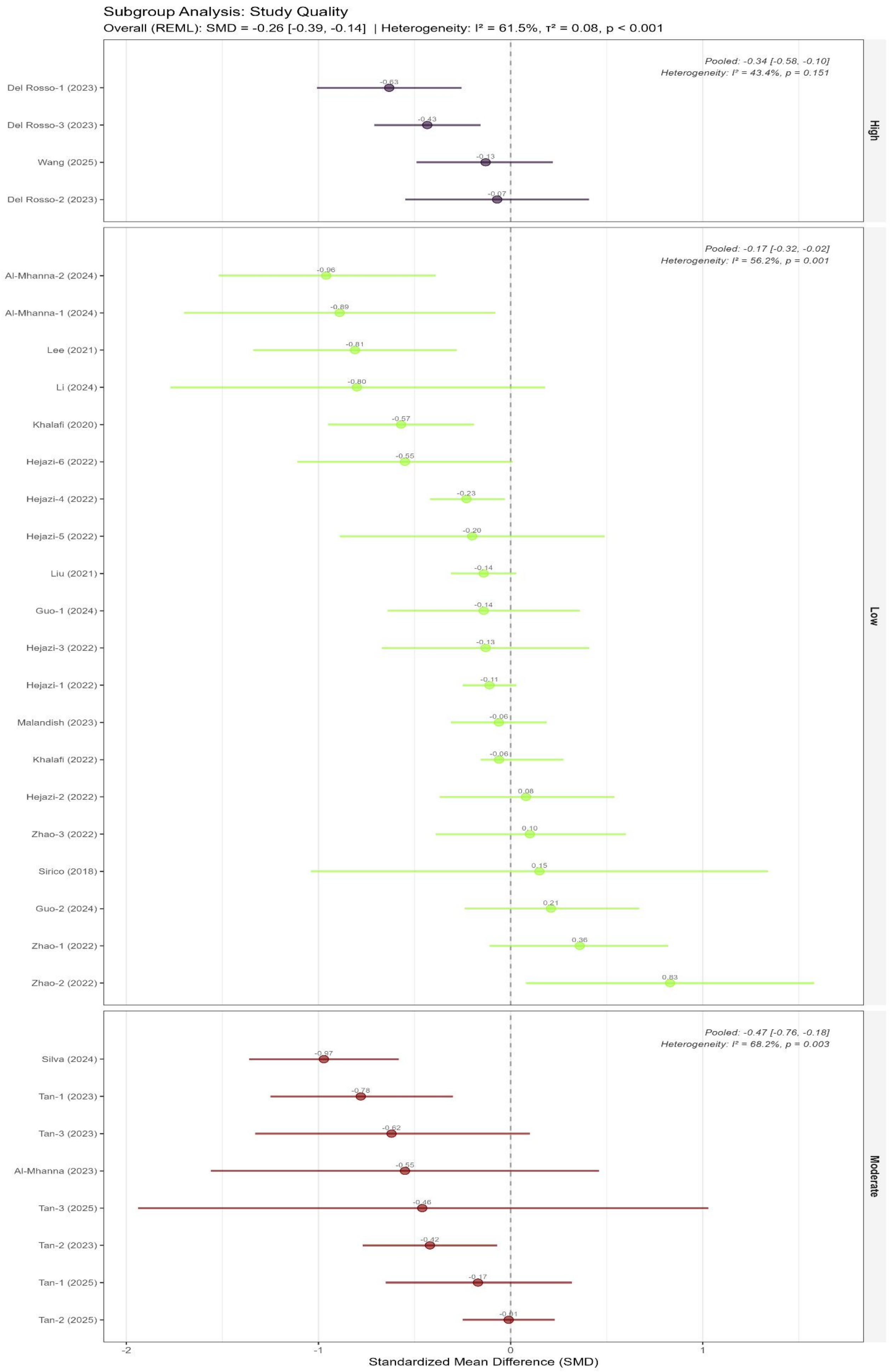


**Supplementary Figure S37. Results of Subgroup Analysis for TNF-α by Age Group**

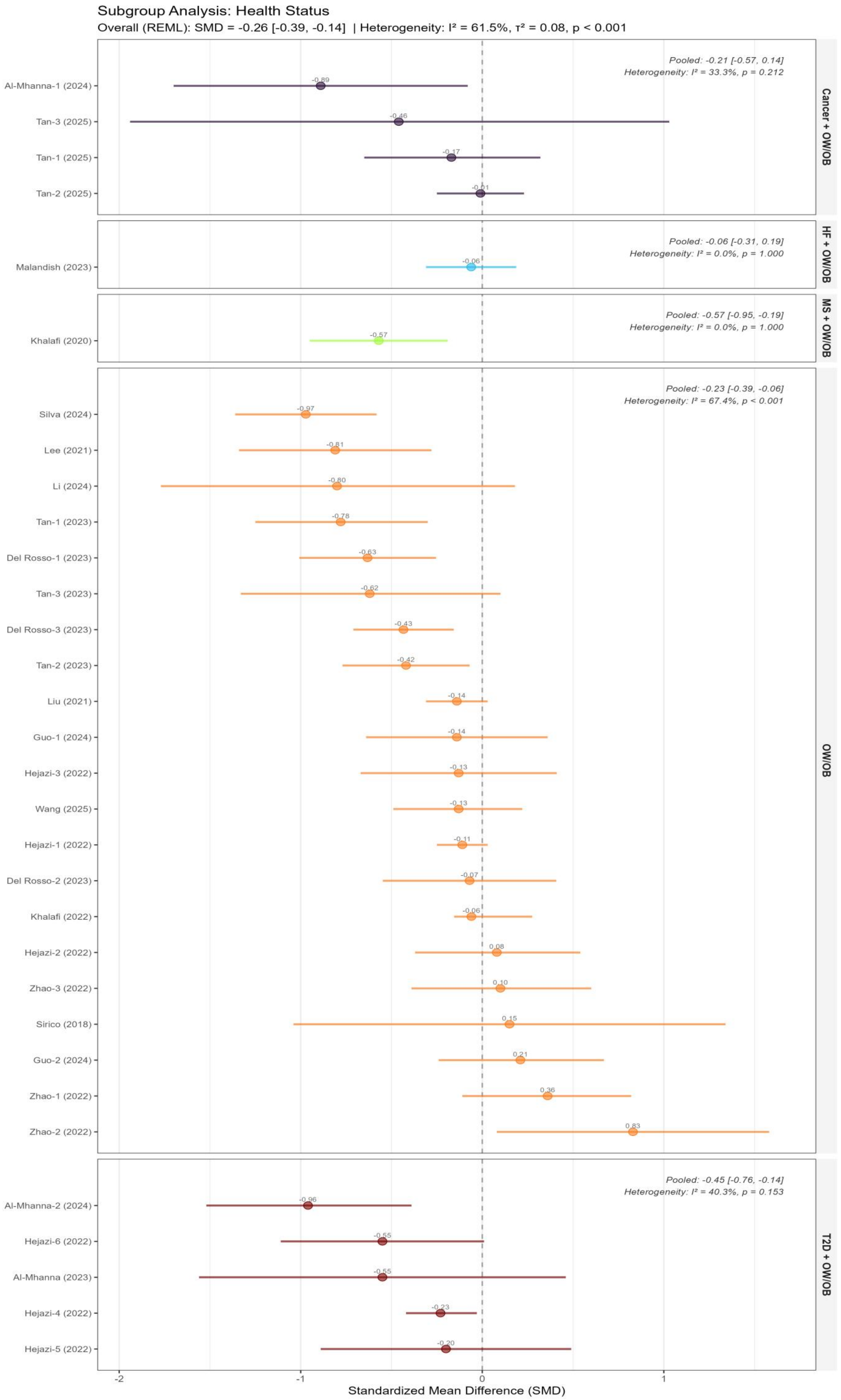


**Supplementary Figure S38. Results of Subgroup Analysis for TNF-α by Health Status**

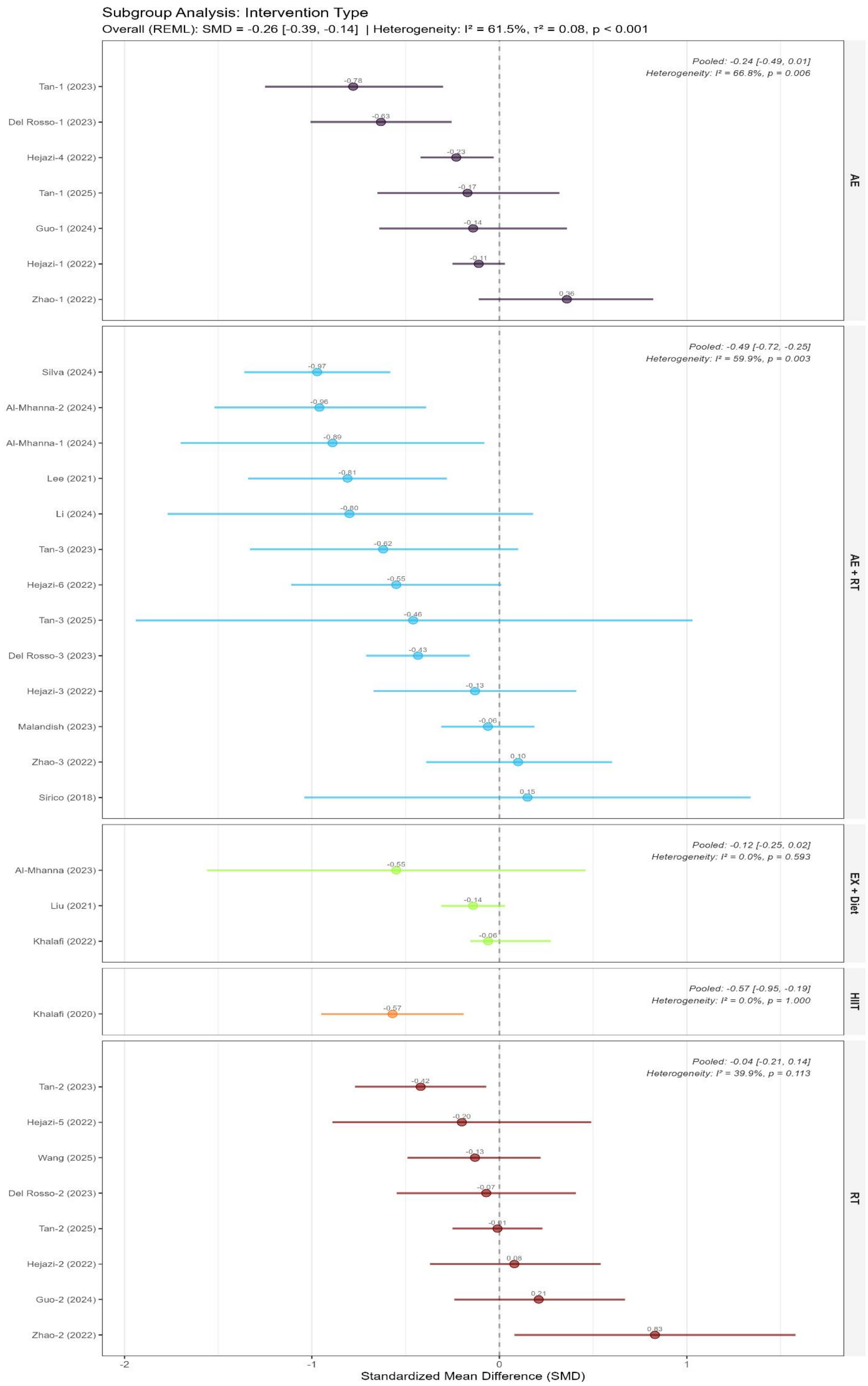


**Supplementary Figure S39. Results of Subgroup Analysis for TNF-α by Intervention Type**

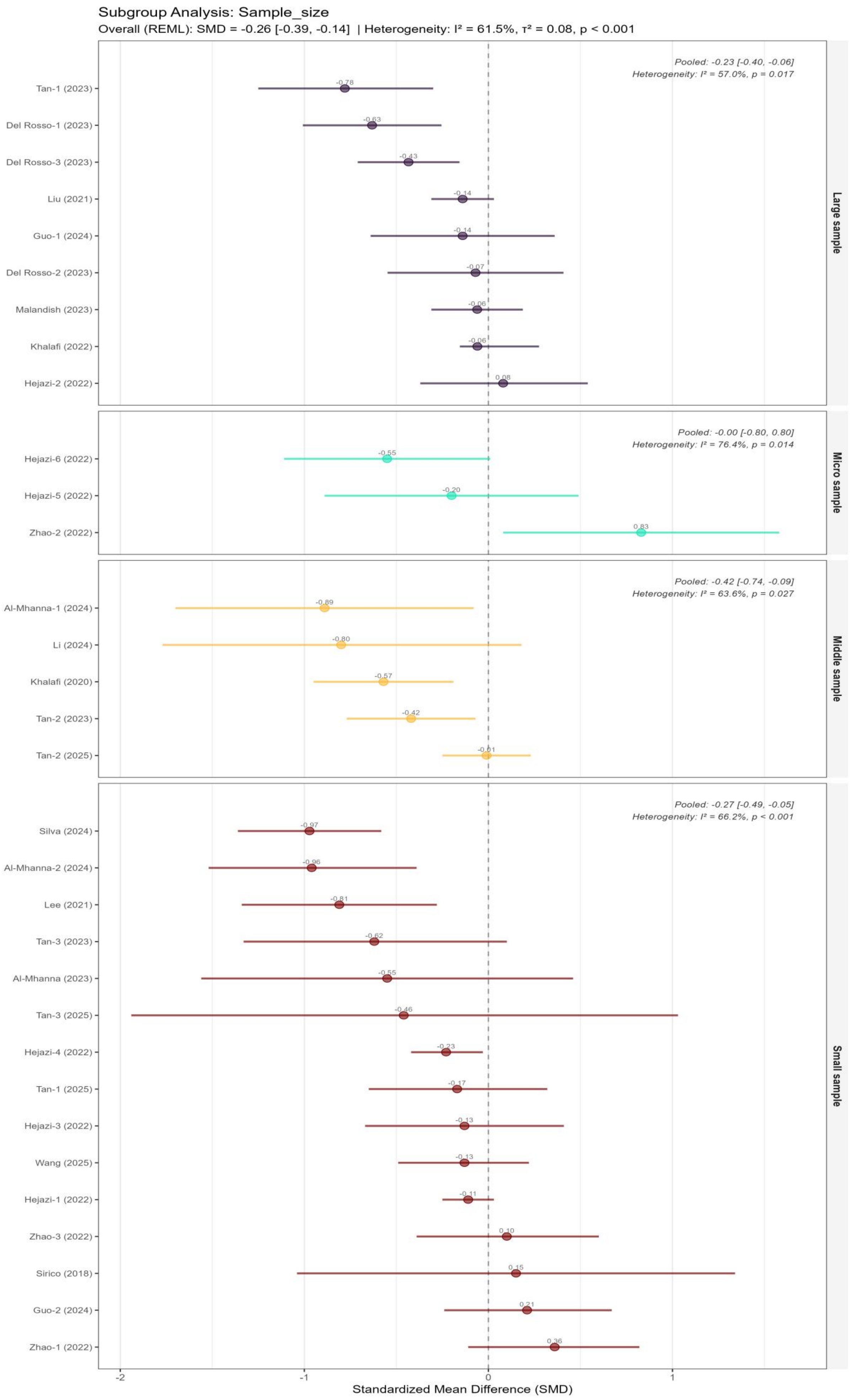


**Supplementary Figure S40. Results of Subgroup Analysis for TNF-α by Sample Size**

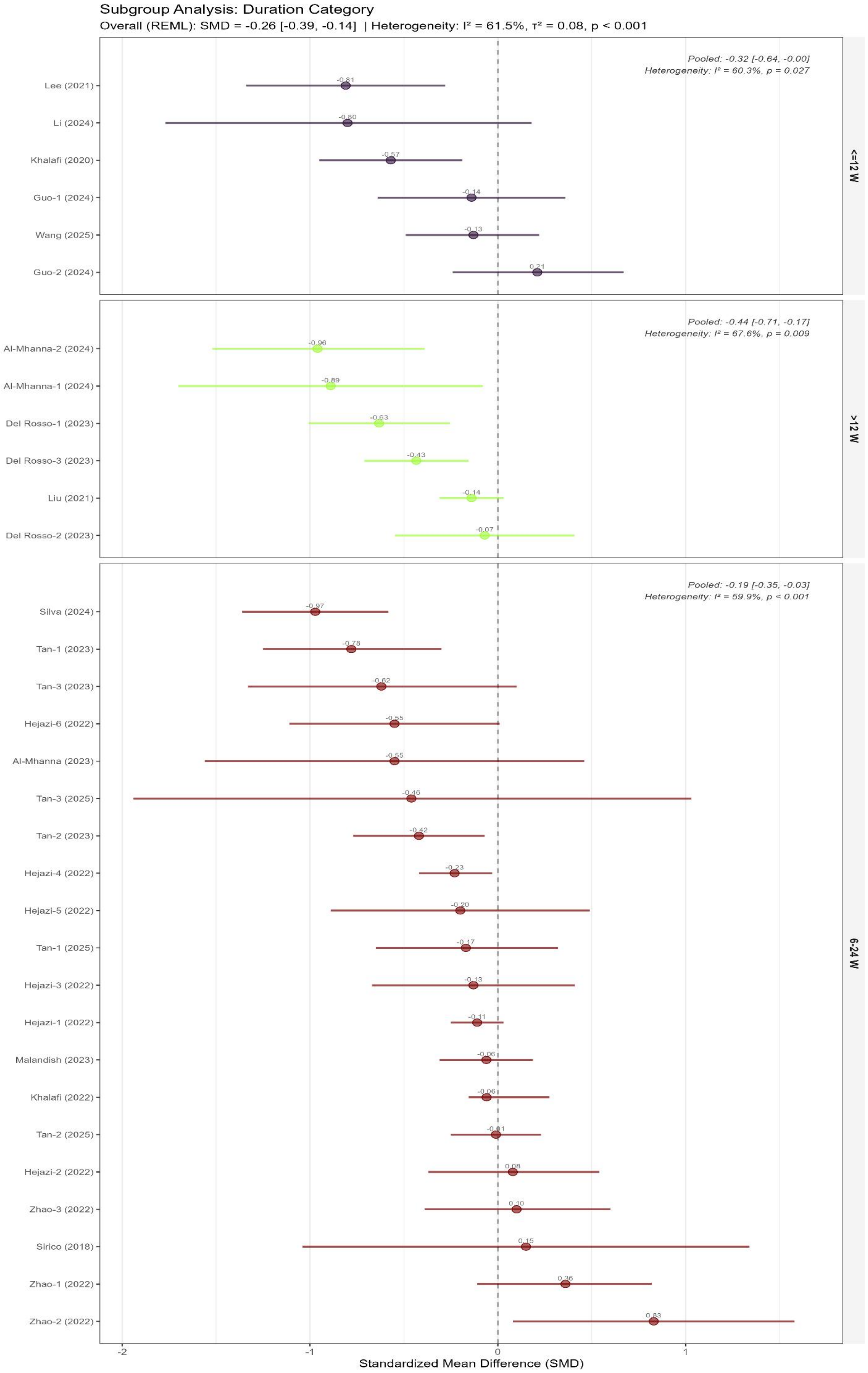


**Supplementary Figure S41. Results of Subgroup Analysis for TNF-α by Intervention Duration**

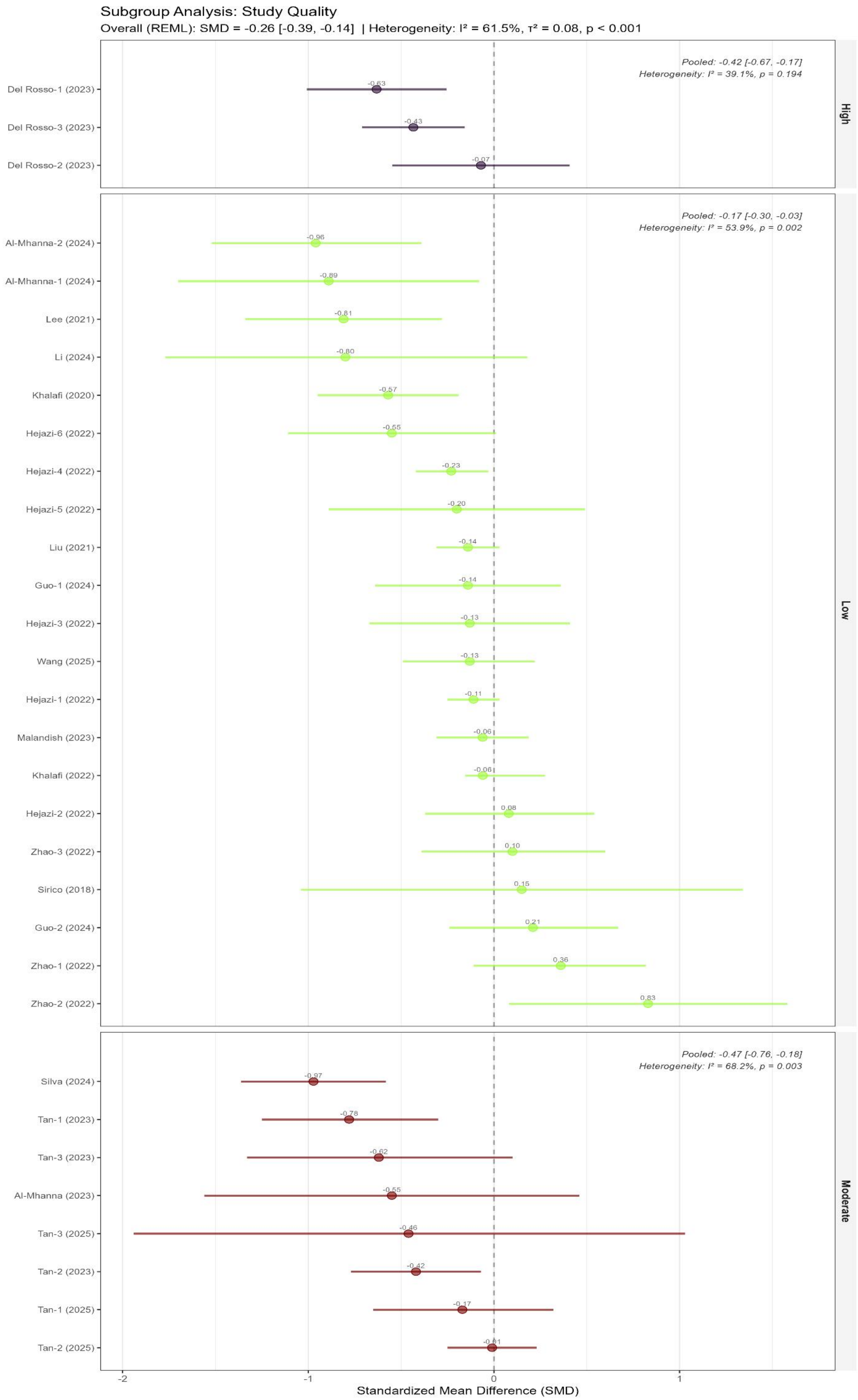


**Supplementary Figure S42. Results of Subgroup Analysis for TNF-α by Study Quality**

### 6.5 For IL-6

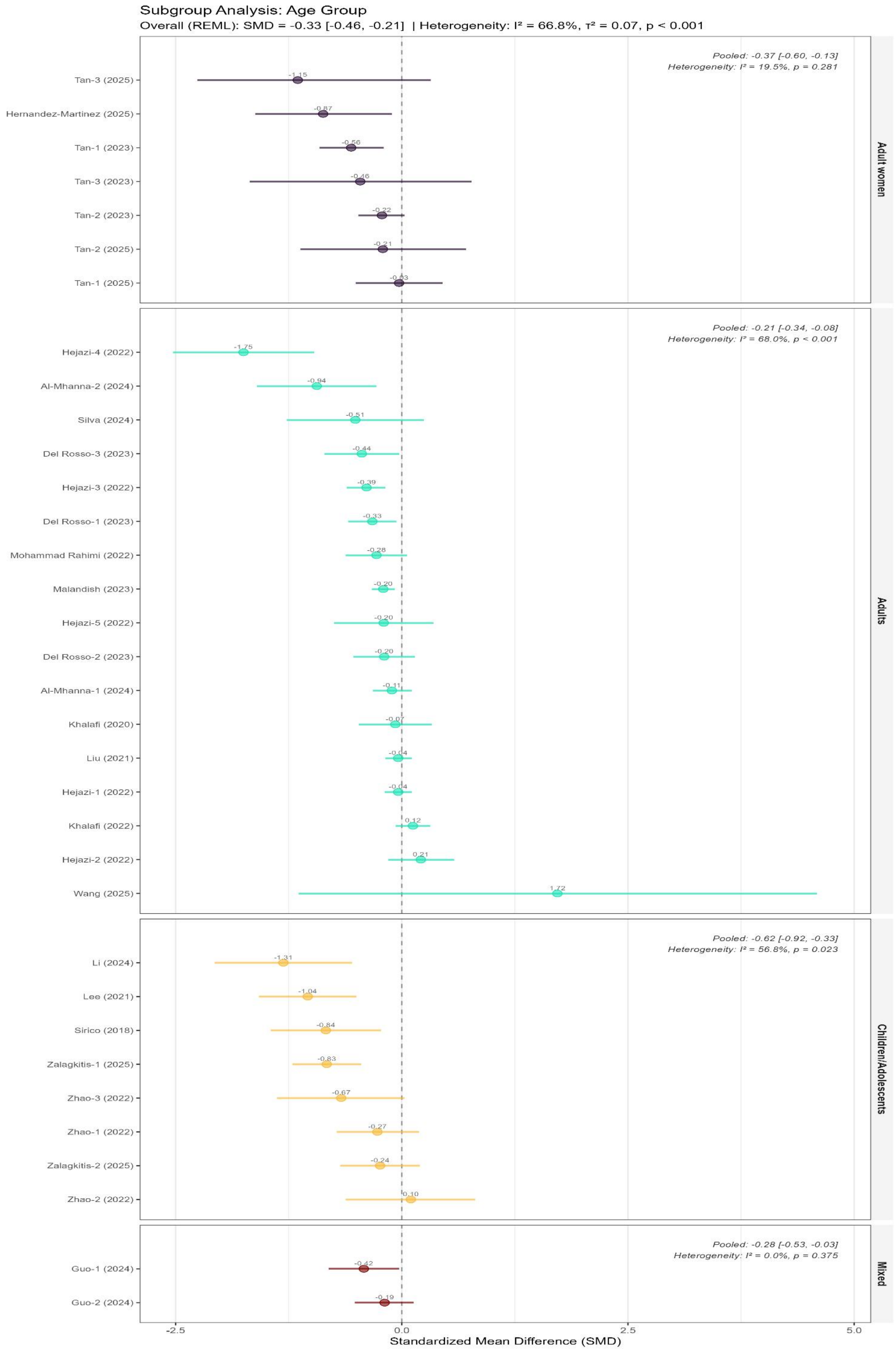


**Supplementary Figure S43. Results of Subgroup Analysis for IL-6 by Age Group**

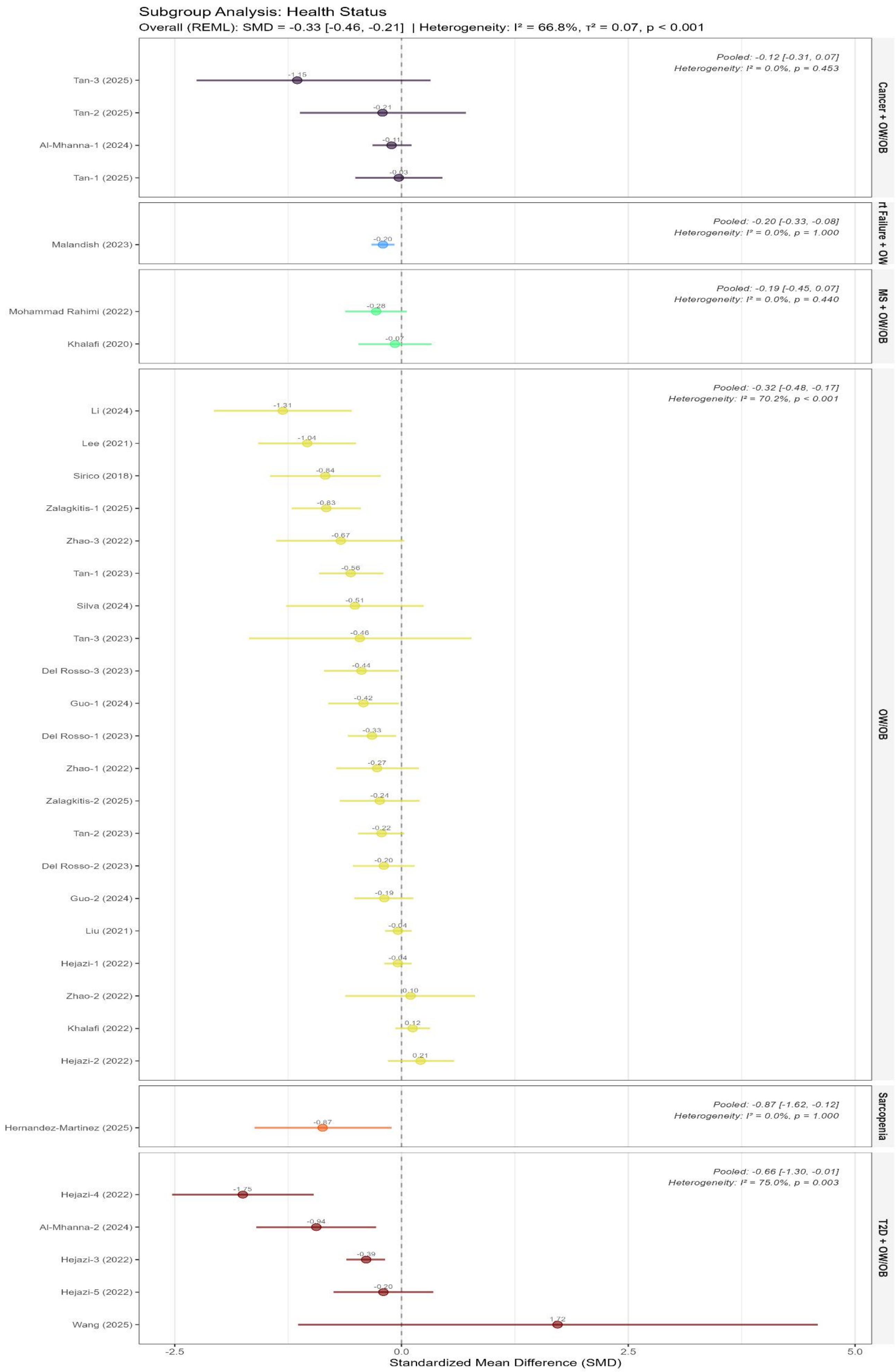


**Supplementary Figure S44. Results of Subgroup Analysis for IL-6 by Health Status**

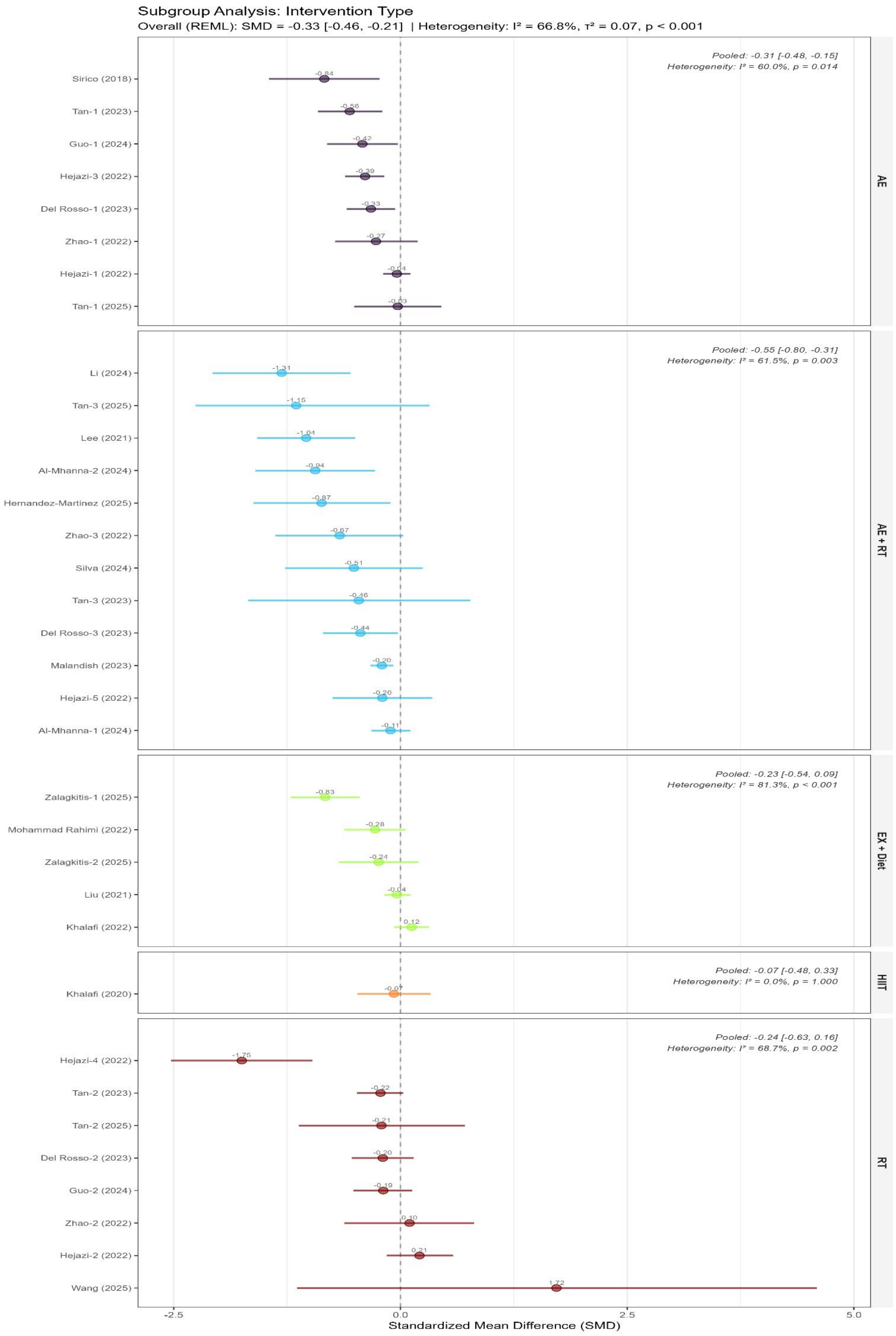


**Supplementary Figure S45. Results of Subgroup Analysis for IL-6 by Intervention Type**

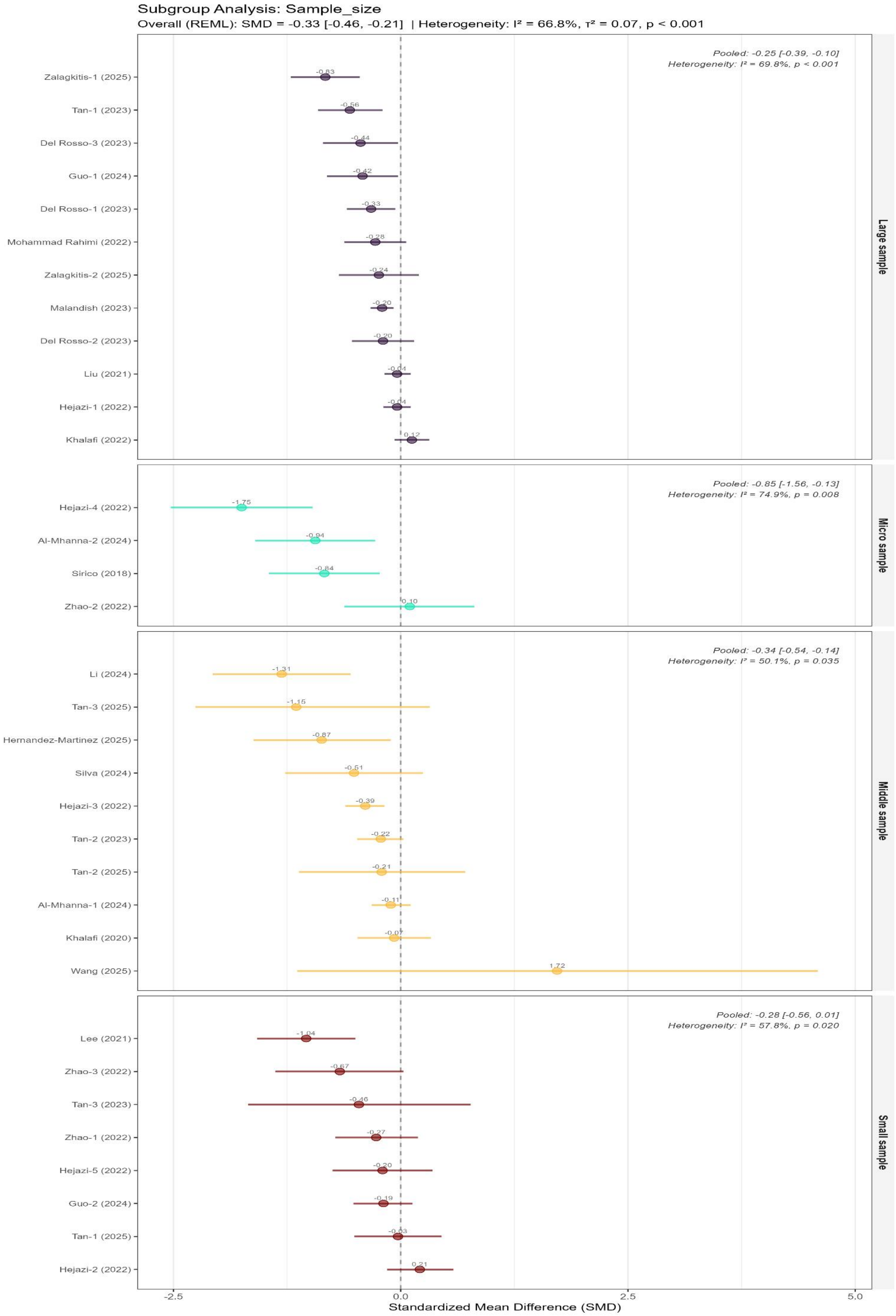


**Supplementary Figure S46. Results of Subgroup Analysis for IL-6 by Sample Size**

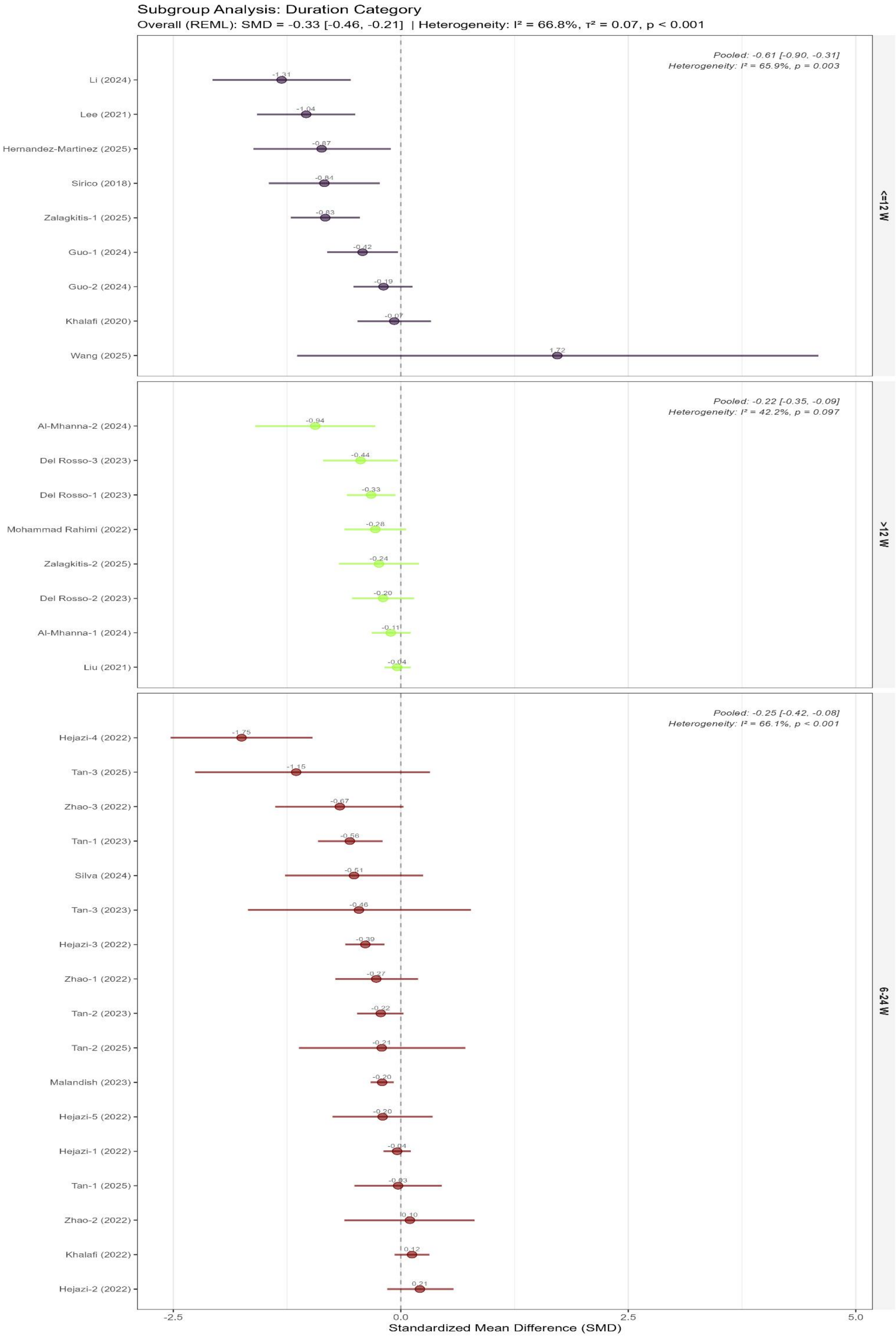


**Supplementary Figure S47. Results of Subgroup Analysis for IL-6 by Intervention Duration**

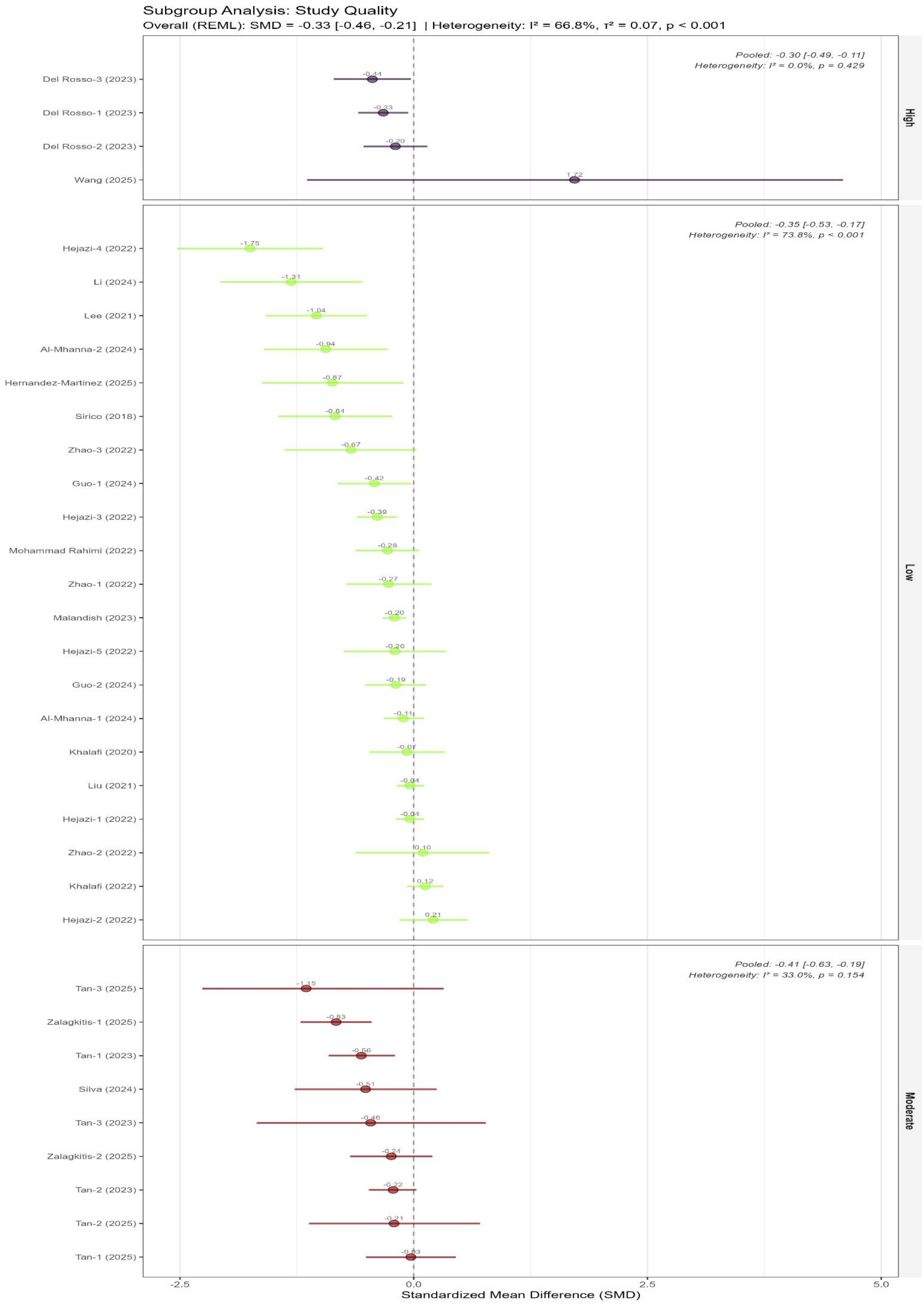


**Supplementary Figure S48. Results of Subgroup Analysis for IL-6 by Study Quality**

# 7 Supplementary Results: Corrected Covered Area (CCA) Overlap Analysis by Outcome

## 7.1 SFor CRP

Supplementary Table S9. Corrected Covered Area Overlap Analysis for CRP

| Primary study | Zalagkitis 1 2025 | Zalagkitis 2 2025 | Wang 2025 | Hernandez-Martinez 2025 | Tan 1 2025 | Tan 2 2025 | Tan 3 2025 | Silva 2024 | Li 2024 | Al-Mhanna-1 2024 | Al-Mhanna-2 2024 | Guo 1 2024 | Guo 2 2024 | Malandish 2023 | Dragoumani 2023 | Tan-1 2023 | Tan-2 2023 | Del Rosso -1 | Del Rosso -2 | Del Rosso -3 | Rahimi 2022 | Zhao-1 2022 | Zhao-2 2022 | Zhao-3 2022 | Zhao-4 2022 | Hejazi-1 2022 | Hejazi-2 2022 | Hejazi-3 2022 | Hejazi-4 2022 | Hejazi-5 2022 | Hejazi-6 2022 | Khalafi 2022 | Liu 2021 | Lee 1 2021 | Lee 2 2021 | Khalafi 2020 | Sirico 2018 | Garcia-Hermoso 2016 |
|---|---|---|---|---|---|---|---|---|---|---|---|---|---|---|---|---|---|---|---|---|---|---|---|---|---|---|---|---|---|---|---|---|---|---|---|---|---|---|
| Abassi 2022[12] | 0 | 0 | 0 | 0 | 0 | 0 | 0 | 0 | 1 | 0 | 0 | 0 | 0 | 0 | 0 | 0 | 0 | 0 | 0 | 0 | 0 | 0 | 0 | 0 | 0 | 0 | 0 | 0 | 0 | 0 | 0 | 0 | 0 | 0 | 0 | 0 | 0 | 0 |
| Abd El-Kader 2016[13] | 0 | 0 | 0 | 0 | 0 | 0 | 0 | 0 | 0 | 0 | 0 | 0 | 0 | 0 | 0 | 1 | 0 | 0 | 0 | 0 | 0 | 0 | 0 | 0 | 0 | 0 | 0 | 0 | 0 | 0 | 0 | 0 | 0 | 0 | 0 | 0 | 0 | 0 |
| Abolahrari-Shirazi 2018[14] | 0 | 0 | 0 | 0 | 0 | 0 | 0 | 0 | 0 | 0 | 0 | 0 | 0 | 1 | 0 | 0 | 0 | 0 | 0 | 0 | 0 | 0 | 0 | 0 | 0 | 0 | 0 | 0 | 0 | 0 | 0 | 0 | 0 | 0 | 0 | 0 | 0 | 0 |
| Abu-Kishk 2014[15] | 0 | 1 | 0 | 0 | 0 | 0 | 0 | 0 | 0 | 0 | 0 | 0 | 0 | 0 | 0 | 0 | 0 | 0 | 0 | 0 | 0 | 0 | 0 | 0 | 0 | 0 | 0 | 0 | 0 | 0 | 0 | 0 | 0 | 0 | 0 | 0 | 0 | 0 |
| Adamopoulos 2014[16] | 0 | 0 | 0 | 0 | 0 | 0 | 0 | 0 | 0 | 0 | 0 | 0 | 0 | 1 | 0 | 0 | 0 | 0 | 0 | 0 | 0 | 0 | 0 | 0 | 0 | 0 | 0 | 0 | 0 | 0 | 0 | 0 | 0 | 0 | 0 | 0 | 0 | 0 |
| Ahmad 2014[17] | 0 | 0 | 0 | 0 | 0 | 0 | 0 | 0 | 0 | 0 | 0 | 0 | 0 | 1 | 0 | 0 | 0 | 0 | 0 | 0 | 0 | 0 | 0 | 0 | 0 | 0 | 0 | 0 | 0 | 0 | 0 | 0 | 0 | 0 | 0 | 0 | 0 | 0 |
| Akbarpour 2013[18] | 0 | 0 | 0 | 0 | 0 | 0 | 0 | 0 | 0 | 0 | 0 | 0 | 0 | 0 | 0 | 0 | 0 | 1 | 0 | 0 | 0 | 0 | 0 | 0 | 0 | 2 | 0 | 0 | 0 | 0 | 0 | 0 | 0 | 0 | 0 | 0 | 0 | 0 |
| Aksoy 2015[19] | 0 | 0 | 0 | 0 | 0 | 0 | 0 | 0 | 0 | 0 | 0 | 0 | 0 | 1 | 0 | 0 | 0 | 0 | 0 | 0 | 0 | 0 | 0 | 0 | 0 | 0 | 0 | 0 | 0 | 0 | 0 | 0 | 0 | 0 | 0 | 0 | 0 | 0 |
| Alberga 2015[20] | 0 | 0 | 0 | 0 | 0 | 0 | 0 | 0 | 0 | 0 | 0 | 0 | 0 | 0 | 0 | 0 | 0 | 0 | 0 | 0 | 0 | 1 | 2 | 3 | 0 | 0 | 0 | 0 | 0 | 0 | 0 | 0 | 0 | 4 | 5 | 0 | 0 | 6 |
| Allen 2017[21] | 0 | 0 | 0 | 0 | 0 | 0 | 0 | 0 | 0 | 0 | 0 | 0 | 0 | 0 | 0 | 0 | 0 | 0 | 0 | 0 | 0 | 0 | 0 | 0 | 0 | 0 | 0 | 0 | 0 | 0 | 0 | 0 | 0 | 0 | 0 | 1 | 0 | 0 |
| Almenning 2015[22] | 0 | 0 | 0 | 0 | 0 | 0 | 0 | 0 | 0 | 0 | 0 | 0 | 0 | 0 | 0 | 0 | 0 | 0 | 0 | 0 | 0 | 0 | 0 | 0 | 0 | 0 | 0 | 0 | 0 | 0 | 0 | 0 | 0 | 0 | 0 | 1 | 0 | 0 |
| Amaro-Gahete 2021[23] | 0 | 0 | 0 | 0 | 0 | 0 | 0 | 1 | 0 | 0 | 0 | 0 | 0 | 0 | 0 | 0 | 0 | 0 | 0 | 0 | 0 | 0 | 0 | 0 | 0 | 0 | 0 | 0 | 0 | 0 | 0 | 0 | 0 | 0 | 0 | 0 | 0 | 0 |
| Annibalini 2017[24] | 0 | 0 | 0 | 0 | 0 | 0 | 0 | 0 | 0 | 0 | 1 | 0 | 0 | 0 | 0 | 0 | 0 | 0 | 0 | 2 | 0 | 0 | 0 | 0 | 0 | 0 | 0 | 0 | 0 | 0 | 3 | 0 | 0 | 0 | 0 | 0 | 0 | 0 |
| Arikawa 2011[25] | 0 | 0 | 0 | 0 | 0 | 0 | 0 | 0 | 0 | 0 | 0 | 0 | 0 | 0 | 0 | 0 | 0 | 0 | 0 | 0 | 0 | 0 | 0 | 0 | 0 | 1 | 0 | 0 | 0 | 0 | 0 | 0 | 0 | 0 | 0 | 0 | 0 | 0 |
| Arsenault 2009[26] | 0 | 0 | 0 | 0 | 0 | 0 | 0 | 0 | 0 | 0 | 0 | 1 | 0 | 0 | 0 | 2 | 0 | 3 | 0 | 0 | 0 | 0 | 0 | 0 | 0 | 4 | 0 | 0 | 0 | 0 | 0 | 0 | 0 | 0 | 0 | 0 | 0 | 0 |
| Auerbach 2013[27] | 0 | 0 | 0 | 0 | 0 | 0 | 0 | 0 | 0 | 0 | 0 | 0 | 0 | 0 | 0 | 0 | 0 | 0 | 0 | 0 | 0 | 0 | 0 | 0 | 0 | 0 | 0 | 0 | 0 | 0 | 0 | 1 | 0 | 0 | 0 | 0 | 0 | 0 |
| Bagheri 2020[28] | 0 | 0 | 0 | 0 | 0 | 0 | 0 | 0 | 0 | 0 | 0 | 0 | 0 | 0 | 0 | 0 |  | 0 | 0 | 0 | 0 | 0 | 0 | 0 | 0 | 1 | 0 | 0 | 0 | 0 | 0 | 0 | 0 | 0 | 0 | 0 | 0 | 0 |
| Bahmanbeglou 2019[29] | 0 | 0 | 0 | 0 | 0 | 0 | 0 | 0 | 0 | 0 | 0 | 0 | 0 | 0 | 0 | 0 | 0 | 0 | 0 | 1 | 0 | 0 | 0 | 0 | 0 | 0 | 0 | 0 | 0 | 0 | 0 | 0 | 0 | 0 | 0 | 1 | 0 | 0 |
| Balagopal 2005[30] | 1 | 0 | 0 | 0 | 0 | 0 | 0 | 0 | 0 | 0 | 0 | 0 | 0 | 0 | 2 | 0 | 0 | 0 | 0 | 0 | 0 | 0 | 0 | 0 | 0 | 0 | 0 | 0 | 0 | 0 | 0 | 0 | 0 | 0 | 0 | 0 | 0 | 0 |
| Balducci 2010[31] | 0 | 0 | 0 | 0 | 0 | 0 | 0 | 0 | 0 | 0 | 0 | 0 | 0 | 0 | 0 | 0 | 0 | 0 | 0 | 1 | 0 | 0 | 0 | 0 | 0 | 0 | 0 | 0 | 2 | 3 | 4 | 0 | 0 | 0 | 0 | 0 | 0 | 0 |
| Balducci 2017[32] | 0 | 0 | 0 | 0 | 0 | 0 | 0 | 0 | 0 | 0 | 0 | 0 | 0 | 0 | 0 | 0 | 0 | 1 | 0 | 0 | 0 | 0 | 0 | 0 | 0 | 0 | 0 | 0 | 0 | 0 | 0 | 0 | 0 | 0 | 0 | 0 | 0 | 0 |
| Balen 2008[33] | 0 | 0 | 0 | 0 | 0 | 0 | 0 | 0 | 0 | 0 | 0 | 0 | 0 | 1 | 0 | 0 | 0 | 0 | 0 | 0 | 0 | 0 | 0 | 0 | 0 | 0 | 0 | 0 | 0 | 0 | 0 | 0 | 0 | 0 | 0 | 0 | 0 | 0 |
| Ben Ounis 2010[34] | 0 | 1 | 0 | 0 | 0 | 0 | 0 | 0 | 0 | 0 | 0 | 0 | 0 | 0 | 0 | 0 | 0 | 0 | 0 | 0 | 0 | 0 | 0 | 0 | 0 | 0 | 0 | 0 | 0 | 0 | 0 | 0 | 0 | 2 | 3 | 0 | 0 | 0 |
| Bhati 2023[35] | 0 | 0 | 1 | 0 | 0 | 0 | 0 | 0 | 0 | 0 | 0 | 0 | 0 | 0 | 0 | 0 | 0 | 0 | 0 | 0 | 0 | 0 | 0 | 0 | 0 | 0 | 0 | 0 | 0 | 0 | 0 | 0 | 0 | 0 | 0 | 0 | 0 | 0 |
| Bo 2007[36] | 0 | 0 | 0 | 0 | 0 | 0 | 0 | 0 | 0 | 0 | 0 | 0 | 0 | 0 | 0 | 0 | 0 | 0 | 0 | 0 | 1 | 0 | 0 | 0 | 0 | 0 | 0 | 0 | 0 | 0 | 0 | 0 | 0 | 0 | 0 | 0 | 0 | 0 |

| Primary study | Zalagkitis 1 2025 | Zalagkitis 2 2025 | Wang 2025 | Hernandez-Martinez 2025 | Tan 1 2025 | Tan 2 2025 | Tan 3 2025 | Silva 2024 | Li 2024 | Al-Mhanna-1 2024 | Al-Mhanna-2 2024 | Guo 1 2024 | Guo 2 2024 | Malandish 2023 | Dragoumani 2023 | Tan-1 2023 | Tan-2 2023 | DelRosso-1 | DelRosso-2 | DelRosso-3 | Rahimi 2022 | Zhao-1 2022 | Zhao-2 2022 | Zhao-3 2022 | Zhao-4 2022 | Hejazi-1 2022 | Hejazi-2 2022 | Hejazi-3 2022 | Hejazi-4 2022 | Hejazi-5 2022 | Hejazi-6 2022 | Khalafi 2022 | Liu 2021 | Lee 1 2021 | Lee 2 2021 | Khalafi 2020 | Sirico 2018 | Garcia-Hermoso 2016 |
|---|---|---|---|---|---|---|---|---|---|---|---|---|---|---|---|---|---|---|---|---|---|---|---|---|---|---|---|---|---|---|---|---|---|---|---|---|---|---|
| Bocca 2014[37] | 0 | 1 | 0 | 0 | 0 | 0 | 0 | 0 | 0 | 0 | 0 | 0 | 0 | 0 | 0 | 0 | 0 | 0 | 0 | 0 | 0 | 0 | 0 | 0 | 0 | 0 | 0 | 0 | 0 | 0 | 0 | 0 | 0 | 0 | 0 | 0 | 0 | 0 |
| Bouchonville 2014[38] | 0 | 0 | 0 | 0 | 0 | 0 | 0 | 0 | 0 | 0 | 0 | 0 | 0 | 0 | 0 | 0 | 0 | 0 | 0 | 0 | 0 | 0 | 0 | 0 | 0 | 0 | 0 | 1 | 0 | 0 | 0 | 2 | 3 | 0 | 0 | 0 | 0 | 0 |
| Brandão Filho 2015[39] | 0 | 0 | 0 | 0 | 0 | 0 | 0 | 0 | 0 | 0 | 0 | 0 | 0 | 0 | 0 | 0 | 0 | 0 | 0 | 0 | 0 | 0 | 0 | 1 | 0 | 0 | 0 | 0 | 0 | 0 | 0 | 0 | 0 | 0 | 0 | 0 | 0 | 0 |
| Brochu 2009[40] | 0 | 0 | 0 | 0 | 0 | 0 | 0 | 0 | 0 | 0 | 0 | 0 | 0 | 0 | 0 | 0 | 0 | 0 | 0 | 0 | 0 | 0 | 0 | 0 | 0 | 0 | 0 | 0 | 0 | 0 | 0 | 0 | 1 | 0 | 0 | 0 | 0 | 0 |
| Brooks 2007[41] | 0 | 0 | 1 | 0 | 0 | 0 | 0 | 0 | 0 | 0 | 0 | 0 | 0 | 0 | 0 | 0 | 0 | 0 | 0 | 0 | 0 | 0 | 0 | 0 | 0 | 0 | 0 | 0 | 0 | 1 | 0 | 0 | 0 | 0 | 0 | 0 | 0 | 0 |
| Brunelli 2015[42] | 0 | 0 | 0 | 0 | 0 | 0 | 0 | 1 | 0 | 0 | 0 | 0 | 0 | 0 | 0 | 0 | 0 | 0 | 0 | 0 | 0 | 0 | 0 | 0 | 0 | 0 | 0 | 0 | 0 | 0 | 0 | 0 | 0 | 0 | 0 | 0 | 0 | 0 |
| Byrkjeland 2011[43] | 0 | 0 | 0 | 0 | 0 | 0 | 0 | 0 | 0 | 0 | 0 | 0 | 0 | 1 | 0 | 0 | 0 | 0 | 0 | 0 | 0 | 0 | 0 | 0 | 0 | 0 | 0 | 0 | 0 | 0 | 0 | 0 | 0 | 0 | 0 | 0 | 0 | 0 |
| Camhi 2010[44] | 0 | 0 | 0 | 0 | 0 | 0 | 0 | 0 | 0 | 0 | 0 | 0 | 0 | 0 | 0 | 0 | 0 | 0 | 0 | 0 | 1 | 0 | 0 | 0 | 0 | 0 | 0 | 0 | 0 | 0 | 0 | 0 | 0 | 0 | 0 | 0 | 0 | 0 |
| Campbell 2009[45] | 0 | 0 | 0 | 0 | 0 | 0 | 0 | 0 | 0 | 0 | 0 | 1 | 0 | 0 | 0 | 2 | 3 | 4 | 0 | 0 | 0 | 0 | 0 | 0 | 0 | 0 | 0 | 0 | 0 | 0 | 0 | 0 | 0 | 0 | 0 | 0 | 0 | 0 |
| Canuto 2012[46] | 0 | 0 | 0 | 0 | 0 | 0 | 0 | 0 | 0 | 0 | 0 | 0 | 0 | 0 | 0 | 0 | 0 | 0 | 0 | 1 | 0 | 0 | 0 | 0 | 0 | 0 | 0 | 0 | 0 | 0 | 0 | 0 | 0 | 0 | 0 | 0 | 0 | 0 |
| Chen 2015[47] | 0 | 0 | 0 | 0 | 0 | 0 | 0 | 0 | 0 | 0 | 0 | 1 | 2 | 0 | 0 | 0 | 0 | 0 | 0 | 0 | 0 | 3 | 4 | 5 | 0 | 0 | 0 | 0 | 0 | 0 | 0 | 0 | 0 | 0 | 0 | 0 | 0 | 0 |
| Cho 2019[48] | 0 | 0 | 0 | 0 | 0 | 0 | 0 | 0 | 0 | 0 | 0 | 0 | 0 | 0 | 0 | 0 | 0 | 0 | 0 | 0 | 0 | 0 | 0 | 0 | 0 | 0 | 0 | 0 | 0 | 0 | 0 | 1 | 2 | 0 | 0 | 0 | 0 | 0 |
| Choi 2012[49] | 0 | 0 | 0 | 0 | 0 | 0 | 0 | 0 | 0 | 0 | 0 | 0 | 0 | 0 | 0 | 0 | 0 | 1 | 0 | 0 | 0 | 0 | 0 | 0 | 0 | 0 | 0 | 0 | 0 | 0 | 0 | 0 | 0 | 0 | 0 | 0 | 0 | 0 |
| Christiansen 2010[50] | 0 | 0 | 0 | 0 | 0 | 0 | 0 | 0 | 0 | 0 | 0 | 0 | 0 | 0 | 0 | 0 | 0 | 1 | 0 | 0 | 0 | 0 | 0 | 0 | 0 | 0 | 0 | 0 | 0 | 0 | 0 | 0 | 0 | 0 | 0 | 0 | 0 | 0 |
| Church 2010[51] | 0 | 0 | 0 | 0 | 0 | 0 | 0 | 0 | 0 | 0 | 1 | 0 | 0 | 0 | 0 | 0 | 0 | 0 | 0 | 0 | 0 | 0 | 0 | 0 | 0 | 0 | 0 | 0 | 0 | 0 | 0 | 0 | 0 | 0 | 0 | 0 | 0 | 0 |
| Cobos-Palacios 2022[52] | 0 | 1 | 0 | 0 | 0 | 0 | 0 | 0 | 0 | 0 | 0 | 0 | 0 | 0 | 0 | 0 | 0 | 0 | 0 | 0 | 0 | 0 | 0 | 0 | 0 | 0 | 0 | 0 | 0 | 0 | 0 | 0 | 0 | 0 | 0 | 0 | 0 | 0 |
| Coll-Risco 2018[53] | 0 | 0 | 0 | 0 | 0 | 0 | 0 | 0 | 0 | 0 | 0 | 0 | 0 | 0 | 0 | 0 | 0 | 0 | 0 | 1 | 0 | 0 | 0 | 0 | 0 | 0 | 0 | 0 | 0 | 0 | 0 | 0 | 0 | 0 | 0 | 0 | 0 | 0 |
| Cordellat 2020[54] | 0 | 1 | 0 | 0 | 0 | 0 | 0 | 0 | 0 | 0 | 0 | 0 | 0 | 0 | 0 | 0 | 0 | 0 | 0 | 0 | 0 | 0 | 0 | 0 | 0 | 0 | 0 | 0 | 0 | 0 | 0 | 0 | 0 | 0 | 0 | 0 | 0 | 0 |
| Croymans 2014[55] | 0 | 0 | 0 | 0 | 0 | 0 | 0 | 0 | 0 | 0 | 0 | 0 | 1 | 0 | 0 | 0 | 0 | 0 | 2 | 0 | 0 | 0 | 0 | 0 | 0 | 0 | 0 | 0 | 0 | 0 | 0 | 0 | 0 | 0 | 0 | 0 | 0 | 0 |
| Cunha 2019[56] | 0 | 0 | 0 | 0 | 0 | 0 | 0 | 0 | 0 | 0 | 0 | 0 | 0 | 0 | 0 | 0 | 1 | 0 | 2 | 0 | 0 | 0 | 0 | 0 | 0 | 0 | 0 | 0 | 0 | 0 | 0 | 0 | 0 | 0 | 0 | 0 | 0 | 0 |
| da Silva 2012[57] | 0 | 1 | 0 | 0 | 0 | 0 | 0 | 0 | 0 | 0 | 0 | 0 | 0 | 0 | 0 | 0 | 0 | 0 | 0 | 0 | 0 | 0 | 0 | 0 | 0 | 0 | 0 | 0 | 0 | 0 | 0 | 0 | 0 | 0 | 0 | 0 | 0 | 0 |
| de Meirelles 2014[58] | 0 | 0 | 0 | 0 | 0 | 0 | 0 | 0 | 0 | 0 | 0 | 0 | 0 | 1 | 0 | 0 | 0 | 0 | 0 | 0 | 0 | 0 | 0 | 0 | 0 | 0 | 0 | 0 | 0 | 0 | 0 | 0 | 0 | 0 | 0 | 0 | 0 | 0 |
| Dieli-Conwright 2018[59] | 0 | 0 | 0 | 1 | 0 | 0 | 1 | 0 | 0 | 2 | 0 | 0 | 0 | 0 | 0 | 0 | 0 | 0 | 0 | 0 | 0 | 0 | 0 | 0 | 0 | 0 | 0 | 0 | 0 | 0 | 0 | 0 | 0 | 0 | 0 | 0 | 0 | 0 |
| Donges 2013[60] | 0 | 0 | 0 | 0 | 0 | 0 | 0 | 1 | 0 | 0 | 0 | 0 | 0 | 0 | 0 | 0 | 0 | 0 | 2 | 3 | 0 | 0 | 0 | 0 | 0 | 0 | 0 | 0 | 0 | 0 | 0 | 0 | 0 | 0 | 0 | 0 | 0 | 0 |
| Eleuteri 2013[61] | 0 | 0 | 0 | 0 | 0 | 0 | 0 | 0 | 0 | 0 | 0 | 0 | 0 | 1 | 0 | 0 | 0 | 0 | 0 | 0 | 0 | 0 | 0 | 0 | 0 | 0 | 0 | 0 | 0 | 0 | 0 | 0 | 0 | 0 | 0 | 0 | 0 | 0 |
| Fairey 2005[62] | 0 | 0 | 0 | 0 | 1 | 0 | 0 | 0 | 0 | 0 | 0 | 0 | 0 | 0 | 0 | 1 | 0 | 0 | 0 | 0 | 0 | 0 | 0 | 0 | 0 | 0 | 0 | 0 | 0 | 0 | 0 | 0 | 0 | 0 | 0 | 0 | 0 | 0 |
| Farpour-Lambert 2009[63] | 0 | 0 | 0 | 0 | 0 | 0 | 0 | 0 | 0 | 0 | 0 | 0 | 0 | 0 | 0 | 0 | 0 | 0 | 0 | 0 | 0 | 0 | 0 | 0 | 0 | 0 | 0 | 0 | 0 | 0 | 0 | 0 | 0 | 0 | 0 | 0 | 0 | 1 |
| Fedewa 2018[64] | 0 | 0 | 0 | 0 | 0 | 0 | 0 | 0 | 0 | 0 | 0 | 0 | 0 | 0 | 0 | 0 | 0 | 1 | 0 | 0 | 0 | 0 | 0 | 0 | 0 | 0 | 0 | 0 | 0 | 0 | 0 | 0 | 0 | 0 | 0 | 0 | 0 | 0 |
| Fisher 2011[65] | 0 | 0 | 0 | 0 | 0 | 0 | 0 | 0 | 0 | 0 | 0 | 0 | 0 | 0 | 0 | 0 | 0 | 0 | 1 | 0 | 0 | 0 | 0 | 0 | 0 | 0 | 0 | 0 | 0 | 0 | 0 | 0 | 2 | 0 | 0 | 0 | 0 | 0 |
| Flandez 2017[66] | 0 | 0 | 0 | 0 | 0 | 0 | 0 | 0 | 0 | 0 | 0 | 0 | 0 | 0 | 0 | 0 | 0 | 0 | 1 | 0 | 0 | 0 | 0 | 0 | 0 | 0 | 0 | 0 | 0 | 0 | 0 | 0 | 0 | 0 | 0 | 0 | 0 | 0 |

| Primary study | Zalagkitis 1 2025 | Zalagkitis 2 2025 | Wang 2025 | Hernandez-Martinez 2025 | Tan 1 2025 | Tan 2 2025 | Tan 3 2025 | Silva 2024 | Li 2024 | Al-Mhanna-1 2024 | Al-Mhanna-2 2024 | Guo 1 2024 | Guo 2 2024 | Malandish 2023 | Dragoumani 2023 | Tan-1 2023 | Tan-2 2023 | Del Rosso-1 | Del Rosso-2 | Del Rosso-3 | Rahimi 2022 | Zhao-1 2022 | Zhao-2 2022 | Zhao-3 2022 | Zhao-4 2022 | Hejazi-1 2022 | Hejazi-2 2022 | Hejazi-3 2022 | Hejazi-4 2022 | Hejazi-5 2022 | Hejazi-6 2022 | Khalafi 2022 | Liu 2021 | Lee 1 2021 | Lee 2 2021 | Khalafi 2020 | Sirico 2018 | Garcia-Hermoso 2016 |
|---|---|---|---|---|---|---|---|---|---|---|---|---|---|---|---|---|---|---|---|---|---|---|---|---|---|---|---|---|---|---|---|---|---|---|---|---|---|---|
| Franklin 2015[67] | 0 | 0 | 0 | 0 | 0 | 0 | 0 | 0 | 0 | 0 | 0 | 0 | 0 | 0 | 0 | 0 | 0 | 0 | 1 | 0 | 0 | 0 | 0 | 0 | 0 | 0 | 0 | 0 | 0 | 0 | 0 | 0 | 0 | 0 | 0 | 0 | 0 | 0 |
| Friedenreich 2012[68] | 0 | 0 | 0 | 0 | 0 | 0 | 0 | 0 | 0 | 0 | 0 | 0 | 0 | 0 | 0 | 0 | 0 | 1 | 0 | 0 | 0 | 0 | 0 | 0 | 0 | 0 | 0 | 0 | 0 | 0 | 0 | 0 | 0 | 0 | 0 | 0 | 0 | 0 |
| Garanty-Bogacka 2011[69] | 0 | 1 | 0 | 0 | 0 | 0 | 0 | 0 | 0 | 0 | 0 | 0 | 0 | 0 | 0 | 0 | 0 | 0 | 0 | 0 | 0 | 0 | 0 | 0 | 0 | 0 | 0 | 0 | 0 | 0 | 0 | 0 | 0 | 0 | 0 | 0 | 0 | 0 |
| Giallauria 2011[70] | 0 | 0 | 0 | 0 | 0 | 0 | 0 | 0 | 0 | 0 | 0 | 0 | 0 | 1 | 0 | 0 | 0 | 0 | 0 | 0 | 0 | 0 | 0 | 0 | 0 | 0 | 0 | 0 | 0 | 0 | 0 | 0 | 0 | 0 | 0 | 0 | 0 | 0 |
| Giannopoulou 2005[71] | 0 | 0 | 0 | 0 | 0 | 0 | 0 | 0 | 0 | 0 | 0 | 0 | 0 | 0 | 0 | 0 | 0 | 0 | 0 | 0 | 0 | 0 | 0 | 0 | 0 | 0 | 0 | 0 | 0 | 0 | 0 | 1 | 2 | 0 | 0 | 0 | 0 | 0 |
| Gomez-Tomas 2018[72] | 0 | 0 | 0 | 0 | 0 | 0 | 0 | 0 | 0 | 0 | 0 | 0 | 0 | 0 | 0 | 0 | 1 | 0 | 0 | 0 | 0 | 0 | 0 | 0 | 0 | 0 | 0 | 0 | 0 | 0 | 0 | 0 | 0 | 0 | 0 | 0 | 0 | 0 |
| Gong 2014[73] | 0 | 1 | 0 | 0 | 0 | 0 | 0 | 0 | 0 | 0 | 0 | 0 | 0 | 0 | 0 | 0 | 0 | 0 | 0 | 0 | 0 | 0 | 0 | 0 | 0 | 0 | 0 | 0 | 0 | 0 | 0 | 0 | 0 | 0 | 0 | 0 | 0 | 0 |
| Gram 2017[74] | 0 | 0 | 0 | 0 | 0 | 0 | 0 | 0 | 0 | 0 | 0 | 0 | 0 | 0 | 0 | 0 | 0 | 1 | 0 | 0 | 0 | 0 | 0 | 0 | 0 | 0 | 0 | 0 | 0 | 0 | 0 | 0 | 0 | 0 | 0 | 0 | 0 | 0 |
| Hagstrom 2016[75] | 0 | 0 | 0 | 0 | 0 | 1 | 0 | 0 | 0 | 0 | 0 | 0 | 0 | 0 | 0 | 0 | 0 | 0 | 0 | 0 | 0 | 0 | 0 | 0 | 0 | 0 | 0 | 0 | 0 | 0 | 0 | 0 | 0 | 0 | 0 | 0 | 0 | 0 |
| Henriquez 2017[76] | 0 | 0 | 0 | 0 | 0 | 0 | 0 | 0 | 0 | 0 | 0 | 0 | 0 | 0 | 0 | 0 | 0 | 0 | 1 | 0 | 0 | 0 | 0 | 0 | 0 | 0 | 0 | 0 | 0 | 0 | 0 | 0 | 0 | 0 | 0 | 0 | 0 | 0 |
| Herder 2009[77] | 0 | 0 | 0 | 0 | 0 | 0 | 0 | 0 | 0 | 0 | 0 | 0 | 0 | 0 | 0 | 0 | 0 | 1 | 0 | 0 | 2 | 0 | 0 | 0 | 0 | 0 | 0 | 0 | 0 | 0 | 0 | 0 | 0 | 0 | 0 | 0 | 0 | 0 |
| Imayama 2012[78] | 0 | 0 | 0 | 0 | 0 | 0 | 0 | 0 | 0 | 0 | 0 | 0 | 0 | 0 | 0 | 0 | 0 | 1 | 0 | 0 | 0 | 0 | 0 | 0 | 0 | 0 | 0 | 0 | 0 | 0 | 0 | 0 | 0 | 0 | 0 | 0 | 0 | 0 |
| Isaksen 2019[79] | 0 | 0 | 0 | 0 | 0 | 0 | 0 | 0 | 0 | 0 | 0 | 0 | 0 | 1 | 0 | 0 | 0 | 0 | 0 | 0 | 0 | 0 | 0 | 0 | 0 | 0 | 0 | 0 | 0 | 0 | 0 | 0 | 0 | 0 | 0 | 0 | 0 | 0 |
| Johannsen 2012[80] | 0 | 0 | 0 | 0 | 0 | 0 | 0 | 0 | 0 | 0 | 0 | 0 | 0 | 0 | 0 | 1 | 0 | 0 | 0 | 0 | 0 | 0 | 0 | 0 | 0 | 2 | 0 | 0 | 0 | 0 | 0 | 0 | 0 | 0 | 0 | 0 | 0 | 0 |
| Jones 2013[81] | 0 | 0 | 0 | 0 | 1 | 0 | 0 | 0 | 0 | 0 | 0 | 0 | 0 | 0 | 0 | 0 | 0 | 0 | 0 | 0 | 0 | 0 | 0 | 0 | 0 | 0 | 0 | 0 | 0 | 0 | 0 | 0 | 0 | 0 | 0 | 0 | 0 | 0 |
| Jorge 2011[82] | 0 | 0 | 1 | 0 | 0 | 0 | 0 | 0 | 0 | 0 | 0 | 0 | 0 | 0 | 0 | 0 | 0 | 0 | 2 | 3 | 0 | 0 | 0 | 0 | 0 | 0 | 0 | 0 | 4 | 5 | 6 | 0 | 0 | 0 | 0 | 0 | 0 | 0 |
| Kadoglou 2007[83] | 0 | 0 | 0 | 0 | 0 | 0 | 0 | 0 | 0 | 0 | 0 | 0 | 0 | 0 | 0 | 0 | 0 | 1 | 0 | 0 | 0 | 0 | 0 | 0 | 0 | 0 | 0 | 0 | 2 | 0 | 0 | 0 | 0 | 0 | 0 | 0 | 0 | 0 |
| Kadoglou 2012[84] | 0 | 0 | 1 | 0 | 0 | 0 | 0 | 0 | 0 | 0 | 0 | 0 | 0 | 0 | 0 | 0 | 0 | 0 | 2 | 0 | 0 | 0 | 0 | 0 | 0 | 0 | 0 | 0 | 0 | 0 | 0 | 0 | 0 | 0 | 0 | 0 | 0 | 0 |
| Kadoglou 2013[85] | 0 | 0 | 1 | 0 | 0 | 0 | 0 | 0 | 0 | 0 | 0 | 0 | 0 | 0 | 0 | 0 | 0 | 0 | 0 | 0 | 0 | 0 | 0 | 0 | 0 | 0 | 0 | 0 | 0 | 0 | 0 | 0 | 0 | 0 | 0 | 0 | 0 | 0 |
| Keating 2015[86] | 0 | 0 | 0 | 0 | 0 | 0 | 0 | 0 | 0 | 0 | 0 | 0 | 0 | 0 | 0 | 0 | 0 | 1 | 0 | 0 | 0 | 0 | 0 | 0 | 0 | 0 | 0 | 0 | 0 | 0 | 0 | 0 | 0 | 0 | 0 | 0 | 0 | 0 |
| Kelishadi 2008[87] | 1 | 0 | 0 | 0 | 0 | 0 | 0 | 0 | 0 | 0 | 0 | 0 | 0 | 0 | 0 | 0 | 0 | 0 | 0 | 0 | 0 | 0 | 0 | 0 | 0 | 0 | 0 | 0 | 0 | 0 | 0 | 0 | 0 | 0 | 0 | 0 | 0 | 0 |
| Kelly 2004[88] | 0 | 0 | 0 | 0 | 0 | 0 | 0 | 0 | 0 | 0 | 0 | 0 | 0 | 0 | 0 | 0 | 0 | 0 | 0 | 0 | 0 | 0 | 0 | 0 | 0 | 0 | 0 | 0 | 0 | 0 | 0 | 0 | 0 | 0 | 0 | 0 | 0 | 1 |
| Kelly 2007[89] | 0 | 0 | 0 | 0 | 0 | 0 | 0 | 0 | 1 | 0 | 0 | 2 | 0 | 0 | 0 | 0 | 0 | 0 | 0 | 0 | 0 | 0 | 0 | 0 | 0 | 0 | 0 | 0 | 0 | 0 | 0 | 0 | 0 | 0 | 0 | 0 | 0 | 0 |
| Khoo 2015[90] | 0 | 0 | 0 | 0 | 0 | 0 | 0 | 0 | 0 | 0 | 0 | 0 | 0 | 0 | 0 | 0 | 0 | 0 | 0 | 0 | 0 | 0 | 0 | 0 | 0 | 0 | 0 | 0 | 0 | 0 | 0 | 1 | 0 | 0 | 0 | 0 | 0 | 0 |
| Kim 2007[91] | 0 | 0 | 0 | 0 | 0 | 0 | 0 | 0 | 0 | 0 | 0 | 1 | 0 | 0 | 0 | 0 | 0 | 0 | 0 | 0 | 0 | 2 | 0 | 0 | 0 | 0 | 0 | 0 | 0 | 0 | 0 | 0 | 0 | 0 | 0 | 0 | 3 | 4 |
| Kim 2008[92] | 0 | 0 | 0 | 0 | 0 | 0 | 0 | 0 | 0 | 0 | 0 | 0 | 0 | 1 | 0 | 0 | 0 | 0 | 0 | 0 | 0 | 0 | 0 | 0 | 0 | 0 | 0 | 0 | 0 | 0 | 0 | 0 | 0 | 0 | 0 | 0 | 0 | 0 |
| Kim 2011[93] | 0 | 0 | 0 | 0 | 0 | 0 | 0 | 0 | 0 | 0 | 0 | 0 | 0 | 1 | 0 | 0 | 0 | 0 | 0 | 0 | 0 | 0 | 0 | 0 | 0 | 0 | 0 | 0 | 0 | 0 | 0 | 0 | 0 | 0 | 0 | 0 | 0 | 0 |
| Kim 2016[94] | 0 | 0 | 0 | 1 | 0 | 0 | 0 | 0 | 0 | 0 | 0 | 0 | 0 | 0 | 0 | 0 | 0 | 0 | 0 | 0 | 0 | 0 | 0 | 0 | 0 | 0 | 0 | 0 | 0 | 0 | 0 | 0 | 0 | 0 | 0 | 0 | 0 | 0 |
| Koh 2017[95] | 0 | 0 | 0 | 0 | 0 | 0 | 0 | 0 | 0 | 0 | 0 | 0 | 0 | 0 | 0 | 0 | 0 | 0 | 0 | 0 | 0 | 0 | 0 | 0 | 0 | 1 | 0 | 0 | 0 | 0 | 0 | 0 | 0 | 0 | 0 | 0 | 0 | 0 |
| Kolahdouzi 2019[96] | 0 | 0 | 0 | 0 | 0 | 0 | 0 | 0 | 0 | 0 | 0 | 0 | 0 | 0 | 0 | 0 | 0 | 0 | 1 | 0 | 0 | 0 | 0 | 0 | 0 | 0 | 0 | 0 | 0 | 0 | 0 | 0 | 0 | 0 | 0 | 0 | 0 | 0 |

| Primary study | Zalagkitis 1 2025 | Zalagkitis 2 2025 | Wang 2025 | Hernandez-Martinez 2025 | Tan 1 2025 | Tan 2 2025 | Tan 3 2025 | Silva 2024 | Li 2024 | Al-Mhanna-1 2024 | Al-Mhanna-2 2024 | Guo 1 2024 | Guo 2 2024 | Malandish 2023 | Dragouamani 2023 | Tan-1 2023 | Tan-2 2023 | DelRosso-1 | DelRosso-2 | DelRosso-3 | Rahimi 2022 | Zhao-1 2022 | Zhao-2 2022 | Zhao-3 2022 | Zhao-4 2022 | Hejazi-1 2022 | Hejazi-2 2022 | Hejazi-3 2022 | Hejazi-4 2022 | Hejazi-5 2022 | Hejazi-6 2022 | Khalafi 2022 | Liu 2021 | Lee 1 2021 | Lee 2 2021 | Khalafi 2020 | Sirico 2018 | Garcia-Hermoso 2016 |
|---|---|---|---|---|---|---|---|---|---|---|---|---|---|---|---|---|---|---|---|---|---|---|---|---|---|---|---|---|---|---|---|---|---|---|---|---|---|---|
| Kondo 2006[97] | 0 | 0 | 0 | 0 | 0 | 0 | 0 | 0 | 0 | 0 | 0 | 0 | 0 | 0 | 0 | 0 | 0 | 0 | 0 | 0 | 0 | 0 | 0 | 0 | 0 | 1 | 0 | 0 | 0 | 0 | 0 | 0 | 0 | 0 | 0 | 0 | 0 | 0 |
| Lam 2015[98] | 0 | 0 | 0 | 0 | 0 | 0 | 0 | 0 | 0 | 0 | 0 | 0 | 0 | 0 | 0 | 0 | 0 | 0 | 0 | 0 | 0 | 0 | 0 | 0 | 0 | 0 | 0 | 0 | 0 | 0 | 0 | 0 | 1 | 0 | 0 | 0 | 0 | 0 |
| Lambert 2008[99] | 0 | 0 | 0 | 0 | 0 | 0 | 0 | 0 | 0 | 0 | 0 | 0 | 0 | 0 | 0 | 0 | 0 | 0 | 0 | 0 | 0 | 0 | 0 | 0 | 0 | 0 | 0 | 0 | 0 | 0 | 0 | 1 | 0 | 0 | 0 | 0 | 0 | 0 |
| Lee 2010[100] | 0 | 0 | 0 | 0 | 0 | 0 | 0 | 0 | 0 | 0 | 0 | 0 | 0 | 0 | 0 | 0 | 0 | 0 | 0 | 0 | 0 | 1 | 0 | 2 | 0 | 0 | 0 | 0 | 0 | 0 | 0 | 0 | 0 | 0 | 0 | 0 | 0 | 0 |
| Lee 2012[101] | 0 | 0 | 0 | 0 | 0 | 0 | 0 | 0 | 0 | 0 | 0 | 1 | 0 | 0 | 0 | 0 | 0 | 0 | 0 | 0 | 0 | 0 | 0 | 0 | 0 | 0 | 0 | 0 | 0 | 0 | 0 | 0 | 0 | 0 | 0 | 0 | 0 | 0 |
| Libardi 2012[102] | 0 | 0 | 0 | 0 | 0 | 0 | 0 | 1 | 0 | 0 | 0 | 0 | 0 | 0 | 0 | 0 | 0 | 0 | 0 | 0 | 0 | 0 | 0 | 0 | 0 | 0 | 0 | 0 | 0 | 0 | 0 | 0 | 0 | 0 | 0 | 0 | 0 | 0 |
| Ligibel 2019[103] | 0 | 0 | 0 | 0 | 0 | 0 | 1 | 0 | 0 | 0 | 0 | 0 | 0 | 0 | 0 | 0 | 0 | 0 | 0 | 0 | 0 | 0 | 0 | 0 | 0 | 0 | 0 | 0 | 0 | 0 | 0 | 0 | 0 | 0 | 0 | 0 | 0 | 0 |
| Liu 2015[104] | 1 | 0 | 0 | 0 | 0 | 0 | 0 | 0 | 0 | 0 | 0 | 0 | 0 | 0 | 0 | 0 | 0 | 0 | 0 | 0 | 0 | 0 | 0 | 0 | 0 | 0 | 0 | 0 | 0 | 0 | 0 | 0 | 0 | 0 | 0 | 0 | 0 | 0 |
| Liu 2018[105] | 1 | 0 | 0 | 0 | 0 | 0 | 0 | 0 | 2 | 0 | 0 | 0 | 0 | 0 | 0 | 0 | 0 | 0 | 0 | 0 | 0 | 0 | 0 | 0 | 0 | 0 | 0 | 0 | 0 | 0 | 0 | 0 | 0 | 0 | 0 | 0 | 0 | 0 |
| Lopes 2016[106] | 0 | 0 | 0 | 0 | 0 | 0 | 0 | 0 | 0 | 0 | 0 | 0 | 0 | 0 | 0 | 0 | 0 | 0 | 0 | 0 | 0 | 0 | 0 | 1 | 0 | 0 | 0 | 0 | 0 | 0 | 0 | 0 | 0 | 0 | 0 | 0 | 0 | 0 |
| Loria-Kohen 2013[107] | 0 | 0 | 0 | 0 | 0 | 0 | 0 | 0 | 0 | 0 | 0 | 0 | 0 | 0 | 0 | 0 | 0 | 0 | 1 | 2 | 0 | 0 | 0 | 0 | 0 | 0 | 0 | 0 | 0 | 0 | 0 | 0 | 0 | 0 | 0 | 0 | 0 | 0 |
| Magalhães 2019[108] | 0 | 0 | 0 | 0 | 0 | 0 | 0 | 0 | 0 | 0 | 1 | 0 | 0 | 0 | 0 | 0 | 0 | 0 | 0 | 0 | 0 | 0 | 0 | 0 | 0 | 0 | 0 | 0 | 0 | 0 | 0 | 0 | 0 | 0 | 0 | 0 | 0 | 0 |
| Magalhães 2020[109] | 0 | 0 | 0 | 0 | 0 | 0 | 0 | 0 | 0 | 0 | 0 | 0 | 0 | 0 | 0 | 0 | 0 | 1 | 0 | 0 | 0 | 0 | 0 | 0 | 0 | 0 | 0 | 0 | 0 | 0 | 0 | 0 | 0 | 0 | 0 | 0 | 0 | 0 |
| Marco 2013[110] | 0 | 0 | 0 | 0 | 0 | 0 | 0 | 0 | 0 | 0 | 0 | 0 | 0 | 1 | 0 | 0 | 0 | 0 | 0 | 0 | 0 | 0 | 0 | 0 | 0 | 0 | 0 | 0 | 0 | 0 | 0 | 0 | 0 | 0 | 0 | 0 | 0 | 0 |
| Marcell 2005[111] | 0 | 0 | 0 | 0 | 0 | 0 | 0 | 0 | 0 | 0 | 0 | 0 | 0 | 0 | 0 | 0 | 0 | 1 | 0 | 0 | 0 | 0 | 0 | 0 | 0 | 0 | 0 | 0 | 0 | 0 | 0 | 0 | 0 | 0 | 0 | 0 | 0 | 0 |
| Martins 2010[112] | 0 | 0 | 0 | 0 | 0 | 0 | 0 | 0 | 0 | 0 | 0 | 0 | 0 | 0 | 0 | 0 | 0 | 0 | 1 | 0 | 0 | 0 | 0 | 0 | 0 | 0 | 0 | 0 | 0 | 0 | 0 | 0 | 0 | 0 | 0 | 0 | 0 | 0 |
| Martins 2018[113] | 0 | 0 | 0 | 0 | 0 | 0 | 0 | 0 | 0 | 0 | 0 | 0 | 0 | 0 | 0 | 0 | 0 | 1 | 0 | 0 | 0 | 0 | 0 | 0 | 0 | 0 | 0 | 0 | 0 | 0 | 0 | 0 | 0 | 0 | 0 | 0 | 0 | 0 |
| Masquio 2023[114] | 0 | 1 | 0 | 0 | 0 | 0 | 0 | 0 | 0 | 0 | 0 | 0 | 0 | 0 | 0 | 0 | 0 | 0 | 0 | 0 | 0 | 0 | 0 | 0 | 0 | 0 | 0 | 0 | 0 | 0 | 0 | 0 | 0 | 0 | 0 | 0 | 0 | 0 |
| MastersonCreber 2015[115] | 0 | 0 | 0 | 0 | 0 | 0 | 0 | 0 | 0 | 0 | 0 | 0 | 0 | 1 | 0 | 0 | 0 | 0 | 0 | 0 | 0 | 0 | 0 | 0 | 0 | 0 | 0 | 0 | 0 | 0 | 0 | 0 | 0 | 0 | 0 | 0 | 0 | 0 |
| Mavros 2014[116] | 0 | 0 | 0 | 0 | 0 | 0 | 0 | 0 | 0 | 0 | 0 | 0 | 0 | 0 | 0 | 0 | 0 | 0 | 1 | 0 | 0 | 0 | 0 | 0 | 0 | 0 | 0 | 0 | 0 | 0 | 0 | 0 | 0 | 0 | 0 | 0 | 0 | 0 |
| Mayerhofer 2020[117] | 0 | 1 | 0 | 0 | 0 | 0 | 0 | 0 | 0 | 0 | 0 | 0 | 0 | 0 | 0 | 0 | 0 | 0 | 0 | 0 | 0 | 0 | 0 | 0 | 0 | 0 | 0 | 0 | 0 | 0 | 0 | 0 | 0 | 0 | 0 | 0 | 0 | 0 |
| McDermott 2004[118] | 0 | 0 | 0 | 0 | 0 | 0 | 0 | 0 | 0 | 0 | 0 | 0 | 0 | 1 | 0 | 0 | 0 | 0 | 0 | 0 | 0 | 0 | 0 | 0 | 0 | 0 | 0 | 0 | 0 | 0 | 0 | 0 | 0 | 0 | 0 | 0 | 0 | 0 |
| Mendham 2015[119] | 0 | 0 | 0 | 0 | 0 | 0 | 0 | 0 | 0 | 0 | 0 | 0 | 0 | 0 | 0 | 0 | 0 | 0 | 0 | 1 | 0 | 0 | 0 | 0 | 0 | 0 | 0 | 0 | 1 | 0 | 0 | 0 | 0 | 0 | 0 | 0 | 0 | 0 |
| Meyer 2006[120] | 0 | 0 | 0 | 0 | 0 | 0 | 0 | 0 | 0 | 0 | 0 | 0 | 0 | 0 | 0 | 0 | 0 | 0 | 0 | 0 | 0 | 1 | 0 | 0 | 0 | 0 | 0 | 0 | 0 | 0 | 0 | 0 | 0 | 0 | 0 | 0 | 0 | 2 |
| Mietus-Snyder 2020[121] | 0 | 0 | 0 | 0 | 0 | 0 | 0 | 0 | 0 | 0 | 0 | 0 | 0 | 0 | 1 | 0 | 0 | 0 | 0 | 0 | 0 | 0 | 0 | 0 | 0 | 0 | 0 | 0 | 0 | 0 | 0 | 0 | 0 | 0 | 0 | 0 | 0 | 0 |

| Primary study | Zalagkitis 1 2025 | Zalagkitis 2 2025 | Wang 2025 | Hernandez-Martinez 2025 | Tan 1 2025 | Tan 2 2025 | Tan 3 2025 | Silva 2024 | Li 2024 | Al-Mhanna-1 2024 | Al-Mhanna-2 2024 | Guo 1 2024 | Guo 2 2024 | Malandish 2023 | Dragoumani 2023 | Tan-1 2023 | Tan-2 2023 | DelRosso-1 | DelRosso-2 | DelRosso-3 | Rahimi 2022 | Zhao-1 2022 | Zhao-2 2022 | Zhao-3 2022 | Zhao-4 2022 | Hejazi-1 2022 | Hejazi-2 2022 | Hejazi-3 2022 | Hejazi-4 2022 | Hejazi-5 2022 | Hejazi-6 2022 | Khalafi 2022 | Liu 2021 | Lee 1 2021 | Lee 2 2021 | Khalafi 2020 | Sirico 2018 | Garcia-Hermoso 2016 |
|---|---|---|---|---|---|---|---|---|---|---|---|---|---|---|---|---|---|---|---|---|---|---|---|---|---|---|---|---|---|---|---|---|---|---|---|---|---|---|
| Milani 2004[122] | 0 | 0 | 0 | 0 | 0 | 0 | 0 | 0 | 0 | 0 | 0 | 0 | 0 | 1 | 0 | 0 | 0 | 0 | 0 | 0 | 0 | 0 | 0 | 0 | 0 | 0 | 0 | 0 | 0 | 0 | 0 | 0 | 0 | 0 | 0 | 0 | 0 | 0 |
| Moghadasi 2011[123] | 0 | 0 | 0 | 0 | 0 | 0 | 0 | 0 | 0 | 0 | 0 | 0 | 0 | 0 | 0 | 0 | 0 | 0 | 0 | 0 | 0 | 0 | 0 | 0 | 0 | 0 | 0 | 0 | 1 | 0 | 0 | 0 | 0 | 0 | 0 | 0 | 0 | 0 |
| Moghadasi 2012[124] | 0 | 0 | 0 | 0 | 0 | 0 | 0 | 0 | 0 | 0 | 0 | 0 | 0 | 0 | 0 | 0 | 0 | 1 | 0 | 0 | 0 | 0 | 0 | 0 | 0 | 0 | 0 | 0 | 0 | 0 | 0 | 0 | 0 | 0 | 0 | 0 | 0 | 0 |
| Montero 2019[125] | 0 | 1 | 0 | 0 | 0 | 0 | 0 | 0 | 0 | 0 | 0 | 0 | 0 | 0 | 0 | 0 | 0 | 0 | 0 | 0 | 0 | 0 | 0 | 0 | 0 | 0 | 0 | 0 | 0 | 0 | 0 | 0 | 0 | 0 | 0 | 0 | 0 | 0 |
| Mora-Rodriguez 2018[126] | 0 | 0 | 0 | 0 | 0 | 0 | 0 | 0 | 0 | 0 | 0 | 0 | 0 | 0 | 0 | 0 | 0 | 1 | 0 | 0 | 0 | 0 | 0 | 0 | 0 | 0 | 0 | 0 | 0 | 0 | 0 | 0 | 0 | 0 | 0 | 1 | 0 | 0 |
| Moraes 2016[127] | 0 | 1 | 0 | 0 | 0 | 0 | 0 | 0 | 0 | 0 | 0 | 0 | 0 | 0 | 0 | 0 | 0 | 0 | 0 | 0 | 0 | 0 | 0 | 0 | 0 | 0 | 0 | 0 | 0 | 0 | 0 | 0 | 0 | 0 | 0 | 0 | 0 | 0 |
| Murphy 2009[128] | 0 | 0 | 0 | 0 | 0 | 0 | 0 | 0 | 1 | 0 | 0 | 0 | 0 | 0 | 0 | 0 | 0 | 0 | 0 | 0 | 0 | 0 | 0 | 0 | 0 | 0 | 0 | 0 | 0 | 0 | 0 | 0 | 0 | 2 | 3 | 0 | 0 | 4 |
| Myers 2010[129] | 0 | 0 | 0 | 0 | 0 | 0 | 0 | 0 | 0 | 0 | 0 | 0 | 0 | 1 | 0 | 0 | 0 | 0 | 0 | 0 | 0 | 0 | 0 | 0 | 0 | 0 | 0 | 0 | 0 | 0 | 0 | 0 | 0 | 0 | 0 | 0 | 0 | 0 |
| Nadi 2019[130] | 0 | 0 | 1 | 0 | 0 | 0 | 0 | 0 | 0 | 0 | 0 | 0 | 0 | 0 | 0 | 0 | 0 | 0 | 0 | 0 | 0 | 0 | 0 | 0 | 0 | 0 | 0 | 0 | 0 | 0 | 0 | 0 | 0 | 0 | 0 | 0 | 0 | 0 |
| Nanri 2012[131] | 0 | 0 | 0 | 0 | 0 | 0 | 0 | 0 | 0 | 0 | 0 | 0 | 0 | 0 | 0 | 0 | 0 | 0 | 0 | 0 | 1 | 0 | 0 | 0 | 0 | 0 | 0 | 0 | 0 | 0 | 0 | 0 | 0 | 0 | 0 | 0 | 0 | 0 |
| Nicklas 2004[132] | 0 | 0 | 0 | 0 | 0 | 0 | 0 | 0 | 0 | 0 | 0 | 0 | 0 | 0 | 0 | 0 | 0 | 0 | 0 | 0 | 0 | 0 | 0 | 0 | 0 | 0 | 0 | 0 | 0 | 0 | 0 | 1 | 2 | 0 | 0 | 0 | 0 | 0 |
| Nikseresht 2014[133] | 0 | 0 | 0 | 0 | 0 | 0 | 0 | 0 | 0 | 0 | 0 | 0 | 0 | 0 | 0 | 0 | 0 | 0 | 0 | 0 | 0 | 0 | 0 | 0 | 0 | 1 | 2 | 0 | 0 | 0 | 0 | 0 | 0 | 0 | 0 | 3 | 0 | 0 |
| Nişancı Kılınç 2013[134] | 0 | 1 | 0 | 0 | 0 | 0 | 0 | 0 | 0 | 0 | 0 | 0 | 0 | 0 | 0 | 0 | 0 | 0 | 0 | 0 | 0 | 0 | 0 | 0 | 0 | 0 | 0 | 0 | 0 | 0 | 0 | 0 | 0 | 0 | 0 | 0 | 0 | 0 |
| Nono Nankam 2020[135] | 0 | 0 | 0 | 0 | 0 | 0 | 0 | 0 | 0 | 0 | 0 | 0 | 0 | 0 | 0 | 0 | 0 | 0 | 0 | 1 | 0 | 0 | 0 | 0 | 0 | 0 | 0 | 0 | 0 | 0 | 0 | 0 | 0 | 0 | 0 | 0 | 0 | 0 |
| Nunes 2016[136] | 0 | 1 | 0 | 0 | 0 | 0 | 0 | 0 | 0 | 0 | 0 | 0 | 0 | 0 | 0 | 0 | 0 | 0 | 0 | 0 | 0 | 0 | 0 | 2 | 0 | 0 | 0 | 0 | 0 | 0 | 0 | 0 | 0 | 0 | 0 | 0 | 0 | 0 |
| Oh 2013[137] | 0 | 0 | 0 | 0 | 0 | 0 | 0 | 0 | 0 | 0 | 0 | 0 | 0 | 0 | 0 | 0 | 0 | 0 | 0 | 0 | 1 | 0 | 0 | 0 | 0 | 0 | 0 | 0 | 0 | 0 | 0 | 0 | 0 | 0 | 0 | 0 | 0 | 0 |
| Oh 2014[138] | 0 | 0 | 0 | 0 | 0 | 0 | 0 | 0 | 0 | 0 | 0 | 0 | 0 | 0 | 0 | 0 | 0 | 0 | 0 | 0 | 0 | 0 | 0 | 0 | 0 | 0 | 0 | 0 | 0 | 0 | 0 | 0 | 1 | 0 | 0 | 0 | 0 | 0 |
| Okada 2010[139] | 0 | 0 | 0 | 0 | 0 | 0 | 0 | 0 | 0 | 0 | 0 | 0 | 0 | 0 | 0 | 0 | 0 | 0 | 0 | 1 | 0 | 0 | 0 | 0 | 0 | 0 | 0 | 0 | 0 | 0 | 2 | 0 | 0 | 0 | 0 | 0 | 0 | 0 |
| Olson 2007[140] | 0 | 0 | 0 | 0 | 0 | 0 | 0 | 0 | 0 | 0 | 0 | 0 | 0 | 0 | 0 | 0 | 0 | 0 | 1 | 0 | 0 | 0 | 0 | 0 | 0 | 0 | 2 | 0 | 0 | 0 | 0 | 0 | 0 | 0 | 0 | 0 | 0 | 0 |
| Park 2007[141] | 0 | 0 | 0 | 0 | 0 | 0 | 0 | 0 | 0 | 0 | 0 | 0 | 0 | 0 | 1 | 0 | 0 | 0 | 0 | 0 | 0 | 2 | 0 | 0 | 0 | 0 | 0 | 0 | 0 | 0 | 0 | 0 | 0 | 0 | 0 | 0 | 0 | 3 |
| Park 2012[142] | 0 | 0 | 0 | 0 | 0 | 0 | 0 | 0 | 0 | 0 | 0 | 0 | 0 | 0 | 0 | 0 | 0 | 0 | 0 | 0 | 0 | 0 | 0 | 1 | 0 | 0 | 0 | 0 | 0 | 0 | 0 | 0 | 0 | 0 | 0 | 0 | 0 | 0 |
| Park 2017[143] | 0 | 0 | 0 | 1 | 0 | 0 | 0 | 0 | 0 | 0 | 0 | 0 | 0 | 0 | 0 | 0 | 0 | 0 | 0 | 0 | 0 | 0 | 0 | 0 | 0 | 0 | 0 | 0 | 0 | 0 | 0 | 0 | 0 | 0 | 0 | 0 | 0 | 0 |
| Parrinello 2010[144] | 0 | 0 | 0 | 0 | 0 | 0 | 0 | 0 | 0 | 0 | 0 | 0 | 0 | 1 | 0 | 0 | 0 | 0 | 0 | 0 | 0 | 0 | 0 | 0 | 0 | 0 | 0 | 0 | 0 | 0 | 0 | 0 | 0 | 0 | 0 | 0 | 0 | 0 |
| Pedersen 2016[145] | 0 | 0 | 0 | 0 | 0 | 0 | 0 | 0 | 0 | 0 | 0 | 0 | 0 | 0 | 0 | 0 | 0 | 0 | 0 | 0 | 0 | 0 | 0 | 0 | 0 | 0 | 0 | 0 | 0 | 0 | 0 | 1 | 0 | 0 | 0 | 0 | 0 | 0 |
| Pérez-López 2021[146] | 0 | 0 | 0 | 0 | 0 | 0 | 0 | 1 | 0 | 0 | 0 | 2 | 0 | 0 | 0 | 0 | 0 | 0 | 0 | 0 | 0 | 0 | 0 | 0 | 0 | 0 | 0 | 0 | 0 | 0 | 0 | 0 | 0 | 0 | 0 | 0 | 0 | 0 |

| Primary study | Zalagkitis 1 2025 | Zalagkitis 2 2025 | Wang 2025 | Hernandez-Martinez 2025 | Tan 1 2025 | Tan 2 2025 | Tan 3 2025 | Silva 2024 | Li 2024 | Al-Mhanna-1 2024 | Al-Mhanna-2 2024 | Guo 1 2024 | Guo 2 2024 | Malandish 2023 | Dragoumani 2023 | Tan-1 2023 | Tan-2 2023 | DelRosso-1 | DelRosso-2 | DelRosso-3 | Rahimi 2022 | Zhao-1 2022 | Zhao-2 2022 | Zhao-3 2022 | Zhao-4 2022 | Hejazi-1 2022 | Hejazi-2 2022 | Hejazi-3 2022 | Hejazi-4 2022 | Hejazi-5 2022 | Hejazi-6 2022 | Khalafi 2022 | Liu 2021 | Lee 1 2021 | Lee 2 2021 | Khalafi 2020 | Sirico 2018 | Garcia-Hermoso 2016 |
|---|---|---|---|---|---|---|---|---|---|---|---|---|---|---|---|---|---|---|---|---|---|---|---|---|---|---|---|---|---|---|---|---|---|---|---|---|---|---|
| Phillips 2012[147] | 0 | 0 | 0 | 0 | 0 | 0 | 0 | 0 | 0 | 0 | 0 | 0 | 0 | 0 | 0 | 0 | 1 | 0 | 2 | 0 | 0 | 0 | 0 | 0 | 0 | 0 | 3 | 0 | 0 | 0 | 0 | 0 | 0 | 0 | 0 | 0 | 0 | 0 |
| Pierce 2008[148] | 0 | 0 | 0 | 0 | 0 | 0 | 0 | 0 | 0 | 0 | 0 | 0 | 0 | 1 | 0 | 0 | 0 | 0 | 0 | 0 | 0 | 0 | 0 | 0 | 0 | 0 | 0 | 0 | 0 | 0 | 0 | 0 | 0 | 0 | 0 | 0 | 0 | 0 |
| Plavsic 2020[149] | 1 | 0 | 0 | 0 | 0 | 0 | 0 | 0 | 0 | 0 | 0 | 0 | 0 | 0 | 0 | 0 | 0 | 0 | 0 | 0 | 0 | 0 | 0 | 0 | 2 | 0 | 0 | 0 | 0 | 0 | 0 | 0 | 0 | 0 | 0 | 0 | 0 | 0 |
| Plotnikoff 2010[150] | 0 | 0 | 1 | 0 | 0 | 0 | 0 | 0 | 0 | 0 | 0 | 0 | 0 | 0 | 0 | 0 | 0 | 0 | 2 | 0 | 0 | 0 | 0 | 0 | 0 | 0 | 0 | 0 | 0 | 0 | 0 | 0 | 0 | 0 | 0 | 0 | 0 | 0 |
| Prescott 2009[151] | 0 | 0 | 0 | 0 | 0 | 0 | 0 | 0 | 0 | 0 | 0 | 0 | 0 | 1 | 0 | 0 | 0 | 0 | 0 | 0 | 0 | 0 | 0 | 0 | 0 | 0 | 0 | 0 | 0 | 0 | 0 | 0 | 0 | 0 | 0 | 0 | 0 | 0 |
| Pullen 2008[152] | 0 | 0 | 0 | 0 | 0 | 0 | 0 | 0 | 0 | 0 | 0 | 0 | 0 | 1 | 0 | 0 | 0 | 0 | 0 | 0 | 0 | 0 | 0 | 0 | 0 | 0 | 0 | 0 | 0 | 0 | 0 | 0 | 0 | 0 | 0 | 0 | 0 | 0 |
| Rahbar 2017[153] | 0 | 0 | 0 | 0 | 0 | 0 | 0 | 0 | 0 | 0 | 0 | 0 | 0 | 0 | 0 | 0 | 0 | 1 | 0 | 0 | 0 | 0 | 0 | 0 | 0 | 0 | 0 | 0 | 0 | 0 | 0 | 0 | 0 | 0 | 0 | 0 | 0 | 0 |
| Ranković 2009[154] | 0 | 0 | 0 | 0 | 0 | 0 | 0 | 0 | 0 | 0 | 0 | 0 | 0 | 1 | 0 | 0 | 0 | 0 | 0 | 0 | 0 | 0 | 0 | 0 | 0 | 0 | 0 | 0 | 0 | 0 | 0 | 0 | 0 | 0 | 0 | 0 | 0 | 0 |
| Ratajczak 2019[155] | 0 | 0 | 0 | 0 | 0 | 0 | 0 | 0 | 0 | 0 | 0 | 0 | 0 | 0 | 0 | 0 | 0 | 1 | 0 | 0 | 0 | 0 | 0 | 0 | 0 | 0 | 0 | 0 | 0 | 0 | 0 | 0 | 0 | 0 | 0 | 0 | 0 | 0 |
| Rech 2019[156] | 0 | 0 | 1 | 0 | 0 | 0 | 0 | 0 | 0 | 0 | 0 | 0 | 0 | 0 | 0 | 0 | 0 | 0 | 0 | 0 | 0 | 0 | 0 | 0 | 0 | 0 | 0 | 0 | 0 | 0 | 0 | 0 | 0 | 0 | 0 | 0 | 0 | 0 |
| Redwine 2020[157] | 0 | 0 | 0 | 0 | 0 | 0 | 0 | 0 | 0 | 0 | 0 | 0 | 0 | 1 | 0 | 0 | 0 | 0 | 0 | 0 | 0 | 0 | 0 | 0 | 0 | 0 | 0 | 0 | 0 | 0 | 0 | 0 | 0 | 0 | 0 | 0 | 0 | 0 |
| Reinehr 2005[158] | 0 | 1 | 0 | 0 | 0 | 0 | 0 | 0 | 0 | 0 | 0 | 0 | 0 | 0 | 0 | 0 | 0 | 0 | 0 | 0 | 0 | 0 | 0 | 0 | 0 | 0 | 0 | 0 | 0 | 0 | 0 | 0 | 0 | 0 | 0 | 0 | 0 | 0 |
| Rejeski 2019[159] | 0 | 0 | 0 | 0 | 0 | 0 | 0 | 0 | 0 | 0 | 0 | 0 | 0 | 0 | 0 | 0 | 0 | 0 | 1 | 0 | 0 | 0 | 0 | 0 | 0 | 0 | 0 | 0 | 0 | 0 | 0 | 0 | 2 | 0 | 0 | 0 | 0 | 0 |
| Reljic 2022[160] | 0 | 0 | 0 | 0 | 0 | 0 | 0 | 0 | 0 | 0 | 0 | 0 | 0 | 0 | 0 | 0 | 0 | 1 | 0 | 0 | 0 | 0 | 0 | 0 | 0 | 0 | 0 | 0 | 0 | 0 | 0 | 0 | 0 | 0 | 0 | 0 | 0 | 0 |
| Roberts 2007[161] | 1 | 0 | 0 | 0 | 0 | 0 | 0 | 0 | 0 | 0 | 0 | 0 | 0 | 0 | 0 | 0 | 0 | 0 | 0 | 0 | 0 | 0 | 0 | 0 | 0 | 0 | 0 | 0 | 0 | 0 | 0 | 0 | 0 | 0 | 0 | 0 | 0 | 0 |
| Roche 2019[162] | 0 | 1 | 0 | 0 | 0 | 0 | 0 | 0 | 0 | 0 | 0 | 0 | 0 | 0 | 0 | 0 | 0 | 0 | 0 | 0 | 0 | 0 | 0 | 0 | 0 | 0 | 0 | 0 | 0 | 0 | 0 | 0 | 0 | 0 | 0 | 0 | 0 | 0 |
| Rodriguez-Krause 2018[163] | 0 | 0 | 0 | 0 | 0 | 0 | 0 | 0 | 0 | 0 | 0 | 0 | 0 | 0 | 0 | 0 | 0 | 1 | 0 | 0 | 0 | 0 | 0 | 0 | 0 | 0 | 0 | 0 | 0 | 0 | 0 | 0 | 0 | 0 | 0 | 0 | 0 | 0 |
| Rohde 2018[164] | 0 | 0 | 0 | 0 | 0 | 0 | 0 | 0 | 0 | 0 | 0 | 0 | 0 | 0 | 0 | 0 | 0 | 1 | 0 | 0 | 0 | 0 | 0 | 0 | 0 | 0 | 0 | 0 | 0 | 0 | 0 | 0 | 0 | 0 | 0 | 0 | 0 | 0 |
| Rokling-Andersen 2007[165] | 0 | 0 | 0 | 0 | 0 | 0 | 0 | 0 | 0 | 0 | 0 | 0 | 0 | 0 | 0 | 0 | 0 | 0 | 0 | 1 | 0 | 0 | 0 | 0 | 0 | 0 | 0 | 0 | 2 | 0 | 0 | 3 | 0 | 0 | 0 | 0 | 0 | 0 |
| Ryan 2014[166] | 0 | 0 | 0 | 0 | 0 | 0 | 0 | 0 | 0 | 0 | 0 | 0 | 0 | 0 | 0 | 1 | 0 | 0 | 0 | 0 | 0 | 0 | 0 | 0 | 0 | 0 | 0 | 0 | 0 | 0 | 0 | 0 | 2 | 0 | 0 | 0 | 0 | 0 |
| Sabouri 2021[167] | 0 | 0 | 1 | 0 | 0 | 0 | 0 | 0 | 0 | 0 | 0 | 0 | 0 | 0 | 0 | 0 | 0 | 0 | 0 | 0 | 0 | 0 | 0 | 0 | 0 | 0 | 0 | 0 | 0 | 0 | 0 | 0 | 0 | 0 | 0 | 0 | 0 | 0 |
| Saeidi 2020[168] | 0 | 0 | 0 | 0 | 0 | 0 | 0 | 0 | 0 | 0 | 0 | 1 | 0 | 0 | 0 | 0 | 0 | 0 | 0 | 0 | 0 | 0 | 0 | 0 | 0 | 0 | 0 | 0 | 0 | 0 | 0 | 0 | 0 | 0 | 0 | 0 | 0 | 0 |
| Santiprabhob 2018[169] | 0 | 1 | 0 | 0 | 0 | 0 | 0 | 0 | 0 | 0 | 0 | 0 | 0 | 0 | 0 | 0 | 0 | 0 | 0 | 0 | 0 | 0 | 0 | 0 | 0 | 0 | 0 | 0 | 0 | 0 | 0 | 0 | 0 | 0 | 0 | 0 | 0 | 0 |
| Santomauro 2012[170] | 0 | 1 | 0 | 0 | 0 | 0 | 0 | 0 | 0 | 0 | 0 | 0 | 0 | 0 | 0 | 0 | 0 | 0 | 0 | 0 | 0 | 0 | 0 | 0 | 0 | 0 | 0 | 0 | 0 | 0 | 0 | 0 | 0 | 0 | 0 | 0 | 0 | 0 |
| Scott 2013[17 | 0 | 0 | 0 | 0 | 0 | 0 | 0 | 0 | 0 | 1 | 0 | 0 | 0 | 0 | 0 | 0 | 0 | 0 | 0 | 0 | 0 | 0 | 0 | 0 | 0 | 0 | 0 | 0 | 0 | 0 | 0 | 2 | 0 | 0 | 0 | 0 | 0 | 0 |

| Primary study | Zalagkitis 1 2025 | Zalagkitis 2 2025 | Wang 2025 | Hernandez-Martinez 2025 | Tan 1 2025 | Tan 2 2025 | Tan 3 2025 | Silva 2024 | Li 2024 | Al-Mhanna-1 2024 | Al-Mhanna-2 2024 | Guo 1 2024 | Guo 2 2024 | Malandish 2023 | Dragoumani 2023 | Tan-1 2023 | Tan-2 2023 | Del Rosso-1 | Del Rosso-2 | Del Rosso-3 | Rahimi 2022 | Zhao-1 2022 | Zhao-2 2022 | Zhao-3 2022 | Zhao-4 2022 | Hejazi-1 2022 | Hejazi-2 2022 | Hejazi-3 2022 | Hejazi-4 2022 | Hejazi-5 2022 | Hejazi-6 2022 | Khalafi 2022 | Liu 2021 | Lee 1 2021 | Lee 2 2021 | Khalafi 2020 | Sirico 2018 | Garcia-Hermoso 2016 |
|---|---|---|---|---|---|---|---|---|---|---|---|---|---|---|---|---|---|---|---|---|---|---|---|---|---|---|---|---|---|---|---|---|---|---|---|---|---|---|
| 1] | | | | | | | | | | | | | | | | | | | | | | | | | | | | | | | | | | | | | | |
| Seo 2019[172] | 0 | 0 | 0 | 0 | 0 | 0 | 0 | 0 | 0 | 0 | 0 | 0 | 0 | 0 | 1 | 0 | 0 | 0 | 0 | 0 | 0 | 0 | 0 | 0 | 0 | 0 | 0 | 0 | 0 | 0 | 0 | 0 | 0 | 0 | 0 | 0 | 0 | 0 |
| Shabani 2019[173] | 0 | 0 | 0 | 0 | 0 | 0 | 0 | 1 | 0 | 0 | 0 | 0 | 0 | 0 | 0 | 0 | 0 | 0 | 0 | 0 | 0 | 0 | 0 | 0 | 0 | 0 | 0 | 0 | 0 | 0 | 0 | 0 | 0 | 0 | 0 | 0 | 0 | 0 |
| Shalitin 2009[174] | 1 | 0 | 0 | 0 | 0 | 0 | 0 | 0 | 0 | 0 | 0 | 0 | 0 | 0 | 0 | 0 | 0 | 0 | 0 | 0 | 0 | 0 | 0 | 0 | 0 | 0 | 0 | 0 | 0 | 0 | 0 | 0 | 0 | 0 | 0 | 0 | 0 | 0 |
| Shin 2006[175] | 0 | 0 | 0 | 0 | 0 | 0 | 0 | 0 | 0 | 0 | 0 | 0 | 0 | 1 | 0 | 0 | 0 | 0 | 0 | 0 | 0 | 0 | 0 | 0 | 0 | 0 | 0 | 0 | 0 | 0 | 0 | 0 | 0 | 0 | 0 | 0 | 0 | 0 |
| Smith 2009[176] | 0 | 0 | 0 | 0 | 0 | 0 | 0 | 0 | 0 | 0 | 0 | 0 | 0 | 0 | 0 | 0 | 0 | 1 | 0 | 0 | 0 | 0 | 0 | 0 | 0 | 0 | 0 | 0 | 0 | 0 | 0 | 0 | 0 | 0 | 0 | 0 | 0 | 0 |
| Snel 2011[177] | 0 | 0 | 0 | 0 | 0 | 0 | 0 | 0 | 0 | 0 | 0 | 0 | 0 | 0 | 0 | 0 | 0 | 0 | 0 | 0 | 0 | 0 | 0 | 0 | 0 | 0 | 0 | 0 | 0 | 0 | 0 | 0 | 1 | 0 | 0 | 0 | 0 | 0 |
| Stewart 2010[178] | 0 | 0 | 0 | 0 | 0 | 0 | 0 | 0 | 0 | 0 | 0 | 0 | 0 | 0 | 0 | 0 | 0 | 1 | 0 | 0 | 0 | 0 | 0 | 0 | 0 | 0 | 0 | 0 | 0 | 0 | 0 | 0 | 0 | 0 | 0 | 0 | 0 | 0 |
| Straznicky 2010[179] | 0 | 0 | 0 | 0 | 0 | 0 | 0 | 0 | 0 | 0 | 0 | 0 | 0 | 0 | 0 | 0 | 0 | 0 | 0 | 0 | 0 | 0 | 0 | 0 | 0 | 0 | 0 | 0 | 0 | 0 | 0 | 0 | 1 | 0 | 0 | 0 | 0 | 0 |
| Straznicky 2011[180] | 0 | 0 | 0 | 0 | 0 | 0 | 0 | 0 | 0 | 0 | 0 | 0 | 0 | 0 | 0 | 0 | 0 | 1 | 0 | 0 | 0 | 0 | 0 | 0 | 0 | 0 | 0 | 0 | 0 | 0 | 0 | 0 | 0 | 0 | 0 | 0 | 0 | 0 |
| Streb 2022[181] | 0 | 0 | 0 | 0 | 0 | 0 | 0 | 1 | 0 | 0 | 0 | 0 | 0 | 0 | 0 | 0 | 0 | 0 | 0 | 0 | 0 | 0 | 0 | 0 | 0 | 0 | 0 | 0 | 0 | 0 | 0 | 0 | 0 | 0 | 0 | 0 | 0 | 0 |
| Sturgeon 2023[182] | 0 | 0 | 0 | 0 | 0 | 0 | 1 | 0 | 0 | 0 | 0 | 0 | 0 | 0 | 0 | 0 | 0 | 0 | 0 | 0 | 0 | 0 | 0 | 0 | 0 | 0 | 0 | 0 | 0 | 0 | 0 | 0 | 0 | 0 | 0 | 0 | 0 | 0 |
| Sukala 2012[183] | 0 | 0 | 0 | 0 | 0 | 0 | 0 | 0 | 0 | 0 | 0 | 0 | 0 | 0 | 0 | 0 | 0 | 0 | 1 | 0 | 0 | 0 | 0 | 0 | 0 | 0 | 0 | 0 | 0 | 0 | 0 | 0 | 0 | 0 | 0 | 0 | 0 | 0 |
| Swift 2012[184] | 0 | 0 | 1 | 0 | 0 | 0 | 0 | 0 | 0 | 0 | 0 | 0 | 0 | 0 | 0 | 0 | 0 | 0 | 0 | 0 | 0 | 0 | 0 | 0 | 0 | 0 | 0 | 0 | 0 | 0 | 0 | 0 | 0 | 0 | 0 | 0 | 0 | 0 |
| Tang 2014[185] | 1 | 0 | 0 | 0 | 0 | 0 | 0 | 0 | 0 | 0 | 0 | 0 | 0 | 0 | 0 | 0 | 0 | 0 | 0 | 0 | 0 | 0 | 0 | 0 | 0 | 0 | 0 | 0 | 0 | 0 | 0 | 0 | 0 | 0 | 0 | 0 | 0 | 0 |
| Tartibian 2015[186] | 0 | 0 | 0 | 0 | 0 | 0 | 0 | 0 | 0 | 0 | 0 | 0 | 0 | 0 | 0 | 1 | 0 | 0 | 0 | 0 | 0 | 0 | 0 | 0 | 0 | 0 | 0 | 0 | 0 | 0 | 0 | 0 | 0 | 0 | 0 | 0 | 0 | 0 |
| Thomson 2010[187] | 0 | 0 | 0 | 0 | 0 | 0 | 0 | 0 | 0 | 0 | 0 | 0 | 0 | 0 | 0 | 0 | 0 | 0 | 1 | 0 | 0 | 0 | 0 | 0 | 0 | 0 | 0 | 0 | 0 | 0 | 0 | 0 | 0 | 0 | 0 | 0 | 0 | 0 |
| Tisi 1997[188] | 0 | 0 | 0 | 0 | 0 | 0 | 0 | 0 | 0 | 0 | 0 | 0 | 0 | 1 | 0 | 0 | 0 | 0 | 0 | 0 | 0 | 0 | 0 | 0 | 0 | 0 | 0 | 0 | 0 | 0 | 0 | 0 | 0 | 0 | 0 | 0 | 0 | 0 |
| Tomeleri 2016[189] | 0 | 0 | 0 | 0 | 0 | 0 | 0 | 0 | 0 | 0 | 0 | 0 | 1 | 0 | 0 | 0 | 2 | 0 | 3 | 0 | 0 | 0 | 0 | 0 | 0 | 0 | 0 | 0 | 0 | 0 | 0 | 0 | 0 | 0 | 0 | 0 | 0 | 0 |
| Tomeleri 2018[190] | 0 | 0 | 0 | 0 | 0 | 0 | 0 | 0 | 0 | 0 | 0 | 0 | 0 | 0 | 0 | 0 | 1 | 0 | 2 | 0 | 0 | 0 | 0 | 0 | 0 | 0 | 0 | 0 | 0 | 0 | 0 | 0 | 0 | 0 | 0 | 0 | 0 | 0 |
| Trussardi Fayh 2013[191] | 0 | 0 | 0 | 0 | 0 | 0 | 0 | 0 | 0 | 0 | 0 | 0 | 0 | 0 | 0 | 0 | 0 | 1 | 0 | 0 | 0 | 0 | 0 | 0 | 0 | 0 | 0 | 0 | 0 | 0 | 0 | 0 | 0 | 0 | 0 | 0 | 0 | 0 |
| Urzi 2019[192] | 0 | 0 | 0 | 0 | 0 | 0 | 0 | 0 | 0 | 0 | 0 | 0 | 0 | 0 | 0 | 1 | 0 | 0 | 0 | 0 | 0 | 0 | 0 | 0 | 0 | 0 | 0 | 0 | 0 | 0 | 0 | 0 | 0 | 0 | 0 | 0 | 0 | 0 |
| Varady 2010[193] | 0 | 0 | 0 | 0 | 0 | 0 | 0 | 0 | 0 | 0 | 0 | 0 | 0 | 0 | 0 | 0 | 0 | 0 | 0 | 0 | 0 | 0 | 0 | 0 | 0 | 1 | 0 | 2 | 0 | 0 | 0 | 0 | 0 | 0 | 0 | 0 | 0 | 0 |
| Vasconcellos 2016[194] | 0 | 0 | 0 | 0 | 0 | 0 | 0 | 0 | 1 | 0 | 0 | 0 | 0 | 0 | 0 | 0 | 0 | 0 | 0 | 0 | 0 | 2 | 0 | 0 | 0 | 0 | 0 | 0 | 0 | 0 | 0 | 0 | 0 | 0 | 0 | 0 | 3 | 4 |
| Vella 2017[195] | 0 | 0 | 0 | 0 | 0 | 0 | 0 | 0 | 0 | 0 | 0 | 1 | 0 | 0 | 0 | 0 | 0 | 2 | 0 | 0 | 0 | 0 | 0 | 0 | 0 | 0 | 0 | 0 | 0 | 0 | 0 | 0 | 0 | 0 | 0 | 0 | 0 | 0 |
| Venojärvi 2013[196] | 0 | 0 | 0 | 0 | 0 | 0 | 0 | 0 | 0 | 0 | 0 | 0 | 0 | 0 | 0 | 0 | 0 | 0 | 0 | 0 | 0 | 0 | 0 | 0 | 0 | 1 | 2 | 0 | 0 | 0 | 0 | 0 | 0 | 0 | 0 | 0 | 0 | 0 |

| Primary study | Zalagkitis 1 2025 | Zalagkitis 2 2025 | Wang 2025 | Hernandez-Martinez 2025 | Tan 1 2025 | Tan 2 2025 | Tan 3 2025 | Silva 2024 | Li 2024 | Al-Manna-1 2024 | Al-Manna-2 2024 | Guo 1 2024 | Guo 2 2024 | Malandish 2023 | Dragoumani 2023 | Tan-1 2023 | Tan-2 2023 | Del Rosso-1 | Del Rosso-2 | Del Rosso-3 | Rahimi 2022 | Zhao-1 2022 | Zhao-2 2022 | Zhao-3 2022 | Zhao-4 2022 | Hejazi-1 2022 | Hejazi-2 2022 | Hejazi-3 2022 | Hejazi-4 2022 | Hejazi-5 2022 | Hejazi-6 2022 | Khalafi 2022 | Liu 2021 | Lee 1 2021 | Lee 2 2021 | Khalafi 2020 | Sirico 2018 | Garcia-Hermoso 2016 |
|---|---|---|---|---|---|---|---|---|---|---|---|---|---|---|---|---|---|---|---|---|---|---|---|---|---|---|---|---|---|---|---|---|---|---|---|---|---|---|
| Vieira 2009[197] | 0 | 0 | 0 | 0 | 0 | 0 | 0 | 0 | 0 | 0 | 0 | 0 | 0 | 0 | 0 | 0 | 0 | 1 | 0 | 0 | 0 | 0 | 0 | 0 | 0 | 0 | 0 | 0 | 0 | 0 | 0 | 0 | 0 | 0 | 0 | 0 | 0 | 0 |
| Vos 2011[198] | 0 | 1 | 0 | 0 | 0 | 0 | 0 | 0 | 0 | 0 | 0 | 0 | 0 | 0 | 0 | 0 | 0 | 0 | 0 | 0 | 0 | 0 | 0 | 0 | 0 | 0 | 0 | 0 | 0 | 0 | 0 | 0 | 0 | 0 | 0 | 0 | 0 | 0 |
| Walther 2008[199] | 0 | 0 | 0 | 0 | 0 | 0 | 0 | 0 | 0 | 0 | 0 | 0 | 0 | 1 | 0 | 0 | 0 | 0 | 0 | 0 | 0 | 0 | 0 | 0 | 0 | 0 | 0 | 0 | 0 | 0 | 0 | 0 | 0 | 0 | 0 | 0 | 0 | 0 |
| Wanderley 2013[200] | 0 | 0 | 0 | 0 | 0 | 0 | 0 | 0 | 0 | 0 | 0 | 0 | 0 | 0 | 0 | 0 | 0 | 0 | 1 | 0 | 0 | 0 | 0 | 0 | 0 | 0 | 0 | 0 | 0 | 0 | 0 | 0 | 0 | 0 | 0 | 0 | 0 | 0 |
| Wang 2006[201] | 0 | 0 | 0 | 0 | 0 | 0 | 0 | 0 | 0 | 0 | 0 | 1 | 0 | 0 | 0 | 0 | 0 | 0 | 0 | 0 | 0 | 0 | 0 | 0 | 0 | 0 | 0 | 0 | 0 | 0 | 0 | 0 | 0 | 0 | 0 | 0 | 0 | 0 |
| Wang 2011[202] | 1 | 0 | 0 | 0 | 0 | 0 | 0 | 0 | 0 | 0 | 0 | 0 | 0 | 0 | 0 | 0 | 0 | 0 | 0 | 0 | 0 | 0 | 0 | 0 | 0 | 0 | 0 | 0 | 0 | 0 | 0 | 0 | 0 | 0 | 0 | 0 | 0 | 0 |
| Wang 2018[203] | 0 | 0 | 0 | 0 | 0 | 0 | 0 | 0 | 0 | 0 | 0 | 1 | 0 | 0 | 0 | 0 | 0 | 0 | 0 | 0 | 0 | 0 | 0 | 0 | 0 | 0 | 0 | 0 | 0 | 0 | 0 | 0 | 0 | 0 | 0 | 0 | 0 | 0 |
| Wedell-Neergaard 2019[204] | 0 | 0 | 0 | 0 | 0 | 0 | 0 | 0 | 0 | 0 | 0 | 1 | 0 | 0 | 0 | 0 | 0 | 0 | 0 | 0 | 0 | 0 | 0 | 0 | 0 | 0 | 0 | 0 | 0 | 0 | 0 | 0 | 0 | 0 | 0 | 0 | 0 | 0 |
| Weiss 2016[205] | 0 | 0 | 0 | 0 | 0 | 0 | 0 | 0 | 0 | 0 | 0 | 0 | 0 | 0 | 0 | 0 | 0 | 1 | 0 | 0 | 0 | 0 | 0 | 0 | 0 | 0 | 0 | 0 | 0 | 0 | 0 | 2 | 3 | 0 | 0 | 0 | 0 | 0 |
| Winters-Stone 2018[206] | 0 | 0 | 0 | 0 | 0 | 1 | 0 | 0 | 0 | 0 | 0 | 0 | 0 | 0 | 0 | 0 | 0 | 0 | 0 | 0 | 0 | 0 | 0 | 0 | 0 | 0 | 0 | 0 | 0 | 0 | 0 | 0 | 0 | 0 | 0 | 0 | 0 | 0 |
| Wong 2008[207] | 0 | 0 | 0 | 0 | 0 | 0 | 0 | 0 | 0 | 0 | 0 | 0 | 0 | 0 | 0 | 0 | 0 | 0 | 0 | 0 | 0 | 0 | 0 | 1 | 0 | 0 | 0 | 0 | 0 | 0 | 0 | 0 | 0 | 0 | 0 | 0 | 0 | 2 |
| Wong 2018[208] | 0 | 0 | 0 | 0 | 0 | 0 | 0 | 0 | 1 | 0 | 0 | 0 | 0 | 0 | 2 | 0 | 0 | 0 | 0 | 0 | 0 | 0 | 0 | 3 | 0 | 0 | 0 | 0 | 0 | 0 | 0 | 0 | 0 | 0 | 0 | 0 | 0 | 0 |
| Wycherley 2010[209] | 0 | 0 | 1 | 0 | 0 | 0 | 0 | 0 | 0 | 0 | 0 | 0 | 0 | 0 | 0 | 0 | 0 | 0 | 0 | 0 | 0 | 0 | 0 | 0 | 0 | 0 | 0 | 0 | 0 | 0 | 0 | 0 | 0 | 0 | 0 | 0 | 0 | 0 |
| Yeh 2011[210] | 0 | 0 | 0 | 0 | 0 | 0 | 0 | 0 | 0 | 0 | 0 | 0 | 0 | 1 | 0 | 0 | 0 | 0 | 0 | 0 | 0 | 0 | 0 | 0 | 0 | 0 | 0 | 0 | 0 | 0 | 0 | 0 | 0 | 0 | 0 | 0 | 0 | 0 |
| Yin 2020[211] | 0 | 1 | 0 | 0 | 0 | 0 | 0 | 0 | 0 | 0 | 0 | 0 | 0 | 0 | 0 | 0 | 0 | 0 | 0 | 0 | 0 | 0 | 0 | 0 | 0 | 0 | 0 | 0 | 0 | 0 | 0 | 0 | 0 | 0 | 0 | 0 | 0 | 0 |
| Yoon 2018[212] | 0 | 0 | 0 | 0 | 0 | 0 | 0 | 0 | 0 | 0 | 0 | 0 | 0 | 0 | 0 | 1 | 0 | 0 | 0 | 0 | 0 | 0 | 0 | 0 | 0 | 0 | 0 | 0 | 0 | 0 | 0 | 0 | 0 | 0 | 0 | 0 | 0 | 0 |
| You 2004[213] | 0 | 0 | 0 | 0 | 0 | 0 | 0 | 0 | 0 | 0 | 0 | 1 | 0 | 0 | 0 | 2 | 0 | 0 | 0 | 0 | 0 | 0 | 0 | 0 | 0 | 0 | 0 | 0 | 0 | 0 | 0 | 0 | 3 | 0 | 0 | 0 | 0 | 0 |
| Zhang 2017[214] | 0 | 0 | 0 | 0 | 0 | 0 | 0 | 0 | 0 | 0 | 0 | 0 | 0 | 0 | 0 | 0 | 0 | 0 | 0 | 0 | 0 | 0 | 0 | 0 | 0 | 0 | 0 | 0 | 1 | 0 | 0 | 0 | 0 | 0 | 0 | 0 | 0 | 0 |
| Zhang 2018[215] | 0 | 0 | 0 | 0 | 0 | 0 | 0 | 0 | 0 | 0 | 0 | 0 | 0 | 0 | 0 | 0 | 0 | 1 | 0 | 0 | 0 | 0 | 0 | 0 | 0 | 0 | 0 | 0 | 0 | 0 | 0 | 0 | 0 | 0 | 0 | 0 | 0 | 0 |
| Zhao 2019[216] | 0 | 0 | 0 | 0 | 0 | 0 | 0 | 0 | 0 | 0 | 0 | 1 | 0 | 0 | 0 | 0 | 0 | 0 | 0 | 0 | 0 | 0 | 0 | 0 | 0 | 0 | 0 | 0 | 0 | 0 | 0 | 0 | 0 | 0 | 0 | 0 | 0 | 0 |

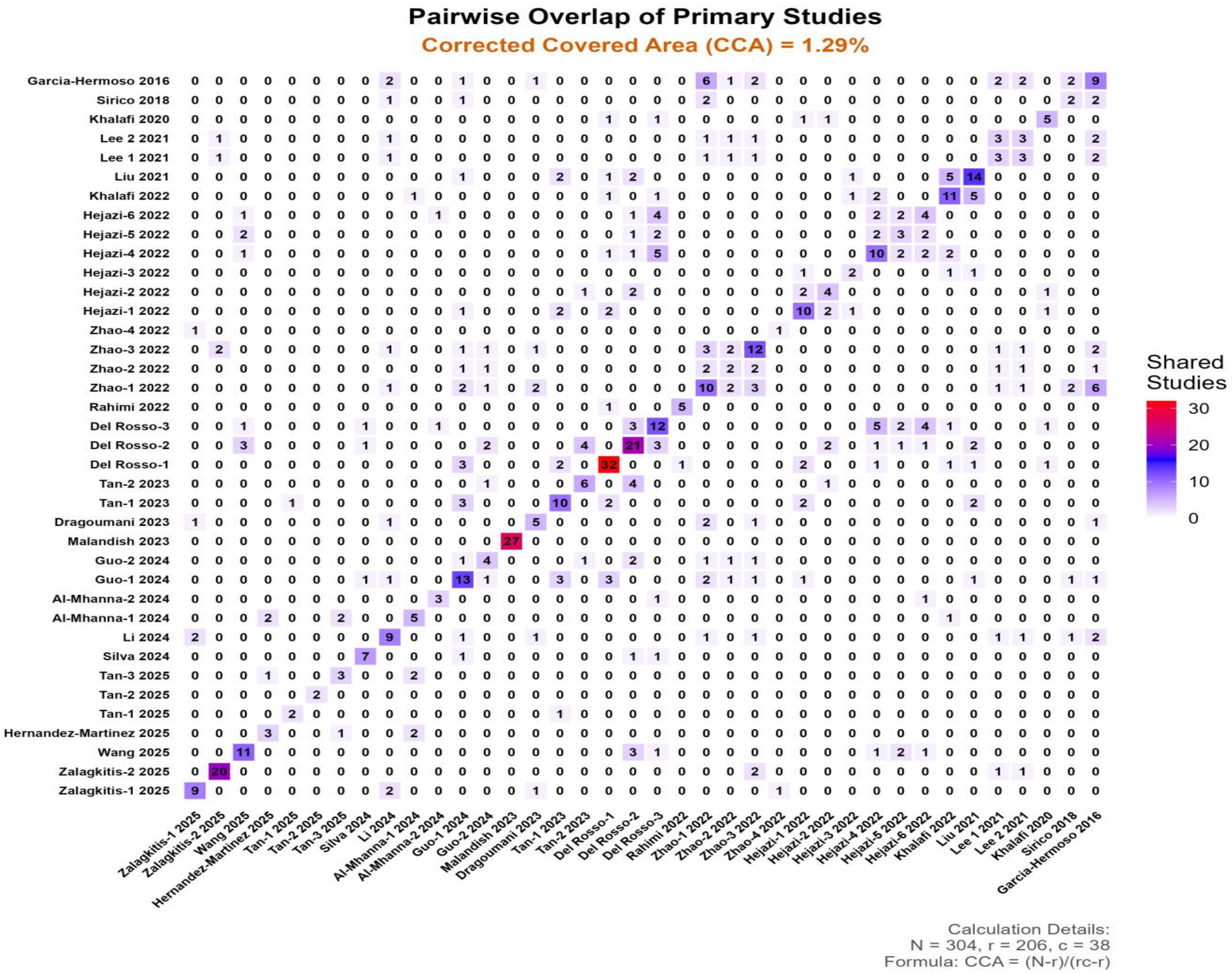


**Supplementary Figure S50. Graphical Overview of the Corrected Covered Area for CRP**

## 7.2 Section 1: For Adiponectin

Supplementary Table S10. Corrected Covered Area Overlap Analysis for Adiponectin

| Primary study | Tan-1(RT) 2025 | Tan-2(CE) 2025 | Silva 2024 | Li 2024 | Al-Mhanna 2024 | Dragoumani 2023 | Tan-1(AE) 2023 | Tan-2(RT) 2023 | DelRosso-1 2023AE | DelRosso-2 2023RT | DelRosso-3 2023AE+RT | Rahimi 2022 | Hejazi-1(AE) 2022 | Hejazi-2(RT) 2022 | Hejazi-3(CE) 2022 | Hejazi-4(HIIT) 2022 | Hejazi-5(AE) 2022 | Hejazi-6(RT) 2022 | Hejazi-7(CE) 2022 | Hejazi-8(HIIT) 2022 | Khalafi 2020 | Sirico 2018 |
|---|---|---|---|---|---|---|---|---|---|---|---|---|---|---|---|---|---|---|---|---|---|---|
| Abbenhardt 2013[217] | 0 | 0 | 0 | 0 | 0 | 0 | 1 | 0 | 2 | 0 | 0 | 0 | 3 | 0 | 0 | 0 | 0 | 0 | 0 | 0 | 0 | 0 |
| Ahmadi 2012[218] | 0 | 0 | 0 | 0 | 0 | 0 | 0 | 0 | 0 | 0 | 0 | 0 | 0 | 0 | 0 | 0 | 1 | 0 | 0 | 0 | 0 | 0 |
| Ahmadizad 2007[219] | 0 | 0 | 0 | 0 | 0 | 0 | 0 | 0 | 0 | 0 | 0 | 0 | 1 | 2 | 0 | 0 | 0 | 0 | 0 | 0 | 0 | 0 |
| Ahmadizad 2014[17] | 0 | 0 | 0 | 0 | 0 | 0 | 0 | 0 | 0 | 1 | 0 | 0 | 0 | 2 | 0 | 0 | 0 | 0 | 0 | 0 | 0 | 0 |
| Akbarpour 2013[18] | 0 | 0 | 0 | 0 | 0 | 0 | 0 | 0 | 1 | 0 | 0 | 0 | 2 | 0 | 0 | 0 | 0 | 0 | 0 | 0 | 0 | 0 |
| Almenning 2015[22] | 0 | 0 | 0 | 0 | 0 | 0 | 0 | 0 | 0 | 0 | 0 | 0 | 0 | 0 | 0 | 0 | 0 | 0 | 0 | 0 | 1 | 0 |
| Annibalini 2017[24] | 0 | 0 | 0 | 0 | 0 | 0 | 0 | 0 | 0 | 0 | 1 | 0 | 0 | 0 | 0 | 0 | 0 | 0 | 1 | 0 | 0 | 0 |
| Arikawa 2011[25] | 0 | 0 | 0 | 0 | 0 | 0 | 0 | 0 | 0 | 0 | 0 | 0 | 1 | 0 | 0 | 0 | 0 | 0 | 0 | 0 | 0 | 0 |
| Armamento-Villareal 2020[220] | 0 | 0 | 0 | 0 | 0 | 0 | 0 | 0 | 0 | 0 | 0 | 0 | 1 | 2 | 3 | 0 | 0 | 0 | 0 | 0 | 0 | 0 |
| Arsenault 2009[26] | 0 | 0 | 0 | 0 | 0 | 0 | 1 | 0 | 2 | 0 | 0 | 0 | 3 | 0 | 0 | 0 | 0 | 0 | 0 | 0 | 0 | 0 |
| Asad 2012[221] | 0 | 0 | 1 | 0 | 0 | 0 | 0 | 0 | 2 | 3 | 4 | 0 | 5 | 6 | 7 | 0 | 0 | 0 | 0 | 0 | 0 | 0 |
| Bagheri 2020[28] | 0 | 0 | 0 | 0 | 0 | 0 | 0 | 0 | 0 | 0 | 0 | 0 | 1 | 0 | 0 | 0 | 0 | 0 | 0 | 0 | 0 | 0 |
| Baigzadeh 2020[222] | 0 | 0 | 0 | 0 | 0 | 0 | 0 | 0 | 0 | 0 | 0 | 0 | 0 | 0 | 0 | 0 | 1 | 0 | 2 | 0 | 0 | 0 |
| Baitul 2017[223] | 0 | 0 | 0 | 0 | 0 | 0 | 1 | 0 | 0 | 0 | 0 | 0 | 0 | 0 | 0 | 0 | 0 | 0 | 0 | 0 | 0 | 0 |
| Balagopal 2005[30] | 0 | 0 | 0 | 0 | 0 | 1 | 0 | 0 | 0 | 0 | 0 | 0 | 0 | 0 | 0 | 0 | 0 | 0 | 0 | 0 | 0 | 0 |
| Balducci 2010a[224] | 0 | 0 | 0 | 0 | 0 | 0 | 0 | 0 | 1 | 0 | 0 | 0 | 0 | 0 | 0 | 0 | 2 | 3 | 4 | 0 | 0 | 0 |
| Balducci 2010b[31] | 0 | 0 | 0 | 0 | 0 | 0 | 0 | 0 | 0 | 0 | 1 | 0 | 0 | 0 | 0 | 0 | 0 | 0 | 0 | 0 | 0 | 0 |
| Beavers 2013[225] | 0 | 0 | 0 | 0 | 0 | 0 | 0 | 0 | 0 | 0 | 0 | 1 | 0 | 0 | 0 | 0 | 0 | 0 | 0 | 0 | 0 | 0 |
| Bouchonville 2014[38] | 0 | 0 | 0 | 0 | 0 | 0 | 0 | 0 | 0 | 0 | 0 | 0 | 0 | 0 | 1 | 0 | 0 | 0 | 0 | 0 | 0 | 0 |
| Boudou 2003[226] | 0 | 0 | 0 | 0 | 0 | 0 | 0 | 0 | 0 | 0 | 0 | 0 | 0 | 0 | 0 | 0 | 0 | 0 | 0 | 1 | 0 | 0 |
| Bouri 2015[227] | 0 | 0 | 0 | 0 | 0 | 0 | 0 | 0 | 0 | 0 | 0 | 0 | 0 | 0 | 0 | 1 | 0 | 0 | 0 | 0 | 0 | 0 |
| Brooks 2007[41] | 0 | 0 | 0 | 0 | 0 | 0 | 0 | 0 | 0 | 0 | 0 | 0 | 0 | 0 | 0 | 0 | 0 | 1 | 0 | 0 | 0 | 0 |
| Brunelli 2015[42] | 0 | 0 | 1 | 0 | 0 | 0 | 0 | 0 | 0 | 0 | 0 | 0 | 0 | 0 | 0 | 0 | 0 | 0 | 0 | 0 | 0 | 0 |
| Corpeleijn 2007[228] | 0 | 0 | 0 | 0 | 0 | 0 | 0 | 0 | 0 | 0 | 0 | 0 | 0 | 0 | 0 | 0 | 1 | 0 | 0 | 0 | 0 | 0 |
| Christiansen 2010[50] | 0 | 0 | 0 | 0 | 0 | 0 | 0 | 0 | 1 | 0 | 0 | 0 | 0 | 0 | 0 | 0 | 0 | 0 | 0 | 0 | 0 | 0 |
| De Souza 2022[229] | 0 | 0 | 0 | 1 | 0 | 0 | 0 | 0 | 0 | 0 | 0 | 0 | 0 | 0 | 0 | 0 | 0 | 0 | 0 | 0 | 0 | 0 |
| Dieli-Conwright 2018a[59] | 0 | 1 | 0 | 0 | 2 | 0 | 0 | 0 | 0 | 0 | 0 | 0 | 0 | 0 | 0 | 0 | 0 | 0 | 0 | 0 | 0 | 0 |
| Dieli-Conwright 2018b[230] | 0 | 1 | 0 | 0 | 0 | 0 | 0 | 0 | 0 | 0 | 0 | 0 | 0 | 0 | 0 | 0 | 0 | 0 | 0 | 0 | 0 | 0 |
| Dogan Dede 2014[231] | 0 | 0 | 0 | 0 | 0 | 0 | 0 | 0 | 0 | 0 | 0 | 0 | 0 | 0 | 0 | 0 | 1 | 0 | 0 | 0 | 0 | 0 |
| Desde 2015[232] | 0 | 0 | 0 | 0 | 0 | 0 | 0 | 0 | 1 | 0 | 0 | 0 | 0 | 0 | 0 | 0 | 0 | 0 | 0 | 0 | 0 | 0 |
| Eizadi 2011[233] | 0 | 0 | 0 | 0 | 0 | 0 | 0 | 0 | 0 | 0 | 0 | 0 | 1 | 0 | 0 | 0 | 0 | 0 | 0 | 0 | 0 | 0 |
| Fatouros 2005[234] | 0 | 0 | 0 | 0 | 0 | 0 | 0 | 0 | 0 | 1 | 0 | 0 | 0 | 2 | 0 | 0 | 0 | 0 | 0 | 0 | 0 | 0 |
| Fazelifar 2013[235] | 0 | 0 | 0 | 0 | 0 | 0 | 0 | 0 | 0 | 0 | 0 | 0 | 0 | 0 | 0 | 0 | 0 | 0 | 0 | 0 | 0 | 1 |
| Figueroa 2013[236] | 0 | 0 | 0 | 0 | 0 | 0 | 0 | 1 | 0 | 2 | 0 | 0 | 0 | 0 | 0 | 0 | 0 | 0 | 0 | 0 | 0 | 0 |
| Friedenreich 2011[237] | 0 | 0 | 0 | 0 | 0 | 0 | 0 | 0 | 0 | 0 | 0 | 0 | 1 | 0 | 0 | 0 | 0 | 0 | 0 | 0 | 0 | 0 |
| Gokulakrishnan 2017[74] | 0 | 0 | 0 | 0 | 0 | 0 | 0 | 0 | 0 | 0 | 0 | 0 | 0 | 0 | 0 | 0 | 1 | 0 | 0 | 0 | 0 | 0 |
| Hara 2005[238] | 0 | 0 | 1 | 0 | 0 | 0 | 0 | 0 | 2 | 0 | 3 | 0 | 4 | 0 | 5 | 0 | 0 | 0 | 0 | 0 | 0 | 0 |
| Ibáñez 2010[239] | 0 | 0 | 0 | 0 | 0 | 0 | 0 | 0 | 0 | 1 | 0 | 0 | 0 | 0 | 0 | 0 | 0 | 2 | 0 | 0 | 0 | 0 |
| Jeon 2013[137] | 0 | 0 | 0 | 1 | 0 | 0 | 0 | 0 | 0 | 0 | 0 | 0 | 0 | 0 | 0 | 0 | 0 | 0 | 0 | 0 | 0 | 0 |

| Primary study | Tan-1(RT) 2025 | Tan-2(CE) 2025 | Silva 2024 | Li 2024 | Al-Mhanna 2024 | Dragoumani 2023 | Tan-1(AE) 2023 | Tan-2(RT) 2023 | DelRosso-1 2023AE | DelRosso-2 2023RT | DelRosso-3 2023AE+RT | Rahimi 2022 | Hejazi-1(AE) 2022 | Hejazi-2(RT) 2022 | Hejazi-3(CE) 2022 | Hejazi-4(HIIT) 2022 | Hejazi-5(AE) 2022 | Hejazi-6(RT) 2022 | Hejazi-7(CE) 2022 | Hejazi-8(HIIT) 2022 | Khalafi 2020 | Sirico 2018 |
|---|---|---|---|---|---|---|---|---|---|---|---|---|---|---|---|---|---|---|---|---|---|---|
| Johannsen 2012[80] | 0 | 0 | 0 | 0 | 0 | 0 | 1 | 0 | 0 | 0 | 0 | 0 | 2 | 0 | 0 | 0 | 0 | 0 | 0 | 0 | 0 | 0 |
| Jorge 2011[82] | 0 | 0 | 0 | 0 | 0 | 0 | 0 | 0 | 1 | 2 | 3 | 0 | 0 | 0 | 0 | 0 | 4 | 5 | 6 | 0 | 0 | 0 |
| Kadoglou 2007a[83] | 0 | 0 | 0 | 0 | 0 | 0 | 0 | 0 | 1 | 0 | 0 | 0 | 0 | 0 | 0 | 0 | 2 | 0 | 0 | 0 | 0 | 0 |
| Kadoglou 2007b[240] | 0 | 0 | 0 | 0 | 0 | 0 | 0 | 0 | 0 | 0 | 0 | 0 | 0 | 0 | 0 | 0 | 1 | 0 | 0 | 0 | 0 | 0 |
| Kahhan 2021[241] | 0 | 0 | 0 | 0 | 0 | 1 | 0 | 0 | 0 | 0 | 0 | 0 | 0 | 0 | 0 | 0 | 0 | 0 | 0 | 0 | 0 | 0 |
| Kelly 2007[89] | 0 | 0 | 0 | 1 | 0 | 0 | 0 | 0 | 0 | 0 | 0 | 0 | 0 | 0 | 0 | 0 | 0 | 0 | 0 | 0 | 0 | 0 |
| Kim 2007[91] | 0 | 0 | 0 | 0 | 0 | 0 | 0 | 0 | 0 | 0 | 0 | 0 | 0 | 0 | 0 | 0 | 0 | 0 | 0 | 0 | 0 | 1 |
| Kim 2015[242] | 0 | 0 | 0 | 0 | 0 | 0 | 0 | 0 | 1 | 0 | 0 | 0 | 2 | 0 | 0 | 0 | 0 | 0 | 0 | 0 | 0 | 0 |
| Kim 2017[243] | 0 | 1 | 0 | 0 | 0 | 0 | 0 | 0 | 0 | 0 | 0 | 0 | 0 | 0 | 0 | 0 | 0 | 0 | 0 | 0 | 0 | 0 |
| Koh 2017[95] | 0 | 0 | 0 | 0 | 0 | 0 | 0 | 0 | 0 | 0 | 0 | 0 | 2 | 0 | 0 | 0 | 0 | 0 | 0 | 0 | 0 | 0 |
| Kondo 2006[97] | 0 | 0 | 0 | 0 | 0 | 0 | 0 | 0 | 0 | 0 | 0 | 0 | 1 | 0 | 0 | 0 | 0 | 0 | 0 | 0 | 0 | 0 |
| Konopko-Zubrzycka 2009[244] | 0 | 0 | 0 | 0 | 0 | 0 | 0 | 0 | 0 | 0 | 0 | 0 | 1 | 0 | 0 | 0 | 0 | 0 | 0 | 0 | 0 | 0 |
| Kortas 2020[245] | 0 | 0 | 0 | 0 | 0 | 0 | 1 | 0 | 0 | 0 | 0 | 0 | 2 | 0 | 0 | 0 | 0 | 0 | 0 | 0 | 0 | 0 |
| Ku 2010[246] | 0 | 0 | 0 | 0 | 0 | 0 | 0 | 1 | 0 | 0 | 0 | 0 | 0 | 0 | 0 | 0 | 2 | 3 | 0 | 0 | 0 | 0 |
| Lee 2012[101] | 0 | 0 | 0 | 0 | 0 | 0 | 1 | 0 | 0 | 0 | 0 | 0 | 0 | 0 | 0 | 0 | 0 | 0 | 0 | 0 | 0 | 0 |
| Ligibel 2008[247] | 0 | 0 | 0 | 0 | 1 | 0 | 0 | 0 | 0 | 0 | 0 | 0 | 0 | 0 | 0 | 0 | 0 | 0 | 0 | 0 | 0 | 0 |
| Ligibel 2009[248] | 0 | 1 | 0 | 0 | 0 | 0 | 0 | 0 | 0 | 0 | 0 | 0 | 0 | 0 | 0 | 0 | 0 | 0 | 0 | 0 | 0 | 0 |
| Ligibel 2019[103] | 0 | 1 | 0 | 0 | 0 | 0 | 0 | 0 | 0 | 0 | 0 | 0 | 0 | 0 | 0 | 0 | 0 | 0 | 0 | 0 | 0 | 0 |
| Lin 2023[249] | 0 | 1 | 0 | 0 | 0 | 0 | 0 | 0 | 0 | 0 | 0 | 0 | 0 | 0 | 0 | 0 | 0 | 0 | 0 | 0 | 0 | 0 |
| Liu 2018[105] | 0 | 0 | 0 | 1 | 0 | 0 | 0 | 0 | 0 | 0 | 0 | 0 | 0 | 0 | 0 | 0 | 0 | 0 | 0 | 0 | 0 | 0 |
| Mendez-Gutierrez 2022[250] | 0 | 0 | 1 | 0 | 0 | 0 | 0 | 0 | 0 | 0 | 0 | 0 | 0 | 0 | 0 | 0 | 0 | 0 | 0 | 0 | 0 | 0 |
| Mendham 2014a[251] | 0 | 0 | 0 | 0 | 0 | 0 | 0 | 0 | 1 | 0 | 0 | 0 | 0 | 0 | 0 | 0 | 1 | 0 | 0 | 0 | 0 | 0 |
| Mendham 2014b[119] | 0 | 0 | 0 | 0 | 0 | 0 | 0 | 0 | 0 | 0 | 1 | 0 | 0 | 0 | 0 | 0 | 0 | 0 | 0 | 0 | 0 | 0 |
| Mietus-Snyder 2020[121] | 0 | 0 | 0 | 0 | 0 | 1 | 0 | 0 | 0 | 0 | 0 | 0 | 0 | 0 | 0 | 0 | 0 | 0 | 0 | 0 | 0 | 0 |
| Moghadasi 2011[124] | 0 | 0 | 0 | 0 | 0 | 0 | 0 | 0 | 0 | 0 | 0 | 0 | 0 | 0 | 0 | 0 | 1 | 0 | 0 | 0 | 0 | 0 |
| Moghadasi 2012[123] | 0 | 0 | 0 | 0 | 0 | 0 | 0 | 0 | 1 | 0 | 0 | 0 | 0 | 0 | 0 | 0 | 0 | 0 | 0 | 1 | 0 | 0 |
| Moradi 2015[252] | 0 | 0 | 0 | 0 | 0 | 0 | 0 | 0 | 0 | 0 | 0 | 0 | 0 | 1 | 0 | 0 | 0 | 0 | 0 | 0 | 0 | 0 |
| Murphy 2009[128] | 0 | 0 | 0 | 1 | 0 | 0 | 0 | 0 | 0 | 0 | 0 | 0 | 0 | 0 | 0 | 0 | 0 | 0 | 0 | 0 | 0 | 0 |
| Marcell 2005[111] | 0 | 0 | 0 | 0 | 0 | 0 | 0 | 0 | 1 | 0 | 0 | 0 | 0 | 0 | 0 | 0 | 0 | 0 | 0 | 0 | 0 | 0 |
| Nambi 2022[253] | 0 | 0 | 0 | 1 | 0 | 0 | 0 | 0 | 0 | 0 | 0 | 0 | 0 | 0 | 0 | 0 | 0 | 0 | 0 | 0 | 0 | 0 |
| Nikseresht 2014[254] | 0 | 0 | 0 | 0 | 0 | 0 | 0 | 0 | 0 | 0 | 0 | 0 | 1 | 2 | 0 | 0 | 0 | 0 | 0 | 0 | 3 | 0 |
| Nono 2020[135] | 0 | 0 | 0 | 0 | 0 | 0 | 0 | 0 | 0 | 0 | 1 | 0 | 0 | 0 | 0 | 0 | 0 | 0 | 0 | 0 | 0 | 0 |
| Oh 2013[137] | 0 | 0 | 0 | 0 | 0 | 0 | 0 | 0 | 0 | 0 | 0 | 1 | 0 | 0 | 0 | 0 | 0 | 0 | 0 | 0 | 0 | 0 |
| Okada 2010[139] | 0 | 0 | 0 | 0 | 0 | 0 | 0 | 0 | 0 | 0 | 1 | 0 | 0 | 0 | 0 | 0 | 0 | 0 | 3 | 0 | 0 | 0 |
| Olson 2007[140] | 0 | 0 | 0 | 0 | 0 | 0 | 0 | 0 | 0 | 1 | 0 | 0 | 0 | 2 | 0 | 0 | 0 | 0 | 0 | 0 | 0 | 0 |
| Park 2007[141] | 0 | 0 | 0 | 0 | 0 | 1 | 0 | 0 | 0 | 0 | 0 | 0 | 0 | 0 | 0 | 0 | 0 | 0 | 0 | 0 | 0 | 0 |
| Parsian 2012[255] | 0 | 0 | 0 | 0 | 0 | 0 | 0 | 0 | 0 | 0 | 0 | 0 | 0 | 0 | 0 | 0 | 1 | 0 | 0 | 0 | 0 | 0 |
| Pedrosa 2011[256] | 0 | 0 | 0 | 0 | 0 | 1 | 0 | 0 | 0 | 0 | 0 | 0 | 0 | 0 | 0 | 0 | 0 | 0 | 0 | 0 | 0 | 0 |
| Phillips 2012[147] | 0 | 0 | 0 | 0 | 0 | 0 | 0 | 1 | 0 | 2 | 0 | 0 | 0 | 3 | 0 | 0 | 0 | 0 | 0 | 0 | 0 | 0 |
| Pourvaghar 2017[257] | 0 | 0 | 0 | 0 | 0 | 0 | 0 | 0 | 0 | 0 | 0 | 0 | 0 | 0 | 0 | 1 | 0 | 0 | 0 | 0 | 0 | 0 |
| Pasqualini 2010[258] | 0 | 0 | 0 | 0 | 0 | 0 | 0 | 0 | 1 | 0 | 0 | 0 | 0 | 0 | 0 | 0 | 0 | 0 | 0 | 0 | 0 | 0 |
| Racil 2013[259] | 0 | 0 | 0 | 1 | 0 | 0 | 0 | 0 | 0 | 0 | 0 | 0 | 0 | 0 | 0 | 0 | 0 | 0 | 0 | 0 | 2 | 0 |

| Primary study | Tan-1(RT) 2025 | Tan-2(CE) 2025 | Silva 2024 | Li 2024 | Al-Mhanna 2024 | Dragoumani 2023 | Tan-1(AE) 2023 | Tan-2(RT) 2023 | DelRosso-1 2023AE | DelRosso-2 2023RT | DelRosso-3 2023AE+RT | Rahimi 2022 | Hejazi-1(AE) 2022 | Hejazi-2(RT) 2022 | Hejazi-3(CE) 2022 | Hejazi-4(HIIT) 2022 | Hejazi-5(AE) 2022 | Hejazi-6(RT) 2022 | Hejazi-7(CE) 2022 | Hejazi-8(HIIT) 2022 | Khalafi 2020 | Sirico 2018 |
|---|---|---|---|---|---|---|---|---|---|---|---|---|---|---|---|---|---|---|---|---|---|---|
| Racil 2016[260] | 0 | 0 | 0 | 1 | 0 | 0 | 0 | 0 | 0 | 0 | 0 | 0 | 0 | 0 | 0 | 0 | 0 | 0 | 0 | 0 | 2 | 3 |
| Reljic 2022[160] | 0 | 0 | 0 | 0 | 0 | 0 | 0 | 0 | 1 | 2 | 0 | 0 | 0 | 0 | 0 | 0 | 0 | 0 | 0 | 0 | 0 | 0 |
| Rahimi 2021[261] | 0 | 0 | 0 | 0 | 0 | 0 | 0 | 0 | 0 | 0 | 0 | 0 | 1 | 2 | 3 | 0 | 0 | 0 | 0 | 0 | 0 | 0 |
| Rashidlamir 2012[262] | 0 | 0 | 0 | 0 | 0 | 0 | 0 | 0 | 0 | 0 | 0 | 0 | 1 | 0 | 0 | 0 | 0 | 0 | 0 | 0 | 0 | 0 |
| Rokling-Andersen 2007[165] | 0 | 0 | 0 | 0 | 0 | 0 | 0 | 0 | 0 | 0 | 1 | 0 | 0 | 0 | 0 | 0 | 2 | 0 | 0 | 0 | 0 | 0 |
| Saeidi 2019[263] | 0 | 0 | 0 | 0 | 0 | 0 | 0 | 1 | 0 | 0 | 0 | 0 | 0 | 0 | 0 | 0 | 0 | 0 | 0 | 0 | 0 | 0 |
| Saghebjoo 2019[264] | 0 | 0 | 0 | 0 | 0 | 0 | 0 | 0 | 0 | 0 | 0 | 0 | 0 | 0 | 0 | 1 | 0 | 0 | 0 | 0 | 2 | 0 |
| Shakeri 2016[265] | 0 | 0 | 0 | 0 | 0 | 0 | 0 | 0 | 0 | 0 | 0 | 0 | 1 | 0 | 0 | 0 | 0 | 0 | 0 | 0 | 0 | 0 |
| Sixt 2010[266] | 0 | 0 | 0 | 0 | 0 | 0 | 0 | 0 | 0 | 0 | 0 | 0 | 0 | 0 | 0 | 0 | 1 | 0 | 0 | 0 | 0 | 0 |
| Sukala 2012[183] | 0 | 0 | 0 | 0 | 0 | 0 | 0 | 0 | 1 | 2 | 0 | 0 | 0 | 0 | 0 | 0 | 0 | 0 | 0 | 0 | 0 | 0 |
| Sokolovska 2020[267] | 0 | 0 | 0 | 0 | 0 | 0 | 0 | 0 | 0 | 0 | 0 | 0 | 0 | 0 | 0 | 0 | 0 | 0 | 0 | 1 | 0 | 0 |
| Taha 2013[268] | 0 | 0 | 0 | 0 | 0 | 0 | 0 | 0 | 0 | 0 | 0 | 0 | 0 | 1 | 0 | 0 | 0 | 0 | 0 | 0 | 0 | 0 |
| Tjønna 2008[269] | 0 | 0 | 0 | 0 | 0 | 0 | 0 | 0 | 0 | 0 | 0 | 0 | 1 | 0 | 0 | 2 | 0 | 0 | 0 | 0 | 0 | 0 |
| Varady 2010[193] | 0 | 0 | 0 | 0 | 0 | 0 | 0 | 0 | 0 | 0 | 0 | 0 | 1 | 0 | 2 | 0 | 0 | 0 | 0 | 0 | 0 | 0 |
| Vasconcellos 2016[194] | 0 | 0 | 0 | 1 | 0 | 0 | 0 | 0 | 0 | 0 | 0 | 0 | 0 | 0 | 0 | 0 | 0 | 0 | 0 | 0 | 0 | 2 |
| Venojärvi 2013[196] | 0 | 0 | 0 | 0 | 0 | 0 | 0 | 0 | 0 | 0 | 0 | 0 | 1 | 2 | 0 | 0 | 0 | 0 | 0 | 0 | 0 | 0 |
| Vos 2011[198] | 0 | 0 | 0 | 0 | 0 | 1 | 0 | 0 | 0 | 0 | 0 | 0 | 0 | 0 | 0 | 0 | 0 | 0 | 0 | 0 | 0 | 0 |
| Vella 2017[195] | 0 | 0 | 0 | 0 | 0 | 0 | 0 | 0 | 1 | 0 | 0 | 0 | 0 | 0 | 0 | 0 | 0 | 0 | 0 | 0 | 0 | 0 |
| Wang 2015[270] | 0 | 0 | 0 | 0 | 0 | 0 | 1 | 0 | 2 | 0 | 0 | 0 | 0 | 0 | 0 | 0 | 0 | 0 | 0 | 0 | 0 | 0 |
| Winters-Stone 2018[206] | 1 | 0 | 0 | 0 | 0 | 0 | 0 | 0 | 0 | 0 | 0 | 0 | 0 | 0 | 0 | 0 | 0 | 0 | 0 | 0 | 0 | 0 |
| Wong 2018[207] | 0 | 0 | 0 | 1 | 0 | 2 | 0 | 0 | 0 | 0 | 0 | 0 | 0 | 0 | 0 | 0 | 0 | 0 | 0 | 0 | 0 | 0 |
| Wu 2011[271] | 0 | 0 | 0 | 0 | 0 | 0 | 0 | 0 | 1 | 0 | 0 | 0 | 0 | 0 | 0 | 0 | 2 | 0 | 0 | 0 | 0 | 0 |
| Yang 2020[272] | 0 | 0 | 0 | 0 | 0 | 0 | 0 | 0 | 0 | 0 | 0 | 0 | 0 | 0 | 0 | 0 | 1 | 0 | 0 | 0 | 0 | 0 |
| Zhang 2017[214] | 0 | 0 | 0 | 0 | 0 | 0 | 0 | 0 | 0 | 0 | 0 | 0 | 0 | 0 | 0 | 0 | 1 | 0 | 0 | 0 | 0 | 0 |
| Zhang 2018[215] | 0 | 0 | 0 | 0 | 0 | 0 | 0 | 0 | 1 | 0 | 0 | 0 | 0 | 0 | 0 | 0 | 0 | 0 | 0 | 0 | 0 | 0 |

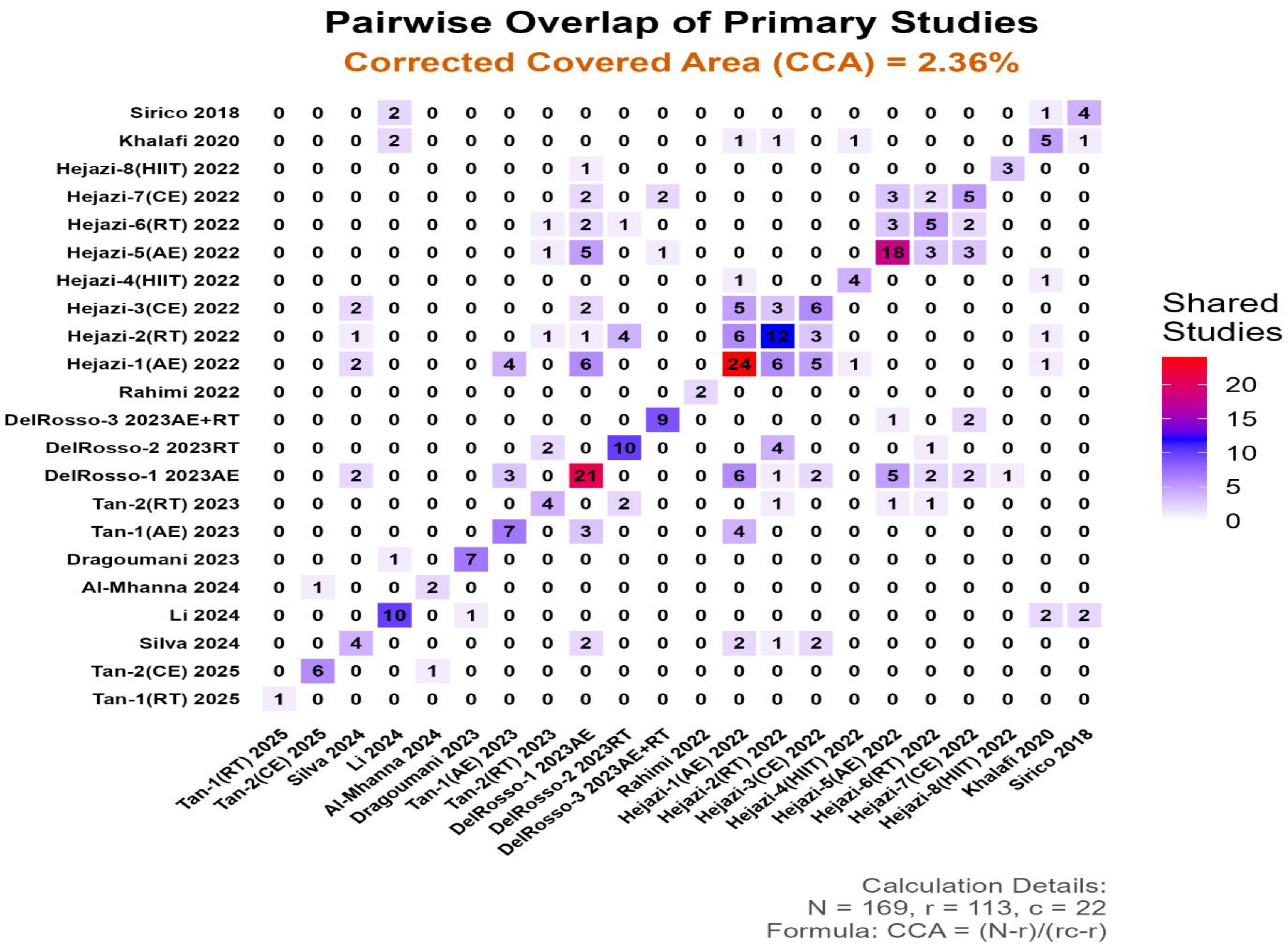


**Supplementary Figure S52. Graphical Overview of the Corrected Covered Area for Adiponectin**

### 7.3 Section 1: For Leptin

Supplementary Table S11. Corrected Covered Area Overlap Analysis for Leptin

| Primary study | Hernandez-Martinez 2025 | Tan-1(RT) 2025 | Tan-2(CE) 2025 | Silva 2024 | Li 2024 | Al-Mhanna 2024 | Dragoumani 2023 | DelRosso-1 2023AE | DelRosso-2 2023RT | DelRosso-3 2023AE+RT | Hejazi-1(AE) 2022 | Hejazi-2(RT) 2022 | Hejazi-3(AE) 2022 | Hejazi-4(RT) 2022 | Hejazi-5(CE) 2022 | Hejazi-6(HIIT) 2022 | Khalafi 2020 | Sirico 2018 |
|---|---|---|---|---|---|---|---|---|---|---|---|---|---|---|---|---|---|---|
| Ahmadizad 2014[17] | 0 | 0 | 0 | 0 | 0 | 0 | 0 | 0 | 1 | 0 | 0 | 2 | 0 | 0 | 0 | 0 | 0 | 0 |
| Abbenhardt 2013[217] | 0 | 0 | 0 | 0 | 0 | 0 | 0 | 1 | 0 | 0 | 0 | 0 | 0 | 0 | 0 | 0 | 0 | 0 |
| Akbarpour 2013[18] | 0 | 0 | 0 | 0 | 0 | 0 | 0 | 1 | 0 | 0 | 2 | 0 | 0 | 0 | 0 | 0 | 0 | 0 |
| Almenning 2015[22] | 0 | 0 | 0 | 0 | 0 | 0 | 0 | 0 | 0 | 0 | 0 | 0 | 0 | 0 | 0 | 0 | 1 | 0 |
| Amaro-Gahete 2021[23] | 0 | 0 | 0 | 1 | 0 | 0 | 0 | 0 | 0 | 0 | 0 | 0 | 0 | 0 | 0 | 0 | 0 | 0 |
| Annibalini 2017[24] | 0 | 0 | 0 | 0 | 0 | 0 | 0 | 0 | 0 | 1 | 0 | 0 | 0 | 0 | 2 | 0 | 0 | 0 |
| Arikawa 2011[25] | 0 | 0 | 0 | 0 | 0 | 0 | 0 | 0 | 0 | 0 | 1 | 0 | 0 | 0 | 0 | 0 | 0 | 0 |
| Armamento-Villareal 2020[220] | 0 | 0 | 0 | 0 | 0 | 0 | 0 | 0 | 0 | 0 | 1 | 0 | 0 | 0 | 0 | 0 | 0 | 0 |
| Balducci 2010a[31] | 0 | 0 | 0 | 0 | 0 | 0 | 0 | 1 | 0 | 0 | 0 | 0 | 2 | 3 | 4 | 0 | 0 | 0 |
| Balducci 2010b[224] | 0 | 0 | 0 | 0 | 0 | 0 | 0 | 0 | 0 | 1 | 0 | 0 | 0 | 0 | 0 | 0 | 0 | 0 |
| Boudou 2003[226] | 0 | 0 | 0 | 0 | 0 | 0 | 0 | 0 | 0 | 0 | 0 | 0 | 1 | 0 | 0 | 0 | 0 | 0 |
| Brunelli 2015[42] | 0 | 0 | 0 | 1 | 0 | 0 | 0 | 0 | 0 | 0 | 0 | 0 | 0 | 0 | 0 | 0 | 0 | 0 |
| Corpeleijn 2007[228] | 0 | 0 | 0 | 0 | 0 | 0 | 0 | 0 | 0 | 0 | 0 | 0 | 1 | 0 | 0 | 0 | 0 | 0 |
| Dieli-Conwright 2018a[59] | 1 | 0 | 2 | 0 | 0 | 3 | 0 | 0 | 0 | 0 | 0 | 0 | 0 | 0 | 0 | 0 | 0 | 0 |
| Dieli-Conwright 2018b[230] | 0 | 0 | 1 | 0 | 0 | 0 | 0 | 0 | 0 | 0 | 0 | 0 | 0 | 0 | 0 | 0 | 0 | 0 |
| Dogan Dede 2014[231] | 0 | 0 | 0 | 0 | 0 | 0 | 0 | 0 | 0 | 0 | 0 | 0 | 1 | 0 | 0 | 0 | 0 | 0 |
| Dede 2015[232] | 0 | 0 | 0 | 0 | 0 | 0 | 0 | 1 | 0 | 0 | 0 | 0 | 0 | 0 | 0 | 0 | 0 | 0 |
| Fatouros 2005[234] | 0 | 0 | 0 | 0 | 0 | 0 | 0 | 0 | 1 | 0 | 0 | 2 | 0 | 0 | 0 | 0 | 0 | 0 |
| Frank 2005[273] | 0 | 0 | 0 | 0 | 0 | 0 | 0 | 1 | 0 | 0 | 0 | 0 | 0 | 0 | 0 | 0 | 0 | 0 |
| Fazelifar 2013[235] | 0 | 0 | 0 | 0 | 0 | 0 | 0 | 0 | 0 | 0 | 0 | 0 | 0 | 0 | 0 | 0 | 0 | 1 |
| Figueroa 2013[236] | 0 | 0 | 0 | 0 | 0 | 0 | 0 | 0 | 1 | 0 | 0 | 0 | 0 | 0 | 0 | 0 | 0 | 0 |
| Fedewa 2018[64] | 1 | 0 | 0 | 0 | 0 | 0 | 0 | 0 | 0 | 0 | 0 | 0 | 0 | 0 | 0 | 0 | 0 | 0 |
| Friedenreich 2011[237] | 0 | 0 | 0 | 0 | 0 | 0 | 0 | 0 | 0 | 0 | 1 | 0 | 0 | 0 | 0 | 0 | 0 | 0 |
| Gokulakrishnan 2017[74] | 0 | 0 | 0 | 0 | 0 | 0 | 0 | 0 | 0 | 0 | 0 | 0 | 1 | 0 | 0 | 0 | 0 | 0 |
| Hara 2005[238] | 0 | 0 | 0 | 1 | 0 | 0 | 0 | 2 | 0 | 3 | 4 | 0 | 0 | 0 | 0 | 0 | 0 | 0 |
| Ibáñez 2010[239] | 0 | 0 | 0 | 0 | 0 | 0 | 0 | 0 | 1 | 0 | 0 | 0 | 0 | 1 | 0 | 0 | 0 | 0 |
| Kahhan 2021[241] | 0 | 0 | 0 | 0 | 0 | 0 | 1 | 0 | 0 | 0 | 0 | 0 | 0 | 0 | 0 | 0 | 0 | 0 |
| Karacabey 2009[274] | 0 | 0 | 0 | 0 | 0 | 0 | 0 | 0 | 0 | 0 | 0 | 0 | 0 | 0 | 0 | 0 | 0 | 1 |
| Kelly 2007[89] | 0 | 0 | 0 | 0 | 1 | 0 | 0 | 0 | 0 | 0 | 0 | 0 | 0 | 0 | 0 | 0 | 0 | 0 |
| Kim 2015[242] | 0 | 0 | 0 | 0 | 0 | 0 | 0 | 1 | 0 | 0 | 2 | 0 | 0 | 0 | 0 | 0 | 0 | 0 |
| Kim 2016[94] | 1 | 0 | 0 | 0 | 0 | 0 | 0 | 0 | 0 | 0 | 0 | 0 | 0 | 0 | 0 | 0 | 0 | 0 |
| Kim 2017[243] | 0 | 0 | 1 | 0 | 0 | 0 | 0 | 0 | 0 | 0 | 0 | 0 | 0 | 0 | 0 | 0 | 0 | 0 |
| Kim 2019[275] | 0 | 0 | 0 | 0 | 0 | 0 | 0 | 0 | 0 | 1 | 0 | 0 | 0 | 0 | 0 | 0 | 0 | 0 |
| Kondo 2006[97] | 0 | 0 | 0 | 0 | 0 | 0 | 0 | 0 | 0 | 0 | 1 | 0 | 0 | 0 | 0 | 0 | 0 | 0 |
| Konopko-Zubrzycka 2009[244] | 0 | 0 | 0 | 0 | 0 | 0 | 0 | 0 | 0 | 0 | 1 | 0 | 0 | 0 | 0 | 0 | 0 | 0 |
| Ku 2010[246] | 0 | 0 | 0 | 0 | 0 | 0 | 0 | 0 | 0 | 0 | 0 | 0 | 1 | 2 | 0 | 0 | 0 | 0 |
| Ligibel 2008[247] | 0 | 0 | 0 | 0 | 0 | 1 | 0 | 0 | 0 | 0 | 0 | 0 | 0 | 0 | 0 | 0 | 0 | 0 |
| Ligibel 2019[103] | 0 | 0 | 1 | 0 | 0 | 0 | 0 | 0 | 0 | 0 | 0 | 0 | 0 | 0 | 0 | 0 | 0 | 0 |
| Lin 2023[249] | 0 | 0 | 1 | 0 | 0 | 0 | 0 | 0 | 0 | 0 | 0 | 0 | 0 | 0 | 0 | 0 | 0 | 0 |
| Liu 2018[105] | 0 | 0 | 0 | 0 | 1 | 0 | 0 | 0 | 0 | 0 | 0 | 0 | 0 | 0 | 0 | 0 | 0 | 0 |

| Primary study | Hernandez-Martinez 2025 | Tan-1(RT) 2025 | Tan-2(CE) 2025 | Silva 2024 | Li 2024 | Al-Mhanna 2024 | Dragoumani 2023 | DelRosso-1 2023AE | DelRosso-2 2023RT | DelRosso-3 2023AE+RT | Hejazi-1(AE) 2022 | Hejazi-2(RT) 2022 | Hejazi-3(AE) 2022 | Hejazi-4(RT) 2022 | Hejazi-5(CE) 2022 | Hejazi-6(HIIT) 2022 | Khalafi 2020 | Sirico 2018 |
|---|---|---|---|---|---|---|---|---|---|---|---|---|---|---|---|---|---|---|
| Loria-Kohen 2013[107] | 0 | 0 | 0 | 0 | 0 | 0 | 0 | 1 | 2 | 3 | 0 | 0 | 0 | 0 | 0 | 0 | 0 | 0 |
| Loimaala 2009[276] | 0 | 0 | 0 | 0 | 0 | 0 | 0 | 0 | 0 | 1 | 0 | 0 | 0 | 0 | 0 | 0 | 0 | 0 |
| Mendez-Gutierrez 2022[250] | 0 | 0 | 0 | 1 | 0 | 0 | 0 | 0 | 0 | 0 | 0 | 0 | 0 | 0 | 0 | 0 | 0 | 0 |
| Mendham 2014[251] | 0 | 0 | 0 | 0 | 0 | 0 | 0 | 1 | 0 | 0 | 0 | 0 | 2 | 0 | 0 | 0 | 0 | 0 |
| Mendham 2015[119] | 0 | 0 | 0 | 0 | 0 | 0 | 0 | 0 | 0 | 1 | 0 | 0 | 0 | 0 | 0 | 0 | 0 | 0 |
| Murakami 2007[277] | 0 | 0 | 0 | 0 | 0 | 0 | 0 | 1 | 0 | 0 | 0 | 0 | 0 | 0 | 0 | 0 | 0 | 0 |
| Nono 2020[135] | 0 | 0 | 0 | 0 | 0 | 0 | 0 | 0 | 0 | 1 | 0 | 0 | 0 | 0 | 0 | 0 | 0 | 0 |
| Nambi 2022[253] | 0 | 0 | 0 | 0 | 1 | 0 | 0 | 0 | 0 | 0 | 0 | 0 | 0 | 0 | 0 | 0 | 0 | 0 |
| Okada 2010[139] | 0 | 0 | 0 | 0 | 0 | 0 | 0 | 0 | 0 | 1 | 0 | 0 | 0 | 0 | 2 | 0 | 0 | 0 |
| Park 2007[141] | 0 | 0 | 0 | 0 | 0 | 0 | 1 | 0 | 0 | 0 | 0 | 0 | 0 | 0 | 0 | 0 | 0 | 0 |
| Pedrosa 2011[256] | 0 | 0 | 0 | 0 | 0 | 0 | 1 | 0 | 2 | 0 | 0 | 0 | 0 | 0 | 0 | 0 | 0 | 0 |
| Phillips 2012[147] | 0 | 0 | 0 | 0 | 0 | 0 | 0 | 0 | 0 | 0 | 0 | 1 | 0 | 0 | 0 | 0 | 0 | 0 |
| Racil 2015[259] | 0 | 0 | 0 | 0 | 0 | 0 | 0 | 0 | 0 | 0 | 0 | 0 | 0 | 0 | 0 | 0 | 1 | 0 |
| Racil 2016[260] | 0 | 0 | 0 | 0 | 1 | 0 | 0 | 0 | 0 | 0 | 0 | 0 | 0 | 0 | 0 | 0 | 2 | 3 |
| Reljic 2022[160] | 0 | 0 | 0 | 0 | 0 | 0 | 0 | 1 | 0 | 2 | 0 | 0 | 0 | 0 | 0 | 0 | 0 | 0 |
| Rashidlamir 2012[262] | 0 | 0 | 0 | 0 | 0 | 0 | 0 | 0 | 0 | 0 | 1 | 0 | 0 | 0 | 0 | 0 | 0 | 0 |
| Rogers 2013[278] | 0 | 0 | 1 | 0 | 0 | 0 | 0 | 0 | 0 | 0 | 0 | 0 | 0 | 0 | 0 | 0 | 0 | 0 |
| Rogers 2014[279] | 0 | 0 | 0 | 0 | 0 | 1 | 0 | 0 | 0 | 0 | 0 | 0 | 0 | 0 | 0 | 0 | 0 | 0 |
| Rokling-Andersen 2007[165] | 0 | 0 | 0 | 0 | 0 | 0 | 0 | 1 | 0 | 2 | 0 | 0 | 3 | 0 | 0 | 0 | 0 | 0 |
| Scott 2013[171] | 0 | 0 | 0 | 0 | 0 | 1 | 0 | 0 | 0 | 0 | 0 | 0 | 0 | 0 | 0 | 0 | 0 | 0 |
| Sokolovska 2020[267] | 0 | 0 | 0 | 0 | 0 | 0 | 0 | 0 | 0 | 0 | 0 | 0 | 0 | 0 | 0 | 1 | 0 | 0 |
| Straznicky 2011[180] | 0 | 0 | 0 | 0 | 0 | 0 | 0 | 1 | 0 | 0 | 0 | 0 | 0 | 0 | 0 | 0 | 0 | 0 |
| Tan 2016[280] | 0 | 0 | 0 | 0 | 0 | 0 | 0 | 1 | 0 | 0 | 0 | 0 | 0 | 0 | 0 | 0 | 0 | 0 |
| Tan 2018 [281] | 0 | 0 | 0 | 0 | 0 | 0 | 0 | 1 | 0 | 0 | 0 | 0 | 0 | 0 | 0 | 0 | 0 | 0 |
| Thong 2000[282] | 0 | 0 | 0 | 0 | 0 | 0 | 0 | 1 | 0 | 0 | 0 | 0 | 0 | 0 | 0 | 0 | 0 | 0 |
| Varady 2010[193] | 0 | 0 | 0 | 0 | 0 | 0 | 0 | 0 | 0 | 0 | 1 | 2 | 0 | 0 | 0 | 0 | 0 | 0 |
| Vatani 2015[283] | 0 | 0 | 0 | 0 | 0 | 0 | 0 | 0 | 0 | 1 | 0 | 0 | 0 | 0 | 0 | 0 | 0 | 0 |
| Vasconcellos 2016[194] | 0 | 0 | 0 | 0 | 1 | 0 | 0 | 0 | 0 | 0 | 0 | 0 | 0 | 0 | 0 | 0 | 0 | 2 |
| Venojärvi 2013[196] | 0 | 0 | 0 | 0 | 0 | 0 | 0 | 0 | 0 | 0 | 1 | 2 | 0 | 0 | 0 | 0 | 0 | 0 |
| Vella 2017[195] | 0 | 0 | 0 | 0 | 0 | 0 | 0 | 1 | 0 | 0 | 0 | 0 | 0 | 0 | 0 | 0 | 0 | 0 |
| Winters-Stone 2018[206] | 0 | 1 | 0 | 0 | 0 | 0 | 0 | 0 | 0 | 0 | 0 | 0 | 0 | 0 | 0 | 0 | 0 | 0 |
| Wong 2018[208] | 0 | 0 | 0 | 0 | 1 | 0 | 2 | 0 | 0 | 0 | 0 | 0 | 0 | 0 | 0 | 0 | 0 | 0 |
| Zhang 2017[214] | 0 | 0 | 0 | 0 | 0 | 0 | 0 | 0 | 0 | 0 | 0 | 0 | 1 | 0 | 0 | 0 | 0 | 0 |
| Zhang 2018[215] | 0 | 0 | 0 | 0 | 0 | 0 | 0 | 1 | 0 | 0 | 0 | 0 | 0 | 0 | 0 | 0 | 0 | 0 |

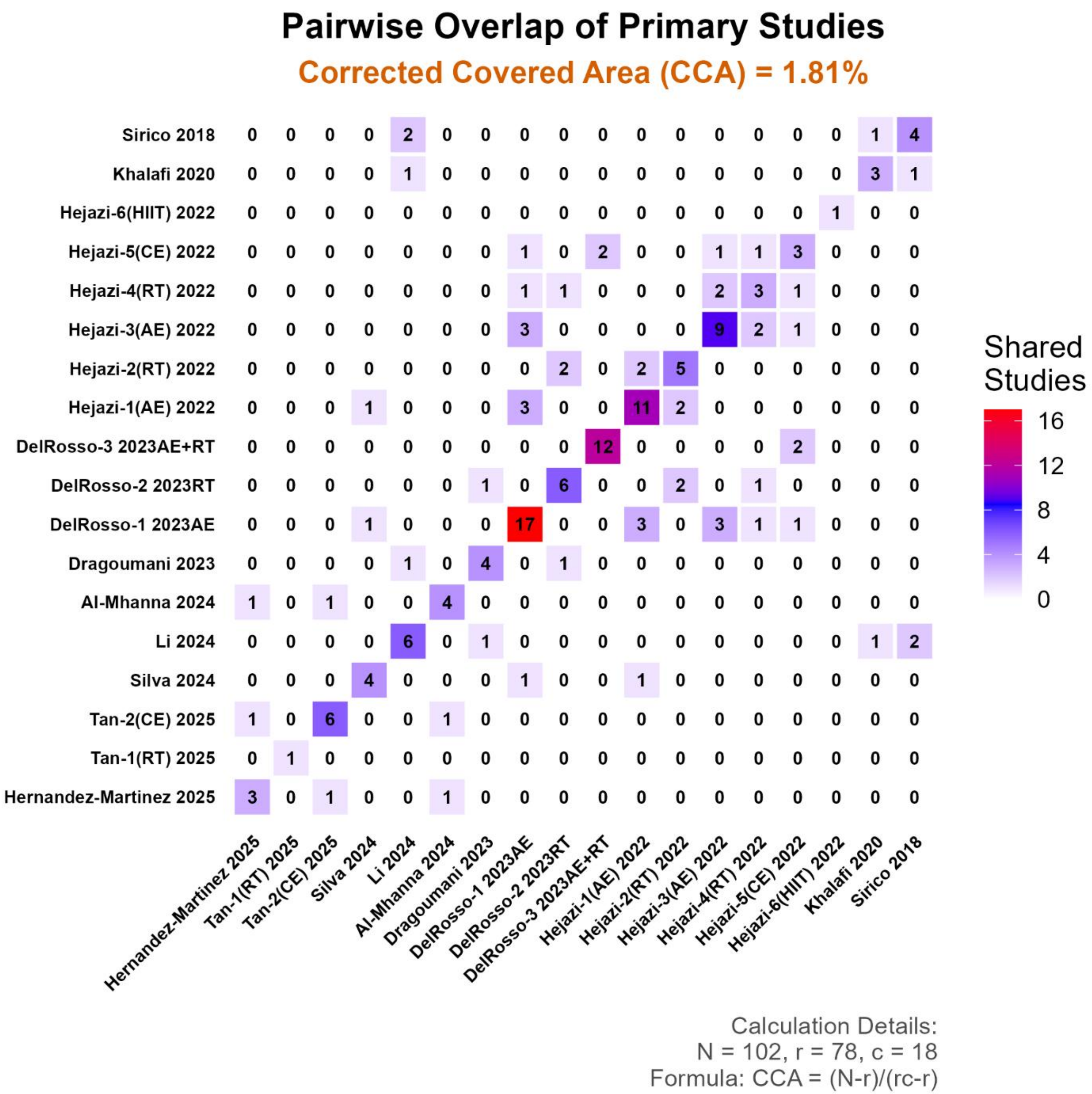


**Supplementary Figure S54. Graphical Overview of the Corrected Covered Area for Leptin**

## 7.4 Section 1: For TNF-α

Supplementary Table S12. Corrected Covered Area Overlap Analysis for TNF-α

| Primary study | Wan g 2025 | Tan-1 (AE) | Tan-2 (RT) | Tan-3 (CE) | Tan-4 (AE) | Tan-5 (RT) | Tan-6 (CE) | Silv a 2024 | Li 202 4 | Al-Mha nna-1 2024 | Al-Mha nna-2 2024 | Guo-1 (AE) | Guo-2 (RT) | Malan dish 2023 | Al-Mha nna 2023 | DelRos so-1_A eT | DelRos so-2_R T | DelRosso -3_AE+R T | Zhao -1 (AT) | Zhao -2 (RT) | Zhao-3 (AT+R T) | Hej azi-1 | Hej azi-2 | Hej azi-3 | Hej azi-4 | Hej azi-5 | Hej azi-6 | Khal afi 2022 | Liu 202 1 | Lee 202 1 | Khal afi 2020 | Siric o 2018 |
|---|---|---|---|---|---|---|---|---|---|---|---|---|---|---|---|---|---|---|---|---|---|---|---|---|---|---|---|---|---|---|---|---|
| Abd El-Kader 2015[284] | 0 | 0 | 0 | 0 | 0 | 0 | 0 | 0 | 0 | 0 | 0 | 0 | 0 | 0 | 0 | 1 | 0 | 0 | 0 | 0 | 0 | 0 | 0 | 0 | 0 | 0 | 0 | 0 | 0 | 0 | 0 | 0 |
| Abd El-Kader 2016[13] | 0 | 0 | 0 | 0 | 1 | 0 | 0 | 0 | 0 | 0 | 0 | 0 | 0 | 0 | 0 | 0 | 0 | 0 | 0 | 0 | 0 | 0 | 0 | 0 | 0 | 0 | 0 | 0 | 0 | 0 | 0 | 0 |
| Abd El-Kader 2020[285] | 0 | 0 | 0 | 0 | 0 | 0 | 0 | 0 | 0 | 0 | 0 | 0 | 0 | 0 | 1 | 0 | 0 | 0 | 0 | 0 | 0 | 0 | 0 | 0 | 0 | 0 | 0 | 0 | 0 | 0 | 0 | 0 |
| Abdollahpour 2016[286] | 0 | 0 | 0 | 0 | 1 | 0 | 0 | 0 | 0 | 0 | 0 | 0 | 0 | 0 | 0 | 0 | 0 | 0 | 0 | 0 | 0 | 0 | 0 | 0 | 0 | 0 | 0 | 0 | 0 | 0 | 0 | 0 |
| Adamopoulos 2002[287] | 0 | 0 | 0 | 0 | 0 | 0 | 0 | 0 | 0 | 0 | 0 | 0 | 0 | 1 | 0 | 0 | 0 | 0 | 0 | 0 | 0 | 0 | 0 | 0 | 0 | 0 | 0 | 0 | 0 | 0 | 0 | 0 |
| Ahmadizad 2015[288] | 0 | 0 | 0 | 0 | 0 | 0 | 0 | 0 | 0 | 0 | 0 | 0 | 0 | 0 | 0 | 0 | 0 | 0 | 0 | 0 | 0 | 0 | 0 | 0 | 0 | 0 | 0 | 0 | 0 | 0 | 1 | 0 |
| Allen 2017[21] | 0 | 0 | 0 | 0 | 0 | 0 | 0 | 0 | 0 | 0 | 0 | 0 | 0 | 0 | 0 | 0 | 0 | 0 | 0 | 0 | 0 | 0 | 0 | 0 | 0 | 0 | 0 | 0 | 0 | 0 | 1 | 0 |
| Annibalini 2017[24] | 0 | 0 | 0 | 0 | 0 | 0 | 0 | 0 | 0 | 0 | 1 | 0 | 0 | 0 | 0 | 0 | 0 | 2 | 0 | 0 | 0 | 0 | 0 | 0 | 0 | 0 | 3 | 0 | 0 | 0 | 0 | 0 |
| Arsenault 2009[26] | 0 | 0 | 0 | 0 | 1 | 0 | 0 | 0 | 0 | 0 | 0 | 2 | 0 | 0 | 0 | 3 | 0 | 0 | 0 | 0 | 0 | 4 | 0 | 0 | 0 | 0 | 0 | 0 | 0 | 0 | 0 | 0 |
| Auerbach 2013[27] | 0 | 0 | 0 | 0 | 0 | 0 | 0 | 0 | 0 | 0 | 0 | 0 | 0 | 0 | 0 | 0 | 0 | 0 | 0 | 0 | 0 | 0 | 0 | 0 | 0 | 0 | 0 | 1 | 0 | 0 | 0 | 0 |
| Balducci 2010[31] | 0 | 0 | 0 | 0 | 0 | 0 | 0 | 0 | 0 | 0 | 0 | 0 | 0 | 0 | 0 | 1 | 0 | 2 | 0 | 0 | 0 | 0 | 0 | 0 | 3 | 4 | 5 | 0 | 0 | 0 | 0 | 0 |
| Balen 2008[33] | 0 | 0 | 0 | 0 | 0 | 0 | 0 | 0 | 0 | 0 | 0 | 0 | 0 | 1 | 0 | 0 | 0 | 0 | 0 | 0 | 0 | 0 | 0 | 0 | 0 | 0 | 0 | 0 | 0 | 0 | 0 | 0 |
| Ben Ounis 2009[289] | 0 | 0 | 0 | 0 | 0 | 0 | 0 | 0 | 0 | 0 | 0 | 0 | 0 | 0 | 0 | 0 | 0 | 0 | 0 | 0 | 0 | 0 | 0 | 0 | 0 | 0 | 0 | 1 | 0 | 0 | 0 | 0 |
| Ben Ounis 2010[34] | 0 | 0 | 0 | 0 | 0 | 0 | 0 | 0 | 0 | 0 | 0 | 0 | 0 | 0 | 0 | 0 | 0 | 0 | 0 | 0 | 0 | 0 | 0 | 0 | 0 | 0 | 0 | 0 | 0 | 1 | 0 | 0 |
| Biteli 2021[290] | 0 | 0 | 0 | 0 | 1 | 0 | 0 | 0 | 0 | 0 | 0 | 0 | 0 | 0 | 0 | 0 | 0 | 0 | 0 | 0 | 0 | 0 | 0 | 0 | 0 | 0 | 0 | 0 | 0 | 0 | 0 | 0 |
| Brunelli 2015[42] | 0 | 0 | 0 | 0 | 0 | 0 | 0 | 1 | 0 | 0 | 0 | 0 | 0 | 0 | 0 | 0 | 0 | 0 | 0 | 0 | 0 | 0 | 0 | 0 | 0 | 0 | 0 | 0 | 0 | 0 | 0 | 0 |
| Byrkjeland 2011[43] | 0 | 0 | 0 | 0 | 0 | 0 | 0 | 0 | 0 | 0 | 0 | 0 | 0 | 1 | 0 | 0 | 0 | 0 | 0 | 0 | 0 | 0 | 0 | 0 | 0 | 0 | 0 | 0 | 0 | 0 | 0 | 0 |
| Chagas 2017[291] | 0 | 0 | 0 | 0 | 1 | 0 | 0 | 0 | 0 | 0 | 0 | 0 | 0 | 0 | 0 | 0 | 0 | 2 | 0 | 0 | 0 | 0 | 0 | 0 | 0 | 0 | 0 | 0 | 0 | 0 | 0 | 0 |
| Chen 2015[47] | 0 | 0 | 0 | 0 | 0 | 0 | 0 | 0 | 0 | 0 | 0 | 1 | 2 | 0 | 0 | 0 | 0 | 0 | 3 | 4 | 5 | 0 | 0 | 0 | 0 | 0 | 0 | 0 | 0 | 0 | 0 | 0 |
| Chow 2021[292] | 0 | 0 | 0 | 0 | 0 | 0 | 0 | 0 | 0 | 0 | 0 | 1 | 0 | 0 | 0 | 0 | 0 | 0 | 0 | 0 | 0 | 0 | 0 | 0 | 0 | 0 | 0 | 0 | 0 | 0 | 0 | 0 |
| Chupel 2017[293] | 0 | 0 | 0 | 0 | 0 | 1 | 0 | 0 | 0 | 0 | 0 | 0 | 0 | 0 | 0 | 0 | 0 | 0 | 0 | 0 | 0 | 0 | 0 | 0 | 0 | 0 | 0 | 0 | 0 | 0 | 0 | 0 |
| Chupel 2018[294] | 0 | 0 | 0 | 0 | 0 | 0 | 1 | 0 | 0 | 0 | 0 | 0 | 0 | 0 | 0 | 0 | 0 | 0 | 0 | 0 | 0 | 0 | 0 | 0 | 0 | 0 | 0 | 0 | 0 | 0 | 0 | 0 |
| Conraads 2002[295] | 0 | 0 | 0 | 0 | 0 | 0 | 0 | 0 | 0 | 0 | 0 | 0 | 0 | 1 | 0 | 0 | 0 | 0 | 0 | 0 | 0 | 0 | 0 | 0 | 0 | 0 | 0 | 0 | 0 | 0 | 0 | 0 |
| De Meirelles 2014[58] | 0 | 0 | 0 | 0 | 0 | 0 | 0 | 0 | 0 | 0 | 0 | 0 | 0 | 1 | 0 | 0 | 0 | 0 | 0 | 0 | 0 | 0 | 0 | 0 | 0 | 0 | 0 | 0 | 0 | 0 | 0 | 0 |
| Dieli-Conwrig ht 2018[59] | 0 | 0 | 0 | 1 | 0 | 0 | 0 | 0 | 0 | 2 | 0 | 0 | 0 | 0 | 0 | 0 | 0 | 0 | 0 | 0 | 0 | 0 | 0 | 0 | 0 | 0 | 0 | 0 | 0 | 0 | 0 | 0 |
| Donges 2013[60] | 0 | 0 | 0 | 0 | 0 | 0 | 0 | 1 | 0 | 0 | 0 | 0 | 0 | 0 | 0 | 2 | 3 | 4 | 0 | 0 | 0 | 0 | 0 | 0 | 0 | 0 | 0 | 0 | 0 | 0 | 0 | 0 |
| Erbs 2010[296] | 0 | 0 | 0 | 0 | 0 | 0 | 0 | 0 | 0 | 0 | 0 | 0 | 0 | 1 | 0 | 0 | 0 | 0 | 0 | 0 | 0 | 0 | 0 | 0 | 0 | 0 | 0 | 0 | 0 | 0 | 0 | 0 |
| Ergun 2013[297] | 0 | 0 | 0 | 0 | 0 | 0 | 0 | 0 | 0 | 1 | 0 | 0 | 0 | 0 | 0 | 0 | 0 | 0 | 0 | 0 | 0 | 0 | 0 | 0 | 0 | 0 | 0 | 0 | 0 | 0 | 0 | 0 |
| Feiereisen 2013[298] | 0 | 0 | 0 | 0 | 0 | 0 | 0 | 0 | 0 | 0 | 0 | 0 | 0 | 1 | 0 | 0 | 0 | 0 | 0 | 0 | 0 | 0 | 0 | 0 | 0 | 0 | 0 | 0 | 0 | 0 | 0 | 0 |
| Fernandes-Silv a 2017[299] | 0 | 0 | 0 | 0 | 0 | 0 | 0 | 0 | 0 | 0 | 0 | 0 | 0 | 1 | 0 | 0 | 0 | 0 | 0 | 0 | 0 | 0 | 0 | 0 | 0 | 0 | 0 | 0 | 0 | 0 | 0 | 0 |
| Fisher 2011[65] | 0 | 0 | 0 | 0 | 0 | 0 | 0 | 0 | 0 | 0 | 0 | 0 | 0 | 0 | 0 | 1 | 2 | 0 | 0 | 0 | 0 | 0 | 0 | 0 | 0 | 0 | 0 | 0 | 3 | 0 | 0 | 0 |
| Franklin 2015[67] | 0 | 0 | 0 | 0 | 0 | 0 | 0 | 0 | 0 | 0 | 0 | 0 | 0 | 0 | 0 | 0 | 1 | 0 | 0 | 0 | 0 | 0 | 0 | 0 | 0 | 0 | 0 | 0 | 0 | 0 | 0 | 0 |
| Friedenreich 2012[68] | 0 | 0 | 0 | 0 | 0 | 0 | 0 | 0 | 0 | 0 | 0 | 0 | 0 | 0 | 0 | 1 | 0 | 0 | 0 | 0 | 0 | 0 | 0 | 0 | 0 | 0 | 0 | 0 | 0 | 0 | 0 | 0 |
| Galedari 2017[300] | 0 | 0 | 0 | 0 | 0 | 0 | 0 | 0 | 0 | 0 | 0 | 0 | 0 | 0 | 0 | 0 | 1 | 0 | 0 | 0 | 0 | 0 | 0 | 0 | 0 | 0 | 0 | 0 | 2 | 0 | 0 | 0 |
| Giannopoulou 2005[71] | 0 | 0 | 0 | 0 | 0 | 0 | 0 | 0 | 0 | 0 | 0 | 0 | 0 | 0 | 0 | 0 | 0 | 0 | 0 | 0 | 0 | 0 | 0 | 0 | 0 | 0 | 0 | 1 | 2 | 0 | 0 | 0 |
| Gielen 2012[301] | 0 | 0 | 0 | 0 | 0 | 0 | 0 | 0 | 0 | 0 | 0 | 0 | 0 | 1 | 0 | 0 | 0 | 0 | 0 | 0 | 0 | 0 | 0 | 0 | 0 | 0 | 0 | 0 | 0 | 0 | 0 | 0 |
| Hagstrom 2016[75] | 0 | 0 | 1 | 0 | 0 | 0 | 0 | 0 | 0 | 0 | 0 | 0 | 0 | 0 | 0 | 0 | 0 | 0 | 0 | 0 | 0 | 0 | 0 | 0 | 0 | 0 | 0 | 0 | 0 | 0 | 0 | 0 |
| Ho 2013[302] | 0 | 0 | 0 | 0 | 0 | 0 | 0 | 0 | 0 | 0 | 0 | 1 | 2 | 0 | 0 | 0 | 0 | 0 | 0 | 0 | 0 | 0 | 0 | 0 | 0 | 0 | 0 | 0 | 0 | 0 | 0 | 0 |
| Johannsen 2012[184] | 0 | 0 | 0 | 0 | 1 | 0 | 0 | 0 | 0 | 0 | 0 | 0 | 0 | 0 | 0 | 0 | 0 | 0 | 0 | 0 | 0 | 1 | 0 | 0 | 0 | 0 | 0 | 0 | 0 | 0 | 0 | 0 |

| Primary study | Wang 2025 | Tan-1 (AE) | Tan-2 (RT) | Tan-3 (CE) | Tan-4 (AE) | Tan-5 (RT) | Tan-6 (CE) | Silva 2024 | Li 2024 | Al-Mhanna-1 2024 | Al-Mhanna-2 2024 | Guo-1 (AE) | Guo-2 (RT) | Malandish 2023 | Al-Mhanna 2023 | DelRosso-1_AeT | DelRosso-2_RT | DelRosso-3_AE+RT | Zhao-1 (AT) | Zhao-2 (RT) | Zhao-3 (AT+RT) | Hejazi-1 | Hejazi-2 | Hejazi-3 | Hejazi-4 | Hejazi-5 | Hejazi-6 | Khalafi 2022 | Liu 2021 | Lee 2021 | Khalafi 2020 | Sirico 2018 |
|---|---|---|---|---|---|---|---|---|---|---|---|---|---|---|---|---|---|---|---|---|---|---|---|---|---|---|---|---|---|---|---|---|
| Jones 2013[81] | 0 | 1 | 0 | 0 | 0 | 0 | 0 | 0 | 0 | 0 | 0 | 0 | 0 | 0 | 0 | 0 | 0 | 0 | 0 | 0 | 0 | 0 | 0 | 0 | 0 | 0 | 0 | 0 | 0 | 0 | 0 | 0 |
| Jorge 2011[82] | 1 | 0 | 0 | 0 | 0 | 0 | 0 | 0 | 0 | 0 | 0 | 0 | 0 | 0 | 0 | 0 | 0 | 0 | 0 | 0 | 0 | 0 | 0 | 0 | 2 | 3 | 4 | 0 | 0 | 0 | 0 | 0 |
| Kadoglou 2007a [240] | 0 | 0 | 0 | 0 | 0 | 0 | 0 | 0 | 0 | 0 | 0 | 0 | 0 | 0 | 0 | 0 | 0 | 0 | 0 | 0 | 0 | 0 | 0 | 0 | 1 | 0 | 0 | 0 | 0 | 0 | 0 | 0 |
| Kadoglou 2007b[83] | 0 | 0 | 0 | 0 | 0 | 0 | 0 | 0 | 0 | 0 | 0 | 0 | 0 | 0 | 0 | 1 | 0 | 2 | 0 | 0 | 0 | 0 | 0 | 0 | 3 | 0 | 0 | 0 | 0 | 0 | 0 | 0 |
| Karavidas 2006[303] | 0 | 0 | 0 | 0 | 0 | 0 | 0 | 0 | 0 | 0 | 0 | 0 | 0 | 1 | 0 | 0 | 0 | 0 | 0 | 0 | 0 | 0 | 0 | 0 | 0 | 0 | 0 | 0 | 0 | 0 | 0 | 0 |
| Kelly 2007[89] | 0 | 0 | 0 | 0 | 0 | 0 | 0 | 0 | 1 | 0 | 0 | 2 | 0 | 0 | 0 | 0 | 0 | 0 | 0 | 0 | 0 | 0 | 0 | 0 | 0 | 0 | 0 | 0 | 0 | 0 | 0 | 0 |
| Kim 2007[91] | 0 | 0 | 0 | 0 | 0 | 0 | 0 | 0 | 0 | 0 | 0 | 1 | 0 | 0 | 0 | 0 | 0 | 0 | 2 | 0 | 0 | 0 | 0 | 0 | 0 | 0 | 0 | 0 | 0 | 0 | 0 | 3 |
| Kim 2008[92] | 0 | 0 | 0 | 0 | 0 | 0 | 0 | 0 | 0 | 0 | 0 | 0 | 0 | 1 | 0 | 0 | 0 | 0 | 0 | 0 | 0 | 0 | 0 | 0 | 0 | 0 | 0 | 0 | 0 | 0 | 0 | 0 |
| Lakhdar 2013[304] | 0 | 0 | 0 | 0 | 0 | 0 | 0 | 0 | 0 | 0 | 0 | 0 | 0 | 0 | 0 | 1 | 0 | 0 | 0 | 0 | 0 | 0 | 0 | 0 | 0 | 0 | 0 | 0 | 2 | 0 | 0 | 0 |
| Lam 2015[98] | 0 | 0 | 0 | 0 | 0 | 0 | 0 | 0 | 0 | 0 | 0 | 0 | 0 | 0 | 0 | 0 | 0 | 0 | 0 | 0 | 0 | 0 | 0 | 0 | 0 | 0 | 0 | 0 | 1 | 0 | 0 | 0 |
| Lambert 2008[99] | 0 | 0 | 0 | 0 | 0 | 0 | 0 | 0 | 0 | 0 | 0 | 0 | 0 | 0 | 0 | 0 | 0 | 0 | 0 | 0 | 0 | 0 | 0 | 0 | 0 | 0 | 0 | 1 | 0 | 0 | 0 | 0 |
| Larsen 2001[305] | 0 | 0 | 0 | 0 | 0 | 0 | 0 | 0 | 0 | 0 | 0 | 0 | 0 | 1 | 0 | 0 | 0 | 0 | 0 | 0 | 0 | 0 | 0 | 0 | 0 | 0 | 0 | 0 | 0 | 0 | 0 | 0 |
| Lee 2012[101] | 0 | 0 | 0 | 0 | 0 | 0 | 0 | 0 | 0 | 0 | 0 | 1 | 0 | 0 | 0 | 0 | 0 | 0 | 0 | 0 | 0 | 0 | 0 | 0 | 0 | 0 | 0 | 0 | 0 | 0 | 0 | 0 |
| Libardi 2012[102] | 0 | 0 | 0 | 0 | 0 | 0 | 0 | 1 | 0 | 0 | 0 | 0 | 0 | 0 | 0 | 0 | 0 | 0 | 0 | 0 | 0 | 0 | 0 | 0 | 0 | 0 | 0 | 0 | 0 | 0 | 0 | 0 |
| Linke 2005[306] | 0 | 0 | 0 | 0 | 0 | 0 | 0 | 0 | 0 | 0 | 0 | 0 | 0 | 1 | 0 | 0 | 0 | 0 | 0 | 0 | 0 | 0 | 0 | 0 | 0 | 0 | 0 | 0 | 0 | 0 | 0 | 0 |
| Liu 2018[105] | 0 | 0 | 0 | 0 | 0 | 0 | 0 | 0 | 1 | 0 | 0 | 0 | 0 | 0 | 0 | 0 | 0 | 0 | 0 | 0 | 0 | 0 | 0 | 0 | 0 | 0 | 0 | 0 | 0 | 0 | 0 | 0 |
| Lopes 2016[106] | 0 | 0 | 0 | 0 | 0 | 0 | 0 | 0 | 0 | 0 | 0 | 0 | 0 | 0 | 0 | 0 | 0 | 0 | 0 | 0 | 1 | 0 | 0 | 0 | 0 | 0 | 0 | 0 | 0 | 0 | 0 | 0 |
| Loria-Kohen 2013[107] | 0 | 0 | 0 | 0 | 0 | 0 | 0 | 0 | 0 | 0 | 0 | 0 | 0 | 0 | 0 | 1 | 2 | 3 | 0 | 0 | 0 | 0 | 0 | 0 | 0 | 0 | 0 | 0 | 0 | 0 | 0 | 0 |
| Magalhaes 2020[109] | 0 | 0 | 0 | 0 | 0 | 0 | 0 | 0 | 0 | 0 | 0 | 0 | 0 | 0 | 0 | 1 | 0 | 0 | 0 | 0 | 0 | 0 | 0 | 0 | 0 | 0 | 0 | 0 | 0 | 0 | 0 | 0 |
| Magalhães 2019[108] | 0 | 0 | 0 | 0 | 0 | 0 | 0 | 0 | 0 | 0 | 1 | 0 | 0 | 0 | 0 | 2 | 0 | 0 | 0 | 0 | 0 | 0 | 0 | 0 | 0 | 0 | 0 | 0 | 0 | 0 | 0 | 0 |
| Martins 2023[307] | 0 | 0 | 1 | 0 | 0 | 0 | 0 | 0 | 0 | 0 | 0 | 0 | 0 | 0 | 0 | 0 | 0 | 0 | 0 | 0 | 0 | 0 | 0 | 0 | 0 | 0 | 0 | 0 | 0 | 0 | 0 | 0 |
| Melo 2019[308] | 0 | 0 | 0 | 0 | 0 | 0 | 0 | 0 | 0 | 0 | 0 | 0 | 0 | 1 | 0 | 0 | 0 | 0 | 0 | 0 | 0 | 0 | 0 | 0 | 0 | 0 | 0 | 0 | 0 | 0 | 0 | 0 |
| Mendham 2014[119] | 0 | 0 | 0 | 0 | 0 | 0 | 0 | 0 | 0 | 0 | 0 | 0 | 0 | 0 | 0 | 1 | 0 | 0 | 0 | 0 | 0 | 0 | 0 | 0 | 2 | 0 | 0 | 0 | 0 | 0 | 0 | 0 |
| Mendham 2015[251] | 0 | 0 | 0 | 0 | 0 | 0 | 0 | 0 | 0 | 0 | 0 | 0 | 0 | 0 | 0 | 0 | 0 | 1 | 0 | 0 | 0 | 0 | 0 | 0 | 0 | 0 | 0 | 0 | 0 | 0 | 0 | 0 |
| Munk 2011[309] | 0 | 0 | 0 | 0 | 0 | 0 | 0 | 0 | 0 | 0 | 0 | 0 | 0 | 1 | 0 | 0 | 0 | 0 | 0 | 0 | 0 | 0 | 0 | 0 | 0 | 0 | 0 | 0 | 0 | 0 | 0 | 0 |
| Murphy 2009[128] | 0 | 0 | 0 | 0 | 0 | 0 | 0 | 0 | 1 | 0 | 0 | 0 | 0 | 0 | 0 | 0 | 0 | 0 | 0 | 0 | 0 | 0 | 0 | 0 | 0 | 0 | 0 | 0 | 0 | 2 | 0 | 0 |
| Nadi 2019[130] | 1 | 0 | 0 | 0 | 0 | 0 | 0 | 0 | 0 | 0 | 0 | 0 | 0 | 0 | 0 | 0 | 0 | 0 | 0 | 0 | 0 | 0 | 0 | 0 | 0 | 0 | 0 | 0 | 0 | 0 | 0 | 0 |
| Nambi 2022[253] | 0 | 0 | 0 | 0 | 0 | 0 | 0 | 0 | 1 | 0 | 0 | 0 | 0 | 0 | 0 | 0 | 0 | 0 | 0 | 0 | 0 | 0 | 0 | 0 | 0 | 0 | 0 | 0 | 0 | 0 | 0 | 0 |
| Nicklas 2004[132] | 0 | 0 | 0 | 0 | 0 | 0 | 0 | 0 | 0 | 0 | 0 | 0 | 0 | 0 | 0 | 0 | 0 | 0 | 0 | 0 | 0 | 0 | 0 | 0 | 0 | 0 | 0 | 1 | 2 | 0 | 0 | 0 |
| Niebauer 2005[310] | 0 | 0 | 0 | 0 | 0 | 0 | 0 | 0 | 0 | 0 | 0 | 0 | 0 | 1 | 0 | 0 | 0 | 0 | 0 | 0 | 0 | 0 | 0 | 0 | 0 | 0 | 0 | 0 | 0 | 0 | 0 | 0 |
| Nikseresht 2014a[254] | 0 | 0 | 0 | 0 | 0 | 0 | 0 | 0 | 0 | 0 | 0 | 0 | 0 | 0 | 0 | 1 | 0 | 0 | 0 | 0 | 0 | 2 | 0 | 0 | 0 | 0 | 0 | 0 | 0 | 0 | 0 | 0 |
| Nikseresht 2014b[133] | 0 | 0 | 0 | 0 | 0 | 0 | 0 | 0 | 0 | 0 | 0 | 0 | 0 | 0 | 0 | 0 | 1 | 0 | 0 | 0 | 0 | 0 | 2 | 0 | 0 | 0 | 0 | 0 | 0 | 0 | 3 | 0 |
| Nono Nankam 2020[135] | 0 | 0 | 0 | 0 | 0 | 0 | 0 | 0 | 0 | 0 | 0 | 0 | 0 | 0 | 0 | 0 | 0 | 1 | 0 | 0 | 0 | 0 | 0 | 0 | 0 | 0 | 0 | 0 | 0 | 0 | 0 | 0 |
| Nunes 2016[136] | 0 | 0 | 0 | 0 | 0 | 1 | 0 | 0 | 0 | 0 | 0 | 0 | 0 | 0 | 0 | 0 | 2 | 0 | 0 | 0 | 0 | 0 | 0 | 0 | 0 | 0 | 0 | 0 | 0 | 0 | 0 | 0 |
| Oh 2014[138] | 0 | 0 | 0 | 0 | 0 | 0 | 0 | 0 | 0 | 0 | 0 | 0 | 0 | 0 | 0 | 0 | 0 | 0 | 0 | 0 | 0 | 0 | 0 | 0 | 0 | 0 | 0 | 0 | 1 | 0 | 0 | 0 |
| Park 2015[311] | 0 | 0 | 0 | 0 | 0 | 0 | 1 | 2 | 0 | 0 | 0 | 0 | 0 | 0 | 0 | 0 | 0 | 0 | 0 | 0 | 0 | 0 | 0 | 0 | 0 | 0 | 0 | 0 | 0 | 0 | 0 | 0 |
| Pedersen 2016[145] | 0 | 0 | 0 | 0 | 0 | 0 | 0 | 0 | 0 | 0 | 0 | 0 | 0 | 0 | 0 | 0 | 0 | 0 | 0 | 0 | 0 | 0 | 0 | 0 | 0 | 0 | 0 | 1 | 0 | 0 | 0 | 0 |
| Peña 2023[312] | 0 | 0 | 0 | 0 | 0 | 0 | 0 | 0 | 1 | 0 | 0 | 0 | 0 | 0 | 0 | 0 | 0 | 0 | 0 | 0 | 0 | 0 | 0 | 0 | 0 | 0 | 0 | 0 | 0 | 0 | 0 | 0 |
| Phillips 2012[147] | 0 | 0 | 0 | 0 | 0 | 1 | 0 | 0 | 0 | 0 | 0 | 0 | 0 | 0 | 0 | 0 | 2 | 0 | 0 | 0 | 0 | 0 | 3 | 0 | 0 | 0 | 0 | 0 | 0 | 0 | 0 | 0 |
| Pierce 2008[148] | 0 | 0 | 0 | 0 | 0 | 0 | 0 | 0 | 0 | 0 | 0 | 0 | 0 | 1 | 0 | 0 | 0 | 0 | 0 | 0 | 0 | 0 | 0 | 0 | 0 | 0 | 0 | 0 | 0 | 0 | 0 | 0 |
| Prescott 2009[151] | 0 | 0 | 0 | 0 | 0 | 0 | 0 | 0 | 0 | 0 | 0 | 0 | 0 | 1 | 0 | 0 | 0 | 0 | 0 | 0 | 0 | 0 | 0 | 0 | 0 | 0 | 0 | 0 | 0 | 0 | 0 | 0 |

| Primary study | Wang 2025 | Tan-1 (AE) | Tan-2 (RT) | Tan-3 (CE) | Tan-4 (AE) | Tan-5 (RT) | Tan-6 (CE) | Silva 2024 | Li 2024 | Al-Mhanna-1 2024 | Al-Mhanna-2 2024 | Guo-1 (AE) | Guo-2 (RT) | Malandish 2023 | Al-Mhanna 2023 | DelRosso-1_AeT | DelRosso-2_RT | DelRosso-3_AE+RT | Zhao-1 (AT) | Zhao-2 (RT) | Zhao-3 (AT+RT) | Hejazi-1 | Hejazi-2 | Hejazi-3 | Hejazi-4 | Hejazi-5 | Hejazi-6 | Khalafi 2022 | Liu 2021 | Lee 2021 | Khalafi 2020 | Sirico 2018 |
|---|---|---|---|---|---|---|---|---|---|---|---|---|---|---|---|---|---|---|---|---|---|---|---|---|---|---|---|---|---|---|---|---|
| Racca 2020[313] | 0 | 0 | 0 | 0 | 0 | 0 | 0 | 0 | 0 | 0 | 0 | 0 | 0 | 1 | 0 | 0 | 0 | 0 | 0 | 0 | 0 | 0 | 0 | 0 | 0 | 0 | 0 | 0 | 0 | 0 | 0 | 0 |
| Rech 2019[156] | 1 | 0 | 0 | 0 | 0 | 0 | 0 | 0 | 0 | 0 | 0 | 0 | 0 | 0 | 0 | 0 | 0 | 0 | 0 | 0 | 0 | 0 | 0 | 0 | 0 | 0 | 0 | 0 | 0 | 0 | 0 | 0 |
| Redwine 2020[157] | 0 | 0 | 0 | 0 | 0 | 0 | 0 | 0 | 0 | 0 | 0 | 0 | 0 | 1 | 0 | 0 | 0 | 0 | 0 | 0 | 0 | 0 | 0 | 0 | 0 | 0 | 0 | 0 | 0 | 0 | 0 | 0 |
| Rejeski 2019[159] | 0 | 0 | 0 | 0 | 0 | 0 | 0 | 0 | 0 | 0 | 0 | 0 | 0 | 0 | 0 | 1 | 0 | 0 | 0 | 0 | 0 | 0 | 0 | 0 | 0 | 0 | 0 | 0 | 0 | 0 | 0 | 0 |
| Rezende 2016[314] | 0 | 0 | 0 | 0 | 1 | 0 | 0 | 0 | 0 | 0 | 0 | 0 | 0 | 0 | 0 | 0 | 0 | 0 | 0 | 0 | 0 | 0 | 0 | 0 | 0 | 0 | 0 | 0 | 0 | 0 | 0 | 0 |
| Rodrigues-Krause 2018[163] | 0 | 0 | 0 | 0 | 0 | 0 | 0 | 0 | 0 | 0 | 0 | 0 | 0 | 0 | 0 | 1 | 0 | 0 | 0 | 0 | 0 | 0 | 0 | 0 | 0 | 0 | 0 | 0 | 0 | 0 | 0 | 0 |
| Rogers 2013[278] | 0 | 0 | 0 | 1 | 0 | 0 | 0 | 0 | 0 | 0 | 0 | 0 | 0 | 0 | 0 | 0 | 0 | 0 | 0 | 0 | 0 | 0 | 0 | 0 | 0 | 0 | 0 | 0 | 0 | 0 | 0 | 0 |
| Rogers 2014[279] | 0 | 0 | 0 | 1 | 0 | 0 | 0 | 0 | 0 | 2 | 0 | 0 | 0 | 0 | 0 | 0 | 0 | 0 | 0 | 0 | 0 | 0 | 0 | 0 | 0 | 0 | 0 | 0 | 0 | 0 | 0 | 0 |
| Rokling-Andersen 2007[165] | 0 | 0 | 0 | 0 | 0 | 0 | 0 | 0 | 0 | 0 | 0 | 0 | 0 | 0 | 0 | 0 | 0 | 1 | 0 | 0 | 0 | 0 | 0 | 0 | 2 | 0 | 0 | 3 | 0 | 0 | 0 | 0 |
| Sabouri 2021[167] | 1 | 0 | 0 | 0 | 0 | 0 | 0 | 0 | 0 | 0 | 0 | 0 | 0 | 0 | 0 | 0 | 0 | 0 | 0 | 0 | 0 | 0 | 0 | 0 | 0 | 0 | 0 | 0 | 0 | 0 | 0 | 0 |
| Salamat 2016[315] | 0 | 0 | 0 | 0 | 0 | 0 | 0 | 1 | 0 | 0 | 0 | 0 | 0 | 0 | 0 | 2 | 3 | 4 | 0 | 0 | 0 | 0 | 0 | 0 | 0 | 0 | 0 | 0 | 0 | 0 | 0 | 0 |
| Saxton 2014[316] | 0 | 0 | 0 | 0 | 0 | 0 | 0 | 0 | 0 | 1 | 0 | 0 | 0 | 0 | 0 | 0 | 0 | 0 | 0 | 0 | 0 | 0 | 0 | 0 | 0 | 0 | 0 | 0 | 0 | 0 | 0 | 0 |
| Shahram 2016[317] | 0 | 0 | 0 | 0 | 0 | 0 | 0 | 0 | 0 | 0 | 0 | 0 | 0 | 0 | 0 | 1 | 2 | 0 | 0 | 0 | 0 | 0 | 0 | 0 | 0 | 0 | 0 | 0 | 0 | 0 | 0 | 0 |
| Silverman 2009[318] | 0 | 0 | 0 | 0 | 1 | 0 | 0 | 0 | 0 | 0 | 0 | 0 | 0 | 0 | 0 | 0 | 0 | 0 | 0 | 0 | 0 | 0 | 0 | 0 | 0 | 0 | 0 | 0 | 2 | 0 | 0 | 0 |
| Snel 2011[177] | 0 | 0 | 0 | 0 | 0 | 0 | 0 | 0 | 0 | 0 | 0 | 0 | 0 | 0 | 0 | 0 | 0 | 0 | 0 | 0 | 0 | 0 | 0 | 0 | 0 | 0 | 0 | 0 | 1 | 0 | 0 | 0 |
| Taheri-Chadorneshin 2019[319] | 0 | 0 | 0 | 0 | 0 | 0 | 0 | 0 | 0 | 0 | 0 | 0 | 0 | 0 | 0 | 0 | 0 | 0 | 0 | 0 | 0 | 0 | 0 | 0 | 0 | 0 | 0 | 0 | 0 | 0 | 1 | 0 |
| Tartibian 2015[186] | 0 | 0 | 0 | 0 | 1 | 0 | 0 | 0 | 0 | 0 | 0 | 0 | 0 | 0 | 0 | 0 | 0 | 0 | 0 | 0 | 0 | 0 | 0 | 0 | 0 | 0 | 0 | 0 | 0 | 0 | 0 | 0 |
| Tomeleri 2016[189] | 0 | 0 | 0 | 0 | 0 | 1 | 0 | 0 | 0 | 0 | 0 | 0 | 2 | 0 | 0 | 0 | 3 | 0 | 0 | 0 | 0 | 0 | 0 | 0 | 0 | 0 | 0 | 0 | 0 | 0 | 0 | 0 |
| Tomeleri 2018[190] | 0 | 0 | 0 | 0 | 0 | 1 | 0 | 0 | 0 | 0 | 0 | 0 | 0 | 0 | 0 | 0 | 2 | 0 | 0 | 0 | 0 | 0 | 0 | 0 | 0 | 0 | 0 | 0 | 0 | 0 | 0 | 0 |
| Trippel 2017[320] | 0 | 0 | 0 | 0 | 0 | 0 | 0 | 0 | 0 | 0 | 0 | 0 | 0 | 1 | 0 | 0 | 0 | 0 | 0 | 0 | 0 | 0 | 0 | 0 | 0 | 0 | 0 | 0 | 0 | 0 | 0 | 0 |
| Tsarouhas 2011[321] | 0 | 0 | 0 | 0 | 0 | 0 | 0 | 0 | 0 | 0 | 0 | 0 | 0 | 1 | 0 | 0 | 0 | 0 | 0 | 0 | 0 | 0 | 0 | 0 | 0 | 0 | 0 | 0 | 0 | 0 | 0 | 0 |
| Vasconcellos 2016[194] | 0 | 0 | 0 | 0 | 0 | 0 | 0 | 0 | 1 | 0 | 0 | 0 | 0 | 0 | 0 | 0 | 0 | 0 | 2 | 0 | 0 | 0 | 0 | 0 | 0 | 0 | 0 | 0 | 0 | 0 | 0 | 3 |
| Vasconcelos 2020[322] | 0 | 0 | 0 | 0 | 0 | 0 | 1 | 0 | 0 | 0 | 0 | 0 | 0 | 0 | 0 | 0 | 0 | 0 | 0 | 0 | 0 | 0 | 0 | 0 | 0 | 0 | 0 | 0 | 0 | 0 | 0 | 0 |
| Vella 2017[195] | 0 | 0 | 0 | 0 | 0 | 0 | 0 | 0 | 0 | 0 | 0 | 1 | 0 | 0 | 0 | 2 | 0 | 0 | 0 | 0 | 0 | 0 | 0 | 0 | 0 | 0 | 0 | 0 | 0 | 0 | 0 | 0 |
| Wanderley 2013[200] | 0 | 0 | 0 | 0 | 0 | 0 | 0 | 0 | 0 | 0 | 0 | 0 | 0 | 0 | 0 | 1 | 2 | 0 | 0 | 0 | 0 | 0 | 0 | 0 | 0 | 0 | 0 | 0 | 0 | 0 | 0 | 0 |
| Wang 2006[201] | 0 | 0 | 0 | 0 | 0 | 0 | 0 | 0 | 0 | 0 | 0 | 1 | 0 | 0 | 0 | 0 | 0 | 0 | 0 | 0 | 0 | 0 | 0 | 0 | 0 | 0 | 0 | 0 | 0 | 0 | 0 | 0 |
| Wang 2018[203] | 0 | 0 | 0 | 0 | 0 | 0 | 0 | 0 | 0 | 0 | 0 | 1 | 0 | 0 | 0 | 0 | 0 | 0 | 0 | 0 | 0 | 0 | 0 | 0 | 0 | 0 | 0 | 0 | 0 | 0 | 0 | 0 |
| Winters-Stone 2018[206] | 0 | 0 | 1 | 0 | 0 | 0 | 0 | 0 | 0 | 0 | 0 | 0 | 0 | 0 | 0 | 0 | 0 | 0 | 0 | 0 | 0 | 0 | 0 | 0 | 0 | 0 | 0 | 0 | 0 | 0 | 0 | 0 |
| Yeh 2011[210] | 0 | 0 | 0 | 0 | 0 | 0 | 0 | 0 | 0 | 0 | 0 | 0 | 0 | 1 | 0 | 0 | 0 | 0 | 0 | 0 | 0 | 0 | 0 | 0 | 0 | 0 | 0 | 0 | 0 | 0 | 0 | 0 |
| You 2004[213] | 0 | 0 | 0 | 0 | 1 | 0 | 0 | 0 | 0 | 0 | 0 | 2 | 0 | 0 | 0 | 0 | 0 | 0 | 0 | 0 | 0 | 0 | 0 | 0 | 0 | 0 | 0 | 0 | 0 | 0 | 0 | 0 |

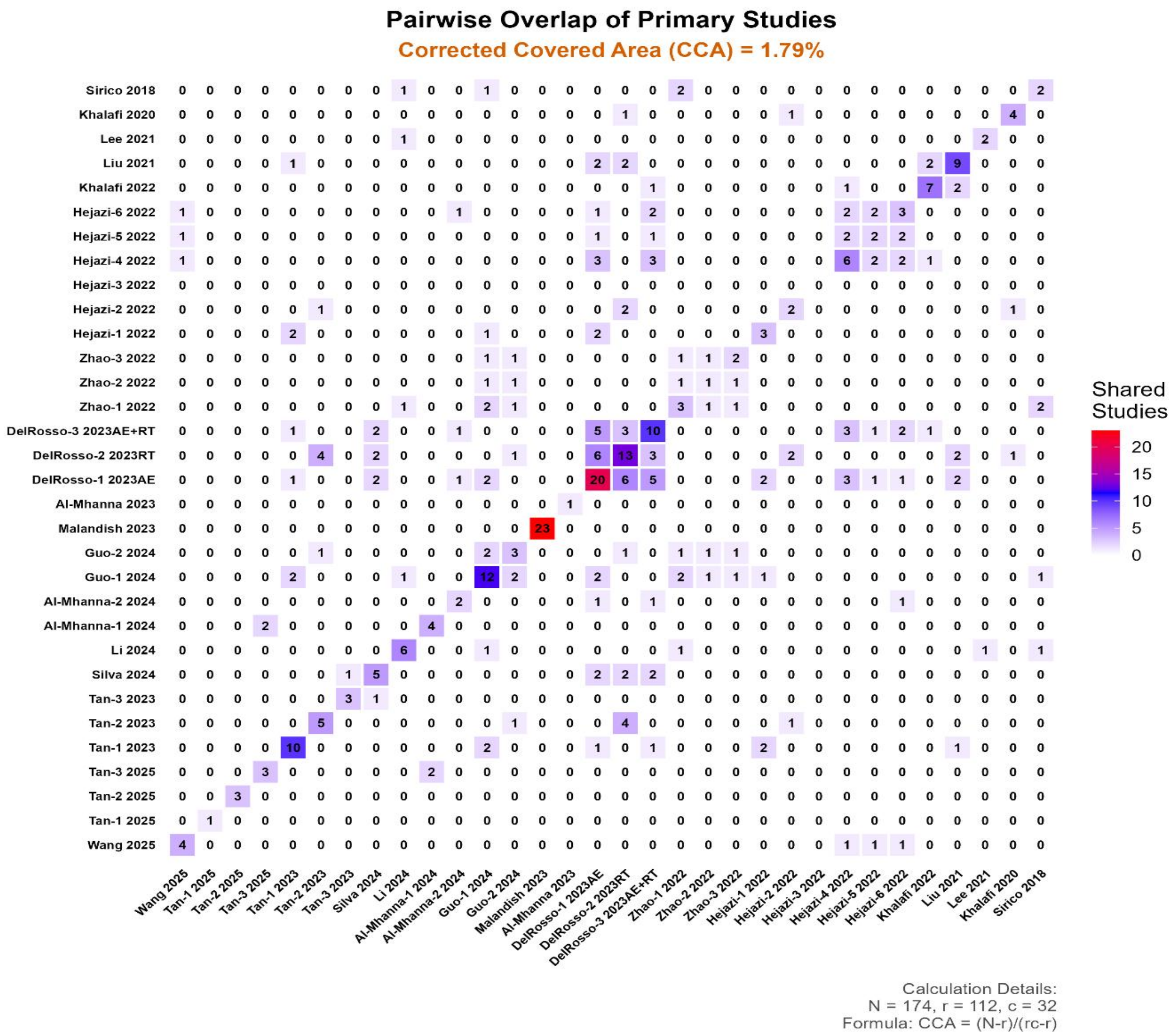


**Supplementary Figure S56. Graphical Overview of the Corrected Covered Area for TNF-α**

## 7.5 Section 1: For IL-1β

Supplementary Table S13. Corrected Covered Area (CCA) Overlap Analysis for IL-1β

| Primary study | Del Rosso 2023 | Ding 2022 | Zalagkitis 2025 | Malandish 2023 |
|---|---|---|---|---|
| Asjari 2021[323] | 0 | 1 | 0 | 0 |
| Auerbach 2013[27] | 0 | 1 | 0 | 0 |
| Balducci 2010[31] | 1 | 0 | 0 | 0 |
| Barron-Cabrera 2020[324] | 0 | 1 | 0 | 0 |
| Butts 2018[325] | 0 | 0 | 0 | 1 |
| Huffman 2008[326] | 0 | 1 | 0 | 0 |
| Kim 2008[92] | 0 | 0 | 0 | 1 |
| Linke 2005[306] | 0 | 0 | 0 | 1 |
| Liu 2018[105] | 0 | 1 | 2 | 0 |
| Masquio 2023[114] | 0 | 0 | 1 | 0 |
| Mendham 2014[251] | 1 | 2 | 0 | 0 |
| Mendham 2015[119] | 0 | 1 | 0 | 0 |
| Prestes 2015[327] | 0 | 1 | 0 | 0 |
| Racca 2020[313] | 0 | 0 | 0 | 1 |
| Reljic 2022[160] | 1 | 0 | 0 | 0 |
| Romeo 2011[328] | 0 | 0 | 1 | 0 |
| Roth 2011[329] | 0 | 0 | 1 | 0 |
| Salamat 2016[315] | 1 | 2 | 0 | 0 |
| So 2013[330] | 0 | 1 | 0 | 0 |
| Tartibian 2015[186] | 0 | 1 | 0 | 0 |
| Tavvafian 2020[331] | 0 | 1 | 0 | 0 |
| Trippel 2017[320] | 0 | 0 | 0 | 1 |
| Zakavi 2020[332] | 0 | 1 | 0 | 0 |

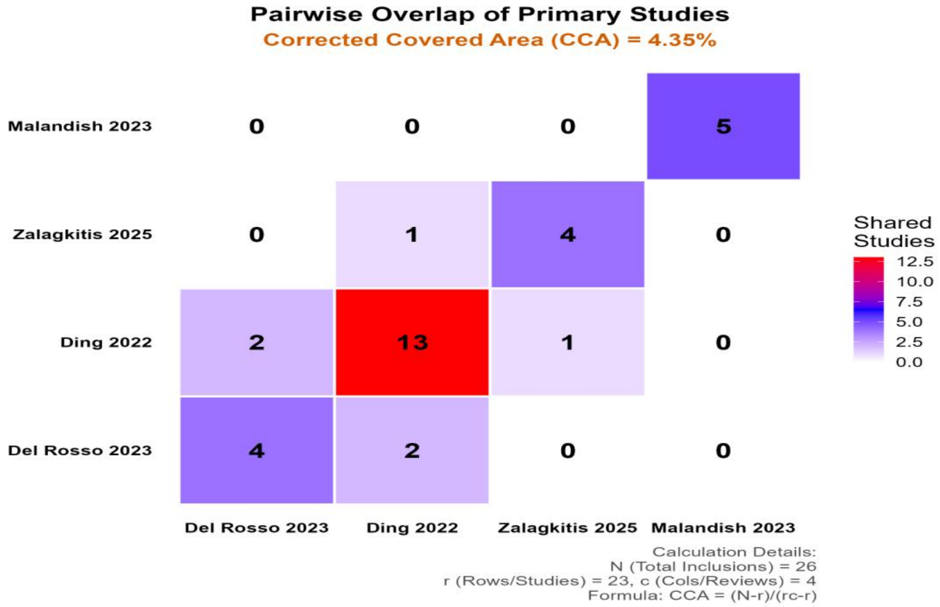


**Supplementary Figure S58. Graphical Overview of the Corrected Covered Area (CCA) for IL-1β**

## 7.6 Section 1: For IL-6

Supplementary Table S14. Corrected Covered Area Overlap Analysis for IL-6

| Primary study | Zalagkitis-1 2025 | Zalagkitis-2 2025 | Wang 2025 | Hernandez-Martinez 2025 | Tan-1 2025 (AE) | Tan-2 2025 (RT) | Tan-3 2025 (CE) | Silva 2024 | Li 2024 | Al-Mhanna-1 2024 | Al-Mhanna-2 2024 | Guo-1 (AE) | Guo-2 (RE) | Malandish 2023 | Tan-1 (AE) | Tan-2 (RT) | Tan-3 (CE) | Del Rosso-1 (AE) | Del Rosso-2 (RT) | Del Rosso-3 (AE+RT) | Rahimi 2022 | Zhao-1 (AT) | Zhao-2 (RT) | Zhao-3 (AT+RT) | Hejazi-1 (AE) | Hejazi-2 (RT) | Hejazi-3 (AE) | Hejazi-4 (RT) | Hejazi-5 (AE+RT) | Khalafi 2022 | Liu 2021 | Lee 2021 | Khalafi 2020 | Sirico 2018 |
|---|---|---|---|---|---|---|---|---|---|---|---|---|---|---|---|---|---|---|---|---|---|---|---|---|---|---|---|---|---|---|---|---|---|---|
| Abd El-Kader 2015[284] | 0 | 0 | 0 | 0 | 0 | 0 | 0 | 0 | 0 | 0 | 0 | 0 | 0 | 0 | 0 | 0 | 0 | 1 | 0 | 0 | 0 | 0 | 0 | 0 | 0 | 0 | 0 | 0 | 0 | 0 | 0 | 0 | 0 | 0 |
| Abd El-Kader 2016[13] | 0 | 0 | 0 | 0 | 0 | 0 | 0 | 0 | 0 | 0 | 0 | 0 | 0 | 0 | 1 | 0 | 0 | 0 | 0 | 0 | 0 | 0 | 0 | 0 | 0 | 0 | 0 | 0 | 0 | 0 | 0 | 0 | 0 | 0 |
| Abdollahpour 2016[286] | 0 | 0 | 0 | 0 | 0 | 0 | 0 | 0 | 0 | 0 | 0 | 0 | 0 | 0 | 1 | 0 | 0 | 0 | 0 | 0 | 0 | 0 | 0 | 0 | 0 | 0 | 0 | 0 | 0 | 0 | 0 | 0 | 0 | 0 |
| Adamopoulos 2002[287] | 0 | 0 | 0 | 0 | 0 | 0 | 0 | 0 | 0 | 0 | 0 | 0 | 0 | 1 | 0 | 0 | 0 | 0 | 0 | 0 | 0 | 0 | 0 | 0 | 0 | 0 | 0 | 0 | 0 | 0 | 0 | 0 | 0 | 0 |
| Ahmadizad 2015[288] | 0 | 0 | 0 | 0 | 0 | 0 | 0 | 0 | 0 | 0 | 0 | 0 | 0 | 0 | 0 | 0 | 0 | 0 | 0 | 0 | 0 | 0 | 0 | 0 | 0 | 0 | 0 | 0 | 0 | 0 | 0 | 0 | 1 | 0 |
| Akbarpour 2013[18] | 0 | 0 | 0 | 0 | 0 | 0 | 0 | 0 | 0 | 0 | 0 | 0 | 0 | 0 | 0 | 0 | 0 | 1 | 0 | 0 | 0 | 0 | 0 | 0 | 2 | 0 | 0 | 0 | 0 | 0 | 0 | 0 | 0 | 0 |
| Annibalini 2017 [24] | 0 | 0 | 0 | 0 | 0 | 0 | 0 | 0 | 0 | 0 | 1 | 0 | 0 | 0 | 0 | 0 | 0 | 0 | 0 | 2 | 0 | 0 | 0 | 0 | 0 | 0 | 0 | 0 | 3 | 0 | 0 | 0 | 0 | 0 |
| Arsenault 2009[26] | 0 | 0 | 0 | 0 | 0 | 0 | 0 | 0 | 0 | 0 | 0 | 1 | 0 | 0 | 2 | 0 | 0 | 3 | 0 | 0 | 0 | 0 | 0 | 0 | 4 | 0 | 0 | 0 | 0 | 0 | 0 | 0 | 0 | 0 |
| Bagheri 2020 [28] | 0 | 0 | 0 | 0 | 0 | 0 | 0 | 0 | 0 | 0 | 0 | 0 | 0 | 0 | 0 | 0 | 0 | 0 | 0 | 0 | 0 | 0 | 0 | 0 | 1 | 0 | 0 | 0 | 0 | 0 | 0 | 0 | 0 | 0 |
| Bahmanbeglou 2019 [333] | 0 | 0 | 0 | 0 | 0 | 0 | 0 | 0 | 0 | 0 | 0 | 0 | 0 | 0 | 0 | 0 | 0 | 0 | 0 | 0 | 0 | 0 | 0 | 0 | 0 | 0 | 0 | 0 | 0 | 0 | 0 | 0 | 1 | 0 |
| Balagopal 2005 [30] | 1 | 0 | 0 | 0 | 0 | 0 | 0 | 0 | 0 | 0 | 0 | 0 | 0 | 0 | 0 | 0 | 0 | 0 | 0 | 0 | 0 | 0 | 0 | 0 | 0 | 0 | 0 | 0 | 0 | 0 | 0 | 0 | 0 | 0 |
| Balducci 2010[31] | 0 | 0 | 0 | 0 | 0 | 0 | 0 | 0 | 0 | 0 | 0 | 0 | 0 | 0 | 0 | 0 | 0 | 1 | 0 | 2 | 0 | 0 | 0 | 0 | 0 | 0 | 3 | 4 | 5 | 0 | 0 | 0 | 0 | 0 |
| Banitalebi 2019[333] | 0 | 0 | 0 | 0 | 0 | 0 | 0 | 0 | 0 | 0 | 0 | 0 | 0 | 0 | 0 | 0 | 0 | 0 | 0 | 0 | 0 | 0 | 0 | 0 | 0 | 0 | 0 | 0 | 0 | 0 | 0 | 0 | 1 | 0 |
| Beavers 2013[225] | 0 | 0 | 0 | 0 | 0 | 0 | 0 | 0 | 0 | 0 | 0 | 0 | 0 | 0 | 0 | 0 | 0 | 0 | 0 | 0 | 1 | 0 | 0 | 0 | 0 | 0 | 0 | 0 | 0 | 0 | 0 | 0 | 0 | 0 |
| Ben Ounis 2009[289] | 1 | 0 | 0 | 0 | 0 | 0 | 0 | 0 | 0 | 0 | 0 | 0 | 0 | 0 | 0 | 0 | 0 | 0 | 0 | 0 | 0 | 0 | 0 | 0 | 0 | 0 | 0 | 0 | 0 | 0 | 0 | 0 | 0 | 0 |
| Ben Ounis 2010[34] | 1 | 0 | 0 | 0 | 0 | 0 | 0 | 0 | 0 | 0 | 0 | 0 | 0 | 0 | 0 | 0 | 0 | 0 | 0 | 0 | 0 | 0 | 0 | 0 | 0 | 0 | 0 | 0 | 0 | 0 | 0 | 1 | 0 | 0 |
| Bhati 2022[35] | 0 | 0 | 1 | 0 | 0 | 0 | 0 | 0 | 0 | 0 | 0 | 0 | 0 | 0 | 0 | 0 | 0 | 0 | 0 | 0 | 0 | 0 | 0 | 0 | 0 | 0 | 0 | 0 | 0 | 0 | 0 | 0 | 0 | 0 |
| Biteli 2021[290] | 0 | 0 | 0 | 0 | 0 | 0 | 0 | 0 | 0 | 0 | 0 | 0 | 0 | 0 | 1 | 0 | 0 | 0 | 0 | 0 | 0 | 0 | 0 | 0 | 0 | 0 | 0 | 0 | 0 | 0 | 0 | 0 | 0 | 0 |
| Bocca 2014[37] | 0 | 1 | 0 | 0 | 0 | 0 | 0 | 0 | 0 | 0 | 0 | 0 | 0 | 0 | 0 | 0 | 0 | 0 | 0 | 0 | 0 | 0 | 0 | 0 | 0 | 0 | 0 | 0 | 0 | 0 | 0 | 0 | 0 | 0 |
| Borfe 2021[334] | 0 | 1 | 0 | 0 | 0 | 0 | 0 | 0 | 0 | 0 | 0 | 0 | 0 | 0 | 0 | 0 | 0 | 0 | 0 | 0 | 0 | 0 | 0 | 0 | 0 | 0 | 0 | 0 | 0 | 0 | 0 | 0 | 0 | 0 |
| Brunelli 2015[42] | 0 | 0 | 0 | 0 | 0 | 0 | 0 | 1 | 0 | 0 | 0 | 0 | 0 | 0 | 0 | 0 | 0 | 0 | 0 | 0 | 0 | 0 | 0 | 0 | 0 | 0 | 0 | 0 | 0 | 0 | 0 | 0 | 0 | 0 |
| Byrkjeland 2011[43] | 0 | 0 | 0 | 0 | 0 | 0 | 0 | 0 | 0 | 0 | 0 | 0 | 0 | 1 | 0 | 0 | 0 | 0 | 0 | 0 | 0 | 0 | 0 | 0 | 0 | 0 | 0 | 0 | 0 | 0 | 0 | 0 | 0 | 0 |
| Campbell 2009[45] | 0 | 0 | 0 | 0 | 0 | 0 | 0 | 0 | 0 | 0 | 0 | 1 | 0 | 0 | 2 | 0 | 0 | 3 | 0 | 0 | 0 | 0 | 0 | 0 | 0 | 0 | 0 | 0 | 0 | 0 | 0 | 0 | 0 | 0 |
| Carrillo 2012[335] | 0 | 0 | 0 | 0 | 0 | 0 | 0 | 0 | 0 | 0 | 0 | 0 | 1 | 0 | 0 | 0 | 0 | 0 | 0 | 0 | 0 | 0 | 0 | 0 | 0 | 0 | 0 | 0 | 0 | 0 | 0 | 0 | 0 | 0 |
| Chagas 2017[291] | 0 | 0 | 0 | 0 | 0 | 0 | 0 | 0 | 0 | 0 | 0 | 0 | 0 | 0 | 1 | 0 | 0 | 0 | 0 | 2 | 0 | 0 | 0 | 0 | 0 | 0 | 0 | 0 | 0 | 0 | 0 | 0 | 0 | 0 |
| Chen 2015[47] | 0 | 0 | 0 | 0 | 0 | 0 | 0 | 0 | 0 | 0 | 0 | 1 | 2 | 0 | 0 | 0 | 0 | 0 | 0 | 0 | 0 | 3 | 4 | 0 | 0 | 0 | 0 | 0 | 0 | 0 | 0 | 0 | 0 | 0 |
| Choi 2012[49] | 0 | 0 | 0 | 0 | 0 | 0 | 0 | 0 | 0 | 0 | 0 | 0 | 0 | 0 | 0 | 0 | 0 | 1 | 0 | 0 | 0 | 0 | 0 | 0 | 0 | 0 | 0 | 0 | 0 | 0 | 0 | 0 | 0 | 0 |
| Chow 2021[292] | 0 | 0 | 0 | 0 | 0 | 0 | 0 | 0 | 0 | 0 | 0 | 1 | 0 | 0 | 0 | 0 | 0 | 0 | 0 | 0 | 0 | 0 | 0 | 0 | 0 | 0 | 0 | 0 | 0 | 0 | 0 | 0 | 0 | 0 |
| Christiansen 2010[50] | 0 | 0 | 0 | 0 | 0 | 0 | 0 | 0 | 0 | 0 | 0 | 1 | 0 | 0 | 0 | 0 | 0 | 1 | 0 | 0 | 0 | 0 | 0 | 0 | 0 | 0 | 0 | 0 | 0 | 0 | 1 | 0 | 0 | 0 |

| Primary study | Zalagkitis-1 2025 | Zalagkitis-2 2025 | Wang 2025 | Hernandez-Martinez 2025 | Tan-1 2025 (AE) | Tan-2 2025 (RT) | Tan-3 2025 (CE) | Silva 2024 | Li 2024 | Al-Mhanna-1 2024 | Al-Mhanna-2 2024 | Guo-1 (AE) | Guo-2 (RE) | Malandish 2023 | Tan-1 (AE) | Tan-2 (RT) | Tan-3 (CE) | Del Rosso-1 (AE) | Del Rosso-2 (RT) | Del Rosso-3 (AE+RT) | Rahimi 2022 | Zhao-1 (AT) | Zhao-2 (RT) | Zhao-3 (AT+RT) | Hejazi-1 (AE) | Hejazi-2 (RT) | Hejazi-3 (AE) | Hejazi-4 (RT) | Hejazi-5 (AE+RT) | Khalafi 2022 | Liu 2021 | Lee 2021 | Khalafi 2020 | Sirico 2018 |
|---|---|---|---|---|---|---|---|---|---|---|---|---|---|---|---|---|---|---|---|---|---|---|---|---|---|---|---|---|---|---|---|---|---|---|
| Chupel 2018[293] | 0 | 0 | 0 | 0 | 0 | 0 | 0 | 0 | 0 | 0 | 0 | 0 | 0 | 0 | 0 | 0 | 1 | 0 | 0 | 0 | 0 | 0 | 0 | 0 | 0 | 0 | 0 | 0 | 0 | 0 | 0 | 0 | 0 | 0 |
| Cobos-Palacios 2022[52] | 0 | 1 | 0 | 0 | 0 | 0 | 0 | 0 | 0 | 0 | 0 | 0 | 0 | 0 | 0 | 0 | 0 | 0 | 0 | 0 | 0 | 0 | 0 | 0 | 0 | 0 | 0 | 0 | 0 | 0 | 0 | 0 | 0 | 0 |
| Conraads 2002[295] | 0 | 0 | 0 | 0 | 0 | 0 | 0 | 0 | 0 | 0 | 0 | 0 | 0 | 1 | 0 | 0 | 0 | 0 | 0 | 0 | 0 | 0 | 0 | 0 | 0 | 0 | 0 | 0 | 0 | 0 | 0 | 0 | 0 | 0 |
| de Meirelles 2014[58] | 0 | 0 | 0 | 0 | 0 | 0 | 0 | 0 | 0 | 0 | 0 | 0 | 0 | 1 | 0 | 0 | 0 | 0 | 0 | 0 | 0 | 0 | 0 | 0 | 0 | 0 | 0 | 0 | 0 | 0 | 0 | 0 | 0 | 0 |
| Dieli-Conwright 2018a[59] | 0 | 0 | 0 | 0 | 0 | 0 | 1 | 0 | 0 | 0 | 0 | 0 | 0 | 0 | 0 | 0 | 0 | 0 | 0 | 0 | 0 | 0 | 0 | 0 | 0 | 0 | 0 | 0 | 0 | 0 | 0 | 0 | 0 | 0 |
| Dieli-Conwright 2018b[230] | 0 | 0 | 0 | 1 | 0 | 0 | 2 | 0 | 0 | 3 | 0 | 0 | 0 | 0 | 0 | 0 | 0 | 0 | 0 | 0 | 0 | 0 | 0 | 0 | 0 | 0 | 0 | 0 | 0 | 0 | 0 | 0 | 0 | 0 |
| Donges 2013[60] | 0 | 0 | 0 | 0 | 0 | 0 | 0 | 1 | 0 | 0 | 0 | 0 | 0 | 0 | 0 | 0 | 0 | 2 | 3 | 4 | 0 | 0 | 0 | 0 | 0 | 0 | 0 | 0 | 0 | 0 | 0 | 0 | 0 | 0 |
| Eleuteri 2013[61] | 0 | 0 | 0 | 0 | 0 | 0 | 0 | 0 | 0 | 0 | 0 | 0 | 0 | 1 | 0 | 0 | 0 | 0 | 0 | 0 | 0 | 0 | 0 | 0 | 0 | 0 | 0 | 0 | 0 | 0 | 0 | 0 | 0 | 0 |
| Ergun 2013[297] | 0 | 0 | 0 | 0 | 0 | 0 | 0 | 0 | 0 | 1 | 0 | 0 | 0 | 0 | 0 | 0 | 0 | 0 | 0 | 0 | 0 | 0 | 0 | 0 | 0 | 0 | 0 | 0 | 0 | 0 | 0 | 0 | 0 | 0 |
| Feiereisen 2013[298] | 0 | 0 | 0 | 0 | 0 | 0 | 0 | 0 | 0 | 0 | 0 | 0 | 0 | 1 | 0 | 0 | 0 | 0 | 0 | 0 | 0 | 0 | 0 | 0 | 0 | 0 | 0 | 0 | 0 | 0 | 0 | 0 | 0 | 0 |
| Fernandes-Silva 2017[299] | 0 | 0 | 0 | 0 | 0 | 0 | 0 | 0 | 0 | 0 | 0 | 0 | 0 | 1 | 0 | 0 | 0 | 0 | 0 | 0 | 0 | 0 | 0 | 0 | 0 | 0 | 0 | 0 | 0 | 0 | 0 | 0 | 0 | 0 |
| Fisher 2011[65] | 0 | 0 | 0 | 0 | 0 | 0 | 0 | 0 | 0 | 0 | 0 | 0 | 0 | 0 | 0 | 0 | 0 | 1 | 2 | 0 | 0 | 0 | 0 | 0 | 0 | 0 | 0 | 0 | 0 | 0 | 3 | 0 | 0 | 0 |
| Friedenreich 2012[68] | 0 | 0 | 0 | 0 | 0 | 0 | 0 | 0 | 0 | 0 | 0 | 0 | 0 | 0 | 0 | 0 | 0 | 1 | 0 | 0 | 0 | 0 | 0 | 0 | 0 | 0 | 0 | 0 | 0 | 0 | 0 | 0 | 0 | 0 |
| Fu 2013[336] | 0 | 0 | 0 | 0 | 0 | 0 | 0 | 0 | 0 | 0 | 0 | 0 | 0 | 1 | 0 | 0 | 0 | 0 | 0 | 0 | 0 | 0 | 0 | 0 | 0 | 0 | 0 | 0 | 0 | 0 | 0 | 0 | 0 | 0 |
| Gallardo-Escribano 2020[337] | 0 | 1 | 0 | 0 | 0 | 0 | 0 | 0 | 0 | 0 | 0 | 0 | 0 | 0 | 0 | 0 | 0 | 0 | 0 | 0 | 0 | 0 | 0 | 0 | 0 | 0 | 0 | 0 | 0 | 0 | 0 | 0 | 0 | 0 |
| Gallistl 2001[338] | 1 | 0 | 0 | 0 | 0 | 0 | 0 | 0 | 0 | 0 | 0 | 0 | 0 | 0 | 0 | 0 | 0 | 0 | 0 | 0 | 0 | 0 | 0 | 0 | 0 | 0 | 0 | 0 | 0 | 0 | 0 | 0 | 0 | 0 |
| Garanty-Bogacka 2011[69] | 1 | 0 | 0 | 0 | 0 | 0 | 0 | 0 | 0 | 0 | 0 | 0 | 0 | 0 | 0 | 0 | 0 | 0 | 0 | 0 | 0 | 0 | 0 | 0 | 0 | 0 | 0 | 0 | 0 | 0 | 0 | 0 | 0 | 0 |
| García-Unciti 2012[339] | 0 | 0 | 0 | 0 | 0 | 0 | 0 | 0 | 0 | 0 | 0 | 0 | 0 | 0 | 0 | 0 | 0 | 0 | 0 | 0 | 0 | 0 | 0 | 0 | 0 | 0 | 0 | 0 | 0 | 0 | 1 | 0 | 0 | 0 |
| Giannopoulou 2005[71] | 0 | 0 | 0 | 0 | 0 | 0 | 0 | 0 | 0 | 0 | 0 | 0 | 0 | 0 | 0 | 0 | 0 | 0 | 0 | 0 | 0 | 0 | 0 | 0 | 0 | 0 | 0 | 0 | 0 | 1 | 2 | 0 | 0 | 0 |
| Gokulakrishnan 2017[74] | 0 | 0 | 0 | 0 | 0 | 0 | 0 | 0 | 0 | 0 | 0 | 0 | 0 | 0 | 0 | 0 | 0 | 0 | 0 | 0 | 0 | 0 | 0 | 0 | 0 | 0 | 1 | 0 | 0 | 0 | 0 | 0 | 0 | 0 |
| Gong 2014[73] | 0 | 1 | 0 | 0 | 0 | 0 | 0 | 0 | 0 | 0 | 0 | 0 | 0 | 0 | 0 | 0 | 0 | 0 | 0 | 0 | 0 | 0 | 0 | 0 | 0 | 0 | 0 | 0 | 0 | 0 | 0 | 0 | 0 | 0 |
| Hagstrom 2016[75] | 0 | 0 | 0 | 0 | 0 | 1 | 0 | 0 | 0 | 0 | 0 | 0 | 0 | 0 | 0 | 0 | 0 | 0 | 0 | 0 | 0 | 0 | 0 | 0 | 0 | 0 | 0 | 0 | 0 | 0 | 0 | 0 | 0 | 0 |
| Henriquez 2017[76] | 0 | 0 | 0 | 0 | 0 | 0 | 0 | 0 | 0 | 0 | 0 | 0 | 0 | 0 | 0 | 0 | 0 | 1 | 2 | 0 | 0 | 0 | 0 | 0 | 0 | 0 | 0 | 0 | 0 | 0 | 0 | 0 | 0 | 0 |
| Herder 2009[77] | 0 | 0 | 0 | 0 | 0 | 0 | 0 | 0 | 0 | 0 | 0 | 0 | 0 | 0 | 0 | 0 | 0 | 1 | 0 | 0 | 2 | 0 | 0 | 0 | 0 | 0 | 0 | 0 | 0 | 0 | 0 | 0 | 0 | 0 |
| Ho 2013[302] | 0 | 0 | 0 | 0 | 0 | 0 | 0 | 0 | 0 | 0 | 0 | 1 | 0 | 0 | 0 | 0 | 0 | 0 | 0 | 0 | 0 | 0 | 0 | 0 | 0 | 0 | 0 | 0 | 0 | 0 | 0 | 0 | 0 | 0 |
| Hutnick 2005[340] | 0 | 0 | 0 | 0 | 0 | 0 | 0 | 0 | 0 | 1 | 0 | 0 | 0 | 0 | 0 | 0 | 0 | 0 | 0 | 0 | 0 | 0 | 0 | 0 | 0 | 0 | 0 | 0 | 0 | 0 | 0 | 0 | 0 | 0 |
| Imayama 2012[78] | 0 | 0 | 0 | 0 | 0 | 0 | 0 | 0 | 0 | 0 | 0 | 1 | 0 | 0 | 2 | 0 | 0 | 3 | 0 | 0 | 0 | 0 | 0 | 0 | 0 | 0 | 0 | 0 | 0 | 0 | 0 | 0 | 0 | 0 |
| Izadpanah 2012[341] | 1 | 0 | 0 | 0 | 0 | 0 | 0 | 0 | 0 | 0 | 0 | 0 | 0 | 0 | 0 | 0 | 0 | 0 | 0 | 0 | 0 | 0 | 0 | 0 | 0 | 0 | 0 | 0 | 0 | 0 | 0 | 0 | 0 | 0 |
| Johannsen 2012[80] | 0 | 0 | 0 | 0 | 0 | 0 | 0 | 0 | 0 | 0 | 0 | 0 | 0 | 0 | 1 | 0 | 0 | 0 | 0 | 0 | 0 | 0 | 0 | 0 | 2 | 0 | 0 | 0 | 0 | 0 | 0 | 0 | 0 | 0 |
| Jones 2013[81] | 0 | 0 | 0 | 0 | 1 | 0 | 0 | 0 | 0 | 0 | 0 | 0 | 0 | 0 | 0 | 0 | 0 | 0 | 0 | 0 | 0 | 0 | 0 | 0 | 0 | 0 | 0 | 0 | 0 | 0 | 0 | 0 | 0 | 0 |
| Jorge 2011[82] | 0 | 0 | 1 | 0 | 0 | 0 | 0 | 0 | 0 | 0 | 2 | 0 | 0 | 0 | 0 | 0 | 0 | 3 | 4 | 5 | 0 | 0 | 0 | 0 | 0 | 0 | 6 | 7 | 8 | 0 | 0 | 0 | 0 | 0 |

| Primary study | Zalagkitis-1 2025 | Zalagkitis-2 2025 | Wang 2025 | Hernandez-Martinez 2025 | Tan-1 2025 (AE) | Tan-2 2025 (RT) | Tan-3 2025 (CE) | Silva 2024 | Li 2024 | Al-Mhanna-1 2024 | Al-Mhanna-2 2024 | Guo-1 (AE) | Guo-2 (RE) | Malandish 2023 | Tan-1 (AE) | Tan-2 (RT) | Tan-3 (CE) | Del Rosso-1 (AE) | Del Rosso-2 (RT) | Del Rosso-3 (AE+RT) | Rahimi 2022 | Zhao-1 (AT) | Zhao-2 (RT) | Zhao-3 (AT+RT) | Hejazi-1 (AE) | Hejazi-2 (RT) | Hejazi-3 (AE) | Hejazi-4 (RT) | Hejazi-5 (AE+RT) | Khalafi 2022 | Liu 2021 | Lee 2021 | Khalafi 2020 | Sirico 2018 |
|---|---|---|---|---|---|---|---|---|---|---|---|---|---|---|---|---|---|---|---|---|---|---|---|---|---|---|---|---|---|---|---|---|---|---|
| Kadoglou 2007[240] | 0 | 0 | 0 | 0 | 0 | 0 | 0 | 0 | 0 | 0 | 0 | 0 | 0 | 0 | 0 | 0 | 0 | 0 | 0 | 0 | 0 | 0 | 0 | 0 | 0 | 0 | 1 | 0 | 0 | 0 | 0 | 0 | 0 | 0 |
| Karavidas 2006[303] | 0 | 0 | 0 | 0 | 0 | 0 | 0 | 0 | 0 | 0 | 0 | 0 | 0 | 1 | 0 | 0 | 0 | 0 | 0 | 0 | 0 | 0 | 0 | 0 | 0 | 0 | 0 | 0 | 0 | 0 | 0 | 0 | 0 | 0 |
| Kelly 2007[89] | 0 | 0 | 0 | 0 | 0 | 0 | 0 | 0 | 1 | 0 | 0 | 2 | 0 | 0 | 0 | 0 | 0 | 0 | 0 | 0 | 0 | 0 | 0 | 0 | 0 | 0 | 0 | 0 | 0 | 0 | 0 | 0 | 0 | 0 |
| Kim 2007[91] | 0 | 0 | 0 | 0 | 0 | 0 | 0 | 0 | 0 | 0 | 0 | 1 | 0 | 0 | 0 | 0 | 0 | 0 | 0 | 0 | 0 | 2 | 0 | 0 | 0 | 0 | 0 | 0 | 0 | 0 | 0 | 0 | 0 | 0 |
| Kim 2008[92] | 0 | 0 | 0 | 0 | 0 | 0 | 0 | 0 | 0 | 0 | 0 | 0 | 0 | 1 | 0 | 0 | 0 | 0 | 0 | 0 | 0 | 0 | 0 | 0 | 0 | 0 | 0 | 0 | 0 | 0 | 0 | 0 | 0 | 0 |
| Kim 2016[94] | 0 | 0 | 0 | 1 | 0 | 0 | 0 | 0 | 0 | 0 | 0 | 0 | 0 | 0 | 0 | 0 | 0 | 0 | 0 | 0 | 0 | 0 | 0 | 0 | 0 | 0 | 0 | 0 | 0 | 0 | 0 | 0 | 0 | 0 |
| Kobayashi 2003[342] | 0 | 0 | 0 | 0 | 0 | 0 | 0 | 0 | 0 | 0 | 0 | 0 | 0 | 1 | 0 | 0 | 0 | 0 | 0 | 0 | 0 | 0 | 0 | 0 | 0 | 0 | 0 | 0 | 0 | 0 | 0 | 0 | 0 | 0 |
| Lakhdar 2013[304] | 0 | 0 | 0 | 0 | 0 | 0 | 0 | 0 | 0 | 0 | 0 | 0 | 0 | 0 | 0 | 0 | 0 | 1 | 0 | 0 | 0 | 0 | 0 | 0 | 0 | 0 | 0 | 0 | 0 | 0 | 2 | 0 | 0 | 0 |
| Lam 2016[98] | 0 | 0 | 0 | 0 | 0 | 0 | 0 | 0 | 0 | 0 | 0 | 0 | 0 | 0 | 0 | 0 | 0 | 0 | 0 | 0 | 0 | 0 | 0 | 0 | 0 | 0 | 0 | 0 | 0 | 1 | 2 | 0 | 0 | 0 |
| Lambert 2008[99] | 0 | 0 | 0 | 0 | 0 | 0 | 0 | 0 | 0 | 0 | 0 | 0 | 0 | 0 | 0 | 0 | 0 | 0 | 0 | 0 | 0 | 0 | 0 | 0 | 0 | 0 | 0 | 0 | 0 | 1 | 0 | 0 | 0 | 0 |
| Larsen 2001[305] | 0 | 0 | 0 | 0 | 0 | 0 | 0 | 0 | 0 | 0 | 0 | 0 | 0 | 1 | 0 | 0 | 0 | 0 | 0 | 0 | 0 | 0 | 0 | 0 | 0 | 0 | 0 | 0 | 0 | 0 | 0 | 0 | 0 | 0 |
| Lee 2012[101] | 0 | 0 | 0 | 0 | 0 | 0 | 0 | 0 | 0 | 0 | 0 | 1 | 0 | 0 | 0 | 0 | 0 | 0 | 0 | 0 | 0 | 0 | 0 | 0 | 0 | 0 | 0 | 0 | 0 | 0 | 0 | 0 | 0 | 0 |
| Libardi 2012[102] | 0 | 0 | 0 | 0 | 0 | 0 | 0 | 1 | 0 | 0 | 0 | 0 | 0 | 0 | 0 | 0 | 0 | 0 | 0 | 0 | 0 | 0 | 0 | 0 | 0 | 0 | 0 | 0 | 0 | 0 | 0 | 0 | 0 | 0 |
| Ligibel 2019[103] | 0 | 0 | 0 | 0 | 0 | 0 | 1 | 0 | 0 | 2 | 0 | 0 | 0 | 0 | 0 | 0 | 0 | 0 | 0 | 0 | 0 | 0 | 0 | 0 | 0 | 0 | 0 | 0 | 0 | 0 | 0 | 0 | 0 | 0 |
| Lira 2011[343] | 0 | 1 | 0 | 0 | 0 | 0 | 0 | 0 | 0 | 0 | 0 | 0 | 0 | 0 | 0 | 0 | 0 | 0 | 0 | 0 | 0 | 0 | 0 | 0 | 0 | 0 | 0 | 0 | 0 | 0 | 0 | 0 | 0 | 0 |
| Liu 2015[104] | 1 | 0 | 0 | 0 | 0 | 0 | 0 | 0 | 0 | 0 | 0 | 0 | 0 | 0 | 0 | 0 | 0 | 0 | 0 | 0 | 0 | 0 | 0 | 0 | 0 | 0 | 0 | 0 | 0 | 0 | 0 | 0 | 0 | 0 |
| Liu 2018[105] | 1 | 0 | 0 | 0 | 0 | 0 | 0 | 0 | 2 | 0 | 0 | 0 | 0 | 0 | 0 | 0 | 0 | 0 | 0 | 0 | 0 | 0 | 0 | 0 | 0 | 0 | 0 | 0 | 0 | 0 | 0 | 0 | 0 | 0 |
| Lopes 2016[106] | 0 | 0 | 0 | 0 | 0 | 0 | 0 | 0 | 0 | 0 | 0 | 0 | 0 | 0 | 0 | 0 | 0 | 0 | 0 | 0 | 0 | 0 | 0 | 1 | 0 | 0 | 0 | 0 | 0 | 0 | 0 | 0 | 0 | 0 |
| Loria-Kohen 2013[107] | 0 | 0 | 0 | 0 | 0 | 0 | 0 | 0 | 0 | 0 | 0 | 0 | 0 | 0 | 0 | 0 | 0 | 1 | 2 | 3 | 0 | 0 | 0 | 0 | 0 | 0 | 0 | 0 | 0 | 0 | 0 | 0 | 0 | 0 |
| Magalhaes 2020[109] | 0 | 0 | 0 | 0 | 0 | 0 | 0 | 0 | 0 | 0 | 0 | 0 | 0 | 0 | 0 | 0 | 0 | 1 | 0 | 0 | 0 | 0 | 0 | 0 | 0 | 0 | 0 | 0 | 0 | 0 | 0 | 0 | 0 | 0 |
| Martins 2018[113] | 0 | 0 | 0 | 0 | 0 | 0 | 0 | 0 | 0 | 0 | 0 | 0 | 0 | 0 | 0 | 0 | 0 | 1 | 0 | 2 | 0 | 0 | 0 | 0 | 0 | 0 | 0 | 0 | 0 | 0 | 0 | 0 | 0 | 0 |
| Martins 2023[307] | 0 | 0 | 0 | 0 | 0 | 1 | 0 | 0 | 0 | 0 | 0 | 0 | 0 | 0 | 0 | 0 | 0 | 0 | 0 | 0 | 0 | 0 | 0 | 0 | 0 | 0 | 0 | 0 | 0 | 0 | 0 | 0 | 0 | 0 |
| Masquio 2023[114] | 0 | 1 | 0 | 0 | 0 | 0 | 0 | 0 | 0 | 0 | 0 | 0 | 0 | 0 | 0 | 0 | 0 | 0 | 0 | 0 | 0 | 0 | 0 | 0 | 0 | 0 | 0 | 0 | 0 | 0 | 0 | 0 | 0 | 0 |
| McDermott 2004[118] | 0 | 0 | 0 | 0 | 0 | 0 | 0 | 0 | 0 | 0 | 0 | 0 | 0 | 1 | 0 | 0 | 0 | 0 | 0 | 0 | 0 | 0 | 0 | 0 | 0 | 0 | 0 | 0 | 0 | 0 | 0 | 0 | 0 | 0 |
| Melo 2019[308] | 0 | 0 | 0 | 0 | 0 | 0 | 0 | 0 | 0 | 0 | 0 | 0 | 0 | 1 | 0 | 0 | 0 | 0 | 0 | 0 | 0 | 0 | 0 | 0 | 0 | 0 | 0 | 0 | 0 | 0 | 0 | 0 | 0 | 0 |
| Mendez-Gutierrez 2022[250] | 0 | 0 | 0 | 0 | 0 | 0 | 0 | 1 | 0 | 0 | 0 | 0 | 0 | 0 | 0 | 0 | 0 | 0 | 0 | 0 | 0 | 0 | 0 | 0 | 0 | 0 | 0 | 0 | 0 | 0 | 0 | 0 | 0 | 0 |
| Mendham 2014[251] | 0 | 0 | 0 | 0 | 0 | 0 | 0 | 0 | 0 | 0 | 0 | 0 | 0 | 0 | 0 | 0 | 0 | 1 | 0 | 0 | 0 | 0 | 0 | 0 | 0 | 0 | 2 | 0 | 0 | 0 | 0 | 0 | 0 | 0 |
| Mendham 2015[119] | 0 | 0 | 0 | 0 | 0 | 0 | 0 | 0 | 0 | 0 | 0 | 0 | 0 | 0 | 0 | 0 | 0 | 0 | 0 | 1 | 0 | 0 | 0 | 0 | 0 | 0 | 0 | 0 | 0 | 0 | 0 | 0 | 0 | 0 |
| Moore 2021[344] | 0 | 1 | 0 | 0 | 0 | 0 | 0 | 0 | 0 | 0 | 0 | 0 | 0 | 0 | 0 | 0 | 0 | 0 | 0 | 0 | 0 | 0 | 0 | 0 | 0 | 0 | 0 | 0 | 0 | 0 | 0 | 0 | 0 | 0 |
| Munk 2011[309] | 0 | 0 | 0 | 0 | 0 | 0 | 0 | 0 | 0 | 0 | 0 | 0 | 0 | 1 | 0 | 0 | 0 | 0 | 0 | 0 | 0 | 0 | 0 | 0 | 0 | 0 | 0 | 0 | 0 | 0 | 0 | 0 | 0 | 0 |
| Murphy 2009[128] | 0 | 0 | 0 | 0 | 0 | 0 | 0 | 0 | 1 | 0 | 0 | 0 | 0 | 0 | 0 | 0 | 0 | 0 | 0 | 0 | 0 | 0 | 0 | 0 | 0 | 0 | 0 | 0 | 0 | 0 | 0 | 2 | 0 | 0 |
| Nambi 2022[253] | 1 | 0 | 0 | 0 | 0 | 0 | 0 | 0 | 2 | 0 | 0 | 0 | 0 | 0 | 0 | 0 | 0 | 0 | 0 | 0 | 0 | 0 | 0 | 0 | 0 | 0 | 0 | 0 | 0 | 0 | 0 | 0 | 0 | 0 |
| Nemet 2013[345] | 1 | 0 | 0 | 0 | 0 | 0 | 0 | 0 | 0 | 0 | 0 | 0 | 0 | 0 | 0 | 0 | 0 | 0 | 0 | 0 | 0 | 0 | 0 | 0 | 0 | 0 | 0 | 0 | 0 | 0 | 0 | 0 | 0 | 0 |

| Primary study | Zalag kitis-1 2025 | Zalag kitis-2 2025 | Wa ng 202 5 | Hernand ez-Marti nez 2025 | Tan-1 2025 (AE) | Tan-2 2025 (RT) | Tan-3 2025 (CE) | Sil va 202 4 | Li 20 24 | Al-Mh anna-1 2024 | Al-Mh anna-2 2024 | Gu o-1 (AE ) | Gu o-2 (RE ) | Mala ndish 2023 | Tan -1 (AE ) | Tan -2 (RT ) | Tan -3 (CE ) | Del Rosso -1 (AE) | Del Rosso -2 (RT) | Del Rosso-3 (AE+R T) | Rah imi 202 2 | Zha o-1 (AT ) | Zha o-2 (RT ) | Zhao- 3 (AT+ RT) | Heja zi-1 (AE) | Heja zi-2 (RT) | Heja zi-3 (AE) | Heja zi-4 (RT) | Hejazi -5 (AE+ RT) | Kha lafi 2022 | Li u 20 21 | Le e 20 21 | Kha lafi 202 0 | Sir ico 20 18 |
|---|---|---|---|---|---|---|---|---|---|---|---|---|---|---|---|---|---|---|---|---|---|---|---|---|---|---|---|---|---|---|---|---|---|---|
| Nicklas 2004[132] | 0 | 0 | 0 | 0 | 0 | 0 | 0 | 0 | 0 | 0 | 0 | 0 | 0 | 0 | 0 | 0 | 0 | 0 | 0 | 0 | 0 | 0 | 0 | 0 | 0 | 0 | 0 | 0 | 0 | 0 | 2 | 0 | 0 | 0 |
| Niebauer 2005[310] | 0 | 0 | 0 | 0 | 0 | 0 | 0 | 0 | 0 | 0 | 0 | 0 | 0 | 1 | 0 | 0 | 0 | 0 | 0 | 0 | 0 | 0 | 0 | 0 | 0 | 0 | 0 | 0 | 0 | 0 | 0 | 0 | 0 | 0 |
| Nikseresht 2014[254] | 0 | 0 | 0 | 0 | 0 | 0 | 0 | 0 | 0 | 0 | 0 | 0 | 0 | 0 | 0 | 0 | 0 | 0 | 0 | 0 | 0 | 0 | 0 | 0 | 1 | 2 | 0 | 0 | 0 | 0 | 0 | 0 | 3 | 0 |
| Nikseresht 2018[346] | 0 | 0 | 0 | 0 | 0 | 0 | 0 | 0 | 0 | 0 | 0 | 0 | 0 | 0 | 0 | 0 | 0 | 0 | 1 | 0 | 0 | 0 | 0 | 0 | 0 | 0 | 0 | 0 | 0 | 0 | 0 | 0 | 0 | 0 |
| Nunes 2016[136] | 0 | 0 | 0 | 0 | 0 | 0 | 0 | 0 | 0 | 0 | 0 | 0 | 0 | 0 | 0 | 1 | 0 | 0 | 2 | 0 | 0 | 0 | 0 | 0 | 0 | 0 | 0 | 0 | 0 | 0 | 0 | 0 | 0 | 0 |
| Oh 2014[138] | 0 | 0 | 0 | 0 | 0 | 0 | 0 | 0 | 0 | 0 | 0 | 0 | 0 | 0 | 0 | 0 | 0 | 0 | 0 | 0 | 0 | 0 | 0 | 0 | 0 | 0 | 0 | 0 | 0 | 0 | 1 | 0 | 0 | 0 |
| Olson 2007[140] | 0 | 0 | 0 | 0 | 0 | 0 | 0 | 0 | 0 | 0 | 0 | 0 | 0 | 0 | 0 | 0 | 0 | 0 | 1 | 0 | 0 | 0 | 0 | 0 | 0 | 2 | 0 | 0 | 0 | 0 | 0 | 0 | 0 | 0 |
| Peña 2023[312] | 0 | 1 | 0 | 0 | 0 | 0 | 0 | 0 | 2 | 0 | 0 | 0 | 0 | 0 | 0 | 0 | 0 | 0 | 0 | 0 | 0 | 0 | 0 | 0 | 0 | 0 | 0 | 0 | 0 | 0 | 0 | 0 | 0 | 0 |
| Pérez-López 2022[146] | 0 | 0 | 0 | 0 | 0 | 0 | 0 | 1 | 0 | 0 | 0 | 0 | 0 | 0 | 0 | 0 | 0 | 0 | 0 | 0 | 0 | 0 | 0 | 0 | 0 | 0 | 0 | 0 | 0 | 0 | 0 | 0 | 0 | 0 |
| Phillips 2012[147] | 0 | 0 | 0 | 0 | 0 | 0 | 0 | 0 | 0 | 0 | 0 | 0 | 0 | 0 | 0 | 0 | 0 | 0 | 1 | 0 | 0 | 0 | 0 | 0 | 0 | 2 | 0 | 0 | 0 | 0 | 0 | 0 | 0 | 0 |
| Pierce 2008[148] | 0 | 0 | 0 | 0 | 0 | 0 | 0 | 0 | 0 | 0 | 0 | 0 | 0 | 1 | 0 | 0 | 0 | 0 | 0 | 0 | 0 | 0 | 0 | 0 | 0 | 0 | 0 | 0 | 0 | 0 | 0 | 0 | 0 | 0 |
| Prescott 2009[151] | 0 | 0 | 0 | 0 | 0 | 0 | 0 | 0 | 0 | 0 | 0 | 0 | 0 | 1 | 0 | 0 | 0 | 0 | 0 | 0 | 0 | 0 | 0 | 0 | 0 | 0 | 0 | 0 | 0 | 0 | 0 | 0 | 0 | 0 |
| Pullen 2008[152] | 0 | 0 | 0 | 0 | 0 | 0 | 0 | 0 | 0 | 0 | 0 | 0 | 0 | 1 | 0 | 0 | 0 | 0 | 0 | 0 | 0 | 0 | 0 | 0 | 0 | 0 | 0 | 0 | 0 | 0 | 0 | 0 | 0 | 0 |
| Racca 2020[313] | 0 | 0 | 0 | 0 | 0 | 0 | 0 | 0 | 0 | 0 | 0 | 0 | 0 | 1 | 0 | 0 | 0 | 0 | 0 | 0 | 0 | 0 | 0 | 0 | 0 | 0 | 0 | 0 | 0 | 0 | 0 | 0 | 0 | 0 |
| Rech 2019[156] | 0 | 0 | 1 | 0 | 0 | 0 | 0 | 0 | 0 | 0 | 0 | 0 | 0 | 0 | 0 | 0 | 0 | 0 | 0 | 0 | 0 | 0 | 0 | 0 | 0 | 0 | 0 | 0 | 0 | 0 | 0 | 0 | 0 | 0 |
| Redwine 2020[157] | 0 | 0 | 0 | 0 | 0 | 0 | 0 | 0 | 0 | 0 | 0 | 0 | 0 | 1 | 0 | 0 | 0 | 0 | 0 | 0 | 0 | 0 | 0 | 0 | 0 | 0 | 0 | 0 | 0 | 0 | 0 | 0 | 0 | 0 |
| Rejeski 2019[159] | 0 | 0 | 0 | 0 | 0 | 0 | 0 | 0 | 0 | 0 | 0 | 0 | 0 | 0 | 0 | 0 | 0 | 1 | 2 | 0 | 0 | 0 | 0 | 0 | 0 | 0 | 0 | 0 | 0 | 0 | 3 | 0 | 0 | 0 |
| Reljic 2022[160] | 0 | 0 | 0 | 0 | 0 | 0 | 0 | 0 | 0 | 0 | 0 | 0 | 0 | 0 | 0 | 0 | 0 | 1 | 2 | 0 | 0 | 0 | 0 | 0 | 0 | 0 | 0 | 0 | 0 | 0 | 0 | 0 | 0 | 0 |
| Rogers 2013[278] | 0 | 0 | 0 | 0 | 0 | 0 | 1 | 0 | 0 | 0 | 0 | 0 | 0 | 0 | 0 | 0 | 0 | 0 | 0 | 0 | 0 | 0 | 0 | 0 | 0 | 0 | 0 | 0 | 0 | 0 | 0 | 0 | 0 | 0 |
| Rogers 2014[279] | 0 | 0 | 0 | 0 | 0 | 0 | 1 | 0 | 0 | 2 | 0 | 0 | 0 | 0 | 0 | 0 | 0 | 0 | 0 | 0 | 0 | 0 | 0 | 0 | 0 | 0 | 0 | 0 | 0 | 0 | 0 | 0 | 0 | 0 |
| Rokling-Andersen 2007[165] | 0 | 0 | 0 | 0 | 0 | 0 | 0 | 0 | 0 | 0 | 0 | 0 | 0 | 0 | 0 | 0 | 0 | 0 | 0 | 1 | 0 | 0 | 0 | 0 | 0 | 0 | 2 | 0 | 0 | 0 | 0 | 0 | 0 | 0 |
| Romeo 2011[328] | 0 | 1 | 0 | 0 | 0 | 0 | 0 | 0 | 0 | 0 | 0 | 0 | 0 | 0 | 0 | 0 | 0 | 0 | 0 | 0 | 0 | 0 | 0 | 0 | 0 | 0 | 0 | 0 | 0 | 0 | 0 | 0 | 0 | 0 |
| Ryan 2014[166] | 0 | 0 | 0 | 0 | 0 | 0 | 0 | 0 | 0 | 0 | 0 | 0 | 0 | 0 | 0 | 0 | 0 | 0 | 0 | 0 | 0 | 0 | 0 | 0 | 0 | 0 | 0 | 0 | 0 | 1 | 0 | 0 | 0 | 0 |
| Sabouri 2021[167] | 0 | 0 | 1 | 0 | 0 | 0 | 0 | 0 | 0 | 0 | 0 | 0 | 0 | 0 | 0 | 0 | 0 | 0 | 0 | 0 | 0 | 0 | 0 | 0 | 0 | 0 | 0 | 0 | 0 | 0 | 0 | 0 | 0 | 0 |
| Salamat 2016[315] | 0 | 0 | 0 | 0 | 0 | 0 | 0 | 1 | 0 | 0 | 0 | 0 | 0 | 0 | 0 | 0 | 0 | 2 | 3 | 4 | 0 | 0 | 0 | 0 | 0 | 0 | 0 | 0 | 0 | 0 | 0 | 0 | 0 | 0 |
| Santiprabhob 2018[169] | 0 | 1 | 0 | 0 | 0 | 0 | 0 | 0 | 0 | 0 | 0 | 0 | 0 | 0 | 0 | 0 | 0 | 0 | 0 | 0 | 0 | 0 | 0 | 0 | 0 | 0 | 0 | 0 | 0 | 0 | 0 | 0 | 0 | 0 |
| Saxton 2014[316] | 0 | 0 | 0 | 0 | 0 | 0 | 0 | 0 | 0 | 1 | 0 | 0 | 0 | 0 | 0 | 0 | 0 | 0 | 0 | 0 | 0 | 0 | 0 | 0 | 0 | 0 | 0 | 0 | 0 | 0 | 0 | 0 | 0 | 0 |
| Shahram 2016[317] | 0 | 0 | 0 | 0 | 0 | 0 | 0 | 0 | 0 | 0 | 0 | 0 | 0 | 0 | 0 | 0 | 0 | 1 | 2 | 0 | 0 | 0 | 0 | 0 | 0 | 0 | 0 | 0 | 0 | 0 | 0 | 0 | 0 | 0 |
| Shalitin 2009[174] | 1 | 0 | 0 | 0 | 0 | 0 | 0 | 0 | 0 | 0 | 0 | 0 | 0 | 0 | 0 | 0 | 0 | 0 | 0 | 0 | 0 | 0 | 0 | 0 | 0 | 0 | 0 | 0 | 0 | 0 | 0 | 0 | 0 | 0 |
| Shin 2006[175] | 0 | 0 | 0 | 0 | 0 | 0 | 0 | 0 | 0 | 0 | 0 | 0 | 0 | 1 | 0 | 0 | 0 | 0 | 0 | 0 | 0 | 0 | 0 | 0 | 0 | 0 | 0 | 0 | 0 | 0 | 0 | 0 | 0 | 0 |
| Silverman 2009[318] | 0 | 0 | 0 | 0 | 0 | 0 | 0 | 0 | 0 | 0 | 0 | 0 | 0 | 0 | 1 | 0 | 0 | 2 | 0 | 0 | 0 | 0 | 0 | 0 | 0 | 0 | 0 | 0 | 0 | 3 | 4 | 0 | 0 | 0 |
| Snel 2011[177] | 0 | 0 | 0 | 0 | 0 | 0 | 0 | 0 | 0 | 0 | 0 | 0 | 0 | 0 | 0 | 0 | 0 | 0 | 0 | 0 | 0 | 0 | 0 | 0 | 0 | 0 | 0 | 0 | 0 | 1 | 2 | 0 | 0 | 0 |
| Straznicky 2010[179] | 0 | 0 | 0 | 0 | 0 | 0 | 0 | 0 | 0 | 0 | 0 | 0 | 0 | 0 | 0 | 0 | 0 | 0 | 0 | 0 | 0 | 0 | 0 | 0 | 0 | 0 | 0 | 0 | 0 | 1 | 0 | 0 | 0 | 0 |

| Primary study | Zalagkitis-1 2025 | Zalagkitis-2 2025 | Wang 2025 | Hernandez-Martinez 2025 | Tan-1 2025 (AE) | Tan-2 2025 (RT) | Tan-3 2025 (CE) | Silva 2024 | Li 2024 | Al-Mhanna-1 2024 | Al-Mhanna-2 2024 | Guo-1 (AE) | Guo-2 (RE) | Malandish 2023 | Tan-1 (AE) | Tan-2 (RT) | Tan-3 (CE) | Del Rosso-1 (AE) | Del Rosso-2 (RT) | Del Rosso-3 (AE+RT) | Rahimi 2022 | Zhao-1 (AT) | Zhao-2 (RT) | Zhao-3 (AT+RT) | Hejazi-1 (AE) | Hejazi-2 (RT) | Hejazi-3 (AE) | Hejazi-4 (RT) | Hejazi-5 (AE+RT) | Khalafi 2022 | Liu 2021 | Lee 2021 | Khalafi 2020 | Sirico 2018 |
|---|---|---|---|---|---|---|---|---|---|---|---|---|---|---|---|---|---|---|---|---|---|---|---|---|---|---|---|---|---|---|---|---|---|---|
| Streb 2022[181] | 0 | 0 | 0 | 0 | 0 | 0 | 0 | 1 | 0 | 0 | 0 | 0 | 0 | 0 | 0 | 0 | 0 | 0 | 0 | 0 | 0 | 0 | 0 | 0 | 0 | 0 | 0 | 0 | 0 | 0 | 0 | 0 | 0 | 0 |
| Tartibian 2015[186] | 0 | 0 | 0 | 0 | 0 | 0 | 0 | 0 | 0 | 0 | 0 | 0 | 0 | 0 | 1 | 0 | 0 | 0 | 0 | 0 | 0 | 0 | 0 | 0 | 0 | 0 | 0 | 0 | 0 | 0 | 0 | 0 | 0 | 0 |
| Tenório 2018[347] | 0 | 1 | 0 | 0 | 0 | 0 | 0 | 0 | 0 | 0 | 0 | 2 | 0 | 0 | 0 | 0 | 0 | 0 | 0 | 0 | 0 | 0 | 0 | 0 | 0 | 0 | 0 | 0 | 0 | 0 | 0 | 0 | 0 | 0 |
| Thompson 2010[187] | 0 | 0 | 0 | 0 | 0 | 0 | 0 | 0 | 0 | 0 | 0 | 0 | 0 | 0 | 0 | 0 | 0 | 1 | 0 | 0 | 0 | 0 | 0 | 0 | 0 | 0 | 0 | 0 | 0 | 0 | 0 | 0 | 0 | 0 |
| Tomeleri 2016[189] | 0 | 0 | 0 | 0 | 0 | 0 | 0 | 0 | 0 | 0 | 0 | 0 | 1 | 0 | 0 | 2 | 0 | 0 | 3 | 0 | 0 | 0 | 0 | 0 | 0 | 0 | 0 | 0 | 0 | 0 | 0 | 0 | 0 | 0 |
| Tomeleri 2018[190] | 0 | 0 | 0 | 0 | 0 | 0 | 0 | 0 | 0 | 0 | 0 | 0 | 0 | 0 | 0 | 1 | 0 | 0 | 2 | 0 | 0 | 0 | 0 | 0 | 0 | 0 | 0 | 0 | 0 | 0 | 0 | 0 | 0 | 0 |
| Trippel 2017[320] | 0 | 0 | 0 | 0 | 0 | 0 | 0 | 0 | 0 | 0 | 0 | 0 | 0 | 1 | 0 | 0 | 0 | 0 | 0 | 0 | 0 | 0 | 0 | 0 | 0 | 0 | 0 | 0 | 0 | 0 | 0 | 0 | 0 | 0 |
| Vasconcellos 2016[194] | 0 | 0 | 0 | 0 | 0 | 0 | 0 | 0 | 1 | 0 | 0 | 0 | 0 | 0 | 0 | 0 | 0 | 0 | 0 | 0 | 0 | 2 | 0 | 0 | 0 | 0 | 0 | 0 | 0 | 0 | 0 | 0 | 0 | 3 |
| Vasconcelos 2020[322] | 0 | 0 | 0 | 0 | 0 | 0 | 0 | 0 | 0 | 0 | 0 | 0 | 0 | 0 | 0 | 0 | 1 | 0 | 0 | 0 | 0 | 0 | 0 | 0 | 0 | 0 | 0 | 0 | 0 | 0 | 0 | 0 | 0 | 0 |
| Vella 2017[195] | 0 | 0 | 0 | 0 | 0 | 0 | 0 | 0 | 0 | 0 | 0 | 1 | 0 | 0 | 0 | 0 | 0 | 2 | 0 | 0 | 0 | 0 | 0 | 0 | 0 | 0 | 0 | 0 | 0 | 0 | 0 | 0 | 0 | 0 |
| Venojärvi 2013[196] | 0 | 0 | 0 | 0 | 0 | 0 | 0 | 0 | 0 | 0 | 0 | 0 | 0 | 0 | 0 | 0 | 0 | 0 | 0 | 0 | 0 | 0 | 0 | 0 | 1 | 2 | 0 | 0 | 0 | 0 | 0 | 0 | 0 | 0 |
| Wanderley 2013[200] | 0 | 0 | 0 | 0 | 0 | 0 | 0 | 0 | 0 | 0 | 0 | 0 | 0 | 0 | 0 | 0 | 0 | 1 | 2 | 0 | 0 | 0 | 0 | 0 | 0 | 0 | 0 | 0 | 0 | 0 | 0 | 0 | 0 | 0 |
| Wang 2006 [201] | 0 | 0 | 0 | 0 | 0 | 0 | 0 | 0 | 0 | 0 | 0 | 1 | 0 | 0 | 0 | 0 | 0 | 0 | 0 | 0 | 0 | 0 | 0 | 0 | 0 | 0 | 0 | 0 | 0 | 0 | 0 | 0 | 0 | 0 |
| Wang 2011 [202] | 1 | 0 | 0 | 0 | 0 | 0 | 0 | 0 | 0 | 0 | 0 | 0 | 0 | 0 | 0 | 0 | 0 | 0 | 0 | 0 | 0 | 0 | 0 | 0 | 0 | 0 | 0 | 0 | 0 | 0 | 0 | 0 | 0 | 0 |
| Wedell-Neergaard 2019 [204] | 0 | 0 | 0 | 0 | 0 | 0 | 0 | 0 | 0 | 0 | 0 | 1 | 0 | 0 | 0 | 0 | 0 | 0 | 0 | 0 | 0 | 0 | 0 | 0 | 0 | 0 | 0 | 0 | 0 | 0 | 0 | 0 | 0 | 0 |
| Winters-Stone 2018 [206] | 0 | 0 | 0 | 0 | 0 | 1 | 0 | 0 | 0 | 0 | 0 | 0 | 0 | 0 | 0 | 0 | 0 | 0 | 0 | 0 | 0 | 0 | 0 | 0 | 0 | 0 | 0 | 0 | 0 | 0 | 0 | 0 | 0 | 0 |
| Yoshimura 2014 [348] | 0 | 0 | 0 | 0 | 0 | 0 | 0 | 0 | 0 | 0 | 0 | 0 | 0 | 0 | 0 | 0 | 0 | 0 | 0 | 0 | 0 | 0 | 0 | 0 | 0 | 0 | 0 | 0 | 0 | 1 | 0 | 0 | 0 | 0 |
| You 2004 [213] | 0 | 0 | 0 | 0 | 0 | 0 | 0 | 0 | 0 | 0 | 0 | 1 | 0 | 0 | 2 | 0 | 0 | 0 | 0 | 0 | 0 | 0 | 0 | 0 | 0 | 0 | 0 | 0 | 0 | 3 | 0 | 0 | 0 | 0 |

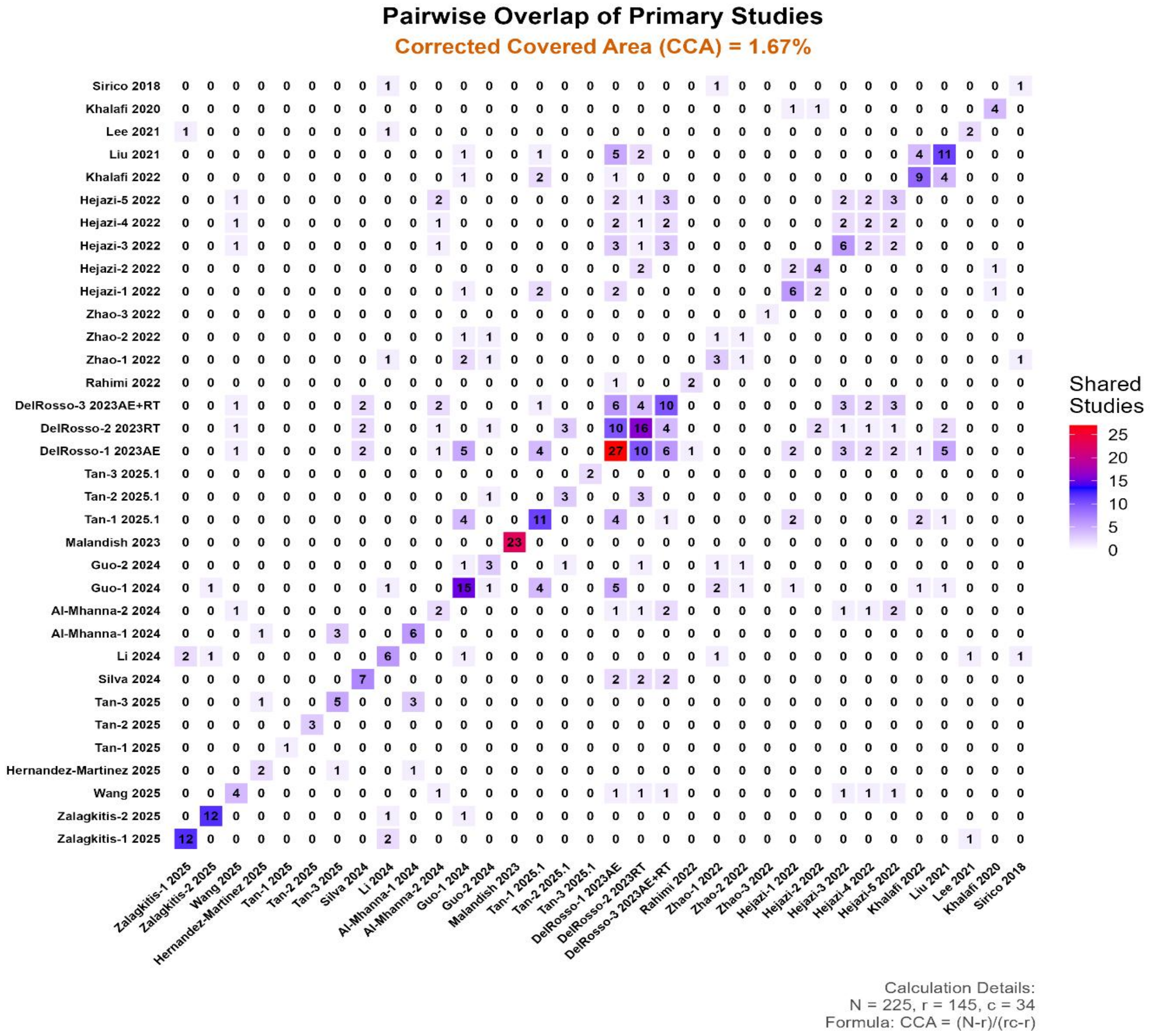


**Supplementary Figure S58. Graphical Overview of the Corrected Covered Area for IL-6**

## 7.7 Section 1: For IL-8

Supplementary Table S15. Corrected Covered Area (CCA) Overlap Analysis for IL-8

| Primary study | Zalagkitis 2025 | Al-Mhanna 2024 | Malandish 2023 | Del Rosso 2023 |
|---|---|---|---|---|
| Abd El-Kader 2015[284] | 0 | 0 | 0 | 1 |
| Balen 2008[33] | 0 | 0 | 1 | 0 |
| Croymans 2014[55] | 0 | 0 | 0 | 1 |
| Dieli-Conwright 2018[230] | 0 | 1 | 0 | 0 |
| Ergun 2013[297] | 0 | 1 | 0 | 0 |
| Hasson 2012[349] | 1 | 0 | 0 | 0 |
| Izadpanah 2012[341] | 1 | 0 | 0 | 0 |
| Munk 2011[309] | 0 | 0 | 1 | 0 |
| Nono Nankam 2020[135] | 0 | 0 | 0 | 1 |
| Racca 2020[313] | 0 | 0 | 1 | 0 |
| Rokling-Andersen 2007[165] | 0 | 0 | 0 | 1 |
| Rogers 2014[279] | 0 | 1 | 0 | 0 |
| Romeo 2011[328] | 1 | 0 | 0 | 0 |
| Roth 2011[329] | 1 | 0 | 0 | 0 |
| Vella 2017[195] | 0 | 0 | 0 | 1 |

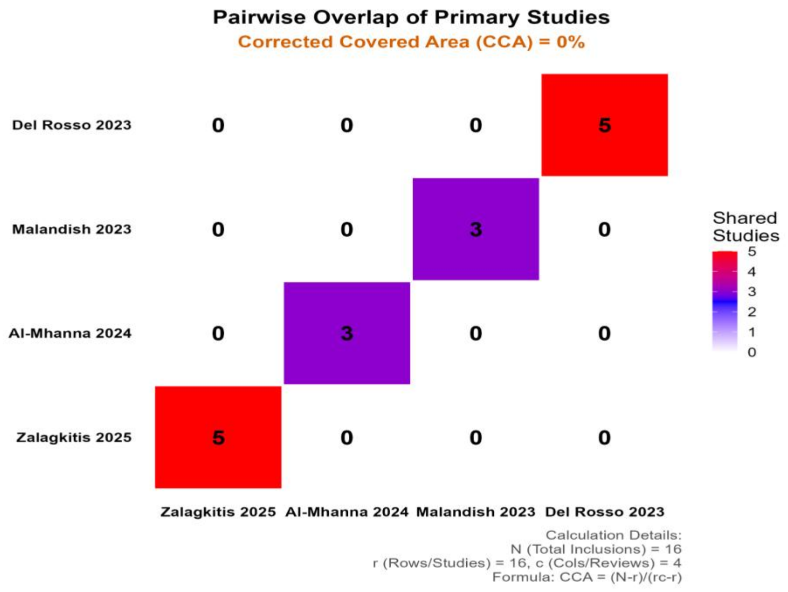


**Supplementary Figure S62. Graphical Overview of the Corrected Covered Area (CCA) for IL-8**

## 7.8 Section 1: For IL-10

Supplementary Table S16. Corrected Covered Area (CCA) Overlap Analysis for IL-10

| Primary study | Del Rosso 2023 | Tan 2025 |
|---|---|---|
| Kadoglou 2007[83] | 1 | 0 |
| Balducci 2010[31] | 1 | 0 |
| Nikseresht 2014[254] | 1 | 0 |
| Conroy 2016[350] | 1 | 0 |
| Shahram 2016[317] | 1 | 0 |
| Chagas 2017[291] | 1 | 0 |
| Nikseresht 2018[346] | 1 | 0 |
| Eizadi 2018[351] | 1 | 0 |
| Alizadeh 2019[352] | 0 | 1 |
| Hagstrom 2016[75] | 0 | 1 |
| Martins 2023[307] | 0 | 1 |
| Rogers 2013[278] | 0 | 1 |
| Rogers 2014[279] | 0 | 1 |

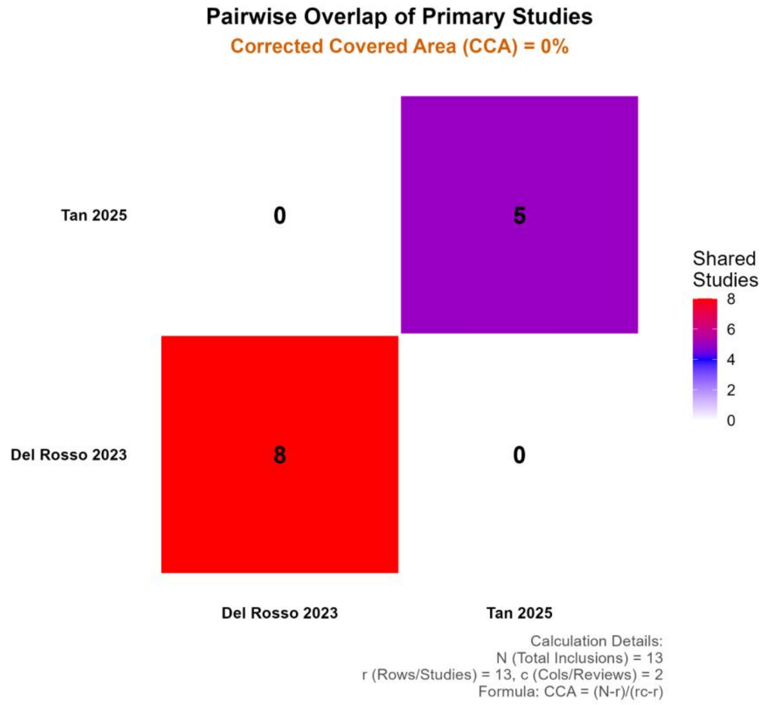


**Supplementary Figure S64. Graphical Overview of the Corrected Covered Area (CCA) for IL-10**

### 7.9 Section 1: For IL-18

Supplementary Table S17. Corrected Covered Area (CCA) Overlap Analysis for IL-18

| Primary study | Del Rosso 2023 | Ding 2022 |
|---|---|---|
| Kadoglou 2007[83] | 1 | 0 |
| Christiansen 2010[50] | 1 | 2 |
| Nikseresht 2016[353] | 1 | 2 |
| Barrón-Cabrera 2020[324] | 0 | 1 |
| Chen 2015[47] | 0 | 1 |
| Zakavi 2020[332] | 0 | 1 |

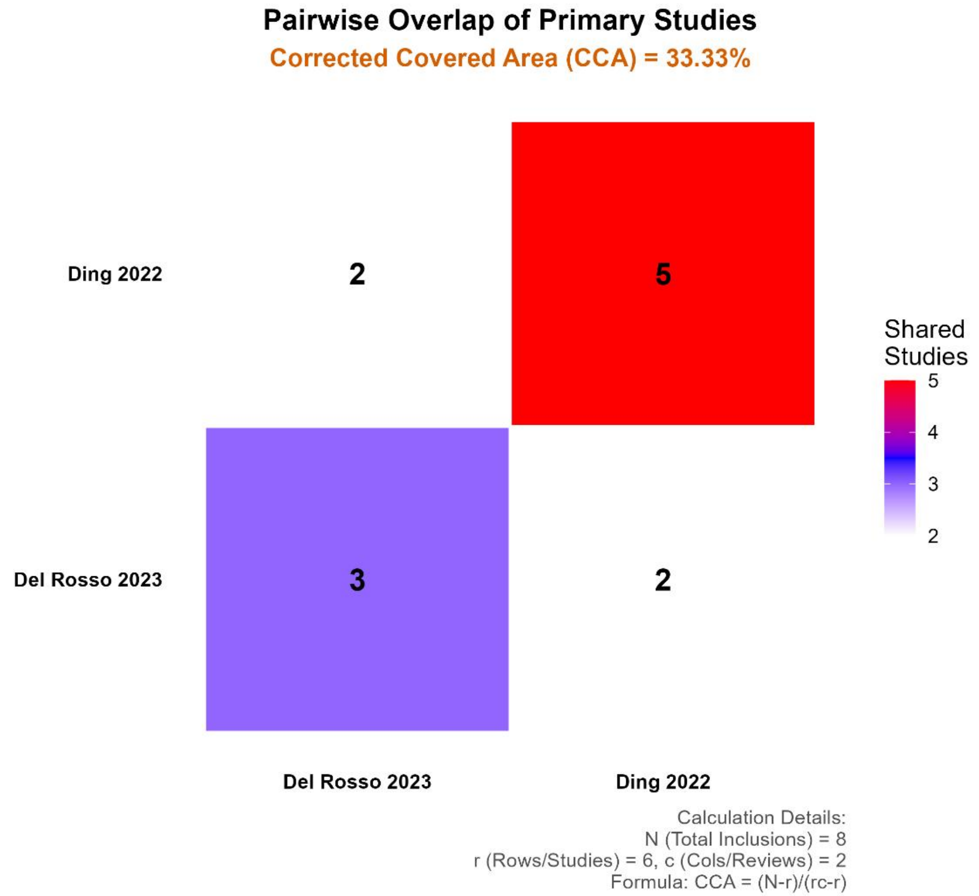


**Supplementary Figure S66. Graphical Overview of the Corrected Covered Area (CCA) for IL-18**

## References


1. Abassi W, Ouerghi N, Nikolaidis PT, Hill L, Racil G, Knechtle B, et al. Interval Training with Different Intensities in Overweight/Obese Adolescent Females. Int J Sports Med. 2022 May;43(5):434-43.https://doi.org/10.1055/a-1648-4653
2. Abd El-Kader SM, Saiem Al-Dahr MH. Weight loss improves biomarkers endothelial function and systemic inflammation in obese postmenopausal Saudi women. Afr Health Sci. 2016 Jun;16(2):533-41.https://doi.org/10.4314/ahs.v16i2.22
3. Abolahrari-Shirazi S, Kojuri J, Bagheri Z, Rojhani-Shirazi Z. Efficacy of combined endurance-resistance training versus endurance training in patients with heart failure after percutaneous coronary intervention: A randomized controlled trial. J Res Med Sci. 2018;23:12.https://doi.org/10.4103/jrms.JRMS_743_17
4. Abu-Kishk I, Alumot-Yehoshua M, Reisler G, Efrati S, Kozer E, Doenyas-Barak K, et al. Lifestyle modifications in an adolescent dormitory: a clinical trial. Korean J Pediatr. 2014 Dec;57(12):520-5.https://doi.org/10.3345/kjp.2014.57.12.520
5. Adamopoulos S, Schmid JP, Dendale P, Poerschke D, Hansen D, Dritsas A, et al. Combined aerobic/inspiratory muscle training vs. aerobic training in patients with chronic heart failure: The Vent-HeFT trial: a European prospective multicentre randomized trial. Eur J Heart Fail. 2014 May;16(5):574-82.https://doi.org/10.1002/ejhf.70
6. Ahmadizad S, Ghorbani S, Ghasemikaram M, Bahmanzadeh M. Effects of short-term nonperiodized, linear periodized and daily undulating periodized resistance training on plasma adiponectin, leptin and insulin resistance. Clin Biochem. 2014 Apr;47(6):417-22.https://doi.org/10.1016/j.clinbiochem.2013.12.019
7. Akbarpour M. The effect of aerobic training on serum adiponectin and leptin levels and inflammatory markers of coronary heart disease in obese men. Biol Sport. 2013 Mar;30(1):21-7.https://doi.org/10.5604/20831862.1029817
8. Aksoy S, Findikoglu G, Ardic F, Rota S, Dursunoglu D. Effect of 10-Week Supervised Moderate-Intensity Intermittent vs. Continuous Aerobic Exercise Programs on Vascular Adhesion Molecules in Patients with Heart Failure. Am J Phys Med Rehabil. 2015 2015/10//;94(10 Suppl 1):898-911.https://doi.org/10.1097/phm.0000000000000306
9. Alberga AS, Prud'homme D, Kenny GP, Goldfield GS, Hadjiyannakis S, Gougeon R, et al. Effects of aerobic and resistance training on abdominal fat, apolipoproteins and high-sensitivity C-reactive protein in adolescents with obesity: the HEARTY randomized clinical trial. Int J Obes (Lond). 2015 Oct;39(10):1494-500.https://doi.org/10.1038/ijo.2015.133
10. Allen NG, Higham SM, Mendham AE, Kastelein TE, Larsen PS, Duffield R. The effect of high-intensity aerobic interval training on markers of systemic inflammation in sedentary populations. Eur J Appl Physiol. 2017 Jun;117(6):1249-56.https://doi.org/10.1007/s00421-017-3613-1
11. Almenning I, Rieber-Mohn A, Lundgren KM, Shetelig Løvvik T, Garnæs KK, Moholdt T. Effects of High Intensity Interval Training and Strength Training on Metabolic, Cardiovascular and Hormonal Outcomes in Women with Polycystic Ovary Syndrome: A Pilot Study. PLoS One. 2015;10(9):e0138793.https://doi.org/10.1371/journal.pone.0138793
12. Amaro-Gahete FJ, Ponce-González JG, Corral-Pérez J, Velázquez-Díaz D, Lavie CJ, Jiménez-Pavón D. Effect of a 12-Week Concurrent Training Intervention on Cardiometabolic Health in Obese Men: A Pilot Study. Front Physiol. 2021;12:630831.https://doi.org/10.3389/fphys.2021.630831
13. Annibalini G, Lucertini F, Agostini D, Vallorani L, Gioacchini A, Barbieri E, et al. Concurrent Aerobic and Resistance Training Has Anti-Inflammatory Effects and Increases Both Plasma and Leukocyte Levels of IGF-1 in Late Middle-Aged Type 2 Diabetic Patients. Oxid Med Cell Longev. 2017;2017:3937842.https://doi.org/10.1155/2017/3937842
14. Arikawa AY, Thomas W, Schmitz KH, Kurzer MS. Sixteen weeks of exercise reduces C-reactive protein levels in young women. Med Sci Sports Exerc. 2011 Jun;43(6):1002-9.https://doi.org/10.1249/MSS.0b013e3182059eda
15. Arsenault BJ, Côté M, Cartier A, Lemieux I, Després JP, Ross R, et al. Effect of exercise training on cardiometabolic risk markers among sedentary, but metabolically healthy overweight or obese post-menopausal women with elevated blood pressure. Atherosclerosis. 2009 Dec;207(2):530-3.https://doi.org/10.1016/j.atherosclerosis.2009.05.009
16. Auerbach P, Nordby P, Bendtsen LQ, Mehlsen JL, Basnet SK, Vestergaard H, et al. Differential effects of endurance training and weight loss on plasma adiponectin multimers and adipose tissue macrophages in younger, moderately overweight men. Am J Physiol Regul Integr Comp Physiol. 2013 Sep;305(5):R490-8.https://doi.org/10.1152/ajpregu.00575.2012

17. Bagheri R, Rashidlamir A, Ashtary-Larky D, Wong A, Grubbs B, Motevalli MS, et al. Effects of green tea extract supplementation and endurance training on irisin, pro-inflammatory cytokines, and adiponectin concentrations in overweight middle-aged men. Eur J Appl Physiol. 2020 Apr;120(4):915-23.https://doi.org/10.1007/s00421-020-04332-6
18. Mallard AR, Hollekim-Strand SM, Coombes JS, Ingul CB. Exercise intensity, redox homeostasis and inflammation in type 2 diabetes mellitus. J Sci Med Sport. 2017 Oct;20(10):893-8.https://doi.org/10.1016/j.jsams.2017.03.014
19. Balagopal P, George D, Patton N, Yarandi H, Roberts WL, Bayne E, et al. Lifestyle-only intervention attenuates the inflammatory state associated with obesity: a randomized controlled study in adolescents. J Pediatr. 2005 Mar;146(3):342-8.https://doi.org/10.1016/j.jpeds.2004.11.033
20. Balducci S, Zanuso S, Nicolucci A, Fernando F, Cavallo S, Cardelli P, et al. Anti-inflammatory effect of exercise training in subjects with type 2 diabetes and the metabolic syndrome is dependent on exercise modalities and independent of weight loss. Nutr Metab Cardiovasc Dis. 2010 Oct;20(8):608-17.https://doi.org/10.1016/j.numecd.2009.04.015
21. Balducci S, D'Errico V, Haxhi J, Sacchetti M, Orlando G, Cardelli P, et al. Effect of a Behavioral Intervention Strategy for Adoption and Maintenance of a Physically Active Lifestyle: The Italian Diabetes and Exercise Study 2 (IDES_2): A Randomized Controlled Trial. Diabetes Care. 2017 Nov;40(11):1444-52.https://doi.org/10.2337/dc17-0594
22. Balen S, Vukelić-Damijani N, Persić V, Ruzić A, Miletić B, Samardiija M, et al. Anti-inflammatory effects of exercise training in the early period after myocardial infarction. Coll Antropol. 2008 Mar;32(1):285-91
23. Ben Ounis O, Elloumi M, Zouhal H, Makni E, Denguezli M, Amri M, et al. Effect of individualized exercise training combined with diet restriction on inflammatory markers and IGF-1/IGFBP-3 in obese children. Ann Nutr Metab. 2010;56(4):260-6.https://doi.org/10.1159/000275888
24. Bhati P, Hussain ME, Deepak KK, Masood S, Anand P. Progressive resistance training ameliorates deteriorating cardiac autonomic dysfunction, subclinical inflammation and endothelial dysfunction in type 2 diabetes mellitus: A randomized control trial. Diabetes Metab Syndr. 2023 May;17(5):102778.https://doi.org/10.1016/j.dsx.2023.102778
25. Bo S, Ciccone G, Baldi C, Benini L, Dusio F, Forastiere G, et al. Effectiveness of a lifestyle intervention on metabolic syndrome. A randomized controlled trial. J Gen Intern Med. 2007 Dec;22(12):1695-703.https://doi.org/10.1007/s11606-007-0399-6
26. Bocca G, Corpeleijn E, Stolk RP, Wolffenbuttel BH, Sauer PJ. Effect of obesity intervention programs on adipokines, insulin resistance, lipid profile, and low-grade inflammation in 3- to 5-y-old children. Pediatr Res. 2014 Feb;75(2):352-7.https://doi.org/10.1038/pr.2013.216
27. Bouchonville M, Armamento-Villareal R, Shah K, Napoli N, Sinacore DR, Qualls C, et al. Weight loss, exercise or both and cardiometabolic risk factors in obese older adults: results of a randomized controlled trial. Int J Obes (Lond). 2014 Mar;38(3):423-31.https://doi.org/10.1038/ijo.2013.122
28. Albuquerque Filho N, Bellaguarda E, Rebouças G, Felipe TR, Dantas P, Knackfuss MI, et al. Concurrent exercise program plus diet intervention on body adiposity and lipid profile in obese adolescents. Gazzetta medica italiana. 2015 06/01;174:259-66
29. Brochu M, Malita MF, Messier V, Doucet E, Strychar I, Lavoie JM, et al. Resistance training does not contribute to improving the metabolic profile after a 6-month weight loss program in overweight and obese postmenopausal women. J Clin Endocrinol Metab. 2009 Sep;94(9):3226-33.https://doi.org/10.1210/jc.2008-2706
30. Brooks N, Layne JE, Gordon PL, Roubenoff R, Nelson ME, Castaneda-Sceppa C. Strength training improves muscle quality and insulin sensitivity in Hispanic older adults with type 2 diabetes. Int J Med Sci. 2006 Dec 18;4(1):19-27.https://doi.org/10.7150/ijms.4.19
31. Brunelli DT, Chacon-Mikahil MP, Gáspari AF, Lopes WA, Bonganha V, Bonfante IL, et al. Combined Training Reduces Subclinical Inflammation in Obese Middle-Age Men. Med Sci Sports Exerc. 2015 Oct;47(10):2207-15.https://doi.org/10.1249/mss.0000000000000658
32. Byrkjeland R, Nilsson BB, Westheim AS, Arnesen H, Seljeflot I. Inflammatory markers as related to disease severity in patients with chronic heart failure: limited effects of exercise training. Scand J Clin Lab Invest. 2011 2011/11//;71(7):598-605.https://doi.org/10.3109/00365513.2011.598943

33. Camhi SM, Stefanick ML, Ridker PM, Young DR. Changes in C-reactive protein from low-fat diet and/or physical activity in men and women with and without metabolic syndrome. Metabolism. 2010;59(1):54-61
34. Campbell KL, Campbell PT, Ulrich CM, Wener M, Alfano CM, Foster-Schubert K, et al. No reduction in C-reactive protein following a 12-month randomized controlled trial of exercise in men and women. Cancer Epidemiol Biomarkers Prev. 2008 Jul;17(7):1714-8.https://doi.org/10.1158/1055-9965.Epi-08-0088
35. Canuto K, Cargo M, Li M, D'Onise K, Esterman A, McDermott R. Pragmatic randomised trial of a 12-week exercise and nutrition program for Aboriginal and Torres Strait Islander women: clinical results immediate post and 3 months follow-up. BMC Public Health. 2012 Oct 31;12:933.https://doi.org/10.1186/1471-2458-12-933
36. Chen Q, Cao J, Zhao L, Yi N. Effects of different exercise modes on body composition, inflammatory factors, and exercise capacity of obese teenagers. 2015 09/28;41:1070-5.https://doi.org/10.13481/j.1671-587x.20150537
37. Cho AR, Moon JY, Kim S, An KY, Oh M, Jeon JY, et al. Effects of alternate day fasting and exercise on cholesterol metabolism in overweight or obese adults: A pilot randomized controlled trial. Metabolism. 2019 Apr;93:52-60.https://doi.org/10.1016/j.metabol.2019.01.002
38. Choi KM, Han KA, Ahn HJ, Hwang SY, Hong HC, Choi HY, et al. Effects of exercise on sRAGE levels and cardiometabolic risk factors in patients with type 2 diabetes: a randomized controlled trial. J Clin Endocrinol Metab. 2012 Oct;97(10):3751-8.https://doi.org/10.1210/jc.2012-1951
39. Christiansen T, Paulsen SK, Bruun JM, Pedersen SB, Richelsen B. Exercise training versus diet-induced weight-loss on metabolic risk factors and inflammatory markers in obese subjects: a 12-week randomized intervention study. Am J Physiol Endocrinol Metab. 2010 Apr;298(4):E824-31.https://doi.org/10.1152/ajpendo.00574.2009
40. Church TS, Blair SN, Cocreham S, Johannsen N, Johnson W, Kramer K, et al. Effects of aerobic and resistance training on hemoglobin A1c levels in patients with type 2 diabetes: a randomized controlled trial. Jama. 2010 Nov 24;304(20):2253-62.https://doi.org/10.1001/jama.2010.1710
41. Cobos-Palacios L, Muñoz-Úbeda M, Gallardo-Escribano C, Ruiz-Moreno MI, Vilches-Pérez A, Vargas-Candela A, et al. Adipokines profile and inflammation biomarkers in prepubertal population with obesity and healthy metabolic state. Children (Basel). 2022;9(1)
42. Coll-Risco I, Borges-Cosic M, Acosta-Manzano P, Camiletti-Moirón D, Aranda P, Aparicio VA. Effects of concurrent exercise on cardiometabolic status during perimenopause: the FLAMENCO Project. Climacteric. 2018 Dec;21(6):559-65.https://doi.org/10.1080/13697137.2018.1526892
43. Cordellat A, Padilla B, Grattarola P, García-Lucerga C, Crehuá-Gaudiza E, Núñez F, et al. Multicomponent exercise training combined with nutritional counselling improves physical function, biochemical and anthropometric profiles in obese children: A pilot study. Nutrients. 2020;12(9)
44. Croymans DM, Krell SL, Oh CS, Katiraie M, Lam CY, Harris RA, et al. Effects of resistance training on central blood pressure in obese young men. J Hum Hypertens. 2014 Mar;28(3):157-64.https://doi.org/10.1038/jhh.2013.81
45. Cunha PM, Ribeiro AS, Nunes JP, Tomeleri CM, Nascimento MA, Moraes GK, et al. Resistance training performed with single-set is sufficient to reduce cardiovascular risk factors in untrained older women: The randomized clinical trial. Active Aging Longitudinal Study. Arch Gerontol Geriatr. 2019 Mar-Apr;81:171-5.https://doi.org/10.1016/j.archger.2018.12.012
46. da Silva PL, de Mello MT, Cheik NC, Sanches PL, Correia FA, de Piano A, et al. Interdisciplinary therapy improves biomarkers profile and lung function in asthmatic obese adolescents. Pediatr Pulmonol. 2012 Jan;47(1):8-17.https://doi.org/10.1002/ppul.21502
47. de Meirelles LR, Matsuura C, Resende Ade C, Salgado AA, Pereira NR, Coscarelli PG, et al. Chronic exercise leads to antiaggregant, antioxidant and anti-inflammatory effects in heart failure patients. Eur J Prev Cardiol. 2014 Oct;21(10):1225-32.https://doi.org/10.1177/2047487313491662
48. Dieli-Conwright CM, Courneya KS, Demark-Wahnefried W, Sami N, Lee K, Buchanan TA, et al. Effects of Aerobic and Resistance Exercise on Metabolic Syndrome, Sarcopenic Obesity, and Circulating Biomarkers in Overweight or Obese Survivors of Breast Cancer: A Randomized Controlled Trial. J Clin Oncol. 2018 Mar 20;36(9):875-83.https://doi.org/10.1200/jco.2017.75.7526

49. Donges CE, Duffield R, Guelfi KJ, Smith GC, Adams DR, Edge JA. Comparative effects of single-mode vs. duration-matched concurrent exercise training on body composition, low-grade inflammation, and glucose regulation in sedentary, overweight, middle-aged men. Appl Physiol Nutr Metab. 2013 Jul;38(7):779-88.https://doi.org/10.1139/apnm-2012-0443
50. Eleuteri E, Mezzani A, Di Stefano A, Vallese D, Gnemmi I, Delle Donne L, et al. Aerobic training and angiogenesis activation in patients with stable chronic heart failure: a preliminary report. Biomarkers. 2013 Aug;18(5):418-24.https://doi.org/10.3109/1354750x.2013.805342
51. Fairey AS, Courneya KS, Field CJ, Bell GJ, Jones LW, Martin BS, et al. Effect of exercise training on C-reactive protein in postmenopausal breast cancer survivors: a randomized controlled trial. Brain Behav Immun. 2005 Sep;19(5):381-8.https://doi.org/10.1016/j.bbi.2005.04.001
52. Farpour-Lambert NJ, Aggoun Y, Marchand LM, Martin XE, Herrmann FR, Beghetti M. Physical activity reduces systemic blood pressure and improves early markers of atherosclerosis in pre-pubertal obese children. J Am Coll Cardiol. 2009 Dec 15;54(25):2396-406.https://doi.org/10.1016/j.jacc.2009.08.030
53. Fedewa MV, Hathaway ED, Higgins S, Forehand RL, Schmidt MD, Evans EM. Moderate, but not vigorous, intensity exercise training reduces C-reactive protein. Acta Cardiol. 2018 Jun;73(3):283-90.https://doi.org/10.1080/00015385.2017.1364832
54. Fisher G, Hyatt TC, Hunter GR, Oster RA, Desmond RA, Gower BA. Effect of diet with and without exercise training on markers of inflammation and fat distribution in overweight women. Obesity (Silver Spring). 2011 Jun;19(6):1131-6.https://doi.org/10.1038/oby.2010.310
55. Flandez J, Belando N, Gargallo P, Fernández-Garrido J, Vargas-Foitzick RA, Devis-Devis J, et al. Metabolic and Functional Profile of Premenopausal Women With Metabolic Syndrome After Training With Elastics as Compared to Free Weights. Biol Res Nurs. 2017 Mar;19(2):190-7.https://doi.org/10.1177/1099800416674307
56. Franklin NC, Robinson AT, Bian JT, Ali MM, Norkeviciute E, McGinty P, et al. Circuit resistance training attenuates acute exertion-induced reductions in arterial function but not inflammation in obese women. Metab Syndr Relat Disord. 2015 Jun;13(5):227-34.https://doi.org/10.1089/met.2014.0135
57. Friedenreich CM, Neilson HK, Woolcott CG, Wang Q, Stanczyk FZ, McTiernan A, et al. Inflammatory marker changes in a yearlong randomized exercise intervention trial among postmenopausal women. Cancer Prev Res (Phila). 2012 Jan;5(1):98-108.https://doi.org/10.1158/1940-6207.Capr-11-0369
58. Garanty-Bogacka B, Syrenicz M, Goral J, Krupa B, Syrenicz J, Walczak M, et al. Changes in inflammatory biomarkers after successful lifestyle intervention in obese children. Endokrynol Pol. 2011;62(6):499-505
59. Giallauria F, Cirillo P, D'Agostino M, Petrillo G, Vitelli A, Pacileo M, et al. Effects of exercise training on high-mobility group box-1 levels after acute myocardial infarction. J Card Fail. 2011 Feb;17(2):108-14.https://doi.org/10.1016/j.cardfail.2010.09.001
60. Giannopoulou I, Fernhall B, Carhart R, Weinstock RS, Baynard T, Figueroa A, et al. Effects of diet and/or exercise on the adipocytokine and inflammatory cytokine levels of postmenopausal women with type 2 diabetes. Metabolism. 2005 Jul;54(7):866-75.https://doi.org/10.1016/j.metabol.2005.01.033
61. Gómez-Tomás C, Chulvi-Medrano I, Carrasco JJ, Alakhdar Y. Effect of a 1-year elastic band resistance exercise program on cardiovascular risk profile in postmenopausal women. Menopause. 2018 Sep;25(9):1004-10.https://doi.org/10.1097/gme.0000000000001113
62. Gong L, Yuan F, Teng J, Li X, Zheng S, Lin L, et al. Weight loss, inflammatory markers, and improvements of iron status in overweight and obese children. J Pediatr. 2014 Apr;164(4):795-800.e2.https://doi.org/10.1016/j.jpeds.2013.12.004
63. Gokulakrishnan K, Ranjani H, Weber MB, Pandey GK, Anjana RM, Balasubramanyam M, et al. Effect of lifestyle improvement program on the biomarkers of adiposity, inflammation and gut hormones in overweight/obese Asian Indians with prediabetes. Acta Diabetol. 2017 Sep;54(9):843-52.https://doi.org/10.1007/s00592-017-1015-9
64. Hagstrom AD, Marshall PW, Lonsdale C, Papalia S, Cheema BS, Toben C, et al. The effect of resistance training on markers of immune function and inflammation in previously sedentary women recovering from breast cancer: a randomized controlled trial. Breast Cancer Res Treat. 2016 Feb;155(3):471-82.https://doi.org/10.1007/s10549-016-3688-0

65. Henríquez S, Monsalves-Alvarez M, Jimenez T, Barrera G, Hirsch S, de la Maza MP, et al. Effects of Two Training Modalities on Body Fat and Insulin Resistance in Postmenopausal Women. J Strength Cond Res. 2017 Nov;31(11):2955-64.https://doi.org/10.1519/jsc.0000000000002089
66. Herder C, Peltonen M, Koenig W, Sütfels K, Lindström J, Martin S, et al. Anti-inflammatory effect of lifestyle changes in the Finnish Diabetes Prevention Study. Diabetologia. 2009 Mar;52(3):433-42.https://doi.org/10.1007/s00125-008-1243-1
67. Imayama I, Ulrich CM, Alfano CM, Wang C, Xiao L, Wener MH, et al. Effects of a caloric restriction weight loss diet and exercise on inflammatory biomarkers in overweight/obese postmenopausal women: a randomized controlled trial. Cancer Res. 2012 May 1;72(9):2314-26.https://doi.org/10.1158/0008-5472.Can-11-3092
68. Isaksen K, Halvorsen B, Munk PS, Aukrust P, Larsen AI. Effects of interval training on inflammatory biomarkers in patients with ischemic heart failure. Scand Cardiovasc J. 2019 Aug;53(4):213-9.https://doi.org/10.1080/14017431.2019.1629004
69. Johannsen NM, Swift DL, Johnson WD, Dixit VD, Earnest CP, Blair SN, et al. Effect of different doses of aerobic exercise on total white blood cell (WBC) and WBC subfraction number in postmenopausal women: results from DREW. PLoS One. 2012;7(2):e31319.https://doi.org/10.1371/journal.pone.0031319
70. Jones SB, Thomas GA, Hesselsweet SD, Alvarez-Reeves M, Yu H, Irwin ML. Effect of exercise on markers of inflammation in breast cancer survivors: the Yale exercise and survivorship study. Cancer Prev Res (Phila). 2013 Feb;6(2):109-18.https://doi.org/10.1158/1940-6207.Capr-12-0278
71. Jorge ML, de Oliveira VN, Resende NM, Paraiso LF, Calixto A, Diniz AL, et al. The effects of aerobic, resistance, and combined exercise on metabolic control, inflammatory markers, adipocytokines, and muscle insulin signaling in patients with type 2 diabetes mellitus. Metabolism. 2011 Sep;60(9):1244-52.https://doi.org/10.1016/j.metabol.2011.01.006
72. Kadoglou NP, Iliadis F, Angelopoulou N, Perrea D, Ampatzidis G, Liapis CD, et al. The anti-inflammatory effects of exercise training in patients with type 2 diabetes mellitus. Eur J Cardiovasc Prev Rehabil. 2007 Dec;14(6):837-43.https://doi.org/10.1097/HJR.0b013e3282efaf50
73. Kadoglou NP, Fotiadis G, Athanasiadou Z, Vitta I, Lampropoulos S, Vrabas IS. The effects of resistance training on ApoB/ApoA-I ratio, Lp(a) and inflammatory markers in patients with type 2 diabetes. Endocrine. 2012 Dec;42(3):561-9.https://doi.org/10.1007/s12020-012-9650-y
74. Kadoglou NP, Fotiadis G, Kapelouzou A, Kostakis A, Liapis CD, Vrabas IS. The differential anti-inflammatory effects of exercise modalities and their association with early carotid atherosclerosis progression in patients with type 2 diabetes. Diabet Med. 2013 Feb;30(2):e41-50.https://doi.org/10.1111/dme.12055
75. Keating SE, Hackett DA, Parker HM, O'Connor HT, Gerofi JA, Sainsbury A, et al. Effect of aerobic exercise training dose on liver fat and visceral adiposity. J Hepatol. 2015 Jul;63(1):174-82.https://doi.org/10.1016/j.jhep.2015.02.022
76. Kelishadi R, Hashemi M, Mohammadifard N, Asgary S, Khavarian N. Association of changes in oxidative and proinflammatory states with changes in vascular function after a lifestyle modification trial among obese children. Clin Chem. 2008 Jan;54(1):147-53.https://doi.org/10.1373/clinchem.2007.089953
77. Kelly AS, Wetzsteon RJ, Kaiser DR, Steinberger J, Bank AJ, Dengel DR. Inflammation, insulin, and endothelial function in overweight children and adolescents: the role of exercise. J Pediatr. 2004 Dec;145(6):731-6.https://doi.org/10.1016/j.jpeds.2004.08.004
78. Kelly AS, Steinberger J, Olson TP, Dengel DR. In the absence of weight loss, exercise training does not improve adipokines or oxidative stress in overweight children. Metabolism. 2007 Jul;56(7):1005-9.https://doi.org/10.1016/j.metabol.2007.03.009
79. Khoo J, Dhamodaran S, Chen DD, Yap SY, Chen RY, Tian RH. Exercise-Induced Weight Loss is More Effective than Dieting for Improving Adipokine Profile, Insulin Resistance, and Inflammation in Obese Men. Int J Sport Nutr Exerc Metab. 2015 Dec;25(6):566-75.https://doi.org/10.1123/ijsnem.2015-0025
80. Kim ES, Im JA, Kim KC, Park JH, Suh SH, Kang ES, et al. Improved insulin sensitivity and adiponectin level after exercise training in obese Korean youth. Obesity (Silver Spring). 2007 Dec;15(12):3023-30.https://doi.org/10.1038/oby.2007.360

81. Kim YJ, Shin YO, Bae JS, Lee JB, Ham JH, Son YJ, et al. Beneficial effects of cardiac rehabilitation and exercise after percutaneous coronary intervention on hsCRP and inflammatory cytokines in CAD patients. Pflugers Arch. 2008 Mar;455(6):1081-8.https://doi.org/10.1007/s00424-007-0356-6
82. Kim C, Kim DY, Moon CJ. Prognostic influences of cardiac rehabilitation in korean acute myocardial infarction patients. Ann Rehabil Med. 2011 Jun;35(3):375-80.https://doi.org/10.5535/arm.2011.35.3.375
83. Kim H, Kim M, Kojima N, Fujino K, Hosoi E, Kobayashi H, et al. Exercise and Nutritional Supplementation on Community-Dwelling Elderly Japanese Women With Sarcopenic Obesity: A Randomized Controlled Trial. J Am Med Dir Assoc. 2016 Nov 1;17(11):1011-9.https://doi.org/10.1016/j.jamda.2016.06.016
84. Koh Y, Park KS. Responses of inflammatory cytokines following moderate intensity walking exercise in overweight or obese individuals. J Exerc Rehabil. 2017 Aug;13(4):472-6.https://doi.org/10.12965/jer.1735066.533
85. Kolahdouzi S, Baghadam M, Kani-Golzar FA, Saeidi A, Jabbour G, Ayadi A, et al. Progressive circuit resistance training improves inflammatory biomarkers and insulin resistance in obese men. Physiol Behav. 2019 Jun 1;205:15-21.https://doi.org/10.1016/j.physbeh.2018.11.033
86. Kondo T, Kobayashi I, Murakami M. Effect of exercise on circulating adipokine levels in obese young women. Endocr J. 2006 Apr;53(2):189-95.https://doi.org/10.1507/endocrj.53.189
87. Lam YY, Ghosh S, Civitarese AE, Ravussin E. Six-month Calorie Restriction in Overweight Individuals Elicits Transcriptomic Response in Subcutaneous Adipose Tissue That is Distinct From Effects of Energy Deficit. J Gerontol A Biol Sci Med Sci. 2016 Oct;71(10):1258-65.https://doi.org/10.1093/gerona/glv194
88. Lambert CP, Wright NR, Finck BN, Villareal DT. Exercise but not diet-induced weight loss decreases skeletal muscle inflammatory gene expression in frail obese elderly persons. J Appl Physiol (1985). 2008 Aug;105(2):473-8.https://doi.org/10.1152/japplphysiol.00006.2008
89. Lee YH, Song YW, Kim HS, Lee SY, Jeong HS, Suh SH, et al. The effects of an exercise program on anthropometric, metabolic, and cardiovascular parameters in obese children. Korean Circ J. 2010 Apr;40(4):179-84.https://doi.org/10.4070/kcj.2010.40.4.179
90. Lee MG, Park KS, Kim DU, Choi SM, Kim HJ. Effects of high-intensity exercise training on body composition, abdominal fat loss, and cardiorespiratory fitness in middle-aged Korean females. Appl Physiol Nutr Metab. 2012 Dec;37(6):1019-27.https://doi.org/10.1139/h2012-084
91. Libardi CA, De Souza GV, Cavaglieri CR, Madruga VA, Chacon-Mikahil MP. Effect of resistance, endurance, and concurrent training on TNF-α, IL-6, and CRP. Med Sci Sports Exerc. 2012 Jan;44(1):50-6.https://doi.org/10.1249/MSS.0b013e318229d2e9
92. Ligibel JA, Dillon D, Giobbie-Hurder A, McTiernan A, Frank E, Cornwell M, et al. Impact of a Pre-Operative Exercise Intervention on Breast Cancer Proliferation and Gene Expression: Results from the Pre-Operative Health and Body (PreHAB) Study. Clin Cancer Res. 2019 Sep 1;25(17):5398-406.https://doi.org/10.1158/1078-0432.Ccr-18-3143
93. Liu M, Gillis LJ, Persadie NR, Atkinson SA, Phillips SM, Timmons BW. Effects of Short-Term Exercise Training With and Without Milk Intake on Cardiometabolic and Inflammatory Adaptations in Obese Adolescents. Pediatr Exerc Sci. 2015 Nov;27(4):518-24.https://doi.org/10.1123/pes.2015-0053
94. Liu M, Lin X, Wang X. Decrease in serum chemerin through aerobic exercise plus dieting and its association with mitigation of cardio-metabolic risk in obese female adolescents. J Pediatr Endocrinol Metab. 2018 Jan 26;31(2):127-35.https://doi.org/10.1515/jpem-2017-0431
95. Lopes WA, Leite N, da Silva LR, Brunelli DT, Gáspari AF, Radominski RB, et al. Effects of 12 weeks of combined training without caloric restriction on inflammatory markers in overweight girls. J Sports Sci. 2016 Oct;34(20):1902-12.https://doi.org/10.1080/02640414.2016.1142107
96. Loria-Kohen V, Fernández-Fernández C, Bermejo LM, Morencos E, Romero-Moraleda B, Gómez-Candela C. Effect of different exercise modalities plus a hypocaloric diet on inflammation markers in overweight patients: a randomised trial. Clin Nutr. 2013 Aug;32(4):511-8.https://doi.org/10.1016/j.clnu.2012.10.015

97. Magalhães JP, Júdice PB, Ribeiro R, Andrade R, Raposo J, Dores H, et al. Effectiveness of high-intensity interval training combined with resistance training versus continuous moderate-intensity training combined with resistance training in patients with type 2 diabetes: A one-year randomized controlled trial. Diabetes Obes Metab. 2019 Mar;21(3):550-9.https://doi.org/10.1111/dom.13551
98. Magalhães JP, Santos DA, Correia IR, Hetherington-Rauth M, Ribeiro R, Raposo JF, et al. Impact of combined training with different exercise intensities on inflammatory and lipid markers in type 2 diabetes: a secondary analysis from a 1-year randomized controlled trial. Cardiovasc Diabetol. 2020 Oct 7;19(1):169.https://doi.org/10.1186/s12933-020-01136-y
99. Marco E, Ramírez-Sarmiento AL, Coloma A, Sartor M, Comin-Colet J, Vila J, et al. High-intensity vs. sham inspiratory muscle training in patients with chronic heart failure: a prospective randomized trial. Eur J Heart Fail. 2013 Aug;15(8):892-901.https://doi.org/10.1093/eurjhf/hft035
100. Marcell TJ, McAuley KA, Traustadóttir T, Reaven PD. Exercise training is not associated with improved levels of C-reactive protein or adiponectin. Metabolism. 2005 Apr;54(4):533-41.https://doi.org/10.1016/j.metabol.2004.11.008
101. Martins RA, Neves AP, Coelho-Silva MJ, Veríssimo MT, Teixeira AM. The effect of aerobic versus strength-based training on high-sensitivity C-reactive protein in older adults. Eur J Appl Physiol. 2010 Sep;110(1):161-9.https://doi.org/10.1007/s00421-010-1488-5
102. Martins FM, de Paula Souza A, Nunes PRP, Michelin MA, Murta EFC, Resende E, et al. High-intensity body weight training is comparable to combined training in changes in muscle mass, physical performance, inflammatory markers and metabolic health in postmenopausal women at high risk for type 2 diabetes mellitus: A randomized controlled clinical trial. Exp Gerontol. 2018 Jul 1;107:108-15.https://doi.org/10.1016/j.exger.2018.02.016
103. Masquio DCL, Campos R, Netto BDM, Carvalho-Ferreira JP, Bueno CR, Jr., Alouan S, et al. Interdisciplinary Therapy Improves the Mediators of Inflammation and Cardiovascular Risk in Adolescents with Obesity. Int J Environ Res Public Health. 2023 Nov 27;20(23).https://doi.org/10.3390/ijerph20237114
104. Masterson Creber RM, Lee CS, Margulies K, Riegel B. Identifying biomarker patterns and predictors of inflammation and myocardial stress. J Card Fail. 2015 Jun;21(6):439-45.https://doi.org/10.1016/j.cardfail.2015.02.006
105. Mavros Y, Kay S, Simpson KA, Baker MK, Wang Y, Zhao RR, et al. Reductions in C-reactive protein in older adults with type 2 diabetes are related to improvements in body composition following a randomized controlled trial of resistance training. J Cachexia Sarcopenia Muscle. 2014 Jun;5(2):111-20.https://doi.org/10.1007/s13539-014-0134-1
106. Mayerhofer E, Ratzinger F, Kienreich NE, Stiel A, Witzeneder N, Schrefl E, et al. A Multidisciplinary Intervention in Childhood Obesity Acutely Improves Insulin Resistance and Inflammatory Markers Independent From Body Composition. Front Pediatr. 2020;8:52.https://doi.org/10.3389/fped.2020.00052
107. McDermott MM, Tiukinhoy S, Greenland P, Liu K, Pearce WH, Guralnik JM, et al. A pilot exercise intervention to improve lower extremity functioning in peripheral arterial disease unaccompanied by intermittent claudication. J Cardiopulm Rehabil. 2004 May-Jun;24(3):187-96.https://doi.org/10.1097/00008483-200405000-00010
108. Mendham AE, Duffield R, Marino F, Coutts AJ. A 12-week sports-based exercise programme for inactive Indigenous Australian men improved clinical risk factors associated with type 2 diabetes mellitus. J Sci Med Sport. 2015 Jul;18(4):438-43.https://doi.org/10.1016/j.jsams.2014.06.013
109. Meyer AA, Kundt G, Lenschow U, Schuff-Werner P, Kienast W. Improvement of early vascular changes and cardiovascular risk factors in obese children after a six-month exercise program. J Am Coll Cardiol. 2006 Nov 7;48(9):1865-70.https://doi.org/10.1016/j.jacc.2006.07.035
110. Mietus-Snyder M, Narayanan N, Krauss RM, Laine-Graves K, McCann JC, Shigenaga MK, et al. Randomized nutrient bar supplementation improves exercise-associated changes in plasma metabolome in adolescents and adult family members at cardiometabolic risk. PLoS One. 2020;15(10):e0240437.https://doi.org/10.1371/journal.pone.0240437
111. Milani RV, Lavie CJ, Mehra MR. Reduction in C-reactive protein through cardiac rehabilitation and exercise training. J Am Coll Cardiol. 2004 Mar 17;43(6):1056-61.https://doi.org/10.1016/j.jacc.2003.10.041

112. Moghadasi M, Mohebbi H, Rahmani-Nia F, Hassan-Nia S, Noroozi H. Effects of short-term lifestyle activity modification on adiponectin mRNA expression and plasma concentrations. Eur J Sport Sci. 2013;13(4):378-85.https://doi.org/10.1080/17461391.2011.635701
113. Moghadasi M, Mohebbi H, Rahmani-Nia F, Hassan-Nia S, Noroozi H, Pirooznia N. High-intensity endurance training improves adiponectin mRNA and plasma concentrations. Eur J Appl Physiol. 2012 Apr;112(4):1207-14.https://doi.org/10.1007/s00421-011-2073-2
114. Montero D, Vicente-Salar N, Herranz M, Micol V, Walther G, Pérez-Martín A, et al. Glutathione-dependent enzyme activities of peripheral blood mononuclear cells decrease during the winter season compared with the summer in normal-weight and severely obese adolescents. J Physiol Biochem. 2019 Aug;75(3):321-7.https://doi.org/10.1007/s13105-019-00693-5
115. Mora-Rodriguez R, Ramirez-Jimenez M, Fernandez-Elias VE, Guio de Prada MV, Morales-Palomo F, Pallares JG, et al. Effects of aerobic interval training on arterial stiffness and microvascular function in patients with metabolic syndrome. J Clin Hypertens (Greenwich). 2018 Jan;20(1):11-8.https://doi.org/10.1111/jch.13130
116. Moraes GG, Reuter CP, Renner JD, Klinger EI, Ferreira MB, Mello ED, et al. Genotypic carriers of the obesity-associated FTO polymorphism exhibit different cardiometabolic profiles after an intervention. An Acad Bras Cienc. 2016 Oct-Dec;88(4):2331-9.https://doi.org/10.1590/0001-3765201620160114
117. Murphy EC, Carson L, Neal W, Baylis C, Donley D, Yeater R. Effects of an exercise intervention using Dance Dance Revolution on endothelial function and other risk factors in overweight children. Int J Pediatr Obes. 2009;4(4):205-14.https://doi.org/10.3109/17477160902846187
118. Myers JN, White JJ, Narasimhan B, Dalman RL. Effects of exercise training in patients with abdominal aortic aneurysm: preliminary results from a randomized trial. J Cardiopulm Rehabil Prev. 2010 Nov-Dec;30(6):374-83.https://doi.org/10.1097/HCR.0b013e3181ebf2db
119. Nadi M, Bambaeichi E, Marandi SM. Comparison of the effect of two therapeutic exercises on the inflammatory and physiological conditions and complications of diabetic neuropathy in female patients. Diabetes Metab Syndr Obes. 2019;12:1493-501.https://doi.org/10.2147/dmso.S206454
120. Nanri A, Tomita K, Matsushita Y, Ichikawa F, Yamamoto M, Nagafuchi Y, et al. Effect of six months lifestyle intervention in Japanese men with metabolic syndrome: randomized controlled trial. J Occup Health. 2012;54(3):215-22.https://doi.org/10.1539/joh.11-0238-oa
121. Nicklas BJ, Ambrosius W, Messier SP, Miller GD, Penninx BW, Loeser RF, et al. Diet-induced weight loss, exercise, and chronic inflammation in older, obese adults: a randomized controlled clinical trial. Am J Clin Nutr. 2004 Apr;79(4):544-51.https://doi.org/10.1093/ajcn/79.4.544
122. Nikseresht M, Sadeghifard N, Agha-Alinejad H, Ebrahim K. Inflammatory markers and adipocytokine responses to exercise training and detraining in men who are obese. J Strength Cond Res. 2014 Dec;28(12):3399-410.https://doi.org/10.1519/jsc.0000000000000553
123. Nişancı Kılınç F, Çağdaş DN. Diet and physical activity interventions do have effects on body composition and metabolic syndrome parameters in overweight and obese adolescents and their mothers. Turk J Pediatr. 2013 May-Jun;55(3):292-9
124. Nono Nankam PA, Mendham AE, De Smidt MF, Keswell D, Olsson T, Blüher M, et al. Changes in systemic and subcutaneous adipose tissue inflammation and oxidative stress in response to exercise training in obese black African women. J Physiol. 2020 Feb;598(3):503-15.https://doi.org/10.1113/jp278669
125. Nunes JE, Cunha HS, Freitas ZR, Nogueira AM, Dâmaso AR, Espindola FS, et al. Interdisciplinary therapy changes superoxide dismutase activity and adiponectin in obese adolescents: a randomised controlled trial. J Sports Sci. 2016;34(10):945-50.https://doi.org/10.1080/02640414.2015.1080384
126. Oh EG, Bang SY, Kim SH, Hyun SS, Chu SH, Jeon JY, et al. Therapeutic lifestyle modification program reduces plasma levels of the chemokines CRP and MCP-1 in subjects with metabolic syndrome. Biol Res Nurs. 2013 Jan;15(1):48-55.https://doi.org/10.1177/1099800411416637
127. Oh S, Tanaka K, Tsujimoto T, So R, Shida T, Shoda J. Regular exercise coupled to diet regimen accelerates reduction of hepatic steatosis and associated pathological conditions in nonalcoholic fatty liver disease. Metab Syndr Relat Disord. 2014 Jun;12(5):290-8.https://doi.org/10.1089/met.2013.0143

128. Okada S, Hiuge A, Makino H, Nagumo A, Takaki H, Konishi H, et al. Effect of exercise intervention on endothelial function and incidence of cardiovascular disease in patients with type 2 diabetes. J Atheroscler Thromb. 2010 Aug 31;17(8):828-33.https://doi.org/10.5551/jat.3798
129. Olson TP, Dengel DR, Leon AS, Schmitz KH. Changes in inflammatory biomarkers following one-year of moderate resistance training in overweight women. Int J Obes (Lond). 2007 Jun;31(6):996-1003.https://doi.org/10.1038/sj.ijo.0803534
130. Park TG, Hong HR, Lee J, Kang HS. Lifestyle plus exercise intervention improves metabolic syndrome markers without change in adiponectin in obese girls. Ann Nutr Metab. 2007;51(3):197-203.https://doi.org/10.1159/000104137
131. Park JH, Miyashita M, Kwon YC, Park HT, Kim EH, Park JK, et al. A 12-week after-school physical activity programme improves endothelial cell function in overweight and obese children: a randomised controlled study. BMC Pediatr. 2012 Jul 31;12:111.https://doi.org/10.1186/1471-2431-12-111
132. Park J, Kwon Y, Park H. Effects of 24-Week Aerobic and Resistance Training on Carotid Artery Intima-Media Thickness and Flow Velocity in Elderly Women with Sarcopenic Obesity. J Atheroscler Thromb. 2017 Nov 1;24(11):1117-24.https://doi.org/10.5551/jat.39065
133. Parrinello G, Torres D, Paterna S, Di Pasquale P, Trapanese C, Licata G. Short-term walking physical training and changes in body hydration status, B-type natriuretic peptide and C-reactive protein levels in compensated congestive heart failure. Int J Cardiol. 2010 Sep 24;144(1):97-100.https://doi.org/10.1016/j.ijcard.2008.12.130
134. Pedersen LR, Olsen RH, Anholm C, Walzem RL, Fenger M, Eugen-Olsen J, et al. Weight loss is superior to exercise in improving the atherogenic lipid profile in a sedentary, overweight population with stable coronary artery disease: A randomized trial. Atherosclerosis. 2016 Mar;246:221-8.https://doi.org/10.1016/j.atherosclerosis.2016.01.001
135. Pérez-López A, Gonzalo-Encabo P, Pérez-Köhler B, García-Honduvilla N, Valadés D. Circulating myokines IL-6, IL-15 and FGF21 response to training is altered by exercise type but not by menopause in women with obesity. Eur J Sport Sci. 2022 Sep;22(9):1426-35.https://doi.org/10.1080/17461391.2021.1939430
136. Phillips MD, Patrizi RM, Cheek DJ, Wooten JS, Barbee JJ, Mitchell JB. Resistance training reduces subclinical inflammation in obese, postmenopausal women. Med Sci Sports Exerc. 2012 Nov;44(11):2099-110.https://doi.org/10.1249/MSS.0b013e3182644984
137. Pierce GL, Schofield RS, Casey DP, Hamlin SA, Hill JA, Braith RW. Effects of exercise training on forearm and calf vasodilation and proinflammatory markers in recent heart transplant recipients: a pilot study. Eur J Cardiovasc Prev Rehabil. 2008 Feb;15(1):10-8.https://doi.org/10.1097/HJR.0b013e3282f0b63b
138. Plavsic L, Knezevic OM, Sovtic A, Minic P, Vukovic R, Mazibrada I, et al. Effects of high-intensity interval training and nutrition advice on cardiometabolic markers and aerobic fitness in adolescent girls with obesity. Appl Physiol Nutr Metab. 2020 Mar;45(3):294-300.https://doi.org/10.1139/apnm-2019-0137
139. Plotnikoff RC, Eves N, Jung M, Sigal RJ, Padwal R, Karunamuni N. Multicomponent, home-based resistance training for obese adults with type 2 diabetes: a randomized controlled trial. Int J Obes (Lond). 2010 Dec;34(12):1733-41.https://doi.org/10.1038/ijo.2010.109
140. Prescott E, Hjardem-Hansen R, Dela F, Ørkild B, Teisner AS, Nielsen H. Effects of a 14-month low-cost maintenance training program in patients with chronic systolic heart failure: a randomized study. Eur J Cardiovasc Prev Rehabil. 2009 Aug;16(4):430-7.https://doi.org/10.1097/HJR.0b013e32831e94f8
141. Pullen PR, Nagamia SH, Mehta PK, Thompson WR, Benardot D, Hammoud R, et al. Effects of yoga on inflammation and exercise capacity in patients with chronic heart failure. J Card Fail. 2008 Jun;14(5):407-13.https://doi.org/10.1016/j.cardfail.2007.12.007
142. Rahbar S, Naimi S, Soltani A, Rahimi A, Akbarzadeh A, Rashedi V, et al. Improvement in Biochemical Parameters in Patients with Type 2 Diabetes After Twenty-Four Sessions of Aerobic Exercise: A Randomized Controlled Trial. Iranian Red Crescent Medical Journal. 2017 05/23;19:e13931.https://doi.org/10.5812/ircmj.13931
143. Ranković G, Milicić B, Savić T, Dindić B, Mancev Z, Pesić G. Effects of physical exercise on inflammatory parameters and risk for repeated acute coronary syndrome in patients with ischemic heart disease. Vojnosanit Pregl. 2009 Jan;66(1):44-8.https://doi.org/10.2298/vsp0901044r

144. Ratajczak M, Skrypnik D, Bogdański P, Mądry E, Walkowiak J, Szulińska M, et al. Effects of Endurance and Endurance-Strength Training on Endothelial Function in Women with Obesity: A Randomized Trial. Int J Environ Res Public Health. 2019 Nov 5;16(21).https://doi.org/10.3390/ijerph16214291
145. Rech A, Botton CE, Lopez P, Quincozes-Santos A, Umpierre D, Pinto RS. Effects of short-term resistance training on endothelial function and inflammation markers in elderly patients with type 2 diabetes: A randomized controlled trial. Exp Gerontol. 2019 Apr;118:19-25.https://doi.org/10.1016/j.exger.2019.01.003
146. Redwine LS, Pung MA, Wilson K, Bangen KJ, Delano-Wood L, Hurwitz B. An exploratory randomized sub-study of light-to-moderate intensity exercise on cognitive function, depression symptoms and inflammation in older adults with heart failure. J Psychosom Res. 2020 Jan;128:109883.https://doi.org/10.1016/j.jpsychores.2019.109883
147. Reinehr T, Stoffel-Wagner B, Roth CL, Andler W. High-sensitive C-reactive protein, tumor necrosis factor alpha, and cardiovascular risk factors before and after weight loss in obese children. Metabolism. 2005 Sep;54(9):1155-61.https://doi.org/10.1016/j.metabol.2005.03.022
148. Rejeski WJ, Marsh AP, Fanning J, Ambrosius WT, Walkup MP, Nicklas BJ. Dietary Weight Loss, Exercise, and Inflammation in Older Adults with Overweight or Obesity and Cardiometabolic Disease. Obesity (Silver Spring). 2019 Nov;27(11):1805-11.https://doi.org/10.1002/oby.22600
149. Reljic D, Dieterich W, Herrmann HJ, Neurath MF, Zopf Y. "HIIT the Inflammation": Comparative Effects of Low-Volume Interval Training and Resistance Exercises on Inflammatory Indices in Obese Metabolic Syndrome Patients Undergoing Caloric Restriction. Nutrients. 2022 May 10;14(10).https://doi.org/10.3390/nu14101996
150. Roberts CK, Chen AK, Barnard RJ. Effect of a short-term diet and exercise intervention in youth on atherosclerotic risk factors. Atherosclerosis. 2007;191(1):98-106
151. Roche J, Isacco L, Perret F, Dumoulin G, Gillet V, Mougin F. Beneficial effects of a lifestyle intervention program on C-reactive protein: impact of cardiorespiratory fitness in obese adolescents with sleep disturbances. Am J Physiol Regul Integr Comp Physiol. 2019 Apr 1;316(4):R376-r86.https://doi.org/10.1152/ajpregu.00309.2018
152. Rodrigues-Krause J, Farinha JB, Ramis TR, Macedo RCO, Boeno FP, Dos Santos GC, et al. Effects of dancing compared to walking on cardiovascular risk and functional capacity of older women: A randomized controlled trial. Exp Gerontol. 2018 Dec;114:67-77.https://doi.org/10.1016/j.exger.2018.10.015
153. Rohde C, Polcwiartek C, Andersen E, Vang T, Nielsen J. Effect of a physical activity intervention on suPAR levels: A randomized controlled trial. J Sci Med Sport. 2018 Mar;21(3):286-90.https://doi.org/10.1016/j.jsams.2017.06.018
154. Rokling-Andersen MH, Reseland JE, Veierød MB, Anderssen SA, Jacobs DR, Jr., Urdal P, et al. Effects of long-term exercise and diet intervention on plasma adipokine concentrations. Am J Clin Nutr. 2007 Nov;86(5):1293-301.https://doi.org/10.1093/ajcn/86.5.1293
155. Ryan AS, Ge S, Blumenthal JB, Serra MC, Prior SJ, Goldberg AP. Aerobic exercise and weight loss reduce vascular markers of inflammation and improve insulin sensitivity in obese women. J Am Geriatr Soc. 2014 Apr;62(4):607-14.https://doi.org/10.1111/jgs.12749
156. Sabouri M, Hatami E, Pournemati P, Shabkhiz F. Inflammatory, antioxidant and glycemic status to different mode of high-intensity training in type 2 diabetes mellitus. Mol Biol Rep. 2021 Jun;48(6):5291-304.https://doi.org/10.1007/s11033-021-06539-y
157. Saeidi A, Seifi-Ski-Shahr F, Soltani M, Daraei A, Shirvani H, Laher I, et al. Resistance training, gremlin 1 and macrophage migration inhibitory factor in obese men: a randomised trial. Arch Physiol Biochem. 2023 Jun;129(3):640-8.https://doi.org/10.1080/13813455.2020.1856142
158. Santiprabhob J, Limprayoon K, Aanpreung P, Charoensakdi R, Kalpravidh RW, Phonrat B, et al. Impact of a group-based treatment program on adipocytokines, oxidative status, inflammatory cytokines and arterial stiffness in obese children and adolescents. J Pediatr Endocrinol Metab. 2018 Jul 26;31(7):733-42.https://doi.org/10.1515/jpem-2018-0012
159. Santomauro M, Paoli-Valeri M, Fernández M, Camacho N, Molina Z, Cicchetti R, et al. [Non-alcoholic fatty liver disease and its association with clinical and biochemical variables in obese children and adolescents: effect of a one-year intervention on lifestyle]. Endocrinol Nutr. 2012 Jun-Jul;59(6):346-53.https://doi.org/10.1016/j.endonu.2012.05.002 (Hígado graso no alcohólico y su asociación con variables clínicas y bioquímicas en niños y adolescentes obesos: efecto de un año de intervención en el estilo de vida.)

160. Scott E, Daley AJ, Doll H, Woodroofe N, Coleman RE, Mutrie N, et al. Effects of an exercise and hypocaloric healthy eating program on biomarkers associated with long-term prognosis after early-stage breast cancer: a randomized controlled trial. Cancer Causes Control. 2013 Jan;24(1):181-91.https://doi.org/10.1007/s10552-012-0104-x
161. Seo YG, Lim H, Kim Y, Ju YS, Lee HJ, Jang HB, et al. The Effect of a Multidisciplinary Lifestyle Intervention on Obesity Status, Body Composition, Physical Fitness, and Cardiometabolic Risk Markers in Children and Adolescents with Obesity. Nutrients. 2019 Jan 10;11(1).https://doi.org/10.3390/nu11010137
162. Shabani A, Shabani R, Dalili S, Hassanzadeh Rad A. The effect of concurrent endurance and resistance training on cardio-respiratory capacity and cardiovascular risk markers among sedentary overweight or obese post-menopausal women. Journal of Nursing and Midwifery Sciences. 2018 01/01;5:123.https://doi.org/10.4103/JNMS.JNMS_34_18
163. Shalitin S, Ashkenazi-Hoffnung L, Yackobovitch-Gavan M, Nagelberg N, Karni Y, Hershkovitz E, et al. Effects of a twelve-week randomized intervention of exercise and/or diet on weight loss and weight maintenance, and other metabolic parameters in obese preadolescent children. Horm Res. 2009;72(5):287-301.https://doi.org/10.1159/000245931
164. Shin YO, Bae JS, Lee JB, Kim JK, Kim YJ, Kim C, et al. Effect of cardiac rehabilitation and statin treatment on anti-HSP antibody titers in patients with coronary artery disease after percutaneous coronary intervention. Int Heart J. 2006 Sep;47(5):671-82.https://doi.org/10.1536/ihj.47.671
165. Smith DT, Carr LJ, Dorozynski C, Gomashe C. Internet-delivered lifestyle physical activity intervention: limited inflammation and antioxidant capacity efficacy in overweight adults. J Appl Physiol (1985). 2009 Jan;106(1):49-56.https://doi.org/10.1152/japplphysiol.90557.2008
166. Snel M, van Diepen JA, Stijnen T, Pijl H, Romijn JA, Meinders AE, et al. Immediate and long-term effects of addition of exercise to a 16-week very low calorie diet on low-grade inflammation in obese, insulin-dependent type 2 diabetic patients. Food Chem Toxicol. 2011 Dec;49(12):3104-11.https://doi.org/10.1016/j.fct.2011.09.032
167. Stewart LK, Earnest CP, Blair SN, Church TS. Effects of different doses of physical activity on C-reactive protein among women. Med Sci Sports Exerc. 2010 Apr;42(4):701-7.https://doi.org/10.1249/MSS.0b013e3181c03a2b
168. Straznicky NE, Lambert EA, Nestel PJ, McGrane MT, Dawood T, Schlaich MP, et al. Sympathetic neural adaptation to hypocaloric diet with or without exercise training in obese metabolic syndrome subjects. Diabetes. 2010 Jan;59(1):71-9.https://doi.org/10.2337/db09-0934
169. Straznicky NE, Grima MT, Lambert EA, Eikelis N, Dawood T, Lambert GW, et al. Exercise augments weight loss induced improvement in renal function in obese metabolic syndrome individuals. J Hypertens. 2011 Mar;29(3):553-64.https://doi.org/10.1097/HJH.0b013e3283418875
170. Streb AR, Braga PGS, de Melo RF, Botelho LJ, Maranhão RC, Del Duca GF. Effects of combined physical exercise on plasma lipid variables, paraoxonase 1 activity, and inflammation parameters in adults with obesity: a randomized clinical trial. J Endocrinol Invest. 2022 Oct;45(10):1991-7.https://doi.org/10.1007/s40618-022-01833-3
171. Sturgeon KM, Brown JC, Sears DD, Sarwer DB, Schmitz KH. WISER Survivor Trial: Combined Effect of Exercise and Weight Loss Interventions on Inflammation in Breast Cancer Survivors. Med Sci Sports Exerc. 2023 Feb 1;55(2):209-15.https://doi.org/10.1249/mss.0000000000003050
172. Sukala WR, Page R, Rowlands DS, Krebs J, Lys I, Leikis M, et al. South Pacific Islanders resist type 2 diabetes: comparison of aerobic and resistance training. Eur J Appl Physiol. 2012 Jan;112(1):317-25.https://doi.org/10.1007/s00421-011-1978-0
173. Swift DL, Johannsen NM, Earnest CP, Blair SN, Church TS. Effect of exercise training modality on C-reactive protein in type 2 diabetes. Med Sci Sports Exerc. 2012 Jun;44(6):1028-34.https://doi.org/10.1249/MSS.0b013e31824526cc
174. Tang Q, Ruan H, Tao Y, Zheng X, Shen X, Cai W. Effects of a summer program for weight management in obese children and adolescents in Shanghai. Asia Pac J Clin Nutr. 2014;23(3):459-64.https://doi.org/10.6133/apjcn.2014.23.3.02
175. Tartibian B, FitzGerald LZ, Azadpour N, Maleki BH. A randomized controlled study examining the effect of exercise on inflammatory cytokine levels in post-menopausal women. Post Reprod Health. 2015 Mar;21(1):9-15.https://doi.org/10.1177/2053369114565708

176. Thompson D, Markovitch D, Betts JA, Mazzatti D, Turner J, Tyrrell RM. Time course of changes in inflammatory markers during a 6-mo exercise intervention in sedentary middle-aged men: a randomized-controlled trial. J Appl Physiol (1985). 2010 Apr;108(4):769-79.https://doi.org/10.1152/japplphysiol.00822.2009
177. Tisi PV, Hulse M, Chulakadabba A, Gosling P, Shearman CP. Exercise training for intermittent claudication: does it adversely affect biochemical markers of the exercise-induced inflammatory response? Eur J Vasc Endovasc Surg. 1997 Nov;14(5):344-50.https://doi.org/10.1016/s1078-5884(97)80283-3
178. Tomeleri CM, Ribeiro AS, Souza MF, Schiavoni D, Schoenfeld BJ, Venturini D, et al. Resistance training improves inflammatory level, lipid and glycemic profiles in obese older women: A randomized controlled trial. Exp Gerontol. 2016 Nov;84:80-7.https://doi.org/10.1016/j.exger.2016.09.005
179. Tomeleri CM, Souza MF, Burini RC, Cavaglieri CR, Ribeiro AS, Antunes M, et al. Resistance training reduces metabolic syndrome and inflammatory markers in older women: A randomized controlled trial. J Diabetes. 2018 Apr;10(4):328-37.https://doi.org/10.1111/1753-0407.12614
180. Trussardi Fayh AP, Lopes AL, Fernandes PR, Reischak-Oliveira A, Friedman R. Impact of weight loss with or without exercise on abdominal fat and insulin resistance in obese individuals: a randomised clinical trial. Br J Nutr. 2013 Aug 28;110(3):486-92.https://doi.org/10.1017/s0007114512005442
181. Urzi F, Marusic U, Ličen S, Buzan E. Effects of Elastic Resistance Training on Functional Performance and Myokines in Older Women-A Randomized Controlled Trial. J Am Med Dir Assoc. 2019 Jul;20(7):830-4.e2.https://doi.org/10.1016/j.jamda.2019.01.151
182. Varady KA, Bhutani S, Church EC, Phillips SA. Adipokine responses to acute resistance exercise in trained and untrained men. Med Sci Sports Exerc. 2010 Mar;42(3):456-62.https://doi.org/10.1249/MSS.0b013e3181ba6dd3
183. Vasconcellos F, Seabra A, Cunha F, Montenegro R, Penha J, Bouskela E, et al. Health markers in obese adolescents improved by a 12-week recreational soccer program: a randomised controlled trial. J Sports Sci. 2016;34(6):564-75.https://doi.org/10.1080/02640414.2015.1064150
184. Vella CA, Taylor K, Drummer D. High-intensity interval and moderate-intensity continuous training elicit similar enjoyment and adherence levels in overweight and obese adults. Eur J Sport Sci. 2017 Oct;17(9):1203-11.https://doi.org/10.1080/17461391.2017.1359679
185. Venojärvi M, Wasenius N, Manderoos S, Heinonen OJ, Hernelahti M, Lindholm H, et al. Nordic walking decreased circulating chemerin and leptin concentrations in middle-aged men with impaired glucose regulation. Ann Med. 2013 Mar;45(2):162-70.https://doi.org/10.3109/07853890.2012.727020
186. Vieira VJ, Hu L, Valentine RJ, McAuley E, Evans EM, Baynard T, et al. Reduction in trunk fat predicts cardiovascular exercise training-related reductions in C-reactive protein. Brain Behav Immun. 2009 May;23(4):485-91.https://doi.org/10.1016/j.bbi.2009.01.011
187. Vos RC, Wit JM, Pijl H, Houdijk EC. Long-term effect of lifestyle intervention on adiposity, metabolic parameters, inflammation and physical fitness in obese children: a randomized controlled trial. Nutr Diabetes. 2011 Oct 3;1(10):e9.https://doi.org/10.1038/nutd.2011.5
188. Walther C, Möbius-Winkler S, Linke A, Bruegel M, Thiery J, Schuler G, et al. Regular exercise training compared with percutaneous intervention leads to a reduction of inflammatory markers and cardiovascular events in patients with coronary artery disease. Eur J Cardiovasc Prev Rehabil. 2008 Feb;15(1):107-12.https://doi.org/10.1097/HJR.0b013e3282f29aa6
189. Wanderley FA, Moreira A, Sokhatska O, Palmares C, Moreira P, Sandercock G, et al. Differential responses of adiposity, inflammation and autonomic function to aerobic versus resistance training in older adults. Exp Gerontol. 2013 Mar;48(3):326-33.https://doi.org/10.1016/j.exger.2013.01.002
190. Wang CX PH, Qiu MC. Effects of exercises on serum concentrations of TNF-α, IL-6 and CRP in subjects with overweight and obesity. 2006; 2006.
191. Wang R, Chen PJ, Chen WH. Diet and exercise improve neutrophil to lymphocyte ratio in overweight adolescents. Int J Sports Med. 2011 Dec;32(12):982-6.https://doi.org/10.1055/s-0031-1283185
192. Wang S. Effects and mechanisms of high-intensity interval aerobic training on vascular endothelial function in obese adolescents. Shandong Medical Journal. 2018;58(20):45-7

193. Wedell-Neergaard AS, Lang Lehrskov L, Christensen RH, Legaard GE, Dorph E, Larsen MK, et al. Exercise-Induced Changes in Visceral Adipose Tissue Mass Are Regulated by IL-6 Signaling: A Randomized Controlled Trial. Cell Metab. 2019 Apr 2;29(4):844-55.e3.https://doi.org/10.1016/j.cmet.2018.12.007
194. Weiss EP, Albert SG, Reeds DN, Kress KS, McDaniel JL, Klein S, et al. Effects of matched weight loss from calorie restriction, exercise, or both on cardiovascular disease risk factors: a randomized intervention trial. Am J Clin Nutr. 2016 Sep;104(3):576-86.https://doi.org/10.3945/ajcn.116.131391
195. Winters-Stone KM, Wood LJ, Stoyles S, Dieckmann NF. The Effects of Resistance Exercise on Biomarkers of Breast Cancer Prognosis: A Pooled Analysis of Three Randomized Trials. Cancer Epidemiol Biomarkers Prev. 2018 Feb;27(2):146-53.https://doi.org/10.1158/1055-9965.Epi-17-0766
196. Wong PC, Chia MY, Tsou IY, Wansaicheong GK, Tan B, Wang JC, et al. Effects of a 12-week exercise training programme on aerobic fitness, body composition, blood lipids and C-reactive protein in adolescents with obesity. Ann Acad Med Singap. 2008 Apr;37(4):286-93
197. Wong A, Sanchez-Gonzalez MA, Son WM, Kwak YS, Park SY. The Effects of a 12-Week Combined Exercise Training Program on Arterial Stiffness, Vasoactive Substances, Inflammatory Markers, Metabolic Profile, and Body Composition in Obese Adolescent Girls. Pediatr Exerc Sci. 2018 Nov 1;30(4):480-6.https://doi.org/10.1123/pes.2017-0198
198. Wycherley TP, Noakes M, Clifton PM, Cleanthous X, Keogh JB, Brinkworth GD. A high-protein diet with resistance exercise training improves weight loss and body composition in overweight and obese patients with type 2 diabetes. Diabetes Care. 2010;33(5):969-76
199. Yeh GY, McCarthy EP, Wayne PM, Stevenson LW, Wood MJ, Forman D, et al. Tai chi exercise in patients with chronic heart failure: a randomized clinical trial. Arch Intern Med. 2011 Apr 25;171(8):750-7.https://doi.org/10.1001/archinternmed.2011.150
200. Yin C, Hu W, Wang M, Lv W, Jia T, Xiao Y. Irisin as a mediator between obesity and vascular inflammation in Chinese children and adolescents. Nutr Metab Cardiovasc Dis. 2020 Feb 10;30(2):320-9.https://doi.org/10.1016/j.numecd.2019.09.025
201. Yoon JR, Ha GC, Ko KJ, Kang SJ. Effects of exercise type on estrogen, tumor markers, immune function, antioxidant function, and physical fitness in postmenopausal obese women. J Exerc Rehabil. 2018 Dec;14(6):1032-40.https://doi.org/10.12965/jer.1836446.223
202. You T, Berman DM, Ryan AS, Nicklas BJ. Effects of hypocaloric diet and exercise training on inflammation and adipocyte lipolysis in obese postmenopausal women. J Clin Endocrinol Metab. 2004 Apr;89(4):1739-46.https://doi.org/10.1210/jc.2003-031310
203. Zhang H, Tong TK, Qiu W, Zhang X, Zhou S, Liu Y, et al. Comparable Effects of High-Intensity Interval Training and Prolonged Continuous Exercise Training on Abdominal Visceral Fat Reduction in Obese Young Women. J Diabetes Res. 2017;2017:5071740.https://doi.org/10.1155/2017/5071740
204. Zhang LY, Liu T, Teng YQ, Yao XY, Zhao TT, Lin LY, et al. Effect of a 12-Week Aerobic Exercise Training on Serum Fetuin-A and Adipocytokine Levels in Type 2 Diabetes. Exp Clin Endocrinol Diabetes. 2018 Sep;126(8):487-92.https://doi.org/10.1055/s-0043-115904
205. ZHAO Jun LJ. Improvement effects of aerobic exercise combined with resistance training on body composition, cardiovascular function and serum C-reactive protein level in male obese college students. Journal of Jilin University(Medicine Edition). 2019 2019-09-28;45(05):1134-40.https://doi.org/10.13481/j.1671-587x.20190527
206. Abd El-Kader SM, Al-Jiffri OH, Al-Shreef FM. Aerobic exercises alleviate symptoms of fatigue related to inflammatory cytokines in obese patients with type 2 diabetes. Afr Health Sci. 2015 Dec;15(4):1142-8.https://doi.org/10.4314/ahs.v15i4.13
207. Abd El-Kader SM, Al-Jiffri OH, Neamatallah ZA, AlKhateeb AM, AlFawaz SS. Weight reduction ameliorates inflammatory cytokines, adipocytokines and endothelial dysfunction biomarkers among Saudi patients with type 2 diabetes. Afr Health Sci. 2020 Sep;20(3):1329-36.https://doi.org/10.4314/ahs.v20i3.39
208. Abdollahpour A, Khosravi N, Eskandari Z, Haghighat S. Effect of Six Months of Aerobic Exercise on Plasma Interleukin-6 and Tumor Necrosis Factor-Alpha as Breast Cancer Risk Factors in Postmenopausal Women: A Randomized Controlled Trial. 2017; 2017.

209. Adamopoulos S, Parissis J, Karatzas D, Kroupis C, Georgiadis M, Karavolias G, et al. Physical training modulates proinflammatory cytokines and the soluble Fas/soluble Fas ligand system in patients with chronic heart failure. J Am Coll Cardiol. 2002 Feb 20;39(4):653-63.https://doi.org/10.1016/s0735-1097(01)01795-8
210. Ahmadizad S, Avansar AS, Ebrahim K, Avandi M, Ghasemikaram M. The effects of short-term high-intensity interval training vs. moderate-intensity continuous training on plasma levels of nesfatin-1 and inflammatory markers. Horm Mol Biol Clin Investig. 2015 Mar;21(3):165-73.https://doi.org/10.1515/hmbci-2014-0038
211. Ben Ounis O, Elloumi M, Lac G, Makni E, Van Praagh E, Zouhal H, et al. Two-month effects of individualized exercise training with or without caloric restriction on plasma adipocytokine levels in obese female adolescents. Ann Endocrinol (Paris). 2009 Sep;70(4):235-41.https://doi.org/10.1016/j.ando.2009.03.003
212. Biteli P, Barbalho SM, Detregiachi CRP, Dos Santos Haber JF, Chagas EFB. Dyslipidemia influences the effect of physical exercise on inflammatory markers on obese women in post-menopause: A randomized clinical trial. Exp Gerontol. 2021 Jul 15;150:111355.https://doi.org/10.1016/j.exger.2021.111355
213. Chagas EFB, Bonfim MR, Turi BC, Brondino NCM, Monteiro HL. Effect of Moderate-Intensity Exercise on Inflammatory Markers Among Postmenopausal Women. J Phys Act Health. 2017 Jun;14(6):479-85.https://doi.org/10.1123/jpah.2016-0319
214. Chow BC, Li S, Zhu X, Jiao J, Quach B, Baker JS, et al. Effects of descending or ascending stair exercise on body composition, insulin sensitivity, and inflammatory markers in young Chinese women with obesity: A randomized controlled trial. J Sports Sci. 2021 Mar;39(5):496-502.https://doi.org/10.1080/02640414.2020.1829362
215. Chupel MU, Direito F, Furtado GE, Minuzzi LG, Pedrosa FM, Colado JC, et al. Strength Training Decreases Inflammation and Increases Cognition and Physical Fitness in Older Women with Cognitive Impairment. Front Physiol. 2017;8:377.https://doi.org/10.3389/fphys.2017.00377
216. Chupel MU, Minuzzi LG, Furtado G, Santos ML, Hogervorst E, Filaire E, et al. Exercise and taurine in inflammation, cognition, and peripheral markers of blood-brain barrier integrity in older women. Appl Physiol Nutr Metab. 2018 Jul;43(7):733-41.https://doi.org/10.1139/apnm-2017-0775
217. Conraads VM, Beckers P, Bosmans J, De Clerck LS, Stevens WJ, Vrints CJ, et al. Combined endurance/resistance training reduces plasma TNF-alpha receptor levels in patients with chronic heart failure and coronary artery disease. Eur Heart J. 2002 Dec;23(23):1854-60.https://doi.org/10.1053/euhj.2002.3239
218. Erbs S, Höllriegel R, Linke A, Beck EB, Adams V, Gielen S, et al. Exercise training in patients with advanced chronic heart failure (NYHA IIIb) promotes restoration of peripheral vasomotor function, induction of endogenous regeneration, and improvement of left ventricular function. Circ Heart Fail. 2010 Jul;3(4):486-94.https://doi.org/10.1161/circheartfailure.109.868992
219. Ergun M, Eyigor S, Karaca B, Kisim A, Uslu R. Effects of exercise on angiogenesis and apoptosis-related molecules, quality of life, fatigue and depression in breast cancer patients. Eur J Cancer Care (Engl). 2013 Sep;22(5):626-37.https://doi.org/10.1111/ecc.12068
220. Feiereisen P, Vaillant M, Gilson G, Delagardelle C. Effects of different training modalities on circulating anabolic/catabolic markers in chronic heart failure. Journal of cardiopulmonary rehabilitation and prevention. 2013 2013 Sep-Oct;33(5):303-8.https://doi.org/10.1097/hcr.0b013e3182a1e4e5
221. Fernandes-Silva MM, Guimarães GV, Rigaud VO, Lofrano-Alves MS, Castro RE, de Barros Cruz LG, et al. Inflammatory biomarkers and effect of exercise on functional capacity in patients with heart failure: Insights from a randomized clinical trial. Eur J Prev Cardiol. 2017 May;24(8):808-17.https://doi.org/10.1177/2047487317690458
222. Galedari M, Azarbayjani MA, Peeri M. Effects of type of exercise along with caloric restriction on plasma apelin 36 and HOMA-IR in overweight men. Science & Sports. 2017;32
223. Gielen S, Sandri M, Kozarez I, Kratzsch J, Teupser D, Thiery J, et al. Exercise training attenuates MuRF-1 expression in the skeletal muscle of patients with chronic heart failure independent of age: the randomized Leipzig Exercise Intervention in Chronic Heart Failure and Aging catabolism study. Circulation. 2012 Jun 5;125(22):2716-27.https://doi.org/10.1161/circulationaha.111.047381
224. Ho SS, Dhaliwal SS, Hills AP, Pal S. Effects of chronic exercise training on inflammatory markers in Australian overweight and obese individuals in a randomized controlled trial. Inflammation. 2013 Jun;36(3):625-32.https://doi.org/10.1007/s10753-012-9584-9

225. Kadoglou NP, Iliadis F, Liapis CD, Perrea D, Angelopoulou N, Alevizos M. Beneficial effects of combined treatment with rosiglitazone and exercise on cardiovascular risk factors in patients with type 2 diabetes. Diabetes Care. 2007;30(9):2242-4.https://doi.org/10.2337/dc07-0341
226. Karavidas AI, Raisakis KG, Parissis JT, Tsekoura DK, Adamopoulos S, Korres DA, et al. Functional electrical stimulation improves endothelial function and reduces peripheral immune responses in patients with chronic heart failure. Eur J Cardiovasc Prev Rehabil. 2006 Aug;13(4):592-7.https://doi.org/10.1097/01.hjr.0000219111.02544.ff
227. Lakhdar N, Denguezli M, Zaouali M, Zbidi A, Tabka Z, Bouassida A. Diet and Diet Combined with Chronic Aerobic Exercise Decreases Body Fat Mass and Alters Plasma and Adipose Tissue Inflammatory Markers in Obese Women. Inflammation. 2013 2013/12/01;36(6):1239-47.https://doi.org/10.1007/s10753-013-9661-8
228. Larsen AI, Aukrust P, Aarsland T, Dickstein K. Effect of aerobic exercise training on plasma levels of tumor necrosis factor alpha in patients with heart failure. Am J Cardiol. 2001 Oct 1;88(7):805-8.https://doi.org/10.1016/s0002-9149(01)01859-8
229. Linke A, Adams V, Schulze PC, Erbs S, Gielen S, Fiehn E, et al. Antioxidative effects of exercise training in patients with chronic heart failure: increase in radical scavenger enzyme activity in skeletal muscle. Circulation. 2005 Apr 12;111(14):1763-70.https://doi.org/10.1161/01.Cir.0000165503.08661.E5
230. Martins FM, Santagnello SB, de Oliveira Junior GN, de Sousa JFR, Michelin MA, Nomelini RS, et al. Lower-Body Resistance Training Reduces Interleukin-1β and Transforming Growth Factor-β1 Levels and Fatigue and Increases Physical Performance in Breast Cancer Survivors. J Strength Cond Res. 2023 Feb 1;37(2):439-51.https://doi.org/10.1519/jsc.0000000000004270
231. Melo X, Abreu A, Santos V, Cunha P, Oliveira M, Pinto R, et al. A Post hoc analysis on rhythm and high intensity interval training in cardiac resynchronization therapy. Scand Cardiovasc J. 2019 Aug;53(4):197-205.https://doi.org/10.1080/14017431.2019.1630747
232. Mendham AE, Duffield R, Marino F, Coutts AJ. Small-sided games training reduces CRP, IL-6 and leptin in sedentary, middle-aged men. Eur J Appl Physiol. 2014 Nov;114(11):2289-97.https://doi.org/10.1007/s00421-014-2953-3
233. Munk PS, Breland UM, Aukrust P, Ueland T, Kvaløy JT, Larsen AI. High intensity interval training reduces systemic inflammation in post-PCI patients. European Journal of Cardiovascular Prevention & Rehabilitation. 2011;18:850 - 7
234. Nambi G, Alghadier M, Elnegamy TE, Basuodan RM, Alwhaibi RM, Vellaiyan A, et al. Clinical (BMI and MRI) and Biochemical (Adiponectin, Leptin, TNF-α, and IL-6) Effects of High-Intensity Aerobic Training with High-Protein Diet in Children with Obesity Following COVID-19 Infection. Int J Environ Res Public Health. 2022 Jun 11;19(12).https://doi.org/10.3390/ijerph19127194
235. Niebauer J, Clark AL, Webb-Peploe KM, Coats AJ. Exercise training in chronic heart failure: effects on pro-inflammatory markers. Eur J Heart Fail. 2005 Mar 2;7(2):189-93.https://doi.org/10.1016/j.ejheart.2004.07.012
236. Nikseresht M, Agha-Alinejad H, Azarbayjani MA, Ebrahim K. Effects of nonlinear resistance and aerobic interval training on cytokines and insulin resistance in sedentary men who are obese. J Strength Cond Res. 2014 Sep;28(9):2560-8.https://doi.org/10.1519/jsc.0000000000000441
237. Park SM, Kwak YS, Ji JG. The Effects of Combined Exercise on Health-Related Fitness, Endotoxin, and Immune Function of Postmenopausal Women with Abdominal Obesity. J Immunol Res. 2015;2015:830567.https://doi.org/10.1155/2015/830567
238. Peña A, Olson ML, Ayers SL, Sears DD, Vega-López S, Colburn AT, et al. Inflammatory Mediators and Type 2 Diabetes Risk Factors before and in Response to Lifestyle Intervention among Latino Adolescents with Obesity. Nutrients. 2023 May 24;15(11).https://doi.org/10.3390/nu15112442
239. Racca V, Torri A, Grati P, Panzarino C, Marventano I, Saresella M, et al. Inflammatory Cytokines During Cardiac Rehabilitation After Heart Surgery and Their Association to Postoperative Atrial Fibrillation. Sci Rep. 2020 May 25;10(1):8618.https://doi.org/10.1038/s41598-020-65581-1

240. Rezende REF, Duarte SMB, Stefano JT, Roschel H, Gualano B, Pinto ALdS, et al. Randomized clinical trial: benefits of aerobic physical activity for 24 weeks in postmenopausal women with nonalcoholic fatty liver disease. Menopause. 2016;23(8):876-83.https://doi.org/10.1097/GME.0000000000000647
241. Rogers LQ, Fogleman A, Trammell R, Hopkins-Price P, Vicari S, Rao K, et al. Effects of a physical activity behavior change intervention on inflammation and related health outcomes in breast cancer survivors: pilot randomized trial. Integr Cancer Ther. 2013 Jul;12(4):323-35.https://doi.org/10.1177/1534735412449687
242. Rogers LQ, Vicari S, Trammell R, Hopkins-Price P, Fogleman A, Spenner A, et al. Biobehavioral factors mediate exercise effects on fatigue in breast cancer survivors. Med Sci Sports Exerc. 2014 Jun;46(6):1077-88.https://doi.org/10.1249/mss.0000000000000210
243. Salamat KM, Azarbayjani MA, Yusof A, Dehghan F. The response of pre-inflammatory cytokines factors to different exercises (endurance, resistance, concurrent) in overweight men. Alexandria Journal of Medicine. 2016 2016/12/01/;52(4):367-70.https://doi.org/10.1016/j.ajme.2015.12.007
244. Saxton JM, Scott EJ, Daley AJ, Woodroofe M, Mutrie N, Crank H, et al. Effects of an exercise and hypocaloric healthy eating intervention on indices of psychological health status, hypothalamic-pituitary-adrenal axis regulation and immune function after early-stage breast cancer: a randomised controlled trial. Breast Cancer Res. 2014 Apr 14;16(2):R39.https://doi.org/10.1186/bcr3643
245. Shahram S, Elham Yadegari Hemat A, Nader S. The Effect of Endurance and Resistance Training on Interleukin-6 and Tumor Necrosis Factor-α in Overweight Young Women. Sport Biosciences. 2016;8(29):263-76
246. Silverman NE, Nicklas BJ, Ryan AS. Addition of aerobic exercise to a weight loss program increases BMD, with an associated reduction in inflammation in overweight postmenopausal women. Calcif Tissue Int. 2009 Apr;84(4):257-65.https://doi.org/10.1007/s00223-009-9232-z
247. TaheriChadorneshin H, Cheragh-Birjandi S, Goodarzy S, Ahmadabadi F. The impact of high intensity interval training on serum chemerin, tumor necrosis factor-alpha and insulin resistance in overweight women. Obesity Medicine. 2019 2019/06/01/;14:100101.https://doi.org/10.1016/j.obmed.2019.100101
248. Trippel TD, Holzendorf V, Halle M, Gelbrich G, Nolte K, Duvinage A, et al. Ghrelin and hormonal markers under exercise training in patients with heart failure with preserved ejection fraction: results from the Ex-DHF pilot study. ESC Heart Fail. 2017 Feb;4(1):56-65.https://doi.org/10.1002/ehf2.12109
249. Tsarouhas K, Tsitsimpikou C, Haliassos A, Georgoulias P, Koutsioras I, Kouretas D, et al. Study of insulin resistance, TNF-α, total antioxidant capacity and lipid profile in patients with chronic heart failure under exercise. In Vivo. 2011 2011 Nov-Dec;25(6):1031-7
250. Vasconcelos ABS, Resende-Neto AG, Nogueira AC, Aragão-Santos JC, Monteiro MRP, Morais Junior GS, et al. Functional and traditional training improve muscle power and reduce proinflammatory cytokines in older women: A randomized controlled trial. Exp Gerontol. 2020 Jul 1;135:110920.https://doi.org/10.1016/j.exger.2020.110920
251. Abbenhardt C, McTiernan A, Alfano CM, Wener MH, Campbell KL, Duggan C, et al. Effects of individual and combined dietary weight loss and exercise interventions in postmenopausal women on adiponectin and leptin levels. J Intern Med. 2013 Aug;274(2):163-75.https://doi.org/10.1111/joim.12062
252. Ahmadi P, Torabi M, Aran Ardabili A, Aghazadeh J. Serum adiponectin and insulin sensitivity affect by aerobic exercise program in patients with type 2 diabetes mellitus. International Journal of Biosciences (IJB). 2012;2(9):94-101
253. Ahmadizad S, Haghighi AH, Hamedinia MR. Effects of resistance versus endurance training on serum adiponectin and insulin resistance index. Eur J Endocrinol. 2007 Nov;157(5):625-31.https://doi.org/10.1530/eje-07-0223
254. Armamento-Villareal R, Aguirre L, Waters DL, Napoli N, Qualls C, Villareal DT. Effect of Aerobic or Resistance Exercise, or Both, on Bone Mineral Density and Bone Metabolism in Obese Older Adults While Dieting: A Randomized Controlled Trial. J Bone Miner Res. 2020 Mar;35(3):430-9.https://doi.org/10.1002/jbmr.3905
255. Asad M, Ravasi AA, Faramarzi M, Pournemati P. The effects of three training methods endurance, resistance and concurrent on adiponectin resting levels in overweighed untrained men. Bratisl Lek Listy. 2012;113(11):664-8.https://doi.org/10.4149/bll_2012_150

256. Delavar S, Baigzadeh M, Safikhani H. The Effect of Training (Aerobic and Concurrent) and Cinnamon Supplementation on the Plasma Levels of Visfatin and Adiponectin in Overweight Diabetic Men. Journal of Clinical Research in Paramedical Sciences. 2020 07/25;In Press.https://doi.org/10.5812/jcrps.90906
257. Baitul Mukarromah S, Susanto H, Kushartanti W, Soegiyanto, Rahayu S. Beneficial Health Effect of Aquarobics (Role of Adiponectin on Women with Obesity). IOP Conference Series: Materials Science and Engineering. 2017 03/01;180:012170.https://doi.org/10.1088/1757-899X/180/1/012170
258. Balducci S, Zanuso S, Nicolucci A, De Feo P, Cavallo S, Cardelli P, et al. Effect of an intensive exercise intervention strategy on modifiable cardiovascular risk factors in subjects with type 2 diabetes mellitus: a randomized controlled trial: the Italian Diabetes and Exercise Study (IDES). Arch Intern Med. 2010 Nov 8;170(20):1794-803.https://doi.org/10.1001/archinternmed.2010.380
259. Beavers KM, Ambrosius WT, Nicklas BJ, Rejeski WJ. Independent and combined effects of physical activity and weight loss on inflammatory biomarkers in overweight and obese older adults. J Am Geriatr Soc. 2013 Jul;61(7):1089-94.https://doi.org/10.1111/jgs.12321
260. Boudou P, Sobngwi E, Mauvais-Jarvis F, Vexiau P, Gautier JF. Absence of exercise-induced variations in adiponectin levels despite decreased abdominal adiposity and improved insulin sensitivity in type 2 diabetic men. Eur J Endocrinol. 2003 Nov;149(5):421-4.https://doi.org/10.1530/eje.0.1490421
261. Zilaei Bouri S, Peeri M, Azarbayjani MA. The Effect of Physical Activity on Adiponectin and Osteocalcin in Overweight Young Females. International Medical Journal. 2015 01/01;22:43-6
262. Corpeleijn E, Feskens EJ, Jansen EH, Mensink M, Saris WH, Blaak EE. Lifestyle intervention and adipokine levels in subjects at high risk for type 2 diabetes: the Study on Lifestyle intervention and Impaired glucose tolerance Maastricht (SLIM). Diabetes Care. 2007 Dec;30(12):3125-7.https://doi.org/10.2337/dc07-0457
263. de Souza F, da Silva LA, Ferreira GS, de Souza MMM, Bobinski F, Palandi J, et al. Karate Training Improves Metabolic Health in Overweight and Obese Adolescents: A Randomized Clinical Trial. Pediatr Exerc Sci. 2022 May 1;34(2):108-18.https://doi.org/10.1123/pes.2020-0193
264. Dieli-Conwright CM, Parmentier JH, Sami N, Lee K, Spicer D, Mack WJ, et al. Adipose tissue inflammation in breast cancer survivors: effects of a 16-week combined aerobic and resistance exercise training intervention. Breast Cancer Res Treat. 2018 Feb;168(1):147-57.https://doi.org/10.1007/s10549-017-4576-y
265. Arslan M, Ipekci SH, Kebapcilar L, Dogan Dede N, Kurban S, Erbay E, et al. Effect of Aerobic Exercise Training on MDA and TNF- α Levels in Patients with Type 2 Diabetes Mellitus. Int Sch Res Notices. 2014;2014:820387.https://doi.org/10.1155/2014/820387
266. Dede N, Ipekci S, Kebapcilar L, Arslan M, Kurban S, Yildiz M, et al. Influence of Exercise on Leptin, Adiponectin and Quality of Life in Type 2 Diabetics. Turkish Journal of Endocrinology and Metabolism. 2015 03/05;19.https://doi.org/10.4274/tjem.2564
267. Eizadi M, Khorshidi D, Dooaly H, Samarikhalaj H. Adiponectin and insulin resistance responses to aerobic training in males with abdominal obesity. Journal of Biodiversity and Environmental Sciences (JBES). 2011;1(6):228-35
268. Fatouros IG, Tournis S, Leontsini D, Jamurtas AZ, Sxina M, Thomakos P, et al. Leptin and adiponectin responses in overweight inactive elderly following resistance training and detraining are intensity related. J Clin Endocrinol Metab. 2005 Nov;90(11):5970-7.https://doi.org/10.1210/jc.2005-0261
269. Fazelifar S, Ebrahim K, Sarkisian V. Effect of concurrent training and detraining on anti-inflammatory biomarker and physical fitness levels in obese children. Revista Brasileira de Medicina do Esporte. 2013 10/01;19:349-54.https://doi.org/10.1590/S1517-86922013000500010
270. Figueroa A, Vicil F, Sanchez-Gonzalez MA, Wong A, Ormsbee MJ, Hooshmand S, et al. Effects of diet and/or low-intensity resistance exercise training on arterial stiffness, adiposity, and lean mass in obese postmenopausal women. Am J Hypertens. 2013 Mar;26(3):416-23.https://doi.org/10.1093/ajh/hps050
271. Friedenreich CM, Neilson HK, Woolcott CG, McTiernan A, Wang Q, Ballard-Barbash R, et al. Changes in insulin resistance indicators, IGFs, and adipokines in a year-long trial of aerobic exercise in postmenopausal women. Endocr Relat Cancer. 2011 Jun;18(3):357-69.https://doi.org/10.1530/erc-10-0303

272. Hara T, Fujiwara H, Nakao H, Mimura T, Yoshikawa T, Fujimoto S. Body composition is related to increase in plasma adiponectin levels rather than training in young obese men. Eur J Appl Physiol. 2005 Aug;94(5-6):520-6.https://doi.org/10.1007/s00421-005-1374-8
273. Ibáñez J, Izquierdo M, Martínez-Labari C, Ortega F, Grijalba A, Forga L, et al. Resistance Training Improves Cardiovascular Risk Factors in Obese Women Despite a Significative Decrease in Serum Adiponectin Levels. Obesity. 2010;18(3):535-41.https://doi.org/10.1038/oby.2009.277
274. Kahhan N, Hossain MJ, Lang J, Harrison C, Canas J, Wysocki T, et al. Durability of Changes in Biomarkers of Cardiometabolic Disease: 1-Year Family-Based Intervention in Children with Obesity. Metab Syndr Relat Disord. 2021 Jun;19(5):264-71.https://doi.org/10.1089/met.2020.0097
275. Kim YS, Nam JS, Yeo DW, Kim KR, Suh SH, Ahn CW. The effects of aerobic exercise training on serum osteocalcin, adipocytokines and insulin resistance on obese young males. Clin Endocrinol (Oxf). 2015 May;82(5):686-94.https://doi.org/10.1111/cen.12601
276. Kim TH, Chang JS, Park KS, Park J, Kim N, Lee JI, et al. Effects of exercise training on circulating levels of Dickkpof-1 and secreted frizzled-related protein-1 in breast cancer survivors: A pilot single-blind randomized controlled trial. PLoS One. 2017;12(2):e0171771.https://doi.org/10.1371/journal.pone.0171771
277. Konopko-Zubrzycka M, Baniukiewicz A, Wróblewski E, Kowalska I, Zarzycki W, Górska M, et al. The effect of intragastric balloon on plasma ghrelin, leptin, and adiponectin levels in patients with morbid obesity. J Clin Endocrinol Metab. 2009 May;94(5):1644-9.https://doi.org/10.1210/jc.2008-1083
278. Kortas J, Ziemann E, Juszczak D, Micielska K, Kozłowska M, Prusik K, et al. Iron Status in Elderly Women Impacts Myostatin, Adiponectin and Osteocalcin Levels Induced by Nordic Walking Training. Nutrients. 2020 Apr 17;12(4).https://doi.org/10.3390/nu12041129
279. Ku YH, Han KA, Ahn H, Kwon H, Koo BK, Kim HC, et al. Resistance exercise did not alter intramuscular adipose tissue but reduced retinol-binding protein-4 concentration in individuals with type 2 diabetes mellitus. J Int Med Res. 2010 May-Jun;38(3):782-91.https://doi.org/10.1177/147323001003800305
280. Ligibel JA, Campbell N, Partridge A, Chen WY, Salinardi T, Chen H, et al. Impact of a mixed strength and endurance exercise intervention on insulin levels in breast cancer survivors. J Clin Oncol. 2008 Feb 20;26(6):907-12.https://doi.org/10.1200/jco.2007.12.7357
281. Ligibel JA, Giobbie-Hurder A, Olenczuk D, Campbell N, Salinardi T, Winer EP, et al. Impact of a mixed strength and endurance exercise intervention on levels of adiponectin, high molecular weight adiponectin and leptin in breast cancer survivors. Cancer Causes Control. 2009 Oct;20(8):1523-8.https://doi.org/10.1007/s10552-009-9358-3
282. Lin D, Sturgeon KM, Gordon BR, Brown JC, Sears DD, Sarwer DB, et al. WISER Survivor Trial: Combined Effect of Exercise and Weight Loss Interventions on Adiponectin and Leptin Levels in Breast Cancer Survivors with Overweight or Obesity. Nutrients. 2023 Aug 4;15(15).https://doi.org/10.3390/nu15153453
283. Mendez-Gutierrez A, Aguilera CM, Osuna-Prieto FJ, Martinez-Tellez B, Rico Prados MC, Acosta FM, et al. Exercise-induced changes on exerkines that might influence brown adipose tissue metabolism in young sedentary adults. European Journal of Sport Science. 2023;23(4):625-36.https://doi.org/10.1080/17461391.2022.2040597
284. Moradi F. Changes of Serum Adiponectin and Testosterone Concentrations Following Twelve Weeks Resistance Training in Obese Young Men. Asian J Sports Med. 2015 Dec;6(4):e23808.https://doi.org/10.5812/asjsm.23808
285. Parsian H, Eizadi M, Khorshidi D, Khanali F. The effect of long-term aerobic exercise on serum adiponectin and insulin sensitivity in type 2 diabetic patients. Pars of Jahrom University of Medical Sciences. 2013 04/01;11:41-8.https://doi.org/10.29252/jmj.11.1.6
286. Pedrosa C, Oliveira BM, Albuquerque I, Simões-Pereira C, Vaz-de-Almeida MD, Correia F. Metabolic syndrome, adipokines and ghrelin in overweight and obese schoolchildren: results of a 1-year lifestyle intervention programme. Eur J Pediatr. 2011 Apr;170(4):483-92.https://doi.org/10.1007/s00431-010-1316-2
287. Pourvaghar MJ, Bahram ME, Sayyah M, Khoshemehry S. The Effects of Three Months of HIIT on Plasma Adiponectin on Overweight College Men. World Academy of Science, Engineering and Technology, International Journal of Medical, Health, Biomedical, Bioengineering and Pharmaceutical Engineering. 2017;11:448-51

288. Pasqualini L, Schillaci G, Innocente S, Pucci G, Coscia F, Siepi D, et al. Lifestyle intervention improves microvascular reactivity and increases serum adiponectin in overweight hypertensive patients. Nutr Metab Cardiovasc Dis. 2010 Feb;20(2):87-92.https://doi.org/10.1016/j.numecd.2009.03.002
289. Racil G, Ben Ounis O, Hammouda O, Kallel A, Zouhal H, Chamari K, et al. Effects of high vs. moderate exercise intensity during interval training on lipids and adiponectin levels in obese young females. Eur J Appl Physiol. 2013 Oct;113(10):2531-40.https://doi.org/10.1007/s00421-013-2689-5
290. Racil G, Zouhal H, Elmontassar W, Ben Abderrahmane A, De Sousa MV, Chamari K, et al. Plyometric exercise combined with high-intensity interval training improves metabolic abnormalities in young obese females more so than interval training alone. Appl Physiol Nutr Metab. 2016 Jan;41(1):103-9.https://doi.org/10.1139/apnm-2015-0384
291. Mohammad Rahimi GR, Bijeh N, Rashidlamir A. Effects of exercise training on serum preptin, undercarboxylated osteocalcin and high molecular weight adiponectin in adults with metabolic syndrome. Exp Physiol. 2020 Mar;105(3):449-59.https://doi.org/10.1113/ep088036
292. Rashidlamir A, Saadatnia A. The effect of eight weeks of aerobic training on the plasma level of adiponectin, leptin, and resistin in healthy middle-aged men. Science & Sports. 2012 2012/12/01/;27(6):351-6.https://doi.org/10.1016/j.scispo.2011.11.004
293. Saeidi A, Jabbour G, Ahmadian M, Abbassi-Daloii A, Malekian F, Hackney AC, et al. Independent and Combined Effects of Antioxidant Supplementation and Circuit Resistance Training on Selected Adipokines in Postmenopausal Women. Front Physiol. 2019;10:484.https://doi.org/10.3389/fphys.2019.00484
294. Saghebjoo M, Farrokhi-Fard M, Hedayati M, Sadeghi-Tabas S. The effect of high-intensity interval training and L-arginine supplementation on the serum levels of adiponectin and lipid profile in overweight and obese young men. Obesity Medicine. 2019 2019/12/01/;16:100139.https://doi.org/10.1016/j.obmed.2019.100139
295. Shakeri N, Shahram S, Ghazalian F, Elham Y. The Effect of Endurance Training on Adiponectin and Insulin Resistance in Overweight Female University Students. International Medical Journal. 2016 01/01;23:533_6
296. Sixt S, Beer S, Blüher M, Korff N, Peschel T, Sonnabend M, et al. Long- but not short-term multifactorial intervention with focus on exercise training improves coronary endothelial dysfunction in diabetes mellitus type 2 and coronary artery disease. Eur Heart J. 2010 Jan;31(1):112-9.https://doi.org/10.1093/eurheartj/ehp398
297. Sokolovska J, Ostrovska K, Pahirko L, Varblane G, Krilatiha K, Cirulnieks A, et al. Impact of interval walking training managed through smart mobile devices on albuminuria and leptin/adiponectin ratio in patients with type 2 diabetes. Physiol Rep. 2020 Jul;8(13):e14506.https://doi.org/10.14814/phy2.14506
298. Taha M, Elsayed S. Effect of Resistive Training on Serum Adiponectin and Liver Fat in Prediabetic Patients with Fatty Liver. 2013 01/01
299. Tjønna AE, Lee SJ, Rognmo Ø, Stølen TO, Bye A, Haram PM, et al. Aerobic interval training versus continuous moderate exercise as a treatment for the metabolic syndrome: a pilot study. Circulation. 2008 Jul 22;118(4):346-54.https://doi.org/10.1161/circulationaha.108.772822
300. Wang X, You T, Murphy K, Lyles MF, Nicklas BJ. Addition of Exercise Increases Plasma Adiponectin and Release from Adipose Tissue. Med Sci Sports Exerc. 2015 Nov;47(11):2450-5.https://doi.org/10.1249/mss.0000000000000670
301. Wu YT, Hwang CL, Chen CN, Chuang LM. Home-based exercise for middle-aged Chinese at diabetic risk: a randomized controlled trial. Prev Med. 2011 May;52(5):337-43.https://doi.org/10.1016/j.ypmed.2011.02.018
302. Yang D, Yang Y, Li Y, Han R. Physical Exercise as Therapy for Type 2 Diabetes Mellitus: From Mechanism to Orientation. Ann Nutr Metab. 2019;74(4):313-21.https://doi.org/10.1159/000500110
303. Frank LL, Sorensen BE, Yasui Y, Tworoger SS, Schwartz RS, Ulrich CM, et al. Effects of exercise on metabolic risk variables in overweight postmenopausal women: a randomized clinical trial. Obes Res. 2005 Mar;13(3):615-25.https://doi.org/10.1038/oby.2005.66
304. Karacabey K. The effect of exercise on leptin, insulin, cortisol and lipid profiles in obese children. J Int Med Res. 2009 Sep-Oct;37(5):1472-8.https://doi.org/10.1177/147323000903700523

305. Kim SW, Jung WS, Park W, Park HY. Twelve Weeks of Combined Resistance and Aerobic Exercise Improves Cardiometabolic Biomarkers and Enhances Red Blood Cell Hemorheological Function in Obese Older Men: A Randomized Controlled Trial. Int J Environ Res Public Health. 2019 Dec 10;16(24).https://doi.org/10.3390/ijerph16245020
306. Loimaala A, Groundstroem K, Rinne M, Nenonen A, Huhtala H, Parkkari J, et al. Effect of long-term endurance and strength training on metabolic control and arterial elasticity in patients with type 2 diabetes mellitus. Am J Cardiol. 2009 Apr 1;103(7):972-7.https://doi.org/10.1016/j.amjcard.2008.12.026
307. Murakami T, Horigome H, Tanaka K, Nakata Y, Katayama Y, Matsui A. Effects of diet with or without exercise on leptin and anticoagulation proteins levels in obesity. Blood Coagul Fibrinolysis. 2007 Jul;18(5):389-94.https://doi.org/10.1097/01.mbc.0000278929.87251.5d
308. Tan S, Wang J, Cao L, Guo Z, Wang Y. Positive effect of exercise training at maximal fat oxidation intensity on body composition and lipid metabolism in overweight middle-aged women. Clin Physiol Funct Imaging. 2016 May;36(3):225-30.https://doi.org/10.1111/cpf.12217
309. Tan S, Du P, Zhao W, Pang J, Wang J. Exercise Training at Maximal Fat Oxidation Intensity for Older Women with Type 2 Diabetes. Int J Sports Med. 2018 May;39(5):374-81.https://doi.org/10.1055/a-0573-1509
310. Thong FS, Hudson R, Ross R, Janssen I, Graham TE. Plasma leptin in moderately obese men: independent effects of weight loss and aerobic exercise. Am J Physiol Endocrinol Metab. 2000 Aug;279(2):E307-13.https://doi.org/10.1152/ajpendo.2000.279.2.E307
311. Sheikholeslami-Vatani D, Siahkouhian M, Hakimi M, Ali-Mohammadi M. The effect of concurrent training order on hormonal responses and body composition in obese men. Science & Sports. 2015 2015/12/01/;30(6):335-41.https://doi.org/10.1016/j.scispo.2015.06.005
312. Banitalebi E, Kazemi A, Faramarzi M, Nasiri S, Haghighi MM. Effects of sprint interval or combined aerobic and resistance training on myokines in overweight women with type 2 diabetes: A randomized controlled trial. Life Sci. 2019 Jan 15;217:101-9.https://doi.org/10.1016/j.lfs.2018.11.062
313. Borfe L, Brand C, Schneiders LB, Mota J, Cavaglieri CR, Leite N, et al. Effects and Responsiveness of a Multicomponent Intervention on Body Composition, Physical Fitness, and Leptin in Overweight/Obese Adolescents. Int J Environ Res Public Health. 2021 Jul 7;18(14).https://doi.org/10.3390/ijerph18147267
314. Carrillo AE, Flynn MG, Pinkston C, Markofski MM, Jiang Y, Donkin SS, et al. Vitamin D supplementation during exercise training does not alter inflammatory biomarkers in overweight and obese subjects. Eur J Appl Physiol. 2012 Aug;112(8):3045-52.https://doi.org/10.1007/s00421-011-2279-3
315. Fu TC, Wang CH, Lin PS, Hsu CC, Cherng WJ, Huang SC, et al. Aerobic interval training improves oxygen uptake efficiency by enhancing cerebral and muscular hemodynamics in patients with heart failure. Int J Cardiol. 2013 Jul 15;167(1):41-50.https://doi.org/10.1016/j.ijcard.2011.11.086
316. Gallardo-Escribano C, Buonaiuto V, Ruiz-Moreno MI, Vargas-Candela A, Vilches-Perez A, Benitez-Porres J, et al. Epigenetic approach in obesity: DNA methylation in a prepubertal population which underwent a lifestyle modification. Clin Epigenetics. 2020 Sep 23;12(1):144.https://doi.org/10.1186/s13148-020-00935-0
317. Gallistl S, Sudi KM, Aigner R, Borkenstein M. Changes in serum interleukin-6 concentrations in obese children and adolescents during a weight reduction program. Int J Obes Relat Metab Disord. 2001 Nov;25(11):1640-3.https://doi.org/10.1038/sj.ijo.0801808
318. García-Unciti M, Izquierdo M, Idoate F, Gorostiaga E, Grijalba A, Ortega-Delgado F, et al. Weight-loss diet alone or combined with progressive resistance training induces changes in association between the cardiometabolic risk profile and abdominal fat depots. Ann Nutr Metab. 2012;61(4):296-304.https://doi.org/10.1159/000342467
319. Hutnick NA, Williams NI, Kraemer WJ, Orsega-Smith E, Dixon RH, Bleznak AD, et al. Exercise and lymphocyte activation following chemotherapy for breast cancer. Med Sci Sports Exerc. 2005 Nov;37(11):1827-35.https://doi.org/10.1249/01.mss.0000175857.84936.1a
320. Izadpanah A, Barnard RJ, Almeda AJ, Baldwin GC, Bridges SA, Shellman ER, et al. A short-term diet and exercise intervention ameliorates inflammation and markers of metabolic health in overweight/obese children. Am J Physiol Endocrinol Metab. 2012 Aug 15;303(4):E542-50.https://doi.org/10.1152/ajpendo.00190.2012

321. Kobayashi N, Tsuruya Y, Iwasawa T, Ikeda N, Hashimoto S, Yasu T, et al. Exercise training in patients with chronic heart failure improves endothelial function predominantly in the trained extremities. Circ J. 2003 Jun;67(6):505-10.https://doi.org/10.1253/circj.67.505
322. Lira FS, Rosa JC, Dos Santos RV, Venancio DP, Carnier J, Sanches Pde L, et al. Visceral fat decreased by long-term interdisciplinary lifestyle therapy correlated positively with interleukin-6 and tumor necrosis factor-α and negatively with adiponectin levels in obese adolescents. Metabolism. 2011 Mar;60(3):359-65.https://doi.org/10.1016/j.metabol.2010.02.017
323. Moore JB, Benítez-Porres J, Skelton JA, Vargas-Candela A, South AM, Gómez-Huelgas R, et al. Examining the Effect of a 1-yr Lifestyle Intervention on Cardiometabolic and Inflammatory Biomarkers in Youth with Overweight or Obesity: A Pilot Study. Translational Journal of the American College of Sports Medicine. 2021;6(2):e000153.https://doi.org/10.1249/tjx.0000000000000153
324. Nemet D, Oren S, Pantanowitz M, Eliakim A. Effects of a multidisciplinary childhood obesity treatment intervention on adipocytokines, inflammatory and growth mediators. Horm Res Paediatr. 2013;79(6):325-32.https://doi.org/10.1159/000348732
325. Nikseresht M. Comparison of Serum Cytokine Levels in Men Who are Obese or Men Who are Lean: Effects of Nonlinear Periodized Resistance Training and Obesity. J Strength Cond Res. 2018 Jun;32(6):1787-95.https://doi.org/10.1519/jsc.0000000000002039
326. Romeo J, Martinez-Gomez D, Diaz LE, Gómez-Martinez S, Marti A, Martin-Matillas M, et al. Changes in cardiometabolic risk factors, appetite-controlling hormones and cytokines after a treatment program in overweight adolescents: preliminary findings from the EVASYON study. Pediatr Diabetes. 2011 Jun;12(4 Pt 2):372-80.https://doi.org/10.1111/j.1399-5448.2010.00753.x
327. Tenório TRS, Balagopal PB, Andersen LB, Ritti-Dias RM, Hill JO, Lofrano-Prado MC, et al. Effect of Low- Versus High-Intensity Exercise Training on Biomarkers of Inflammation and Endothelial Dysfunction in Adolescents With Obesity: A 6-Month Randomized Exercise Intervention Study. Pediatr Exerc Sci. 2018 Feb 1;30(1):96-105.https://doi.org/10.1123/pes.2017-0067
328. Yoshimura E, Kumahara H, Tobina T, Matsuda T, Watabe K, Matono S, et al. Aerobic exercise attenuates the loss of skeletal muscle during energy restriction in adults with visceral adiposity. Obes Facts. 2014 2014;7(1):26-35.https://doi.org/10.1159/000358576
329. Asjari M, Abedi B, Fatolahi H. Effects of aerobic training and licorice extract consumption on inflammation and antioxidant states in overweight women. Obesity Medicine. 2021 2021/01/01/;21:100271.https://doi.org/10.1016/j.obmed.2020.100271
330. Barrón-Cabrera E, González-Becerra K, Rosales-Chávez G, Mora-Jiménez A, Hernández-Cañaveral I, Martínez-López E. Low-grade chronic inflammation is attenuated by exercise training in obese adults through down-regulation of ASC gene in peripheral blood: a pilot study. Genes Nutr. 2020 Aug 27;15(1):15.https://doi.org/10.1186/s12263-020-00674-0
331. Butts B, Butler J, Dunbar SB, Corwin E, Gary RA. Effects of Exercise on ASC Methylation and IL-1 Cytokines in Heart Failure. Med Sci Sports Exerc. 2018 Sep;50(9):1757-66.https://doi.org/10.1249/mss.0000000000001641
332. Huffman KM, Slentz CA, Bales CW, Houmard JA, Kraus WE. Relationships between adipose tissue and cytokine responses to a randomized controlled exercise training intervention. Metabolism. 2008 Apr;57(4):577-83.https://doi.org/10.1016/j.metabol.2007.11.023
333. Prestes J, da Cunha Nascimento D, Tibana RA, Teixeira TG, Vieira DC, Tajra V, et al. Understanding the individual responsiveness to resistance training periodization. Age (Dordr). 2015 Jun;37(3):9793.https://doi.org/10.1007/s11357-015-9793-x
334. Roth CL, Kratz M, Ralston MM, Reinehr T. Changes in adipose-derived inflammatory cytokines and chemokines after successful lifestyle intervention in obese children. Metabolism. 2011 Apr;60(4):445-52.https://doi.org/10.1016/j.metabol.2010.03.023
335. So WY, Song M, Park YH, Cho BL, Lim JY, Kim SH, et al. Body composition, fitness level, anabolic hormones, and inflammatory cytokines in the elderly: a randomized controlled trial. Aging Clin Exp Res. 2013 May;25(2):167-74.https://doi.org/10.1007/s40520-013-0032-y

336. Tavvafian N, Darabi H, Ahani A, Naghizadeh H, Hajiaghaee R, Rahmati-Ahmadabad S, et al. Effects of glycyrrhizic acid supplementation during nonlinear resistance training on inflammatory markers and muscular damage indices in overweight young men. Obesity Medicine. 2020 2020/03/01/;17:100178.https://doi.org/10.1016/j.obmed.2019.100178
337. Zakavi I, Nayebifar S, Ghasemi E, Valipour A. Anti-inflammatory properties of combined aquatic extract of Ferulago angulata boiss with aerobic exercise on pro-inflammatory indices in obese males. J Res Med Sci. 2020;25:86.https://doi.org/10.4103/jrms.JRMS_475_19
338. Hasson RE, Adam TC, Davis JN, Kelly LA, Ventura EE, Byrd-Williams CE, et al. Randomized controlled trial to improve adiposity, inflammation, and insulin resistance in obese African-American and Latino youth. Obesity (Silver Spring). 2012 Apr;20(4):811-8.https://doi.org/10.1038/oby.2010.343
339. Conroy SM, Courneya KS, Brenner DR, Shaw E, O'Reilly R, Yasui Y, et al. Impact of aerobic exercise on levels of IL-4 and IL-10: results from two randomized intervention trials. Cancer Med. 2016 Sep;5(9):2385-97.https://doi.org/10.1002/cam4.836
340. Eizadi M, Laleh B, Khorshidi D. THE EFFECT OF AEROBIC TRAINING WITH DIFFERENCE DURATIONS ON SERUM IL-10 IN MIDDLE-AGED OBESE FEMALES. Acta Endocrinol (Buchar). 2018 Oct-Dec;14(4):563-9.https://doi.org/10.4183/aeb.2018.563
341. Alizadeh AM, Isanejad A, Sadighi S, Mardani M, Kalaghchi B, Hassan ZM. High-intensity interval training can modulate the systemic inflammation and HSP70 in the breast cancer: a randomized control trial. J Cancer Res Clin Oncol. 2019 Oct;145(10):2583-93.https://doi.org/10.1007/s00432-019-02996-y
342. Nikseresht M, Hafezi Ahmadi MR, Hedayati M. Detraining-induced alterations in adipokines and cardiometabolic risk factors after nonlinear periodized resistance and aerobic interval training in obese men. Appl Physiol Nutr Metab. 2016 Oct;41(10):1018-25.https://doi.org/10.1139/apnm-2015-0693
