# Supplementary material for: Exercise-induced changes in systemic inflammatory biomarkers in overweight and obese populations: a bibliometric analysis and umbrella review of meta-analyses
Source: Front Immunol. 2026 May 20;17:1838118. doi: 10.3389/fimmu.2026.1838118 (PMC13230183; doi:10.3389/fimmu.2026.1838118)
Supplement: Supplementary file 3 [file SupplementaryFile3.pdf]

Supplementary File 3

Table Characteristics of Original Studies from Included Meta-Analyses

Included study (first author, year)	Original study (first author, year)	Athlete type	Age (years)	Sex & sample size	outcome	Quality Assessment Scale (Evidence)
Zalagkitis 2025 (1)	Masquio, 2023	EG: obese adolescents (non-athletes)<br>CG: NA	EG: 15.7±1.2; age range=15–19<br>CG: NA	EG: n=29 (M/F NR) final analysis n=22<br>CG: NA	CRP, IL-6, TNF-α	RoB (Zalagkitis 2025, Table S3): Some concerns [+/+/?/+/+/+]
	Pena, 2023	EG: obese adolescents (non-athletes)<br>CG: obese adolescents (non-athletes)	EG: mean age=NR; age range=12–16<br>CG: mean age=NR; age range=12–16	EG: n=40 (M/F NR)<br>CG: n=24 (M/F NR)	IL-6, TNF-α	RoB (Zalagkitis 2025, Table S3): Low [+/+/+/+/+/+]
	Cobos-Palacios, 2022	EG: obese children (non-athletes)<br>CG: obese children (non-athletes)	EG: NR<br>CG: NR<br>Total: 7.8±1.4	EG: NR<br>CG: NR<br>Total: n=144 (M=75, F=69)	CRP, IL-6, TNF-α	RoB (Zalagkitis 2025, Table S3): Some concerns [N/+/?/?/+/+]
	Nambi, 2022	EG: obese boys (non-athletes)<br>CG: obese boys (non-athletes)	EG: 10.12±1.2<br>CG: 10.56±1.4	EG: n=38 (M=38, F=0)<br>CG: n=38 (M=38, F=0)	IL-6, TNF-α	RoB (Zalagkitis 2025, Table S3): Low [+/+/+/+/+/+]
	Borfe, 2021	EG: overweight adolescents (non-athletes)<br>CG: overweight adolescents (non-athletes)	EG: 13.00±1.06<br>CG: 13.13±1.41	EG: n=17 (M=8, F=9)<br>CG: n=16 (M=8, F=8)	IL-6, TNF-α	RoB (Zalagkitis 2025, Table S3): Some concerns [+/+/?/N/+/+]
	Moore, 2021	EG: overweight/obese children (non-athletes)<br>CG: NA	EG: 8.7±1.1<br>CG: NA	EG: n=50 (M=26, F=24)<br>CG: NA	IL-6, TNF-α	RoB (Zalagkitis 2025, Table S3): Some concerns [N/+/?/N/+/+]
	Skelly, 2021	EG: overweight/obese youth (BMI >85th–97th percentile); higher dairy intake + mixed-exercise (12 weeks)<br>CG: overweight/obese youth (BMI >85th–97th percentile); low dairy intake + mixed-exercise (12 weeks)	EG: mean age=14.6±2.3; age range=10–18<br>CG: mean age=14.9±2.3; age range=10–18	EG: n=23 (M/F NR)<br>CG: n=23 (M/F NR)	TNF-α	RoB (Zalagkitis 2025, Table S3): Low [+/+/+/+/+/+]
	Cordellat, 2020	EG: obese children/adolescents (non-athletes)<br>CG: obese children/adolescents (non-athletes)	EG: NR<br>CG: NR<br>Total: 10.8±1.6	EG: NR<br>CG: NR<br>Total: n=22 (M=9, F=13)	CRP, TNF-α	RoB (Zalagkitis 2025, Table S3): Some concerns [N/+/?/N/+/+]
	Gallardo-Escribano, 2020	EG: obese children (non-athletes)<br>CG: NR	EG: mean age=NR; age range=4–9<br>CG: NR	EG: n=131 (M=70, F=61)<br>CG: NR	IL-6, TNF-α	RoB (Zalagkitis 2025, Table S3): Some concerns [+/+/?/N/+/+]
	Mayerhofer, 2020	EG: overweight/obese children (non-athletes)<br>CG: NA	EG: mean age=12±1; age range=10–14<br>CG: NA	EG: n=236 (M/F NR) final analysis n=195 (M=93, F=102)<br>CG: NA	CRP	RoB (Zalagkitis 2025, Table S3): Some concerns [N/+/?/+/+/+]
	Plavsic, 2020	EG: obese female adolescents (non-athletes); diet+HIIT<br>CG: obese female adolescents (non-athletes); diet	EG: 15.8±1.6<br>CG: 15.8±1.6	EG: n=22 (M=0, F=22)<br>CG: n=22 (M=0, F=22)	hsCRP	RoB (Zalagkitis 2025, Table S3): Low [+/+/+/+/+/+]
	Yin, 2020	EG: obese children/adolescents (non-athletes)<br>CG: lean children/adolescents (non-athletes)	EG: mean age=10.52±2.33; age range=7–14<br>CG: mean age=9.71±1.81; age range=7–14	EG: n=120 (M/F NR)<br>CG: n=85 (M/F NR)	CRP	RoB (Zalagkitis 2025, Table S3): Some concerns [+/?/?/+/+/+]
	Farpour-Lambert, 2019	EG: obese children (BMI >97th percentile); moderate (n=21) + high (n=31) intensity lifestyle program (12 months)	EG: mean age=NR; age range=7.5–11.9<br>CG: mean age=NR;	EG: n=52 (M/F NR)<br>CG: n=22 (M/F NR)	hs-CRP	RoB (Zalagkitis 2025, Table S3): Low [+/+/+/+/+/+]

Included study (first author, year)	Original study (first author, year)	Athlete type	Age (years)	Sex & sample size	outcome	Quality Assessment Scale (Evidence)
		CG: obese children (BMI >97th percentile); usual care (n=22)	age range=7.5–11.9			
	Montero, 2019	EG: severe obese adolescents (non-athletes)<br>CG: normal-weight adolescents (non-athletes)	EG: NR<br>CG: NR	EG: n=27 (M/F NR)<br>CG: n=20 (M/F NR)	CRP, CRP	RoB (Zalagkitis 2025, Table S3): High [-/+/?/N/+/ ?]
	Roche, 2019	EG: severe obese adolescents (non-athletes)<br>CG: NA	EG: 14.6±1.2<br>CG: NA	EG: n=32 (M=16, F=16) final analysis n=23 (M=10, F=13)<br>CG: NA	CRP	RoB (Zalagkitis 2025, Table S3): Some concerns [+/+/?/+/+/+]
	Liu, 2018	EG: obese female adolescents (non-athletes)<br>CG: obese female adolescents (non-athletes)	EG: mean age=14.7±0.8; age range=14–16<br>CG: mean age=14.7±0.8; age range=14–16	EG: n=30 (M=0, F=30)<br>CG: n=20 (M=0, F=20)	CRP, IL-6, TNF-α	RoB (Zalagkitis 2025, Table S3): Low [+/+/+/+/+/+]
	Santiprabhob, 2018	EG: obese youths (non-athletes)<br>CG: NA	EG: mean age=NR; age range=8–18<br>CG: NA	EG: n=126 (M/F NR) completed n=115 (M=60, F=55)<br>CG: NA	hsCRP, IL-6	RoB (Zalagkitis 2025, Table S3): Some concerns [+/+/?/N/+/+]
	Tenório, 2018	EG: obese adolescents (non-athletes); HIT<br>CG: obese adolescents (non-athletes); LIT	EG: mean age=15±1.4; age range=13–18<br>CG: mean age=15±1.4; age range=13–18	EG: n=31 (M/F NR)<br>CG: n=31 (M/F NR)<br>Total: n=62 (M=28, F=34)	IL-6, TNF-α	RoB (Zalagkitis 2025, Table S3): Low [+/+/+/+/+/+]
	Moraes, 2016	EG: children/adolescents (non-athletes)<br>CG: children/adolescents (non-athletes)	EG: mean age=10.2±2.2; age range=8–16<br>CG: mean age=11.3±1.6; age range=8–16	EG: n=19 (M=9, F=10)<br>CG: n=17 (M=6, F=11)	hsCRP	RoB (Zalagkitis 2025, Table S3): High [-/+/?/N/+/+]
	Nunes, 2016	EG: obese adolescents (non-athletes)<br>CG: obese adolescents (non-athletes)	EG: 16.18±1.51<br>CG: 15.4±1.20	EG: n=17 (M=8, F=9)<br>CG: n=8 (M=4, F=4)	CRP	RoB (Zalagkitis 2025, Table S3): Some concerns [+/?/?/+/+/+]
	Rainone, 2016	EG: obese adolescents (non-athletes)<br>CG: normal-weight adolescents (non-athletes)	EG: mean age=NR; median age=13.6; age range=NR<br>CG: mean age=NR; median age=14.1; age range=NR	EG: n=22 (M/F NR)<br>CG: n=18 (M/F NR)	IL-6, TNF-α	RoB (Zalagkitis 2025, Table S3): High [−/+/?/N/N/+]
	Liu, 2015	EG: adolescents (non-athletes)<br>CG: adolescents (non-athletes)	EG: mean age=NR; age range=Male 11–15; Female 9–13<br>CG: mean age=NR; age range=Male 11–15; Female 9–13	EG: n=25 (M/F NR)<br>CG: n=29 (M/F NR)	CRP, IL-6, TNF-α	RoB (Zalagkitis 2025, Table S3): Low [+/+/+/+/+/+]
	Abu-Kishk, 2014	EG: obese adolescents (non-athletes)<br>CG: obese adolescents (non-athletes)	EG: mean age=15.17±1.74; age range=12–18<br>CG: mean age=16.0±1.52; age range=12–18	EG: n=36 (M=22, F=14) final analysis n=21<br>CG: n=8 (M=6, F=2)	CRP	RoB (Zalagkitis 2025, Table S3): Some concerns [+/?/+/?/+/+]
	Bocca, 2014	EG: obese children (non-athletes)<br>CG: obese children (non-athletes)	EG: 4.6±0.8<br>CG: 4.7±0.8	EG: n=40 (M=12, F=28)<br>CG: n=35 (M=9, F=26)	hsCRP, IL-6, TNF-α	RoB (Zalagkitis 2025, Table S3): Low [+/+/+/+/+/+]
	Gong, 2014	EG: children (non-athletes)	EG: 8.8±1.3	EG: n=160 (M=102,	CRP, IL-6	RoB (Zalagkitis 2025, Table S3): Low [+/+/+/+/+/+]

Included study (first author, year)	Original study (first author, year)	Athlete type	Age (years)	Sex & sample size	outcome	Quality Assessment Scale (Evidence)
		CG: children (non-athletes)	CG: 8.9±1.3	F=58)<br>CG: n=166 (M=110, F=56)		
	Tang, 2014	EG: obese children/adolescents (non-athletes)<br>CG: NA	EG: mean age=NR; age range=7–17<br>CG: NA	EG: n=20 (M=11, F=9)<br>CG: NA	CRP	RoB (Zalagkitis 2025, Table S3): Some concerns [+/+/?/N/+/+]
	Nemet, 2013	EG: children with obesity (non-athletes)<br>CG: children with obesity (non-athletes)	EG: mean age=10.41±1.96; age range=6–13<br>CG: mean age=10.49±2.67; age range=6–13	EG: n=21 (M/F NR)<br>CG: n=20 (M/F NR)	IL-6	RoB (Zalagkitis 2025, Table S3): Low [+/+/+/+/+/+]
	Nisanci Kilins, 2013	EG: overweight children/adolescents (non-athletes)<br>CG: NA	EG: mean age=12.52±2.85; age range=9–17<br>CG: NA	EG: n=19 (M=11, F=8)<br>CG: NA	CRP	RoB (Zalagkitis 2025, Table S3): Some concerns [+/+/?/N/+/+]
	Roberts, 2013	EG: obese group (BMI >85th percentile; 13/19 >95th percentile)<br>CG: normal-weight group	EG: 13.1±0.5<br>CG: 11.2±0.5	EG: n=19 (M=9, F=10)<br>CG: n=14 (M=6, F=8)	IL-6, IL-8, TNF-α, IL-10	RoB (Zalagkitis 2025, Table S3): High [−/+/?/N/+/+]
	da Silva, 2012	EG: obese adolescents (non-athletes)<br>CG: NR	EG: 17±1.4<br>CG: NR	EG: n=50 (M/F NR)<br>CG: NR	CRP	RoB (Zalagkitis 2025, Table S3): Some concerns [N/+/?/N/N/+]
	Hasson, 2012	EG: obese adolescents (non-athletes) (two exercise groups)<br>CG: obese adolescents (non-athletes)	EG: 15.4±1.1 (Total)<br>CG: 15.4±1.1 (Total)	EG: n=39 (M/F NR); n=31 (M/F NR)<br>CG: n=30 (M/F NR)	TNF-α	RoB (Zalagkitis 2025, Table S3): Low [+/+/+/+/+/+]
	Izadpanah, 2012	EG: overweight/obese children/adolescents (non-athletes)<br>CG: NR	EG: mean age=13.0±0.5; age range=8–17<br>CG: NR	EG: n=21 (M=9, F=12)<br>CG: NR	IL-6, TNF-α	RoB (Zalagkitis 2025, Table S3): Some concerns [N/+/?/N/N/N]
	Santomauro, 2012	EG: obese children/adolescents with NAFLD (non-athletes)<br>CG: obese children/adolescents without NAFLD (non-athletes)	EG: mean age=NR; age range=7–18<br>CG: mean age=NR; age range=7–18	EG: n=24 (M/F NR)<br>CG: n=12 (M/F NR)	CRP	RoB (Zalagkitis 2025, Table S3): Some concerns [?/+/?/N/+/+]
	Rynders, 2012	EG: diet and exercise group (BMI >95th percentile)<br>CG: diet and exercise + metformin group (BMI >95th percentile)	EG: 14.3±2.4 (Total)<br>CG: 14.3±2.4 (Total)	EG: n=NR (M/F NR)<br>CG: n=NR (M/F NR)<br>Total: n=37 (M/F NR)<br>final analysis n=16 (M/F NR); not complete n=15	hs-CRP, IL-6	RoB (Zalagkitis 2025, Table S3): Some concerns [+/+/?/+/+/+]
	Garanty-Bogacka, 2011	EG: obese children/adolescents (non-athletes)<br>CG: NR	EG: mean age=14.2±2.6; age range=8–18<br>CG: NR	EG: n=50 (M=21, F=29)<br>CG: NR	CRP, IL-6	RoB (Zalagkitis 2025, Table S3): Some concerns [N/+/?/N/+/N]
	Lira, 2011	EG: obese adolescents (non-athletes)<br>CG: NR	EG: mean age=16.6±1.67; age range=15–19<br>CG: NR	EG: n=18 (M=7, F=11)<br>CG: NR	IL-6, TNF-α	RoB (Zalagkitis 2025, Table S3): Some concerns [+/+/?/N/+/+]
	Romeo, 2011	EG: obese adolescents (non-athletes)<br>CG: NA	EG: mean age=NR; age range=13–16<br>CG: NA	EG: n=31 (M/F NR)<br>measured n=25 (M=13, F=12)<br>CG: NA	IL-6, TNF-α	RoB (Zalagkitis 2025, Table S3): Some concerns [N/+/?/+/+/+]
	Roth, 2011	EG: children with obesity (non-athletes); weight reduction<br>CG: children with obesity (non-athletes); no weight	EG: 10.7±0.29<br>CG: 10.7±0.29	EG: n=29 (M/F NR)<br>CG: n=33 (M/F NR)	TNF-α	RoB (Zalagkitis 2025, Table S3): Some concerns [+/+/?/N/+/+]

Included study (first author, year)	Original study (first author, year)	Athlete type	Age (years)	Sex & sample size	outcome	Quality Assessment Scale (Evidence)
		reduction				
	Vos, 2011	EG: obese adolescents (non-athletes)<br>CG: obese adolescents (non-athletes)	EG: 13.3±2.0<br>CG: 13.1±1.9	EG: n=40 (M=18, F=22)<br>CG: n=39 (M=19, F=20)	hsCRP	RoB (Zalagkitis 2025, Table S3): Low [+/+/+/+/+/+]
	Wang, 2011	EG: obese male adolescents (non-athletes)<br>CG: NA	EG: mean age=13.3±2.5; age range=10–19<br>CG: NA	EG: n=43 (M=43, F=0)<br>CG: NA	CRP, IL-6, TNF-α	RoB (Zalagkitis 2025, Table S3): Some concerns [+/+/?/N/+/+]
	Ben Ounis, 2010	EG: obese adolescents (non-athletes)<br>CG: obese adolescents (non-athletes)	EG: 13.1±0.8<br>CG: 13.3±0.6	EG: n=14 (M=7, F=7)<br>CG: n=14 (M=7, F=7)	CRP, IL-6, TNF-α	RoB (Zalagkitis 2025, Table S3): Low [+/+/+/+/+/+]
	Ben Ounis, 2009	EG: obese adolescents (non-athletes); female only (diet / training / diet+training arms)<br>CG: NR	EG: 13.2±0.3; 13.1±0.9; 13.1±0.8<br>CG: NR	EG: total n=27 (M=0, F=27) (group n NR)<br>CG: NR	IL-6, TNF-α	RoB (Zalagkitis 2025, Table S3): Low [+/+/+/+/+/+]
	Shalitin, 2009	EG: obese children (non-athletes) (exercise / diet / diet+exercise arms)<br>CG: NR	EG: 8.21±1.78; 8.51±1.52; 8.2±1.56<br>CG: NR	EG: n=52 (M/F NR); n=55 (M/F NR); n=55 (M/F NR)<br>CG: NR	CRP, IL-6	RoB (Zalagkitis 2025, Table S3): Low [+/+/+/+/+/+]
	Kelishadi, 2008	EG: obese children/adolescents (non-athletes)<br>CG: NR	EG: mean age=NR; age range=12–18<br>CG: NR	EG: n=35 (M=19, F=16)<br>CG: NR	CRP	RoB (Zalagkitis 2025, Table S3): Some concerns [N/+/?/N/+/+]
	Roberts, 2007	EG: overweight children/adolescents (non-athletes)<br>CG: NA	EG: mean age=13±0.5; age range=8–17<br>CG: NA	EG: n=19 (M/F NR)<br>CG: NA	CRP	RoB (Zalagkitis 2025, Table S3): Some concerns [+/+/?/N/+/+]
	Balagopal-1, 2005	EG: obese adolescents (baseline obese n=15; lean baseline-only n=6 (M=3, F=3))<br>CG: obese adolescents	EG: 15.6±0.3<br>CG: 15.9±0.5	EG: n=8 (M=4, F=4)<br>CG: n=7 (M=4, F=3)	CRP, IL-6	RoB (Zalagkitis 2025, Table S3): Low [+/+/+/+/+/+]
	Balagopal-2, 2005	EG: obese adolescents (baseline obese n=15; lean baseline-only n=6 (M=3, F=3))<br>CG: obese adolescents	EG: 15.6±0.3<br>CG: 15.9±0.5	EG: n=8 (M=4, F=4)<br>CG: n=7 (M=4, F=3)	CRP, IL-6	RoB (Zalagkitis 2025, Table S3): Some concerns [+/+/+/ ?/+/+]
	Reinehr, 2005	EG: children with obesity (non-athletes)<br>CG: nonobese children (non-athletes)	EG: mean age=11; age range=9–13<br>CG: mean age=11; age range=10–13	EG: n=31 (M/F NR)<br>CG: n=14 (M/F NR)	hsCRP, TNF-α	RoB (Zalagkitis 2025, Table S3): Some concerns [?/+/?/N/+/+]
	Gallistl, 2001	EG: children with obesity (non-athletes)<br>CG: lean children (non-athletes)	EG: 11.9±1.8 (F); 11.6±1.7 (M)<br>CG: 11.7±1.6	EG: n=49 (M=18, F=31)<br>CG: n=69 (M=28, F=41)	IL-6	RoB (Zalagkitis 2025, Table S3): High [-/+/?/N/+/+]
Wang 2025 (2)	Bhati 2023	IG: Patients with T2DM and cardiac autonomic neuropathy (CAN); progressive resistance training<br>CG: Patients with T2DM and CAN; usual care	IG: 52.8±6.82<br>CG: 54.0±8.18	IG: n=28 (M=15, F=13)<br>CG: n=28 (M=17, F=11)	IL-6, IL-18, hsCRP	Cochrane RoB (Source NR, RoB figure): Some concerns [? / ? / ? / ? / ? / ?]
	Sabouri 2021	IG: Adults with T2DM; HIIT<br>CG: Adults with T2DM; control	IG: 52.02±4.59<br>CG: 52.28±3.16	IG: n=16 (M=9, F=7)<br>CG: n=13 (M=6, F=7)	TNF-α, CRP, IL-6	Cochrane RoB (Source NR, RoB figure): Some concerns [+ / ? / ? / + / + / ?]
		IG: Adults with T2DM; strength training (ST)<br>CG: Adults with T2DM; control	IG: 51.31±4.47<br>CG: 52.28±3.16	IG: n=15 (M=7, F=8)<br>CG: n=13 (M=6, F=7)	TNF-α, CRP, IL-6	Cochrane RoB (Source NR, RoB figure): Some concerns [+ / ? / ? / + / + / ?]
		IG: Adults with T2DM; HIIT+ST<br>CG: Adults with T2DM; control	IG: 52.53±4.80<br>CG: 52.28±3.16	IG: n=15 (M=10, F=5)<br>CG: n=13 (M=6, F=7)	TNF-α, CRP, IL-6	Cochrane RoB (Source NR, RoB figure): Some concerns [+ / ? / ? / + / + / ?]
	Nadi 2019	IG: Women with diabetic neuropathy; low-intensity resistance training<br>CG: Women with diabetic neuropathy; control (daily activities)	IG: 56.13±3.39<br>CG: 54.80±3.29	IG: n=15 (F=15)<br>CG: n=15 (F=15)	CRP, TNF-α, IL-10	Cochrane RoB (Source NR, RoB figure): High [? / - / ? / + / + / -]
		IG: Women with diabetic neuropathy; peripheral neuropathy exercises (EPN)	IG: 55.46±2.47<br>CG: 54.80±3.29	IG: n=15 (F=15)<br>CG: n=15 (F=15)	CRP, TNF-α, IL-10	Cochrane RoB (Source NR, RoB figure): High [? / - / ? / + / + / -]

Included study (first author, year)	Original study (first author, year)	Athlete type	Age (years)	Sex & sample size	outcome	Quality Assessment Scale (Evidence)
		CG: Women with diabetic neuropathy; control (daily activities)				
	Rech 2019	IG: Elderly patients with T2DM; resistance training<br>CG: Elderly patients with T2DM; active control (stretching)	IG: 70.5±7.4<br>CG: 68±6.5	IG: n=17 (M=10, F=7)<br>CG: n=21 (M=10, F=11)	TNF-α, IL-6, IL-1β, IL-10, CRP	Cochrane RoB (Source NR, RoB figure): Some concerns [+ / ? / ? / + / + / ?]
	Kadoglou-1 2012	IG: Overweight/obese patients with T2DM; supervised resistance training (REG)<br>CG: Overweight/obese patients with T2DM; exercise counseling/usual care	IG: 61.5±5.4<br>CG: 64.6±4.3	IG: n=23 (M=7, F=16)<br>CG: n=24 (M=5, F=19)	hsCRP	Cochrane RoB (Source NR, RoB figure): Some concerns [+ / + / ? / + / ? / ?]
	Kadoglou-2 2012	IG: Overweight/obese patients with T2DM; supervised aerobic exercise<br>CG: Overweight/obese patients with T2DM; control (physical activity advice)	IG: 58.3±5.4<br>CG: 57.9±7.2	IG: n=21 (M=6, F=15)<br>CG: n=24 (M=7, F=17)	hsCRP	Cochrane RoB (Source NR, RoB figure): Some concerns [+ / + / ? / + / + / ?]
		IG: Overweight/obese patients with T2DM; supervised resistance training<br>CG: Overweight/obese patients with T2DM; control (physical activity advice)	IG: 56.1±5.3<br>CG: 57.9±7.2	IG: n=23 (M=7, F=16)<br>CG: n=24 (M=7, F=17)	hsCRP	Cochrane RoB (Source NR, RoB figure): Some concerns [+ / + / ? / + / + / ?]
		IG: Overweight/obese patients with T2DM; aerobic exercise + resistance training<br>CG: Overweight/obese patients with T2DM; control (physical activity advice)	IG: 57.9±6.5<br>CG: 57.9±7.2	IG: n=22 (M=5, F=17)<br>CG: n=24 (M=7, F=17)	hsCRP	Cochrane RoB (Source NR, RoB figure): Some concerns [+ / + / ? / + / + / ?]
	Swift 2012	IG: Adults with T2DM; aerobic exercise training<br>CG: Adults with T2DM; control	IG: 55.8±7.9<br>CG: 58.5±8.6	IG: n=50 (M=20, F=30)<br>CG: n=37 (M=11, F=26)	CRP	Cochrane RoB (Source NR, RoB figure): Low [+ / + / + / + / + / +]
		IG: Adults with T2DM; resistance exercise training<br>CG: Adults with T2DM; control	IG: 58.7±8.0<br>CG: 58.5±8.6	IG: n=58 (M=26, F=32)<br>CG: n=37 (M=11, F=26)	CRP	Cochrane RoB (Source NR, RoB figure): Low [+ / + / + / + / + / +]
		IG: Adults with T2DM; combination (aerobic + resistance) training<br>CG: Adults with T2DM; control	IG: 56.7±7.8<br>CG: 58.5±8.6	IG: n=59 (M=23, F=36)<br>CG: n=37 (M=11, F=26)	CRP	Cochrane RoB (Source NR, RoB figure): Low [+ / + / + / + / + / +]
	Jorge 2011	IG: Adults with T2DM; aerobic training<br>CG: Adults with T2DM; control (stretching)	IG: 52.09±8.71<br>CG: 53.41±9.82	IG: n=12 (M=5, F=7)<br>CG: n=12 (M=4, F=8)	hs-CRP, TNF-α, IL-6	Cochrane RoB (Source NR, RoB figure): Some concerns [? / ? / ? / + / ? / ?]
		IG: Adults with T2DM; resistance training<br>CG: Adults with T2DM; control (stretching)	IG: 54.10±8.94<br>CG: 53.41±9.82	IG: n=12 (M=5, F=7)<br>CG: n=12 (M=4, F=8)	hs-CRP, TNF-α, IL-6	Cochrane RoB (Source NR, RoB figure): Some concerns [? / ? / ? / + / ? / ?]
		IG: Adults with T2DM; combined training (aerobic + resistance)<br>CG: Adults with T2DM; control (stretching)	IG: 57.90±8.06<br>CG: 53.41±9.82	IG: n=12 (M=4, F=8)<br>CG: n=12 (M=4, F=8)	hs-CRP, TNF-α, IL-6	Cochrane RoB (Source NR, RoB figure): Some concerns [? / ? / ? / + / ? / ?]
	Plotnikoff 2010	IG: Obese adults with T2DM; home-based progressive RT<br>CG: Obese adults with T2DM; usual care/no training	IG: 55±12<br>CG: 54±12	IG: n=27 (M=8, F=19)<br>CG: n=21 (M=8, F=13)	CRP	Cochrane RoB (Source NR, RoB figure): Some concerns [+ / ? / ? / + / + / ?]
	Wycherley 2010	IG: Overweight/obese patients with T2DM; energy-restricted standard carbohydrate diet + RT<br>CG: Overweight/obese patients with T2DM; energy-restricted standard carbohydrate diet	56.1±7.5	IG: n=17 (M/F NR)<br>CG: n=16 (M/F NR)	CRP	Cochrane RoB (Source NR, RoB figure): Some concerns [+ / ? / ? / + / + / ?]
		IG: Overweight/obese patients with T2DM; energy-restricted higher-protein diet + RT<br>CG: Overweight/obese patients with T2DM; energy-restricted higher-protein diet	56.1±7.5	IG: n=14 (M/F NR)<br>CG: n=12 (M/F NR)	CRP	Cochrane RoB (Source NR, RoB figure): Some concerns [+ / ? / ? / + / + / ?]
	Brooks 2007	IG: Hispanic older adults with T2DM; strength training + standard care<br>CG: Hispanic older adults with T2DM; standard care	IG: 66±2<br>CG: 66±1	IG: n=31 (M=21, F=10)<br>CG: n=31 (M=19, F=12)	CRP, Adiponectin	Cochrane RoB (Source NR, RoB figure): Some concerns [? / + / ? / + / ? / ?]
Hernandez-Martinez 2025 (3)	Mendes, 2024	EG: Adults with sarcopenic obesity (women 85%, men 15%)<br>CG: Adults with sarcopenic obesity (women 85%, men	EG: 44.08±13.32<br>CG: 50.4±11.1	EG: n=12 (M=NR, F=NR; women 85%, men 15%)	Leptin, IL-6, CRP	RoB2 (Hernandez-Martinez 2025, RoB domains figure/Section 3.2): High [+/−/+/+/+]<br>TESTEX (Hernandez-Martinez 2025, Table 2): 12/15

Included study (first author, year)	Original study (first author, year)	Athlete type	Age (years)	Sex & sample size	outcome	Quality Assessment Scale (Evidence)
		15%)		CG: n=10 (M=NR, F=NR; women 85%, men 15%)		
	Dieli-Conwright, 2018	EG: Adults with sarcopenic obesity (women 100%)<br>CG: Adults with sarcopenic obesity (women 100%)	EG: 52.8±10.6<br>CG: 53.6±10.1	EG: n=50 (M=0, F=50)<br>CG: n=50 (M=0, F=50)	Leptin, IL-6, CRP	RoB2 (Hernandez-Martinez 2025, RoB domains figure/Section 3.2): Low [+/+/+/+/+]<br>TESTEX (Hernandez-Martinez 2025, Table 2): 12/15
	Park, 2017	EG: Adults with sarcopenic obesity (women 100%)<br>CG: Adults with sarcopenic obesity (women 100%)	EG: 73.5±7.1<br>CG: 74.7±5.1	EG: n=25 (M=0, F=25)<br>CG: n=25 (M=0, F=25)	Leptin, IL-6, CRP	RoB2 (Hernandez-Martinez 2025, RoB domains figure/Section 3.2): Some concerns [+/ ?/ +/ +/ +]<br>TESTEX (Hernandez-Martinez 2025, Table 2): 12/15
	Kim, 2016	EG: Adults with sarcopenic obesity (women 100%)<br>CG: Adults with sarcopenic obesity (women 100%; non-exercise comparator arm NR)	EG: 81.4±4.3<br>CG: NR (two non-exercise arms reported: 81.2±4.9 and 81.1±5.1; mapping to "a/b" NR)	EG: n=35 (M=0, F=35)<br>CG: n=34 (M=0, F=34)	Leptin, IL-6, CRP	RoB2 (Hernandez-Martinez 2025, RoB domains figure/Section 3.2): High [?/−/+/ ?/ ?]<br>TESTEX (Hernandez-Martinez 2025, Table 2): 11/15
		EG: Adults with sarcopenic obesity (women 100%)<br>CG: Adults with sarcopenic obesity (women 100%; non-exercise comparator arm NR)	EG: 81.4±4.3<br>CG: NR (two non-exercise arms reported: 81.2±4.9 and 81.1±5.1; mapping to "a/b" NR)	EG: n=35 (M=0, F=35)<br>CG: n=34 (M=0, F=34)	Leptin, IL-6, CRP	RoB2 (Hernandez-Martinez 2025, RoB domains figure/Section 3.2): High [?/−/+/ ?/ ?]<br>TESTEX (Hernandez-Martinez 2025, Table 2): 11/15
Tan 2025 (4)	Lin, 2023	EG: Obese, BCSs (stage I–III)<br>CG: Obese, BCSs (stage I–III)	EG: 59.2±8.2<br>CG: 59.0±8.6	EG: n=84 (M=NR, F=NR)<br>CG: n=88 (M=NR, F=NR)	Leptin, Adiponectin	RoB (Tan 2025, Fig. 3): High [?/+/-/+/?/?/?]
	Martins, 2023	EG: Obese or overweight, PW, BCSs (stage I–III)<br>CG: Obese or overweight, PW, BCSs (stage I–III)	EG: 52.1±10.1<br>CG: 57.7±8.8	EG: n=11 (M=0, F=11)<br>CG: n=11 (M=0, F=11)	TNF-α, IL-6, IL-10	RoB (Tan 2025, Fig. 3): High [+/+/-/-/?/?/?]
	Sturgeon, 2023	EG: Obese, BCSs (post-operative)<br>CG: Obese, BCSs (post-operative)	EG: 59.2±8.2<br>CG: 58.7±8.4	EG: n=80 (M=NR, F=NR)<br>CG: n=81 (M=NR, F=NR)	CRP	RoB (Tan 2025, Fig. 3): High [+/+/-/+/?/?/?]
	Alizadeh, 2019	EG: Obese or overweight, PW, BCSs (stage I–III)<br>CG: Obese or overweight, PW, BCSs (stage I–III)	EG: 49.2±9.7<br>CG: 48.42±7.54	EG: n=24 (M=0, F=24)<br>CG: n=24 (M=0, F=24)	IL-6, IL-10	RoB (Tan 2025, Fig. 3): High [+/+/-/+/+/+/+]
	Ligibel, 2019	EG: Obese or overweight, PW, BCSs<br>CG: Obese or overweight, PW, BCSs	EG: 52.3±9.6<br>CG: 53.1±7.9	EG: n=26 (M=0, F=26)<br>CG: n=22 (M=0, F=22)	CRP, IL-6, Leptin, Adiponectin	RoB (Tan 2025, Fig. 3): High [?/?/-/?/?/+/+]
	Dieli-Conwright-1 2018	EG: Obese or overweight, PW, BCSs (stage I–III)<br>CG: Obese or overweight, PW, BCSs (stage I–III)	EG: 53.0±10.0<br>CG: 55.0±4.5	EG: n=10 (M=0, F=10)<br>CG: n=10 (M=0, F=10)	CRP, IL-6, Leptin, Adiponectin	RoB (Tan 2025, Fig. 3): High [+/+/-/+/+/+/+]
	Dieli-Conwright-2 2018	EG: Obese or overweight, PW, BCSs (stage 0–III)<br>CG: Obese or overweight, PW, BCSs (stage 0–III)	EG: 52.8±10.6<br>CG: 53.6±10.1	EG: n=46 (M=0, F=46)<br>CG: n=45 (M=0, F=45)	TNF-α, IL-6, Leptin, Adiponectin	RoB (Tan 2025, Fig. 3): High [+/+/-/+/+/+/+]
	Winters-Stone, 2018	EG: Obese or overweight, PW, BCSs (stage 0–IIIC)<br>CG: Obese or overweight, PW, BCSs (stage 0–IIIC)	EG: 59.8±11.4<br>CG: 59.3±11.6	EG: n=109 (M=0, F=109)<br>CG: n=106 (M=0, F=106)	CRP, TNF-α, IL-6, Leptin, Adiponectin	RoB (Tan 2025, Fig. 3): High [+/+/-/+/+/+/+]
	Kim, 2017	EG: Obese or overweight, PW, BCSs (stage I–IIIA)<br>CG: Obese or overweight, PW, BCSs (stage I–IIIA)	EG: 56±6.5<br>CG: 49.3±4.8	EG: n=11 (M=0, F=11)<br>CG: n=13 (M=0, F=13)	Leptin, Adiponectin	RoB (Tan 2025, Fig. 3): High [+/+/-/+/+/+/+]
	Hagstrom, 2016	EG: Obese or overweight, PW, BCSs (stage I–IIIA)<br>CG: Obese or overweight, PW, BCSs (stage I–IIIA)	EG: 51.2±8.5<br>CG: 52.7±9.4	EG: n=19 (M=0, F=19)<br>CG: n=15 (M=0, F=15)	CRP, TNF-α, IL-6, IL-10	RoB (Tan 2025, Fig. 3): High [+/+/-/+/-/+/+]
	Rogers, 2014	EG: Obese, BCSs (stage I–II)<br>CG: Obese, BCSs (stage I–II)	EG: 55.2±9.1<br>CG: 57.2±5.5	EG: n=20 (M=NR, F=NR)<br>CG: n=22 (M=NR,	TNF-α, IL-6, IL-10	RoB (Tan 2025, Fig. 3): High [+/+/-/+/+/+/+]

Included study (first author, year)	Original study (first author, year)	Athlete type	Age (years)	Sex & sample size	outcome	Quality Assessment Scale (Evidence)
				F=NR)		
	Jones, 2013	EG: Obese or overweight, PW, BCSs (stage 0–IIIA)<br>CG: Obese or overweight, PW, BCSs (stage 0–IIIA)	EG: 56.4±9.6<br>CG: 55.4±7.6	EG: n=36 (M=0, F=36)<br>CG: n=31 (M=0, F=31)	CRP, TNF-α, IL-6	RoB (Tan 2025, Fig. 3): High [+/+/-/+/-/+/+]
	Rogers, 2013	EG: Obese, BCSs (stage I–IIIA)<br>CG: Obese, BCSs (stage I–IIIA)	EG: 58.0±6.1<br>CG: 53.7±13.9	EG: n=11 (M=NR, F=NR)<br>CG: n=9 (M=NR, F=NR)	TNF-α, IL-6, Leptin, IL-10	RoB (Tan 2025, Fig. 3): High [+/+/-/+/+/+/+]
	Ligibel, 2009	EG: Obese or overweight, PW, BCSs (stage I–IIIA)<br>CG: Obese or overweight, PW, BCSs (stage I–IIIA)	EG: 52±9<br>CG: 53±9	EG: n=51 (M=0, F=51)<br>CG: n=49 (M=0, F=49)	Leptin, Adiponectin	RoB (Tan 2025, Fig. 3): High [?/?/-/?/?/+/+]
	Fairey, 2005	EG: Obese or overweight, PW, BCSs (stage I–III)<br>CG: Obese or overweight, PW	EG: 59±5<br>CG: 58±6	EG: n=24 (M=0, F=24)<br>CG: n=28 (M=0, F=28)	CRP	RoB (Tan 2025, Fig. 3): High [+/+/-/+/+/+/+]
Silva 2024 (5)	Mendez-Gutierrez 2022	EG: Young sedentary adults; age range = 18–25<br>CG: Young sedentary adults; age range = 18–25	EG: mean age = 22.1±2.2 (arm-specific age NR for moderate/control); age range = 18–25<br>CG: mean age = 22.1±2.2 (arm-specific age NR for moderate/control); age range = 18–25	EG: final analysis n=37 (M/F NR; study reports 68% female at baseline)<br>CG: final analysis n=37 (M/F NR; study reports 68% female at baseline)	IL-6, Leptin, Adiponectin	RoB2 (Silva 2024, Fig. 3): High [?/+/−/+/+]
		EG: Young sedentary adults; age range = 18–25<br>CG: Young sedentary adults; age range = 18–25	EG: mean age = 22.1±2.2 (reported for vigorous-intensity group; arm-specific age NR); age range = 18–25<br>CG: mean age = 22.1±2.2 (reported for vigorous-intensity group; arm-specific age NR); age range = 18–25	EG: randomized n=110 (baseline 68% female); final analysis n=35 (M/F NR)<br>CG: final analysis n=37 (M/F NR)	IL-6, Leptin, Adiponectin	RoB2 (Silva 2024, Fig. 3): High [?/+/−/+/+]
	Streb, 2022	EG: Sedentary obese adults; age range = 20–50<br>CG: Sedentary obese adults; age range = 20–50	EG: mean age = 37.0±1.0 (reported for total sample); age range = 20–50<br>CG: mean age = 37.0±1.0 (reported for total sample); age range = 20–50	EG: randomized n=46; final analysis n=21 (M/F NR)<br>CG: randomized n=23; final analysis n=15 (M/F NR)	CRP, IL-6	RoB2 (Silva 2024, Fig. 3): Some concerns [+/ ?/ +/ +/ +]
	Amaro-Gahete, 2021	EG: Obese sedentary men; age range = 35–55<br>CG: Obese sedentary men; age range = 35–55	EG: mean age = 41.3±4.4; age range = 35–55<br>CG: mean age = 43.7±6.1; age range = 35–55	EG: n=6 (M=6, F=0)<br>CG: n=6 (M=6, F=0)	CRP, Leptin	RoB2 (Silva 2024, Fig. 3): Some concerns [+/+/+/+/?]
	Pérez-López, 2021	EG: Sedentary postmenopausal women; age range = 50–65<br>CG: Sedentary postmenopausal women; age range = 50–65	EG: mean age = 58.7±2.9; age range = 50–65<br>CG: mean age = 56.9±5.8; age range =	EG: randomized n=NR; final analysis n=13 (M=0, F=13)<br>CG: randomized n=NR; final analysis n=12 (M=0,	CRP, IL-6	RoB2 (Silva 2024, Fig. 3): Some concerns [+/ ?/ +/ +/ +]

Included study (first author, year)	Original study (first author, year)	Athlete type	Age (years)	Sex & sample size	outcome	Quality Assessment Scale (Evidence)
			50–65	F=12)		
	Shabani, 2019	EG: Sedentary overweight/obese women; age range = 50–60<br>CG: Sedentary overweight/obese women; age range = 50–60	EG: mean age = 54.83±4.72; age range = 50–60<br>CG: mean age = 56.90±4.93; age range = 50–60	EG: n=12 (M=0, F=12)<br>CG: n=12 (M=0, F=12)<br>final analysis n=10	CRP	RoB2 (Silva 2024, Fig. 3): Some concerns [?/?/+/+/?]
	Salamat, 2016	EG: Sedentary overweight men<br>CG: Sedentary overweight men	EG: mean age = 22.9±3.34<br>CG: mean age = 23.8±4.11	EG: n=11 (M=11, F=0)<br>CG: n=10 (M=10, F=0)	TNF-α, IL-6	RoB2 (Silva 2024, Fig. 3): Some concerns [?/+/+/+/?]
	Brunelli, 2015	EG: Sedentary middle-aged obese men<br>CG: Sedentary middle-aged obese men	EG: mean age = 49.29±1.31<br>CG: mean age = 48.0±1.72	EG: n=27 (M=27, F=0)<br>final analysis n=17<br>CG: n=27 (M=27, F=0)<br>final analysis n=13	CRP, TNF-α, IL-6, Leptin, Adiponectin	RoB2 (Silva 2024, Fig. 3): Some concerns [?/?/+/+/?]
	Park, 2015	EG: Sedentary postmenopausal middle-aged women with abdominal obesity<br>CG: Sedentary postmenopausal middle-aged women with abdominal obesity	EG: mean age = 57.20±2.57<br>CG: mean age = 57.20±1.69	EG: n=10 (M=0, F=10)<br>CG: n=10 (M=0, F=10)	TNF-α	RoB2 (Silva 2024, Fig. 3): Some concerns [?/+/+/+/?]
	Donges, 2013	EG: Sedentary middle-aged men; age range = 40–65<br>CG: Sedentary middle-aged men; age range = 40–65	EG: mean age = 46.2±5.05; age range = 40–65<br>CG: mean age = 49.5±7.35; age range = 40–65	EG: n=13 (M=13, F=0)<br>CG: n=8 (M=8, F=0)	CRP, TNF-α, IL-6	RoB2 (Silva 2024, Fig. 3): Some concerns [?/?/+/+/?]
	Asad, 2012	EG: Sedentary healthy male college students<br>CG: Sedentary healthy male college students	EG: mean age = 21.38±2.6<br>CG: mean age = 21.44±1.13	EG: n=14 (M=14, F=0)<br>final analysis n=13<br>CG: n=10 (M=10, F=0)	Adiponectin	RoB2 (Silva 2024, Fig. 3): Some concerns [?/+/+/+/?]
	Libardi, 2012	EG: Sedentary middle-aged healthy men<br>CG: Sedentary middle-aged healthy men	EG: mean age = 48.5±5.35<br>CG: mean age = 49.1±5.78	EG: n=11 (M=11, F=0)<br>CG: n=13 (M=13, F=0)	CRP, TNF-α, IL-6	RoB2 (Silva 2024, Fig. 3): Some concerns [?/?/+/+/?]
	Hara, 2005	EG: Young obese male subjects<br>CG: Young obese male subjects	EG: mean age = 18.4±0.5<br>CG: mean age = 19.4±1.0	EG: n=7 (M=7, F=0)<br>CG: n=7 (M=7, F=0)	Leptin, Adiponectin	RoB2 (Silva 2024, Fig. 3): Some concerns [+/+/+/+/?]
Li 2024 (6)	Peña, 2023	EG: children/adolescents with obesity/overweight (meta population)<br>CG: children/adolescents with obesity/overweight (meta population)	EG: 13.4±1.4<br>CG: 13.2±1.4	EG: n=40 (M=24, F=16)<br>CG: n=24 (M=15, F=9)	IL-6, TNF-α	Cochrane RoB: High [? / + / - / ? / + / + / +]
	Abassi, 2022	EG: children/adolescents with obesity/overweight (meta population) (female only)<br>CG: children/adolescents with obesity/overweight (meta population) (female only)	EG: 16.4 ± 1.0<br>CG:16.4 ± 1.0	EG: n=13 (M=0, F=13)<br>CG: n=12 (M=0, F=12)	CRP	Cochrane RoB: High [? / + / - / ? / + / + / +]
	Fabricio, 2022	EG: children/adolescents with obesity/overweight (meta population)<br>CG: children/adolescents with obesity/overweight (meta population)	EG: NR<br>CG: NR	EG: n=20 (M/F NR)<br>CG: n=19 (M/F NR)	Adiponectin	Cochrane RoB: High [? / - / - / ? / + / + / +]
	Nambi, 2022	EG: children/adolescents with obesity/overweight (meta population) (male only)	EG: 10.12±1.2<br>CG: 10.56±1.4	EG: n=38 (M=38, F=0)<br>CG: n=38 (M=38, F=0)	IL-6, Leptin, Adiponectin,	Cochrane RoB: High [? / + / - / ? / + / + / +]

Included study (first author, year)	Original study (first author, year)	Athlete type	Age (years)	Sex & sample size	outcome	Quality Assessment Scale (Evidence)
		CG: children/adolescents with obesity/overweight (meta population) (male only)			TNF-α	
	Liu, 2018	EG: children/adolescents with obesity/overweight (meta population) (female only)<br>CG: children/adolescents with obesity/overweight (meta population) (female only)	EG: 14.6±0.7<br>CG: 14.7±0.8	EG: n=30 (M=0, F=30)<br>CG: n=20 (M=0, F=20)	IL-6, CRP, Leptin, Adiponectin	Cochrane RoB:High[+/+/-/?/+/+/+]
	Wong,2018	EG:children/adolescents with obesity/overweight(meta population)(female only)<br>CG:children/adolescents with obesity/overweight(meta population)(female only)	EG:15.2±1.2<br>CG:15.3±1.1	EG:n=15(M=0,F=15)<br>CG:n=15(M=0,F=15)	IL-6, Leptin, Adiponectin, TNF-α	Cochrane RoB:High[?/+/-/?/+/+/+]
	Racil,2016	EG:children/adolescents with obesity/overweight(meta population)(female only)<br>CG:children/adolescents with obesity/overweight(meta population)(female only)	EG:16.5±1.2<br>CG:16.9±1.0	EG:n=26(M=0,F=26)<br>CG:n=19(M=0,F=19)	Leptin, Adiponectin	Cochrane RoB:High[?/+/-/?/+/+/+]
	Vasconcellos,2016	EG:children/adolescents with obesity/overweight(meta population)<br>CG:children/adolescents with obesity/overweight(meta population)	EG:14.1±1.3<br>CG:14.8±1.4	EG:n=10(M=8,F=2)<br>CG:n=10(M=6,F=4)	IL-6, CRP, Leptin, Adiponectin	Cochrane RoB:High[?/+/-/?/+/+/+]
	Jeon,2013	EG:children/adolescents with obesity/overweight(meta population)<br>CG:children/adolescents with obesity/overweight(meta population)	EG:NR<br>CG:NR	EG:n=8(M/F NR)<br>CG:n=7(M/F NR)	Adiponectin, TNF-α	Cochrane RoB:High[?/+/-/?/+/+/+]
	Racil,2013	EG:children/adolescents with obesity/overweight(meta population)(female only)<br>CG:children/adolescents with obesity/overweight(meta population)(female only)	EG:15.9±0.3<br>CG:15.9±0.3	EG:n=11(M=0,F=11)<br>CG:n=12(M=0,F=12)	Adiponectin	Cochrane RoB:High[?/+/-/?/+/+/+]
	Murphy,2009	EG:children/adolescents with obesity/overweight(meta population)<br>CG:children/adolescents with obesity/overweight(meta population)	EG:10.21±1.67<br>EG:10.21±1.67	EG:n=23(M/F NR)<br>CG:n=12(M/F NR)	IL-6, CRP, Adiponectin, TNF-α	Cochrane RoB:High[?/+/-/?/+/+/+]
	Kelly,2007	EG:children/adolescents with obesity/overweight(meta population)<br>CG:children/adolescents with obesity/overweight(meta population)	EG:10.8±0.67<br>CG:11.0±0.71	EG:n=9(M=4,F=5)<br>CG:n=10(M=4,F=6)	IL-6, CRP, Leptin, Adiponectin	Cochrane RoB:High[+/+/-/?/+/+/+]
Al-Mhanna-1 2024 (7)	Ligibel,2019	EG:BC patients before treatment(CART)<br>CG:BC patients before treatment(standard treatment)	EG:52.3±9.6<br>CG:53.1±7.9	EG:n=26(M/F NR)<br>CG:n=22(M/F NR)	IL-6, CRP	RoB(Al-Mhanna 2024I,Fig S1):Low[+/+/+/+/+/+/+]
	Dieli-Conwright,2018	EG:BC survivors after treatment(CART)<br>CG:BC survivors after treatment(standard treatment)	EG:52.8±10.6<br>CG:53.6±10.1	EG:n=46(M/F NR)<br>CG:n=45(M/F NR)	IL-6, IL-8, TNF-α, CRP	RoB(Al-Mhanna 2024I,Fig S1):Some concerns[?/+/?/?/+/+/+]
	Rogers,2014	EG:BC survivors after treatment(CART)<br>CG:BC survivors after treatment(standard treatment)	EG:57.2±5.5<br>CG:55.2±9.1	EG:n=20(M/F NR)<br>CG:n=22(M/F NR)	IL-6, IL-8, TNF-α	RoB(Al-Mhanna 2024I,Fig S1):Low[+/+/+/+/+/+/+]
	Saxton,2014	EG:BC survivors after treatment(CART)<br>CG:BC survivors after treatment(standard treatment)	EG:55.8±10.0<br>CG:55.3±8.8	EG:n=44(M/F NR)<br>CG:n=41(M/F NR)	IL-6, TNF-α	RoB(Al-Mhanna 2024I,Fig S1):Low[+/+/+/+/+/+/+]
	Ergun,2013	EG:BC survivors after treatment(CART)<br>CG:BC survivors after treatment(standard treatment)	EG:49.7±8.35<br>CG:50.3±10.4	EG:n=20(M/F NR)<br>CG:n=20(M/F NR)	IL-6, IL-8, TNF-α	RoB(Al-Mhanna 2024I,Fig S1):High[?/+/-/+/+/+/+]
	Scott,2013	EG:BC survivors after treatment(CART)<br>CG:BC survivors after treatment(standard treatment)	EG:55.6±10.2<br>CG:55.9±8.9	EG:n=43(M/F NR)measured n=41<br>CG:n=40(M/F NR)measured n=38	CRP	RoB (Al-Mhanna 2024I, Fig S1): Low [+/+/+/+/+/+/+]
	Hutnick, 2005	EG: BC survivors after treatment (CART)<br>CG: BC survivors after treatment (standard treatment)	EG: 48.5±10.6<br>CG: 52.3±9.2	EG: n=21 (M/F NR)<br>CG: n=15 (M/F NR)	IL-6	RoB (Al-Mhanna 2024I, Fig S1): High [?/?/-/?/?/+/+]

Included study (first author, year)	Original study (first author, year)	Athlete type	Age (years)	Sex & sample size	outcome	Quality Assessment Scale (Evidence)
AL-Mhanna-2 2024 (8)	Magalhães, 2019	EG: New Zealand communities; hypertension<br>CG: New Zealand communities; hypertension	EG: 58.5±7.7 (group split NR)<br>CG: 58.5±7.7 (group split NR)	EG: n=16 (M/F NR)<br>CG: n=22 (M/F NR)	CRP, TNF-α, IL-6	Cochrane RoB (AL-Mhanna-2 2024, Fig.3): Low [+/+/+/+/+/+/+]
	Annibalini, 2017	EG: Adults (country: Italy; population NR)<br>CG: Adults (country: Italy; population NR)	EG: 29.3±3.7 (group split NR)<br>CG: 29.3±3.7 (group split NR)	EG: n=8 (M/F NR)<br>CG: n=8 (M/F NR)	CRP, TNF-α, IL-6	Cochrane RoB (AL-Mhanna-2 2024, Fig.3): Some concerns [?/?/?/?/+/+/+]
	Jorge, 2011	EG: Overweight/obese adults with type 2 diabetes mellitus (combined aerobic + resistance training)<br>CG: Overweight/obese adults with type 2 diabetes mellitus (control)	EG: 57.90±9.82<br>CG: 53.42±9.82	EG: n=12 (M=4, F=8)<br>CG: n=12 (M=4, F=8)	CRP, TNF-α, IL-6	Cochrane RoB (AL-Mhanna-2 2024, Fig.3): Some concerns [?/?/?/?/?/+/+]
	Church, 2010	EG: Adults (comorbidities reported; population detail NR)<br>CG: Adults	EG: 55.8±8.7 (group split NR)<br>CG: 55.8±8.7 (group split NR)	EG: n=76 (M/F NR)<br>CG: n=41 (M/F NR)	CRP, TNF-α, IL-6	Cochrane RoB (AL-Mhanna-2 2024, Fig.3): High [?/?/−/+/+/+/+]
Guo, 2024 (9)	Andres E., 2022	EG: Obese population (self-control)<br>CG: NA	EG: 26.0±4.5<br>CG: NA	EG: n=13 (M/F NR)<br>CG: NA	CRP, IL-6, TNF-α	Cochrane RoB (Guo 2024, Fig.3): Some concerns [+/+/?/?/+/?/?]
	Alberto, 2021	EG: Obese population (female only)<br>CG: Obese population (female only)	EG: 43.1±2.8<br>CG: 56.9±5.8	EG: n=10 (M=0, F=10)<br>CG: n=12 (M=0, F=12)	CRP, IL-6, TNF-α	Cochrane RoB (Guo 2024, Fig.3): Some concerns [+/+/?/?/+/?/?]
		EG: Obese population (female only)<br>CG: Obese population (female only)	EG: 43.1±2.8<br>CG: 56.9±5.8	EG: n=10 (M=0, F=10)<br>CG: n=12 (M=0, F=12)	CRP, IL-6, TNF-α	Cochrane RoB (Guo 2024, Fig.3): Some concerns [+/+/?/?/+/?/?]
	Ayoub Saeidi, 2020	EG: Obese population (male only)<br>CG: Obese population (male only)	EG: 27.50±9.4<br>CG: 27.50±9.4	EG: n=11 (M=11, F=0)<br>CG: n=11 (M=11, F=0)	CRP, IL-6, TNF-α	Cochrane RoB (Guo 2024, Fig.3): Some concerns [+/+/?/?/+/?/?]
	Bik Chu Chow, 2020	EG: Obese population<br>CG: Obese population	EG: 19.7±0.9<br>CG: 19.4±0.5	EG: n=10 (M=0, F=10)<br>CG: n=11 (M=0, F=11)	CRP, IL-6, TNF-α	Cochrane RoB (Guo 2024, Fig.3): Some concerns [+/+/?/?/+/?/?]
		EG: Obese population<br>CG: Obese population	EG: 19.9±0.9<br>CG: 19.4±0.5	EG: n=10 (M=0, F=10)<br>CG: n=10 (M=0, F=10)	CRP, IL-6, TNF-α	Cochrane RoB (Guo 2024, Fig.3): Some concerns [+/+/?/?/+/?/?]
	Anne-Sophic, 2019	EG: Obese population (self-control)<br>CG: NA	EG: 39±13<br>CG: NA	EG: n=14 (M/F NR)<br>CG: NA	CRP, IL-6, TNF-α	Cochrane RoB (Guo 2024, Fig.3): Some concerns [+/+/?/?/+/?/?]
	Zhao Jun, 2019	EG: Obese population (study detail NR)<br>CG: Obese population (study detail NR)	EG: 21.68±0.98<br>CG: 21.45±1.02	EG: n=12 (M=12, F=0)<br>CG: n=12 (M=12, F=0)	CRP, IL-6, TNF-α	Cochrane RoB (Guo 2024, Fig.3): Some concerns [+/+/?/?/+/?/?]
	Mahmoud, 2018	EG: Obese population<br>CG: Obese population	EG: 40.1±3.1<br>CG: 40.1±3.1	EG: n=12 (M=12, F=0)<br>CG: n=10 (M=10, F=0)	CRP, IL-6, TNF-α	Cochrane RoB (Guo 2024, Fig.3): Some concerns [+/+/?/?/+/?/?]
	Wang Kun, 2018	EG: Obese population (self-control)<br>CG: NA	EG: 13.21±1.28<br>CG: NA	EG: n=45 (M/F NR)<br>CG: NA	CRP, IL-6, TNF-α	Cochrane RoB (Guo 2024, Fig.3): Some concerns [+/+/?/?/+/?/?]
		EG: Obese population (self-control)<br>CG: NA	EG: 13.21±1.28<br>CG: NA	EG: n=45 (M/F NR)<br>CG: NA	CRP, IL-6, TNF-α	Cochrane RoB (Guo 2024, Fig.3): Some concerns [+/+/?/?/+/?/?]
	Chantal A. Vella, 2017	EG: Obese population (self-control)<br>CG: NA	EG: 28.9±8.1<br>CG: NA	EG: n=9 (M/F NR)<br>CG: NA	CRP, IL-6, TNF-α	Cochrane RoB (Guo 2024, Fig.3): Some concerns [+/+/?/?/+/?/?]
	Thiago R.S., 2017	EG: Obese population (self-control)<br>CG: NA	EG: 15±14<br>CG: NA	EG: n=31 (M/F NR)<br>CG: NA	CRP, IL-6, TNF-α	Cochrane RoB (Guo 2024, Fig.3): Some concerns [+/+/?/?/+/?/?]
	Crisielli, 2016	EG: Obese population (detail NR)<br>CG: Obese population (detail NR)	EG: NR<br>CG: NR	EG: n=19 (M/F NR)<br>CG: n=19 (M/F NR)	CRP, IL-6, TNF-α	Cochrane RoB (Guo 2024, Fig.3): Some concerns [+/+/?/?/+/?/?]
	Chen Qiong, 2015	EG: Obese population (self-control)<br>CG: NA	EG: 14.1±3.1<br>CG: NA	EG: n=15 (M=15, F=0)<br>CG: NA	CRP, IL-6, TNF-α	Cochrane RoB (Guo 2024, Fig.3): Some concerns [+/+/?/?/+/?/?]
		EG: Obese population (self-control)<br>CG: NA	EG: 13.9±2.2<br>CG: NA	EG: n=15 (M=15, F=0)<br>CG: NA	CRP, IL-6, TNF-α	Cochrane RoB (Guo 2024, Fig.3): Some concerns [+/+/?/?/+/?/?]
	DM Croymans, 2013	EG: Obese population<br>CG: Obese population	EG: mean age = NR; age range = 20.8–22.8	EG: n=28 (M=28, F=0)<br>CG: n=8 (M=8, F=0)	CRP, IL-6, TNF-α	Cochrane RoB (Guo 2024, Fig.3): Some concerns [+/+/?/?/+/?/?]

Included study (first author, year)	Original study (first author, year)	Athlete type	Age (years)	Sex & sample size	outcome	Quality Assessment Scale (Evidence)
			CG: mean age = NR; age range = 20.8–22.8			
	Suleen S. Ho, 2013	EG: Obese population CG: Obese population	EG: mean age = 55; age range = 44–62 CG: mean age = 52; age range = 40–66	EG: n=15 (M/F NR) CG: n=16 (M/F NR)	CRP, IL-6, TNF-α	Cochrane RoB (Guo 2024, Fig.3): Some concerns [+/+/?/?/+/?/?]
		EG: Obese population CG: Obese population	EG: mean age = 52; age range = 43–59 CG: mean age = 52; age range = 40–66	EG: n=16 (M/F NR) CG: n=16 (M/F NR)	CRP, IL-6, TNF-α	Cochrane RoB (Guo 2024, Fig.3): Some concerns [+/+/?/?/+/?/?]
	Man Gyoon Lee, 2012	EG: Obese population CG: Obese population	EG: 41.6±4.5 CG: 38.3±4.9	EG: n=8 (M=0, F=8) CG: n=7 (M=0, F=7)	CRP, IL-6, TNF-α	Cochrane RoB (Guo 2024, Fig.3): Some concerns [+/+/?/?/+/?/?]
	Tore Christiansen, 2010	EG: Obese population (self-control) CG: NA	EG: 37.2±7 CG: NA	EG: n=19 (M/F NR) CG: NA	CRP, IL-6, TNF-α	Cochrane RoB (Guo 2024, Fig.3): Some concerns [+/+/?/?/+/?/?]
	Peter T. Campbell, 2009	EG: Obese population CG: Obese population	EG: 60.5±7.0 CG: 60.9±6.8	EG: n=47 (M=0, F=47) CG: n=57 (M=0, F=57)	CRP, IL-6, TNF-α	Cochrane RoB (Guo 2024, Fig.3): Some concerns [+/+/?/?/+/?/?]
	Benoit, 2009	EG: Obese population (female only) CG: Obese population (female only)	EG: 57.3±6.6 CG: 57.2±6.1	EG: n=267 (M=0, F=267) CG: n=82 (M=0, F=82)	CRP, IL-6, TNF-α	Cochrane RoB (Guo 2024, Fig.3): Some concerns [+/+/?/?/+/?/?]
	Eun Sung Kim, 2007	EG: Obese population CG: Obese population	EG: 17.0±0.1 CG: 17.0±0.1	EG: n=14 (M=14, F=0) CG: n=12 (M=12, F=0)	CRP, IL-6, TNF-α	Cochrane RoB (Guo 2024, Fig.3): Some concerns [+/+/?/?/+/?/?]
	Kelly, 2007	EG: Obese population CG: Obese population	EG: 10.8±0.67 CG: 11.0±0.71	EG: n=9 (M/F NR) CG: n=10 (M/F NR)	CRP, IL-6, TNF-α	Cochrane RoB (Guo 2024, Fig.3): Some concerns [+/+/?/?/+/?/?]
	Wang Chaoxun, 2006	EG: Obese population CG: Obese population	EG: 22.5±4.8 CG: 24.7±5.3	EG: n=32 (M/F NR) CG: n=35 (M/F NR)	CRP, IL-6, TNF-α	Cochrane RoB (Guo 2024, Fig.3): Some concerns [+/+/?/?/+/?/?]
	TONGJIAN, 2004	EG: Obese population CG: Obese population	EG: 59±1 CG: 57±1	EG: n=17 (M=0, F=17) CG: n=17 (M=0, F=17)	CRP, IL-6, TNF-α	Cochrane RoB (Guo 2024, Fig.3): Some concerns [+/+/?/?/+/?/?]
Malandish 2023 (10)	Racca, 2020	EG (Aerobic): Patients with not experience atrial fibrillation (NoAF) Patients with postoperative atrial fibrillation (POAF) Patients with permanent atrial fibrillation (AF) EG (Aerobic): Patients with not experience atrial fibrillation (NoAF) Patients with postoperative atrial fibrillation (POAF) Patients with permanent atrial fibrillation (AF) EG (Aerobic): Patients with not experience atrial fibrillation (NoAF) Patients with postoperative atrial fibrillation (POAF) Patients with permanent atrial fibrillation (AF) CG: Patients with not experience atrial fibrillation (NoAF) Patients with postoperative atrial fibrillation (POAF) Patients with permanent atrial fibrillation (AF)	EG (Aerobic): 77.5±6.5 EG (Aerobic): 77.5±6.5 EG (Aerobic): 77.5±6.5 CG: 66.2±10.6	EG (Aerobic): n=14 (M=NR, F=NR) EG (Aerobic): n=14 (M=NR, F=NR) EG (Aerobic): n=14 (M=NR, F=NR) CG: n=40 (M=NR, F=NR)	CRP, TNF-α	PEDRO scale (Score: 10, Low risk of bias)
	Redwine, 2020	EG (Aerobic): Patients with heart failure (AHA Class III) EG (Resistance): Patients with heart failure (AHA Class III) CG: Patients with heart failure (AHA Class III)	EG (Aerobic): 63±9 EG (Resistance): NR CG: NR	EG (Aerobic): n=24 (M=NR, F=NR) EG (Resistance): n=22 (M=NR, F=NR) CG: n=NR (M=NR, F=NR)	IL-6, CRP, TNF-α	PEDRO scale (Score: 13, Low risk of bias)
	Isaksen, 2019	EG: Patients with ischemic heart failure (LVEF < 40%) CG: Patients with ischemic heart failure (LVEF < 40%)	EG: 69±9 CG: 66±9	EG: n=19 (M=19, F=0) CG: n=11 (M=11, F=0)	IL-6, CRP, TNF-α	PEDRO scale (Score: 13, Low risk of bias)
	Melo, 2019	EG: Patients with chronic heart failure (atrial fibrillation) (NYHA Class II-IV) CG: Patients with chronic heart failure (atrial fibrillation) (NYHA Class II-IV)	EG: NR CG: NROverall (group not specified): 65.9±6.2; 69.4±7.2	EG: n=7 (M=NR, F=NR) CG: n=9 (M=NR, F=NR)	IL-6, CRP, TNF-α	PEDRO scale (Score: 11, Low risk of bias)

Included study (first author, year)	Original study (first author, year)	Athlete type	Age (years)	Sex & sample size	outcome	Quality Assessment Scale (Evidence)
	Melo, 2019	EG: Patients with chronic heart failure (sinus rhythm) (NYHA Class II-IV)<br>CG: Patients with chronic heart failure (sinus rhythm) (NYHA Class II-IV)	EG: NR<br>CG: NROverall (group not specified): 66.2±14.57	EG: n=11 (M=NR, F=NR)<br>CG: n=10 (M=NR, F=NR)	IL-6, CRP, TNF-α	PEDRO scale (Score: 11, Low risk of bias)
	Abolahrari-Shirazi, 2018	EG (Concurrent): Patients with heart failure (NYHA Class I- III)<br>EG (Aerobic): Patients with heart failure (NYHA Class I- III)<br>CG: Patients with heart failure (NYHA Class I- III)	EG (Concurrent): 56.76±8.71<br>EG (Aerobic): 57.64±7.85<br>CG: 57.32±9.41	EG (Concurrent): n=25 (M=NR, F=NR)<br>EG (Aerobic): n=25 (M=NR, F=NR)<br>CG: n=25 (M=NR, F=NR)	IL-6, CRP, TNF-α	PEDRO scale (Score: 15, Low risk of bias)
	Butts, 2018	EG: Patients with heart failure (NYHA Class II- III)<br>CG: Patients with heart failure (NYHA Class II- III)	EG: 60±8.7<br>CG: 58.19±12.8	EG: n=38 (M=NR, F=NR)<br>CG: n=16 (M=NR, F=NR)	IL-6, CRP, TNF-α	PEDRO scale (Score: 12, Low risk of bias)
	Fernandes-Silva, 2017	EG: Patients with heart failure (NYHA Class IV)<br>CG: Patients with heart failure (NYHA Class IV)	EG: 51±7<br>CG: 48±7	EG: n=28 (M=NR, F=NR)<br>CG: n=16 (M=NR, F=NR)	IL-6, CRP, TNF-α	PEDRO scale (Score: 12, Low risk of bias)
	Trippel, 2017	EG: Patients with heart failure<br>CG: Patients with heart failure	EG: NRCG: NROverall (group not specified): 64.4±7.2	EG: n=43 (M=NR, F=NR)<br>CG: n=19 (M=NR, F=NR)	IL-6, CRP, TNF-α	PEDRO scale (Score: 11, Low risk of bias)
	Aksoy, 2015	EG (Continuous): patients with chronic heart failure (NYHA Class II-III)<br>EG (Intermittent): patients with chronic heart failure (NYHA Class II-III)<br>CG: patients with chronic heart failure (NYHA Class II-III)	EG (Continuous): 63.7±8.8<br>EG (Intermittent): 59.6±6.9<br>CG: 57.5±11.2	EG (Continuous): n=15 (M=NR, F=NR)<br>EG (Intermittent): n=15 (M=NR, F=NR)<br>CG: n=15 (M=NR, F=NR)	IL-6, CRP, TNF-α	PEDRO scale (Score: 7, Some risk of bias)
	Masterson-Creber, 2015	EG: Patients with chronic heart failure (NYHA Class II-IV)<br>CG: Patients with chronic heart failure (NYHA Class II-IV)	EG: NR<br>CG: NR<br>Overall (group not specified): 70.1±10.75; 58.66±11.91	EG: n=163 (M=NR, F=NR)<br>CG: n=157 (M=NR, F=NR)	IL-6, CRP, TNF-α	PEDRO scale (Score: 11, Low risk of bias)
	Adamopoulos, 2014	EG: Patients with chronic heart failure (NYHA Class I-III)<br>CG: Patients with chronic heart failure (NYHA Class I-III)	EG: 57.8±11.7<br>CG: NR	EG: n=21 (M=NR, F=NR)<br>CG: n=22 (M=NR, F=NR)	IL-6, CRP, TNF-α	PEDRO scale (Score: 12, Low risk of bias)
	Ahmad, 2014	EG: Patients with chronic heart failure (NYHA Class II-IV)<br>CG: Patients with chronic heart failure (NYHA Class II-IV)	EG: 59.36±12.41<br>CG: 59.23±12.86<br>Overall (group not specified): 58.3±13.2	EG: n=477 (M=NR, F=NR)<br>CG: n=451 (M=NR, F=NR)	IL-6, CRP, TNF-α	PEDRO scale (Score: 12, Low risk of bias)
	de Meirelles, 2014	EG: Patients with heart failure (NYHA class II and III)<br>CG: Patients with heart failure (NYHA class II and III)	EG: 54±3<br>CG: 55±2	EG: n=15 (M=NR, F=NR)<br>CG: n=15 (M=NR, F=NR)	IL-6, CRP, TNF-α	PEDRO scale (Score: 11, Low risk of bias)
	Eleuteri, 2013	EG: Patients with chronic heart failure (NYHA class II)<br>CG: Patients with chronic heart failure (NYHA class II)	EG: 66±2<br>CG: 63±2	EG: n=11 (M=11, F=0)<br>CG: n=10 (M=10, F=0)	IL-6, CRP, TNF-α	PEDRO scale (Score: 13, Low risk of bias)
	Feiereisen, 2013	EG (Concurrent): Patients with chronic heart failure (NYHA class II-III)<br>EG (Resistance): Patients with chronic heart failure (NYHA class II-III)	EG (Concurrent): 60.6±5.6<br>EG (Resistance): 57.9±5.8	EG (Concurrent): n=15 (M=NR, F=NR)<br>EG (Resistance): n=15 (M=NR, F=NR)	IL-6, CRP, TNF-α	PEDRO scale (Score: 11, Low risk of bias)

Included study (first author, year)	Original study (first author, year)	Athlete type	Age (years)	Sex & sample size	outcome	Quality Assessment Scale (Evidence)
		EG (Aerobic): Patients with chronic heart failure (NYHA class II-III)<br>CG: Patients with chronic heart failure (NYHA class II-III)	EG (Aerobic): 59.4±6.5<br>CG: 55.5±7.5	EG (Aerobic): n=15 (M=NR, F=NR)<br>CG: n=15 (M=NR, F=NR)		
	Fu, 2013	EG (Aerobic interval): patients with heart failure (NYHA Class II- III)<br>EG (Aerobic continuous): patients with heart failure (NYHA Class II- III)<br>CG: patients with heart failure (NYHA Class II- III)	EG (Aerobic interval): 67.5±1.8<br>EG (Aerobic continuous): 66.3±2.1<br>CG: NR	EG (Aerobic interval): n=15 (M=NR, F=NR)<br>EG (Aerobic continuous): n=15 (M=NR, F=NR)<br>CG: n=NR (M=NR, F=NR)	IL-6, CRP, TNF-α	PEDRO scale (Score: 10, Low risk of bias)
	Marco, 2013	EG: patients with chronic heart failure (NYHA class II-III)<br>CG: patients with chronic heart failure (NYHA class II-III)	EG: 68.5±8.88<br>CG: NR<br>Overall (group not specified): 52±9.94	EG: n=11 (M=NR, F=NR)<br>CG: n=11 (M=NR, F=NR)	IL-6, CRP, TNF-α	PEDRO scale (Score: 12, Low risk of bias)
	Gielen, 2012	EG (Aerobic): Patients with chronic heart failure ≤ 55 yrs and ≥ 65 yrs (NYHA Class II-III)<br>EG (Aerobic): Patients with chronic heart failure ≤ 55 yrs and ≥ 65 yrs (NYHA Class II-III)<br>CG: Patients with chronic heart failure ≤ 55 yrs and ≥ 65 yrs (NYHA Class II-III)<br>CG: Patients with chronic heart failure ≤ 55 yrs and ≥ 65 yrs (NYHA Class II-III)	EG (Aerobic): 72±15.49<br>EG (Aerobic): 72±15.49<br>CG: 72±11.61<br>CG: 72±11.61	EG (Aerobic): n=15 (M=NR, F=NR)<br>EG (Aerobic): n=15 (M=NR, F=NR)<br>CG: n=15 (M=NR, F=NR)<br>CG: n=15 (M=NR, F=NR)	IL-6, CRP, TNF-α	PEDRO scale (Score: 14, Low risk of bias)
	Byrkjeland, 2011	EG: Patients with chronic heart failure (NYHA Class I-IIIB)<br>CG: Patients with chronic heart failure (NYHA Class I-IIIB)	EG: 68.8±7.9<br>CG: NR	EG: n=40 (M=NR, F=NR)<br>CG: n=40 (M=NR, F=NR)	IL-6, CRP, TNF-α	PEDRO scale (Score: 12, Low risk of bias)
	Giallauria, 2011	CG: Patients with acute myocardial infarction (AHA Class IIB or III)	CG: 60±8	CG: n=15 (M=NR, F=NR)	IL-6, CRP, TNF-α	PEDRO scale (Score: 14, Low risk of bias)
	Kim, 2011	EG: Patients with acute myocardial infarction<br>CG: Patients with acute myocardial infarction	EG: 61.93±10.67<br>CG: 64.49±9.31	EG: n=69 (M=NR, F=NR)<br>CG: n=72 (M=NR, F=NR)	IL-6, CRP, TNF-α	PEDRO scale (Score: 12, Low risk of bias)
	Lara Fernandes, 2011	EG: Patients with coronary artery disease<br>CG: Patients with coronary artery disease	EG: 60.7±6.7<br>CG: NR	EG: n=15 (M=NR, F=NR)<br>CG: n=19 (M=NR, F=NR)	IL-6, CRP, TNF-α	PEDRO scale (Score: 12, Low risk of bias)
	Munk, 2011	EG: Patients with angina pectoris<br>CG: Patients with angina pectoris	EG: 59.5±10<br>CG: 60.7±9	EG: n=18 (M=NR, F=NR)<br>CG: n=18 (M=NR, F=NR)	IL-6, CRP, TNF-α	PEDRO scale (Score: 14, Low risk of bias)
	Tsarouhas, 2011	EG: Patients with chronic heart failure (NYHA Class II-III)<br>CG: Patients with chronic heart failure (NYHA Class II-III)	EG: 66.8±13.1<br>CG: 67±5.6	EG: n=27 (M=NR, F=NR)<br>CG: n=12 (M=NR, F=NR)	IL-6, CRP, TNF-α	PEDRO scale (Score: 10, Low risk of bias)
	Yeh, 2011	EG: Patients with chronic heart failure (NYHA Class I-III)<br>CG: Patients with chronic heart failure (NYHA Class I-III)	EG: 68.1±11.9<br>CG: 66.6±12.1<br>Overall (group not specified): 60±7.07	EG: n=50 (M=NR, F=NR)<br>CG: n=50 (M=NR, F=NR)	IL-6, CRP, TNF-α	PEDRO scale (Score: 10, Low risk of bias)
	Erbs, 2010	EG: Patients with advanced chronic heart Failure (NYHA Class IIIB)<br>CG: Patients with advanced chronic heart Failure (NYHA	EG: 60±11<br>CG: 62±10	EG: n=18 (M=18, F=0)<br>CG: n=19 (M=19, F=0)	IL-6, CRP, TNF-α	PEDRO scale (Score: 13, Low risk of bias)

Included study (first author, year)	Original study (first author, year)	Athlete type	Age (years)	Sex & sample size	outcome	Quality Assessment Scale (Evidence)
		Class IIIB)				
	Myers, 2010	EG: Patients with abdominal aortic aneurysm<br>CG: Patients with abdominal aortic aneurysm	EG: 73.1±6<br>CG: 70.4±9	EG: n=26 (M=NR, F=NR)<br>CG: n=31 (M=NR, F=NR)	IL-6, CRP, TNF-α	PEDRO scale (Score: 13, Low risk of bias)
	Parrinello, 2010	EG: Patients with compensated<br>CG: Patients with compensated	EG: 62.3±4.9<br>CG: NR	EG: n=11 (M=NR, F=NR)<br>CG: n=11 (M=NR, F=NR)	IL-6, CRP, TNF-α	PEDRO scale (Score: 11, Low risk of bias)
	Prescott, 2009	EG: Patients with chronic systolic heart failure (NYHA Class II–IV)<br>CG: Patients with chronic systolic heart failure (NYHA Class II–IV)	EG: 68±11<br>CG: 66.9±12.5<br>Overall (group not specified): 54.2±6.4	EG: n=20 (M=NR, F=NR)<br>CG: n=23 (M=NR, F=NR)	IL-6, CRP, TNF-α	PEDRO scale (Score: 10, Low risk of bias)
	Rankovi, 2009	EG: Patients with ischemic heart disease<br>CG: Patients with ischemic heart disease	EG: 62.7±7.1<br>CG: 58.4±7.6	EG: n=22 (M=NR, F=NR)<br>CG: n=30 (M=NR, F=NR)	IL-6, CRP, TNF-α	PEDRO scale (Score: 9, Low risk of bias)
	Balen, 2008	EG: Patients with myocardial infarction<br>CG: Patients with myocardial infarction	EG: 59±9<br>CG: 61±10	EG: n=30 (M=NR, F=NR)<br>CG: n=30 (M=NR, F=NR)	IL-6, CRP, TNF-α	PEDRO scale (Score: 12, Low risk of bias)
	Pierce, 2008	EG: congestive heart failure (NYHA Class II-III) Patients with heart transplant recipients<br>CG: congestive heart failure (NYHA Class II-III) Patients with heart transplant recipients	EG: 53.5±13.6<br>CG: 63.2±5	EG: n=8 (M=NR, F=NR)<br>CG: n=6 (M=NR, F=NR)	IL-6, CRP, TNF-α	PEDRO scale (Score: 10, Low risk of bias)
	Pullen, 2008	EG: Patients with chronic heart failure (NYHA Class II-III)<br>CG: Patients with chronic heart failure (NYHA Class II-III)	EG: 52.1±3.3<br>CG: 50.5±12.8	EG: n=9 (M=NR, F=NR)<br>CG: n=10 (M=NR, F=NR)	IL-6, CRP, TNF-α	PEDRO scale (Score: 12, Low risk of bias)
	Walther, 2008	EG: Patients with coronary artery disease<br>CG: Patients with coronary artery disease	EG: 62±7.14<br>CG: NR	EG: n=51 (M=51, F=0)<br>CG: n=50 (M=50, F=0)	IL-6, CRP, TNF-α	PEDRO scale (Score: 13, Low risk of bias)
	Karavidas, 2006	EG: Patients with chronic heart failure (NYHA Class II-III)<br>CG: Patients with chronic heart failure (NYHA Class II-III)	EG: 57.4±15.3<br>CG: 63.8±8.1	EG: n=16 (M=NR, F=NR)<br>CG: n=8 (M=NR, F=NR)	IL-6, CRP, TNF-α	PEDRO scale (Score: 12, Low risk of bias)
	Shin, 2006	EG (Aerobic): Patients with coronary artery disease/ acute myocardial infarction<br>EG (Aerobic): Patients with coronary artery disease/ acute myocardial infarction<br>CG: Patients with coronary artery disease/ acute myocardial infarction<br>CG: Patients with coronary artery disease/ acute myocardial infarction	EG (Aerobic): 60.6±9.72<br>EG (Aerobic): 60.6±9.72<br>CG: 52.5±12.64<br>CG: 52.5±12.64<br>Overall (group not specified): 65±9	EG (Aerobic): n=14 (M=NR, F=NR)<br>EG (Aerobic): n=14 (M=NR, F=NR)<br>CG: n=10 (M=NR, F=NR)<br>CG: n=10 (M=NR, F=NR)	IL-6, CRP, TNF-α	PEDRO scale (Score: 10, Low risk of bias)
	Linke, 2005	EG: Patients with chronic heart failure (NYHA Class II-III)<br>CG: Patients with chronic heart failure (NYHA Class II-III)	EG: 55±6.92<br>CG: NR	EG: n=12 (M=12, F=0)<br>CG: n=11 (M=11, F=0)	IL-6, CRP, TNF-α	PEDRO scale (Score: 10, Low risk of bias)
	Niebauer, 2005	EG: Patients with chronic heart failure<br>CG: Patients with chronic heart failure	EG: 53.6±9.2<br>CG: 51.3±6.9	EG: n=18 (M=NR, F=NR)<br>CG: n=9 (M=NR, F=NR)	IL-6, CRP, TNF-α	PEDRO scale (Score: 10, Low risk of bias)
	Mc Dermott, 2004	EG: Peripheral arterial patients<br>CG: Peripheral arterial patients	EG: 69.4±9.6<br>CG: NR	EG: n=24 (M=NR, F=NR)<br>CG: n=8 (M=NR, F=NR)	IL-6, CRP, TNF-α	PEDRO scale (Score: 10, Low risk of bias)
	Milani, 2004	EG: Patients with coronary heart disease<br>CG: Patients with coronary heart disease	EG: 66.7±11<br>CG: 63.9±11.1	EG: n=235 (M=NR, F=NR)	IL-6, CRP, TNF-α	PEDRO scale (Score: 9, Low risk of bias)

Included study (first author, year)	Original study (first author, year)	Athlete type	Age (years)	Sex & sample size	outcome	Quality Assessment Scale (Evidence)
				CG: n=42 (M=NR, F=NR)		
	Kobayashi, 2003	EG: Patients with chronic heart failure (NYHA Class II-III)<br>CG: Patients with chronic heart failure (NYHA Class II-III)	EG: 55±7.48<br>CG: 62±7.48	EG: n=14 (M=NR, F=NR)<br>CG: n=14 (M=NR, F=NR)	IL-6, CRP, TNF-α	PEDRO scale (Score: 11, Low risk of bias)
	Adamopoulos, 2002	EG: Patients with chronic heart failure (NYHA Class II-III)<br>CG: Patients with chronic heart failure (NYHA Class II-III)	EG: 55.0±9.79<br>CG: NR	EG: n=24 (M=NR, F=NR)<br>CG: n=20 (M=NR, F=NR)	IL-6, CRP, TNF-α	PEDRO scale (Score: 13, Low risk of bias)
	Conraads, 2002	EG: Patients with chronic heart failure and coronary artery disease (NYHA Class I–II/ III–IV)<br>CG: Patients with chronic heart failure and coronary artery disease (NYHA Class I–II/ III–IV)	EG: 54.75±13.22<br>CG: 62.0±14.27<br>Overall (group not specified): 71.5±7.8	EG: n=23 (M=NR, F=NR)<br>CG: n=18 (M=NR, F=NR)	IL-6, CRP, TNF-α	PEDRO scale (Score: 10, Low risk of bias)
	Larsen, 2001	EG: Patients with heart failure (NYHA Class II- III)<br>CG: Patients with heart failure (NYHA Class II- III)	EG: 67±8<br>CG: 62±5<br>Overall (group not specified): 59.5±7.3	EG: n=28 (M=28, F=0)<br>CG: n=16 (M=16, F=0)	IL-6, CRP, TNF-α	PEDRO scale (Score: 10, Low risk of bias)
	Tisi, 1997	EG: Patients with intermittent claudication<br>CG: Patients with intermittent claudication	EG: NR<br>CG: NR<br>Overall (group not specified): 69.3; 66.2	EG: n=67 (M=NR, F=NR)<br>CG: n=15 (M=NR, F=NR)	IL-6, CRP, TNF-α	PEDRO scale (Score: 13, Low risk of bias)
Dragoumani 2023 (11)	Kahhan, 2021	EG: children with overweight/obesity (BMI%ile ≥85; Tanner stage ≤3); intervention=family-based lifestyle program (INT)<br>CG: children with overweight/obesity (BMI%ile ≥85; Tanner stage ≤3); control=education-only (EDU)	Total: mean age = 10	Total: n=87 (M=29, F=58)	CRP, leptin, adiponectin, TNF-α, IL-6	RoB2: Some concerns [?/?/?/+/?]
	Thomsen, 2021	EG: overweight children (11–13 years); intervention=intensive day-camp (increased physical activity + healthy diet)<br>CG: overweight children (11–13 years); control=standard intervention arm	EG: 12±NR<br>CG: NA	EG: n=99 (M=45, F=54)<br>CG: NA	CRP, leptin, adiponectin, TNF-α, IL-6	RoB2: Some concerns [?/?/?/+/?]
	Mietus-Snyder, 2020	EG: adolescents with obesity (Teen BMI >95th percentile; 14–18 years) + parent adult caretaker (PAC) family units; intervention=lifestyle counseling + supervised group exercise + nutrient bar supplementation (INT)<br>CG: adolescents with obesity (Teen BMI >95th percentile; 14–18 years) + PAC family units; control=lifestyle counseling + supervised group exercise only (CONT)	Total: mean age = 15.5	Total: n=18 (M/F=NR)	CRP, Adiponectin	RoB2: Some concerns [?/?/+/+/?]
	Seo, 2019	EG: children/adolescents with overweight/obesity (age 6–16; BMI ≥85th percentile); intervention=exercise group (16-week program)<br>CG: children/adolescents with overweight/obesity (age 6–16; BMI ≥85th percentile); comparison=usual care group (16-week program)	Total: mean age = 12.5	Total: n=70 (M=45, F=25)	CRP	RoB2: High [−/?/?/+/?]
	Wong, 2018	EG: obese adolescent girls (BMI ≥95th percentile; sedentary; with hyperinsulinemia and abdominal obesity); intervention=combined exercise training (CET)<br>CG: obese adolescent girls (BMI ≥95th percentile; sedentary); control=no-exercise	Total: mean age = 15.3	Total: n=30 (M=0, F=30)	CRP, Leptin, Adiponectin	RoB2: Some concerns [?/?/?/+/?]
	Rynders, 2012	EG: obese pubertal adolescents (10–17 years);	EG: 14.2±NR	EG: n=16 (M=7, F=9)	IL-6	RoB2: High [?/?/−/+/?]

Included study (first author, year)	Original study (first author, year)	Athlete type	Age (years)	Sex & sample size	outcome	Quality Assessment Scale (Evidence)
		intervention=diet + exercise (DE) + metformin (DEM)<br>CG: obese pubertal adolescents (10–17 years); comparison=diet + exercise only (DE)	CG: NA	CG: NA		
	Vos, 2011	EG: children with obesity; intervention=family-based multidisciplinary cognitive behavioral lifestyle treatment (3 months)<br>CG: children with obesity; control=standard care/advice on physical activity and nutrition	Total: mean age = 13.2	Total: n=113 (M=51, F=62)	CRP, Adiponectin	RoB2: Some concerns [?/?/?/+/?]
	Pedrosa, 2010	EG: Portuguese overweight/obese schoolchildren (7–9 years); intervention=individual treatment (IT) within 1-year outpatient lifestyle program<br>CG: Portuguese overweight/obese schoolchildren (7–9 years); comparison=group-based treatment (GT) within 1-year outpatient lifestyle program	Total: mean age = 8.7	Total: n=61 (M=27, F=34)	CRP, Leptin, Adiponectin	RoB2: Some concerns [?/?/?/+/?]
	Park, 2007	EG: obese adolescent girls (13–15 years; Korea); intervention=lifestyle plus exercise (LIFE+EX)<br>CG: obese adolescent girls (13–15 years; Korea); control=maintain usual lifestyle (CON)	Total: mean age = 14.2	Total: n=40 (M=0, F=40)	CRP, Leptin, Adiponectin	RoB2: Some concerns [?/?/?/+/?]
	Balagopal, 2005	EG: obese adolescents (Tanner stage IV); intervention=3-month lifestyle-only intervention (randomized)<br>CG: obese adolescents (Tanner stage IV); control=maintain lifestyle (per protocol)	Total: mean age = 15.8	Total: n=21 (M=11, F=10)	CRP, Adiponectin, IL-6	RoB2: Some concerns [?/?/?/+/?]
Tan 2023 (12)	Biteli, 2021	EG: Obese, PW; intervention=AE<br>CG: Obese, PW; intervention=CON	EG: 58.5±6.5<br>CG: 61.2±7.7	EG: n=11 (M=NR, F=NR)<br>CG: n=13 (M=NR, F=NR)	CRP, TNF-α, IL-6, Adiponectin	Cochrane RoB (NR, Fig 3): High [+/?/-/?/?/+/?]
	Kortas, 2020	EG: Obese/Overweight, PW; intervention=AE<br>CG: Obese/Overweight, PW; intervention=CON	EG: 66.7±4.76<br>CG: 66.1±4.83	EG: n=18 (M=NR, F=NR)<br>CG: n=18 (M=NR, F=NR)	CRP, TNF-α, IL-6, Adiponectin	Cochrane RoB (NR, Fig 3): High [+/+/-/+/+/+/ ?]
	Vasconcelos, 2020	EG: Obese/Overweight, PW; intervention=CE<br>CG: Obese/Overweight, PW; intervention=CON	EG: 64.8±3.03<br>CG: 65.9±5.79	EG: n=16 (M=NR, F=NR)<br>CG: n=11 (M=NR, F=NR)	CRP, TNF-α, IL-6, Adiponectin	Cochrane RoB (NR, Fig 3): High [+/+/-/+/+/+/+]
	Saeidi, 2019	EG: Obese/Overweight, PW; intervention=RT<br>CG: Obese/Overweight, PW; intervention=CON	EG: 58±5<br>CG: 56±5	EG: n=12 (M=NR, F=NR)<br>CG: n=12 (M=NR, F=NR)	CRP, TNF-α, IL-6, Adiponectin	Cochrane RoB (NR, Fig 3): High [+/ ?/-/+/+/+/+]
	Urzi, 2019	EG: Obese/Overweight, PW; intervention=RT<br>CG: Obese/Overweight, PW; intervention=CON	EG: 84.4±7.7<br>CG: 88.9±5.3	EG: n=11 (M=NR, F=NR)<br>CG: n=9 (M=NR, F=NR)	CRP, TNF-α, IL-6, Adiponectin	Cochrane RoB (NR, Fig 3): High [+/?/-/+/+/+/+]
	Chupel, 2018	EG: Obese/Overweight, PW; intervention=CE<br>CG: Obese/Overweight, PW; intervention=CON	EG: 83.5±7.3<br>CG: 82±7.5	EG: n=13 (M=NR, F=NR)<br>CG: n=12 (M=NR, F=NR)	CRP, TNF-α, IL-6, Adiponectin	Cochrane RoB (NR, Fig 3): High [?/?/-/+/+/+/+]
	Cunha, 2018	EG: Obese/Overweight, PW; intervention=RT<br>CG: Obese/Overweight, PW; intervention=CON	EG: 71.4±5.71<br>CG: 69.4±4.45	EG: n=25 (M=NR, F=NR)<br>CG: n=23 (M=NR, F=NR)	CRP, TNF-α, IL-6, Adiponectin	Cochrane RoB (NR, Fig 3): High [?/?/-/?/+/+/ ?]
	Gómez-Tomás, 2018	EG: Obese/Overweight, PW; intervention=RT	EG: 70.8±4.42	EG: n=18 (M=NR,	CRP, TNF-α, IL-6,	Cochrane RoB (NR, Fig 3): High [+/+/-/+/+/+/+]

Included study (first author, year)	Original study (first author, year)	Athlete type	Age (years)	Sex & sample size	outcome	Quality Assessment Scale (Evidence)
		CG: Obese/Overweight, PW; intervention=CON	CG: 70.4±5.44	F=NR)<br>CG: n=20 (M=NR, F=NR)	Adiponectin	
	Tomeleri, 2018	EG: Obese/Overweight, PW; intervention=RT<br>CG: Obese/Overweight, PW; intervention=CON	EG: 72.1±6.3<br>CG: 68.8±4.9	EG: n=22 (M=NR, F=NR)<br>CG: n=23 (M=NR, F=NR)	CRP, TNF-α, IL-6, Adiponectin	Cochrane RoB (NR, Fig 3): High [+/+/-/+/+/+/+]
	Yoon, 2018	EG: Obese/Overweight, PW; intervention=AE<br>CG: Obese/Overweight, PW; intervention=CON	EG: 53.7±3.37<br>CG: 52.5±2.68	EG: n=10 (M=NR, F=NR)<br>CG: n=10 (M=NR, F=NR)	CRP, TNF-α, IL-6, Adiponectin	Cochrane RoB (NR, Fig 3): High [+/+/-/+/+/+/+]
		EG: Obese/Overweight, PW; intervention=RT<br>CG: Obese/Overweight, PW; intervention=CON	EG: 52.2±2.15<br>CG: 52.5±2.68	EG: n=10 (M=NR, F=NR)<br>CG: n=10 (M=NR, F=NR)	CRP, TNF-α, IL-6, Adiponectin	Cochrane RoB (NR, Fig 3): High [+/+/-/+/+/+/+]
	Abdollahpour, 2017	EG: Obese, PW; intervention=AE<br>CG: Obese, PW; intervention=CON	EG: 58.8±6.4<br>CG: 55.3±5.2	EG: n=22 (M=NR, F=NR)<br>CG: n=19 (M=NR, F=NR)	CRP, TNF-α, IL-6, Adiponectin	Cochrane RoB (NR, Fig 3): High [?/?/-/?/+/+/+]
	Baitul, 2017	EG: Obese, PW; intervention=AE<br>CG: Obese, PW; intervention=CON	EG: 46.7±1.30<br>CG: 46.7±1.35	EG: n=12 (M=NR, F=NR)<br>CG: n=12 (M=NR, F=NR)	CRP, TNF-α, IL-6, Adiponectin	Cochrane RoB (NR, Fig 3): High [?/?/-/?/?/+/?]
	Chagas, 2017	EG: Overweight, PW; intervention=AE<br>CG: Overweight, PW; intervention=CON	EG: 61.3±6.4<br>CG: 59.8±7.1	EG: measured n=35<br>CG: measured n=35	CRP, TNF-α, IL-6, Adiponectin	Cochrane RoB (NR, Fig 3): High [+/+/-/+/+/+/+]
	Chupel, 2017	EG: Obese/Overweight, PW; intervention=RT<br>CG: Obese/Overweight, PW; intervention=CON	EG: 83.5±5.13<br>CG: 82.1±6.41	EG: n=16 (M=NR, F=NR)<br>CG: n=17 (M=NR, F=NR)	CRP, TNF-α, IL-6, Adiponectin	Cochrane RoB (NR, Fig 3): High [?/?/-/+/+/+/+]
	Abd El-Kader, 2016	EG: Obese/Overweight, PW; intervention=AE<br>CG: Obese/Overweight, PW; intervention=CON	EG: 51.1±5.63<br>CG: 50.4±5.27	EG: n=40 (M=NR, F=NR)<br>CG: n=40 (M=NR, F=NR)	CRP, TNF-α, IL-6, Adiponectin	Cochrane RoB (NR, Fig 3): High [+/+/-/+/+/+/+]
	Nunes, 2016	EG: Obese/Overweight, PW; intervention=RT<br>CG: Obese/Overweight, PW; intervention=CON	EG: 62.0±2.7<br>CG: 62.0±2.7	EG: n=11 (M=NR, F=NR)<br>CG: n=11 (M=NR, F=NR)	CRP, TNF-α, IL-6, Adiponectin	Cochrane RoB (NR, Fig 3): High [+/ ?/-/+/+/+/+]
	Rezende, 2016	EG: Obese, PW, NAFLD; intervention=AE<br>CG: Obese, PW, NAFLD; intervention=CON	EG: 56.2±7.8<br>CG: 54.5±8.9	EG: n=19 (M=NR, F=NR)<br>CG: n=21 (M=NR, F=NR)	CRP, TNF-α, IL-6, Adiponectin	Cochrane RoB (NR, Fig 3): High [+/+/-/+/+/+/+]
	Tomeleri, 2016	EG: Obese/Overweight, PW; intervention=RT<br>CG: Obese/Overweight, PW; intervention=CON	EG: 66.8±3.2<br>CG: 69.5±4.7	EG: n=19 (M=NR, F=NR)<br>CG: n=19 (M=NR, F=NR)	CRP, TNF-α, IL-6, Adiponectin	Cochrane RoB (NR, Fig 3): High [+/+/-/+/+/+/+]
	Park, 2015	EG: Obese/Overweight, PW; intervention=CE<br>CG: Obese/Overweight, PW; intervention=CON	EG: 57.2±2.57<br>CG: 57.2±1.69	EG: n=10 (M=NR, F=NR)<br>CG: n=10 (M=NR, F=NR)	CRP, TNF-α, IL-6, Adiponectin	Cochrane RoB (NR, Fig 3): High [+/+/-/+/+/+/+]
	Tartibian, 2015	EG: Obese/Overweight, PW; intervention=AE<br>CG: Obese/Overweight, PW; intervention=CON	EG: 57.1±7.5<br>CG: 57.2±2.2	EG: n=14 (M=NR, F=NR)	CRP, TNF-α, IL-6, Adiponectin	Cochrane RoB (NR, Fig 3): High [?/?/-/?/?/+/+]

Included study (first author, year)	Original study (first author, year)	Athlete type	Age (years)	Sex & sample size	outcome	Quality Assessment Scale (Evidence)
				CG: n=14 (M=NR, F=NR)		
	Wang, 2015	EG: Obese/Overweight, PW; intervention=AE<br>CG: Obese/Overweight, PW; intervention=CON	EG: 58.4±5.2<br>CG: 58.5±6.1	EG: n=48 (M=NR, F=NR)<br>CG: n=22 (M=NR, F=NR)	CRP, TNF-α, IL-6, Adiponectin	Cochrane RoB (NR, Fig 3): High [+/+/-/+/+/+/+]
	Ryan, 2014	EG: Obese, PW; intervention=AE<br>CG: Obese, PW; intervention=CON	EG: 60±1<br>CG: 61±1	EG: n=37 (M=NR, F=NR)<br>CG: n=40 (M=NR, F=NR)	CRP, TNF-α, IL-6, Adiponectin	Cochrane RoB (NR, Fig 3): High [?/?/-/?/+/+/+]
	Abbenhardt, 2013	EG: Obese/Overweight, PW; intervention=AE<br>CG: Obese/Overweight, PW; intervention=CON	EG: 58.1±5.0<br>CG: 57.4±4.4	EG: n=117 (M=NR, F=NR)<br>CG: n=87 (M=NR, F=NR)	CRP, TNF-α, IL-6, Adiponectin	Cochrane RoB (NR, Fig 3): High [+/+/-/+/+/+/+]
	Figueroa, 2013	EG: Obese/Overweight, PW; intervention=RT<br>CG: Obese/Overweight, PW; intervention=CON	EG: 54±1<br>CG: 54±1	EG: n=14 (M=NR, F=NR)<br>CG: n=13 (M=NR, F=NR)	CRP, TNF-α, IL-6, Adiponectin	Cochrane RoB (NR, Fig 3): High [?/?/-/+/+/+/+]
	Imayama, 2012	EG: Obese/Overweight, PW; intervention=AE<br>CG: Obese/Overweight, PW; intervention=CON	EG: 58.1±5.0<br>CG: 57.4±4.4	EG: n=117 (M=NR, F=NR)<br>CG: n=89 (M=NR, F=NR)	CRP, TNF-α, IL-6, Adiponectin	Cochrane RoB (NR, Fig 3): High [+/+/-/+/+/+/+]
	Johannsen, 2012	EG: Obese/Overweight, PW; intervention=AE<br>CG: Obese/Overweight, PW; intervention=CON	EG: 56.6±6.5<br>CG: 57.1±5.7	EG: n=103 (M=NR, F=NR)<br>CG: n=102 (M=NR, F=NR)	CRP, TNF-α, IL-6, Adiponectin	Cochrane RoB (NR, Fig 3): High [+/+/-/+/ -/+/+]
	Lee, 2012	EG: Obese/Overweight, PW; intervention=AE<br>CG: Obese/Overweight, PW; intervention=CON	EG: 54.7±2.76<br>CG: 54.2±2.91	EG: n=8 (M=NR, F=NR)<br>CG: n=8 (M=NR, F=NR)	CRP, TNF-α, IL-6, Adiponectin	Cochrane RoB (NR, Fig 3): High [+/ ?/-/+/+/+/+]
	Phillips, 2012	EG: Obese/Overweight, PW; intervention=RT<br>CG: Obese/Overweight, PW; intervention=CON	EG: 64.8±2.4<br>CG: 66.4±2.8	EG: n=11 (M=NR, F=NR)<br>CG: n=12 (M=NR, F=NR)	CRP, TNF-α, IL-6, Adiponectin	Cochrane RoB (NR, Fig 3): High [?/?/-/?/?/+/?]
	Ku, 2010	EG: Obese/Overweight, PW; intervention=AE<br>CG: Obese/Overweight, T2DM; intervention=CON	EG: 55.7±7.0<br>CG: 57.8±8.1	EG: n=15 (M=NR, F=NR)<br>CG: n=16 (M=NR, F=NR)	CRP, TNF-α, IL-6, Adiponectin	Cochrane RoB (NR, Fig 3): High [+/?/-/?/+/+/+]
		EG: Obese/Overweight, T2DM; intervention=RT<br>CG: Obese/Overweight, T2DM; intervention=CON	EG: 55.7±6.2<br>CG: 57.8±8.1	EG: n=13 (M=NR, F=NR)<br>CG: n=16 (M=NR, F=NR)	CRP, TNF-α, IL-6, Adiponectin	Cochrane RoB (NR, Fig 3): High [+/?/-/?/+/+/+]
	Arsenault, 2009	EG: Obese/Overweight, PW; intervention=AE<br>CG: Obese, PW; intervention=CON	EG: 57.3±6.6<br>CG: 57.2±6.1	EG: n=267 (M=NR, F=NR)<br>CG: n=82 (M=NR, F=NR)	CRP, TNF-α, IL-6, Adiponectin	Cochrane RoB (NR, Fig 3): High [+/+/-/+/+/+/+]
	Silverman, 2009	EG: Obese, PW; intervention=AE<br>CG: Obese, PW; intervention=CON	EG: 60±5<br>CG: 58±5	EG: n=46 (M=NR, F=NR)<br>CG: n=40 (M=NR, F=NR)	CRP, TNF-α, IL-6, Adiponectin	Cochrane RoB (NR, Fig 3): High [?/?/-/?/+/+/+]
	Campbell, 2008	EG: Obese, PW; intervention=AE<br>CG: Obese, PW; intervention=CON	EG: 60.5±7.0<br>CG: 60.9±6.8	EG: n=53 (M=NR, F=NR) measured n=47<br>CG: n=62 (M=NR,	CRP, TNF-α, IL-6, Adiponectin	Cochrane RoB (NR, Fig 3): High [+/ ?/-/+/+/+/+]

Included study (first author, year)	Original study (first author, year)	Athlete type	Age (years)	Sex & sample size	outcome	Quality Assessment Scale (Evidence)
				F=NR) measured n=57		
	Fairey, 2005	EG: Obese/Overweight, PW; intervention=AE<br>CG: Obese/Overweight, PW; intervention=CON	EG: 59±5<br>CG: 58±6	EG: n=24 (M=NR, F=NR)<br>CG: n=28 (M=NR, F=NR)	CRP, TNF-α, IL-6, Adiponectin	Cochrane RoB (NR, Fig 3): High [+/+/-/+/+/+/ ?]
	You, 2004	EG: Obese, PW; intervention=AE<br>CG: Obese, PW; intervention=CON	EG: 59±1<br>CG: 57±1	EG: n=15 (M=NR, F=NR) measured n=17<br>CG: n=15 (M=NR, F=NR) measured n=17	CRP, TNF-α, IL-6, Adiponectin	Cochrane RoB (NR, Fig 3): High [?/?/-/?/?/+/+]
Al-Mhanna 2023 (13)	Legaard 2022	EG: T2DM (<10 years; BMI 25–40; no insulin); intervention=exercise-based lifestyle (12 months)<br>CG: T2DM; standard care	EG: 53.3±9.4<br>CG: 56.2±8.0	EG: n=56 (M=30, F=26)<br>CG: n=21 (M=11, F=10)	TNF-α	Cochrane RoB (this review, Fig.3): Low [+/+/+/+/+/+/+]
	Abd El-Kader 2020	EG: T2DM; intervention=diet control + aerobic treadmill (3 months; 3 sessions/week; 30 min at 60–70% HRmax; 1200 kcal/day)<br>CG: T2DM; no intervention	EG: 41.5±5.2<br>CG: 42.2±4.9	EG: n=50 (M=34, F=16)<br>CG: n=50 (M=32, F=18)	TNF-α	Cochrane RoB (this review, Fig.3): Some concerns [?/?/?/?/+/+/+]
Del Rosso 2023 (14)	Reljic 2022	EG: OB/MetS; intervention=AeT / RT (12 weeks)<br>CG: NR	EG: 53.7±11.4<br>CG: NR	EG: n=104 (M/F NR)<br>CG: n=NR (M/F NR)	CRP, IL-1β, IL-6, adiponectin	NR (no quality assessment reported)
	Magalhães 2020	EG: OB/T2DM; intervention=AeT / HIIT (52 weeks)<br>CG: NR	EG: 58.4±8.6<br>CG: NR	EG: n=80 (M/F NR)<br>CG: n=NR (M/F NR)	CRP, IL-6, TNF-α	NR (no quality assessment reported)
	Nono 2020	EG: OB; intervention=COMB (12 weeks)<br>CG: NR	EG: 23.5±3.5<br>CG: NR	EG: n=35 (M=0, F=35)<br>CG: n=NR (M/F NR)	CRP, TNF-α, IL-8, leptin, adiponectin	NR (no quality assessment reported)
	Cunha 2019	EG: OW; intervention=RT (12 weeks)<br>CG: NR	EG: 70.2±5.1<br>CG: NR	EG: n=48 (M=0, F=48)<br>CG: n=NR (M/F NR)	CRP	NR (no quality assessment reported)
	Kim 2019	EG: OW; intervention=COMB (12 weeks)<br>CG: NR	EG: 68.8±0.9<br>CG: NR	EG: n=20 (M=20, F=0)<br>CG: n=NR (M/F NR)	CRP, leptin	NR (no quality assessment reported)
	Kolahdouzi 2019	EG: OB; intervention=RT (8 weeks)<br>CG: NR	EG: 23.0±3.2<br>CG: NR	EG: n=30 (M=30, F=0)<br>CG: n=NR (M/F NR)	CRP	NR (no quality assessment reported)
	Ratajczak 2019	EG: OB; intervention=AeT / COMB (12 weeks)<br>CG: NR	EG: 50.0±9.0<br>CG: NR	EG: n=39 (M=0, F=39)<br>CG: n=NR (M/F NR)	CRP	NR (no quality assessment reported)
	Rejeski 2019	EG: OB; intervention=D+AeT / D+RT (78 weeks)<br>CG: NR	EG: 66.9±4.7<br>CG: NR	EG: n=222 (M/F NR)<br>CG: n=NR (M/F NR)	CRP, IL-6	NR (no quality assessment reported)
	Coll-Risco 2018	EG: OW; intervention=COMB (16 weeks)<br>CG: NR	EG: 52.8±4.5<br>CG: NR	EG: n=150 (M=0, F=150)<br>CG: n=NR (M/F NR)	CRP	NR (no quality assessment reported)
	Eizadi 2018	EG: OB; intervention=AeT (12 weeks)<br>CG: NR	EG: mean age=NR; age range=30–40<br>CG: NR	EG: n=30 (M=0, F=30)<br>CG: n=NR (M/F NR)	IL-10	NR (no quality assessment reported)
	Fedewa 2018	EG: OW/OB; intervention=AeT / HIIT (6 weeks)<br>CG: NR	EG: 20.2±1.7<br>CG: NR	EG: n=44 (M=0, F=44)<br>CG: n=NR (M/F NR)	CRP	NR (no quality assessment reported)
	Hsieh 2018	EG: T2DM; intervention=RT (12 weeks)<br>CG: NR	EG: 71.2±4.3<br>CG: NR	EG: n=30 (M/F NR)<br>CG: n=NR (M/F NR)	CRP	NR (no quality assessment reported)
	Martins 2018	EG: OW; intervention=HIIT / COMB (12 weeks)<br>CG: NR	EG: 64.6±6.5<br>CG: NR	EG: n=16 (M=0, F=16)<br>CG: n=NR (M/F NR)	CRP, IL-6	NR (no quality assessment reported)
	Mora-Rodriguez 2018	EG: MetS; intervention=AeT (26 weeks)<br>CG: NR	EG: 53.5±8.9<br>CG: NR	EG: n=46 (M/F NR)<br>CG: n=NR (M/F NR)	CRP	NR (no quality assessment reported)
	Nikseresht 2018	EG: OB; intervention=RT (12 weeks)<br>CG: NR	EG: 39.6±3.1<br>CG: NR	EG: n=22 (M=22, F=0)<br>CG: n=NR (M/F NR)	IL-6, IL-10	NR (no quality assessment reported)
	Rohde 2018	EG: OW; intervention=PA (22 weeks)<br>CG: NR	EG: 37.7±7.6<br>CG: NR	EG: n=115 (M=115, F=0)<br>CG: n=NR (M/F NR)	CRP	NR (no quality assessment reported)
	Rodriguez-Krause 2018	EG: OW; intervention=AeT (8 weeks)	EG: 65.0±5.0	EG: n=30 (M=0, F=30)	CRP, TNF-α	NR (no quality assessment reported)

Included study (first author, year)	Original study (first author, year)	Athlete type	Age (years)	Sex & sample size	outcome	Quality Assessment Scale (Evidence)
		CG: NR	CG: NR	CG: n=NR (M/F NR)		
	Tomeleri 2018	EG: OW; intervention=RT (12 weeks)<br>CG: NR	EG: 70.4±5.7<br>CG: NR	EG: n=45 (M=0, F=45)<br>CG: n=NR (M/F NR)	CRP, IL-6, TNF-α	NR (no quality assessment reported)
	Zhang 2018	EG: T2DM/OW; intervention=AeT (12 weeks)<br>CG: NR	EG: 47.1±10.8<br>CG: NR	EG: n=32 (M/F NR)<br>CG: n=NR (M/F NR)	CRP, leptin, adiponectin	NR (no quality assessment reported)
	Annibalini 2017	EG: T2DM; intervention=COMB (16 weeks)<br>CG: NR	EG: 58.5±7.9<br>CG: NR	EG: n=16 (M=16, F=0)<br>CG: n=NR (M/F NR)	CRP, IL-6, TNF-α, leptin, adiponectin	NR (no quality assessment reported)
	Balducci 2017	EG: T2DM/OB; intervention=PA (16 weeks)<br>CG: NR	EG: 61.6±9.9<br>CG: NR	EG: n=300 (M/F NR)<br>CG: n=NR (M/F NR)	CRP	NR (no quality assessment reported)
	Chagas 2017	EG: OW/OB; intervention=COMB (20 weeks)<br>CG: NR	EG: 60.5±6.7<br>CG: NR	EG: n=70 (M=0, F=70)<br>CG: n=NR (M/F NR)	IL-6, TNF-α, IL-10	NR (no quality assessment reported)
	Flandez 2017	EG: MetS/OB; intervention=RT (12 weeks)<br>CG: NR	EG: 46.5±3.7<br>CG: NR	EG: n=62 (M=0, F=62)<br>CG: n=NR (M/F NR)	CRP	NR (no quality assessment reported)
	Galedari 2017	EG: OW; intervention=D+AeT / D+HIIT / D+RT (12 weeks)<br>CG: NR	EG: 30.9±7.0<br>CG: NR	EG: n=40 (M=40, F=0)<br>CG: n=NR (M/F NR)	TNF-α	NR (no quality assessment reported)
	Gram 2017	EG: OW; intervention=AeT / PA (26 weeks)<br>CG: NR	EG: 34.7±2.6<br>CG: NR	EG: n=90 (M/F NR)<br>CG: n=NR (M/F NR)	CRP	NR (no quality assessment reported)
	Henriquez 2017	EG: OW/OB; intervention=AeT / RT (26 weeks)<br>CG: NR	EG: 56.5±5.6<br>CG: NR	EG: n=34 (M=0, F=34)<br>CG: n=NR (M/F NR)	CRP, IL-6	NR (no quality assessment reported)
	Rahbar 2017	EG: T2DM/OW; intervention=AeT (8 weeks)<br>CG: NR	EG: 48.5±4.9<br>CG: NR	EG: n=28 (M/F NR)<br>CG: n=NR (M/F NR)	CRP	NR (no quality assessment reported)
	Vella 2017	EG: OW/OB; intervention=AeT / HIIT (8 weeks)<br>CG: NR	EG: 26.2±7.8<br>CG: NR	EG: n=17 (M/F NR)<br>CG: n=NR (M/F NR)	CRP, IL-6, IL-8, TNF-α, leptin, adiponectin	NR (no quality assessment reported)
	Conroy 2016	EG: OW; intervention=AeT (52 weeks)<br>CG: NR	EG: 60.9±5.5<br>CG: NR	EG: n=310 (M=0, F=310)<br>CG: n=NR (M/F NR)	IL-10	NR (no quality assessment reported)
	Nikseresht 2016	EG: OB; intervention=AeT / RT (12 weeks)<br>CG: NR	EG: 39.6±4.3<br>CG: NR	EG: n=33 (M/F NR)<br>CG: n=NR (M/F NR)	IL-18	NR (no quality assessment reported)
	Nunes 2016	EG: OW; intervention=RT (16 weeks)<br>CG: NR	EG: 60.9±8.9<br>CG: NR	EG: n=32 (M=0, F=32)<br>CG: n=NR (M/F NR)	IL-6, TNF-α	NR (no quality assessment reported)
	Salamat 2016	EG: OW; intervention=AeT / RT / COMB (8 weeks)<br>CG: NR	EG: 23.7±3.3<br>CG: NR	EG: n=43 (M/F NR)<br>CG: n=NR (M/F NR)	IL-6, IL-1α, TNF-α	NR (no quality assessment reported)
	Shahram 2016	EG: OW; intervention=AeT / RT (12 weeks)<br>CG: NR	EG: 22.5±1.6<br>CG: NR	EG: n=30 (M=0, F=30)<br>CG: n=NR (M/F NR)	IL-6, IL-10, TNF-α	NR (no quality assessment reported)
	Tan 2016	EG: OW; intervention=AeT (10 weeks)<br>CG: NR	EG: 50.2±6.9<br>CG: NR	EG: n=30 (M=0, F=30)<br>CG: n=NR (M/F NR)	leptin	NR (no quality assessment reported)
	Tomeleri 2016	EG: OW; intervention=RT (8 weeks)<br>CG: NR	EG: 68.1±3.9<br>CG: NR	EG: n=38 (M=0, F=38)<br>CG: n=NR (M/F NR)	CRP, IL-6, TNF-α	NR (no quality assessment reported)
	Weiss 2016	EG: OW; intervention=D+PA (14 weeks)<br>CG: NR	EG: 57.0±5.0<br>CG: NR	EG: n=52 (M/F NR)<br>CG: n=NR (M/F NR)	CRP	NR (no quality assessment reported)
	Abd El-Kader 2015	EG: T2DM/OB; intervention=AeT (12 weeks)<br>CG: NR	EG: 43.8±6.0<br>CG: NR	EG: n=80 (M/F NR)<br>CG: n=NR (M/F NR)	IL-6, IL-8, TNF-α	NR (no quality assessment reported)
	Desde 2015	EG: T2DM/OB; intervention=AeT (12 weeks)<br>CG: NR	EG: 54.0±8.0<br>CG: NR	EG: n=60 (M/F NR)<br>CG: n=NR (M/F NR)	adiponectin, leptin	NR (no quality assessment reported)
	Franklin 2015	EG: OW/OB; intervention=RT (8 weeks)<br>CG: NR	EG: 30.5±7.2<br>CG: NR	EG: n=18 (M=0, F=18)<br>CG: n=NR (M/F NR)	CRP, TNF-α	NR (no quality assessment reported)
	Keating 2015	EG: OB; intervention=AeT (8 weeks)<br>CG: NR	EG: 41.6±2.8<br>CG: NR	EG: n=48 (M/F NR)<br>CG: n=NR (M/F NR)	CRP	NR (no quality assessment reported)
	Kim 2015	EG: OW; intervention=AeT (8 weeks)	EG: 25.7±2.8	EG: n=39 (M/F NR)	leptin, adiponectin	NR (no quality assessment reported)

Included study (first author, year)	Original study (first author, year)	Athlete type	Age (years)	Sex & sample size	outcome	Quality Assessment Scale (Evidence)
		CG: NR	CG: NR	CG: n=NR (M/F NR)		
	Mendham 2015	EG: T2DM/OB; intervention=COMB (12 weeks)<br>CG: NR	EG: 37.8±13.3<br>CG: NR	EG: n=21 (M/F NR)<br>CG: n=NR (M/F NR)	CRP, IL-1α, IL-6, IL-10, TNF-α, leptin, adiponectin	NR (no quality assessment reported)
	Vatani 2015	EG: OB; intervention=COMB (8 weeks)<br>CG: NR	EG: 23.2±1.4<br>CG: NR	EG: n=30 (M/F NR)<br>CG: n=NR (M/F NR)	leptin	NR (no quality assessment reported)
	Wang 2015	EG: OB; intervention=D+AeT (20 weeks)<br>CG: NR	EG: 58.5±5.6<br>CG: NR	EG: n=70 (M=0, F=70)<br>CG: n=NR (M/F NR)	adiponectin	NR (no quality assessment reported)
	Ahmadizad 2014	EG: OW; intervention=RT (8 weeks)<br>CG: NR	EG: 23.4±0.6<br>CG: NR	EG: n=32 (M=32, F=0)<br>CG: n=NR (M/F NR)	leptin, adiponectin	NR (no quality assessment reported)
	Croymans 2014	EG: OB; intervention=RT (12 weeks)<br>CG: NR	EG: 21.6±1.9<br>CG: NR	EG: n=36 (M/F NR)<br>CG: n=NR (M/F NR)	CRP, IL-8	NR (no quality assessment reported)
	Mendham 2014	EG: OW; intervention=AeT (8 weeks)<br>CG: NR	EG: 48.6±6.6<br>CG: NR	EG: n=32 (M/F NR)<br>CG: n=NR (M/F NR)	CRP, IL-6, TNF-α, IL-1α, IL-10, leptin, adiponectin	NR (no quality assessment reported)
	Mavros 2014	EG: T2DM/OB; intervention=RT (12 weeks)<br>CG: NR	EG: 68.2±5.7<br>CG: NR	EG: n=88 (M/F NR)<br>CG: n=NR (M/F NR)	CRP	NR (no quality assessment reported)
	Nikseresht 2014	EG: OB; intervention=AeT / RT (12 weeks)<br>CG: NR	EG: 39.6±4.3<br>CG: NR	EG: n=34 (M/F NR)<br>CG: n=NR (M/F NR)	IL-10, TNF-α	NR (no quality assessment reported)
	Abbenhardt 2013	EG: OW/OB; intervention=AeT / D+AeT (52 weeks)<br>CG: NR	EG: 57.8±4.7<br>CG: NR	EG: n=204 (M=0, F=204)<br>CG: n=NR (M/F NR)	leptin, adiponectin	NR (no quality assessment reported)
	Akbarpour 2013	EG: OW/OB; intervention=AeT (12 weeks)<br>CG: NR	EG: 22.9±2.6<br>CG: NR	EG: n=60 (M=60, F=0)<br>CG: n=NR (M/F NR)	CRP, IL-6, adiponectin, leptin	NR (no quality assessment reported)
	Donges 2013	EG: OW/OB; intervention=AeT / RT / COMB (12 weeks)<br>CG: NR	EG: 48.2±1.9<br>CG: NR	EG: n=47 (M=47, F=0)<br>CG: n=NR (M/F NR)	CRP, IL-6, TNF-α	NR (no quality assessment reported)
	Figueroa 2013	EG: OB; intervention=D+RT (12 weeks)<br>CG: NR	EG: 54.0±1.0<br>CG: NR	EG: n=41 (M=0, F=41)<br>CG: n=NR (M/F NR)	leptin, adiponectin	NR (no quality assessment reported)
	Lakhdar 2013	EG: OW/OB; intervention=D+AeT (24 weeks)<br>CG: NR	EG: 38.0±4.4<br>CG: NR	EG: n=30 (M=0, F=30)<br>CG: n=NR (M/F NR)	IL-6, TNF-α	NR (no quality assessment reported)
	Loria-Kohen 2013	EG: OW; intervention=D+AeT / D+RT / D+COMB (22 weeks)<br>CG: NR	EG: 36.4±8.3<br>CG: NR	EG: n=84 (M/F NR)<br>CG: n=NR (M/F NR)	CRP, IL-6, TNF-α, leptin	NR (no quality assessment reported)
	Trussardi Fayh 2013	EG: OB; intervention=D+AeT (12 weeks)<br>CG: NR	EG: 31.8±6.0<br>CG: NR	EG: n=35 (M/F NR)<br>CG: n=NR (M/F NR)	CRP	NR (no quality assessment reported)
	Wanderley 2013	EG: OW; intervention=AeT / RT (34 weeks)<br>CG: NR	EG: 68.3±5.5<br>CG: NR	EG: n=50 (M/F NR)<br>CG: n=NR (M/F NR)	CRP, IL-6, TNF-α	NR (no quality assessment reported)
	Asad 2012	EG: OW/OB; intervention=AeT / RT / COMB (8 weeks)<br>CG: NR	EG: 21.4±1.4<br>CG: NR	EG: n=44 (M=44, F=0)<br>CG: n=NR (M/F NR)	adiponectin	NR (no quality assessment reported)
	Canuto 2012	EG: OB; intervention=COMB (12 weeks)<br>CG: NR	EG: 40.2±10.8<br>CG: NR	EG: n=59 (M=0, F=59)<br>CG: n=NR (M/F NR)	CRP	NR (no quality assessment reported)
	Choi 2012	EG: T2DM/OW; intervention=AeT (12 weeks)<br>CG: NR	EG: 54.4±6.6<br>CG: NR	EG: n=75 (M=0, F=75)<br>CG: n=NR (M/F NR)	CRP, IL-6	NR (no quality assessment reported)
	Friedenreich 2012	EG: OW; intervention=AeT (52 weeks)<br>CG: NR	EG: 60.9±5.5<br>CG: NR	EG: n=310 (M=0, F=310)<br>CG: n=NR (M/F NR)	CRP, IL-6, TNF-α	NR (no quality assessment reported)
	Imayama 2012	EG: OW/OB; intervention=AeT / D+AeT (52 weeks)<br>CG: NR	EG: 57.9±4.6<br>CG: NR	EG: n=438 (M=0, F=438)<br>CG: n=NR (M/F NR)	CRP, IL-6	NR (no quality assessment reported)
	Kadoglou et al -2 2012	EG: T2DM/OB; intervention=RT (12 weeks)<br>CG: NR	EG: 61.3±2.1<br>CG: NR	EG: n=47 (M/F NR)<br>CG: n=NR (M/F NR)	CRP	NR (no quality assessment reported)
	Moghadasi 2012	EG: OW/OB; intervention=AeT (12 weeks)<br>CG: NR	EG: 41.2±6.1<br>CG: NR	EG: n=16 (M=16, F=0)<br>CG: n=NR (M/F NR)	CRP, adiponectin	NR (no quality assessment reported)

Included study (first author, year)	Original study (first author, year)	Athlete type	Age (years)	Sex & sample size	outcome	Quality Assessment Scale (Evidence)
	Phillips 2012	EG: OB; intervention=RT (12 weeks)<br>CG: NR	EG: 65.6±2.6<br>CG: NR	EG: n=23 (M=0, F=23)<br>CG: n=NR (M/F NR)	CRP, IL-6, leptin, adiponectin	NR (no quality assessment reported)
	Sukala 2012	EG: T2DM/OB; intervention=AeT / RT (16 weeks)<br>CG: NR	EG: 49.0±5.0<br>CG: NR	EG: n=18 (M/F NR)<br>CG: n=NR (M/F NR)	CRP, adiponectin	NR (no quality assessment reported)
	Fisher 2011	EG: OW; intervention=D+AeT / D+RT (8 weeks)<br>CG: NR	EG: mean age=NR; age range=20–41<br>CG: NR	EG: n=126 (M=0, F=126)<br>CG: n=NR (M/F NR)	CRP, TNF-α, IL-6	NR (no quality assessment reported)
	Jorge 2011	EG: T2DM/OB; intervention=AeT / RT / COMB (12 weeks)<br>CG: NR	EG: 54.3±9.3<br>CG: NR	EG: n=48 (M/F NR)<br>CG: n=NR (M/F NR)	CRP, IL-6, adiponectin	NR (no quality assessment reported)
	Straznicky 2011	EG: MetS/OB; intervention=D+AeT (12 weeks)<br>CG: NR	EG: 54.3±1.6<br>CG: NR	EG: n=38 (M/F NR)<br>CG: n=NR (M/F NR)	CRP, leptin	NR (no quality assessment reported)
	Wu 2011	EG: DR/OW; intervention=PA (39 weeks)<br>CG: NR	EG: 54.0±5.0<br>CG: NR	EG: n=135 (M/F NR)<br>CG: n=NR (M/F NR)	adiponectin	NR (no quality assessment reported)
	Balducci 2010a	EG: T2DM/OB; intervention=COMB (52 weeks)<br>CG: NR	EG: 58.8±8.5<br>CG: NR	EG: n=563 (M/F NR)<br>CG: n=NR (M/F NR)	CRP	NR (no quality assessment reported)
	Balducci 2010b	EG: MetS/T2DM/OB; intervention=AeT / COMB / PA (52 weeks)<br>CG: NR	EG: 62.0±7.9<br>CG: NR	EG: n=82 (M/F NR)<br>CG: n=NR (M/F NR)	CRP, IL-1α, IL-6, IL-10, TNF-α, leptin, adiponectin	NR (no quality assessment reported)
	Christiansen 2010	EG: OB; intervention=D+AeT (52 weeks)<br>CG: NR	EG: 36.7±7.3<br>CG: NR	EG: n=59 (M/F NR)<br>CG: n=NR (M/F NR)	CRP, IL-6, IL-18, adiponectin	NR (no quality assessment reported)
	Ibanez 2010	EG: OB; intervention=D+RT (16 weeks)<br>CG: NR	EG: 50.0±6.2<br>CG: NR	EG: n=34 (M=0, F=34)<br>CG: n=NR (M/F NR)	leptin, adiponectin	NR (no quality assessment reported)
	Martins 2010	EG: OW; intervention=AeT / RT (16 weeks)<br>CG: NR	EG: 76.8±7.2<br>CG: NR	EG: n=45 (M/F NR)<br>CG: n=NR (M/F NR)	CRP	NR (no quality assessment reported)
	Okada 2010	EG: T2DM/OW; intervention=COMB (12 weeks)<br>CG: NR	EG: 63.2±7.2<br>CG: NR	EG: n=38 (M/F NR)<br>CG: n=NR (M/F NR)	CRP, leptin, adiponectin	NR (no quality assessment reported)
	Pasqualini 2010	EG: HBP/OW; intervention=AeT (8 weeks)<br>CG: NR	EG: 43.5±6.5<br>CG: NR	EG: n=48 (M/F NR)<br>CG: n=NR (M/F NR)	adiponectin	NR (no quality assessment reported)
	Plotnikoff 2010	EG: T2DM/OB; intervention=RT (16 weeks)<br>CG: NR	EG: 54.5±12.0<br>CG: NR	EG: n=48 (M/F NR)<br>CG: n=NR (M/F NR)	CRP	NR (no quality assessment reported)
	Stewart 2010	EG: OB; intervention=AeT (26 weeks)<br>CG: NR	EG: 57.3±6.4<br>CG: NR	EG: n=411 (M=0, F=411)<br>CG: n=NR (M/F NR)	CRP	NR (no quality assessment reported)
	Thompson 2010	EG: OW; intervention=AeT (24 weeks)<br>CG: NR	EG: 53.0±4.5<br>CG: NR	EG: n=41 (M=41, F=0)<br>CG: n=NR (M/F NR)	CRP, IL-6	NR (no quality assessment reported)
	Arsenault 2009	EG: HBP/OB; intervention=AeT (26 weeks)<br>CG: NR	EG: 57.2±6.4<br>CG: NR	EG: n=349 (M=0, F=349)<br>CG: n=NR (M/F NR)	CRP, adiponectin, TNF-α, IL-6	NR (no quality assessment reported)
	Campbell 2009	EG: OB; intervention=AeT (52 weeks)<br>CG: NR	EG: 60.7±6.9<br>CG: NR	EG: n=108 (M=0, F=108)<br>CG: n=NR (M/F NR)	CRP, IL-6	NR (no quality assessment reported)
	Herder 2009	EG: IGT/OB; intervention=D+PA (52 weeks)<br>CG: NR	EG: 55.4±7.0<br>CG: NR	EG: n=406 (M/F NR)<br>CG: n=NR (M/F NR)	CRP, IL-6	NR (no quality assessment reported)
	Loimaala 2009	EG: T2DM; intervention=COMB (104 weeks)<br>CG: NR	EG: 53.9±5.6<br>CG: NR	EG: n=48 (M=48, F=0)<br>CG: n=NR (M/F NR)	leptin	NR (no quality assessment reported)
	Silverman 2009	EG: OB; intervention=D+AeT (26 weeks)<br>CG: NR	EG: 59.0±5.0<br>CG: NR	EG: n=86 (M=0, F=86)<br>CG: n=NR (M/F NR)	IL-6, TNF-α	NR (no quality assessment reported)
	Smith 2009	EG: OB; intervention=PA (16 weeks)<br>CG: NR	EG: 43.7±6.1<br>CG: NR	EG: n=41 (M/F NR)<br>CG: n=NR (M/F NR)	CRP, TNF-α	NR (no quality assessment reported)
	Vieira 2009	EG: OW; intervention=AeT (43 weeks)<br>CG: NR	EG: 70.0±0.7<br>CG: NR	EG: n=127 (M/F NR)<br>CG: n=NR (M/F NR)	CRP	NR (no quality assessment reported)
	Campbell 2008	EG: OW/OB; intervention=AeT (12 weeks)	EG: 55.2±6.7	EG: n=188 (M/F NR)	CRP	NR (no quality assessment reported)

Included study (first author, year)	Original study (first author, year)	Athlete type	Age (years)	Sex & sample size	outcome	Quality Assessment Scale (Evidence)
		CG: NR	CG: NR	CG: n=NR (M/F NR)		
	Kadoglou 2007	EG: T2DM/OB; intervention=AeT (26 weeks)<br>CG: NR	EG: 61.5±5.9<br>CG: NR	EG: n=60 (M/F NR)<br>CG: n=NR (M/F NR)	CRP, adiponectin, TNF-α, IL-18, IL-10	NR (no quality assessment reported)
	Murakami 2007	EG: OW; intervention=D+AeT (12 weeks)<br>CG: NR	EG: 49.6±2.0<br>CG: NR	EG: n=42 (M/F NR)<br>CG: n=NR (M/F NR)	leptin	NR (no quality assessment reported)
	Olson 2007	EG: OW; intervention=RT (52 weeks)<br>CG: NR	EG: 38.5±5.5<br>CG: NR	EG: n=28 (M=0, F=28)<br>CG: n=NR (M/F NR)	CRP, IL-6, adiponectin	NR (no quality assessment reported)
	Rokling-Andersen 2007	EG: MetS/OW; intervention=COMB / D+COMB (52 weeks)<br>CG: NR	EG: 45.1±2.5<br>CG: NR	EG: n=188 (M=188, F=0)<br>CG: n=NR (M/F NR)	CRP, IL-6, TNF-α, IL-8, leptin, adiponectin	NR (no quality assessment reported)
	Fatouros 2005	EG: OW; intervention=RT (52 weeks)<br>CG: NR	EG: 70.3±3.8<br>CG: NR	EG: n=50 (M=50, F=0)<br>CG: n=NR (M/F NR)	leptin, adiponectin	NR (no quality assessment reported)
	Frank 2005	EG: OB; intervention=AeT (52 weeks)<br>CG: NR	EG: 60.6±6.7<br>CG: NR	EG: n=170 (M=0, F=170)<br>CG: n=NR (M/F NR)	leptin	NR (no quality assessment reported)
	Hara 2005	EG: OB; intervention=AeT / COMB (8 weeks)<br>CG: NR	EG: 19.2±1.1<br>CG: NR	EG: n=21 (M=0, F=21)<br>CG: n=NR (M/F NR)	leptin, adiponectin	NR (no quality assessment reported)
	Marcell 2005	EG: OB/IR; intervention=AeT / PA (16 weeks)<br>CG: NR	EG: 45.3±8.3<br>CG: NR	EG: n=51 (M/F NR)<br>CG: n=NR (M/F NR)	CRP, adiponectin	NR (no quality assessment reported)
	Thong 2000	EG: OB; intervention=AeT / D+AeT (12 weeks)<br>CG: NR	EG: 44±1.8<br>CG: NR	EG: n=52 (M=52, F=0)<br>CG: n=NR (M/F NR)	leptin	NR (no quality assessment reported)
Rahimi 2022 (15)	Beavers, 2013	EG: mixed sex adults (BMI 33±4 kg/m²)<br>CG: mixed sex adults (BMI 33±4 kg/m²)	EG: 67±5<br>CG: 67±5	EG: n=98 (M=NR, F=NR)<br>CG: n=93 (M=NR, F=NR)	CRP, IL-6, Adiponectin	TESTEX (Rahimi 2021, Table S1): 10/15 [1/1/0/1/0/2/0/2/1/1/0/1]
	Oh, 2013	EG: female adults (Korea; BMI 26±4 kg/m²)<br>CG: female adults (Korea; BMI 26±4 kg/m²)	EG: 60±8<br>CG: 67±9	EG: n=31 (M=0, F=31)<br>CG: n=21 (M=0, F=21)	CRP, IL-6, Adiponectin	TESTEX (Rahimi 2021, Table S1): 9/15 [1/1/0/1/0/1/1/2/1/0/0/1]
	Nanri, 2012	EG: male adults (Japan; BMI 26±2 kg/m²)<br>CG: male adults (Japan; BMI 26±2 kg/m²)	EG: 54±6<br>CG: 53±7	EG: n=49 (M=49, F=0)<br>CG: n=53 (M=53, F=0)	CRP, IL-6, Adiponectin	TESTEX (Rahimi 2021, Table S1): 10/15 [1/1/0/1/0/2/1/2/1/0/0/1]
	Camhi, 2010	EG: female adults (BMI 26±4 kg/m²)<br>CG: female adults (BMI 26±4 kg/m²)	EG: 57±5<br>CG: 58±5	EG: n=12 (M=0, F=12)<br>CG: n=9 (M=0, F=9)	CRP, IL-6, Adiponectin	TESTEX (Rahimi 2021, Table S1): 9/15 [1/1/0/1/0/2/0/2/1/0/0/1]
		EG: male adults (BMI 27±3 kg/m²)<br>CG: male adults (BMI 27±3 kg/m²)	EG: 49±8<br>CG: 49±10	EG: n=14 (M=14, F=0)<br>CG: n=9 (M=9, F=0)	CRP, IL-6, Adiponectin	TESTEX (Rahimi 2021, Table S1): 9/15 [1/1/0/1/0/2/0/2/1/0/0/1]
	Herder, 2009	EG: mixed sex adults (Germany; BMI 31±5 kg/m²)<br>CG: mixed sex adults (Germany; BMI 31±5 kg/m²)	EG: 56±7<br>CG: 55±7	EG: n=265 (M=NR, F=NR) measured n=207<br>CG: n=257 (M=NR, F=NR) measured n=199	CRP, IL-6, Adiponectin	TESTEX (Rahimi 2021, Table S1): 11/15 [1/1/0/1/1/2/1/2/1/0/0/1]
	Bo, 2007	EG: mixed sex adults (Italy; BMI 30±4 kg/m²)<br>CG: mixed sex adults (Italy; BMI 30±5 kg/m²)	EG: 56±6<br>CG: 56±6	EG: n=169 (M=NR, F=NR)<br>CG: n=166 (M=NR, F=NR)	CRP, IL-6, Adiponectin	TESTEX (Rahimi 2021, Table S1): 10/15 [1/1/1/1/1/1/0/2/1/0/0/1]
Zhao 2022 (16)	Plavsic, 2020	EG: adolescents; HIIT<br>CG: adolescents; control	EG: 15.5±1.5<br>CG: 16.2±1.3	EG: n=22 (M=0, F=22)<br>CG: n=22 (M=0, F=22)	CRP, TNF-α, IL-6	Cochrane RoB (Zhao 2022, Fig.2): Low [+/+/+/+/+/+/+]
	Wong, 2018	EG: adolescents; AT+RT<br>CG: adolescents; control	EG: 15.2±1.2<br>CG: 15.3±1.1	EG: n=15 (M=0, F=15)<br>CG: n=15 (M=0, F=15)	CRP, TNF-α, IL-6	Cochrane RoB (Zhao 2022, Fig.2): Some concerns [+/+/?/+/+/+/+]
	Lopes, 2016	EG: adolescents; AT+RT<br>CG: adolescents; control	EG: 14.6±1.15<br>CG: 14.4±1.16	EG: n=17 (M=0, F=17)<br>CG: n=16 (M=0, F=16)	CRP, TNF-α, IL-6	Cochrane RoB (Zhao 2022, Fig.2): Some concerns [+/+/?/+/+/+/+]
	Alberga, 2015	EG: adolescents; AT<br>CG: adolescents; control	EG: 15.5±1.4<br>CG: 15.6±1.3	EG: n=75 (M=22, F=53)<br>CG: n=76 (M=24, F=52)	CRP, TNF-α, IL-6	Cochrane RoB (Zhao 2022, Fig.2): Some concerns [?/+/?/+/+/+/+]
		EG: adolescents; RT	EG: 15.9±1.5	EG: n=78 (M=23, F=55)	CRP, TNF-α, IL-6	Cochrane RoB (Zhao 2022, Fig.2): Some concerns

Included study (first author, year)	Original study (first author, year)	Athlete type	Age (years)	Sex & sample size	outcome	Quality Assessment Scale (Evidence)
		CG: adolescents; control	CG: 15.6±1.3	CG: n=76 (M=24, F=52)		[?/+/?/+/+/+/+]
		EG: adolescents; AT+RT<br>CG: adolescents; control	EG: 15.5±1.3<br>CG: 15.6±1.3	EG: n=75 (M=22, F=53)<br>CG: n=76 (M=24, F=52)	CRP, TNF-α, IL-6	Cochrane RoB (Zhao 2022, Fig.2): Some concerns [?/+/?/+/+/+/+]
	Chen, 2015	EG: adolescents; AT<br>CG: adolescents; control	EG: 14.1±3.1<br>CG: 14.4±3.2	EG: n=15 (M=15, F=0)<br>CG: n=15 (M=15, F=0)	CRP, TNF-α, IL-6	Cochrane RoB (Zhao 2022, Fig.2): High [?/+/-/?/+/+/+]
		EG: adolescents; RT<br>CG: adolescents; control	EG: 13.9±2.2<br>CG: 14.4±3.2	EG: n=15 (M=15, F=0)<br>CG: n=15 (M=15, F=0)	CRP, TNF-α, IL-6	Cochrane RoB (Zhao 2022, Fig.2): High [?/+/-/?/+/+/+]
		EG: adolescents; AT+RT<br>CG: adolescents; control	EG: 14.2±3.8<br>CG: 14.4±3.2	EG: n=15 (M=15, F=0)<br>CG: n=15 (M=15, F=0)	CRP, TNF-α, IL-6	Cochrane RoB (Zhao 2022, Fig.2): High [?/+/-/?/+/+/+]
	Filho, 2015	EG: adolescents; AT+RT<br>CG: adolescents; control	EG: 13.4±1.3<br>CG: 13.7±0.9	EG: n=13 (M=6, F=7)<br>CG: n=12 (M=6, F=6)	CRP, TNF-α, IL-6	Cochrane RoB (Zhao 2022, Fig.2): Some concerns [?/+/?/+/+/+/+]
	Nunes, 2015	EG: adolescents; AT+RT<br>CG: adolescents; control	EG: 16.18±1.51<br>CG: 15.4±1.2	EG: n=17 (M=8, F=9)<br>CG: n=8 (M=4, F=4)	CRP, TNF-α, IL-6	Cochrane RoB (Zhao 2022, Fig.2): Some concerns [?/+/+/+/+/+/+]
	Vasconcellos, 2015	EG: adolescents; AT<br>CG: adolescents; control	EG: 14.1±1.3<br>CG: 14.8±1.4	EG: n=10 (M=8, F=2)<br>CG: n=10 (M=6, F=4)	CRP, TNF-α, IL-6	Cochrane RoB (Zhao 2022, Fig.2): Some concerns [+/+/?/+/+/+/+]
	Park, 2012	EG: adolescents; AT+RT<br>CG: adolescents; control	EG: 12.1±0.1<br>CG: 12.2±0.1	EG: n=15 (M=7, F=8)<br>CG: n=14 (M=7, F=7)	CRP, TNF-α, IL-6	Cochrane RoB (Zhao 2022, Fig.2): Some concerns [+/?/?/+/+/+/+]
	Lee, 2010	EG: adolescents; AT<br>CG: adolescents; control	EG: 13±1<br>CG: 13±1	EG: n=16 (M=NR, F=NR)<br>CG: n=18 (M=NR, F=NR)	CRP, TNF-α, IL-6	Cochrane RoB (Zhao 2022, Fig.2): High [+/?/-/+/+/+/+]
		EG: adolescents; AT+RT<br>CG: adolescents; control	EG: 13±1<br>CG: 13±1	EG: n=20 (M=NR, F=NR)<br>CG: n=18 (M=NR, F=NR)	CRP, TNF-α, IL-6	Cochrane RoB (Zhao 2022, Fig.2): High [+/?/-/+/+/+/+]
	Wong, 2008	EG: adolescents; AT+RT<br>CG: adolescents; control	EG: 13.75±1.06<br>CG: 14.25±1.54	EG: n=12 (M=12, F=0)<br>CG: n=12 (M=12, F=0)	CRP, TNF-α, IL-6	Cochrane RoB (Zhao 2022, Fig.2): High [?/?/-/+/+/+/+]
	Kim, 2007	EG: adolescents; AT<br>CG: adolescents; control	EG: 17±0.11<br>CG: 17±0.11	EG: n=14 (M=14, F=0)<br>CG: n=12 (M=12, F=0)	CRP, TNF-α, IL-6	Cochrane RoB (Zhao 2022, Fig.2): High [?/+/-/+/+/+/+]
	Park, 2007	EG: adolescents; AT<br>CG: adolescents; control	EG: 14.2±0.5<br>CG: 14.1±0.5	EG: n=19 (M=0, F=19)<br>CG: n=21 (M=0, F=21)	CRP, TNF-α, IL-6	Cochrane RoB (Zhao 2022, Fig.2): High [+/+/-/+/+/+/+]
	Meyer, 2006	EG: adolescents; AT<br>CG: adolescents; control	EG: 13.7±2.1<br>CG: 14.7±2.2	EG: n=33 (M=17, F=16)<br>CG: n=34 (M=17, F=17)	CRP, TNF-α, IL-6	Cochrane RoB (Zhao 2022, Fig.2): Low [+/+/+/+/+/+/+]
Hejazi-1 2022 (17)	Mohammad Rahimi 2021	EG: Obese/Metabolic ; mode=AE<br>CG: Obese/Metabolic ; mode=CON	EG: 45.5±NR<br>CG: 45.5±NR	EG: n=10 (M=10, F=0)<br>CG: n=3 (M=3, F=0)	Adiponectin, TNF-α, IL-6, Leptin, CRP	TESTEX (Hejzi-1, Table S1): 12 [1/1/1/1/1/2/0/2/1/0/1/1]
		EG: Obese/Metabolic ; mode=RT<br>CG: Obese/Metabolic ; mode=CON	EG: 45.5±NR<br>CG: 45.5±NR	EG: n=10 (M=10, F=0)<br>CG: n=3 (M=3, F=0)	Adiponectin, TNF-α, IL-6, Leptin, CRP	TESTEX (Hejzi-1, Table S1): 12 [1/1/1/1/1/2/0/2/1/0/1/1]
		EG: Obese/Metabolic ; mode=AIE+RT<br>CG: Obese/Metabolic ; mode=CON	EG: 45.5±NR<br>CG: 45.5±NR	EG: n=10 (M=10, F=0)<br>CG: n=4 (M=4, F=0)	Adiponectin, TNF-α, IL-6, Leptin, CRP	TESTEX (Hejzi-1, Table S1): 12 [1/1/1/1/1/2/0/2/1/0/1/1]
	Armamento-Villareal 2020	EG: Obese ; mode=AE<br>CG: Obese ; mode=CON	EG: 70±NR<br>CG: 70±NR	EG: n=40 (M=NR, F=NR)<br>CG: n=13 (M=NR, F=NR)	Adiponectin, TNF-α, IL-6, Leptin, CRP	TESTEX (Hejzi-1, Table S1): 12 [1/0/0/1/1/2/1/2/1/1/1/1]
		EG: Obese ; mode=RT<br>CG: Obese ; mode=CON	EG: 70±NR<br>CG: 70±NR	EG: n=40 (M=NR, F=NR)<br>CG: n=13 (M=NR, F=NR)	Adiponectin, TNF-α, IL-6, Leptin, CRP	TESTEX (Hejzi-1, Table S1): 12 [1/0/0/1/1/2/1/2/1/1/1/1]

Included study (first author, year)	Original study (first author, year)	Athlete type	Age (years)	Sex & sample size	outcome	Quality Assessment Scale (Evidence)
		EG: Obese ; mode=AE+RT<br>CG: Obese ; mode=CON	EG: 70±NR<br>CG: 70±NR	EG: n=40 (M=NR, F=NR)<br>CG: n=14 (M=NR, F=NR)	Adiponectin, TNF-α, IL-6, Leptin, CRP	TESTEX (Hejzi-1, Table S1): 12 [1/0/0/1/1/2/1/2/1/1/1/1]
	Bagheri 2020	EG: Overweight ; mode=AE<br>CG: Overweight ; mode=CON	EG: 44.13±NR<br>CG: 44.13±NR	EG: n=15 (M=15, F=0)<br>CG: n=15 (M=15, F=0)	Adiponectin, TNF-α, IL-6, Leptin, CRP	TESTEX (Hejzi-1, Table S1): 11 [1/1/1/1/1/1/0/2/1/0/1/1]
	Kortas 2020	EG: Overweight (Poland); mode=AE<br>CG: Overweight (Poland); mode=CON	EG: 66.45±NR<br>CG: 66.45±NR	EG: n=18 (M=0, F=18)<br>CG: n=18 (M=0, F=18)	Adiponectin, TNF-α, IL-6, Leptin, CRP	TESTEX (Hejzi-1, Table S1): 10 [1/1/0/1/0/2/0/2/1/0/1/1]
	Saghebjoo 2019	EG: Overweight ; mode=HIIT<br>CG: Overweight ; mode=CON	EG: 24.5±NR<br>CG: 24.5±NR	EG: n=10 (M=10, F=0)<br>CG: n=10 (M=10, F=0)	Adiponectin, TNF-α, IL-6, Leptin, CRP	TESTEX (Hejzi-1, Table S1): 7 [1/0/0/1/0/1/0/2/1/0/0/1]
	Banaeifar 2017	EG: Obese ; mode=AE<br>CG: Obese ; mode=CON	EG: 38±NR<br>CG: 38±NR	EG: n=12 (M=12, F=0)<br>CG: n=12 (M=12, F=0)	Adiponectin, TNF-α, IL-6, Leptin, CRP	TESTEX (Hejzi-1, Table S1): 8 [1/0/0/1/0/1/0/2/1/0/1/1]
	Koh 2017	EG: Obese ; mode=AE<br>CG: Obese ; mode=CON	EG: 41.5±NR<br>CG: 41.5±NR	EG: n=15 (M=NR, F=NR)<br>CG: n=12 (M=NR, F=NR)	Adiponectin, TNF-α, IL-6, Leptin, CRP	TESTEX (Hejzi-1, Table S1): 7 [1/0/0/1/0/1/0/2/1/0/0/1]
	Pourvaghar 2017	EG: Overweight ; mode=HIIT<br>CG: Overweight ; mode=CON	EG: 22.9±NR<br>CG: 22.9±NR	EG: n=12 (M=12, F=0)<br>CG: n=12 (M=12, F=0)	Adiponectin, TNF-α, IL-6, Leptin, CRP	TESTEX (Hejzi-1, Table S1): 8 [1/0/0/1/0/1/0/2/1/0/1/1]
	Shakeri 2016	EG: Overweight ; mode=AE<br>CG: Overweight ; mode=CON	EG: 22.58±NR<br>CG: 22.58±NR	EG: n=10 (M=0, F=10)<br>CG: n=10 (M=0, F=10)	Adiponectin, TNF-α, IL-6, Leptin, CRP	TESTEX (Hejzi-1, Table S1): 8 [1/0/0/1/0/1/0/2/1/0/1/1]
	Bouri 2015	EG: Overweight ; mode=HIIT<br>CG: Overweight ; mode=CON	EG: 23.14±NR<br>CG: 23.14±NR	EG: n=7 (M=0, F=7)<br>CG: n=7 (M=0, F=7)	Adiponectin, TNF-α, IL-6, Leptin, CRP	TESTEX (Hejzi-1, Table S1): 8 [1/0/0/1/0/1/0/2/1/0/1/1]
	Kim 2015	EG: Obese ; mode=AE<br>CG: Obese ; mode=CON	EG: 25.7±NR<br>CG: 25.7±NR	EG: n=29 (M=29, F=0)<br>CG: n=10 (M=10, F=0)	Adiponectin, TNF-α, IL-6, Leptin, CRP	TESTEX (Hejzi-1, Table S1): 9 [1/1/0/1/0/1/0/2/1/0/1/1]
	Moradi 2015	EG: Obese ; mode=RT<br>CG: Obese ; mode=CON	EG: 26.95±NR<br>CG: 26.95±NR	EG: n=10 (M=10, F=0)<br>CG: n=11 (M=11, F=0)	Adiponectin, TNF-α, IL-6, Leptin, CRP	TESTEX (Hejzi-1, Table S1): 8 [1/0/0/1/0/1/0/2/1/0/1/1]
	Ahmadizad 2014	EG: Overweight ; mode=RT (DUP)<br>CG: Overweight ; mode=CON	EG: 23.4±NR<br>CG: 23.4±NR	EG: n=8 (M=8, F=0)<br>CG: n=3 (M=3, F=0)	Adiponectin, TNF-α, IL-6, Leptin, CRP	TESTEX (Hejzi-1, Table S1): 9 [1/0/0/1/0/1/1/2/1/0/1/1]
		EG: Overweight ; mode=RT (LP)<br>CG: Overweight ; mode=CON	EG: 23.4±NR<br>CG: 23.4±NR	EG: n=8 (M=8, F=0)<br>CG: n=2 (M=2, F=0)	Adiponectin, TNF-α, IL-6, Leptin, CRP	TESTEX (Hejzi-1, Table S1): 9 [1/0/0/1/0/1/1/2/1/0/1/1]
		EG: Overweight ; mode=RT (NP)<br>CG: Overweight ; mode=CON	EG: 23.4±NR<br>CG: 23.4±NR	EG: n=8 (M=8, F=0)<br>CG: n=3 (M=3, F=0)	Adiponectin, TNF-α, IL-6, Leptin, CRP	TESTEX (Hejzi-1, Table S1): 9 [1/0/0/1/0/1/1/2/1/0/1/1]
	Bouchonville 2014	EG: Obese ; mode=AE+RT<br>CG: Obese ; mode=CON	EG: 69.75±NR<br>CG: 69.75±NR	EG: n=26 (M=0, F=26)<br>CG: n=27 (M=0, F=27)	Adiponectin, TNF-α, IL-6, Leptin, CRP	TESTEX (Hejzi-1, Table S1): 12 [1/1/0/1/0/2/1/2/1/1/1/1]
	Nikseresht 2014	EG: Obese ; mode=AE<br>CG: Obese ; mode=CON	EG: 26.6±NR<br>CG: 26.6±NR	EG: n=10 (M=10, F=0)<br>CG: n=6 (M=6, F=0)	Adiponectin, TNF-α, IL-6, Leptin, CRP	TESTEX (Hejzi-1, Table S1): 9 [1/0/0/1/0/1/0/2/1/1/1/1]

Included study (first author, year)	Original study (first author, year)	Athlete type	Age (years)	Sex & sample size	outcome	Quality Assessment Scale (Evidence)
		EG: Obese ; mode=RT<br>CG: Obese ; mode=CON	EG: 26.6±NR<br>CG: 26.6±NR	EG: n=12 (M=12, F=0)<br>CG: n=5 (M=5, F=0)	Adiponectin, TNF-α, IL-6, Leptin, CRP	TESTEX (Hejzi-1, Table S1): 9<br>[1/0/0/1/0/1/0/2/1/1/1/1]
	Abbenhardt 2013	EG: Obese ; mode=AE<br>CG: Obese ; mode=CON	EG: 57.9±NR<br>CG: 57.9±NR	EG: n=117 (M=0, F=117)<br>CG: n=87 (M=0, F=87)	Adiponectin, TNF-α, IL-6, Leptin, CRP	TESTEX (Hejzi-1, Table S1): 10<br>[1/1/0/1/0/1/1/2/1/0/1/1]
	Akbarpour 2013	EG: Obese ; mode=AE<br>CG: Obese ; mode=CON	EG: 22.95±NR<br>CG: 22.95±NR	EG: n=30 (M=30, F=0)<br>CG: n=30 (M=30, F=0)	Adiponectin, TNF-α, IL-6, Leptin, CRP	TESTEX (Hejzi-1, Table S1): 8<br>[1/0/0/1/0/1/0/2/1/0/1/1]
	Mohamed Taha 2013	EG: Obese/Fatty Liver ; mode=RT<br>CG: Obese/Fatty Liver ; mode=CON	EG: 34.59±NR<br>CG: 34.59±NR	EG: n=30 (M=0, F=30)<br>CG: n=30 (M=0, F=30)	Adiponectin, TNF-α, IL-6, Leptin, CRP	TESTEX (Hejzi-1, Table S1): 9<br>[1/0/0/1/0/2/0/2/1/0/1/1]
	Venojärvi 2013	EG: Obese (Finland); mode=NW<br>CG: Obese (Finland); mode=CON	EG: 54.33±NR<br>CG: 54.33±NR	EG: n=39 (M=39, F=0)<br>CG: n=20 (M=20, F=0)	Adiponectin, TNF-α, IL-6, Leptin, CRP	TESTEX (Hejzi-1, Table S1): 8<br>[1/0/0/1/0/1/0/2/1/0/1/1]
		EG: Obese (Finland); mode=RT<br>CG: Obese (Finland); mode=CON	EG: 54.33±NR<br>CG: 54.33±NR	EG: n=36 (M=36, F=0)<br>CG: n=20 (M=20, F=0)	Adiponectin, TNF-α, IL-6, Leptin, CRP	TESTEX (Hejzi-1, Table S1): 8<br>[1/0/0/1/0/1/0/2/1/0/1/1]
	Asad 2012	EG: Overweight ; mode=AE<br>CG: Overweight ; mode=CON	EG: 21.45±NR<br>CG: 21.45±NR	EG: n=12 (M=12, F=0)<br>CG: n=3 (M=3, F=0)	Adiponectin, TNF-α, IL-6, Leptin, CRP	TESTEX (Hejzi-1, Table S1): 8<br>[1/0/0/1/0/1/0/2/1/0/1/1]
		EG: Overweight ; mode=RT<br>CG: Overweight ; mode=CON	EG: 21.45±NR<br>CG: 21.45±NR	EG: n=9 (M=9, F=0)<br>CG: n=3 (M=3, F=0)	Adiponectin, TNF-α, IL-6, Leptin, CRP	TESTEX (Hejzi-1, Table S1): 8<br>[1/0/0/1/0/1/0/2/1/0/1/1]
		EG: Overweight ; mode=AE+RT<br>CG: Overweight ; mode=CON	EG: 21.45±NR<br>CG: 21.45±NR	EG: n=14 (M=14, F=0)<br>CG: n=4 (M=4, F=0)	Adiponectin, TNF-α, IL-6, Leptin, CRP	TESTEX (Hejzi-1, Table S1): 8<br>[1/0/0/1/0/1/0/2/1/0/1/1]
	Johannsen 2012	EG: Obese ; mode=AE<br>CG: Obese ; mode=CON	EG: 57.3±NR<br>CG: 57.3±NR	EG: n=304 (M=0, F=304)<br>CG: n=86 (M=0, F=86)	Adiponectin, TNF-α, IL-6, Leptin, CRP	TESTEX (Hejzi-1, Table S1): 11<br>[1/1/0/1/1/1/0/2/1/1/1/1]
	Phillips 2012	EG: Obese ; mode=RT<br>CG: Obese ; mode=CON	EG: 65.6±NR<br>CG: 65.6±NR	EG: n=11 (M=0, F=11)<br>CG: n=12 (M=0, F=12)	Adiponectin, TNF-α, IL-6, Leptin, CRP	TESTEX (Hejzi-1, Table S1): 11<br>[1/1/0/1/0/2/0/2/1/1/1/1]
	Rashidlamir 2012	EG: Overweight ; mode=AE<br>CG: Overweight ; mode=CON	EG: 38.56±NR<br>CG: 38.56±NR	EG: n=15 (M=15, F=0)<br>CG: n=15 (M=15, F=0)	Adiponectin, TNF-α, IL-6, Leptin, CRP	TESTEX (Hejzi-1, Table S1): 8<br>[1/0/0/1/0/1/0/2/1/0/1/1]
	Arikawa 2011	EG: Obese ; mode=AE<br>CG: Obese ; mode=CON	EG: 25.2±NR<br>CG: 25.2±NR	EG: n=166 (M=0, F=166)<br>CG: n=153 (M=0, F=153)	Adiponectin, TNF-α, IL-6, Leptin, CRP	TESTEX (Hejzi-1, Table S1): 10<br>[1/0/0/1/0/1/1/2/1/1/1/1]
	Eizadi 2011	EG: Obese ; mode=AE<br>CG: Obese ; mode=CON	EG: 39.5±NR<br>CG: 39.5±NR	EG: n=14 (M=14, F=0)<br>CG: n=14 (M=14, F=0)	Adiponectin, TNF-α, IL-6, Leptin, CRP	TESTEX (Hejzi-1, Table S1): 8<br>[1/0/0/1/0/1/0/2/1/0/1/1]
	Friedenreich 2011	EG: Overweight ; mode=AE<br>CG: Overweight ; mode=CON	EG: 60.9±NR<br>CG: 60.9±NR	EG: n=160 (M=0, F=160)<br>CG: n=160 (M=0, F=160)	Adiponectin, TNF-α, IL-6, Leptin, CRP	TESTEX (Hejzi-1, Table S1): 12<br>[1/1/0/1/1/2/1/2/1/0/1/1]
	Varady 2010	EG: Overweight ; mode=R<br>CG: Overweight ; mode=CON	EG: 18.75±NR<br>CG: 18.75±NR	EG: n=12 (M=12, F=0)<br>CG: n=3 (M=3, F=0)	Adiponectin, TNF-α, IL-6, Leptin, CRP	TESTEX (Hejzi-1, Table S1): 7<br>[1/0/0/1/0/1/0/2/1/0/0/1]
		EG: Overweight ; mode=WT<br>CG: Overweight ; mode=CON	EG: 18.75±NR<br>CG: 18.75±NR	EG: n=10 (M=10, F=0)<br>CG: n=3 (M=3, F=0)	Adiponectin, TNF-α, IL-6,	TESTEX (Hejzi-1, Table S1): 7<br>[1/0/0/1/0/1/0/2/1/0/0/1]

Included study (first author, year)	Original study (first author, year)	Athlete type	Age (years)	Sex & sample size	outcome	Quality Assessment Scale (Evidence)
					Leptin, CRP	
		EG: Overweight ; mode=RT+R<br>CG: Overweight ; mode=CON	EG: 18.75±NR<br>CG: 18.75±NR	EG: n=11 (M=11, F=0)<br>CG: n=4 (M=4, F=0)	Adiponectin, TNF-α, IL-6, Leptin, CRP	TESTEX (Hejzi-1, Table S1): 7<br>[1/0/0/1/0/1/0/2/1/0/0/1]
	Arsenault 2009	EG: Obese ; mode=RT<br>CG: Obese ; mode=CON	EG: 57.3±NR<br>CG: 57.3±NR	EG: n=267 (M=0, F=267)<br>CG: n=82 (M=0, F=82)	Adiponectin, TNF-α, IL-6, Leptin, CRP	TESTEX (Hejzi-1, Table S1): 8<br>[1/0/0/1/0/2/0/2/1/0/0/1]
	Konopko-Zubrzycka 2009	EG: Obese ; mode=RT<br>CG: Obese ; mode=CON	EG: 41.9±NR<br>CG: 41.9±NR	EG: n=21 (M=NR, F=NR)<br>CG: n=15 (M=NR, F=NR)	Adiponectin, TNF-α, IL-6, Leptin, CRP	TESTEX (Hejzi-1, Table S1): 7<br>[1/0/0/1/0/1/0/2/1/0/0/1]
	Tjønna 2008	EG: Obese ; mode=RT<br>CG: Obese ; mode=CON	EG: 52.3±NR<br>CG: 52.3±NR	EG: n=8 (M=NR, F=NR)<br>CG: n=4 (M=NR, F=NR)	Adiponectin, TNF-α, IL-6, Leptin, CRP	TESTEX (Hejzi-1, Table S1): 11<br>[1/1/0/1/1/1/0/2/1/1/1/1]
		EG: Obese ; mode=RT<br>CG: Obese ; mode=CON	EG: 52.3±NR<br>CG: 52.3±NR	EG: n=11 (M=NR, F=NR)<br>CG: n=5 (M=NR, F=NR)	Adiponectin, TNF-α, IL-6, Leptin, CRP	TESTEX (Hejzi-1, Table S1): 11<br>[1/1/0/1/1/1/0/2/1/1/1/1]
	Ahmadizad 2007	EG: Obese ; mode=RT<br>CG: Obese ; mode=CON	EG: 40.26±NR<br>CG: 40.26±NR	EG: n=8 (M=8, F=0)<br>CG: n=4 (M=4, F=0)	Adiponectin, TNF-α, IL-6, Leptin, CRP	TESTEX (Hejzi-1, Table S1): 8<br>[1/0/0/1/0/1/0/2/1/0/1/1]
		EG: Obese ; mode=RT<br>CG: Obese ; mode=CON	EG: 40.26±NR<br>CG: 40.26±NR	EG: n=8 (M=8, F=0)<br>CG: n=4 (M=4, F=0)	Adiponectin, TNF-α, IL-6, Leptin, CRP	TESTEX (Hejzi-1, Table S1): 8<br>[1/0/0/1/0/1/0/2/1/0/1/1]
	Olson 2007	EG: Overweight ; mode=RT<br>CG: Overweight ; mode=CON	EG: 39±NR<br>CG: 39±NR	EG: n=16 (M=0, F=16)<br>CG: n=12 (M=0, F=12)	Adiponectin, TNF-α, IL-6, Leptin, CRP	TESTEX (Hejzi-1, Table S1): 9<br>[1/0/0/1/0/2/1/2/1/0/0/1]
	Kondo 2006	EG: Obese ; mode=AE<br>CG: Obese ; mode=CON	EG: 18±NR<br>CG: 18±NR	EG: n=8 (M=0, F=8)<br>CG: n=8 (M=0, F=8)	Adiponectin, TNF-α, IL-6, Leptin, CRP	TESTEX (Hejzi-1, Table S1): 8<br>[1/0/0/1/0/1/0/2/1/0/1/1]
	Fatouros 2005	EG: Overweight ; mode=HRT<br>CG: Overweight ; mode=CON	EG: 70.35±NR<br>CG: 70.35±NR	EG: n=10 (M=10, F=0)<br>CG: n=4 (M=4, F=0)	Adiponectin, TNF-α, IL-6, Leptin, CRP	TESTEX (Hejzi-1, Table S1): 8<br>[1/0/0/1/0/1/0/2/1/0/1/1]
		EG: Overweight ; mode=LRT<br>CG: Overweight ; mode=CON	EG: 70.35±NR<br>CG: 70.35±NR	EG: n=10 (M=10, F=0)<br>CG: n=3 (M=3, F=0)	Adiponectin, TNF-α, IL-6, Leptin, CRP	TESTEX (Hejzi-1, Table S1): 8<br>[1/0/0/1/0/1/0/2/1/0/1/1]
		EG: Overweight ; mode=MRT<br>CG: Overweight ; mode=CON	EG: 70.35±NR<br>CG: 70.35±NR	EG: n=10 (M=10, F=0)<br>CG: n=3 (M=3, F=0)	Adiponectin, TNF-α, IL-6, Leptin, CRP	TESTEX (Hejzi-1, Table S1): 8<br>[1/0/0/1/0/1/0/2/1/0/1/1]
	Hara 2005	EG: Obese ; mode=AE<br>CG: Obese ; mode=CON	EG: 19.2±NR<br>CG: 19.2±NR	EG: n=7 (M=7, F=0)<br>CG: n=4 (M=4, F=0)	Adiponectin, TNF-α, IL-6, Leptin, CRP	TESTEX (Hejzi-1, Table S1): 8<br>[1/1/0/1/0/1/0/2/1/0/0/1]
		EG: Obese ; mode=AE+RT<br>CG: Obese ; mode=CON	EG: 19.2±NR<br>CG: 19.2±NR	EG: n=7 (M=7, F=0)<br>CG: n=3 (M=3, F=0)	Adiponectin, TNF-α, IL-6, Leptin, CRP	TESTEX (Hejzi-1, Table S1): 8<br>[1/1/0/1/0/1/0/2/1/0/0/1]
Hejazi-2 2022 (18)	Baigzadeh 2020	EG: Overwt/T2D; intervention=AE<br>CG: Overwt/T2D; control	EG: 46.0±NR<br>CG: 46.0±NR	EG: n=10 (M=NR, F=NR)<br>CG: n=5 (M=NR, F=NR)	Adiponectin, TNF-α, IL-6, Leptin, CRP	TESTEX (this review, Table S1): 7/15
		EG: Overwt/T2D; intervention=AE+RT<br>CG: Overwt/T2D; control	EG: 46.0±NR<br>CG: 46.0±NR	EG: n=10 (M=NR, F=NR)<br>CG: n=5 (M=NR, F=NR)	Adiponectin, TNF-α, IL-6, Leptin, CRP	TESTEX (this review, Table S1): 7/15

Included study (first author, year)	Original study (first author, year)	Athlete type	Age (years)	Sex & sample size	outcome	Quality Assessment Scale (Evidence)
	Sokolovska 2020	EG: T2D; intervention=IWT<br>CG: T2D; control	EG: 60.8±NR<br>CG: 60.8±NR	EG: n=14 (M=NR, F=NR)<br>CG: n=26 (M=NR, F=NR)	Adiponectin, TNF-α, IL-6, Leptin, CRP	TESTEX (this review, Table S1): 10/15
	Yang 2020	EG: T2D ; mode=AE<br>CG: T2D ; mode=CON	EG: 46.5±NR<br>CG: 46.5±NR	EG: n=30 (M/F NR)<br>CG: n=30 (M/F NR)	Adiponectin	TESTEX (Source NR, Table S1): 7 [1/0/0/1/0/1/0/2/1/0/0/1]
	Annibalini 2017	EG: T2D; intervention=AE+RT<br>CG: T2D; control	EG: 58.5±NR<br>CG: 58.5±NR	EG: n=8 (M=NR, F=NR)<br>CG: n=8 (M=NR, F=NR)	Adiponectin, TNF-α, IL-6, Leptin, CRP	TESTEX (this review, Table S1): 9/15
	Gokulakrishnan 2017	EG: T2D; intervention=AE<br>CG: T2D; control	EG: 44.5±NR<br>CG: 44.5±NR	EG: n=75 (M=NR, F=NR)<br>CG: n=75 (M=NR, F=NR)	Adiponectin, TNF-α, IL-6, Leptin, CRP	TESTEX (this review, Table S1): 7/15
	Zhang 2017	EG: T2D; intervention=AE<br>CG: T2D; control	EG: 46.9±NR<br>CG: 46.9±NR	EG: n=16 (M=NR, F=NR)<br>CG: n=16 (M=NR, F=NR)	Adiponectin, TNF-α, IL-6, Leptin, CRP	TESTEX (this review, Table S1): 8/15
	Doğan Dede 2014	EG: T2D; intervention=AE<br>CG: T2D; control	EG: 54.0±NR<br>CG: 54.0±NR	EG: n=30 (M=NR, F=NR)<br>CG: n=30 (M=NR, F=NR)	Adiponectin, TNF-α, IL-6, Leptin, CRP	TESTEX (this review, Table S1): 10/15
	Mendham 2014	EG: T2D; intervention=AE<br>CG: T2D; control	EG: 48.6±NR<br>CG: 48.6±NR	EG: n=11 (M=NR, F=NR)<br>CG: n=10 (M=NR, F=NR)	Adiponectin, TNF-α, IL-6, Leptin, CRP	TESTEX (this review, Table S1): 12/15
	Ahmadi 2012	EG: Obesity/T2D (Iran); mode=AE<br>CG: Obesity/T2D (Iran); mode=CON	EG: 44.0±NR<br>CG: 44.0±NR	EG: n=15 (M=15, F=0)<br>CG: n=15 (M=15, F=0)	Adiponectin	TESTEX (Source NR, Table S1): 8 [1/0/0/1/0/1/0/2/1/0/1/1]
	Moghadasi 2012	EG: Obesity/T2D; intervention=HIET<br>CG: Obesity/T2D; control	EG: 41.2±NR<br>CG: 41.2±NR	EG: n=8 (M=NR, F=NR)<br>CG: n=4 (M=NR, F=NR)	Adiponectin, TNF-α, IL-6, Leptin, CRP, Adiponectin, TNF-α, IL-6, Leptin, CRP	TESTEX (this review, Table S1): 8/15
	Parsian 2012	EG: T2D (Iran); mode=AE<br>CG: T2D (Iran); mode=CON	EG: 44.0±NR<br>CG: 44.0±NR	EG: n=15 (M=15, F=0)<br>CG: n=15 (M=15, F=0)	Adiponectin	TESTEX (Source NR, Table S1): 8 [1/0/0/1/0/1/0/2/1/0/1/1]
	Jorge 2011	EG: T2D; intervention=AE<br>CG: T2D; control	EG: 53.9±NR<br>CG: 53.9±NR	EG: n=12 (M=NR, F=NR)<br>CG: n=4 (M=NR, F=NR)	Adiponectin, TNF-α, IL-6, Leptin, CRP	TESTEX (this review, Table S1): 9/15
		EG: T2D; intervention=RT<br>CG: T2D; control	EG: 53.9±NR<br>CG: 53.9±NR	EG: n=12 (M=NR, F=NR)<br>CG: n=4 (M=NR, F=NR)	Adiponectin, TNF-α, IL-6, Leptin, CRP	TESTEX (this review, Table S1): 9/15
		EG: T2D; intervention=AE+RT<br>CG: T2D; control	EG: 53.9±NR<br>CG: 53.9±NR	EG: n=12 (M=NR, F=NR)<br>CG: n=4 (M=NR, F=NR)	Adiponectin, TNF-α, IL-6, Leptin, CRP	TESTEX (this review, Table S1): 9/15
	Moghadasi 2011	EG: Obesity/T2D; intervention=LAM<br>CG: Obesity/T2D; control	EG: 42.5±NR<br>CG: 42.5±NR	EG: n=8 (M=NR, F=NR)<br>CG: n=4 (M=NR, F=NR)	Adiponectin, TNF-α, IL-6, Leptin, CRP	TESTEX (this review, Table S1): 8/15
	Wu 2011	EG: T2D; intervention=AE<br>CG: T2D; control	EG: 54.0±NR<br>CG: 54.0±NR	EG: n=68 (M=NR, F=NR)<br>CG: n=67 (M=NR, F=NR)	Adiponectin, TNF-α, IL-6, Leptin, CRP	TESTEX (this review, Table S1): 13/15

Included study (first author, year)	Original study (first author, year)	Athlete type	Age (years)	Sex & sample size	outcome	Quality Assessment Scale (Evidence)
	Balducci 2010	EG: T2D; intervention=LAE<br>CG: T2D; control	EG: 62.1±NR<br>CG: 62.1±NR	EG: n=20 (M=NR, F=NR)<br>CG: n=7 (M=NR, F=NR)	Adiponectin, TNF-α, IL-6, Leptin, CRP	TESTEX (this review, Table S1): 10/15
		EG: T2D; intervention=RT<br>CG: T2D; control	EG: 62.1±NR<br>CG: 62.1±NR	EG: n=20 (M=NR, F=NR)<br>CG: n=7 (M=NR, F=NR)	Adiponectin, TNF-α, IL-6, Leptin, CRP	TESTEX (this review, Table S1): 10/15
		EG: T2D; intervention=AE+RT<br>CG: T2D; control	EG: 62.1±NR<br>CG: 62.1±NR	EG: n=22 (M=NR, F=NR)<br>CG: n=6 (M=NR, F=NR)	Adiponectin, TNF-α, IL-6, Leptin, CRP	TESTEX (this review, Table S1): 10/15
	Ibáñez 2010	EG: Obesity/T2D; intervention=RT<br>CG: Obesity/T2D; control	EG: 50.1±NR<br>CG: 50.1±NR	EG: n=13 (M=NR, F=NR)<br>CG: n=9 (M=NR, F=NR)	Adiponectin, TNF-α, IL-6, Leptin, CRP	TESTEX (this review, Table S1): 8/15
	Ku 2010	EG: T2D; intervention=AE<br>CG: T2D; control	EG: 56.4±NR<br>CG: 56.4±NR	EG: n=15 (M=NR, F=NR)<br>CG: n=8 (M=NR, F=NR)	Adiponectin, TNF-α, IL-6, Leptin, CRP	TESTEX (this review, Table S1): 9/15
		EG: T2D; intervention=RT<br>CG: T2D; control	EG: 56.4±NR<br>CG: 56.4±NR	EG: n=13 (M=NR, F=NR)<br>CG: n=8 (M=NR, F=NR)	Adiponectin, TNF-α, IL-6, Leptin, CRP	TESTEX (this review, Table S1): 9/15
	Okada 2010	EG: T2D; intervention=AE+RT<br>CG: T2D; control	EG: 63.2±NR<br>CG: 63.2±NR	EG: n=21 (M=NR, F=NR)<br>CG: n=17 (M=NR, F=NR)	Adiponectin, TNF-α, IL-6, Leptin, CRP	TESTEX (this review, Table S1): 8/15
	Sixt 2010	EG: T2D/CAD; intervention=AE<br>CG: T2D/CAD; control	EG: NR<br>CG: NR	EG: n=11 (M=NR, F=NR)<br>CG: n=12 (M=NR, F=NR)	Adiponectin, TNF-α, IL-6, Leptin, CRP	TESTEX (this review, Table S1): 7/15
	Brooks 2007	EG: T2D; intervention=RT<br>CG: T2D; control	EG: 66.0±NR<br>CG: 66.0±NR	EG: n=31 (M=NR, F=NR)<br>CG: n=31 (M=NR, F=NR)	Adiponectin, TNF-α, IL-6, Leptin, CRP	TESTEX (this review, Table S1): 10/15
	Corpeleijn 2007	EG: T2D; intervention=AE<br>CG: T2D; control	EG: 56.7±NR<br>CG: 56.7±NR	EG: n=50 (M=NR, F=NR)<br>CG: n=53 (M=NR, F=NR)	Adiponectin, TNF-α, IL-6, Leptin, CRP	TESTEX (this review, Table S1): 9/15
	Kadoglou -1 2007	EG: Overwt/T2D; intervention=AE<br>CG: Overwt/T2D; control	EG: 61.6±NR<br>CG: 61.6±NR	EG: n=30 (M=NR, F=NR)<br>CG: n=30 (M=NR, F=NR)	Adiponectin, TNF-α, IL-6, Leptin, CRP	TESTEX (this review, Table S1): 8/15
	Kadoglou -2 2007	EG: T2D; intervention=AE<br>CG: T2D; control	EG: 58.6±NR<br>CG: 58.6±NR	EG: n=23 (M=NR, F=NR)<br>CG: n=23 (M=NR, F=NR)	Adiponectin, TNF-α, IL-6, Leptin, CRP	TESTEX (this review, Table S1): 9/15
	Rokling-Andersen 2007	EG: T2D; intervention=AE<br>CG: T2D; control	EG: 45.1±NR<br>CG: 45.1±NR	EG: n=48 (M=NR, F=NR)<br>CG: n=37 (M=NR, F=NR)	Adiponectin, TNF-α, IL-6, Leptin, CRP	TESTEX (this review, Table S1): 9/15
	Boudou 2003	EG: T2D; intervention=AE+IE<br>CG: T2D; control	EG: 45.4±NR<br>CG: 45.4±NR	EG: n=8 (M=NR, F=NR)<br>CG: n=8 (M=NR, F=NR)	Adiponectin, TNF-α, IL-6, Leptin, CRP	TESTEX (this review, Table S1): 9/15
Khalafi 2022 (19)	Cho, 2019	EG: Overweight/obese (BMI 26.9±3.9 kg/m²)<br>CG: Overweight/obese (BMI 27.8±3.4 kg/m²)	EG: 38.6±8.2<br>CG: 33.5±5.0	EG: n=NR (overall N=26; Sex=F/M)	CRP, IL-6, TNF-α	RoB checklist (Khalafi 2022, RoB Assessment Check): Total=6/8 [√/√/?/√/√/x/√/√]

Included study (first author, year)	Original study (first author, year)	Athlete type	Age (years)	Sex & sample size	outcome	Quality Assessment Scale (Evidence)
				CG: n=NR (overall N=26; Sex=F/M)		
	Pedersen, 2016	EG: Overweight with stable CAD (BMI 32.03±4.07 kg/m²)<br>CG: Overweight with stable CAD (BMI 31.13±2.41 kg/m²)	EG: 62.3±5.7<br>CG: 63.6±6.8	EG: n=NR (overall N=55; Sex=F/M)<br>CG: n=NR (overall N=55; Sex=F/M)	CRP, IL-6, TNF-α	RoB checklist (Khalafi 2022, RoB Assessment Check): Total=6/8 [√/√/?/x/√/√/√/√]
	Weiss, 2016	EG: Overweight (BMI 27.0±1.5 kg/m²)<br>CG: Overweight (BMI 27.7±1.7 kg/m²)	EG: 56±6<br>CG: 57±5	EG: n=NR (overall N=52; Sex=F/M)<br>CG: n=NR (overall N=52; Sex=F/M)	CRP, IL-6, TNF-α	RoB checklist (Khalafi 2022, RoB Assessment Check): Total=5/8 [?/√/√/x/√/x/√/√]
	Khoo, 2015	EG: Obese (BMI 32.1±2.6 kg/m²)<br>CG: Obese (BMI 32.1±3.0 kg/m²)	EG: 43.3±9.0<br>CG: 41.8±7.2	EG: n=NR (overall N=80; Sex=M)<br>CG: n=NR (overall N=80; Sex=M)	CRP, IL-6, TNF-α	RoB checklist (Khalafi 2022, RoB Assessment Check): Total=8/8 [√/√/√/√/√/√/√/√]
	Bouchonville, 2014	EG: Obese-older adults (BMI 36.9±5.4 kg/m²)<br>CG: Obese-older adults (BMI 37.2±4.5 kg/m²)	EG: 70±4<br>CG: 70±4	EG: n=NR (overall N=80; Sex=F/M)<br>CG: n=NR (overall N=80; Sex=F/M)	CRP, IL-6, TNF-α	RoB checklist (Khalafi 2022, RoB Assessment Check): Total=8/8 [√/√/√/√/√/√/√/√]
	Wiklund, 2014	EG: Overweight/obese (BMI 28.4±2.1 kg/m²)<br>CG: Overweight/obese (BMI 31.3±3.1 kg/m²)	EG: 41.9±7.3<br>CG: 42.2±7.5	EG: n=NR (overall N=83; Sex=F)<br>CG: n=NR (overall N=83; Sex=F)	CRP, IL-6, TNF-α	RoB checklist (Khalafi 2022, RoB Assessment Check): Total=6/8 [√/√/x/√/√/x/√/√]
	Auerbach, 2013	EG: Moderately overweight (BMI 28.1±1.3 kg/m²; group-specific NR)<br>CG: Moderately overweight (BMI 28.1±1.3 kg/m²; group-specific NR)	EG: mean age = NR; age range = 20–40<br>CG: mean age = NR; age range = 20–40	EG: n=NR (overall N=24; Sex=M)<br>CG: n=NR (overall N=24; Sex=M)	CRP, IL-6, TNF-α	RoB checklist (Khalafi 2022, RoB Assessment Check): Total=3/8 [x/√/√/x/x/x/x/√]
	Scott, 2013	EG: Overweight/obese asthma (BMI 32.8±2.5 kg/m²)<br>CG: Overweight/obese asthma (BMI 34.7±4.0 kg/m²)	EG: 42.2±11.5<br>CG: 44.7±14.7	EG: n=NR (overall N=38; Sex=F/M)<br>CG: n=NR (overall N=38; Sex=F/M)	CRP, IL-6, TNF-α	RoB checklist (Khalafi 2022, RoB Assessment Check): Total=4/8 [?/√/√/?/√/x/x/√]
	Christiansen, 2010	EG: Obese (BMI 33.3±4.0 kg/m²)<br>CG: Obese (BMI 35.3±4.0 kg/m²)	EG: 37.2±7.0<br>CG: 35.0±3.0	EG: n=NR (overall N=59; Sex=F/M)<br>CG: n=NR (overall N=59; Sex=F/M)	CRP, IL-6, TNF-α	RoB checklist (Khalafi 2022, RoB Assessment Check): Total=5/8 [√/√/?/x/x/x/√/√]
	Ounis, 2009	EG: Obese adolescents (BMI 30.4±1.8 kg/m²)<br>CG: Obese adolescents (BMI 30.5±2.2 kg/m²)	EG: 13.1±0.9<br>CG: 13.2±0.3	EG: n=NR (overall N=27; Sex=F)<br>CG: n=NR (overall N=27; Sex=F)	CRP, IL-6, TNF-α	RoB checklist (Khalafi 2022, RoB Assessment Check): Total=3/8 [x/√/x/x/x/√/x/√]
	Lambert, 2008	EG: Frail obese elderly persons (BMI 38.5±1.7 kg/m²)<br>CG: Frail obese elderly persons (BMI 37.1±2.8 kg/m²)	EG: 69.6±1.4<br>CG: 68.5±1.4	EG: n=NR (overall N=16; Sex=F/M)<br>CG: n=NR (overall N=16; Sex=F/M)	CRP, IL-6, TNF-α	RoB checklist (Khalafi 2022, RoB Assessment Check): Total=4/8 [?/√/√/x/x/√/x/√]
	Rokling-Andersen, 2007	EG: Overweight with diabetes/CVD risk factors (BMI 28.5±3.3 kg/m²)<br>CG: Overweight with diabetes/CVD risk factors (BMI 29.2±3.8 kg/m²)	EG: 45.1±2.5<br>CG: 45.1±2.5	EG: n=NR (overall N=151; Sex=M)<br>CG: n=NR (overall N=151; Sex=M)	CRP, IL-6, TNF-α	RoB checklist (Khalafi 2022, RoB Assessment Check): Total=4/8 [√/√/?/x/x/√/?/√]
	Giannopoulou, 2005	EG: Postmenopausal women with T2D (BMI 35.9±1.9 kg/m²)<br>CG: Postmenopausal women with T2D (BMI 34.3±1.9 kg/m²)	EG: 55.5±1.7<br>CG: 58.5±1.7	EG: n=NR (overall N=33; Sex=F)<br>CG: n=NR (overall N=33; Sex=F)	CRP, IL-6, TNF-α	RoB checklist (Khalafi 2022, RoB Assessment Check): Total=2/8 [x/√/?/x/x/x/x/√]
	Nicklas, 2004	EG: Older, obese adults (BMI 34.6±5.8 kg/m²)	EG: 69±6	EG: n=NR (overall	CRP, IL-6, TNF-α	RoB checklist (Khalafi 2022, RoB Assessment

Included study (first author, year)	Original study (first author, year)	Athlete type	Age (years)	Sex & sample size	outcome	Quality Assessment Scale (Evidence)
		CG: Older, obese adults (BMI 34.4±4.9 kg/m²)	CG: 68±5	N=202; Sex=F/M)<br>CG: n=NR (overall N=202; Sex=F/M)		Check): Total=5/8 [√/√/√/√/x/x/x/√]
Ding 2022 (20)	Asjari (2021)	EG1: Overweight women<br>EG2: Overweight women<br>CG1: Overweight women<br>CG2: Overweight women	EG1: 35.2±6.57<br>EG2: 36.2±5.32<br>CG1: 32.5±4.67<br>CG2: 35.3±7.39	EG1: n=10 (M=0, F=10)<br>EG2: n=10 (M=0, F=10)<br>CG1: n=10 (M=0, F=10)<br>CG2: n=10 (M=0, F=10)	IL-1β, IL-18	Cochrane RoB (Source NR, RoB figure): High [?/?/−/?/+/+/+]
	Barrón-Cabrera (2020)	EG: Obese adults<br>CG: Obese adults	EG: 33.6±9.6<br>CG: 40±8.1	EG: n=15 (M=5, F=10)<br>CG: n=22 (M=6, F=16)	IL-1β, IL-18	Cochrane RoB (Source NR, RoB figure): High [?/?/−/?/+/+/+]
	Tavvafian (2020)	EG1: Overweight young men<br>EG2: Overweight young men<br>EG3: Overweight young men<br>CG1: Overweight young men<br>CG2: Overweight young men<br>CG3: Overweight young men	EG1: 22.25±1.52<br>EG2: 22.25±1.52<br>EG3: 22.25±1.52<br>CG1: 22.25±1.52<br>CG2: 22.25±1.52<br>CG3: 22.25±1.52	EG1: n=8 (M=8, F=0)<br>EG2: n=8 (M=8, F=0)<br>EG3: n=8 (M=8, F=0)<br>CG1: n=8 (M=8, F=0)<br>CG2: n=8 (M=8, F=0)<br>CG3: n=8 (M=8, F=0)	IL-1β, IL-18	Cochrane RoB (Source NR, RoB figure): High [?/?/−/?/+/+/+]
	Zakavi (2020)	EG1: Obese men<br>EG2: Obese men<br>CG1: Obese men<br>CG2: Obese men	EG1: 34.59±2.24<br>EG2: 34.59±2.24<br>CG1: 34.59±2.24<br>CG2: 34.59±2.24	EG1: n=10 (M=10, F=0)<br>EG2: n=10 (M=10, F=0)<br>CG1: n=10 (M=10, F=0)<br>CG2: n=10 (M=10, F=0)	IL-1β, IL-18	Cochrane RoB (Source NR, RoB figure): High [+/ ? /−/?/+/+/+]
	Liu (2018)	EG: Obese female adolescents<br>CG: Obese female adolescents	EG: 14.6±0.7<br>CG: 14.7±0.8	EG: n=30 (M=0, F=30)<br>CG: n=20 (M=0, F=20)	IL-1β, IL-18	Cochrane RoB (Source NR, RoB figure): High [?/?/−/?/+/+/+]
	Nikseresht (2016)	EG1: Obese men<br>EG2: Obese men<br>CG: Obese men	EG1: 40.4±5.2<br>EG2: 39.6±3.7<br>CG: 38.9±4.1	EG1: n=12 (M=12, F=0)<br>EG2: n=10 (M=10, F=0)<br>CG: n=11 (M=11, F=0)	IL-1β, IL-18	Cochrane RoB (Source NR, RoB figure): High [?/?/−/?/+/+/+]
	Salamat (2016)	EG1: Overweight men<br>EG2: Overweight men<br>EG3: Overweight men<br>CG: Overweight men	EG1: 24.6±2.56<br>EG2: 23.5±3.21<br>EG3: 22.9±3.34<br>CG: 23.8±4.11	EG1: n=11 (M=11, F=0)<br>EG2: n=11 (M=11, F=0)<br>EG3: n=11 (M=11, F=0)<br>CG: n=10 (M=10, F=0)	IL-1β, IL-18	Cochrane RoB (Source NR, RoB figure): High [?/?/−/?/+/+/+]
	Chen (2015)	EG1: Obese teenagers<br>EG2: Obese teenagers<br>EG3: Obese teenagers<br>CG: Obese teenagers	EG1: 14.1±3.1<br>EG2: 13.9±2.2<br>EG3: 14.2±3.8<br>CG: 14.4±3.2	EG1: n=15 (M=15, F=0)<br>EG2: n=15 (M=15, F=0)<br>EG3: n=15 (M=15, F=0)<br>CG: n=15 (M=15, F=0)	IL-1β, IL-18	Cochrane RoB (Source NR, RoB figure): High [?/?/−/?/+/+/+]
	Mendham (2015)	EG: Inactive men<br>CG: Inactive men	EG: 39.5±10.6<br>CG: 36.1±16.1	EG: n=16 (M=16, F=0)<br>CG: n=10 (M=10, F=0)	IL-1β, IL-18	Cochrane RoB (Source NR, RoB figure): High [+/ ? /−/?/+/+/+]
	Prestes (2015)	EG1: Sedentary elderly women<br>EG2: Sedentary elderly women<br>CG: Sedentary elderly women	EG1: 69.20±6.05<br>EG2: 65.52±4.72<br>CG: 66.90±7.56	EG1: n=20 (M=0, F=20)<br>EG2: n=19 (M=0, F=19)<br>CG: n=10 (M=0, F=10)	IL-1β, IL-18	Cochrane RoB (Source NR, RoB figure): High [?/?/−/?/+/+/+]
	Tartibian (2015)	EG: Post-menopausal women<br>CG: Post-menopausal women	EG: 57.1±7.5<br>CG: 57.2±2.2	EG: n=14 (M=0, F=14)<br>CG: n=14 (M=0, F=14)	IL-1β, IL-18	Cochrane RoB (Source NR, RoB figure): High [+/+/−/?/+/+/+]
	Mendham (2014)	EG1: Sedentary middle-aged men<br>EG2: Sedentary middle-aged men<br>CG: Sedentary middle-aged men	EG1: 46.8±6.6<br>EG2: 49.5±6.6<br>CG: 49.2±7.0	EG1: n=10 (M=10, F=0)<br>EG2: n=11 (M=11, F=0)<br>CG: n=11 (M=11, F=0)	IL-1β, IL-18	Cochrane RoB (Source NR, RoB figure): High [?/?/−/?/+/+/+]
	Auerbach (2013)	EG: Overweight men<br>CG: Overweight men	EG: mean age=NR; age range=20–40<br>CG: mean age=NR; age range=20–40	EG: n=12 (M=12, F=0)<br>CG: n=12 (M=12, F=0)	IL-1β, IL-18	Cochrane RoB (Source NR, RoB figure): High [?/?/−/?/+/+/+]
	So (2013)	EG: Elderly participants<br>CG: Elderly participants	EG: 71.6±5.5<br>CG: 68.4±5.8	EG: n=18 (M=6, F=12)<br>CG: n=22 (M=7, F=15)	IL-1β, IL-18	Cochrane RoB (Source NR, RoB figure): High [?/?/−/+/ ? /+/+]
	Christiansen (2010)	EG: Obese subjects<br>CG: Obese subjects	EG: 37.5±8.0<br>CG: 35.6±7.0	EG: n=21 (M=10, F=11)<br>CG: n=19 (M=10, F=9)	IL-1β, IL-18	Cochrane RoB (Source NR, RoB figure): High [?/?/−/+/+/+/?]

Included study (first author, year)	Original study (first author, year)	Athlete type	Age (years)	Sex & sample size	outcome	Quality Assessment Scale (Evidence)
	Huffman (2008)	EG1: Overweight to mild obese participants<br>EG2: Overweight to mild obese participants<br>EG3: Overweight to mild obese participants<br>CG: Overweight to mild obese participants	EG1: mean age=NR; age range=40–69<br>EG2: mean age=NR; age range=40–69<br>EG3: mean age=NR; age range=40–69<br>CG: mean age=NR; age range=40–69	EG1: n=42 (M=22, F=20)<br>EG2: n=49 (M=26, F=23)<br>EG3: n=48 (M=30, F=18)<br>CG: n=50 (M=23, F=27)	IL-1β, IL-18	Cochrane RoB (Source NR, RoB figure): High [+/+/–/?/+/+/+]
Liu 2021 (21)	Cho, 2019	EG: NP adults (South Korea); BMI 28.0±2.6; EX+CR (AE+RE)<br>CG: NP adults (South Korea); BMI 27.8±3.4; CR	EG: 34.5±5.7<br>CG: 33.5±5.0	EG: n=9 (M=NR, F=NR)<br>CG: n=8 (M=NR, F=NR)	CRP, TNF-α, IL-6	RoB (Liu, Fig.5): Some concerns [+/+/+/?/+/?/+]
	Rejeski, 2019	EG: NP adults ; BMI 33.5±3.5 (group-specific NR); EX+CR (AE)<br>CG: NP adults ; BMI 33.5±3.5 (group-specific NR); CR	EG: 66.9±4.7 (group-specific NR)<br>CG: 66.9±4.7 (group-specific NR)	EG: n=79 (M=NR, F=NR)<br>CG: n=68 (M=NR, F=NR)	CRP, TNF-α, IL-6	RoB (Liu, Fig.5): High [+/+/-/+/+/?/+]
		EG: NP adults ; BMI 33.5±3.5 (group-specific NR); EX+CR (RE)<br>CG: NP adults ; BMI 33.5±3.5 (group-specific NR); CR	EG: 66.9±4.7 (group-specific NR)<br>CG: 66.9±4.7 (group-specific NR)	EG: n=75 (M=NR, F=NR)<br>CG: n=68 (M=NR, F=NR)	CRP, TNF-α, IL-6	RoB (Liu, Fig.5): High [+/+/-/+/+/?/+]
	Galedari, 2017	EG: NP adults ; BMI 28.9±1.32; EX+CR (AE)<br>CG: NP adults ; BMI 29.2±2.4; CR	EG: 28.8±6.1<br>CG: 32.6±6.8	EG: n=12 (M=12, F=0)<br>CG: n=8 (M=8, F=0)	CRP, TNF-α, IL-6	RoB (Liu, Fig.5): High [+/+/-/?/+/+/+]
		EG: NP adults ; BMI 29.0±2.9; EX+CR (RE)<br>CG: NP adults ; BMI 29.2±2.4; CR	EG: 31.7±7.7<br>CG: 32.6±6.8	EG: n=10 (M=10, F=0)<br>CG: n=8 (M=8, F=0)	CRP, TNF-α, IL-6	RoB (Liu, Fig.5): High [+/+/-/?/+/+/+]
		EG: NP adults ; BMI 29.6±1.5; EX+CR (HT)<br>CG: NP adults ; BMI 29.2±2.4; CR	EG: 30.8±7.6<br>CG: 32.6±6.8	EG: n=10 (M=10, F=0)<br>CG: n=8 (M=8, F=0)	CRP, TNF-α, IL-6	RoB (Liu, Fig.5): High [+/+/-/?/+/+/+]
	Weiss, 2016	EG: SP adults ; BMI 28.3±1.8; EX+CR (AE)<br>CG: SP adults ; BMI 27.7±1.7; CR	EG: 57±7<br>CG: 57±5	EG: n=19 (M=NR, F=NR)<br>CG: n=17 (M=NR, F=NR)	CRP, TNF-α, IL-6	RoB (Liu, Fig.5): High [-/?/-/+/ ?/+/+]
	Lam, 2015	EG: NP adults ; BMI 27.9±0.6; EX+CR (AE)<br>CG: NP adults ; BMI 27.7±0.5; CR	EG: 37.9±1.8<br>CG: 39.0±2.1	EG: n=8 (M=NR, F=NR)<br>CG: n=8 (M=NR, F=NR)	CRP, TNF-α, IL-6	RoB (Liu, Fig.5): High [?/+/ ?/+/+/+/-]
	Bouchonville, 2014	EG: SP adults ; BMI 37.2±5.4; EX+CR (AE+RE)<br>CG: SP adults ; BMI 37.2±4.5; CR	EG: 70±4<br>CG: 70±4	EG: n=28 (M=NR, F=NR)<br>CG: n=26 (M=NR, F=NR)	CRP, TNF-α, IL-6	RoB (Liu, Fig.5): High [-/+/ ?/+/+/+/+]
	Oh, 2014	EG: NP adults ; BMI 29.2±0.4; EX+CR (AE)<br>CG: NP adults ; BMI 28.5±0.8; CR	EG: 49.1±1.3<br>CG: 53.2±2.1	EG: n=52 (M=52, F=0)<br>CG: n=20 (M=20, F=0)	CRP, TNF-α, IL-6	NR (no quality assessment reported)
	Ryan, 2014	EG: SP adults ; BMI 32±1; EX+CR (AE)<br>CG: SP adults ; BMI 33±1; CR	EG: 60±1<br>CG: 61±1	EG: n=37 (M=0, F=37) measured n=CRP=36<br>CG: n=40 (M=0, F=40) measured n=CRP=40	CRP, TNF-α, IL-6	NR (no quality assessment reported)
	Lakhdar, 2013	EG: NP adults (Tunisia); BMI 32.98±2.17; EX+CR (AE)<br>CG: NP adults (Tunisia); BMI 33.02±1.89; CR	EG: 38.90±4.37<br>CG: 38.90±3.94	EG: n=10 (M=0, F=10)<br>CG: n=10 (M=0, F=10)	CRP, TNF-α, IL-6	RoB (Liu, Fig.5): High [+/ ?/-/+/+/?/+]
	García-Unciti, 2012	EG: SP adults (Spain); BMI 35±3.1; EX+CR (RE)<br>CG: SP adults (Spain); BMI 34.6±3.4; CR	EG: 48.6±6.4<br>CG: 51.4±5.5	EG: n=13 (M=0, F=13)<br>CG: n=12 (M=0, F=12)	CRP, TNF-α, IL-6	RoB (Liu, Fig.5): High [+/ ?/-/+/ ?/+/+]
	Fisher, 2011	EG: SP adults ; BMI 28±1; EX+CR (AE)<br>CG: SP adults ; BMI 28±1; CR	EG: mean age = NR; age range = 20–41<br>CG: mean age = NR; age range = 20–41	EG: n=43 (M=0, F=43)<br>CG: n=29 (M=0, F=29)	CRP, TNF-α, IL-6	RoB (Liu, Fig.5): High [?/+/ -/+/+/+/+]
		EG: SP adults ; BMI 28±1; EX+CR (RE)	EG: mean age = NR;	EG: n=54 (M=0, F=54)	CRP, TNF-α, IL-6	RoB (Liu, Fig.5): High [?/+/ -/+/+/+/+]

Included study (first author, year)	Original study (first author, year)	Athlete type	Age (years)	Sex & sample size	outcome	Quality Assessment Scale (Evidence)
		CG: SP adults ; BMI 28±1; CR	age range = 20–41<br>CG: mean age = NR; age range = 20–41	CG: n=29 (M=0, F=29)		
	Snel, 2011	EG: NP adults (Netherlands); BMI 36.4±1.1; EX+CR (AE)<br>CG: NP adults (Netherlands); BMI 37.9±1.4; CR	EG: 56±2<br>CG: 59±2	EG: n=14 (M=NR, F=NR)<br>CG: n=13 (M=NR, F=NR)	CRP, TNF-α, IL-6	NR (no quality assessment reported)
	Christiansen, 2010	EG: NP adults ; BMI 34.2±3; EX+CR (AE)<br>CG: NP adults ; BMI 35.3±4; CR	EG: 37.5±8<br>CG: 35.6±7	EG: n=21 (M=NR, F=NR)<br>CG: n=19 (M=NR, F=NR)	CRP, TNF-α, IL-6	RoB (Liu, Fig.5): High [+/ ?/-/+/+/-/?]
	Straznicky, 2010	EG: SP adults; BMI 31.8±0.8; EX+CR (AE)<br>CG: SP adults; BMI 32.2±0.9; CR	EG: 54±1<br>CG: 55±1	EG: n=20 (M=20, F=0)<br>CG: n=20 (M=20, F=0)	CRP, TNF-α, IL-6	RoB (Liu, Fig.5): High [+/ ?/-/ ?/-/+/+]
	Brochu, 2009	EG: SP adults ; BMI 32.6±4.9; EX+CR (RE)<br>CG: SP adults ; BMI 32.2±4.6; CR	EG: 57.2±5.0<br>CG: 58.0±4.7	EG: n=36 (M=0, F=36)<br>CG: n=71 (M=0, F=71)	CRP, TNF-α, IL-6	RoB (Liu, Fig.5): High [+/-/-/+/+/?/+]
	Silverman, 2009	EG: SP adults ; BMI 32.1±4.2; EX+CR (AE)<br>CG: SP adults ; BMI 32.6±4.6; CR	EG: 60±5<br>CG: 58±5	EG: n=46 (M=0, F=46)<br>CG: n=40 (M=0, F=40)	CRP, TNF-α, IL-6	NR (no quality assessment reported)
	Giannopoulou, 2005	EG: NP adults ; BMI 33.7±1.9; EX+CR (AE)<br>CG: NP adults ; BMI 34.3±1.9; CR	EG: 57.4±1.7<br>CG: 58.5±1.7	EG: n=11 (M=0, F=11)<br>CG: n=11 (M=0, F=11)	CRP, TNF-α, IL-6	RoB (Liu, Fig.5): High [-/ ?/-/+/ ?/-/+]
	Nicklas, 2004	EG: SP adults ; BMI 33.9±5.6; EX+CR (AE+RE)<br>CG: SP adults ; BMI 34.4±4.9; CR	EG: 68±7<br>CG: 68±5	EG: n=64 (M=NR, F=NR) measured n=CRP=53, IL-6=53, TNF-α=53<br>CG: n=71 (M=NR, F=NR) measured n=CRP=53, IL-6=53, TNF-α=53	CRP, TNF-α, IL-6	RoB (Liu, Fig.5): High [+/+/-/?/+/-/+]
	You, 2004	EG: SP adults ; BMI mean BMI = NR; BMI range = 25–40; EX+CR (AE)<br>CG: SP adults ; BMI mean BMI = NR; BMI range = 25–40; CR	EG: 59±1<br>CG: 57±1	EG: n=17 (M=0, F=17)<br>CG: n=17 (M=0, F=17)	CRP, TNF-α, IL-6	RoB (Liu, Fig.5): High [+/+/-/?/+/?/-]
Lee 2021 (22)	Alberga, 2015	EG: adolescents (BMI 34.7±4.2 kg/m²)<br>CG: adolescents (BMI 34.1±4.9 kg/m²)	EG: mean age = NR; age range = 14–18<br>CG: mean age = NR; age range = 14–18	EG: n=75 (M=NR, F=NR)<br>CG: n=76 (M=NR, F=NR)	CRP, TNF-α, IL-6	RoB2 : Some concerns [+/?/?/+/?/?]
		EG: adolescents (BMI 35.1±4.6 kg/m²)<br>CG: adolescents (BMI 34.1±4.9 kg/m²)	EG: mean age = NR; age range = 14–18<br>CG: mean age = NR; age range = 14–18	EG: n=78 (M=NR, F=NR)<br>CG: n=76 (M=NR, F=NR)	CRP, TNF-α, IL-6	RoB2 : Some concerns [+/?/?/+/?/?]
		EG: adolescents (BMI 34.6±4.2 kg/m²)<br>CG: adolescents (BMI 34.1±4.9 kg/m²)	EG: mean age = NR; age range = 14–18<br>CG: mean age = NR; age range = 14–18	EG: n=75 (M=NR, F=NR)<br>CG: n=76 (M=NR, F=NR)	CRP, TNF-α, IL-6	RoB2 : Some concerns [+/?/?/+/?/?]
	Ben Ounis, 2010	EG: adolescents (BMI 13.1±0.8 kg/m²)<br>CG: adolescents (BMI 13.1±0.8 kg/m²)	EG: 13.1±0.8<br>CG: 13.3±0.6	EG: n=14 (M=NR, F=NR)<br>CG: n=14 (M=NR, F=NR)	CRP, TNF-α, IL-6	RoB2 : Some concerns [?/?/+/+/??]
	Murphy, 2009	EG: children (BMI 27.9±4.8 kg/m²)<br>CG: children (BMI 31.8±5.0 kg/m²)	EG: mean age = 10.21±1.67; age range = NR (reported overall; group-specific NR)	EG: n=23 (M=NR, F=NR)<br>CG: n=12 (M=NR, F=NR)	CRP, TNF-α, IL-6	RoB2 : High [?/?/−/+/?/−]

Included study (first author, year)	Original study (first author, year)	Athlete type	Age (years)	Sex & sample size	outcome	Quality Assessment Scale (Evidence)
			CG: mean age = 10.21±1.67; age range = NR (reported overall; group-specific NR)			
Khalafi 2020 (23)	Bahmanbeglou (2019)	IG: Overweight men with stage 1 hypertension; SDHIIT<br>CG: Overweight men with stage 1 hypertension; CON	IG: 48.0±5.0<br>CG: 47.0±3.0	Total: n=15 (M only)	Adiponectin, Leptin, IL-6, CRP, TNF-α	Criteria checklist (Khalafi 2020, Quality assessment table): 5/8 [✓/✓/✓/×/×/✓/×/✓]
		IG: Overweight men with stage 1 hypertension; LDHIIT<br>CG: Overweight men with stage 1 hypertension; CON	IG: 48.8±5.0<br>CG: 47.0±3.0	Total: n=15 (M only)	Adiponectin, Leptin, IL-6, CRP, TNF-α	Criteria checklist (Khalafi 2020, Quality assessment table): 5/8 [✓/✓/✓/×/×/✓/×/✓]
	Banitalebi (2019)	IG: Type 2 diabetes women; SIT<br>CG: Type 2 diabetes women; CON	IG: 55.36±5.94<br>CG: 55.71±6.40	Total: n=28 (F only)	Adiponectin, Leptin, IL-6, CRP, TNF-α	Criteria checklist (Khalafi 2020, Quality assessment table): 6/8 [✓/✓/✓/✓/×/✓/×/✓]
	Saghebjoo (2019)	IG: Overweight/obese men; HIIT<br>CG: Overweight/obese men; CON	IG: 23.9±1.4<br>CG: 24.4±0.7	Total: n=20 (M only)	Adiponectin, Leptin, IL-6, CRP, TNF-α	Criteria checklist (Khalafi 2020, Quality assessment table): 4/8 [✓/✓/✓/×/×/✓/×/×]
	TaheriChadorneshin (2019)	IG: Overweight women; HIIT<br>CG: Overweight women; CON	IG: 30.03±3.13<br>CG: 30.03±3.13	Total: n=28 (F only)	Adiponectin, Leptin, IL-6, CRP, TNF-α	Criteria checklist (Khalafi 2020, Quality assessment table): 4/8 [×/✓/✓/×/×/✓/×/✓]
	Mora-Rodriguez (2018)	IG: Metabolic syndrome; interval training (HIIT/AIT per study)<br>CG: Metabolic syndrome; CON	IG: 53.5±8.9<br>CG: 53.5±8.9	Total: n=46 (M/F mixed)	Adiponectin, Leptin, IL-6, CRP, TNF-α	Criteria checklist (Khalafi 2020, Quality assessment table): 6/8 [✓/✓/✓/×/×/✓/✓/✓]
	Allen (2017)	IG: Overweight adults; HIIT<br>CG: Overweight adults; CON	IG: 49.2±6.1<br>CG: 49.2±6.1	Total: n=27 (M/F mixed)	Adiponectin, Leptin, IL-6, CRP, TNF-α	Criteria checklist (Khalafi 2020, Quality assessment table): 6/8 [✓/✓/✓/×/×/✓/✓/✓]
		IG: Overweight adults; PIST<br>CG: Overweight adults; CON	IG: 49.2±6.1<br>CG: 49.2±6.1	Total: n=28 (M/F mixed)	Adiponectin, Leptin, IL-6, CRP, TNF-α	Criteria checklist (Khalafi 2020, Quality assessment table): 6/8 [✓/✓/✓/×/×/✓/✓/✓]
	Racil (2016)	IG: Obese females; HIIT<br>CG: Obese females; CON	IG: 14.2±1.2<br>CG: 14.2±1.2	Total: n=47 (F only)	Adiponectin, Leptin, IL-6, CRP, TNF-α	Criteria checklist (Khalafi 2020, Quality assessment table): 3/8 [×/×/✓/×/×/✓/×/✓]
	Almenning （2015）	EG: Polycystic ovary syndrome (PCOS); intervention=HIIT (10 wk; 3 sessions/week; walking/running and/or cycling; HIIT details reported)<br>CG: Polycystic ovary syndrome (PCOS); control=CON (non-intervention)	EG: NR (overall: 27.2±5.5)<br>CG: NR (overall: 27.2±5.5)	Total: n=20 (M=0, F=20)	CRP, adiponectin, leptin, TNF-α, IL-1β, IL-6, IL-8, IL-18, IL-10	Criteria checklist (Khalafi 2020, Quality assessment table): 6/8 [✓/✓/✓/×/×/✓/✓/✓]
	Ahmadizad (2015)	IG: Overweight men; HIIT<br>CG: Overweight men; CON	IG: 25±1<br>CG: 25±1	Total: n=30 (M only)	Adiponectin, Leptin, IL-6, CRP, TNF-α	Criteria checklist (Khalafi 2020, Quality assessment table): 4/8 [✓/×/✓/×/×/✓/×/✓]
		IG: Polycystic ovary syndrome; HIIT<br>CG: Polycystic ovary syndrome; CON	IG: 27.2±5.5<br>CG: 27.2±5.5	Total: n=20 (F only)	Adiponectin, Leptin, IL-6, CRP, TNF-α	Criteria checklist (Khalafi 2020, Quality assessment table): 6/8 [✓/✓/✓/×/×/✓/✓/✓]
	Racil (2015)	IG: Obese females; HIIT (± plyometric per study)<br>CG: Obese females; CON	IG: 16.6±0.9<br>CG: 16.9±1.0	Total: n=42 (F only)	Adiponectin, Leptin, IL-6, CRP, TNF-α	Criteria checklist (Khalafi 2020, Quality assessment table): 3/8 [×/×/✓/×/×/✓/×/✓]
	Nikseresht (2014)	IG: Obese men; AIT (interval training)<br>CG: Obese men; CON	IG: 39.6±3.7<br>CG: 38.9±4.1	Total: n=21 (M only)	Adiponectin, Leptin, IL-6, CRP, TNF-α	Criteria checklist (Khalafi 2020, Quality assessment table): 5/8 [✓/✓/✓/×/×/✓/×/✓]
	Racil (2013)	IG: Obese females; HIIT<br>CG: Obese females; CON	IG: 15.6±0.7<br>CG: 15.9±1.2	Total: n=34 (F only)	Adiponectin, Leptin, IL-6, CRP,	Criteria checklist (Khalafi 2020, Quality assessment table): 4/8 [×/✓/✓/×/×/✓/×/✓]

Included study (first author, year)	Original study (first author, year)	Athlete type	Age (years)	Sex & sample size	outcome	Quality Assessment Scale (Evidence)
					TNF-α	
Sirico 2018 (24)	Racil, 2016	EG: obese female adolescents (P+HIIT)<br>CG: obese female adolescents (none) (arm a: P+HIIT vs none)	EG: 16.5±1.2<br>CG: 16.9±1.0	EG: n=26 (M=0, F=26)<br>CG: n=19 (M=0, F=19)	Adiponectin, Leptin, IL-6, CRP, TNF-α	Cochrane RoB (Meta-analysis, Fig.2): Some concerns [?/?/+/+/+/+/+]
		EG: obese female adolescents (HIIT)<br>CG: obese female adolescents (none) (arm b: HIIT vs none)	EG: 16.6±0.9<br>CG: 16.9±1.0	EG: n=23 (M=0, F=23)<br>CG: n=19 (M=0, F=19)	Adiponectin, Leptin, IL-6, CRP, TNF-α	Cochrane RoB (Meta-analysis, Fig.2): Some concerns [?/?/+/+/+/+/+]
	Monteiro et al., 2015	EG1: Obese adolescents (Brazil; Aerobic: walk/run)<br>EG2: Obese adolescents (Brazil; Concurrent: Res+Aerobic)<br>CG: Obese adolescents (Brazil; non-exercise control)	EG1 (Aerobic): 11.00 ± 1.02<br>EG2 (Concurrent): 11.42 ± 1.34<br>CG: 11.04 ± 1.90	EG1 (Aerobic): n=18 (M=10, F=8)<br>EG2 (Concurrent): n=14 (M=9, F=5)<br>CG: n=16 (M=8, F=8)	IL-6, TNF-α	RoB (Figure 2): High [-/?/+/+/+/-/+]
	Vasconcellos, 2015	EG: obese adolescents (Brazil; recreational soccer program)<br>CG: obese adolescents (Brazil; non-exercise control)	EG: 14.1±1.1<br>CG: 14.8±1.4	EG: n=10 (M=8, F=2)<br>CG: n=10 (M=6, F=4）	Adiponectin, Leptin, IL-6, CRP, TNF-α	Cochrane RoB (Meta-analysis, Fig.2;): Low [+/+/+/+/+/+/+]
	Fazelifar-1, 2013	EG: obese boys (Iran; concurrent training)<br>CG: obese boys (Iran; none)	EG: mean age = NR; age range = 11–13<br>CG: mean age = NR; age range = 11–13	EG: n=12 (M=12, F=0)<br>CG: n=12 (M=12, F=0)	Adiponectin, Leptin, IL-6, CRP, TNF-α	Cochrane RoB (Meta-analysis, Fig.2): High [?/?/+/+/?/+/-]
	Fazelifar-2, 2013	EG: obese young boys (Iran; BMI >28 kg/m²; concurrent training)<br>CG: obese young boys (Iran; BMI >28 kg/m²; none)	EG: mean age = NR; age range = 11–13<br>CG: mean age = NR; age range = 11–13	EG: n=12 (M=12, F=0)<br>CG: n=12 (M=12, F=0)	Adiponectin, Leptin, IL-6, CRP, TNF-α	Cochrane RoB (Meta-analysis, Fig.2): High [?/?/+/+/?/+/-]
	Karacabey, 2009	EG: obese boys (Turkey; BMI 34.9±4.1 kg/m²)<br>CG: obese boys (Turkey; BMI 35.5±3.2 kg/m²)	EG: 11.8±0.5; age range = 10–12<br>CG: 11.2±0.8; age range = 10–12	EG: n=20 (M=20, F=0)<br>CG: n=20 (M=20, F=0)	Adiponectin, Leptin, IL-6, CRP, TNF-α	Cochrane RoB (Meta-analysis, Fig.2): Some concerns [?/?/+/+/+/+/+]
	Kim, 2007	EG: obese male adolescents (Korea; BMI 29.6±0.6 kg/m²)<br>CG: obese male adolescents (Korea; BMI 29.4±0.7 kg/m²)	EG: 17±0.11 (SE; reported overall obese baseline); age range = NR<br>CG: 17±0.11 (SE; reported overall obese baseline); age range = NR	EG: n=14 (M=14, F=0)<br>CG: n=12 (M=12, F=0)	Adiponectin, Leptin, IL-6, CRP, TNF-α	Cochrane RoB (Meta-analysis, Fig.2): Some concerns [?/?/+/+/+/+/+]
García-Hermoso 2016 (25)	Alberga (2015)	EG: adolescents with obesity (BMI ≥85p); Machines; 22 wk, 2 sessions/wk, 20–40 min, 70–85% HRmax<br>CG: adolescents with obesity; no intervention	EG: 15.5±1.4<br>CG: 15.6±1.3	EG: n=74 (M=NR, F=NR)<br>CG: n=74 (M=NR, F=NR)	CRP	Delphi (Source NR, Table 1): 6
	Vasconcellos (2015)	EG: obese adolescents (BMI >2 SD); Soccer; 12 wk, 3 sessions/wk, 60 min, intensity NR<br>CG: no intervention	EG: 14.1±1.3<br>CG: 14.8±1.4	EG: n=10 (M=NR, F=NR)<br>CG: n=10 (M=NR, F=NR)	CRP	Delphi (Source NR, Table 1): 5
	Farpour-Lambert (2009)	EG: pre-pubertal obese children (BMI ≥97p); Multisports; 12 wk, 3 sessions/wk, 60 min, 55–65% HRmax<br>CG: obese children; no intervention	EG: 9.1±1.4<br>CG: 8.8±1.6	EG: n=22 (M=NR, F=NR)<br>CG: n=22 (M=NR, F=NR)	CRP	Delphi (Source NR, Table 1): 6
	Murphy (2009)	EG: overweight children (BMI ≥85p); Dance; 12 wk, 5 sessions/wk, 10–30 min, intensity NR<br>CG: no intervention	EG: mean age = NR; age range = 7–12<br>CG: mean age = NR; age range = 7–12	EG: n=23 (M=NR, F=NR)<br>CG: n=12 (M=NR, F=NR)	CRP	Delphi (Source NR, Table 1): 3

Included study (first author, year)	Original study (first author, year)	Athlete type	Age (years)	Sex & sample size	outcome	Quality Assessment Scale (Evidence)
	Wong (2008)	EG: adolescents with obesity (BMI >25); Machines + multisports; 12 wk, 2 sessions/wk, 45–62 min, 65–85% VO2max<br>CG: no intervention	EG: 13.7±1.1<br>CG: 14.2±1.5	EG: n=12 (M=NR, F=NR)<br>CG: n=12 (M=NR, F=NR)	CRP	Delphi (Source NR, Table 1): 4
	Kim (2007)	EG: obese Korean youth (BMI NR); Skipping rope; 6 wk, 5 sessions/wk, 40 min, intensity NR<br>CG: no intervention	EG: 17.0±0.11<br>CG: 16.8±0.13	EG: n=14 (M=NR, F=NR)<br>CG: n=12 (M=NR, F=NR)	CRP	Delphi (Source NR, Table 1): 4
	Park (2007)	EG: obese girls (BMI ≥95p); Walking; 12 wk, 6 sessions/wk, 30–40 min, 55–75% VO2max<br>CG: no intervention	EG: 14.2±0.5<br>CG: 14.1±0.5	EG: n=22 (M=NR, F=NR)<br>CG: n=22 (M=NR, F=NR)	CRP	Delphi (Source NR, Table 1): 4
	Meyer (2006)	EG: obese children (BMI ≥97p); Multisports; 24 wk, 3 sessions/wk, 60–90 min, intensity NR<br>CG: no intervention	EG: 13.7±2.1<br>CG: 14.7±2.2	EG: n=33 (M=NR, F=NR)<br>CG: n=34 (M=NR, F=NR)	CRP	Delphi (Source NR, Table 1): 4
	Kelly (2004)	EG: overweight/obese children/adolescents (BMI ≥85p); Stationary cycling; 8 wk, 4 sessions/wk, 30 min, 50–60% HRmax<br>CG: no intervention	EG: 10.8±0.67<br>CG: 11.0±0.71	EG: n=9 (M=NR, F=NR)<br>CG: n=10 (M=NR, F=NR)	CRP	Delphi (Source NR, Table 1): 4

Abbreviations: EG, exercise group; IG, intervention group; CG, control group; CON, control/usual care as defined by the trial; NR, not reported; NA, not applicable / no control group; AeT/AE, aerobic training/exercise; RT, resistance training; ST, strength training; COMB, combined aerobic + resistance training; HIIT, high-intensity interval training; SIT, sprint interval training; AIT, aerobic interval training; PA, physical activity; CR, caloric restriction; HRmax, maximal heart rate; VO_2max, maximal oxygen uptake; %1RM, one-repetition maximum percentage; CRP, C-reactive protein; hs-CRP, high-sensitivity C-reactive protein; IL, interleukin; TNF-α, tumor necrosis factor-alpha. Note: "final analysis n=" indicates analyzed sample; "measured n=" indicates biomarker-specific measurement sample; a/b/c denote different arms/comparisons within the same trial.

Quality appraisal information for the included studies was extracted directly from the original systematic reviews/meta-analyses (tables/figures/sections reporting study quality or risk of bias) and recorded in a standardized manner by tool type. Domain-based instruments (e.g., RoB2/Cochrane RoB) were transcribed verbatim as domain judgements and overall ratings (Low/Some concerns/High), together with the original symbol/traffic-light coding (e.g., +, ?, −, N). Score-based instruments (e.g., TESTEX and the PEDro-derived scoring used in some reviews) were recorded as total scores and, where provided by the authors, the corresponding risk categories using the original cut-offs. Checklist-based assessments were documented as item-level fulfilment (✓/×/?) and the total number of criteria met (e.g., x/8), without reclassification unless explicit thresholds were reported. If no quality assessment was reported, this was coded as NR. For Delphi scores reported without the underlying scale range or interpretive thresholds, only the reported score was retained and labelled "scale/threshold NR", with no attempt to map it to risk categories or compare it quantitatively with other tools.

References：

1. Zalagkitis C, Philippou A, Karatzanos E, Metsios GS, Dinas PC. Combined effects of physical activity and diet on chronic inflammation of overweight/obese children and adolescents: A systematic review and meta-analysis. *J Sports Sci*. (2025) 43:2841-2857. doi: 10.1080/02640414.2025.2561349

2. Wang J, Fan S, Wang J. Resistance training enhances metabolic and muscular health and reduces systemic inflammation in middle-aged and older adults with type 2 diabetes: A meta-analysis. *Diabetes Res Clin Pract*. (2025) 229: doi: 10.1016/j.diabres.2025.112941

3. Hernández-Martínez J, Vasquez-Carrasco E, Cid-Calfucura I, Sandoval C, Herrera-Valenzuela T, Núñez-Espinosa C, et al. Effects of concurrent training on biomarkers, morphological variables, and physical performance in people with sarcopenic obesity: A meta-analysis with meta-regression. *Medicina*. (2025) 61: doi: 10.3390/medicina61091697

4. Tan L, Mei JY, Tang RH, Huang D, Qi K, Ossowski Z, et al. Can exercise as a complementary technique manage inflammatory markers in women with breast cancer who are overweight and obese? A systematic review and meta-analysis. *Complement Ther Med*. (2025) 88: doi: 10.1016/j.ctim.2024.103119

5. Silva FM, Duarte-Mendes P, Teixeira AM, Soares CM, Ferreira JP. The effects of combined exercise training on glucose metabolism and inflammatory markers in sedentary adults: A systematic review and meta-analysis. *Sci Rep*. (2024) 14: doi: 10.1038/s41598-024-51832-y

6. Li LY, Li SM, Pang BX, Wei JP, Wang QH. Effects of exercise training on glucose metabolism indicators and inflammatory markers in obese children and adolescents: A meta-analysis. *World J Diabetes*. (2024) 15: doi: 10.4239/wjd.v15.i6.1353

7. Al-Mhanna SB, Batrakoulis A, Norhayati NM, Mohamed M, Drenowatz C, Irekeola AA, et al. Combined aerobic and resistance training improves body composition, alters cardiometabolic risk, and ameliorates cancer-related indicators in breast cancer patients and survivors with overweight/obesity: A systematic review and meta-analysis of randomized controlled trials. *J Sports Sci Med*. (2024) 23:366-395. doi: 10.52082/jssm.2024.366

8. Al-Mhanna SB, Batrakoulis A, Wan Ghazali WS, Mohamed M, Aldayel A, Alhussain MH, et al. Effects of combined aerobic and resistance training on glycemic control, blood pressure, inflammation, cardiorespiratory fitness and quality of life in patients with type 2 diabetes and overweight/obesity: A systematic review and meta-analysis. *PeerJ*. (2024) 12:e17525. doi: 10.7717/peerj.17525

9. Guo YQ, Qian HN, Xin XY, Liu QL. Effects of different exercise modalities on inflammatory markers in the obese and overweight populations: Unraveling the mystery of exercise and inflammation. *Front Physiol*. (2024) 15: doi: 10.3389/fphys.2024.1405094

10. Malandish A, Gulati M. The impacts of exercise interventions on inflammaging markers in overweight/obesity patients with heart failure: A systematic review and meta-analysis of randomized controlled trials. *IJC Heart Vasc*. (2023) 47: doi: 10.1016/j.ijcha.2023.101234

11. Dragoumani K, Troumbis A, Bacopoulou F, Chrousos G. Childhood and adolescent obesity with somatic indicators of stress, inflammation, and dysmetabolism before and after intervention: A meta-analysis. *J Pers Med*. (2023) 13: doi: 10.3390/jpm13091322

12. Tan L, Yan WH, Yang WL, Kamionka A, Lipowski M, Zhao ZJ, et al. Effect of exercise on inflammatory markers in postmenopausal women with overweight and obesity: A systematic review and meta-analysis. *Exp Gerontol*. (2023) 183: doi: 10.1016/j.exger.2023.112310

13. Al-Mhanna SB, Rocha-Rodriguesc S, Mohamed M, Batrakoulis A, Aldhahi MI, Afolabi HA, et al. Effects of combined aerobic exercise and diet on cardiometabolic health in patients with obesity and type 2 diabetes: A systematic review and meta-analysis. *BMC Sports Sci Med Rehabil*. (2023) 15: doi: 10.1186/s13102-023-00766-5

14. Del Rosso S, Baraquet ML, Barale A, Defagó MD, Tortosa F, Perovic NR, et al. Long-term effects of different exercise training modes on cytokines and adipokines in individuals with overweight/obesity and cardiometabolic diseases: A systematic review, meta-analysis, and meta-regression of randomized controlled trials. *Obes Rev*. (2023) 24:e13564. doi: 10.1111/obr.13564

15. Rahimi GRM, Yousefabadi HA, Niyazi A, Rahimi NM, Alikhajeh Y. Effects of lifestyle intervention on inflammatory markers and waist circumference in overweight/obese adults with metabolic syndrome: A systematic review and meta-analysis of randomized controlled trials. *Biol Res Nurs*. (2022) 24:94-105. doi: 10.1177/10998004211044754

16. Zhao HT, Cheng RH, Teng J, Song G, Huang CJ, Yuan S, et al. A meta-analysis of the effects of different training modalities on the inflammatory response in adolescents with obesity. *Int J Environ Res Public Health*. (2022) 19: doi: 10.3390/ijerph192013224

17. Hejazi K, Wong A. Effects of exercise training on inflammatory and cardiometabolic health markers in overweight and obese adults: A systematic review and meta-analysis of randomized controlled trials. *J Sports Med Phys Fitness*. (2023) 63:345-359. doi: 10.23736/s0022-4707.22.14103-4

18. Hejazi K, Rahimi GRM, Rosenkranz SK. Effects of exercise training on inflammatory and cardiometabolic risk biomarkers in patients with type 2 diabetes mellitus: A systematic review and meta-analysis of randomized controlled trials. *Biol Res Nurs*. (2023) 25:250-266. doi: 10.1177/10998004221132841

19. Khalafi M, Symonds ME, Akbari A. The impact of exercise training versus caloric restriction on inflammation markers: A systemic review and meta-analysis. *Crit Rev Food Sci Nutr*. (2022) 62:4226-4241. doi: 10.1080/10408398.2021.1873732

20. Ding YJ, Xu X. Anti-inflammatory effect of exercise training through reducing inflammasome activation-related inflammatory cytokine levels in overweight/obese populations: A systematic review and meta-analysis. *COMPLEMENTARY THERAPIES IN CLINICAL PRACTICE*. (2022) 49: doi: 10.1016/j.ctcp.2022.101656

21. Liu Y, Hong F, Reddy VR, Arifullah A, Ji L, Zhang Y, et al. Calorie restriction with exercise intervention improves inflammatory response in overweight and obese adults: A systematic review and meta-analysis. *Front Physiol*. (2021) 12:754731. doi: 10.3389/fphys.2021.754731

22. Lee J. Influences of exercise interventions on overweight and obesity in children and adolescents. *Public Health Nurs*. (2021) 38:502-516. doi: 10.1111/phn.12862

23. Khalafi M, Symonds ME. The impact of high-intensity interval training on inflammatory markers in metabolic disorders: A meta-analysis. *Scand J Med Sci Sports*. (2020) 30:2020-2036. doi: 10.1111/sms.13754

24. Sirico F, Bianco A, D'Alicandro G, Castaldo C, Montagnani S, Spera R, et al. Effects of physical exercise on adiponectin, leptin, and inflammatory markers in childhood obesity: Systematic review and meta-analysis. *Child Obes*. (2018) 14:207-217. doi: 10.1089/chi.2017.0269

25. García Hermoso A, Sánchez–López M, Escalante Y, Saavedra JM, Martinez Vizcaino V. Exercise-based interventions and c-reactive protein in overweight and obese youths: A meta-analysis of randomized controlled trials. *Pediatr Res*. (2016) 79:522-527. doi: 10.1038/pr.2015.274
