# Supplementary material for: Exercise-induced changes in systemic inflammatory biomarkers in overweight and obese populations: a bibliometric analysis and umbrella review of meta-analyses
Source: Front Immunol. 2026 May 20;17:1838118. doi: 10.3389/fimmu.2026.1838118 (PMC13230183; doi:10.3389/fimmu.2026.1838118)
Supplement: Supplementary file 4 [file SupplementaryFile4.pdf]

Supplementary File 4

Contents

1. AMSTAR 2 and ROBIS Comprehensive Assessment Form 1

1.1. AMSTER (A Measure Tool to Assess Systematic Reviews) - 2 2

1.2. ROBIS:Tool to assess risk of bias in systematic reviews 3

2. Quality Assessment of Included Meta-Analyses 5

2.1. Detailed Risk of Bias Assessment for the Systematic Review by Constantine Zalagkitis et al. (2025) 5

2.2. Detailed Risk of Bias Assessment for the Systematic Review by Jingwen Wang et al. (2025) 7

2.3. Detailed Risk of Bias Assessment for the Systematic Review by Jordan Hernandez - Martinez et al. (2025) 9

2.4. Detailed Risk of Bias Assessment for the Systematic Review by Liang Tan et al. (2025) 11

2.5. Detailed Risk of Bias Assessment for the Systematic Review by Fernanda M. Silva et al. (2024) 13

2.6. Detailed Risk of Bias Assessment for the Systematic Review by Le-Yang Li et al. (2022) 15

2.7. Detailed Risk of Bias Assessment for the Systematic Review by Sameer Badri Al-Mhanna et al.-1 (2024) 17

2.8. Detailed Risk of Bias Assessment for the Systematic Review by Sameer Badri Al-Mhanna et al.-2 (2024) 19

2.9. Detailed Risk of Bias Assessment for the Systematic Review by Yongqing Guo et al. (2024) 21

2.10. Detailed Risk of Bias Assessment for the Systematic Review by Abbas Malandish et al. (2023) 23

2.11. Detailed Risk of Bias Assessment for the Systematic Review by Konstantina Dragoumani et al. (2023) 25

2.12. Detailed Risk of Bias Assessment for the Systematic Review by Liang Tan et al. (2023) 27
2.13. Detailed Risk of Bias Assessment for the Systematic Review by Sameer Badri Al-Mhanna et al. (2023) 29
2.14. Detailed Risk of Bias Assessment for the Systematic Review by Sebastian Del Rosso et al. (2023) 31
2.15. Detailed Risk of Bias Assessment for the Systematic Review by Gholam Rasul Mohammad Rahimi et al. (2022) 33
2.16. Detailed Risk of Bias Assessment for the Systematic Review by Haotian Zhao et al. (2022) 35
2.17. Detailed Risk of Bias Assessment for the Systematic Review by Keyvan Hejazi et al. (2022) I 37
2.18. Detailed Risk of Bias Assessment for the Systematic Review by Keyvan Hejazi et al. (2022) II 39
2.19. Detailed Risk of Bias Assessment for the Systematic Review by Mousa Khalafi et al. (2022) 41
2.20. Detailed Risk of Bias Assessment for the Systematic Review by Yijian Ding et al. (2022) 43
2.21. Detailed Risk of Bias Assessment for the Systematic Review by Yubo Liu et al. (2021) 45
2.22. Detailed Risk of Bias Assessment for the Systematic Review by Junga Lee et al. (2021) 47
2.23. Detailed Risk of Bias Assessment for the Systematic Review by Mousa Khalafi et al. (2020) 49
2.24. Detailed Risk of Bias Assessment for the Systematic Review by Felice Sirico et al. (2018) 51
2.25. Detailed Risk of Bias Assessment for the Systematic Review by Antonio García - Hermoso et al. (2016) 53

1. AMSTAR 2 and ROBIS Comprehensive Assessment Form

Review	AMSTAR 2																	ROBIS					Overall Quality
	Q1	Q2	Q3	Q4	Q5	Q6	Q7	Q8	Q9	Q10	Q11	Q12	Q13	Q14	Q15	Q16	Rating	D1	D2	D3	D4	RISK	
Zalagkitis 2025 [1]	Y	Y	Y	PY	Y	Y	N	Y	Y	N	Y	Y	Y	Y	Y	Y	Low	✔	✔	✔	✔	Low	Moderate
Wang 2025 [2]	Y	Y	N	PY	Y	Y	Y	Y	Y	N	Y	N	Y	Y	Y	N	Moderate	✔	✔	✔	✔	Low	High
Hernandez-Martinez 2025 [3]	Y	Y	Y	Y	Y	N	N	Y	Y	N	Y	Y	Y	Y	Y	Y	Low	✔	?	✔	X	High	Low
Tan 2025 [4]	Y	Y	Y	PY	Y	Y	N	Y	Y	N	Y	Y	Y	Y	Y	Y	Low	✔	X	✔	✔	Low	Low
Silva 2024 [5]	Y	Y	Y	PY	Y	Y	N	Y	Y	N	Y	Y	Y	Y	Y	Y	Low	✔	✔	✔	✔	Low	Moderate
Li 2024 [6]	Y	Y	N	PY	Y	Y	N	Y	Y	N	Y	N	N	Y	Y	PY	Critically low	✔	✔	✔	✔	Low	Low
Al-Mhanna-1 2024 [7]	Y	N	Y	PY	N	N	N	Y	Y	N	Y	N	Y	Y	N	Y	Critically low	✔	✔	✔	✔	Low	Low
AL-Mhanna-2 2024 [8]	Y	N	PY	PY	Y	Y	Y	Y	Y	N	Y	Y	Y	Y	Y	Y	Low	✔	X	X	X	High	Low
Guo, 2024 [9]	Y	N	Y	PY	Y	N	N	Y	Y	N	Y	N	N	Y	N	Y	Critically low	X	✔	✔	✔	High	Low
Malandish 2023 [10]	Y	Y	Y	PY	N	N	N	Y	Y	N	Y	N	Y	Y	Y	Y	Low	✔	X	✔	✔	High	Low
Dragoumani 2023 [11]	Y	Y	PY	PY	Y	Y	N	Y	N	N	Y	Y	Y	Y	Y	Y	Low	✔	X	X	X	High	Low
Tan 2023 [12]	Y	Y	PY	PY	Y	Y	N	Y	Y	N	Y	Y	Y	Y	Y	Y	Low	✔	✔	✔	✔	Low	Moderate
Al-Mhanna 2023 [13]	Y	Y	Y	PY	Y	Y	N	Y	Y	N	Y	Y	Y	Y	Y	Y	Low	✔	✔	✔	✔	Low	Moderate
Del Rosso 2023 [14]	Y	Y	Y	PY	Y	Y	Y	Y	Y	N	Y	Y	Y	Y	Y	Y	Moderate	✔	✔	✔	✔	Low	High
Rahimi 2022 [15]	Y	N	Y	PY	Y	Y	N	Y	Y	N	Y	N	N	N	N	Y	Critically low	✔	✔	✔	X	High	Low
Zhao 2022 [16]	Y	N	Y	PY	Y	Y	N	Y	Y	N	Y	N	Y	Y	Y	Y	Critically low	✔	X	✔	✔	High	Low
Hejazi-1 2022 [17]	Y	N	Y	PY	Y	Y	N	Y	Y	N	Y	Y	N	Y	N	Y	Critically low	✔	✔	✔	X	High	Low
Hejazi-2 2022 [18]	Y	Y	Y	PY	Y	Y	N	Y	Y	N	Y	Y	N	Y	N	Y	Critically low	✔	✔	✔	X	High	Low
Khalafi 2022 [19]	Y	N	Y	PY	Y	Y	N	Y	N	N	Y	N	Y	Y	Y	Y	Critically low	✔	X	X	✔	High	Low
Ding 2022 [20]	Y	Y	Y	PY	Y	Y	N	Y	Y	N	Y	N	Y	Y	Y	Y	Low	✔	✔	✔	✔	Low	Moderate
Liu 2021 [21]	Y	N	Y	PY	Y	Y	N	Y	Y	Y	N	N	Y	Y	N	Y	Critically low	✔	✔	✔	✔	low	Low
Lee 2021 [22]	Y	N	Y	PY	N	PY	PY	Y	N	N	Y	N	N	N	Y	Y	Critically low	✔	?	X	?	High	Low
Khalafi 2020 [23]	Y	N	Y	Y	Y	Y	N	Y	Y	N	Y	N	N	Y	Y	Y	Critically low	✔	X	X	X	High	Low
Sirico 2018 [24]	Y	N	Y	PY	Y	Y	N	Y	Y	N	Y	Y	N	N	N	Y	Critically low	✔	✔	✔	✔	Low	Low
García-Hermoso 2016 [25]	Y	N	Y	PY	Y	Y	N	Y	Y	N	Y	Y	Y	Y	Y	Y	Critically low	✔	✔	✔	✔	Low	Low

1.1. AMSTER (A Measure Tool to Assess Systematic Reviews) - 2

Question	Items	Evaluation options
Q1	Did the research questions and inclusion criteria for the review include the components of PICO?	Yes<br>No
Q2	Did the report of the review contain an explicit statement that the review methods were established prior to the conduct of the review and did the report justify any significant deviations from the protocol?	Yes<br>Partial Yes<br>No
Q3	Did the review authors explain their selection of the study designs for inclusion in the review?	Yes<br>No
Q4	Did the review authors use a comprehensive literature search strategy?	Yes<br>Partial Yes<br>No
Q5	Did the review authors perform study selection in duplicate?	Yes<br>No
Q6	Did the review authors perform data extraction in duplicate?	Yes<br>No
Q7	Did the review authors provide a list of excluded studies and justify the exclusions?	Yes<br>Partial YeS<br>No
Q8	Did the review authors describe the included studies in adequate detail?	Yes<br>Partial Yes<br>No
Q9	Did the review authors use a satisfactory technique for assessing the risk of bias (RoB) in individual studies that were included in the review?	Yes<br>Partial Yes<br>No<br>Includes only NRSI or RCTs
Q10	Did the review authors report on the sources of funding for the studies included in the review?	Yes<br>No
Q11	If meta-analysis was performed did the review authors use appropriate methods for statistical combination of results?	Yes<br>No<br>No meta-analysis conducted
Q12	If meta-analysis was performed, did the review authors assess the potential impact of RoB in individual studies on the results of the meta-analysis or other evidence synthesis?	Yes<br>No<br>No meta-analysis conducted
Q13	Did the review authors account for RoB in individual studies when interpreting/ discussing the results of the review?	Yes<br>No
Q14	Did the review authors provide a satisfactory explanation for, and discussion of, any heterogeneity observed in the results of the review?	Yes<br>No
Q15	If they performed quantitative synthesis did the review authors carry out an adequate investigation of publication bias (small study bias) and discuss its likely impact on the results of the review?	Yes<br>No<br>No meta-analysis conducted
Q16	Did the review authors report any potential sources of conflict of interest, including any funding they received for conducting the review?	Yes<br>No

1.2. ROBIS:Tool to assess risk of bias in systematic reviews

DOMAIN 1: STUDY ELIGIBILITY CRITERIA	
Describe the study eligibility criteria, any restrictions on eligibility and whether there was evidence that objectives and eligibility criteria were pre-specified:	
1.1Did the review adhere to pre-defined objectives and eligibility criteria?	Y/PY/PN/N/NI
1.2 Were the eligibility criteria appropriate for the review question?	Y/PY/PN/N/NI
1.3 Were eligibility criteria unambiguous?	Y/PY/PN/N/NI
1.4 Were any restrictions in eligibility criteria based on study characteristics appropriate (e.g. date, sample size, study quality, outcomes measured)?	Y/PY/PN/N/NI
1.5 Were any restrictions in eligibility criteria based on sources of information appropriate (e.g. publication status or format, language, availability of data)?	Y/PY/PN/N/NI
Concerns regarding specification of study eligibility criteria	LOW/HIGH/UNCLEAR

DOMAIN 2: IDENTIFICATION AND SELECTION OF STUDIES	
Describe methods of study identification and selection (e.g. number of reviewers involved):	
2.1 Did the search include an appropriate range of databases/electronic sources for published and unpublished reports?	Y/PY/PN/N/NI
2.2 Were methods additional to database searching used to identify relevant reports?	Y/PY/PN/N/NI
2.3 Were the terms and structure of the search strategy likely to retrieve as many eligible studies as possible?	Y/PY/PN/N/NI
2.4 Were restrictions based on date, publication format, or language appropriate?	Y/PY/PN/N/NI
2.5 Were efforts made to minimise error in selection of studies?	Y/PY/PN/N/NI
Concerns regarding methods used to identify and/or select studies	LOW/HIGH/UNCLEAR

DOMAIN 3: DATA COLLECTION AND STUDY APPRAISAL	
Describe methods of data collection, what data were extracted from studies or collected through other means, how risk of bias was assessed (e.g. number of reviewers involved) and the tool used to assess risk of bias:	
3.1 Were efforts made to minimise error in data collection?	Y/PY/PN/N/NI
2.2 Were methods additional to database searching used to identify relevant reports?	Y/PY/PN/N/NI
3.3 Were all relevant study results collected for use in the synthesis?	Y/PY/PN/N/NI
3.4 Was risk of bias (or methodological quality) formally assessed using appropriate criteria?	Y/PY/PN/N/NI
3.5 Were efforts made to minimise error in risk of bias assessment?	Y/PY/PN/N/NI
Concerns regarding methods used to collect data and appraise studie	LOW/HIGH/UNCLEAR

DOMAIN 4: SYNTHESIS AND FINDINGS	
Describe synthesis methods:	
4.1 Did the synthesis include all studies that it should?	Y/PY/PN/N/NI
4.2 Were all pre-defined analyses reported or departures explained?	Y/PY/PN/N/NI
4.3 Was the synthesis appropriate given the nature and similarity in the research questions, study designs and outcomes across included studies?	Y/PY/PN/N/NI
4.4 Was between-study variation (heterogeneity) minimal or addressed in the synthesis?	Y/PY/PN/N/NI
4.5 Were the findings robust, e.g. as demonstrated through funnel plot or sensitivity analyses?	Y/PY/PN/N/NI
4.6 Were biases in primary studies minimal or addressed in the synthesis?	Y/PY/PN/N/NI
Concerns regarding the synthesis and findings	LOW/HIGH/UNCLEAR

2. Quality Assessment of Included Meta-Analyses

2.1. Detailed Risk of Bias Assessment for the Systematic Review by Constantine Zalagkitis et al. (2025)

Phase 1: Assessing relevance

Category	Target question	Review being assessed
Patients / Population(s)	Healthy overweight/obese children and adolescents (BMI >85th percentile, aged 0-18 years).	Healthy overweight/obese children and adolescents (BMI >85th percentile, aged 3-18 years).
Intervention(s)	Combined physical activity/exercise (measurable type / frequency / intensity) and diet / nutrition.	Combined physical activity/exercise (aerobic/resistance/combined) and diet (calorie restriction/Mediterranean diet/etc., measurable parameters).
Comparator(s)	Non-exercise control or standard care.	Non-intervention control group or baseline values of the experimental group (for SGDS).
Outcome(s)	Serum/plasma inflammatory markers (CRP, TNF-α, IL-6, IL-8, IL-10, IL-1β).	Serum/plasma inflammatory markers (CRP, TNF-α, IL-6, IL-8, IL-10, IL-1β).

Phase 2: Identifying concerns with the review process

Domain	Signaling Questions	Response	Evidence / Rationale	Risk Bias Assessment
DOMAIN 1: STUDY ELIGIBILITY CRITERIA	1.1 Did the review adhere to pre-defined objectives and eligibility criteria?	Y	The review was registered on INPLASY (INPLASY2024110126) with a published protocol. Eligibility criteria were pre-specified before search (detailed in Methods section) and consistently applied to all identified studies.	LOW
	1.2 Were the eligibility criteria appropriate for the review question?	Y	Criteria aligned with the research question (assessing combined interventions on chronic inflammation in target population): clear definitions for population (BMI >85th percentile, 0-18 years), intervention (combined physical activity + diet, ≥2 weeks), comparator (non-intervention/baseline), and outcomes (inflammatory biomarkers).	
	1.3 Were eligibility criteria unambiguous?	Y	Criteria aligned with the research question	
	1.4 Were all restrictions in eligibility criteria based on study characteristics appropriate?	Y	Explicit criteria for population, intervention , study design , and excluded studies . However, no explicit clinical diagnostic criteria for "healthy" status beyond "absence of disease/infirmity" were provided.	
	1.5 Were any restrictions in eligibility criteria based on sources of information appropriate?	Y	No inappropriate restrictions on publication time, sample size, or study quality.	
DOMAIN 2: IDENTIFICATION AND SELECTION OF STUDIES	2.1 Did the search include an appropriate range of databases/electronic sources?	Y	Searched 4 core databases (PubMed, Ovid, SportDiscus, Web of Science)	LOW
	2.2 Were methods additional to database searching used to identify relevant reports?	Y	Supplementary search methods included screening reference lists of eligible studies (identified 9 additional studies) and handsearching conference abstracts.	
	2.3 Were the terms and structure of the search strategy likely to retrieve as many eligible studies as possible?	Y	Detailed search algorithms (provided in Supplement), with Boolean combinations to maximize retrieval.	
	2.4 Were restrictions based on date, publication format, or language appropriate?	Y	No restrictions on publication date (searched up to February 2025) or format (included full-text and abstracts). Non-English studies were translated, ensuring no language bias.	
	2.5 Were efforts made to minimise error in selection of studies?	Y	Two independent investigators screened titles/abstracts and full-texts. Disagreements were resolved by a third investigator, per standard operating procedures to reduce selection bias.	
DOMAIN 3: DATA COLLECTION AND STUDY APPRAISAL	3.1 Were efforts made to minimise error in data collection?	Y	Two independent investigators extracted data using a pre-defined data extraction form. Disagreements were resolved by a third investigator, ensuring consistency.	LOW
	3.2 Were sufficient study characteristics available for interpretation?	Y	Supplement Table S2 provides detailed characteristics of included studies: study design, participant demographics, intervention details, diet content, and main outcomes.	
	3.3 Were all relevant study results collected for use in the synthesis?	Y	Extracted data for all pre-specified outcomes across all eligible studies. For meta-analysis, continuous data were extracted; narrative synthesis was used for studies with missing meta-analysis data.	
	3.4 Was risk of bias (or methodological quality) formally assessed using appropriate criteria?	Y	RCTs were assessed with the Cochrane Risk of Bias Tool, and non-RCTs with the RTI-IB Tool.	
	3.5 Were efforts made to minimise error in risk of bias assessment?	Y	Two independent investigators conducted bias assessments. Disagreements were resolved by a third investigator, reducing assessor bias.	

DOMAIN 4: SYNTHESIS AND FINDINGS	4.1 Did the synthesis include all studies that it should?	Y	All 50 eligible studies were included in either meta-analysis or narrative synthesis. No arbitrary exclusion of eligible studies.	LOW
	4.2 Were all pre-defined analyses reported or departures explained?	PY	Pre-defined analyses were reported. A departure was explained in "Deviations from the published protocol". Cumulative Meta-analysis was used but not pre-specified, with no explicit explanation.	
	4.3 Was the synthesis appropriate given the nature of studies?	Y	Random-effects model was used for meta-analysis.	
	4.4 Was between-study variation (heterogeneity) minimal or addressed in the synthesis?	Y	Heterogeneity was quantified using I^2. Subgroup analyses and Meta-regression were conducted to explore heterogeneity sources, per standard methods.	
	4.5 Were the findings robust (e.g., funnel plot/sensitivity analyses)?	Y	Funnel plots were used to assess publication bias . Sensitivity analyses were not explicitly reported but subgroup analyses across key variables demonstrated consistent direction of effects, supporting robustness.	
	4.6 Were biases in primary studies minimal or addressed in the synthesis?	Y	Primary study bias was assessed, studies with high risk of bias were retained but their impact was considered in the discussion.	

Phase 3: Judging risk of bias

Summarize the concerns identified during the Phase 2 assessment:

Domain	Concern	Evidence / Rationale
1. Eligibility Criteria	LOW	All signaling questions answered "Yes" or "Probably Yes"; criteria are pre-defined, appropriate, and unambiguous.
2. Identification/Selection	LOW	Comprehensive search strategy, supplementary methods, and error-minimization steps ensure minimal bias.
3. Data Collection/Appraisal	LOW	Rigorous data extraction and bias assessment procedures with independent investigators and third-party resolution.
4. Synthesis and Findings	LOW	Cumulative Meta-analysis was used without explicit explanation of departure from pre-defined protocol; no impact on overall bias risk.

RISK OF BIAS IN THE REVIEW

Signaling Question	Response	Evidence / Rationale
A. Did the interpretation of findings address all of the concerns identified in Domains 1 to 4?	Y	The discussion section addresses limitations and strengths of the review process. No unaddressed concerns from Phase 2.
B. Was the relevance of identified studies to the review's research question appropriately considered?	Y	All included studies directly address the combined effects of physical activity and diet on inflammatory biomarkers in overweight/obese children/adolescents. The discussion compares findings.
C. Did the reviewers avoid emphasizing results on the basis of their statistical significance?	Y	Results are presented objectively: significant effects (CRP, IL-6, IL-8, IL-1β) and non-significant effects (TNF-α, IL-10) are equally reported. Conclusions are based on GRADE certainty of evidence and effect magnitude, not just statistical significance.

Y=YES, PY=PROBABLY YES, PN=PROBABLY NO, N=NO, NI=NO INFORMATION

2.2. Detailed Risk of Bias Assessment for the Systematic Review by Jingwen Wang et al. (2025)

Phase 1: Assessing relevance

Category	Target question	Review being assessed
Patients / Population(s)	Middle-aged and older adults (≥50 years) with type 2 diabetes (T2DM) and overweight/obesity (BMI ≥25 kg/m²).	Middle-aged and older adults (mean age 57.8±8.4 years) with T2DM, overweight/obesity (mean BMI 30.9±3.8 kg/m²), 55.8% women.
Intervention(s)	Resistance training (RT) alone (structured, measurable intensity/frequency/duration); excluding combined aerobic/dietary interventions.	Resistance training alone (equipment/free weights/elastic bands; 1-4 sets, 8-12 reps, 40%-85% 1RM; progressive overload); no combined interventions.
Comparator(s)	Non-exercise control (usual care/wait-list) or baseline values (for single-group designs).	Non-exercise control group (n=966) or baseline values; no active exercise comparators.
Outcome(s)	Metabolic markers (fasting insulin, HOMA-IR, fasting glucose, HbA1c, BMI), muscle markers (muscle mass, upper/lower-body strength), inflammatory markers (CRP, TNF-α, IL-6).	All pre-defined outcomes reported; metabolic (MD: fasting insulin -1.35 μIU/mL, HbA1c -0.55%), muscle (MD: muscle mass +0.89 kg), inflammatory (SMD: CRP -0.80) markers.

Phase 2: Identifying concerns with the review process

Domain	Signaling Questions	Response	Evidence / Rationale	Risk Bias Assessment
DOMAIN 1: STUDY ELIGIBILITY CRITERIA	1.1 Did the review adhere to pre-defined objectives and eligibility criteria?	Y	Protocol registered in PROSPERO (CRD42024617992). PICOS clearly stated in Methods.	LOW
	1.2 Were the eligibility criteria appropriate for the review question?	Y	Criteria include: RCTs, age ≥50, BMI ≥25, T2D, resistance training alone, non-exercise control. Appropriate for research question.	
	1.3 Were eligibility criteria unambiguous?	Y	Clear definitions for participants, intervention, comparators, outcomes.	
	1.4 Were all restrictions in eligibility criteria based on study characteristics appropriate?	PY	Restriction to RCTs is appropriate for causal inference. Age ≥50 and BMI ≥25 are justified for the target population.	
	1.5 Were any restrictions in eligibility criteria based on sources of information appropriate?	PY	Language restriction (English only) may introduce language bias and limit comprehensiveness.	
DOMAIN 2: IDENTIFICATION AND SELECTION OF STUDIES	2.1 Did the search include an appropriate range of databases/electronic sources?	Y	PubMed, Web of Science, Scopus, Cochrane CENTRAL. Supplementary searching of Google Scholar and reference lists.	LOW
	2.2 Were methods additional to database searching used to identify relevant reports?	PY	Grey literature searched (Google Scholar), reference lists checked. No mention of trial registries beyond those in databases.	
	2.3 Were the terms and structure of the search strategy likely to retrieve as many eligible studies as possible?	Y	Detailed search strategy provided in Supplementary Table S1 with MeSH and keywords.	
	2.4 Were restrictions based on date, publication format, or language appropriate?	PY	No date restrictions. Language restricted to English	
	2.5 Were efforts made to minimise error in selection of studies?	Y	Two reviewers independently screened titles/abstracts and full texts. Discrepancies resolved by discussion or third reviewer.	
DOMAIN 3: DATA COLLECTION AND STUDY APPRAISAL	3.1 Were efforts made to minimise error in data collection?	Y	Two reviewers independently extracted data using standardized forms. Verified by third reviewer.	LOW
	3.2 Were sufficient study characteristics available for interpretation?	Y	Table 1 provides detailed study characteristics. Supplementary tables include exclusion reasons and risk of bias.	
	3.3 Were all relevant study results collected for use in the synthesis?	Y	Data extracted for all predefined outcomes: insulin, HOMA-IR, glucose, HbA1c, BMI, muscle mass, strength, CRP, TNF-α, IL-6.	
	3.4 Was risk of bias (or methodological quality) formally assessed using appropriate criteria?	Y	Cochrane RoB 2.0 tool used. Assessment summarized in Supplementary Table S5 and Figure 2.	
	3.5 Were efforts made to minimise error in risk of bias assessment?	Y	Two reviewers independently assessed RoB. Disagreements resolved through discussion.	
DOMAIN 4: SYNTHESIS AND FINDINGS	4.1 Did the synthesis include all studies that it should?	Y	All eligible studies included in meta-analysis. Excluded studies listed with reasons in Supplementary Table S2.	LOW
	4.2 Were all pre-defined analyses reported or departures explained?	Y	All outcomes pre-specified in methods were analyzed and reported. Subgroup and sensitivity analyses conducted as planned.	

4.3 Was the synthesis appropriate given the nature of studies?	Y	Random-effects model used due to expected heterogeneity. Appropriate for varied interventions and populations.
4.4 Was between-study variation (heterogeneity) minimal or addressed in the synthesis?	PY	High heterogeneity (I^2 up to 94%) acknowledged. Subgroup analyses performed (duration, frequency, sex, BMI). Sensitivity analyses conducted.
4.5 Were the findings robust (e.g., funnel plot/sensitivity analyses)?	Y	Sensitivity (leave-one-out) analyses performed. Funnel plots and Egger's test used to assess publication bias (Figures S1–S5).
4.6 Were biases in primary studies minimal or addressed in the synthesis?	PY	RoB assessment performed. Sensitivity analysis excluding high-risk studies mentioned. Some primary studies had high/some concern RoB.

Phase 3: Judging risk of bias

Summarize the concerns identified during the Phase 2 assessment:

Domain	Concern	Evidence / Rationale
1. Eligibility Criteria	LOW	Protocol was pre-registered (PROSPERO). PICOS criteria were clearly defined and appropriate. A language restriction was applied.
2. Identification/Selection	LOW	Comprehensive search across 4 major databases plus supplementary grey literature and reference checking. Dual independent screening was performed. The English-language restriction remains a limitation, but the search strategy was otherwise thorough and reproducible.
3. Data Collection/Appraisal	LOW	Duplicate independent data extraction and risk of bias assessment (using Cochrane RoB 2.0). Sufficient study characteristics and outcome data were extracted. Processes were clearly described to minimize error.
4. Synthesis and Findings	LOW	Appropriate random-effects meta-analysis was conducted. High heterogeneity was transparently reported and explored via pre-specified subgroup and sensitivity analyses. Publication bias was assessed via funnel plots and Egger's test where applicable. All pre-defined outcomes were reported.

RISK OF BIAS IN THE REVIEW

Signaling Question	Response	Evidence / Rationale
A. Did the interpretation of findings address all of the concerns identified in Domains 1 to 4?	Y	Discussion acknowledges limitations: language restriction, high heterogeneity, variability in protocols, lack of blinding in some trials.
B. Was the relevance of identified studies to the review's research question appropriately considered?	Y	All studies fit PICOS criteria. Subgroup analyses (e.g., by BMI, sex) reflect consideration of relevance.
C. Did the reviewers avoid emphasizing results on the basis of their statistical significance?	Y	Reported effect sizes with CIs, acknowledged non-significant outcomes (TNF-α, IL-6), and discussed clinical vs. statistical significance.

Y=YES, PY=PROBABLY YES, PN=PROBABLY NO, N=NO, NI=NO INFORMATION

2.3. Detailed Risk of Bias Assessment for the Systematic Review by Jordan Hernandez-Martinez et al. (2025)

Phase 1: Assessing relevance

Category	Target question	Review being assessed
Patients / Population(s)	Adults with sarcopenic obesity (≥18 years; meets ESPEN/EASO/Asian/Brazilian sarcopenic obesity diagnostic criteria).	Adults with sarcopenic obesity (mean age 68.9±11.1 years, 453 participants); ≥80% female; diagnosed per ESPEN/EASO/Asian criteria.
Intervention(s)	Concurrent training (CT: resistance + endurance training) with measurable duration/frequency/intensity (≥4 weeks, ≥1 session/week, ≥30 min/session).	CT (resistance + endurance training); 8-24 weeks duration, 2-5 sessions/week, 50-80 min/session; intensity 40%-80% 1RM (resistance) or 50%-80% HRmax (endurance).
Comparator(s)	Non-exercise control group (usual care/no structured exercise) or baseline values.	Non-exercise control group (usual care/no structured exercise); no active exercise comparators.
Outcome(s)	Biomarkers (IGF-1, IL-6, CRP, leptin, cholesterol, triglycerides), morphological variables (BMI, waist circumference, body fat, appendicular skeletal muscle mass), physical performance (walking speed, maximal isometric handgrip strength [MIHS], knee extension strength).	All pre-defined outcomes reported, significant effects on IGF-1, leptin, BMI, waist circumference, body fat, ASM/weight, walking speed, knee extension strength.

Phase 2: Identifying concerns with the review process

Domain	Signaling Questions	Response	Evidence / Rationale	Risk Bias Assessment
DOMAIN 1: STUDY ELIGIBILITY CRITERIA	1.1 Did the review adhere to pre-defined objectives and eligibility criteria?	Y	Protocol registered in PROSPERO (CRD420251052935). PRISMA guidelines followed. PICOS criteria clearly stated in Table 1 and Section 2.2.	LOW
	1.2 Were the eligibility criteria appropriate for the review question?	Y	PICOS appropriate for examining effects of CT in sarcopenic obesity on targeted outcomes. Criteria for diagnosis, intervention, and outcomes well-aligned with research question.	
	1.3 Were eligibility criteria unambiguous?	Y	Clear definitions for population (diagnostic criteria specified), intervention (type, dose minimums), comparator, outcomes, and study design (RCTs).	
	1.4 Were all restrictions in eligibility criteria based on study characteristics appropriate?	PY	Restriction to RCTs is appropriate. Minimum intervention duration (4 weeks) is justified. No restrictions on sample size or publication date.	
	1.5 Were any restrictions in eligibility criteria based on sources of information appropriate?	Y	No restrictions based on publication status or language.	
DOMAIN 2: IDENTIFICATION AND SELECTION OF STUDIES	2.1 Did the search include an appropriate range of databases/electronic sources?	Y	Six databases searched: PubMed, MEDLINE, CINAHL Complete, Scopus, Cochrane Library, Web of Science. No language restrictions.	UNCLEAR
	2.2 Were methods additional to database searching used to identify relevant reports?	NI	No mention of checking reference lists, contacting authors, or searching trial registries beyond those included in databases	
	2.3 Were the terms and structure of the search strategy likely to retrieve as many eligible studies as possible?	Y	Detailed search string provided using MeSH and free-text terms related to key concepts.	
	2.4 Were restrictions based on date, publication format, or language appropriate?	Y	No restrictions based on date or publication format. No language restriction applied.	
	2.5 Were efforts made to minimise error in selection of studies?	Y	Two reviewers independently screened titles/abstracts and full texts. No discrepancies reported. (Section 2.4)	
DOMAIN 3: DATA COLLECTION AND STUDY APPRAISAL	3.1 Were efforts made to minimise error in data collection?	Y	Two reviewers independently extracted data. Discrepancies resolved by discussion or third reviewer.	LOW
	3.2 Were sufficient study characteristics available for interpretation?	Y	Table 3 provides extensive study characteristics: country, design, population, intervention details, outcomes, adherence.	
	3.3 Were all relevant study results collected for use in the synthesis?	Y	Data extracted for all pre-specified biomarkers, morphological variables, and physical performance outcomes. (Section 2.6, Table 4)	
	3.4 Was risk of bias (or methodological quality) formally assessed using appropriate criteria?	Y	Cochrane RoB 2.0 tool used independently by two reviewers, validated by a third.	
	3.5 Were efforts made to minimise error in risk of bias assessment?	Y	Two reviewers independently assessed RoB, with third reviewer for validation.	

DOMAIN 4: SYNTHESIS AND FINDINGS	4.1 Did the synthesis include all studies that it should?	Y	All 8 eligible RCTs included in meta-analysis.	HIGH
	4.2 Were all pre-defined analyses reported or departures explained?	Y	Protocol registered. Most pre-specified analyses reported. Minor departure: cumulative meta-analysis used but not pre-specified in PROSPERO summary (mentioned in results).	
	4.3 Was the synthesis appropriate given the nature of studies?	Y	Random-effects model used due to anticipated heterogeneity. Methods for continuous outcomes (Hedges' g) appropriate. Subgroup and meta-regression performed.	
	4.4 Was between-study variation (heterogeneity) minimal or addressed in the synthesis?	PN	High statistical heterogeneity (I^2 up to 98.6%) for many outcomes. Addressed via random-effects model, subgroup analysis, and meta-regression, but residual heterogeneity remains substantial and partly unexplained.	
	4.5 Were the findings robust (e.g., funnel plot/sensitivity analyses)?	Y	Leave-one-out sensitivity and influence analyses performed for outcomes with ≥4 studies. Funnel plots and Egger's test used for publication bias. However, for outcomes with few studies (n<4), robustness could not be assessed.	
	4.6 Were biases in primary studies minimal or addressed in the synthesis?	Y	RoB assessment showed 3/8 studies high risk, 3/8 some concerns. Sensitivity analysis excluding high-risk studies was performed where possible. However, the synthesis includes studies with high RoB, which may influence results.	

Phase 3: Judging risk of bias

Summarize the concerns identified during the Phase 2 assessment:

Domain	Concern	Evidence / Rationale
1. Eligibility Criteria	LOW	Protocol pre-registered, criteria clear and appropriate. Language restriction: none.
2. Identification/Selection	HIGH	Lack of supplementary search methods (grey literature, reference checking) increases risk of missing relevant studies. Only database searching used.
3. Data Collection/Appraisal	LOW	Duplicate independent processes for data extraction and RoB assessment using Cochrane RoB 2.0. Sufficient detail provided.
4. Synthesis and Findings	HIGH	high heterogeneity (I^2 >90% for many outcomes) undermines reliability of pooled estimates. Sensitivity analyses show results for several biomarkers (leptin, CRP, triglycerides, body fat mass) are heavily influenced by single studies, indicating instability.

RISK OF BIAS IN THE REVIEW

Signaling Question	Response	Evidence / Rationale
A. Did the interpretation of findings address all of the concerns identified in Domains 1 to 4?	PY	Discussion acknowledges limitations: high heterogeneity, risk of bias in primary studies, influence of single studies on some outcomes, lack of dietary control, limited generalizability (mostly older women). However, does not explicitly discuss the limitation of not using supplementary search methods.
B. Was the relevance of identified studies to the review's research question appropriately considered?	Y	All included RCTs directly assessed CT in sarcopenic obesity populations with relevant outcomes. Subgroup analyses (e.g., by training dose) reflect consideration of relevant modifiers.
C. Did the reviewers avoid emphasizing results on the basis of their statistical significance?	N	Reported effect sizes with 95% CIs for all outcomes. Discussed non-significant findings (e.g., IL-6, CRP, MIHs) and highlighted clinical relevance beyond statistical significance.

Y=YES, PY=PROBABLY YES, PN=PROBABLY NO, N=NO, NI=NO INFORMATION

2.4. Detailed Risk of Bias Assessment for the Systematic Review by Liang Tan et al. (2025)

Phase 1: Assessing relevance

Category	Target question	Review being assessed
Patients / Population(s)	Overweight/obese women with breast cancer (BMI ≥25 kg/m², stage I-III, postmenopausal/pre-menopausal).	Overweight/obese women with breast cancer (mean BMI 25-34.2 kg/m², stage I-III; 562 intervention/554 control participants; mean age 48.42-59.8 years).
Intervention(s)	Exercise (aerobic/resistance/combined/HIIT) with measurable frequency/duration/intensity (≥12 weeks).	Exercise (aerobic/AE, resistance/RT, combined/CE, high-intensity interval/HIIT); 12 weeks-12 months, 2-4 sessions/week, moderate-high intensity.
Comparator(s)	Non-exercise control group (usual care/no structured exercise).	Non-exercise control group (usual care/no structured exercise); no active exercise comparators.
Outcome(s)	Inflammatory markers (CRP, TNF-α, IL-6, IL-10, leptin, adiponectin).	All pre-defined outcomes reported; significant effects on CRP, IL-6, leptin, adiponectin; non-significant on TNF-α, IL-10.

Phase 2: Identifying concerns with the review process

Domain	Signaling Questions	Response	Evidence / Rationale	Risk Bias Assessment
DOMAIN 1: STUDY ELIGIBILITY CRITERIA	1.1 Did the review adhere to pre-defined objectives and eligibility criteria?	PY	A protocol was registered in PROSPERO (CRD42023492958). PICOS criteria are stated in the Methods. However, there is a discrepancy: the text states "Unlimited" duration, yet the flow diagram excluded a study for "intervention time less than 12 weeks".	LOW
	1.2 Were the eligibility criteria appropriate for the review question?	Y	The PICOS criteria (women with overweight/obesity and breast cancer, exercise interventions, inflammatory markers, RCTs) are appropriate for the research question.	
	1.3 Were eligibility criteria unambiguous?	Y	Criteria for population (overweight/obese BC patients), intervention (exercise), outcomes (specific inflammatory markers), and study design (RCTs) are clearly listed.	
	1.4 Were all restrictions in eligibility criteria based on study characteristics appropriate?	PY	Restriction to RCTs is appropriate for causal inference. No other major restrictions on study characteristics (e.g., sample size) were applied.	
	1.5 Were any restrictions in eligibility criteria based on sources of information appropriate?	PY	The review was restricted to studies "Written in English". This introduces a potential for language bias and limits the comprehensiveness of the evidence.	
DOMAIN 2: IDENTIFICATION AND SELECTION OF STUDIES	2.1 Did the search include an appropriate range of databases/electronic sources?	Y	Five major databases were searched: PubMed, Cochrane, Embase, Web of Science, and EBSCO.	HIGH
	2.2 Were methods additional to database searching used to identify relevant reports?	NI	The manuscript does not explicitly state that reference lists of included studies, grey literature, or trial registries (beyond those in databases) were searched. The PRISMA checklist claims this was done, but it's not detailed in the methods.	
	2.3 Were the terms and structure of the search strategy likely to retrieve as many eligible studies as possible?	Y	A detailed search strategy is provided in Supplementary Table S1, including MeSH terms and keywords related to key concepts.	
	2.4 Were restrictions based on date, publication format, or language appropriate?	PN	No date restrictions were applied. However, the English-language restriction is a limitation. There is also a discrepancy in the reported search end date (May 2024 in Abstract vs. Nov 2024 in Methods).	
	2.5 Were efforts made to minimise error in selection of studies?	Y	Two researchers independently screened titles/abstracts and full texts using EndNote software. Disagreements were resolved by discussion or a third reviewer.	
DOMAIN 3: DATA COLLECTION AND STUDY APPRAISAL	3.1 Were efforts made to minimise error in data collection?	Y	Data extraction was performed independently by two researchers using standardized forms. Disagreements were resolved by discussion.	LOW
	3.2 Were sufficient study characteristics available for interpretation?	Y	Supplementary Table S3 provides detailed characteristics of included studies (age, BMI, intervention type, intensity, duration, outcomes).	
	3.3 Were all relevant study results collected for use in the synthesis?	Y	Data were extracted for all pre-specified inflammatory markers (CRP, TNF-α, IL-6, leptin, IL-10, adiponectin). Means and SDs were extracted or calculated.	
	3.4 Was risk of bias (or methodological quality) formally assessed using appropriate criteria?	Y	The Cochrane Risk of Bias Tool (RoB 1) was used to assess the included RCTs across standard domains.	
	3.5 Were efforts made to minimise error in risk of bias assessment?	Y	Quality assessment was carried out independently by two researchers, with disagreements resolved by a third researcher.	
DOMAIN 4:	4.1 Did the synthesis include all studies that it should?	Y	All eligible studies were included in the meta-analyses. One study was excluded from meta-analysis due to insufficient data, which is justified.	LOW

SYNTHESIS AND FINDINGS	4.2 Were all pre-defined analyses reported or departures explained?	Y	The analyzed outcomes matched those specified in the methods. Subgroup analyses by exercise type were conducted as planned.
	4.3 Was the synthesis appropriate given the nature of studies?	Y	A random-effects model was appropriately used due to expected clinical and methodological variability. Different effect measures (MD/SMD) were used based on outcome type.
	4.4 Was between-study variation (heterogeneity) minimal or addressed in the synthesis?	Y	Heterogeneity was very high for several key outcomes (e.g., IL-6 I^2=94%). The authors addressed this via subgroup analysis (by exercise type).
	4.5 Were the findings robust (e.g., funnel plot/sensitivity analyses)?	Y	Sensitivity analyses (leave-one-out) were performed. Publication bias was assessed via funnel plots and Egger's test, with no significant bias detected for the reported outcomes.
	4.6 Were biases in primary studies minimal or addressed in the synthesis?	PY	The RoB of primary studies was assessed. Sensitivity analysis excluding trials "at risk of assessing bias" was mentioned, though not fully detailed. The synthesis included studies with varied RoB.

Phase 3: Judging risk of bias

Summarize the concerns identified during the Phase 2 assessment:

Domain	Concern	Evidence / Rationale
1. Eligibility Criteria	LOW	Protocol was pre-registered (PROSPERO CRD42023492958). PICOS criteria were clearly defined and appropriate for the research question. The main limitation was a restriction to English-language publications, which may introduce selection bias. A minor discrepancy regarding intervention duration criteria was noted but is unlikely to have significantly impacted study selection.
2. Identification/Selection	HIGH	The search covered five major databases (PubMed, Cochrane, Embase, Web of Science, EBSCO). Dual, independent screening was performed. The primary limitation is the lack of explicit reporting on supplementary search methods (e.g., checking reference lists, searching grey literature), and the English-language restriction. There is also a discrepancy in the reported search end date (May 2024 in Abstract vs. Nov 2024 in Methods).The search strategy itself was detailed and reproducible.
3. Data Collection/Appraisal	LOW	The review employed rigorous, independent duplicate processes for both data extraction and quality assessment (using the Cochrane RoB Tool). Sufficient study characteristics and outcome data were extracted and presented. These processes were clearly described to minimize error.
4. Synthesis and Findings	LOW	Appropriate statistical methods were used (random-effects models). All pre-specified outcomes were analyzed. High statistical heterogeneity was present for several outcomes (e.g., IL-6 I^2=94%) but was transparently reported and explored via pre-specified subgroup analyses (by exercise type). Sensitivity analyses and publication bias assessments (funnel plots, Egger's test) were conducted, supporting the robustness of the findings.

RISK OF BIAS IN THE REVIEW

Signaling Question	Response	Evidence / Rationale
A. Did the interpretation of findings address all of the concerns identified in Domains 1 to 4?	PN	The discussion acknowledges limitations: high heterogeneity, potential publication bias, and the English-language restriction. It does not explicitly discuss the lack of supplementary search methods or the discrepancy in search dates/duration criteria.
B. Was the relevance of identified studies to the review's research question appropriately considered?	Y	All included studies directly addressed the PICO question. Subgroup analyses by exercise type reflect consideration of intervention relevance.
C. Did the reviewers avoid emphasizing results on the basis of their statistical significance?	Y	Effect sizes with 95% CIs are reported for all outcomes. Non-significant findings (e.g., for TNF-伪, IL-10) are clearly reported and discussed.

Y=YES, PY=PROBABLY YES, PN=PROBABLY NO, N=NO, NI=NO INFORMATION

2.5. Detailed Risk of Bias Assessment for the Systematic Review by Fernanda M. Silva et al. (2024)

Phase 1: Assessing relevance

Category	Target question	Review being assessed
Patients / Population(s)	Non-diabetic sedentary adults (18-64 years, BMI ≥25 kg/m², <150 min/week physical activity).	Non-diabetic sedentary adults (18-64 years; mean BMI 29.03±3.59 kg/m²; 852 participants: 476 intervention/376 control).
Intervention(s)	Combined exercise training (aerobic + resistance) with measurable frequency/duration/intensity (≥4 weeks, 3-6 sessions/week).	Combined exercise training (aerobic + resistance); 8-24 weeks, 3-6 sessions/week, 50-90 min/session (aerobic: 40%-85% HRmax/VO_2peak; resistance: 20%-90% 1RM).
Comparator(s)	Non-exercise control (habitual sedentary lifestyle, no structured exercise/diet intervention).	Non-exercise control (habitual lifestyle, no structured exercise; no concurrent diet interventions).
Outcome(s)	Glucose metabolism markers (fasting glucose, insulin, HOMA-IR, HbA_{1c}), adipokines (adiponectin, leptin), inflammatory cytokines (IL-6, TNF-α, CRP).	All pre-defined outcomes reported; significant improvements in fasting glucose, insulin, HOMA-IR, TNF-α, CRP; no significant effects on HbA_{1c}, adiponectin, leptin, IL-6.

Phase 2: Identifying concerns with the review process

Domain	Signaling Questions	Response	Evidence / Rationale	Risk Bias Assessment
DOMAIN 1: STUDY ELIGIBILITY CRITERIA	1.1 Did the review adhere to pre-defined objectives and eligibility criteria?	Y	Study protocol registered in PROSPERO (CRD42023381237) with pre-defined objectives and eligibility criteria; full-text explicitly states adherence to registered criteria.	LOW
	1.2 Were the eligibility criteria appropriate for the review question?	Y	Criteria target non-diabetic sedentary adults, supervised combined exercise, and key metabolic/inflammatory outcomes, directly aligning with the review's objective.	
	1.3 Were eligibility criteria unambiguous?	PY	Criteria clearly define age (18-64 years), activity level (<150 min/week), intervention type (combined aerobic + resistance), and exclusion of comorbidities (e.g., T2DM, cardiovascular disease); minor ambiguity in "sedentary" definition (no objective activity measurement specified).	
	1.4 Were all restrictions in eligibility criteria based on study characteristics appropriate?	Y	Restrictions (e.g., RCT design, ≥4 weeks intervention) are justified for answering the review question; no unnecessary restrictions (e.g., sample size, publication year) were applied.	
	1.5 Were any restrictions in eligibility criteria based on sources of information appropriate?	PY	No restrictions on publication status.language (included English, Portuguese, Spanish, French).	
DOMAIN 2: IDENTIFICATION AND SELECTION OF STUDIES	2.1 Did the search include an appropriate range of databases/electronic sources?	PY	Searched 4 core databases (PubMed, Web of Science, Scopus, Cochrane Library) covering sport science, medicine, and public health; appropriate for the topic.	LOW
	2.2 Were methods additional to database searching used to identify relevant reports?	Y	Hand-searched reference lists of included studies and clinical trial registries (e.g., ICTRP) to retrieve unpublished/ongoing studies.	
	2.3 Were the terms and structure of the search strategy likely to retrieve as many eligible studies as possible?	Y	Search strategy follows PICO framework, includes MeSH terms and free-text keywords (e.g., "combined training", "insulin resistance"), and is provided in Supplementary Table S1 for reproducibility.	
	2.4 Were restrictions based on date, publication format, or language appropriate?	PY	No date restrictions; included articles, meeting abstracts, and book chapters. Language restriction	
	2.5 Were efforts made to minimise error in selection of studies?	Y	Two independent reviewers screened titles/abstracts and full-texts; disagreements resolved via consensus or third reviewer.	
DOMAIN 3: DATA COLLECTION AND STUDY APPRAISAL	3.1 Were efforts made to minimise error in data collection?	Y	Two independent reviewers extracted data using standardized forms; disagreements resolved via consensus.	LOW
	3.2 Were sufficient study characteristics available for interpretation?	Y	Extracted detailed characteristics: sample size, age, BMI, intervention details, adherence, and outcome data. presented in Table 1 and Supplementary Table S2.	
	3.3 Were all relevant study results collected for use in the synthesis?	Y	Collected data for all pre-defined outcomes.Converted SE to SD per Cochrane Handbook; contacted authors for missing data .	
	3.4 Was risk of bias (or methodological quality) formally assessed using appropriate criteria?	Y	Used Cochrane RoB 2 tool (validated for RCTs) to assess 5 bias domains,results reported in Supplementary Figure S1.	
	3.5 Were efforts made to minimise error in risk of bias assessment?	Y	Two independent reviewers conducted RoB 2 assessments, disagreements resolved via consensus or third reviewer.	

DOMAIN 4: SYNTHESIS AND FINDINGS	4.1 Did the synthesis include all studies that it should?	Y	All 24 eligible RCTs were included in the synthesis; no unexplained exclusions.	LOW
	4.2 Were all pre-defined analyses reported or departures explained?	PY	Pre-defined analyses were reported, but cumulative Meta-analysis was used without pre-registration or explanation in the protocol.	
	4.3 Was the synthesis appropriate given the nature of studies?	Y	Used random-effects model,standardized mean difference (SMD) with 95% CI.	
	4.4 Was between-study variation (heterogeneity) minimal or addressed in the synthesis?	PY	Heterogeneity was tested via Q-statistic, I^2, and τ^2; high heterogeneity was addressed via subgroup analysis and meta-regression, though not fully resolved.	
	4.5 Were the findings robust (e.g., funnel plot/sensitivity analyses)?	Y	Sensitivity analyses ("one-study-removed") confirmed result stability; funnel plots and Egger's test assessed publication bias for outcomes with ≥10 studies.	
	4.6 Were biases in primary studies minimal or addressed in the synthesis?	Y	Excluded high RoB studies in sensitivity analyses; no significant change in results, indicating robustness to primary study biases.	

Phase 3: Judging risk of bias

Summarize the concerns identified during the Phase 2 assessment:

Domain	Concern	Evidence / Rationale
1. Eligibility Criteria	LOW	Low risk, no significant concerns.
2. Identification/Selection	LOW	Low risk, no significant concerns.
3. Data Collection/Appraisal	LOW	Low risk, no significant concerns.
4. Synthesis and Findings	LOW	Unreported pre-registration of cumulative Meta-analysis; unresolved heterogeneity for some outcomes.

RISK OF BIAS IN THE REVIEW

Signaling Question	Response	Evidence / Rationale
A. Did the interpretation of findings address all of the concerns identified in Domains 1 to 4?	Y	Discussion section explicitly addresses heterogeneity and limitations.
B. Was the relevance of identified studies to the review's research question appropriately considered?	Y	All included studies align with PICO criteria; discussion compares findings with previous reviews to contextualize relevance.
C. Did the reviewers avoid emphasizing results on the basis of their statistical significance?	Y	Presented both significant and non-significant outcomes objectively; discussed limitations of non-significant findings.

Y=YES, PY=PROBABLY YES, PN=PROBABLY NO, N=NO, NI=NO INFORMATION

2.6. Detailed Risk of Bias Assessment for the Systematic Review by Le-Yang Li et al. (2022)

Phase 1: Assessing relevance

Category	Target question	Review being assessed
Patients / Population(s)	Obese/overweight children and adolescents (<20 years old), no severe comorbidities.	Obese/overweight children and adolescents (<20 years old); 1010 participants (510 intervention/500 control) from 28 RCTs; mean BMI ≥25 kg/m².
Intervention(s)	Exercise training (aerobic/resistance/combined/HIIT) with measurable frequency/duration/intensity (4 weeks-6 months, ≥3 sessions/week).	Exercise training (aerobic/RT/combined/HIIT); 4 weeks-6 months, 3-5 sessions/week, 24-90 min/session (aerobic: 50%-85% HRmax/VO_2peak; resistance: 40%-80% 1RM; HIIT: 85%-250% VO_2peak).
Comparator(s)	Non-exercise control (habitual lifestyle, no structured exercise/diet intervention).	Non-exercise control (no structured exercise; concurrent diet interventions excluded if control group not exposed).
Outcome(s)	Glucose metabolism (FBG, FINS, HOMA-IR), inflammatory markers (IL-6, TNF-α, CRP), adipokines (leptin, adiponectin).	All pre-defined outcomes reported; significant improvements in FBG, FINS, HOMA-IR, IL-6, CRP, leptin, adiponectin; no significant effect on TNF-α.

Phase 2: Identifying concerns with the review process

Domain	Signaling Questions	Response	Evidence / Rationale	Risk Bias Assessment
DOMAIN 1: STUDY ELIGIBILITY CRITERIA	1.1 Did the review adhere to pre-defined objectives and eligibility criteria?	Y	Registered on PROSPERO (CRD42023472704) with pre-specified eligibility criteria; full text followed the criteria.	LOW
	1.2 Were the eligibility criteria appropriate for the review question?	Y	Criteria (RCT design, participants <20 years old, exercise intervention) directly address the aim of assessing exercise effects on target indicators.	
	1.3 Were eligibility criteria unambiguous?	PY	Clearly defined study type, participants, intervention, and exclusion criteria; only lacked explicit disease diagnostic standards for obesity.	
	1.4 Were all restrictions in eligibility criteria based on study characteristics appropriate?	Y	No inappropriate restrictions on publication time, sample size, or study quality; only included RCTs (appropriate for intervention effect assessment).	
	1.5 Were any restrictions in eligibility criteria based on sources of information appropriate?	Y	Included both published and unpublished studies; no inappropriate restrictions on data availability.	
DOMAIN 2: IDENTIFICATION AND SELECTION OF STUDIES	2.1 Did the search include an appropriate range of databases/electronic sources?	Y	Searched PubMed, Web of Science, Embase,supplemented by reference lists and conference abstracts.	LOW
	2.2 Were methods additional to database searching used to identify relevant reports?	Y	Hand-searched conference abstracts, reviewed reference lists of included studies, and searched clinical trial registries (ICTRP, ClinicalTrials.gov).	
	2.3 Were the terms and structure of the search strategy likely to retrieve as many eligible studies as possible?	Y	Provided detailed search strategies for each database (Table S1) with synonyms and related terms; strategy was reproducible.	
	2.4 Were restrictions based on date, publication format, or language appropriate?	PY	Restricted to English publications, potentially missing non-English eligible studies.	
	2.5 Were efforts made to minimise error in selection of studies?	Y	Two independent reviewers screened titles/abstracts and full texts; disputes resolved by a third senior researcher.	
DOMAIN 3: DATA COLLECTION AND STUDY APPRAISAL	3.1 Were efforts made to minimise error in data collection?	Y	Two independent reviewers extracted data using pre-designed forms; disputes resolved via discussion or third-party consultation	LOW
	3.2 Were sufficient study characteristics available for interpretation?	Y	Provided detailed characteristics of included studies (Table 1), including intervention type, duration, sample size, and outcomes.	
	3.3 Were all relevant study results collected for use in the synthesis?	Y	Extracted all target indicators and reported effect sizes for each.	
	3.4 Was risk of bias (or methodological quality) formally assessed using appropriate criteria?	Y	Used the Cochrane Risk of Bias Tool to assess 7 domains.	
	3.5 Were efforts made to minimise error in risk of bias assessment?	Y	Two independent reviewers conducted bias assessment; disputes resolved by a third researcher.	

DOMAIN 4: SYNTHESIS AND FINDINGS	4.1 Did the synthesis include all studies that it should?	Y	Included 28 RCTs after removing duplicates and ineligible studies; flowchart (Figure 1) documented the process transparently.	LOW
	4.2 Were all pre-defined analyses reported or departures explained?	PY	Reported pre-specified analyses, no unexplained departures from the PROSPERO protocol.	
	4.3 Was the synthesis appropriate given the nature of studies?	Y	Used random-effects models for high heterogeneity (I^2>80%); matched analysis methods to data types (dichotomous/continuous).	
	4.4 Was between-study variation (heterogeneity) minimal or addressed in the synthesis?	Y	Assessed heterogeneity via I^2 and Q-test; conducted subgroup analysis and meta-regression to explore sources.	
	4.5 Were the findings robust (e.g., funnel plot/sensitivity analyses)?	Y	Sensitivity analysis confirmed result stability, funnel plots evaluated publication bias.	
	4.6 Were biases in primary studies minimal or addressed in the synthesis?	Y	Excluded high-bias-risk studies and conducted sensitivity analysis.	

Phase 3: Judging risk of bias

Summarize the concerns identified during the Phase 2 assessment:

Domain	Concern	Evidence / Rationale
1. Eligibility Criteria	LOW	No critical concerns; criteria were pre-defined, appropriate, and unambiguous.
2. Identification/Selection	LOW	Language restriction to English may limit generalizability, but no evidence of missed key studies.
3. Data Collection/Appraisal	LOW	Rigorous processes minimised error.
4. Synthesis and Findings	LOW	Appropriate statistical methods, heterogeneity handling, and robustness checks.

RISK OF BIAS IN THE REVIEW

Signaling Question	Response	Evidence / Rationale
A. Did the interpretation of findings address all of the concerns identified in Domains 1 to 4?	Y	Discussion section acknowledged limitations and their potential impacts.
B. Was the relevance of identified studies to the review's research question appropriately considered?	Y	All included studies directly focused on exercise effects on target indicators in the target population.
C. Did the reviewers avoid emphasizing results on the basis of their statistical significance?	Y	Reported both statistically significant and non-significant results objectively; no overinterpretation.

Y=YES, PY=PROBABLY YES, PN=PROBABLY NO, N=NO, NI=NO INFORMATION

2.7. Detailed Risk of Bias Assessment for the Systematic Review by Sameer Badri Al-Mhanna et al.-1 (2024)

Phase 1: Assessing relevance

Category	Target question	Review being assessed
Patients / Population(s)	Breast cancer patients/survivors with overweight/obesity (BMI ≥25 kg/m²), no severe contraindications to exercise.	Breast cancer patients/survivors with overweight/obesity (mean BMI 26.6-36.4 kg/m²); 1148 female participants (574 intervention/574 control) from 17 RCTs; mean age 54.0±3.4 years.
Intervention(s)	Combined aerobic and resistance training (CART) with measurable frequency/duration/intensity (8-52 weeks, 2-6 sessions/week).	CART (aerobic + resistance); 8-52 weeks, 2-6 sessions/week, 40-90 min/session (aerobic: 40%-85% HRmax/HRR; resistance: 40%-80% 1RM).
Comparator(s)	Standard treatment (no structured exercise, usual care without exercise intervention).	Standard treatment (no structured exercise; concurrent non-exercise interventions balanced between groups).
Outcome(s)	Body composition (BMI, BF, FM, FFM), cardiometabolic indicators (lipids), inflammation (TNF-α, IL-6), adipokines (leptin, adiponectin), cancer-related indicators (fatigue, sleep, QoL).	All pre-defined outcomes reported; significant improvements in BMI, BF, FM, FFM, TC, TG, TNF-α, leptin, NK cells, fatigue, sleep, QoL; no significant effects on LDL-C, IL-6, adiponectin.

Phase 2: Identifying concerns with the review process

Domain	Signaling Questions	Response	Evidence / Rationale	Risk Bias Assessment
DOMAIN 1: STUDY ELIGIBILITY CRITERIA	1.1 Did the review adhere to pre-defined objectives and eligibility criteria?	PY	The protocol was registered in PROSPERO (CRD42022308214), and the review states that eligibility criteria were defined a priori. However, no direct comparison between the protocol and the published review is provided to confirm full adherence.	LOW
	1.2 Were the eligibility criteria appropriate for the review question?	Y	PICOS is clearly defined and aligns with the research question.	
	1.3 Were eligibility criteria unambiguous?	Y	Inclusion/exclusion criteria are clearly listed with specific details	
	1.4 Were all restrictions in eligibility criteria based on study characteristics appropriate?	Y	Restrictions are justified and appropriate.	
	1.5 Were any restrictions in eligibility criteria based on sources of information appropriate?	Y	No language restrictions; included published and unpublished studies.	
DOMAIN 2: IDENTIFICATION AND SELECTION OF STUDIES	2.1 Did the search include an appropriate range of databases/electronic sources?	Y	Multiple databases (PubMed, Web of Science, Scopus, etc.) and supplementary sources (trial registries, reference lists) were searched.	LOW
	2.2 Were methods additional to database searching used to identify relevant reports?	Y	reference lists and trial registries were searched.	
	2.3 Were the terms and structure of the search strategy likely to retrieve as many eligible studies as possible?	Y	Search strategy included relevant keywords and Boolean operators (Table S1).	
	2.4 Were restrictions based on date, publication format, or language appropriate?	Y	No restrictions applied.	
	2.5 Were efforts made to minimise error in selection of studies?	Y	Four authors independently screened studies; conflicts resolved by a fifth author.	
DOMAIN 3: DATA COLLECTION AND STUDY APPRAISAL	3.1 Were efforts made to minimise error in data collection?	Y	Two authors independently extracted data; disagreements resolved through discussion/third author.	LOW
	3.2 Were sufficient study characteristics available for interpretation?	Y	Detailed study characteristics are provided in Table 1.	
	3.3 Were all relevant study results collected for use in the synthesis?	Y	All pre-specified outcomes were extracted and analyzed.	
	3.4 Was risk of bias (or methodological quality) formally assessed using appropriate criteria?	Y	Cochrane Risk of Bias Tool and GRADE were used.	
	3.5 Were efforts made to minimise error in risk of bias assessment?	Y	Two authors independently assessed risk of bias; disagreements resolved by a third author.	
DOMAIN 4:	4.1 Did the synthesis include all studies that it should?	Y	All 17 included studies were synthesized; exclusions are justified in Table S3.	LOW

SYNTHESIS AND FINDINGS	4.2 Were all pre-defined analyses reported or departures explained?	PY	Most pre-specified analyses were reported; subgroup and sensitivity analyses were conducted. Some outcomes had few studies but were still included.
	4.3 Was the synthesis appropriate given the nature of studies?	Y	Random-effects model used; SMD/MD appropriately chosen; heterogeneity assessed.
	4.4 Was between-study variation (heterogeneity) minimal or addressed in the synthesis?	Y	I^2 reported; subgroup and sensitivity analyses performed to address heterogeneity.
	4.5 Were the findings robust (e.g., funnel plot/sensitivity analyses)?	PY	Sensitivity analyses conducted for performance/detection bias; funnel plots not mentioned.
	4.6 Were biases in primary studies minimal or addressed in the synthesis?	PY	Risk of bias was assessed and considered in sensitivity analyses.

Phase 3: Judging risk of bias

Summarize the concerns identified during the Phase 2 assessment:

Domain	Concern	Evidence / Rationale
1. Eligibility Criteria	LOW	Clear and appropriate criteria, minor uncertainty in protocol adherence.
2. Identification/Selection	LOW	Comprehensive search and independent screening.
3. Data Collection/Appraisal	LOW	Independent processes and standard tools used.
4. Synthesis and Findings	LOW	Appropriate methods; heterogeneity and bias addressed.

RISK OF BIAS IN THE REVIEW

Signaling Question	Response	Evidence / Rationale
A. Did the interpretation of findings address all of the concerns identified in Domains 1 to 4?	Y	Discussion acknowledges limitations (small samples, heterogeneity, low GRADE).
B. Was the relevance of identified studies to the review's research question appropriately considered?	Y	Included studies matched PICO; irrelevant studies were excluded.
C. Did the reviewers avoid emphasizing results on the basis of their statistical significance?	Y	Results presented with effect sizes and CIs; discussion highlights uncertainty due to low-quality evidence.

Y=YES, PY=PROBABLY YES, PN=PROBABLY NO, N=NO, NI=NO INFORMATION

2.8. Detailed Risk of Bias Assessment for the Systematic Review by Sameer Badri Al-Mhanna et al.-2 (2024)

Phase 1: Assessing relevance

Category	Target question	Review being assessed
Patients / Population(s)	Overweight/obese adults with type 2 diabetes (BMI ≥25 kg/m²), no severe exercise contraindications.	Overweight/obese adults with T2DM (mean BMI ≥25 kg/m²); 1192 participants (596 intervention/596 control) from 20 RCTs; mean age 57±7 years.
Intervention(s)	Combined aerobic and resistance training (CART) with measurable frequency/duration/intensity (8-60 weeks, 2-5 sessions/week).	CART (aerobic + resistance); 8-60 weeks, 2-5 sessions/week, 40-90 min/session (aerobic: 40%-65% HRR/HRmax; resistance: 40%-60% 1RM).
Comparator(s)	Standard treatment (usual diabetes care, no structured exercise intervention).	Standard treatment (no structured exercise; concurrent non-exercise interventions balanced between groups).
Outcome(s)	Glycemic control ($HbA_{1}c$), blood pressure (SBP/DBP/RHR), inflammation (CRP/TNF-α/IL-6), cardiorespiratory fitness (CRF), QoL, BMI.	All pre-defined outcomes reported; significant improvements in $HbA_{1}c$, DBP, TNF-α, IL-6, CRF, BMI, QoL; no significant effects on RHR, SBP, CRP.

Phase 2: Identifying concerns with the review process

Domain	Signaling Questions	Response	Evidence / Rationale	Risk Bias Assessment
DOMAIN 1: STUDY ELIGIBILITY CRITERIA	1.1 Did the review adhere to pre-defined objectives and eligibility criteria?	Y	Protocol registered in PROSPERO (CRD42022355612). Inclusion/exclusion criteria clearly stated in Methods (pages 4–5).	LOW
	1.2 Were the eligibility criteria appropriate for the review question?	Y	Criteria align with PICO: overweight/obese adults with T2DM, CART intervention, standard care control, cardiometabolic outcomes.	
	1.3 Were eligibility criteria unambiguous?	Y	Inclusion/exclusion criteria are clearly listed with specific details	
	1.4 Were all restrictions in eligibility criteria based on study characteristics appropriate?	Y	RCTs and CCTs included, no unreasonable restrictions on study design, duration, or outcomes.	
	1.5 Were any restrictions in eligibility criteria based on sources of information appropriate?	Y	No language restrictions, included published and unpublished studies	
DOMAIN 2: IDENTIFICATION AND SELECTION OF STUDIES	2.1 Did the search include an appropriate range of databases/electronic sources?	Y	Searched PubMed, Web of Science, Scopus, Cochrane Library, Google Scholar, Science Direct (Table S1).	HIGH
	2.2 Were methods additional to database searching used to identify relevant reports?	Y	Reference lists of included studies and relevant reviews were screened.	
	2.3 Were the terms and structure of the search strategy likely to retrieve as many eligible studies as possible?	PN	Search strategy (Table S1) appears simplistic ("Exercise" OR "Training" AND "Diabet*"), may miss relevant synonyms or MeSH terms.	
	2.4 Were restrictions based on date, publication format, or language appropriate?	Y	No date or language restrictions; included unpublished studies.	
	2.5 Were efforts made to minimise error in selection of studies?	Y	Four authors independently screened titles/abstracts; disagreements resolved by fifth author.	
DOMAIN 3: DATA COLLECTION AND STUDY APPRAISAL	3.1 Were efforts made to minimise error in data collection?	Y	Two authors independently extracted data; third resolved discrepancies.	HIGH
	3.2 Were sufficient study characteristics available for interpretation?	Y	Table 1 provides detailed study characteristics (PICO, setting, duration, outcomes).	
	3.3 Were all relevant study results collected for use in the synthesis?	Y	All pre-specified outcomes extracted and analyzed.	
	3.4 Was risk of bias (or methodological quality) formally assessed using appropriate criteria?	Y	Cochrane Risk of Bias Tool used (Table S2).	
	3.5 Were efforts made to minimise error in risk of bias assessment?	PN	ROB assessment done by two authors, but many studies rated "unclear" in key domains (e.g., blinding, allocation concealment), suggesting incomplete reporting or assessment.	
DOMAIN 4: SYNTHESIS AND FINDINGS	4.1 Did the synthesis include all studies that it should?	Y	All eligible studies included in meta-analyses where possible.	HIGH
	4.2 Were all pre-defined analyses reported or departures explained?	Y	All outcomes from protocol analyzed; subgroup and sensitivity analyses performed.	
	4.3 Was the synthesis appropriate given the	PY	High heterogeneity (I^2 up to 90%) not fully explained; random-effects model used but	

nature of studies?		clinical/methodological diversity not adequately addressed.
4.4 Was between-study variation (heterogeneity) minimal or addressed in the synthesis?	N	High heterogeneity in several outcomes (e.g., SBP I^2=90%, CRF I^2=67%) with limited exploration.
4.5 Were the findings robust (e.g., funnel plot/sensitivity analyses)?	PY	Some sensitivity analyses performed (e.g., excluding high ROB studies), but not for all outcomes.
4.6 Were biases in primary studies minimal or addressed in the synthesis?	Y	Subgroup analysis compared short/long-term intervention effects with no significant differences; high-bias studies (e.g., performance bias) included but did not affect overall results; non-significant outcomes (RHR, SBP, CRP) explicitly reported.

Phase 3: Judging risk of bias

Summarize the concerns identified during the Phase 2 assessment:

Domain	Concern	Evidence / Rationale
1. Eligibility Criteria	LOW	Clear and appropriate criteria, minor uncertainty in protocol adherence.
2. Identification/Selection	HIGH	Inadequate search strategy may miss relevant evidence.
3. Data Collection/Appraisal	HIGH	Risk of bias assessment incomplete; many studies with unclear/high ROB.
4. Synthesis and Findings	HIGH	High heterogeneity and unaddressed primary study biases.

RISK OF BIAS IN THE REVIEW

Signaling Question	Response	Evidence / Rationale
A. Did the interpretation of findings address all of the concerns identified in Domains 1 to 4?	N	Discussion acknowledges heterogeneity and ROB but does not fully address implications for conclusions.
B. Was the relevance of identified studies to the review's research question appropriately considered?	Y	Studies match PICO; relevance discussed in limitations.
C. Did the reviewers avoid emphasizing results on the basis of their statistical significance?	PN	Abstract and conclusions highlight positive findings despite low/very low certainty evidence (GRADE, Table S3).

Y=YES, PY=PROBABLY YES, PN=PROBABLY NO, N=NO, NI=NO INFORMATION

2.9. Detailed Risk of Bias Assessment for the Systematic Review by Yongqing Guo et al. (2024)

Phase 1: Assessing relevance

Category	Target question	Review being assessed
Patients / Population(s)	Obese/overweight individuals (BMI 25.0-29.9/≥30.0 kg/m²), 10-70 years old, no severe exercise contraindications.	Obese/overweight individuals (BMI 25.0-29.9/≥30.0 kg/m²); 1,135 participants (642 intervention/493 control) from 22 studies (19 RCTs/3 self-controlled trials); age range 10-70 years.
Intervention(s)	Aerobic exercise (AE) or resistance exercise (RE) with measurable frequency/duration/intensity (6-52 weeks, 2-5 sessions/week).	AE (cycling, running/walking): 6-52 weeks, 2-5 sessions/week, 30-75 min/session (50%-85% HRR/VO_2max); RE (resistance machines, bench press): 8-52 weeks, 2-3 sessions/week, 8-15 reps/set, 40%-95% 1-RM.
Comparator(s)	Various controls (usual care, no exercise, self-pre-intervention).	Controls include no exercise, usual care, and self-pre-intervention; no concurrent exercise interventions in control groups.
Outcome(s)	Inflammatory markers: C-reactive protein (CRP), interleukin-6 (IL-6), tumor necrosis factor-alpha (TNF-α).	All pre-defined outcomes reported; AE/RE reduce CRP (AE more effective), AE reduces IL-6, neither affects TNF-α; subgroup analyses by exercise type.

Phase 2: Identifying concerns with the review process

Domain	Signaling Questions	Response	Evidence / Rationale	Risk Bias Assessment
DOMAIN 1: STUDY ELIGIBILITY CRITERIA	1.1 Did the review adhere to pre-defined objectives and eligibility criteria?	PY	The review states it adhered to PICOS principle (P2, 2.2). However, the published methods section contains an error (defining both C and S as "All types of comparisons/control..."), creating ambiguity about the pre-defined plan.	HIGH
	1.2 Were the eligibility criteria appropriate for the review question?	PN	The criteria are broadly appropriate. However, including "self-control design" (P3, 2.2) for a question about effects of exercise is problematic as it lacks a concurrent control, potentially introducing bias. The age range (10-70) is very wide, combining physiologically distinct groups.	
	1.3 Were eligibility criteria unambiguous?	N	Major Concern. In Section 2.2 (P3), the criteria for "C (Comparison)" and "S (Study design)" are identical: "All types of comparisons/control, including self-control design." This conflation creates significant ambiguity regarding what study designs were actually eligible.	
	1.4 Were all restrictions in eligibility criteria based on study characteristics appropriate?	Y	Restrictions were minimal: RCTs and self-controlled trials were included. No restrictions on publication date (last 2 decades searched), sample size, or quality. This is appropriate for a comprehensive review.	
	1.5 Were any restrictions in eligibility criteria based on sources of information appropriate?	Y	No restrictions based on publication status or language were applied, which is appropriate to minimize publication bias.	
DOMAIN 2: IDENTIFICATION AND SELECTION OF STUDIES	2.1 Did the search include an appropriate range of databases/electronic sources?	Y	PubMed, Web of Science, CNKI, and Wanfang Database were searched (P2, 2.1). This covers major English and Chinese biomedical databases.	LOW
	2.2 Were methods additional to database searching used to identify relevant reports?	Y	The review states "an additional 2 articles supplemented through references	
	2.3 Were the terms and structure of the search strategy likely to retrieve as many eligible studies as possible?	PY	The review states "utilizing both free-text and MeSH terms" for WoS/PubMed (P2, 2.1). However, no full search strategy is provided in the paper or supplement, making reproducibility and assessment of completeness difficult.	
	2.4 Were restrictions based on date, publication format, or language appropriate?	Y	Search spanned "the last 2 decades" (P2, 2.1). No restrictions on publication format or language mentioned, which is appropriate.	
	2.5 Were efforts made to minimise error in selection of studies?	Y	Two researchers independently conducted literature screening	
DOMAIN 3: DATA COLLECTION AND STUDY APPRAISAL	3.1 Were efforts made to minimise error in data collection?	Y	two researchers independently collected data using a pre-defined form, with disagreements resolved by a third researcher.	LOW
	3.2 Were sufficient study characteristics available for interpretation?	Y	Table 1 (P6) provides detailed characteristics (author, design, population, intervention). The text also describes interventions (P5, 3.3).	
	3.3 Were all relevant study results collected for use in the synthesis?	Y	The outcomes (CRP, IL-6, TNF-α) are clearly defined. Data for these were extracted for meta-analysis (Figures 4,5,6/8,9).	
	3.4 Was risk of bias (or methodological quality) formally assessed using appropriate criteria?	Y	"This study opted for the Cochrane standard for assessment" (P3, 2.4). Results are shown in Figures 2 & 3.	
	3.5 Were efforts made to minimise error in	PY	Implied by "assessed according to Cochrane standards" and presentation of results, but the process	

	risk of bias assessment?		(independent/duplicate) is not explicitly described for quality assessment as it is for data extraction.	
DOMAIN 4: SYNTHESIS AND FINDINGS	4.1 Did the synthesis include all studies that it should?	Y	Based on the stated inclusion criteria and search results, the synthesis includes relevant studies. The PRISMA flow diagram (P3, 3.1) is provided.	LOW
	4.2 Were all pre-defined analyses reported or departures explained?	PY	Subgroup analysis by exercise modality (AE/RE) and outcome (CRP/IL-6/TNF-α) is reported as planned. Sensitivity analysis is reported (P10-11, 3.5). The use of cumulative meta-analysis is noted but not pre-specified (P4, Table 3 rationale).	
	4.3 Was the synthesis appropriate given the nature of studies?	Y	A random-effects model was appropriately used due to high heterogeneity (P5, 2.5; P5, 3.4.1). SMD was used for continuous outcomes. Subgroup analysis was conducted.	
	4.4 Was between-study variation (heterogeneity) minimal or addressed in the synthesis?	Y	High heterogeneity was acknowledged (I^2=77-87%). The random-effects model, subgroup analyses, and exploration of sources in the discussion (P12) appropriately address this.	
	4.5 Were the findings robust (e.g., funnel plot/sensitivity analyses)?	Y	Sensitivity analyses were conducted (P10-11, 3.5). Funnel plots were generated and discussed for all outcomes, acknowledging potential publication bias for CRP and IL-6.	
	4.6 Were biases in primary studies minimal or addressed in the synthesis?	Y	The risk of bias in primary studies was assessed (Fig 2,3). The authors state: "study excluded high bias risk literature for sensitivity analysis" (P4, Table 3).	

Phase 3: Judging risk of bias

Summarize the concerns identified during the Phase 2 assessment:

Domain	Concern	Evidence / Rationale
1. Eligibility Criteria	HIGH	Ambiguous and potentially inappropriate eligibility criteria create a high risk of bias in the composition of the included studies.
2. Identification/Selection	LOW	Search could have been more comprehensive, but processes were adequate.
3. Data Collection/Appraisal	LOW	Processes were adequate and standard tools were used.
4. Synthesis and Findings	LOW	Synthesis methods were robust and appropriate for the data.

RISK OF BIAS IN THE REVIEW

Signaling Question	Response	Evidence / Rationale
A. Did the interpretation of findings address all of the concerns identified in Domains 1 to 4?	PN	The discussion (P12-13) extensively addresses heterogeneity and limitations related to population diversity, intervention intensity/duration, and study design (Domain 4 & partial Domain 1). However, it does not acknowledge or discuss the critical methodological flaw of ambiguous eligibility criteria (Domain 1).
B. Was the relevance of identified studies to the review's research question appropriately considered?	PY	The studies included appear relevant to the broad PICO. The discussion contextualizes findings considering variations in study populations and interventions.
C. Did the reviewers avoid emphasizing results on the basis of their statistical significance?	Y	The results are reported with effect sizes and confidence intervals. The conclusion is nuanced, stating effects for CRP/IL-6 but no effect for TNF-α, based on the analysis rather than statistical significance alone.

Y=YES, PY=PROBABLY YES, PN=PROBABLY NO, N=NO, NI=NO INFORMATION

2.10. Detailed Risk of Bias Assessment for the Systematic Review by Abbas Malandish et al. (2023)

Phase 1: Assessing relevance

Category	Target question	Review being assessed
Patients / Population(s)	Overweight/obese patients with heart failure (HF, including HFrEF/HFpEF/HFmrEF), age ≥18 years, no severe exercise contraindications.	Overweight/obese HF patients (BMI ≥25/30 kg/m² + HF diagnosis); 3693 participants (2141 intervention/1552 control) from 46 RCTs (57 intervention arms); age range 48–77.5 years, covering HFrEF/HFpEF/HFmrEF.
Intervention(s)	Exercise interventions (aerobic, resistance, concurrent training) with measurable frequency/duration/intensity (3 weeks–24 months, 2–7 sessions/week).	Exercise (aerobic: 44 arms; concurrent:10 arms; resistance:3 arms); 3 weeks–24 months, 2–7 sessions/week, 20–120 min/session (intensity: 40%–95% VO_2peak/HRmax/1RM).
Comparator(s)	Usual care, optimal medical therapy, sham intervention, or no structured exercise.	Controls include usual care, medical therapy, sham training, or no exercise; no concurrent exercise interventions.
Outcome(s)	Inflammaging markers: TNF-α, IL-6, IL-1β, IL-8, hs-CRP.	All pre-defined outcomes reported; exercise reduces IL-6 (SMD=-0.205, p=0.002) and hs-CRP (SMD=-0.379, p=0.001); subgroup analysis shows TNF-α reduced in middle-aged/HFrEF/concurrent training/high-intensity groups; no effects on IL-1β/IL-8.

Phase 2: Identifying concerns with the review process

Domain	Signaling Questions	Response	Evidence / Rationale	Risk Bias Assessment
DOMAIN 1: STUDY ELIGIBILITY CRITERIA	1.1 Did the review adhere to pre-defined objectives and eligibility criteria?	Y	The protocol was registered in PROSPERO (CRD42022347164). The methods section describes eligibility criteria (PICO) that align with the stated objectives.	LOW
	1.2 Were the eligibility criteria appropriate for the review question?	Y	The criteria are directly aligned with the review's question of evaluating the impact of exercise on inflammaging in this population.	
	1.3 Were eligibility criteria unambiguous?	Y	Criteria for participants, interventions, comparators, and outcomes are stated.	
	1.4 Were all restrictions in eligibility criteria based on study characteristics appropriate?	PY	Restrictions included: only RCTs, human studies, English language , minimum 2-week follow-up. No restrictions on date or publication status were applied.	
	1.5 Were any restrictions in eligibility criteria based on sources of information appropriate?	Y	No restrictions were placed on publication status. This minimizes publication bias.	
DOMAIN 2: IDENTIFICATION AND SELECTION OF STUDIES	2.1 Did the search include an appropriate range of databases/electronic sources?	Y	The electronic databases of searching including Scopus, PubMed, Web of Science, and Google Scholar	HIGH
	2.2 Were methods additional to database searching used to identify relevant reports?	PN	The search strategy section only mentions database searching. There is no mention of checking reference lists of included studies or relevant reviews, contacting experts,	
	2.3 Were the terms and structure of the search strategy likely to retrieve as many eligible studies as possible?	PY	The provided search strategy (in methods) uses broad keywords for exercise, inflammation, and HF.	
	2.4 Were restrictions based on date, publication format, or language appropriate?	PY	The review restricted inclusion to English language articles only. This is a well-documented source of language bias, as it may systematically exclude relevant studies published in other languages, potentially affecting the completeness and conclusions of the meta-analysis. No restriction on date or format was applied,.	
	2.5 Were efforts made to minimise error in selection of studies?	Y	The authors state: "The titles and abstracts of all articles were screened... by two corresponding authors (A M and M G)." and "full-text of articles were assessed for eligibility process by two corresponding authors." This duplicate, independent screening process minimizes error in study selection.	
DOMAIN 3: DATA COLLECTION AND STUDY APPRAISAL	3.1 Were efforts made to minimise error in data collection?	PY	The authors state: "Data extraction process was performed by corresponding author (A M) and any disagreement was resolved by discussion among corresponding authors (A M and M G).	LOW
	3.2 Were sufficient study characteristics available for interpretation?	Y	Table 2 (participant characteristics) and Table 3 (intervention characteristics) provide extensive details on age, BMI, sample size, country, exercise type, frequency, duration, protocol, and control group. This is sufficient for interpretation and subgroup analysis.	
	3.3 Were all relevant study results collected for use in the synthesis?	Y	The review aimed to collect data on pre-specified inflammaging markers (TNF-α, IL-6, IL-1β, IL-8, hs-CRP). Forest plots are presented for all these outcomes where data were available. Data from multiple intervention arms within a study were handled appropriately.	
	3.4 Was risk of bias (or methodological quality) formally assessed using appropriate criteria?	PY	The authors used the PEDro scale to assess methodological quality. While PEDro is a valid tool for physiotherapy trials, the Cochrane Risk of Bias (RoB) tool is the current standard recommended by Cochrane for assessing RCTs in systematic reviews.	

	3.5 Were efforts made to minimise error in risk of bias assessment?	PY	The methodology states the Pedro scale was used but does not explicitly describe the process. Table 1 presents the scores, implying an assessment was done, but the process to minimize error is not clearly reported.	
DOMAIN 4: SYNTHESIS AND FINDINGS	4.1 Did the synthesis include all studies that it should?	Y	Based on the studies identified and included, the synthesis includes all eligible studies that passed the inclusion criteria.	LOW
	4.2 Were all pre-defined analyses reported or departures explained?	PY	The main pre-defined analyses (meta-analysis on each marker, subgroup analyses by age, BMI, exercise type, intensity, duration, LVEF) are reported. Sensitivity analyses and publication bias assessments (funnel plots, Egger's test) were conducted. The protocol is registered, but no explicit comparison between planned and executed analyses is provided in the paper to confirm no selective reporting.	
	4.3 Was the synthesis appropriate given the nature of studies?	Y	A random-effects model was appropriately used due to expected clinical and methodological heterogeneity. Standardized Mean Difference (SMD) was used, which is suitable for combining continuous outcomes measured on different scales.	
	4.4 Was between-study variation (heterogeneity) minimal or addressed in the synthesis?	Y	Heterogeneity was assessed (I^2 statistic reported). Significant heterogeneity was found for several outcomes (e.g., TNF-α, hs-CRP). The authors addressed this by using a random-effects model and performing extensive pre-specified subgroup analyses to explore sources of heterogeneity.	
	4.5 Were the findings robust (e.g., funnel plot/sensitivity analyses)?	PY	Sensitivity analyses were conducted. Funnel plots and Egger's tests were performed to assess publication bias for each outcome. Asymmetry was found for some markers (IL-6, IL-1β, hs-CRP), which the authors acknowledge as a limitation. This indicates the findings for these outcomes may not be robust.	
	4.6 Were biases in primary studies minimal or addressed in the synthesis?	PY	The risk of bias in primary studies was assessed with the PEDro scale (Table 1). While most studies scored as "Low" risk, the tool itself is not the most specific for RCT bias.	

Phase 3: Judging risk of bias

Summarize the concerns identified during the Phase 2 assessment:

Domain	Concern	Evidence / Rationale
1. Eligibility Criteria	LOW	Criteria were pre-specified, appropriate, and sufficiently clear.
2. Identification/Selection	HIGH	no supplementary search methods, potentially insensitive search strategy, and a restrictive English-only language policy.
3. Data Collection/Appraisal	LOW	Data extraction was systematic. The use of the PEDro scale (instead of Cochrane RoB) and unclear reporting on the independence of the quality assessment process introduce some concerns about the rigor of study appraisal.
4. Synthesis and Findings	LOW	Methods were statistically appropriate. Concerns arise from the presence of publication bias for key outcomes and the lack of formal analysis to incorporate the risk of bias from primary studies into the results.

RISK OF BIAS IN THE REVIEW

Signaling Question	Response	Evidence / Rationale
A. Did the interpretation of findings address all of the concerns identified in Domains 1 to 4?	PN	The potential for language bias due to the English-only restriction. The implications of using the PEDro scale instead of a more RCT-specific tool.
B. Was the relevance of identified studies to the review's research question appropriately considered?	Y	The studies included all met the PICO eligibility criteria, which were well-aligned with the research question. The subgroup analyses demonstrate a detailed consideration of the relevance and applicability of the findings.
C. Did the reviewers avoid emphasizing results on the basis of their statistical significance?	Y	The results are presented primarily through effect sizes (SMD) and confidence intervals. The discussion interprets the clinical importance of "small" or "medium" effect sizes as per Cochrane guidelines, rather than over-emphasizing p-values. Conclusions are drawn based on the magnitude and direction of effects.

Y=YES, PY=PROBABLY YES, PN=PROBABLY NO, N=NO, NI=NO INFORMATION

2.11. Detailed Risk of Bias Assessment for the Systematic Review by Konstantina Dragoumani et al. (2023)

Phase 1: Assessing relevance

Category	Target question	Review being assessed
Patients / Population(s)	Obese/overweight children and adolescents (8–18 years old), no severe intervention contraindications.	Obese/overweight children and adolescents (mean age 8–16 years); 649 participants (intervention/control not separately reported) from 11 studies (7 RCTs/3 randomized non-controlled/1 cohort); intervention duration 3–12 months.
Intervention(s)	Lifestyle interventions (diet, exercise) or drugs/supplements, alone or in combination.	Interventions include diet+exercise (10 studies), exercise alone (1 study), plus drugs/supplements (2 studies); 3–12 months, no unified frequency/duration/intensity reporting but all are structured interventions.
Comparator(s)	No intervention, usual care, or non-intervention control.	Controls include no intervention, usual care, or standard monitoring; no concurrent structured interventions.
Outcome(s)	Somatic indicators of stress, inflammation (CRP, IL-6, TNF-α, leptin, adiponectin) and dysmetabolism (glucose, insulin, HOMA-IR, 血脂，blood pressure).	All pre-defined outcomes reported; significant reductions in body fat, insulin, DBP, and increased adiponectin; trend toward reduced leptin/glucose/HOMA-IR; no significant changes in BMI/WC; IL-6/TNF-α results inconsistent.

Phase 2: Identifying concerns with the review process

Domain	Signaling Questions	Response	Evidence / Rationale	Risk Bias Assessment
DOMAIN 1: STUDY ELIGIBILITY CRITERIA	1.1 Did the review adhere to pre-defined objectives and eligibility criteria?	PY	The review states clear objectives and pre-defined PICOs (Section 2.2, Figure 2). The PRISMA flowchart (Fig 2) demonstrates adherence.	LOW
	1.2 Were the eligibility criteria appropriate for the review question?	Y	Criteria focus on intervention studies in obese/overweight children/adolescents measuring relevant biomarkers, aligning with the research question (Section 2.2).	
	1.3 Were eligibility criteria unambiguous?	PY	Criteria are listed but lack operational detail. E.g., "obesity" is not defined by specific BMI cut-offs. "Controlled or not controlled randomized and nonrandomized trials" is broad and potentially ambiguous regarding quality thresholds.	
	1.4 Were all restrictions in eligibility criteria based on study characteristics appropriate?	PY	Restrictions include: mean age ≤18, intervention studies, English language. These seem reasonable for the research question, though the inclusion of a single cohort study (Mietus-Snyder 2020) among RCTs may introduce design heterogeneity.	
	1.5 Were any restrictions in eligibility criteria based on sources of information appropriate?	PY	Restriction to English language only is stated in the criteria (Section 2.2). While common, this can introduce language bias	
DOMAIN 2: IDENTIFICATION AND SELECTION OF STUDIES	2.1 Did the search include an appropriate range of databases/electronic sources?	Y	Three major databases searched: Web of Science, Scopus, PubMed (Section 2.1).	HIGH
	2.2 Were methods additional to database searching used to identify relevant reports?	Y	Yes. Reference lists of relevant articles were checked. Conference abstracts and trial registries (ICTRP, ClinicalTrials.gov) were searched (implied by search strings in Table A1).	
	2.3 Were the terms and structure of the search strategy likely to retrieve as many eligible studies as possible?	Y	Search strategies are provided in Appendix (Tables A1, A2). They use comprehensive keyword combinations (PICO elements, synonyms, truncation).	
	2.4 Were restrictions based on date, publication format, or language appropriate?	PY	The review states: "Initially, no language or time frame restriction was applied" (Section 2.1). However, the eligibility criteria explicitly require trials "published in English" (Section 2.2).	
	2.5 Were efforts made to minimise error in selection of studies?	PN	Implied by PRISMA adherence. Figure 2 suggests a structured screening process, though the text does not explicitly state dual independent screening for title/abstract.	
DOMAIN 3: DATA COLLECTION AND STUDY APPRAISAL	3.1 Were efforts made to minimise error in data collection?	PY	Stated: "Another author (T.A.) compared the extraction forms, all differences were reviewed, discussed, and corrected." (Section 2.3). Suggests independent extraction and consensus, but details on initial extraction process are limited.	HIGH
	3.2 Were sufficient study characteristics available for interpretation?	Y	Table 2 provides extensive study characteristics (population, age, gender, intervention, duration, trial type).	
	3.3 Were all relevant study results collected for use in the synthesis?	Y	Table 3 shows which biomarkers were extracted from each study. All reported relevant quantitative data has been collected.	
	3.4 Was risk of bias (or methodological quality) formally assessed using appropriate	N	The review does not mention assessing the risk of bias or methodological quality of the included primary studies.	

	criteria?			
	3.5 Were efforts made to minimise error in risk of bias assessment?	N	Not applicable, as risk of bias was not assessed	
DOMAIN 4: SYNTHESIS AND FINDINGS	4.1 Did the synthesis include all studies that it should?	PY	The meta-analysis appears to include all eligible studies from the 11 selected. One study (Farpour-Lambert 2019) had insufficient data for meta-analysis but was included in qualitative synthesis (Section 3.1).	HIGH
	4.2 Were all pre-defined analyses reported or departures explained?	PY	Pre-specified outcomes are listed. All main outcomes are reported in Table 4. Subgroup analyses are reported. The use of cumulative meta-analysis was not pre-specified but is noted.	
	4.3 Was the synthesis appropriate given the nature of studies?	Y	A random-effects model was chosen appropriately due to expected heterogeneity (Section 2.4). Subgroup analyses were conducted. Forest and funnel plots were generated.	
	4.4 Was between-study variation (heterogeneity) minimal or addressed in the synthesis?	Y	Heterogeneity was assessed using I^2 statistic (Table 4, all analyses). High heterogeneity was present for many outcomes. Authors acknowledge heterogeneity and used a random-effects model.	
	4.5 Were the findings robust (e.g., funnel plot/sensitivity analyses)?	Y	Sensitivity analyses were performed. Funnel plots were generated to assess publication bias (Section 2.4).	
	4.6 Were biases in primary studies minimal or addressed in the synthesis?	N	Biases in primary studies were not assessed, therefore they could not be addressed in the synthesis.	

Phase 3: Judging risk of bias

Summarize the concerns identified during the Phase 2 assessment:

Domain	Concern	Evidence / Rationale
1. Eligibility Criteria	HIGH	Ambiguous criteria for "obesity," inclusion of diverse and potentially lower-quality study designs (cohort, non-controlled), and an explicit, inappropriate "English-only" publication restriction.
2. Identification/Selection	LOW	Comprehensive search strategy conflicted by a language restriction applied at the eligibility stage, creating methodological inconsistency.
3. Data Collection/Appraisal	HIGH	No assessment of risk of bias in included primary studies.
4. Synthesis and Findings	HIGH	Synthesis incorporates studies of unknown bias.

RISK OF BIAS IN THE REVIEW

Signaling Question	Response	Evidence / Rationale
A. Did the interpretation of findings address all of the concerns identified in Domains 1 to 4?	N	The discussion (Section 4) mentions limitations like small sample sizes, varying intervention durations, and heterogeneity. However, it does not acknowledge the major limitation of not assessing the risk of bias in the included studies (Domain 3). It also fails to discuss the potential impact of the "English-only" restriction (Domain 1) or the methodological inconsistency in the search/selection process (Domain 2).
B. Was the relevance of identified studies to the review's research question appropriately considered?	PY	The included studies all involve obese/overweight youth and measure the targeted biomarkers. The inclusion of one cohort study and several non-controlled RCTs, while a concern for bias (Domain 1), is at least acknowledged in the characteristics table.
C. Did the reviewers avoid emphasizing results on the basis of their statistical significance?	Y	The results and discussion present both statistically significant and non-significant findings. The abstract and conclusions note improvements in some markers but also highlight non-significant changes in others.

Y=YES, PY=PROBABLY YES, PN=PROBABLY NO, N=NO, NI=NO INFORMATION

2.12. Detailed Risk of Bias Assessment for the Systematic Review by Liang Tan et al. (2023)

Phase 1: Assessing relevance

Category	Target question	Review being assessed
Patients / Population(s)	Postmenopausal women with overweight/obesity (BMI ≥25/30 kg/m²), no severe exercise contraindications.	Postmenopausal overweight/obese women; 2229 participants (1261 intervention/968 control) from 34 RCTs; age 46.7–88.9 years, BMI 25.0–34.8 kg/m², some with T2DM/NAFLD.
Intervention(s)	Exercise interventions (aerobic/AE, resistance/RT, combined/CE) with measurable frequency/duration/intensity (≥8 weeks, 2–5 sessions/week).	Exercise (AE: main type; RT; CE); 8 weeks–12 months, 2–5 sessions/week, 25–75 minutes/session (intensity: 45%–85% HRmax/HRR/1RM, mainly moderate to high intensity).
Comparator(s)	Usual care, no structured exercise, or routine treatment.	Controls include no exercise, routine care, or standard monitoring; no concurrent exercise interventions.
Outcome(s)	Inflammatory markers: CRP, TNF-α, IL-6, adiponectin.	All pre-defined outcomes reported; exercise significantly reduces CRP (MD=-0.59, p<0.00001), TNF-α (MD=-0.65, p<0.00001), IL-6 (MD=-0.48, p<0.00001), and increases adiponectin (MD=0.33, p=0.04); subgroup analysis shows AE is more effective for IL-6/adiponectin, while CE has no significant effect on TNF-α.

Phase 2: Identifying concerns with the review process

Domain	Signaling Questions	Response	Evidence / Rationale	Risk Bias Assessment
DOMAIN 1: STUDY ELIGIBILITY CRITERIA	1.1 Did the review adhere to pre-defined objectives and eligibility criteria?	PY	Protocol registered in PROSPERO (CRD42023422920), but full protocol not provided. Eligibility criteria clearly stated in methods.	LOW
	1.2 Were the eligibility criteria appropriate for the review question?	Y	PICO clearly defined: population, intervention (exercise), comparator (control), outcomes (inflammatory markers).	
	1.3 Were eligibility criteria unambiguous?	Y	Clear inclusion/exclusion criteria provided in Section 2.2.	
	1.4 Were all restrictions in eligibility criteria based on study characteristics appropriate?	PY	Only English-language RCTs included; no justification provided for language restriction. May introduce language bias.	
	1.5 Were any restrictions in eligibility criteria based on sources of information appropriate?	PY	Only published studies included; no search for grey literature mentioned.	
DOMAIN 2: IDENTIFICATION AND SELECTION OF STUDIES	2.1 Did the search include an appropriate range of databases/electronic sources?	Y	Five databases searched: PubMed, Cochrane, Embase, Web of Science, EBSCO.	LOW
	2.2 Were methods additional to database searching used to identify relevant reports?	PY	No mention of hand-searching reference lists, contacting authors, or searching trial registries beyond initial database search.	
	2.3 Were the terms and structure of the search strategy likely to retrieve as many eligible studies as possible?	Y	Search strategy provided (Table S1) with broad terms.	
	2.4 Were restrictions based on date, publication format, or language appropriate?	PY	Only English studies included. Date restriction not explicitly stated.	
	2.5 Were efforts made to minimise error in selection of studies?	Y	Two reviewers independently screened studies using EndNote.Disagreements resolved by discussion/third reviewer.	
DOMAIN 3: DATA COLLECTION AND STUDY APPRAISAL	3.1 Were efforts made to minimise error in data collection?	Y	Two reviewers independently extracted data. Disagreements resolved by discussion.	
	3.2 Were sufficient study characteristics available for interpretation?	Y	Table S3 provides detailed study characteristics (participants, interventions, outcomes).	
	3.3 Were all relevant study results collected for use in the synthesis?	Y	All pre-specified outcomes (CRP, TNF-伪, IL-6, adiponectin) extracted and analyzed.	
	3.4 Was risk of bias (or methodological quality) formally assessed using appropriate criteria?	Y	Cochrane Risk of Bias Tool used; results shown in Figures 2 & 3.	
	3.5 Were efforts made to minimise error in risk of bias assessment?	Y	Two reviewers independently assessed bias.Conflicts resolved by discussion/third reviewer.	

DOMAIN 4: SYNTHESIS AND FINDINGS	4.1 Did the synthesis include all studies that it should?	Y	All eligible studies included in meta-analysis; exclusion of 11 studies with insufficient data justified.	LOW
	4.2 Were all pre-defined analyses reported or departures explained?	PY	Subgroup analyses by exercise type reported; no protocol deviations mentioned.	
	4.3 Was the synthesis appropriate given the nature of studies?	Y	Random-effects model used. Subgroup analyses performed. Heterogeneity assessed.	
	4.4 Was between-study variation (heterogeneity) minimal or addressed in the synthesis?	PY	High heterogeneity in some analyses (e.g., CRP, TNF-α); explored via subgroup analysis but not fully resolved.	
	4.5 Were the findings robust (e.g., funnel plot/sensitivity analyses)?	Y	Funnel plots, Egger's test, and sensitivity analyses performed (Fig. S1, S2.Table S4).	
	4.6 Were biases in primary studies minimal or addressed in the synthesis?	PY	Risk of bias assessed and considered in discussion; no quantitative adjustment for bias.	

Phase 3: Judging risk of bias

Summarize the concerns identified during the Phase 2 assessment:

Domain	Concern	Evidence / Rationale
1. Eligibility Criteria	LOW	Clear criteria but language/publication restrictions may bias inclusion.
2. Identification/Selection	LOW	Limited search methods and language bias may have omitted relevant studies.
3. Data Collection/Appraisal	LOW	Rigorous extraction and bias assessment methods.
4. Synthesis and Findings	LOW	Appropriate synthesis with sensitivity analyses, though heterogeneity not fully resolved.

RISK OF BIAS IN THE REVIEW

Signaling Question	Response	Evidence / Rationale
A. Did the interpretation of findings address all of the concerns identified in Domains 1 to 4?	Y	The risk of bias for all four domains in Phase 2 is "low".
B. Was the relevance of identified studies to the review's research question appropriately considered?	Y	Studies directly matched PICO; relevance discussed in results and discussion.
C. Did the reviewers avoid emphasizing results on the basis of their statistical significance?	Y	Both statistical and clinical significance discussed

Y=YES, PY=PROBABLY YES, PN=PROBABLY NO, N=NO, NI=NO INFORMATION

2.13. Detailed Risk of Bias Assessment for the Systematic Review by Sameer Badri Al-Mhanna et al. (2023)

Phase 1: Assessing relevance

Category	Target question	Review being assessed
Patients / Population(s)	Obese/overweight patients with type 2 diabetes (T2DM, BMI ≥25/30 kg/m²), no severe intervention contraindications.	Obese/overweight T2DM patients; 1192 participants from 13 studies (15 reports); BMI 29.7–37.5 kg/m², intervention duration 8 weeks–2 years; some with hypertension/ischemic heart disease.
Intervention(s)	Combined aerobic exercise and diet (AEDT), with measurable frequency/duration/intensity.	AEDT (aerobic exercise + dietary intervention); aerobic exercise: 3–5 sessions/week, 30–50 min/session (intensity: 55–80% VO_2max/HRmax); diet: low-calorie/low-fat/high-fiber, 1200–2000 kcal/day.
Comparator(s)	Standard treatment (usual lifestyle, no structured exercise/diet intervention).	Controls include usual care, routine lifestyle advice, no exercise/diet intervention; no concurrent structured interventions.
Outcome(s)	Cardiometabolic health indicators: anthropometrics (BMI, weight, WC), blood pressure, lipids, glucose metabolism (HbA1c, FBG), inflammation markers (CRP, IL-6, TNF-α), adipokines (adiponectin, leptin).	All pre-defined outcomes reported; AEDT significantly improves BMI (SMD=-0.33, p=0.0001)、weight (SMD=-2.69, p=0.01)、SBP/DBP、TC/TG、HbA1c (SMD=-0.52, p=0.01)、FBG、FI、IL-6、CRP; no significant effects on fat mass、HDL-C、LDL-C、TNF-α.

Phase 2: Identifying concerns with the review process

Domain	Signaling Questions	Response	Evidence / Rationale	Risk Bias Assessment
DOMAIN 1: STUDY ELIGIBILITY CRITERIA	1.1 Did the review adhere to pre-defined objectives and eligibility criteria?	Y	The protocol was registered in PROSPERO (CRD42023390330), indicating pre-defined objectives and criteria. The published methods align with the PICOS framework described in the protocol.	LOW
	1.2 Were the eligibility criteria appropriate for the review question?	Y	Criteria clearly defined	
	1.3 Were eligibility criteria unambiguous?	Y	Clear inclusion/exclusion criteria provided. Definitions for population, intervention, outcomes, and study design are explicit.	
	1.4 Were all restrictions in eligibility criteria based on study characteristics appropriate?	Y	Restrictions (RCTs/CCTs, no language/date limits) are appropriate and justified to focus on relevant evidence.	
	1.5 Were any restrictions in eligibility criteria based on sources of information appropriate?	Y	No restrictions on publication status or language; appropriate to minimize publication bias.	
DOMAIN 2: IDENTIFICATION AND SELECTION OF STUDIES	2.1 Did the search include an appropriate range of databases/electronic sources?	PY	Six databases searched (PubMed,Web of Science,Scopus,Google Scholar,Cochrane Library,Science Direct）.Grey literature beyond conference abstracts were systematically searched.	LOW
	2.2 Were methods additional to database searching used to identify relevant reports?	Y	Reference lists of included studies and relevant reviews were checked.	
	2.3 Were the terms and structure of the search strategy likely to retrieve as many eligible studies as possible?	PY	Search strategy (Table S1) is overly simplistic (e.g., single-line Boolean strings in some databases). Key synonyms for T2DM (e.g., "non-insulin dependent diabetes"), obesity ("overweight"), or exercise modalities may be missing. Search strategy lacks transparency for some databases (e.g., Scopus, Science Direct).	
	2.4 Were restrictions based on date, publication format, or language appropriate?	Y	No restrictions on date, format, or language. Appropriate.	
	2.5 Were efforts made to minimise error in selection of studies?	Y	Four authors independently screened titles/abstracts and full texts, with a fifth resolving conflicts.	
DOMAIN 3: DATA COLLECTION AND STUDY APPRAISAL	3.1 Were efforts made to minimise error in data collection?	Y	Two authors independently extracted data using a pre-defined form.Conflicts resolved by discussion/third author.	LOW
	3.2 Were sufficient study characteristics available for interpretation?	Y	Table S3 provides extensive details on participants, interventions, comparators, outcomes, and study context.	
	3.3 Were all relevant study results collected for use in the synthesis?	Y	Data for all pre-specified primary and secondary outcomes appear to have been extracted, as shown in forest plots and Table S4.	
	3.4 Was risk of bias (or methodological quality) formally assessed using appropriate criteria?	Y	Cochrane Risk of Bias tool (RoB 1) was used for RCTs, appropriate for intervention reviews. Detailed judgments per study in Table S2.	
	3.5 Were efforts made to minimise error in	Y	Two authors independently assessed RoB, with disagreements resolved by a third. Process described,	

	risk of bias assessment?			
DOMAIN 4: SYNTHESIS AND FINDINGS	4.1 Did the synthesis include all studies that it should?	Y	All included studies appear in relevant syntheses.	LOW
	4.2 Were all pre-defined analyses reported or departures explained?	Y	All planned outcomes (primary & secondary) from PICOS were reported in results. Subgroup analyses based on comorbidities were performed as planned.	
	4.3 Was the synthesis appropriate given the nature of studies?	Y	Random-effects meta-analysis was appropriate given anticipated clinical heterogeneity.	
	4.4 Was between-study variation (heterogeneity) minimal or addressed in the synthesis?	PY	High heterogeneity was acknowledged but not adequately investigated. Sensitivity analyses excluding high RoB studies were mentioned but not shown for all outcomes.	
	4.5 Were the findings robust (e.g., funnel plot/sensitivity analyses)?	Y	Publication bias assessed via funnel plot/Egger's test for outcomes with ≥10 studies (HDL-C, TG, TC). Sensitivity analyses are mentioned	
	4.6 Were biases in primary studies minimal or addressed in the synthesis?	Y	Table S2 shows that many included studies had "High" or "Unclear" risk of bias in key domains. The synthesis pooled these studies without statistical adjustment. Sensitivity analysis based on RoB is mentioned.	

Phase 3: Judging risk of bias

Summarize the concerns identified during the Phase 2 assessment:

Domain	Concern	Evidence / Rationale
1. Eligibility Criteria	LOW	Clear, pre-specified, and appropriate criteria
2. Identification/Selection	LOW	Search strategy was limited, increasing the risk of missing relevant studies.
3. Data Collection/Appraisal	LOW	Robust and systematic processes for data extraction and risk of bias assessment.
4. Synthesis and Findings	LOW	Sensitivity analysis based on RoB is mentioned.

RISK OF BIAS IN THE REVIEW

Signaling Question	Response	Evidence / Rationale
A. Did the interpretation of findings address all of the concerns identified in Domains 1 to 4?	Y	The risk of bias for all four domains in Phase 2 is "low".
B. Was the relevance of identified studies to the review's research question appropriately considered?	Y	The PICOS of included studies match the review question. The subgroup analysis based on comorbidities shows consideration of clinical relevance.
C. Did the reviewers avoid emphasizing results on the basis of their statistical significance?	PY	The abstract and conclusions emphasize "significant improvements" ($p<0.05$) for many outcomes. While non-significant findings are reported, the narrative focus is on positive, statistically significant results, potentially leading to overinterpretation given the methodological concerns (high heterogeneity, risk of bias in primary studies).

Y=YES, PY=PROBABLY YES, PN=PROBABLY NO, N=NO, NI=NO INFORMATION

2.14. Detailed Risk of Bias Assessment for the Systematic Review by Sebastian Del Rosso et al. (2023)

Phase 1: Assessing relevance

Category	Target question	Review being assessed
Patients / Population(s)	Individuals with overweight/obesity (BMI ≥25/30 kg/m²) and cardiometabolic diseases (T2DM/MetS), no severe exercise contraindications.	Cytokines (CRP, IL-6, TNF-α, IL-18, etc.) and adipokines (leptin, adiponectin, etc.), plus glucose/lipid metabolism markers.
Intervention(s)	Long-term exercise training (aerobic/AeT, resistance/RT, combined/COMB, physical activity/PA) with measurable parameters.	AeT (68 studies): 3–5 sessions/week, 30–60 min/session (50–85% VO_2max/HRmax); RT (41 studies): 2–3 sessions/week, 3–4 sets×8–15 reps (40–95% 1RM); COMB (24 studies): AeT+RT combination; PA (10 studies): leisure/spontaneous activity.
Comparator(s)	No structured exercise, usual care, or non-exercise control.	Controls include no exercise, routine lifestyle advice, or standard monitoring; no concurrent exercise interventions.
Outcome(s)	Cytokines (CRP, IL-6, TNF-α, IL-18, etc.) and adipokines (leptin, adiponectin, etc.), plus glucose/lipid metabolism markers.	All pre-defined outcomes reported; all exercise modes (except PA) reduce CRP (Hedges's g=-0.236, p<0.001)、IL-6 (-0.276, p=0.001)、TNF-α (-0.491, p<0.001)、leptin (-0.571, p<0.001); COMB superior to AeT for CRP; ΔVO_2max moderates CRP/IL-6/TNF-α.

Phase 2: Identifying concerns with the review process

Domain	Signaling Questions	Response	Evidence / Rationale	Risk Bias Assessment
DOMAIN 1: STUDY ELIGIBILITY CRITERIA	1.1 Did the review adhere to pre-defined objectives and eligibility criteria?	Y	Registered in PROSPERO (CRD42021286663); pre-specified PICO framework; eligibility criteria consistently applied in study selection.	LOW
	1.2 Were the eligibility criteria appropriate for the review question?	Y	Criteria targeted overweight/obese populations with cardiometabolic conditions; interventions (4 exercise modes) directly address the research question on inflammatory markers; outcomes align with cytokines/adipokines.	
	1.3 Were eligibility criteria unambiguous?	Y	Clear inclusion/exclusion criteria: RCT design, ≥4-week duration, human studies, no concurrent supplements, pre/post inflammatory marker measurements.	
	1.4 Were all restrictions in eligibility criteria based on study characteristics appropriate?	Y	o inappropriate restrictions on publication year (2000–2022), sample size, or outcome measurement; focus on RCTs is justified for intervention effect evaluation.	
	1.5 Were any restrictions in eligibility criteria based on sources of information appropriate?	Y	No restrictions on publication status (included unpublished/ongoing studies via ICTRP/ClinicalTrials.gov) or language	
DOMAIN 2: IDENTIFICATION AND SELECTION OF STUDIES	2.1 Did the search include an appropriate range of databases/electronic sources?	Y	Searched Medline, Cochrane, Embase (core databases for clinical/epidemiological research); supplementary search of relevant review references.	LOW
	2.2 Were methods additional to database searching used to identify relevant reports?	Y	Hand-searched conference abstracts, reference lists of included studies, and clinical trial registries (ICTRP, ClinicalTrials.gov) to capture unpublished/ongoing research.	
	2.3 Were the terms and structure of the search strategy likely to retrieve as many eligible studies as possible?	Y	Used MeSH terms + keywords aligned with PICO; search strategy detailed in Figure 1; reproducible and comprehensive for exercise, inflammatory markers, and target populations.	
	2.4 Were restrictions based on date, publication format, or language appropriate?	Y	No language restrictions. Date range (2000–2022) justified to capture contemporary evidence. No arbitrary publication format limits.	
	2.5 Were efforts made to minimise error in selection of studies?	Y	Two independent reviewers screened titles/abstracts and full texts; discrepancies resolved by a third reviewer (NRP); duplicate removal via EndNote.	
DOMAIN 3: DATA COLLECTION AND STUDY APPRAISAL	3.1 Were efforts made to minimise error in data collection?	Y	Two independent reviewers extracted data into a customized database, third reviewer resolved discrepancies	LOW
	3.2 Were sufficient study characteristics available for interpretation?	Y	Table S1 summarizes key study details (sample size, age, BMI, intervention type/frequency/duration/intensity, outcomes); subgroup analyses (sex, age, body composition) provide additional context.	
	3.3 Were all relevant study results collected for use in the synthesis?	Y	Extracted both primary and secondary outcomes,reported pre/post changes and between-group comparisons.	
	3.4 Was risk of bias (or methodological quality) formally assessed using appropriate criteria?	Y	Used Downs and Black 26-item checklist (methodological quality) and Cochrane RoB 2 tool (bias risk for RCTs); comprehensive assessment of randomization, blinding, missing data, etc.	

	3.5 Were efforts made to minimise error in risk of bias assessment?	Y	Three independent reviewers conducted methodological quality assessment; two reviewers assessed bias risk for main outcomes; consensus achieved via discussion/third-party arbitration.	
DOMAIN 4: SYNTHESIS AND FINDINGS	4.1 Did the synthesis include all studies that it should?	Y	PRISMA flow diagram (Figure 3) documents full study selection process; 106 eligible studies included after screening 1,827 records; no evidence of arbitrary exclusion of eligible studies.	LOW
	4.2 Were all pre-defined analyses reported or departures explained?	Y	Conducted pre-specified meta-analysis, subgroup analysis, meta-regression, and network meta-analysis; all deviations were explained.	
	4.3 Was the synthesis appropriate given the nature of studies?	Y	Used DerSimonian-Laird random-effects model,network meta-analysis for head-to-head exercise mode comparisons,data transformation per Cochrane guidelines.	
	4.4 Was between-study variation (heterogeneity) minimal or addressed in the synthesis?	Y	Assessed heterogeneity via, explored sources via subgroup/meta-regression, acknowledged high heterogeneity and justified random-effects model use.	
	4.5 Were the findings robust (e.g., funnel plot/sensitivity analyses)?	Y	Sensitivity analysis.Egger test + funnel plots for publication bias. Meta-regression identified moderators.Findings consistent across subgroups.	
	4.6 Were biases in primary studies minimal or addressed in the synthesis?	Y	Excluded high bias risk studies in sensitivity analysis; adjusted for confounding via subgroup/meta-regression; discussed impact of primary study bias on overall findings in the discussion section.	

Phase 3: Judging risk of bias

Summarize the concerns identified during the Phase 2 assessment:

Domain	Concern	Evidence / Rationale
1. Eligibility Criteria	LOW	Clear, appropriate, and pre-specified criteria; consistent application.
2. Identification/Selection	LOW	Comprehensive search strategy; rigorous dual screening process.
3. Data Collection/Appraisal	LOW	Dual data extraction/bias assessment; sufficient study characteristic reporting.
4. Synthesis and Findings	LOW	Appropriate statistical methods; thorough heterogeneity and robustness testing.

RISK OF BIAS IN THE REVIEW

Signaling Question	Response	Evidence / Rationale
A. Did the interpretation of findings address all of the concerns identified in Domains 1 to 4?	Y	Discussion section acknowledges limitations and their impact,links findings to primary study bias and methodological choices.
B. Was the relevance of identified studies to the review's research question appropriately considered?	Y	All included studies align with PICO, subgroup analyses focus on populations/interventions relevant to the research question, comparisons between exercise modes address the core objective.
C. Did the reviewers avoid emphasizing results on the basis of their statistical significance?	Y	Reports both statistically significant and non-significant findings, discusses clinical relevance alongside statistical significance, no overstatement of positive results.

Y=YES, PY=PROBABLY YES, PN=PROBABLY NO, N=NO, NI=NO INFORMATION

2.15. Detailed Risk of Bias Assessment for the Systematic Review by Gholam Rasul Mohammad Rahimi et al. (2022)

Phase 1: Assessing relevance

Category	Target question	Review being assessed
Patients / Population(s)	Overweight/obese adults with metabolic syndrome (MS, BMI ≥25/30 kg/m²), no severe intervention contraindications.	Overweight/obese MS adults; 1246 participants (638 intervention/608 control) from 6 RCTs (7 arms); mean age 57.4±6.7 years, BMI 28.4±7.6 kg/m²; no severe contraindications reported.
Intervention(s)	Lifestyle intervention (combined diet + exercise) with measurable duration/frequency (≥4 weeks).	Diet + exercise intervention; 6–12 months duration, 2–5 sessions/week; diet (low-fat/hypocaloric, 1500–calorie-controlled), exercise (aerobic/walking/yoga, moderate intensity, 30–60 min/session).
Comparator(s)	Usual care, no structured diet/exercise intervention.	Controls include usual care, routine lifestyle advice; no concurrent structured diet/exercise interventions.
Outcome(s)	Inflammatory markers (CRP, IL-6, adiponectin) and waist circumference (WC).	All pre-defined outcomes reported; lifestyle intervention significantly reduces CRP (WMD=-0.52 mg/ml, p<0.00001)、IL-6 (WMD=-0.50 pg/ml, p<0.00001)、WC (WMD=-3.12 cm, p<0.0001), increases adiponectin (WMD=0.81 μg/ml, p<0.00001).

Phase 2: Identifying concerns with the review process

Domain	Signaling Questions	Response	Evidence / Rationale	Risk Bias Assessment
DOMAIN 1: STUDY ELIGIBILITY CRITERIA	1.1 Did the review adhere to pre-defined objectives and eligibility criteria?	PY	The protocol was not prospectively registered (e.g., PROSPERO), and no pre-published protocol was cited. Eligibility criteria were described in the methods.Followed PRISMA guidelines	LOW
	1.2 Were the eligibility criteria appropriate for the review question?	Y	Criteria were clearly aligned with the PICO: RCTs, overweight/obese adults with Metabolic Syndrome, lifestyle interventions (diet + exercise) vs. usual care, outcomes of inflammatory markers and waist circumference.	
	1.3 Were eligibility criteria unambiguous?	Y	Criteria were generally clear	
	1.4 Were all restrictions in eligibility criteria based on study characteristics appropriate?	Y	Restrictions were appropriate: RCTs only, adults ≥18 years, intervention duration >4 weeks, no language or date restrictions were applied (appropriate).	
	1.5 Were any restrictions in eligibility criteria based on sources of information appropriate?	PY	The search was limited to English-language publications. No justification was provided for excluding non-English studies.	
DOMAIN 2: IDENTIFICATION AND SELECTION OF STUDIES	2.1 Did the search include an appropriate range of databases/electronic sources?	PY	Six databases were searched . However, no specific mention of searching clinical trial registries for ongoing/unpublished studies, and grey literature searching was not systematic.	LOW
	2.2 Were methods additional to database searching used to identify relevant reports?	PY	Only reference lists of relevant reviews and primary studies were examined. There was no mention of contacting experts, hand-searching key journals, or searching conference abstracts systematically.	
	2.3 Were the terms and structure of the search strategy likely to retrieve as many eligible studies as possible?	PY	Supplemental material includes a complex search strategy with MeSH and free-text terms covering key concepts (exercise, diet, metabolic syndrome, inflammation, etc.). The strategy appears comprehensive for the listed databases.	
	2.4 Were restrictions based on date, publication format, or language appropriate?	PY	The search was restricted to English language without justification, which is a potential source of bias. No date restrictions were applied. Publication format restrictions were not specified.	
	2.5 Were efforts made to minimise error in selection of studies?	Y	Two reviewers independently screened titles/abstracts and full texts. Disagreements were resolved by discussion or with a third reviewer.	
DOMAIN 3: DATA COLLECTION AND STUDY APPRAISAL	3.1 Were efforts made to minimise error in data collection?	Y	Two reviewers independently extracted data using a pre-designed form. Disagreements were resolved by discussion or with a third reviewer.	LOW
	3.2 Were sufficient study characteristics available for interpretation?	Y	Table 1 provides detailed characteristics of included studies, sufficient for interpretation.	
	3.3 Were all relevant study results collected for use in the synthesis?	PY	Pre- and post-intervention means/SDs were extracted for all pre-specified outcomes. For studies with multiple intervention arms, the control group was split appropriately.	
	3.4 Was risk of bias (or methodological quality) formally assessed using appropriate criteria?	PY	The TESTEX scale was used, which is a general tool for exercise training studies, not a tool specifically designed to assess risk of bias in RCTs. TESTEX evaluates reporting and study quality, not bias domains like randomization, blinding, attrition.	
	3.5 Were efforts made to minimise error in risk of bias assessment?	PY	Two reviewers independently assessed study quality using TESTEX. Disagreements were resolved by consensus or a third reviewer.	

DOMAIN 4: SYNTHESIS AND FINDINGS	4.1 Did the synthesis include all studies that it should?	Y	All included studies contributing to each outcome appear to have been included in the respective meta-analyses.	HIGH
	4.2 Were all pre-defined analyses reported or departures explained?	NI	There is no pre-published protocol or registered analysis plan. It is unclear if all pre-planned analyses (e.g., specific subgroup analyses) were conducted and reported.	
	4.3 Was the synthesis appropriate given the nature of studies?	PY	A random-effects model was used when substantial heterogeneity was present ($I^2 > 50\%$).	
	4.4 Was between-study variation (heterogeneity) minimal or addressed in the synthesis?	PN	High statistical heterogeneity was present for CRP ($I^2 = 98\%$) and moderate for WC ($I^2 = 72\%$). The authors acknowledged this but performed only limited exploration. The causes of high heterogeneity were not adequately investigated .	
	4.5 Were the findings robust (e.g., funnel plot/sensitivity analyses)?	PN	Funnel plots (Egger plots) showed evidence of publication bias for most outcomes (Supplementary Figures). Sensitivity analyses were performed (e.g., excluding high RoB studies) but were limited. The high heterogeneity and publication bias threaten the robustness of the pooled estimates.	
	4.6 Were biases in primary studies minimal or addressed in the synthesis?	PY	The primary studies' risk of bias was not assessed with a proper tool	

Phase 3: Judging risk of bias

Summarize the concerns identified during the Phase 2 assessment:

Domain	Concern	Evidence / Rationale
1. Eligibility Criteria	LOW	Lack of prospective protocol registration
2. Identification/Selection	HIGH	Lack of systematic grey literature/search of trial registries; reliance on reference checking only; English-language restriction.
3. Data Collection/Appraisal	HIGH	Use of an inappropriate tool for assessing risk of bias in RCTs, failing to properly evaluate key bias domains .
4. Synthesis and Findings	HIGH	High/unexplained heterogeneity for key outcomes; evidence of publication bias; inadequate investigation of robustness; inability to address primary study bias due to flawed appraisal.

RISK OF BIAS IN THE REVIEW

Signaling Question	Response	Evidence / Rationale
A. Did the interpretation of findings address all of the concerns identified in Domains 1 to 4?	N	The discussion acknowledges some limitations (small sample size, moderate quality of RCTs, publication bias, heterogeneity) but does not adequately address the core methodological flaws: the impact of language bias, the consequences of using an inappropriate quality assessment tool, or the potential for missed studies due to incomplete searching.
B. Was the relevance of identified studies to the review's research question appropriately considered?	Y	The included studies all matched the PICO framework, and their relevance is clear from the characteristics table.
C. Did the reviewers avoid emphasizing results on the basis of their statistical significance?	PY	The conclusions focus on the direction and magnitude of effect sizes (WMDs) and their clinical significance, not solely on p-values. Confidence intervals are consistently reported.

Y=YES, PY=PROBABLY YES, PN=PROBABLY NO, N=NO, NI=NO INFORMATION

2.16. Detailed Risk of Bias Assessment for the Systematic Review by Haotian Zhao et al. (2022)

Phase 1: Assessing relevance

Category	Target question	Review being assessed
Patients / Population(s)	Obese adolescents (13–18 years old) defined by WHO or national standards, no metabolic/cardiovascular diseases, no severe contraindications to exercise.	Obese adolescents (13–18 years old); 781 participants from 14 randomized controlled trials (RCTs); balanced gender ratio, mean age 12.1–17.0 years; no concurrent metabolic/cardiovascular diseases per inclusion criteria.
Intervention(s)	Different training modalities (aerobic [AT], resistance [RT], combined [AT+RT], high-intensity interval training [HIIT]) with measurable frequency/duration (6–24 weeks, 2–6 sessions/week).	AT (7 studies): 3–6 sessions/week, 8–24 weeks; RT (2 studies): 3 sessions/week, 8 weeks; AT+RT (9 studies): 2–3 sessions/week, 8–24 weeks; HIIT (1 study): 2 sessions/week, 12 weeks; all moderate-to-high intensity.
Comparator(s)	No structured training, usual lifestyle.	Controls maintained usual lifestyle without structured exercise; no concurrent interventions affecting inflammatory markers.
Outcome(s)	Inflammatory markers: interleukin-6 (IL-6), tumor necrosis factor-α (TNF-α), C-reactive protein (CRP).	All pre-defined outcomes reported; AT+RT significantly reduces CRP (SMD=-0.60, p=0.02) and tends to reduce IL-6 (SMD=-0.67, p=0.06); AT reduces CRP (SMD=-0.35, p=0.03); no training modality affects TNF-α (pooled SMD=0.34, p=0.03).

Phase 2: Identifying concerns with the review process

Domain	Signaling Questions	Response	Evidence / Rationale	Risk Bias Assessment
DOMAIN 1: STUDY ELIGIBILITY CRITERIA	1.1 Did the review adhere to pre-defined objectives and eligibility criteria?	PY	The review states it was conducted "strictly in accordance with PRISMA guidelines." Inclusion/exclusion criteria are clearly listed in Section 2.2. However, no explicit reference to a published or registered protocol is made, though the methods suggest criteria were pre-defined.	LOW
	1.2 Were the eligibility criteria appropriate for the review question?	Y	Criteria directly address the review question	
	1.3 Were eligibility criteria unambiguous?	Y	Inclusion/exclusion criteria are clearly listed with specific details. "Overweight/obesity" definition refers to WHO or national standards, which is reasonable.	
	1.4 Were all restrictions in eligibility criteria based on study characteristics appropriate?	Y	No restrictions on publication date, sample size, or study quality are mentioned,Language restriction is not mentioned	
	1.5 Were any restrictions in eligibility criteria based on sources of information appropriate?	Y	The review excluded "conference abstracts, dissertations, case studies, reviews, and other gray literature,"	
DOMAIN 2: IDENTIFICATION AND SELECTION OF STUDIES	2.1 Did the search include an appropriate range of databases/electronic sources?	PY	Databases searched: CNKI, Wanfang, PubMed, Web of Science, EBSCO. These cover major biomedical and Chinese databases. However, no mention of searching trial registries	HIGH
	2.2 Were methods additional to database searching used to identify relevant reports?	PY	The authors state: "tracking the relevant literature references." No mention of contacting experts, hand-searching journals, or searching gray literature beyond reference lists.	
	2.3 Were the terms and structure of the search strategy likely to retrieve as many eligible studies as possible?	PN	Search terms are listed broadly , but no full search strategy is provided in the paper or supplement, limiting reproducibility.	
	2.4 Were restrictions based on date, publication format, or language appropriate?	PN	No date restriction mentioned . Excluded gray literature, which may miss unpublished data. No language restriction stated.	
	2.5 Were efforts made to minimise error in selection of studies?	Y	Two reviewers independently screened titles/abstracts and full texts, disagreements resolved by consensus.	
DOMAIN 3: DATA COLLECTION AND STUDY APPRAISAL	3.1 Were efforts made to minimise error in data collection?	Y	Two reviewers independently screened titles/abstracts and full texts, disagreements resolved by consensus.	LOW
	3.2 Were sufficient study characteristics available for interpretation?	Y	Table 1 provides detailed study characteristics (author, year, sample size, age, training mode, frequency, duration, outcomes).	
	3.3 Were all relevant study results collected for use in the synthesis?	Y	Outcomes pre-specified (IL-6, TNF-伪, CRP) and appear to have been extracted for all included studies where measured.	
	3.4 Was risk of bias (or methodological quality) formally assessed using appropriate criteria?	Y	"Cochrane Risk of Bias (ROB) tool in RevMan5.4.1" used to assess included RCTs. Tool is appropriate for RCTs.	
	3.5 Were efforts made to minimise error in	Y	Two authors independently extracted data using a pre-defined form. Disagreements were resolved through	

	risk of bias assessment?		discussion.	
DOMAIN 4: SYNTHESIS AND FINDINGS	4.1 Did the synthesis include all studies that it should?	PY	All studies meeting inclusion criteria appear to be included in the meta-analyses (Figures 3-5). However, some studies may have contributed to multiple comparisons, which is not clearly addressed.	LOW
	4.2 Were all pre-defined analyses reported or departures explained?	PY	Methods section describes intended subgroup analyses (by training modality) and sensitivity analyses, which were conducted.	
	4.3 Was the synthesis appropriate given the nature of studies?	Y	Random-effects or fixed-effect models used appropriately based on heterogeneity (I^2). Subgroup analyses by training modality are clinically sensible.	
	4.4 Was between-study variation (heterogeneity) minimal or addressed in the synthesis?	Y	Heterogeneity assessed using I^2; random-effects model used when $I^2 > 50\%$. High heterogeneity for CRP (I^2=76%) is discussed in limitations.	
	4.5 Were the findings robust (e.g., funnel plot/sensitivity analyses)?	Y	Sensitivity analyses performed by excluding individual studies, funnel plots presented for IL-6, TNF-α, CRP. Asymmetry in CRP funnel plot is noted and discussed as a limitation	
	4.6 Were biases in primary studies minimal or addressed in the synthesis?	PY	Risk of bias assessment shows 2 low-risk, 7 medium-risk, 5 high-risk studies. Sensitivity analysis excluding high-risk studies is not explicitly reported, though overall risk of bias is discussed as a limitation.	

Phase 3: Judging risk of bias

Summarize the concerns identified during the Phase 2 assessment:

Domain	Concern	Evidence / Rationale
1. Eligibility Criteria	LOW	Criteria are clear and appropriate.
2. Identification/Selection	HIGH	Good screening process, but search strategy reporting is incomplete and gray literature/registries were not systematically searched.
3. Data Collection/Appraisal	LOW	Independent duplicate processes used for data extraction and risk of bias assessment.
4. Synthesis and Findings	HIGH	No reference to a pre-registered protocol, making it impossible to assess selective reporting.

RISK OF BIAS IN THE REVIEW

Signaling Question	Response	Evidence / Rationale
A. Did the interpretation of findings address all of the concerns identified in Domains 1 to 4?	PN	It does not address the major concern regarding the lack of a pre-registered protocol and its implications for selective reporting bias. It also does not discuss the potential impact of excluding gray literature or not searching trial registries.
B. Was the relevance of identified studies to the review's research question appropriately considered?	Y	The included studies all appear relevant to the PICO question. Subgroup analysis by training modality directly addresses the review's aim.
C. Did the reviewers avoid emphasizing results on the basis of their statistical significance?	PY	Results are presented with effect sizes and confidence intervals. Conclusions appear balanced, noting non-significant findings for IL-6 reduction and no effect on TNF-α. However, the abstract highlights “AT + RT being the best training modality” based on significant CRP findings, which is consistent with the results but could be seen as emphasizing statistical significance.

Y=YES, PY=PROBABLY YES, PN=PROBABLY NO, N=NO, NI=NO INFORMATION

2.17. Detailed Risk of Bias Assessment for the Systematic Review by Keyvan Hejazi et al. (2022) I

Phase 1: Assessing relevance

Category	Target question	Review being assessed
Patients / Population(s)	Overweight/obese adults (BMI ≥25 kg/m²), ≥18 years old, sedentary (＜150 min/week physical activity) prior to enrollment, no severe comorbidities contraindicating exercise.	Overweight/obese adults (BMI 25–47.2 kg/m²); 2,752 participants from 35 randomized controlled trials (RCTs); mean age 18–70.5 years; 18 male-only, 13 female-only, 4 mixed-sex studies; 17 obese (BMI≥30 kg/m²) and 18 overweight (BMI 25–29.9 kg/m²) cohorts; all sedentary at baseline.
Intervention(s)	Exercise training (aerobic [AT], resistance [RT], combined [AT+RT], interval [IT]/HIIT) with measurable parameters (≥8 weeks, 2–5 sessions/week, 15–90 min/session).	AT (15 studies): 3–5 sessions/week, 8–52 weeks; RT (5 studies): 3 sessions/week, 8–24 weeks; AT+RT (4 studies): 3 sessions/week, 8–24 weeks; IT/HIIT (3 studies): 2–3 sessions/week, 8–12 weeks; all meet duration/frequency requirements.
Comparator(s)	Usual care, sham exercise, or no structured physical activity.	Controls received no exercise intervention or maintained habitual lifestyle; no concurrent dietary/supplement interventions (excluded per inclusion criteria).
Outcome(s)	Usual care, sham exercise, or no structured physical activity.	Controls received no exercise intervention or maintained habitual lifestyle; no concurrent dietary/supplement interventions (excluded per inclusion criteria).

Phase 2: Identifying concerns with the review process

Domain	Signaling Questions	Response	Evidence / Rationale	Risk Bias Assessment
DOMAIN 1: STUDY ELIGIBILITY CRITERIA	1.1 Did the review adhere to pre-defined objectives and eligibility criteria?	PY	The protocol was not registered and no prior published protocol was mentioned. Methods state it followed PRISMA,	LOW
	1.2 Were the eligibility criteria appropriate for the review question?	Y	PICOS criteria clearly described: overweight/obese adults, exercise interventions >8 weeks, control groups, inflammatory/cardiometabolic outcomes, RCTs.	
	1.3 Were eligibility criteria unambiguous?	PY	Most criteria clear, though “regular physical activity” prior to study not strictly defined (stated as <150 min/week).	
	1.4 Were all restrictions in eligibility criteria based on study characteristics appropriate?	PY	Only RCTs included.language restricted to English. No date restrictions.	
	1.5 Were any restrictions in eligibility criteria based on sources of information appropriate?	PY	Excluded reviews, conference abstracts, non-English studies	
DOMAIN 2: IDENTIFICATION AND SELECTION OF STUDIES	2.1 Did the search include an appropriate range of databases/electronic sources?	Y	PubMed, CINAHL, MEDLINE, Scopus, Web of Science, Google Scholar, Cochrane.	LOW
	2.2 Were methods additional to database searching used to identify relevant reports?	PY	Reference lists of included studies checked; no mention of contacting experts or grey literature searches.	
	2.3 Were the terms and structure of the search strategy likely to retrieve as many eligible studies as possible?	Y	Search strategy provided in supplementary material; includes MeSH and free-text terms.	
	2.4 Were restrictions based on date, publication format, or language appropriate?	PY	No date or language restrictions mentioned, excluded non-English studies	
	2.5 Were efforts made to minimise error in selection of studies?	Y	Two reviewers independently screened titles/abstracts and full texts, disagreements resolved by consensus.	
DOMAIN 3: DATA COLLECTION AND STUDY APPRAISAL	3.1 Were efforts made to minimise error in data collection?	Y	Two reviewers independently extracted data using a pre-defined form; consensus used for disagreements.	LOW
	3.2 Were sufficient study characteristics available for interpretation?	Y	Table 1 provides detailed study characteristics	
	3.3 Were all relevant study results collected for use in the synthesis?	Y	Pre/post means and SDs extracted for all outcomes	
	3.4 Was risk of bias (or methodological quality) formally assessed using appropriate criteria?	PY	Used TESTEX tool, but not a standard bias assessment tool like Cochrane RoB.	
	3.5 Were efforts made to minimise error in risk of bias assessment?	Y	Two reviewers independently assessed quality using TESTEX, disagreements resolved by consensus.	

DOMAIN 4: SYNTHESIS AND FINDINGS	4.1 Did the synthesis include all studies that it should?	Y	All 35 included studies appear in forest plots/analyses for relevant outcomes.	HIGH
	4.2 Were all pre-defined analyses reported or departures explained?	PN	No protocol available; subgroup analyses conducted but unclear if all were pre-specified.	
	4.3 Was the synthesis appropriate given the nature of studies?	Y	Random-effects model used; high heterogeneity acknowledged and explored via subgroup/sensitivity analyses.	
	4.4 Was between-study variation (heterogeneity) minimal or addressed in the synthesis?	PN	High statistical heterogeneity for many outcomes, subgroup analyses performed but residual heterogeneity remained high.	
	4.5 Were the findings robust (e.g., funnel plot/sensitivity analyses)?	Y	Sensitivity analysis using leave-one-out method performed; funnel plots and Egger's test used for publication bias.	
	4.6 Were biases in primary studies minimal or addressed in the synthesis?	PY	TESTEX scores indicated moderate quality; sensitivity analysis excluding high risk studies not explicitly performed.	

Phase 3: Judging risk of bias

Summarize the concerns identified during the Phase 2 assessment:

Domain	Concern	Evidence / Rationale
1. Eligibility Criteria	LOW	The review states it followed PRISMA and had a priori criteria. However, no protocol registration (e.g., PROSPERO) is mentioned.
2. Identification/Selection	LOW	The process was systematic, comprehensive, and reproducible.
3. Data Collection/Appraisal	HIGH	There is high heterogeneity (I^2 > 90%) for several key outcome measures, and the review failed to adequately explain or address the sources of this heterogeneity.
4. Synthesis and Findings	LOW	The process of data extraction and quality appraisal is rigorous, but the assessment tool used (TESTEX) is not specifically designed to evaluate the risk of bias in randomized controlled trials.

RISK OF BIAS IN THE REVIEW

Signaling Question	Response	Evidence / Rationale
A. Did the interpretation of findings address all of the concerns identified in Domains 1 to 4?	N	High heterogeneity and lack of protocol registration were not fully mitigated in interpretation.
B. Was the relevance of identified studies to the review's research question appropriately considered?	Y	Included studies matched PICOS; discussed generalisability in limitations.
C. Did the reviewers avoid emphasizing results on the basis of their statistical significance?	Y	Conclusions aligned with results; acknowledged limitations including heterogeneity and publication bias.

Y=YES, PY=PROBABLY YES, PN=PROBABLY NO, N=NO, NI=NO INFORMATION

2.18. Detailed Risk of Bias Assessment for the Systematic Review by Keyvan Hejazi et al. (2022) II

Phase 1: Assessing relevance

Category	Target question	Review being assessed
Patients / Population(s)	Adults (≥18 years) with type 2 diabetes mellitus (T2DM), sedentary (<150 min/week physical activity) prior to enrollment, no severe comorbidities contraindicating exercise.	Adults with T2DM; 1257 participants from 25 randomized controlled trials (RCTs); mean age 40–67 years; 9 male-only, 2 female-only, 14 mixed-sex studies; 2 normal-weight, 9 overweight (BMI 25–29.9 kg/m²), 13 obese (BMI≥30 kg/m²) cohorts; all sedentary at baseline.
Intervention(s)	Exercise training (aerobic [AT], resistance [RT], combined [AT+RT], interval [IT]/HIIT) with measurable parameters (≥8 weeks, 2–5 sessions/week, 15–150 min/session).	AT (14 studies): 3–5 sessions/week, 8–52 weeks; RT (2 studies): 3 sessions/week, 16 weeks; AT+RT (2 studies): 3 sessions/week, 16–12 weeks; IT/HIIT (3 studies): 3–4 sessions/week, 8–12 weeks; all meet duration/frequency requirements.
Comparator(s)	Usual care or no structured physical activity.	Controls received no exercise intervention; no concurrent dietary/supplement interventions (excluded per inclusion criteria).
Outcome(s)	Inflammatory markers (adiponectin, TNF-α, IL-6, CRP, leptin) and cardiometabolic markers (fasting insulin [FI], fasting blood glucose [FBG], HOMA-IR, lipid profile).	CAll pre-defined outcomes reported; ET significantly increases adiponectin (WMD=1.94 µg/mL, p=0.0001) and decreases TNF-α (WMD=-2.44 pg/mL, p=0.001), IL-6 (WMD=-4.80 pg/mL, p<0.0001), CRP (WMD=-0.82 mg/L, p=0.0006), FI (WMD=-7.36 µIU/mL, p=0.0004), HOMA-IR (WMD=-0.41, p=0.01); no significant effects on lipid profile.

Phase 2: Identifying concerns with the review process

Domain	Signaling Questions	Response	Evidence / Rationale	Risk Bias Assessment
DOMAIN 1: STUDY ELIGIBILITY CRITERIA	1.1 Did the review adhere to pre-defined objectives and eligibility criteria?	PY	The review states the PICOS criteria and mentions a protocol registered on PROSPERO (CRD42022307396). Adherence is probable but not explicitly verified against the protocol within the published report.	LOW
	1.2 Were the eligibility criteria appropriate for the review question?	Y	The PICOS criteria are directly aligned with the stated research objectives.	
	1.3 Were eligibility criteria unambiguous?	Y	Criteria for population, intervention, comparator, outcomes, and study design are clearly stated in the "Selection Criteria" section.	
	1.4 Were all restrictions in eligibility criteria based on study characteristics appropriate?	PY	Justification for language restriction is not provided.	
	1.5 Were any restrictions in eligibility criteria based on sources of information appropriate?	PY	The search included gray literature (Google Scholar, trial registries) and was not restricted by publication status. The English language restriction, while common, may have excluded relevant studies.	
DOMAIN 2: IDENTIFICATION AND SELECTION OF STUDIES	2.1 Did the search include an appropriate range of databases/electronic sources?	Y	Comprehensive search across 6 major databases (PubMed/Medline, Embase, Cochrane, CINAHL, Scopus, Web of Science) plus Google Scholar.	LOW
	2.2 Were methods additional to database searching used to identify relevant reports?	Y	Reference lists of included studies and related reviews were hand-searched.	
	2.3 Were the terms and structure of the search strategy likely to retrieve as many eligible studies as possible?	Y	The strategy used a mix of MeSH and free-text terms for key concepts (population, intervention, outcomes). The full strategy for one database is provided in supplementary material.	
	2.4 Were restrictions based on date, publication format, or language appropriate?	PY	Search was from inception to Jan 2022. Language was restricted to English.	
	2.5 Were efforts made to minimise error in selection of studies?	Y	Two reviewers independently screened titles/abstracts and full texts. Disagreements were resolved via discussion.	
DOMAIN 3: DATA COLLECTION	3.1 Were efforts made to minimise error in data collection?	Y	Two authors independently extracted data using a pre-defined form. Disagreements were resolved through discussion.	LOW
	3.2 Were sufficient study characteristics available for interpretation?	Y	Extensive study characteristics are provided in Table 1	
	3.3 Were all relevant study results collected	Y	The review lists all pre-specified biomarkers and reports extracting all relevant data. Methods for handling	

AND STUDY APPRAISAL	for use in the synthesis?		unreported data are described.	
	3.4 Was risk of bias (or methodological quality) formally assessed using appropriate criteria?	PY	Used TESTEX tool, but not a standard bias assessment tool like Cochrane RoB.	
	3.5 Were efforts made to minimise error in risk of bias assessment?	Y	Two reviewers independently assessed risk of bias. Disagreements were resolved through discussion.	
DOMAIN 4: SYNTHESIS AND FINDINGS	4.1 Did the synthesis include all studies that it should?	Y	The meta-analysis includes all eligible studies for each outcome.	HIGH
	4.2 Were all pre-defined analyses reported or departures explained?	Y	Pre-specified subgroup analyses are reported. The protocol was registered, and the methods section details all planned analyses.	
	4.3 Was the synthesis appropriate given the nature of studies?	PN	Random-effects models were used appropriately given expected heterogeneity. Subgroup analyses were conducted to explore heterogeneity.	
	4.4 Was between-study variation (heterogeneity) minimal or addressed in the synthesis?	PY	Heterogeneity was very high for most primary outcomes. While acknowledged and explored via subgroup analysis, it remains substantial and largely unexplained, casting doubt on the summary estimates.	
	4.5 Were the findings robust (e.g., funnel plot/sensitivity analyses)?	Y	Funnel plots and Egger's test were used to assess publication bias for all outcomes. Sensitivity analyses were performed.	
	4.6 Were biases in primary studies minimal or addressed in the synthesis?	PN	The TESTEX assessment indicated common methodological shortcomings in primary studies (e.g., poor reporting of randomization, allocation concealment, blinding). The synthesis did not formally weight studies by RoB or perform a meta-regression on RoB items, though a sensitivity analysis excluding high RoB studies was mentioned for one outcome.	

Phase 3: Judging risk of bias

Summarize the concerns identified during the Phase 2 assessment:

Domain	Concern	Evidence / Rationale
1. Eligibility Criteria	LOW	Criteria were clear, pre-defined, and appropriate.
2. Identification/Selection	LOW	Comprehensive search with independent screening. Language restriction is a minor concern.
3. Data Collection/Appraisal	LOW	Rigorous dual-review process with appropriate tools.
4. Synthesis and Findings	HIGH	High unexplained heterogeneity and insufficient handling of prevalent risk of bias in primary studies undermine confidence in the synthesized results.

RISK OF BIAS IN THE REVIEW

Signaling Question	Response	Evidence / Rationale
A. Did the interpretation of findings address all of the concerns identified in Domains 1 to 4?	PN	The discussion acknowledges high heterogeneity and limitations of primary studies but does not adequately frame the results with the necessary caution. The conclusions present the findings as more definitive than the high risk of bias in the synthesis warrants.
B. Was the relevance of identified studies to the review's research question appropriately considered?	PY	The PICOS framework was used, and study characteristics are well-documented, showing clear relevance.
C. Did the reviewers avoid emphasizing results on the basis of their statistical significance?	PY	While p-values are reported, the discussion also considers effect sizes (SMD). However, conclusions often highlight "significant" changes without equal emphasis on the high heterogeneity that qualifies them.

Y=YES, PY=PROBABLY YES, PN=PROBABLY NO, N=NO, NI=NO INFORMATION

2.19. Detailed Risk of Bias Assessment for the Systematic Review by Mousa Khalafi et al. (2022)

Phase 1: Assessing relevance

Category	Target question	Review being assessed
Patients / Population(s)	Overweight/obese individuals (BMI ≥25 kg/m²), no severe comorbidities contraindicating lifestyle interventions, aged ≥13 years.	Overweight/obese individuals; 2108 participants from 38 trials (32 articles); mean age 13.1–70 years; mixed-sex, male-only, and female-only cohorts; BMI 26.9–44 kg/m²; includes individuals with comorbidities (T2D, asthma, CVD) and healthy populations.
Intervention(s)	Exercise training (EX: aerobic/RT/combined/HIIT), caloric restriction (CR), EX+CR; intervention duration ≥4 weeks.	EX (aerobic/RT/combined/HIIT): 2–7 sessions/week, 6 weeks–18 months; CR (250–1000 kcal/day deficit/low-calorie diets): ≥4 weeks; EX+CR (combination of above); all meet duration requirements.
Comparator(s)	Caloric restriction (CR) alone.	Controls received CR alone; no concurrent confounding interventions (e.g., supplements).
Outcome(s)	Inflammatory markers: C-reactive protein (CRP), interleukin-6 (IL-6), tumor necrosis factor-α (TNF-α).	All pre-defined outcomes reported; EX+CR vs. CR: significantly reduces IL-6 (SMD=-0.15, p=0.02) and TNF-α (SMD=-0.22, p=0.002), tends to reduce CRP (SMD=-0.10, p=0.06); EX vs. CR: CR superior for CRP (SMD=0.21, p=0.01), no differences for IL-6/TNF-α.

Phase 2: Identifying concerns with the review process

Domain	Signaling Questions	Response	Evidence / Rationale	Risk Bias Assessment
DOMAIN 1: STUDY ELIGIBILITY CRITERIA	1.1 Did the review adhere to pre-defined objectives and eligibility criteria?	PY	The authors state the review followed PRISMA and Cochrane guidelines. However, the study protocol was not registered in PROSPERO or any other database,	LOW
	1.2 Were the eligibility criteria appropriate for the review question?	Y	Criteria are clearly focused on the PICO	
	1.3 Were eligibility criteria unambiguous?	PY	Criteria are generally clear (e.g., intervention ≥4 weeks, human subjects, specific markers).	
	1.4 Were all restrictions in eligibility criteria based on study characteristics appropriate?	PY	Restrictions on study design (RCTs implied but not explicitly stated), duration (≥4 weeks), and population (overweight/obese) are reasonable. However, the exclusion of non-English studies may introduce language bias, a potential threat to comprehensiveness.	
	1.5 Were any restrictions in eligibility criteria based on sources of information appropriate?	PY	The restriction to English-language publications only is a source of potential bias, as relevant studies in other languages may have been missed.	
DOMAIN 2: IDENTIFICATION AND SELECTION OF STUDIES	2.1 Did the search include an appropriate range of databases/electronic sources?	Y	The authors searched PubMed, Scopus, Web of Science, and Cochrane Library	HIGH
	2.2 Were methods additional to database searching used to identify relevant reports?	Y	The authors hand-searched reference lists of retrieved articles and relevant reviews to identify additional studies.	
	2.3 Were the terms and structure of the search strategy likely to retrieve as many eligible studies as possible?	PN	The search strategy is described with key terms (exercise, caloric restriction, inflammation markers) but the full electronic search strategy for at least one database is not provided in the paper or supplementary materials, making reproducibility and assessment of comprehensiveness difficult.	
	2.4 Were restrictions based on date, publication format, or language appropriate?	PY	The language restriction may lead to the exclusion of relevant data. No date restriction is mentioned,	
	2.5 Were efforts made to minimise error in selection of studies?	Y	Yes. The process was conducted independently by two reviewers, with disagreements resolved by discussion or a third reviewer.	
DOMAIN 3: DATA COLLECTION AND STUDY APPRAISAL	3.1 Were efforts made to minimise error in data collection?	Y	Data extraction was performed independently by two reviewers using a piloted form, with disagreements resolved by a third reviewer (M.E.S.).	HIGH
	3.2 Were sufficient study characteristics available for interpretation?	Y	provide extensive details on participant characteristics (age, sex, BMI, health status) and intervention characteristics (type, duration, intensity, dietary details). Supplementary tables provide raw data.	
	3.3 Were all relevant study results collected for use in the synthesis?	Y	Pre- and post-intervention means/SD or mean differences/SD were extracted for all inflammation markers and body weight. The authors describe calculating data from figures or other statistics when necessary.	
	3.4 Was risk of bias (or methodological quality) formally assessed using appropriate criteria?	PN	Quality was assessed using "an eight-item checklist adapted from the PRISMA statement". This is a non-standard tool.	
	3.5 Were efforts made to minimise error in	Y	Data extraction was performed independently by two reviewers using a piloted form, with disagreements	

	risk of bias assessment?		resolved by a third reviewer (M.E.S.).	
DOMAIN 4: SYNTHESIS AND FINDINGS	4.1 Did the synthesis include all studies that it should?	PY	The forest plots (Figures 2-7) and supplementary figures appear to include all studies that met the inclusion criteria and reported data for each outcome. The flow diagram (Figure 1) accounts for excluded studies.	HIGH
	4.2 Were all pre-defined analyses reported or departures explained?	Y	The authors state the protocol was not registered. Therefore, it is impossible to verify if all planned analyses were reported. However, the methods section describes the intended analyses (meta-analysis, subgroup, sensitivity), and these are all presented in the results.	
	4.3 Was the synthesis appropriate given the nature of studies?	Y	A random-effects model was appropriately used for most analyses due to expected clinical/methodological heterogeneity. SMD was used for meta-analysis of different scales. Subgroup analyses (duration, BMI, exercise type) and sensitivity analyses were conducted to explore heterogeneity.	
	4.4 Was between-study variation (heterogeneity) minimal or addressed in the synthesis?	Y	Heterogeneity was quantified using I^2 statistics and reported for each analysis.	
	4.5 Were the findings robust (e.g., funnel plot/sensitivity analyses)?	Y	Sensitivity analyses were performed by removing each study individually. Publication bias was assessed via funnel plots and Egger's test where applicable.	
	4.6 Were biases in primary studies minimal or addressed in the synthesis?	N	The quality assessment (Supplementary Table 3) shows varying and often moderate risk of bias in primary studies (scores ranging from 1 to 8 out of 8). The authors discuss this as a limitation but did not perform a formal meta-analysis stratified by risk of bias or exclude high-risk studies.	

Phase 3: Judging risk of bias

Summarize the concerns identified during the Phase 2 assessment:

Domain	Concern	Evidence / Rationale
1. Eligibility Criteria	LOW	Lack of protocol registration; language bias
2. Identification/Selection	HIGH	Unreproducible search strategy
3. Data Collection/Appraisal	HIGH	Generally robust methods
4. Synthesis and Findings	HIGH	The pooled results may be influenced by biased primary studies, and the integrity of the analysis plan cannot be verified

RISK OF BIAS IN THE REVIEW

Signaling Question	Response	Evidence / Rationale
A. Did the interpretation of findings address all of the concerns identified in Domains 1 to 4?	PN	the lack of protocol registration, inclusion of participants with comorbidities, variability in CR interventions, and the generally moderate quality of primary studies. However, it does not explicitly discuss the critical limitation of the English-only search restriction or the implications of an unreproducible search strategy. While some concerns are noted, the most critical methodological threats to the review's validity are not fully addressed in the interpretation.
B. Was the relevance of identified studies to the review's research question appropriately considered?	Y	The authors discuss the clinical relevance of findings in the context of overweight/obese populations and various health conditions. Subgroup analyses further demonstrate consideration of relevance.
C. Did the reviewers avoid emphasizing results on the basis of their statistical significance?	PY	The results are presented with effect sizes and confidence intervals. The discussion interprets the magnitude and direction of effects rather than relying solely on p-values. However, p-values are still prominently reported in the abstract and results, which could lead to over-interpretation of borderline results. Overall, the emphasis is reasonably balanced.

Y=YES, PY=PROBABLY YES, PN=PROBABLY NO, N=NO, NI=NO INFORMATION

2.20. Detailed Risk of Bias Assessment for the Systematic Review by Yijian Ding et al. (2022)

Phase 1: Assessing relevance

Category	Target question	Review being assessed
Patients / Population(s)	Overweight/obese individuals (BMI ≥25 kg/m²), generally healthy (no inflammatory/cardiovascular/neurological/psychological diseases), aged ≥13.9 years.	Overweight/obese individuals; 779 participants from 16 randomized controlled trials (RCTs); mean age 13.9–71.6 years; 9 overweight (BMI 25–29.9 kg/m²), 6 obese (BMI ≥30 kg/m²), 1 mixed BMI cohort; 8 male-only, 4 female-only, 4 mixed-sex studies; all free of confounding diseases.
Intervention(s)	Exercise training (aerobic [AE], resistance [RE], combined [AE+RE], high-intensity interval training [HIIT]); ≥4 weeks, ≥2 sessions/week.	AE/RE/AE+RE/HIIT: 2–7 sessions/week, 4 weeks–6 months; all meet duration/frequency requirements; no concurrent confounding interventions (except 3 with diet/3 with pharmacology, accounted for in subgroup analyses).
Comparator(s)	No structured exercise (resting control group), with consistent other treatments.	Controls received no exercise intervention; other treatments (diet/pharmacology) balanced between groups; no additional confounding interventions.
Outcome(s)	Inflammasome activation-related inflammatory cytokines: interleukin-1β (IL-1β), interleukin-18 (IL-18).	All pre-defined outcomes reported; exercise training significantly reduces IL-1β (SMD=-0.527, p<0.001) and IL-18 (SMD=-0.799, p=0.011); subgroup analyses identify modifiers (BMI, gender, intensity, duration).

Phase 2: Identifying concerns with the review process

Domain	Signaling Questions	Response	Evidence / Rationale	Risk Bias Assessment
DOMAIN 1: STUDY ELIGIBILITY CRITERIA	1.1 Did the review adhere to pre-defined objectives and eligibility criteria?	PY	The review states it was registered in PROSPERO (CRD42020205648) and follows PRISMA 2020. Objectives and eligibility criteria are clearly defined in the Methods section (Section 2.2).	LOW
	1.2 Were the eligibility criteria appropriate for the review question?	Y	Criteria clearly specify: participants (BMI ≥25 kg/m², generally healthy), intervention (exercise training ≥2 times/week for ≥4 weeks), comparator (resting control), outcomes (IL-1β and IL-18 in plasma/serum), and study design (RCT). These align with the research question.	
	1.3 Were eligibility criteria unambiguous?	Y	Clear and detailed descriptions of inclusion/exclusion criteria are provided in Section 2.2.	
	1.4 Were all restrictions in eligibility criteria based on study characteristics appropriate?	Y	Restrictions are justified: only RCTs, only overweight/obese otherwise healthy populations, only IL-1β/IL-18 protein levels. No inappropriate restrictions (e.g., sample size, study duration) were applied.	
	1.5 Were any restrictions in eligibility criteria based on sources of information appropriate?	PY	Language was restricted to English and Chinese, which may exclude relevant studies in other languages. However, the search included both international and Chinese databases, which may partially mitigate this limitation.	
DOMAIN 2: IDENTIFICATION AND SELECTION OF STUDIES	2.1 Did the search include an appropriate range of databases/electronic sources?	Y	Six databases were searched: PubMed, Web of Science, Embase, Cochrane Library, Wanfang, CNKI. These cover major English and Chinese biomedical databases.	LOW
	2.2 Were methods additional to database searching used to identify relevant reports?	PY	Reference lists of included studies and relevant reviews were examined. No mention of contacting experts or searching trial registries beyond those included in database searches.	
	2.3 Were the terms and structure of the search strategy likely to retrieve as many eligible studies as possible?	Y	Search terms covered exercise, cytokine names, and RCT-related terms. Full strategies for each database are provided in Supplementary Table S1.	
	2.4 Were restrictions based on date, publication format, or language appropriate?	PY	No date restrictions were applied, which is appropriate. Language was restricted to English and Chinese, which may introduce language bias.	
	2.5 Were efforts made to minimise error in selection of studies?	Y	Two reviewers independently screened titles/abstracts and full texts, with disagreements resolved through discussion. This is clearly stated in.	
DOMAIN 3: DATA COLLECTION AND STUDY APPRAISAL	3.1 Were efforts made to minimise error in data collection?	Y	Two reviewers independently extracted data using a pre-designed form, with disagreements resolved by discussion or third reviewer.	LOW
	3.2 Were sufficient study characteristics available for interpretation?	Y	Detailed study characteristics are provided in Supplementary Tables S2 and S3	
	3.3 Were all relevant study results collected for use in the synthesis?	Y	Data for IL-1β and IL-18 were extracted from all included studies.	
	3.4 Was risk of bias (or methodological quality) formally assessed using appropriate criteria?	Y	Cochrane Risk of Bias tool was used for each RCT.	
	3.5 Were efforts made to minimise error in risk of bias assessment?	PY	The text states that two reviewers assessed RoB, but it is not explicitly stated whether they did so independently.	

DOMAIN 4: SYNTHESIS AND FINDINGS	4.1 Did the synthesis include all studies that it should?	Y	All included studies have been incorporated into the meta-analyses	LOW
	4.2 Were all pre-defined analyses reported or departures explained?	PY	Subgroup analyses were pre-specified (BMI, gender, exercise type, intensity, duration, diet). All planned analyses appear to have been reported. No clear protocol is available for comparison, but the methods section outlines the planned analyses.	
	4.3 Was the synthesis appropriate given the nature of studies?	Y	Random-effects models were used due to expected heterogeneity. Subgroup and sensitivity analyses were performed. Methods align with Cochrane recommendations.	
	4.4 Was between-study variation (heterogeneity) minimal or addressed in the synthesis?	Y	I^2 was reported and interpreted. Heterogeneity was explored via subgroup and sensitivity analyses.	
	4.5 Were the findings robust (e.g., funnel plot/sensitivity analyses)?	Y	Sensitivity analyses were conducted by omitting each study (Table 3). Funnel plots and Egger's/Begg's tests were used to assess publication bias (Fig. 5).	
	4.6 Were biases in primary studies minimal or addressed in the synthesis?	PY	The Cochrane RoB tool was applied, and overall study quality was discussed.	

Phase 3: Judging risk of bias

Summarize the concerns identified during the Phase 2 assessment:

Domain	Concern	Evidence / Rationale
1. Eligibility Criteria	LOW	Criteria were clear, pre-specified, and appropriate.
2. Identification/Selection	LOW	Comprehensive search and independent screening. Language restriction is a minor limitation.
3. Data Collection/Appraisal	LOW	Systematic duplicate processes for data extraction and RoB assessment.
4. Synthesis and Findings	LOW	Appropriate meta-analytic methods, heterogeneity handling, and sensitivity analyses. No major flaws identified.

RISK OF BIAS IN THE REVIEW

Signaling Question	Response	Evidence / Rationale
A. Did the interpretation of findings address all of the concerns identified in Domains 1 to 4?	Y	All signaling questions answered Y/PY, criteria are clear, appropriate, and well-specified.
B. Was the relevance of identified studies to the review's research question appropriately considered?	Y	Included studies all matched the PICO criteria. Subgroup analyses were used to explore relevance.
C. Did the reviewers avoid emphasizing results on the basis of their statistical significance?	Y	The discussion interprets findings in the context of clinical relevance, magnitude of effect (SMD), and consistency across subgroups, not solely statistical significance.

Y=YES, PY=PROBABLY YES, PN=PROBABLY NO, N=NO, NI=NO INFORMATION

2.21. Detailed Risk of Bias Assessment for the Systematic Review by Yubo Liu et al. (2021)

Phase 1: Assessing relevance

Category	Target question	Review being assessed
Patients / Population(s)	Overweight/obese adults (BMI ≥25 kg/m²), aged ≥18 years, no confounding inflammatory/chronic diseases.	Overweight/obese adults; 1196 participants from 23 trials (19 articles); mean age 18–75 years; BMI 27.7–37.2 kg/m²; mixed-sex, male-only, female-only cohorts; excluded children/adolescents and individuals with confounding diseases.
Intervention(s)	Combined exercise (EX: aerobic/resistance/HIIT) + calorie restriction (CR); intervention duration ≥8 weeks.	EX+CR: aerobic/resistance/combined/HIIT, 3–7 sessions/week, 8–72 weeks; CR: calorie deficit/low-calorie diets; all meet duration/frequency requirements; no concurrent confounding interventions.
Comparator(s)	Calorie restriction (CR) alone.	Controls received CR alone; other treatments balanced between groups; no additional confounding interventions.
Outcome(s)	Inflammatory biomarkers: C-reactive protein (CRP), interleukin-6 (IL-6), tumor necrosis factor-α (TNF-α).	All pre-defined outcomes reported; EX+CR vs. CR: significantly reduces CRP (SMD=-0.16, p=0.02)

Phase 2: Identifying concerns with the review process

Domain	Signaling Questions	Response	Evidence / Rationale	Risk Bias Assessment
DOMAIN 1: STUDY ELIGIBILITY CRITERIA	1.1 Did the review adhere to pre-defined objectives and eligibility criteria?	PY	A protocol was mentioned but not registered on PROSPERO (no ID given). Objectives and criteria are clearly stated in Methods.	LOW
	1.2 Were the eligibility criteria appropriate for the review question?	Y	Criteria are directly aligned with the PICO question	
	1.3 Were eligibility criteria unambiguous?	Y	Criteria are specified clearly, including participant characteristics (age ≥18, BMI criteria implied), intervention details, comparator, outcomes, study design (RCTs and non-RCTs with MINORS score >12), language (English), and publication type (full-text). Exclusions are also explicitly listed.	
	1.4 Were all restrictions in eligibility criteria based on study characteristics appropriate?	Y	Restrictions are justified: only human adults, studies measuring at least one of the three specified inflammatory biomarkers, minimum intervention duration (implied by exclusion of studies <4 weeks), and inclusion of both RCTs and high-quality non-RCTs. These are appropriate for the review question.	
	1.5 Were any restrictions in eligibility criteria based on sources of information appropriate?	PY	The search included published and unpublished sources (conference abstracts, trial registries). No language restrictions were applied during the search, but only English full-text articles were included in the final synthesis. This is a potential limitation but common and not inherently inappropriate. The restriction to English is noted.	
DOMAIN 2: IDENTIFICATION AND SELECTION OF STUDIES	2.1 Did the search include an appropriate range of databases/electronic sources?	Y	Comprehensive search across 6 major databases: PubMed, Web of Science, EMBASE, Cochrane, Scopus, Google Scholar. Supplemental searches included manual checking of reference lists and searching trial registries (ICTRP, ClinicalTrials.gov).	LOW
	2.2 Were methods additional to database searching used to identify relevant reports?	Y	Manual search of reference lists and search of conference abstracts were performed.	
	2.3 Were the terms and structure of the search strategy likely to retrieve as many eligible studies as possible?	PY	The search strategy is reported with specific keywords covering exercise, calorie restriction, and inflammation (Page 3). The strategy appears comprehensive for the topic.	
	2.4 Were restrictions based on date, publication format, or language appropriate?	PY	While no date restrictions were applied (searched up to April 2021), the final inclusion was restricted to English-language full-text articles. This introduces a potential language bias. The restriction is reported but may have excluded relevant studies.	
	2.5 Were efforts made to minimise error in selection of studies?	Y	Study selection was performed independently by two authors (YL, FH) with disagreements resolved by discussion or a third reviewer (MK), as stated in the "Inclusion and Exclusion Criteria" section.	
DOMAIN 3: DATA COLLECTION AND STUDY APPRAISAL	3.1 Were efforts made to minimise error in data collection?	Y	Data extraction was performed independently by two reviewers (YL, FH) and verified by a third (MK).	LOW
	3.2 Were sufficient study characteristics available for interpretation?	Y	Extensive study characteristics are provided in Table 1, including participant details, intervention specifics , and outcomes.	
	3.3 Were all relevant study results collected for use in the synthesis?	Y	Data for all pre-specified outcomes (CRP, IL-6, TNF-伪) were extracted from all included studies, as evidenced by the forest plots and results section.	
	3.4 Was risk of bias (or methodological quality) formally assessed using appropriate criteria?	Y	RCTs were assessed using the Cochrane Risk of Bias tool, and non-RCTs were assessed using the MINORS tool.	

	3.5 Were efforts made to minimise error in risk of bias assessment?	Y	The quality assessment was performed by two authors independently (YL, FH) with discrepancies resolved by a third reviewer (MK).	
DOMAIN 4: SYNTHESIS AND FINDINGS	4.1 Did the synthesis include all studies that it should?	Y	The meta-analysis includes all 23 trials that met the inclusion criteria.	LOW
	4.2 Were all pre-defined analyses reported or departures explained?	PY	The methods state the intention to perform meta-analysis, meta-regression, and subgroup analysis based on lifestyle. All are reported in the results. The protocol was not registered on PROSPERO, so it's unclear if all pre-planned analyses (e.g., specific sensitivity analyses) were reported. However, the reported analyses align with the stated methods.	
	4.3 Was the synthesis appropriate given the nature of studies?	Y	A random-effects model was used, which is appropriate given the expected clinical and methodological heterogeneity. Subgroup analysis and meta-regression were used to explore sources of heterogeneity	
	4.4 Was between-study variation (heterogeneity) minimal or addressed in the synthesis?	Y	Heterogeneity was assessed using I^2 statistics. Significant heterogeneity was found for some outcomes (e.g., I^2=83.1% for IL-6 overall), and the authors appropriately investigated sources via subgroup analysis (lifestyle) and meta-regression.	
	4.5 Were the findings robust (e.g., funnel plot/sensitivity analyses)?	PY	While funnel plots and Egger's test were mentioned for publication bias, no results are presented in the main text or supplementary figures for the primary meta-analysis.	
	4.6 Were biases in primary studies minimal or addressed in the synthesis?	Y	The risk of bias in primary studies was assessed, and the authors state that excluding high-risk studies in a sensitivity analysis did not change the results	

Phase 3: Judging risk of bias

Summarize the concerns identified during the Phase 2 assessment:

Domain	Concern	Evidence / Rationale
1. Eligibility Criteria	LOW	Criteria were clear, pre-defined, and appropriate.
2. Identification/Selection	LOW	The restriction to English-language articles for inclusion introduces a potential for language bias,.
3. Data Collection/Appraisal	LOW	Processes were rigorous and duplicated to minimize error.
4. Synthesis and Findings	LOW	The synthesis methods were otherwise appropriate.

RISK OF BIAS IN THE REVIEW

Signaling Question	Response	Evidence / Rationale
A. Did the interpretation of findings address all of the concerns identified in Domains 1 to 4?	Y	All signaling questions answered Y/PY; criteria are clear, appropriate, and well-specified.
B. Was the relevance of identified studies to the review's research question appropriately considered?	Y	The discussion interprets findings in the context of the research question and compares them with previous meta-analyses. Subgroup analysis based on lifestyle directly addresses the relevance of participant characteristics to the intervention effect.
C. Did the reviewers avoid emphasizing results on the basis of their statistical significance?	Y	The results report both statistically significant (CRP in normal lifestyle subgroup) and non-significant findings (IL-6 and TNF-α in the overall analysis) with balanced interpretation. The conclusion emphasizes the modifying effect of lifestyle, not just statistical significance.

Y=YES, PY=PROBABLY YES, PN=PROBABLY NO, N=NO, NI=NO INFORMATION

2.22. Detailed Risk of Bias Assessment for the Systematic Review by Junga Lee et al. (2021)

Phase 1: Assessing relevance

Category	Target question	Review being assessed
Patients / Population(s)	Overweight/obese children and adolescents (BMI ≥25 kg/m²), aged 7–18 years, no severe comorbidities.	Overweight/obese children and adolescents; 27 randomized controlled trials (RCTs) with unspecified total sample size; age range 7–18 years; mixed-sex cohorts; no exclusion for comorbidities but focused on primary overweight/obesity.
Intervention(s)	Exercise training (aerobic [AE], resistance [RE], combined [AE+RE]); ≥12 weeks, ≥2 sessions/week.	AE/RE/AE+RE: 2–5 sessions/week, 8–36 weeks (average 36 weeks), 30–90 min/session (average 60 min); moderate-to-vigorous intensity; all meet duration/frequency requirements.
Comparator(s)	Calorie restriction (CR) alone.	Controls received CR alone; other treatments balanced between groups; no additional confounding interventions.
Outcome(s)	Inflammatory biomarkers: C-reactive protein (CRP), interleukin-6 (IL-6), tumor necrosis factor-α (TNF-α).	All pre-defined outcomes reported; EX+CR vs. CR: significantly reduces CRP (SMD=-0.16, p=0.02)

Phase 2: Identifying concerns with the review process

Domain	Signaling Questions	Response	Evidence / Rationale	Risk Bias Assessment
DOMAIN 1: STUDY ELIGIBILITY CRITERIA	1.1 Did the review adhere to pre-defined objectives and eligibility criteria?	PY	PRISMA guidelines followed, eligibility criteria described in Methods. No mention of a priori protocol registration (e.g., PROSPERO).	LOW
	1.2 Were the eligibility criteria appropriate for the review question?	Y	Criteria clearly align with review question	
	1.3 Were eligibility criteria unambiguous?	Y	Inclusion/exclusion criteria clearly stated	
	1.4 Were all restrictions in eligibility criteria based on study characteristics appropriate?	PY	No restrictions on study characteristics beyond RCTs, publication date (1990–2020).	
	1.5 Were any restrictions in eligibility criteria based on sources of information appropriate?	Y	Searched published/unpublished studies, no language restrictions, reference lists checked. Appropriate.	
DOMAIN 2: IDENTIFICATION AND SELECTION OF STUDIES	2.1 Did the search include an appropriate range of databases/electronic sources?	PY	PubMed,EMBASE and reference lists. No Cochrane Central or other key databases mentioned.	UNCLEAR
	2.2 Were methods additional to database searching used to identify relevant reports?	Y	Reference lists of previous reviews and meta-analyses were checked.	
	2.3 Were the terms and structure of the search strategy likely to retrieve as many eligible studies as possible?	PY	Search terms included key concepts (exercise, obesity, children, etc.), but full search strategy not provided.	
	2.4 Were restrictions based on date, publication format, or language appropriate?	PY	Date range (1990–2020) reasonable, no language restrictions, included unpublished studies.	
	2.5 Were efforts made to minimise error in selection of studies?	NI	No description of duplicate screening or independent selection process.	
DOMAIN 3: DATA COLLECTION AND STUDY APPRAISAL	3.1 Were efforts made to minimise error in data collection?	NI	No mention of independent data extraction or piloting of forms.	HIGH
	3.2 Were sufficient study characteristics available for interpretation?	Y	Table 1 provides detailed study characteristics.	
	3.3 Were all relevant study results collected for use in the synthesis?	Y	Outcomes specified and extracted; all relevant measures appear included.	
	3.4 Was risk of bias (or methodological quality) formally assessed using appropriate criteria?	N	No assessment of risk of bias in included studies mentioned.	
	3.5 Were efforts made to minimise error in risk of bias assessment?	NI	Not applicable due to lack of RoB assessment.	

DOMAIN 4: SYNTHESIS AND FINDINGS	4.1 Did the synthesis include all studies that it should?	Y	All eligible studies appear included in meta-analyses where appropriate.	UNCLEAR
	4.2 Were all pre-defined analyses reported or departures explained?	NI	No protocol or pre-registered analysis plan mentioned.	
	4.3 Was the synthesis appropriate given the nature of studies?	Y	Random/fixed effects models used appropriately; subgroup/sensitivity analyses conducted.	
	4.4 Was between-study variation (heterogeneity) minimal or addressed in the synthesis?	Y	Heterogeneity assessed (I^2, Q-statistic), sensitivity analyses performed.	
	4.5 Were the findings robust (e.g., funnel plot/sensitivity analyses)?	Y	Funnel plot, Begg & Egger tests, sensitivity analyses reported.	
	4.6 Were biases in primary studies minimal or addressed in the synthesis?	NI	No assessment of primary study biases; thus cannot judge if addressed.	

Phase 3: Judging risk of bias

Summarize the concerns identified during the Phase 2 assessment:

Domain	Concern	Evidence / Rationale
1. Eligibility Criteria	LOW	Criteria clear, appropriate, and well-reported.
2. Identification/Selection	UNCLEAR	Limited database search, no information on independent screening.
3. Data Collection/Appraisal	HIGH	No assessment of risk of bias in included studies; no independent data extraction.
4. Synthesis and Findings	UNCLEAR	No protocol, primary study biases not considered in synthesis.

RISK OF BIAS IN THE REVIEW

Signaling Question	Response	Evidence / Rationale
A. Did the interpretation of findings address all of the concerns identified in Domains 1 to 4?	N	Discussion does not address lack of RoB assessment, limited search, or missing protocol.
B. Was the relevance of identified studies to the review's research question appropriately considered?	Y	Included studies match review question; limitations discussed.
C. Did the reviewers avoid emphasizing results on the basis of their statistical significance?	Y	Both significant and non-significant results reported and interpreted.

Y=YES, PY=PROBABLY YES, PN=PROBABLY NO, N=NO, NI=NO INFORMATION

2.23. Detailed Risk of Bias Assessment for the Systematic Review by Mousa Khalafi et al. (2020)

Phase 1: Assessing relevance

Category	Target question	Review being assessed
Patients / Population(s)	Individuals with metabolic disorders (diabetes, metabolic syndrome, PCOS, NAFLD, overweight/obesity), no age restrictions, inactive/sedentary.	Individuals with metabolic disorders; 29 studies involving 841 participants; age 14.2–60 years; 6 male-only, 9 female-only, 13 mixed-sex cohorts; BMI 22.7–37.4 kg/m²; all inactive/sedentary.
Intervention(s)	High-intensity interval training (HIIT)/sprint interval training (SIT); ≥2 weeks, ≥3 sessions/week, intensity ≥85% HRmax/80% VO_2peak.	HIIT/SIT: running/cycling/elliptical, 3–5 sessions/week, 2–24 weeks (average 12 weeks), 80–100% HRmax/VO_2peak; all meet intensity/duration/frequency requirements.
Comparator(s)	Non-training control (CON) or moderate/low-intensity training (MICT/LIT).	Controls include CON (8 studies) and MICT/LIT (15 studies); 4 studies have both; no concurrent confounding interventions (e.g., supplements/diet).
Outcome(s)	Inflammatory markers (IL-6, TNF-α, CRP) and adipokines (adiponectin, leptin).	All pre-defined outcomes reported; HIIT vs. CON: significantly increases adiponectin (SMD=0.85, p=0.02), decreases leptin (SMD=-1.26, p=0.02) and TNF-α (SMD=-0.57, p=0.003); no effects on IL-6/CRP; moderated by intervention duration/age/BMI.

Phase 2: Identifying concerns with the review process

Domain	Signaling Questions	Response	Evidence / Rationale	Risk Bias Assessment
DOMAIN 1: STUDY ELIGIBILITY CRITERIA	1.1 Did the review adhere to pre-defined objectives and eligibility criteria?	PY	The review states it followed PRISMA and had a priori criteria. However, no protocol registration (e.g., PROSPERO) is mentioned. Pre-defined criteria appear in Methods but not explicitly referenced as a prior protocol.	LOW
	1.2 Were the eligibility criteria appropriate for the review question?	Y	Criteria are highly appropriate: Population (metabolic disorders), Intervention (HIIT/SIT), Comparator (CON/MICT/LIT), Outcomes (inflammatory markers/adipokines), Study design (RCTs/non-RCTs), Minimum duration (≥2 weeks) (PDF P2-3).	
	1.3 Were eligibility criteria unambiguous?	Y	Criteria are explicitly detailed in section 2.2, including specific disorders, marker lists, intensity thresholds (≥85% HRpeak), and exclusion rules (PDF P2-3).	
	1.4 Were all restrictions in eligibility criteria based on study characteristics appropriate?	PY	Restrictions on study design (controlled trials), minimum duration (2 weeks), and intensity are justified for the question. Restriction to English-language studies is a common but potentially limiting choice that could introduce selection bias.	
	1.5 Were any restrictions in eligibility criteria based on sources of information appropriate?	PY	Restriction to English language is stated. While common, this may exclude relevant data. No other source restrictions (e.g., publication status) were inappropriate; the search included unpublished meeting abstracts and trial registries (PDF P2-3).	
DOMAIN 2: IDENTIFICATION AND SELECTION OF STUDIES	2.1 Did the search include an appropriate range of databases/electronic sources?	PN	Only two databases were searched. This is unlikely to be comprehensive for exercise science literature.	HIGH
	2.2 Were methods additional to database searching used to identify relevant reports?	Y	The authors searched reference lists of retrieved articles and sought unpublished studies via conference abstracts and trial registries (ICTGP, ClinicalTrials.gov).	
	2.3 Were the terms and structure of the search strategy likely to retrieve as many eligible studies as possible?	PY	While search terms are provided and relevant, the limitation to only two databases significantly reduces confidence that all eligible studies were identified.	
	2.4 Were restrictions based on date, publication format, or language appropriate?	PY	Restriction to English language is stated.No date restriction	
	2.5 Were efforts made to minimise error in selection of studies?	Y	Two independent researchers screened titles/abstracts and full texts. Disagreements resolved by discussion.	
DOMAIN 3: DATA COLLECTION AND STUDY APPRAISAL	3.1 Were efforts made to minimise error in data collection?	Y	Two independent reviewers extracted data using a pre-defined form. Disagreements were resolved by discussion	HIGH
	3.2 Were sufficient study characteristics available for interpretation?	Y	Table 1 provides detailed participant and study characteristics. Table 2 provides detailed intervention characteristics	
	3.3 Were all relevant study results collected for use in the synthesis?	Y	Relevant outcome data (marker levels post-intervention) for all pre-specified inflammatory markers were extracted for synthesis	
	3.4 Was risk of bias (or methodological quality) formally assessed using appropriate	PN	Quality was assessed using "an eight-item checklist adapted from the PRISMA statement". This is a non-standard tool. The supplementary Word file confirms this custom checklist was used.	

	criteria?			
	3.5 Were efforts made to minimise error in risk of bias assessment?	Y	Two reviewers (M Kh and MES) independently assessed the quality assessment of studies.	
DOMAIN 4: SYNTHESIS AND FINDINGS	4.1 Did the synthesis include all studies that it should?	Y	Based on the included studies identified by their search, the meta-analysis includes all eligible studies for each outcome comparison.	HIGH
	4.2 Were all pre-defined analyses reported or departures explained?	PY	No protocol is available for comparison. The analysis plan described in the methods (random-effects models, subgroup/meta-regression for moderators, sensitivity analysis, publication bias) appears to have been followed.	
	4.3 Was the synthesis appropriate given the nature of studies?	PY	A random-effects model was appropriately chosen due to expected heterogeneity. Quantitative synthesis was justified as >5 studies per outcome. Subgrouping by comparator was sensible. Significant heterogeneity was present and acknowledged.	
	4.4 Was between-study variation (heterogeneity) minimal or addressed in the synthesis?	PY	High heterogeneity (I^2) was reported for many analyses. The authors explored sources via meta-regression and performed sensitivity analyses .	
	4.5 Were the findings robust (e.g., funnel plot/sensitivity analyses)?	Y	Sensitivity analyses were conducted. Publication bias was assessed via funnel plots and Egger's test, with no significant bias found for most outcomes.	
	4.6 Were biases in primary studies minimal or addressed in the synthesis?	PN	The quality assessment tool was inadequate, so the risk of bias in primary studies was not properly evaluated. While a sensitivity analysis excluding "low quality" studies is mentioned in Table 3 notes , the basis for defining "low quality" is flawed. Therefore, biases in primary studies were not reliably addressed in the synthesis.	

Phase 3: Judging risk of bias

Summarize the concerns identified during the Phase 2 assessment:

Domain	Concern	Evidence / Rationale
1. Eligibility Criteria	LOW	Eligibility criteria were clearly defined, appropriate for the research question, and mostly unambiguous. Minor concerns exist due to the lack of a registered protocol and the restriction to English-language studies, but these are not severe enough to raise a high risk of bias in this domain.
2. Identification/Selection	HIGH	The search strategy was limited to only two electronic databases (PubMed and Scopus),
3. Data Collection/Appraisal	HIGH	The review used a non-standard, non-validated quality assessment tool
4. Synthesis and Findings	HIGH	Because the risk of bias in the primary studies was not reliably assessed (due to the flawed tool).

RISK OF BIAS IN THE REVIEW

Signaling Question	Response	Evidence / Rationale
A. Did the interpretation of findings address all of the concerns identified in Domains 1 to 4?	PN	the limited search strategy or the use of an inappropriate quality assessment tool.
B. Was the relevance of identified studies to the review's research question appropriately considered?	Y	The review question and inclusion criteria were clearly focused on metabolic disorders. The discussion interprets findings specifically within the context of these populations.
C. Did the reviewers avoid emphasizing results on the basis of their statistical significance?	Y	The abstract and conclusions report both statistically significant (TNF-α, leptin, adiponectin) and non-significant (IL-6, CRP) findings. Effect sizes and confidence intervals are reported throughout.

Y=YES, PY=PROBABLY YES, PN=PROBABLY NO, N=NO, NI=NO INFORMATION

2.24. Detailed Risk of Bias Assessment for the Systematic Review by Felice Sirico et al. (2018)

Phase 1: Assessing relevance

Category	Target question	Review being assessed
Patients / Population(s)	Obese children/adolescents (≤18 years old, BMI >95th percentile for age/sex), no concurrent metabolic diseases.	Obese children/adolescents; 7 randomized controlled trials (RCTs) with 250 participants; mean age ≤18 years; BMI >95th percentile; mixed-sex cohorts; no concurrent dietary interventions or metabolic comorbidities.
Intervention(s)	Physical exercise (aerobic, resistance, concurrent) without concomitant dietary intervention; supervised, ≥12 weeks.	Exercise interventions: aerobic/resistance/concurrent, 3 sessions/week, 12–unspecified weeks, supervised; no dietary intervention; all meet core intervention requirements.
Comparator(s)	No lifestyle modification (no exercise, no dietary change).	Controls received no exercise/dietary intervention; no confounding interventions.
Outcome(s)	Adipokines (adiponectin, leptin) and inflammatory markers (IL-6, CRP, TNF-α).	All pre-defined outcomes reported; exercise significantly increases adiponectin (SMD=0.69, p=0.04), decreases leptin (SMD=-1.13, p=0.004) and IL-6 (SMD=-0.84, p=0.007); CRP shows a non-significant reduction trend.

Phase 2: Identifying concerns with the review process

Domain	Signaling Questions	Response	Evidence / Rationale	Risk Bias Assessment
DOMAIN 1: STUDY ELIGIBILITY CRITERIA	1.1 Did the review adhere to pre-defined objectives and eligibility criteria?	PY	The review states it followed PRISMA and had a priori criteria. However, no protocol registration (e.g., PROSPERO) is mentioned. Pre-defined criteria appear in Methods but not explicitly referenced as a prior protocol.	LOW
	1.2 Were the eligibility criteria appropriate for the review question?	Y	Criteria fit the research question: obese children (BMI >95th), exercise intervention, no diet change, inflammatory markers.	
	1.3 Were eligibility criteria unambiguous?	Y	Clear inclusion/exclusion criteria described in Methods.	
	1.4 Were all restrictions in eligibility criteria based on study characteristics appropriate?	Y	No restrictions on study characteristics beyond RCT/non-RCT.	
	1.5 Were any restrictions in eligibility criteria based on sources of information appropriate?	PY	Language restriction (English only) may introduce bias by excluding relevant non-English studies.	
DOMAIN 2: IDENTIFICATION AND SELECTION OF STUDIES	2.1 Did the search include an appropriate range of databases/electronic sources?	Y	5 databases searched (PubMed, Web of Science, Medline, Scopus, CINAHL), plus reference lists, conference abstracts, and trial registries.	LOW
	2.2 Were methods additional to database searching used to identify relevant reports?	Y	reference scanning, conference abstracts, and clinical trial registries were used.	
	2.3 Were the terms and structure of the search strategy likely to retrieve as many eligible studies as possible?	Y	Detailed search strategy provided (Supplementary Table S2), using comprehensive terms and Boolean logic.	
	2.4 Were restrictions based on date, publication format, or language appropriate?	PY	English-only restriction may miss relevant studies and introduce language bias. Date restriction (inception to March 2017) is acceptable.	
	2.5 Were efforts made to minimise error in selection of studies?	Y	Two reviewers independently screened titles/abstracts and full texts, with third reviewer resolving disagreements.	
DOMAIN 3: DATA COLLECTION AND STUDY APPRAISAL	3.1 Were efforts made to minimise error in data collection?	Y	Two reviewers independently extracted data using a pre-designed form, with third reviewer verification.	LOW
	3.2 Were sufficient study characteristics available for interpretation?	Y	Table 1 provides adequate study characteristics (participants, intervention, outcomes).	
	3.3 Were all relevant study results collected for use in the synthesis?	Y	All relevant outcome data (adiponectin, leptin, IL-6, etc.) were extracted.	
	3.4 Was risk of bias (or methodological quality) formally assessed using appropriate criteria?	Y	Cochrane Risk of Bias tool was used for RCTs.	
	3.5 Were efforts made to minimise error in risk of bias assessment?	Y	Two reviewers independently assessed risk of bias, with third reviewer adjudication.	

DOMAIN 4: SYNTHESIS AND FINDINGS	4.1 Did the synthesis include all studies that it should?	PY	One study (Monteiro et al.) was excluded from quantitative synthesis due to missing numerical data. Authors attempted to contact authors, but exclusion may still introduce bias.	LOW
	4.2 Were all pre-defined analyses reported or departures explained?	PY	Planned analyses (meta-analysis, subgroup, sensitivity) were reported. No protocol was referenced to confirm all pre-defined analyses.	
	4.3 Was the synthesis appropriate given the nature of studies?	Y	Random-effects model used due to expected heterogeneity; SMD used for continuous outcomes.	
	4.4 Was between-study variation (heterogeneity) minimal or addressed in the synthesis?	Y	Heterogeneity was assessed and explored via sensitivity analyses and meta-regression.	
	4.5 Were the findings robust (e.g., funnel plot/sensitivity analyses)?	Y	Sensitivity analyses performed (excluding high-risk studies, etc.); funnel plot shown (Supplementary Fig. S1), though limited by small number of studies (<10).	
	4.6 Were biases in primary studies minimal or addressed in the synthesis?	Y	Risk of bias in primary studies was assessed and considered in sensitivity analyses.	

Phase 3: Judging risk of bias

Summarize the concerns identified during the Phase 2 assessment:

Domain	Concern	Evidence / Rationale
1. Eligibility Criteria	LOW	Language restriction (English only) may introduce bias, and lack of explicit protocol registration raises concerns about adherence to pre-defined criteria.
2. Identification/Selection	LOW	English-only search restriction may lead to missing relevant non-English studies, introducing language bias.
3. Data Collection/Appraisal	LOW	Rigorous methods were used for data extraction and appraisal, with independent duplicate processes and appropriate tools.
4. Synthesis and Findings	LOW	Appropriate synthesis methods were used, heterogeneity was addressed, and sensitivity analyses were performed to test robustness.

RISK OF BIAS IN THE REVIEW

Signaling Question	Response	Evidence / Rationale
A. Did the interpretation of findings address all of the concerns identified in Domains 1 to 4?	PY	The risk of bias for all four domains in Phase 2 is "low".
B. Was the relevance of identified studies to the review's research question appropriately considered?	PY	Included studies directly addressed the research question (exercise in obese children, inflammatory markers).
C. Did the reviewers avoid emphasizing results on the basis of their statistical significance?	Y	Results are presented with confidence intervals, and discussion contextualizes findings without overemphasis on statistical significance.

Y=YES, PY=PROBABLY YES, PN=PROBABLY NO, N=NO, NI=NO INFORMATION

2.25. Detailed Risk of Bias Assessment for the Systematic Review by Antonio García-Hermoso et al. (2016)

Phase 1: Assessing relevance

Category	Target question	Review being assessed
Patients / Population(s)	Overweight/obese children and adolescents (6–18 years old), no concurrent dietary interventions.	Overweight/obese children/adolescents; 9 randomized controlled trials (RCTs) involving 427 participants (219 intervention, 208 control); age 6–18 years; 2 male-only, 1 female-only, 6 mixed-sex cohorts; BMI defined by percentile/WHO/nation-specific criteria.
Intervention(s)	Physical exercise (aerobic, resistance, multisports) without concomitant dietary intervention; ≥8 weeks.	Exercise interventions: multisports/soccer/dance/walking/cycling, 2–6 sessions/week, 8–24 weeks, 20–90 min/session; no dietary co-interventions; all meet core intervention requirements.
Comparator(s)	No structured exercise or dietary restriction (usual care control).	Controls received no exercise/dietary intervention; no confounding interventions.
Outcome(s)	Circulating C-reactive protein (CRP) levels.	Primary outcome reported: exercise shows a non-significant reduction trend in CRP (WMD=-0.72 mg/l, 95% CI: -1.52 to 0.08, p=0.077); subgroup analyses for weight status/age/duration/frequency/session length show no significant moderators.

Phase 2: Identifying concerns with the review process

Domain	Signaling Questions	Response	Evidence / Rationale	Risk bias assessment
DOMAIN 1: STUDY ELIGIBILITY CRITERIA	1.1 Did the review adhere to pre-defined objectives and eligibility criteria?	PY	The review states that it followed PRISMA guidelines and pre-defined inclusion criteria.	LOW
	1.2 Were the eligibility criteria appropriate for the review question?	PY	Criteria were appropriate but broad in terms of obesity definitions and exercise types, which may introduce heterogeneity.	
	1.3 Were eligibility criteria unambiguous?	PY	Overweight/obesity definitions varied across included studies, and no unified diagnostic criteria were required.	
	1.4 Were all restrictions in eligibility criteria based on study characteristics appropriate?	Y	Only RCTs were included	
	1.5 Were any restrictions in eligibility criteria based on sources of information appropriate?	Y	No language or publication status restrictions were applied.	
DOMAIN 2: IDENTIFICATION AND SELECTION OF STUDIES	2.1 Did the search include an appropriate range of databases/electronic sources?	Y	Seven databases were searched, including grey literature sources (conference abstracts, trial registries).	LOW
	2.2 Were methods additional to database searching used to identify relevant reports?	Y	Manual searching of reference lists was performed.	
	2.3 Were the terms and structure of the search strategy likely to retrieve as many eligible studies as possible?	PY	Search terms were broad, but no full search strategy is provided in the paper to verify completeness.	
	2.4 Were restrictions based on date, publication format, or language appropriate?	Y	No restrictions on language or publication date; all formats considered.	
	2.5 Were efforts made to minimise error in selection of studies?	Y	Two reviewers independently screened titles/abstracts and full texts. Disagreements resolved by consensus or third reviewer.	
DOMAIN 3: DATA COLLECTION AND STUDY APPRAISAL	3.1 Were efforts made to minimise error in data collection?	Y	Two reviewers independently extracted data using a pre-defined form. Disagreements resolved by consensus or third reviewer.	LOW
	3.2 Were sufficient study characteristics available for interpretation?	PY	Key study characteristics are provided in Table 1	
	3.3 Were all relevant study results collected for use in the synthesis?	Y	Pre- and post-intervention CRP data were extracted for all included studies.	
	3.4 Was risk of bias (or methodological quality) formally assessed using appropriate criteria?	Y	The Delphi List was used for quality assessment, which is appropriate for RCTs.	
	3.5 Were efforts made to minimise error in risk of bias assessment?	Y	Two reviewers independently assessed risk of bias. Disagreements resolved by consensus or third reviewer.	

DOMAIN 4: SYNTHESIS AND FINDINGS	4.1 Did the synthesis include all studies that it should?	Y	All nine eligible studies were included in the meta-analysis.	LOW
	4.2 Were all pre-defined analyses reported or departures explained?	Y	Subgroup analyses were conducted, protocol (e.g., PROSPERO) is referenced to confirm pre-specified plans.	
	4.3 Was the synthesis appropriate given the nature of studies?	Y	High heterogeneity ($I^2 = 69\%$) was noted, but random-effects model was appropriately used.	
	4.4 Was between-study variation (heterogeneity) minimal or addressed in the synthesis?	PY	Heterogeneity was high and only partially explored through subgroup analyses	
	4.5 Were the findings robust (e.g., funnel plot/sensitivity analyses)?	Y	Sensitivity analysis and funnel plot/Egger's test were conducted, showing stability and no significant publication bias.	
	4.6 Were biases in primary studies minimal or addressed in the synthesis?	PY	No sensitivity analysis excluding high-risk studies was performed.	

Phase 3: Judging risk of bias

Summarize the concerns identified during the Phase 2 assessment:

Domain	Concern	Evidence / Rationale
1. Eligibility Criteria	LOW	Clear diagnostic criteria
2. Identification/Selection	LOW	Comprehensive search and rigorous screening.
3. Data Collection/Appraisal	LOW	Systematic data extraction and quality assessment.
4. Synthesis and Findings	LOW	High heterogeneity, lack of protocol adherence, and inadequate handling of primary study biases.

RISK OF BIAS IN THE REVIEW

Signaling Question	Response	Evidence / Rationale
A. Did the interpretation of findings address all of the concerns identified in Domains 1 to 4?	PY	The discussion acknowledges some limitations
B. Was the relevance of identified studies to the review's research question appropriately considered?	PY	Studies generally matched the PICO, but variability in populations and interventions is acknowledged.
C. Did the reviewers avoid emphasizing results on the basis of their statistical significance?	Y	The conclusion appropriately notes a non-significant trend and calls for more research.

Y=YES, PY=PROBABLY YES, PN=PROBABLY NO, N=NO, NI=NO INFORMATION

References

1. Zalagkitis C, Philippou A, Karatzanos E, Metsios GS, Dinas PC. Combined effects of physical activity and diet on chronic inflammation of overweight/obese children and adolescents: A systematic review and meta-analysis. J Sports Sci. 2025;43(22):2841-57. https://doi.org/10.1080/02640414.2025.2561349

2. Wang J, Fan S, Wang J. Resistance training enhances metabolic and muscular health and reduces systemic inflammation in middle-aged and older adults with type 2 diabetes: A meta-analysis. Diabetes Res Clin Pract. 2025;229. https://doi.org/10.1016/j.diabres.2025.112941

3. Hernández-Martínez J, Vasquez-Carrasco E, Cid-Calfucura I, et al. Effects of concurrent training on biomarkers, morphological variables, and physical performance in people with sarcopenic obesity: A meta-analysis with meta-regression. Medicina. 2025;61(9). https://doi.org/10.3390/medicina61091697

4. Tan L, Mei JY, Tang RH, et al. Can exercise as a complementary technique manage inflammatory markers in women with breast cancer who are overweight and obese? A systematic review and meta-analysis. Complement Ther Med. 2025 MAR;88. https://doi.org/10.1016/j.ctim.2024.103119

5. Silva FM, Duarte-Mendes P, Teixeira AM, Soares CM, Ferreira JP. The effects of combined exercise training on glucose metabolism and inflammatory markers in sedentary adults: A systematic review and meta-analysis. Sci Rep. 2024 JAN 22;14(1). https://doi.org/10.1038/s41598-024-51832-y

6. Li LY, Li SM, Pang BX, Wei JP, Wang QH. Effects of exercise training on glucose metabolism indicators and inflammatory markers in obese children and adolescents: A meta-analysis. World J Diabetes. 2024 JUN 15;15(6). https://doi.org/10.4239/wjd.v15.i6.1353

7. Al-Mhanna SB, Batrakoulis A, Norhayati NM, et al. Combined aerobic and resistance training improves body composition, alters cardiometabolic risk, and ameliorates cancer-related indicators in breast cancer patients and survivors with overweight/obesity: A systematic review and meta-analysis of randomized controlled trials. J Sports Sci Med. 2024;23(2):366-95. https://doi.org/10.52082/jssm.2024.366

8. Al-Mhanna SB, Batrakoulis A, Wan Ghazali WS, et al. Effects of combined aerobic and resistance training on glycemic control, blood pressure, inflammation, cardiorespiratory fitness and quality of life in patients with type 2 diabetes and overweight/obesity: A systematic review and meta-analysis. PeerJ. 2024;12:e17525. https://doi.org/10.7717/peerj.17525

9. Guo YQ, Qian HN, Xin XY, Liu QL. Effects of different exercise modalities on inflammatory markers in the obese and overweight populations: Unraveling the mystery of exercise and inflammation. Front Physiol. 2024 JUN 12;15. https://doi.org/10.3389/fphys.2024.1405094

10. Malandish A, Gulati M. The impacts of exercise interventions on inflammaging markers in overweight/obesity patients with heart failure: A systematic review and meta-analysis of randomized controlled trials. IJC Heart Vasc. 2023;47. https://doi.org/10.1016/j.ijcha.2023.101234

11. Dragoumani K, Troumbis A, Bacopoulou F, Chrousos G. Childhood and adolescent obesity with somatic indicators of stress, inflammation, and dysmetabolism before and after intervention: A meta-analysis. J Pers Med. 2023 SEP;13(9). https://doi.org/10.3390/jpm13091322

12. Tan L, Yan WH, Yang WL, et al. Effect of exercise on inflammatory markers in postmenopausal women with overweight and obesity: A systematic review and meta-analysis. Exp Gerontol. 2023 NOV;183. https://doi.org/10.1016/j.exger.2023.112310

13. Al-Mhanna SB, Rocha-Rodriguesc S, Mohamed M, et al. Effects of combined aerobic exercise and diet on cardiometabolic health in patients with obesity and type 2 diabetes: A systematic review and meta-analysis. BMC Sports Sci Med Rehabil. 2023 DEC 4;15(1). https://doi.org/10.1186/s13102-023-00766-5

14. Del Rosso S, Baraquet ML, Barale A, et al. Long-term effects of different exercise training modes on cytokines and adipokines in individuals with overweight/obesity and cardiometabolic diseases: A systematic review, meta-analysis, and meta-regression of randomized controlled trials. Obes Rev. 2023 Jun;24(6):e13564. https://doi.org/10.1111/obr.13564

15. Rahimi GRM, Yousefabadi HA, Niyazi A, Rahimi NM, Alikhajeh Y. Effects of lifestyle intervention on inflammatory markers and waist circumference in overweight/obese adults with metabolic syndrome: A systematic review and meta-analysis of randomized controlled trials. Biol Res Nurs. 2022 Jan;24(1):94-105. https://doi.org/10.1177/10998004211044754

16. Zhao HT, Cheng RH, Teng J, et al. A meta-analysis of the effects of different training modalities on the inflammatory response in adolescents with obesity. Int J Environ Res Public Health. 2022 OCT;19(20). https://doi.org/10.3390/ijerph192013224

17. Hejazi K, Wong A. Effects of exercise training on inflammatory and cardiometabolic health markers in overweight and obese adults: A systematic review and meta-analysis of randomized controlled trials. J Sports Med Phys Fitness. 2023 Feb;63(2):345-59. https://doi.org/10.23736/s0022-4707.22.14103-4

18. Hejazi K, Rahimi GRM, Rosenkranz SK. Effects of exercise training on inflammatory and cardiometabolic risk biomarkers in patients with type 2 diabetes mellitus: A systematic review and meta-analysis of randomized controlled trials. Biol Res Nurs. 2023 APR;25(2):250-66. https://doi.org/10.1177/10998004221132841

19. Khalafi M, Symonds ME, Akbari A. The impact of exercise training versus caloric restriction on inflammation markers: A systemic review and meta-analysis. Crit Rev Food Sci Nutr. 2022;62(15):4226-41. https://doi.org/10.1080/10408398.2021.1873732

20. Ding YJ, Xu X. Anti-inflammatory effect of exercise training through reducing inflammasome activation-related inflammatory cytokine levels in overweight/obese populations: A systematic review and meta-analysis. COMPLEMENTARY THERAPIES IN CLINICAL PRACTICE. 2022 NOV;49. https://doi.org/10.1016/j.ctcp.2022.101656

21. Liu Y, Hong F, Reddy VR, et al. Calorie restriction with exercise intervention improves inflammatory response in overweight and obese adults: A systematic review and meta-analysis. Front Physiol. 2021;12:754731. https://doi.org/10.3389/fphys.2021.754731

22. Lee J. Influences of exercise interventions on overweight and obesity in children and adolescents. Public Health Nurs. 2021 May;38(3):502-16. https://doi.org/10.1111/phn.12862

23. Khalafi M, Symonds ME. The impact of high-intensity interval training on inflammatory markers in metabolic disorders: A meta-analysis. Scand J Med Sci Sports. 2020;30(11):2020-36. https://doi.org/10.1111/sms.13754

24. Sirico F, Bianco A, D'Alicandro G, et al. Effects of physical exercise on adiponectin, leptin, and inflammatory markers in childhood obesity: Systematic review and meta-analysis. Child Obes. 2018;14(4):207-17. https://doi.org/10.1089/chi.2017.0269

25. García Hermoso A, Sánchez–López M, Escalante Y, Saavedra JM, Martinez Vizcaino V. Exercise-based interventions and c-reactive protein in overweight and obese youths: A meta-analysis of randomized controlled trials. Pediatr Res. 2016;79(4):522-7. https://doi.org/10.1038/pr.2015.274
